# Global investments in pandemic preparedness and COVID-19: development assistance and domestic spending on health between 1990 and 2026

**DOI:** 10.1016/S2214-109X(23)00007-4

**Published:** 2023-01-24

**Authors:** Global Burden of Disease 2021 Health Financing Collaborator Network, Angela E Micah, Kayleigh Bhangdia, Ian E Cogswell, Dylan Lasher, Brendan Lidral-Porter, Emilie R Maddison, Trang Nhu Ngoc Nguyen, Nishali Patel, Paola Pedroza, Juan Solorio, Hayley Stutzman, Golsum Tsakalos, Yifeng Wang, Wesley Warriner, Yingxi Zhao, Bianca S Zlavog, Cristiana Abbafati, Jaffar Abbas, Mohsen Abbasi-Kangevari, Zeinab Abbasi-Kangevari, Michael Abdelmasseh, Deldar Morad Abdulah, Aidin Abedi, Kedir Hussein Abegaz, E S Abhilash, Richard Gyan Aboagye, Hassan Abolhassani, Michael R M Abrigo, Hiwa Abubaker Ali, Eman Abu-Gharbieh, Mohammed Hussien Adem, Muhammad Sohail Afzal, Ali Ahmadi, Haroon Ahmed, Tarik Ahmed Rashid, Budi Aji, Hossein Akbarialiabad, Yibeltal Akelew, Hanadi Al Hamad, Khurshid Alam, Fahad Mashhour Alanezi, Turki M Alanzi, Mohammed Khaled Al-Hanawi, Robert Kaba Alhassan, Syed Mohamed Aljunid, Sami Almustanyir, Rajaa M Al-Raddadi, Nelson Alvis-Guzman, Nelson J Alvis-Zakzuk, Azmeraw T Amare, Edward Kwabena Ameyaw, Mostafa Amini-Rarani, Hubert Amu, Robert Ancuceanu, Tudorel Andrei, Sumadi Lukman Anwar, Francis Appiah, Muhammad Aqeel, Jalal Arabloo, Morteza Arab-Zozani, Aleksandr Y Aravkin, Olatunde Aremu, Raphael Taiwo Aruleba, Seyyed Shamsadin Athari, Leticia Avila-Burgos, Martin Amogre Ayanore, Samad Azari, Atif Amin Baig, Abere Tilahun Bantie, Amadou Barrow, Pritish Baskaran, Sanjay Basu, Abdul-Monim Mohammad Batiha, Bernhard T Baune, Zombor Berezvai, Nikha Bhardwaj, Pankaj Bhardwaj, Sonu Bhaskar, Micheal Kofi Boachie, Virginia Bodolica, João Silva Botelho Botelho, Dejana Braithwaite, Nicholas J K Breitborde, Reinhard Busse, Lucero Cahuana-Hurtado, Ferrán Catalá-López, Collins Chansa, Jaykaran Charan, Vijay Kumar Chattu, Simiao Chen, Isaac Sunday Chukwu, Omid Dadras, Lalit Dandona, Rakhi Dandona, Abdollah Dargahi, Sisay Abebe Debela, Edgar Denova-Gutiérrez, Belay Desye, Samath Dhamminda Dharmaratne, Nancy Diao, Linh Phuong Doan, Milad Dodangeh, Wendel Mombaque dos Santos, Leila Doshmangir, John Dube, Ebrahim Eini, Maysaa El Sayed Zaki, Maha El Tantawi, Daniel Berhanie Enyew, Sharareh Eskandarieh, Mohamad Ezati Asar, Adeniyi Francis Fagbamigbe, Emerito Jose A Faraon, Ali Fatehizadeh, Hamed Fattahi, Ginenus Fekadu, Florian Fischer, Nataliya A Foigt, Kayode Raphael Fowobaje, Alberto Freitas, Takeshi Fukumoto, Nancy Fullman, Peter Andras Gaal, Amiran Gamkrelidze, M A Garcia-Gordillo, Mesfin Gebrehiwot, Urge Gerema, Mansour Ghafourifard, Seyyed-Hadi Ghamari, Reza Ghanbari, Ahmad Ghashghaee, Ali Gholamrezanezhad, Mahaveer Golechha, Davide Golinelli, Yitayal Ayalew Goshu, Girma Garedew Goyomsa, Avirup Guha, Damitha Asanga Gunawardane, Bhawna Gupta, Samer Hamidi, Harapan Harapan, Reza Hashempour, Khezar Hayat, Golnaz Heidari, Ileana Heredia-Pi, Claudiu Herteliu, Demisu Zenbaba Heyi, Kamal Hezam, Yuta Hiraike, Mbuzeleni Mbuzeleni Hlongwa, Ramesh Holla, Mohammad Enamul Hoque, Mehdi Hosseinzadeh, Sorin Hostiuc, Salman Hussain, Olayinka Stephen Ilesanmi, Mustapha Immurana, Arnaud Iradukunda, Nahlah Elkudssiah Ismail, Gaetano Isola, Linda Merin J, Mihajlo Jakovljevic, Mahsa Jalili, Manthan Dilipkumar Janodia, Tahereh Javaheri, Sathish Kumar Jayapal, Digisie Mequanint Jemere, Tamas Joo, Nitin Joseph, Jacek Jerzy Jozwiak, Mikk Jürisson, Billingsley Kaambwa, Vidya Kadashetti, Rajendra Kadel, Dler Hussein Kadir, Laleh R Kalankesh, Rajesh Kamath, Himal Kandel, Rami S Kantar, Shama D Karanth, Ibraheem M Karaye, Salah Eddin Karimi, Bekalu Getnet Kassa, Gbenga A Kayode, Leila Keikavoosi-Arani, Vikash Ranjan Keshri, Cumali Keskin, Yousef Saleh Khader, Morteza Abdullatif Khafaie, Himanshu Khajuria, Hamid Reza Khayat Kashani, Zemene Demelash Kifle, Hanna Kim, Jihee Kim, Min Seo Kim, Yun Jin Kim, Adnan Kisa, Stefan Kohler, Farzad Kompani, Soewarta Kosen, Sindhura Lakshmi Koulmane Laxminarayana, Ai Koyanagi, Kewal Krishan, Dian Kusuma, Judit Lám, Demetris Lamnisos, Anders O Larsson, Sang-woong Lee, Shaun Wen Huey Lee, Wei-Chen Lee, Yo Han Lee, Jacopo Lenzi, Lee-Ling Lim, László Lorenzovici, Rafael Lozano, Vanessa Sintra Machado Machado, Farzan Madadizadeh, Mohammed Magdy Abd El Razek, Razzagh Mahmoudi, Azeem Majeed, Mohammad-Reza Malekpour, Ana Laura Manda, Borhan Mansouri, Mohammad Ali Mansournia, Lorenzo Giovanni Mantovani, Carlos Alberto Marrugo Arnedo, Miquel Martorell, Ali Masoud, Elezebeth Mathews, Richard James Maude, Enkeleint A Mechili, Entezar Mehrabi Nasab, José João João Mendes Mendes, Atte Meretoja, Tuomo J Meretoja, Mohamed Kamal Mesregah, Tomislav Mestrovic, Andreea Mirica, Erkin M Mirrakhimov, Mizan Kiros Mirutse, Moonis Mirza, Mohammad Mirza-Aghazadeh-Attari, Awoke Misganaw, Marcello Moccia, Javad Moghadasi, Esmaeil Mohammadi, Mokhtar Mohammadi, Abdollah Mohammadian-Hafshejani, Marita Mohammadshahi, Shafiu Mohammed, Mohammad Mohseni, Ali H Mokdad, Lorenzo Monasta, Elias Mossialos, Ebrahim Mostafavi, Haleh Mousavi Isfahani, Christine Mpundu-Kaambwa, Shruti Murthy, Saravanan Muthupandian, Ahamarshan Jayaraman Nagarajan, Kovin S Naidoo, Mukhammad David Naimzada, Vinay Nangia, Atta Abbas Naqvi, Biswa Prakash Nayak, Rawlance Ndejjo, Trang Huyen Nguyen, Nafise Noroozi, Jean Jacques Noubiap, Khan M Nuruzzaman, Chimezie Igwegbe Nzoputam, Ogochukwu Janet Nzoputam, Bogdan Oancea, Felix Chukwudi Abrahams Obi, Abiola Ogunkoya, In-Hwan Oh, Osaretin Christabel Okonji, Andrew T Olagunju, Tinuke O Olagunju, Babayemi Oluwaseun Olakunde, Ahmed Omar Bali, Obinna E Onwujekwe, John Nelson Opio, Adrian Otoiu, Nikita Otstavnov, Stanislav S Otstavnov, Mayowa O Owolabi, Tamás Palicz, Raffaele Palladino, Adrian Pana, Tarang Parekh, Deepak Kumar Pasupula, Jay Patel, George C Patton, Uttam Paudel, Mihaela Paun, Shrikant Pawar, Simone Perna, Navaraj Perumalsamy, Ionela-Roxana Petcu, Zahra Zahid Piracha, Mohsen Poursadeqiyan, Naeimeh Pourtaheri, Sergio I Prada, Sima Rafiei, Pankaja Raghav Raghav, Fakher Rahim, Mohammad Hifz Ur Rahman, Mosiur Rahman, Amir Masoud Rahmani, Chhabi Lal Ranabhat, Temam Beshir Raru, Sina Rashedi, Mohammad-Mahdi Rashidi, Ramin Ravangard, Salman Rawaf, Reza Rawassizadeh, Elrashdy Moustafa Mohamed Redwan, Robert C Reiner, Andre M N Renzaho, Maryam Rezaei, Nazila Rezaei, Mavra A Riaz, Jefferson Antonio Buendia Rodriguez, Aly M A Saad, Basema Saddik, Saeid Sadeghian, Mohammad Reza Saeb, Umar Saeed, Maitreyi Sahu, Morteza Saki, Payman Salamati, Hedayat Salari, Sana Salehi, Abdallah M Samy, Juan Sanabria, Francesco Sanmarchi, João Vasco Santos, Milena M Santric-Milicevic, Bruno Piassi Sao Jose, Yaser Sarikhani, Brijesh Sathian, Maheswar Satpathy, Miloje Savic, Yaser Sayadi, Falk Schwendicke, Subramanian Senthilkumaran, Sadaf G Sepanlou, Edson Serván-Mori, Naomi Setshegetso, Allen Seylani, Saeed Shahabi, Masood Ali Shaikh, Murad Ziyaudinovich Shakhmardanov, Mohd Shanawaz, Mequannent Melaku Sharew Sharew, Nigussie Tadesse Sharew, Rajesh Sharma, Maryam Shayan, Aziz Sheikh, Suchitra M Shenoy, Adithi Shetty, Pavanchand H Shetty, K M Shivakumar, Luís Manuel Lopes Rodrigues Silva, Wudneh Simegn, Jasvinder A Singh, Kuldeep Singh, Natia Skhvitaridze, Valentin Yurievich Skryabin, Anna Aleksandrovna Skryabina, Bogdan Socea, Yonatan Solomon, Suhang Song, Simona Cătălina Ștefan, Muhammad Suleman, Rafael Tabarés-Seisdedos, Nathan Y Tat, Vivian Y Tat, Belay Negash Tefera, Ales Tichopad, Ruoyan Tobe-Gai, Marcos Roberto Tovani-Palone, Lorainne Tudor Car, Derara Girma Tufa, Tommi Juhani Vasankari, Milena Vasic, Dominique Vervoort, Vasily Vlassov, Bay Vo, Linh Gia Vu, Yasir Waheed, Richard G Wamai, Cong Wang, Gizachew Tadesse Wassie, Nuwan Darshana Wickramasinghe, Sanni Yaya, Arzu Yigit, Vahit Yiğit, Naohiro Yonemoto, Mustafa Z Younis, Chuanhua Yu, Ismaeel Yunusa, Leila Zaki, Burhan Abdullah Zaman, Alireza Zangeneh, Ali Zare Dehnavi, Mikhail Sergeevich Zastrozhin, Wu Zeng, Zhi-Jiang Zhang, Liesl J Zuhlke, Yves Miel H Zuniga, Simon I Hay, Christopher J L Murray, Joseph L Dieleman

## Abstract

**Background:**

The COVID-19 pandemic highlighted gaps in health surveillance systems, disease prevention, and treatment globally. Among the many factors that might have led to these gaps is the issue of the financing of national health systems, especially in low-income and middle-income countries (LMICs), as well as a robust global system for pandemic preparedness. We aimed to provide a comparative assessment of global health spending at the onset of the pandemic; characterise the amount of development assistance for pandemic preparedness and response disbursed in the first 2 years of the COVID-19 pandemic; and examine expectations for future health spending and put into context the expected need for investment in pandemic preparedness.

**Methods:**

In this analysis of global health spending between 1990 and 2021, and prediction from 2021 to 2026, we estimated four sources of health spending: development assistance for health (DAH), government spending, out-of-pocket spending, and prepaid private spending across 204 countries and territories. We used the Organisation for Economic Co-operation and Development (OECD)'s Creditor Reporting System (CRS) and the WHO Global Health Expenditure Database (GHED) to estimate spending. We estimated development assistance for general health, COVID-19 response, and pandemic preparedness and response using a keyword search. Health spending estimates were combined with estimates of resources needed for pandemic prevention and preparedness to analyse future health spending patterns, relative to need.

**Findings:**

In 2019, at the onset of the COVID-19 pandemic, US$9·2 trillion (95% uncertainty interval [UI] 9·1–9·3) was spent on health worldwide. We found great disparities in the amount of resources devoted to health, with high-income countries spending $7·3 trillion (95% UI 7·2–7·4) in 2019; 293·7 times the $24·8 billion (95% UI 24·3–25·3) spent by low-income countries in 2019. That same year, $43·1 billion in development assistance was provided to maintain or improve health. The pandemic led to an unprecedented increase in development assistance targeted towards health; in 2020 and 2021, $1·8 billion in DAH contributions was provided towards pandemic preparedness in LMICs, and $37·8 billion was provided for the health-related COVID-19 response. Although the support for pandemic preparedness is 12·2% of the recommended target by the High-Level Independent Panel (HLIP), the support provided for the health-related COVID-19 response is 252·2% of the recommended target. Additionally, projected spending estimates suggest that between 2022 and 2026, governments in 17 (95% UI 11–21) of the 137 LMICs will observe an increase in national government health spending equivalent to an addition of 1% of GDP, as recommended by the HLIP.

**Interpretation:**

There was an unprecedented scale-up in DAH in 2020 and 2021. We have a unique opportunity at this time to sustain funding for crucial global health functions, including pandemic preparedness. However, historical patterns of underfunding of pandemic preparedness suggest that deliberate effort must be made to ensure funding is maintained.

**Funding:**

Bill & Melinda Gates Foundation.

## Introduction

March 11, 2022, marked the 2 years since WHO declared COVID-19 a Public Health Emergency of International Concern.^1^ More than 18 million excess deaths have been attributed globally to the virus between Jan 1, 2020, and Dec 31, 2021,^2^, ^3^ making it one of the leading causes of death over this period. Beyond this enormous loss of life, the COVID-19 pandemic brought with it catastrophic social and economic losses. The latest reports estimate a resulting 3·3% decrease in global gross domestic product (GDP) in 2020.^4^, ^5^ Globally, countries—high-income and low-income alike—have responded with economic and social packages to mitigate the consequences of public health measures instituted to manage the pandemic. Furthermore, as a direct consequence of interventions such as social mobilisation efforts and improvements to cold-chain storage facilities, additional spending in the health sector has been necessary worldwide.^6^

Understanding how much has been spent in the health sector as a result of the COVID-19 pandemic is an important initial step for understanding how existing funding resources have been used, as well as what kind of spending will be needed to effectively prepare for the next pandemic. It is valuable to examine global health spending at the onset of the pandemic in order to understand the financial context of the pandemic and the relative magnitude of change in spending that would be necessary to adequately prevent the next global pandemic.

**Research in context**

**Evidence before this study**

WHO and the Global Burden of Disease Health Financing Collaborator network have both produced estimates of total health spending, government health spending, prepaid private spending, and out-of-pocket spending from 1995 to 2018. Past estimates highlight that, although health spending continues to increase over time worldwide, inequities in health spending also continue to persist. Research published in 2021 estimated that US$13·7 billion of development assistance was provided in 2020 for the health-related COVID-19 response. We searched PubMed for all results published in English between Feb 2, 2022, and March 11, 2022, using the keywords associated with development assistance for pandemic preparedness and response and global public goods. We searched for “donor support for pandemic preparedness and response”, “development assistance for pandemic preparedness and response”, “external funding for pandemic preparedness and response”, “donor support for global public goods”, and “external funding for global public goods”. Our search yielded 10 articles on financing pandemic preparedness and response. All the articles, including three commission reports, emphasised an underinvestment in pandemic preparedness and response globally. These underinvestment claims relied on needs estimates from the World Bank on the annual cost of building a pandemic preparedness system across all low-income and middle-income countries ($3·4 billion compared with the less than $1 billion raised for cross-border externalities in 2013) and historical shortfalls in funding ($225 million annually for epidemic and pandemic prevention and control activities) faced by WHO's Health Emergencies and Health System Preparedness Programmes. Some studies also highlighted the compelling investment case for supporting global pandemic preparedness and response efforts by providing estimates of the funding needed compared with the economic, social, and health loss that could be associated with pandemics. Two articles provided estimates of international funding for global functions. The first article found that $4·7 billion was dedicated to global functions in 2013. Global health functions are defined to include the provision of global public goods, management of cross-border externalities (outbreak preparedness and response included,) and leadership and stewardship. This figure is a high-bound estimate since preparedness and response is only one of several items included under global health functions. The second article estimates spending on global functions in 2017 to be $7·0 billion. Spending specifically for epidemic and pandemic preparedness and response was $0·18 billion in 2013, $1·01 billion in 2015, and $0·48 billion in 2017. Furthermore, an analysis of pandemic preparedness and response financing needs and gaps prepared for the G20 Joint Finance & Health Task Force by teams from the World Bank and WHO included estimates of available funding for pandemic preparedness and response. The analysis shows that $3·1 billion was available for national pandemic preparedness and response activities and $1·2 billion was available for global pandemic preparedness and response activities. It is unclear what years are covered by these estimates. An additional search using “funding for global health security” found other existing tracking efforts such as the global health security tracking portal. This is a portal run by a team at Georgetown University in partnership with Talus Analytics that tracks commitments and disbursements for global health security. Funding flows for global health security are tracked from 2014 to 2022 and reported at the country level. Detailed data on who is funding, what is being funded, and how much in funding has been committed and disbursed is provided for each country.

**Added value of this study**

This study provides crucial information about past investments in health, pandemic preparedness, and health-care spending attributed to COVID-19, and highlights the potential for increased investment in pandemic preparedness. Specifically, this study adds to previous literature a review of donor support for pandemic investments that covers a longer time series (1990–2021) than other existing studies and compares these estimates with total development assistance for health disbursed in the same period. Our study is also the most recent study to bring together projections of necessary funding with the available and expected funding data to examine potential deficits in fund availability for pandemic preparedness. Additionally, our study estimates how much donor support was targeted towards the COVID-19 health sector response for 2020 and 2021, and projects future availability of development assistance for health and government health spending and the potential for pandemic funding. To put these estimates into context, this research also estimates domestic and total health spending for 204 countries from 1995 to 2019.

**Implications of all the available evidence**

The marked increase in donor funding for COVID-19 suggests a potential for increased pandemic preparedness funding going forward. However, chronic underfunding of pandemic preparedness might persist into the future without proactive measures to change course post pandemic. Now more than ever, the COVID-19 pandemic has provided the impetus and raised awareness for the importance of investing in robust health systems, and so this moment presents the unique opportunity to end the cycle of panic and neglect that has long characterised pandemic preparedness financing efforts.

To this end, pandemic preparedness and response has become an issue of high interest and some debate. According to WHO, pandemic preparedness means having national response plans, resources, and the capacity to support operations in the event of a pandemic,^7^, ^8^ although some research has shown that traditional measures of pandemic preparedness were not associated with COVID-19 outcomes.^7^ To respond to this finding and discover what is necessary to be truly prepared for a pandemic, there have been several high-level convenings aimed at understanding how a local epidemic became a global pandemic and how globally we can prevent, or more effectively manage, the effects of such large-scale catastrophes going forward. These convenings have included the G20 High Level Independent Panel on Pandemic Preparedness (HLIP) and the WHO-convened Independent Panel for Pandemic Preparedness and Response.^9^, ^10^ Both bodies have estimated costs of what will be needed in additional funding to implement more robust global systems of pandemic preparedness to manage the next pandemic. The HLIP proposed that national governments put an additional 1% of GDP towards national spending on health, inclusive of pandemic preparedness, and an additional US$15 billion in development assistance support annually for the next 5 years. WHO's Independent Panel proposed an additional $10 billion in health spending for pandemic preparedness. Although the reports from these bodies have provided insight into resources that might be needed, there is very little understanding of what is currently being spent or the available resources for pandemic preparedness and response from a global perspective.

This study has three objectives: first, to provide an assessment of global health spending at the onset of the COVID-19 pandemic; second, to characterise the amount of development assistance for health (DAH) resources for pandemic preparedness and the health-related COVID-19 response before and during the pandemic; and third, to examine expectations for future health spending and expected need for investment in pandemic preparedness. Health spending projections were developed by drawing from historical trends and relationships with key covariates and represent a baseline of expected spending if current trends and relationships with key covariates persist. We focus on the external support of pandemic preparedness and response to highlight its importance as a crucial global public good that is essential for global health security and equity. For this work, resources for pandemic preparedness are defined as investments in systems to prepare for and prevent a pandemic, and resources for the COVID-19 response are defined as the funds provided since the beginning of the pandemic with a focus on prevention and treatment. Pandemic preparedness investments are tracked at three levels: global, regional, and national. This study makes a distinctive contribution to the literature in that it examines funding for pandemic preparedness in the context of global health spending at the onset of the pandemic and prospectively, given the proposals from the High Level Independent Panel. The findings from this study can inform global health stakeholders about the magnitude of change in spending on pandemic preparedness and response needed to pre-empt or better manage the next pandemic. This manuscript was produced as part of the GBD Collaborator Network and in accordance with the Global Burden of Diseases, Injuries, and Risk Factors Study (GBD) Protocol.

## Methods

### Overview

In this analysis of global health spending between 1990 and 2026, we examined four mutually exclusive sources of health spending: development assistance, government spending, out-of-pocket spending, and prepaid private spending. By aggregating these four financing sources, we estimated total health spending for 204 countries and territories. Each health spending measure covers specific time periods largely determined by data availability (appendix p 40). We tracked DAH, which captures the financial and non-financial resources that international development agencies transferred to low-income and middle-income countries, with the primary intention of promoting or improving health, from 1990 to 2021. For development assistance, we relied on data from the Organisation for Economic Co-operation and Development (OECD)'s Creditor Reporting System (CRS)^11^ among others described in the appendix (p 62). We used the April 2022 OECD CRS update version, which was the latest publicly available downloadable dataset at the time of analyses for this study. Government health spending captures national spending on health-related activities, including social health insurance. Prepaid private health spending includes private insurance and health services provided by non-governmental organisations. Out-of-pocket spending captures payments for health commodities and services by individuals at the point of use. For estimates of domestic health spending (government health spending, out-of-pocket spending, and prepaid private spending), we modelled estimates from the WHO Global Health Expenditure Database (GHED).^12^ Estimates for domestic health spending were made from 1995 to 2019. Future health spending estimates were generated using the available retrospective data and extended to 2026.

### Estimating development assistance for health, COVID-19, and pandemic preparedness, 1990–2021

To generate the estimates of donor contributions, we relied on disbursement data on project-level activities reported in the databases of international development agencies such as the OECD's CRS, the Bill & Melinda Gates Foundation, and the Global Fund to Fight AIDS, Tuberculosis and Malaria. Additionally, we leveraged income or revenue statements from each agency to ensure that resources transferred between agencies were accounted for only once. Preliminary estimates of disbursement were generated for the most recent year using budget data. We searched these international databases using keywords to isolate relevant projects, including the following keywords: “coordination”, “communication”, “surveillance”, “laboratory”, “infection prevention control”, “case management”, “operational support”, “border”, “essential health services”, and “diagnostics manufacture”. We also incorporated additional keywords related to vaccines and immunisation to appropriately capture the evolution of the global COVID-19 response efforts (appendix p 128). Keywords were translated into nine languages (English, Spanish, French, Portuguese, Italian, German, Dutch, Norwegian, and Swedish) to identify the associated health focus areas and programme areas for each project. In aggregate, we identified ten health focus areas and 49 programme areas.

The COVID-19 estimates were generated from results from development agencies that have databases that include reporting in 2020 or 2021, or both years. For development agencies that did not have up-to-date reporting, we relied on the UN Office of the Coordinator for Humanitarian Affairs Financial Tracking Service database^13^ and the International Aid Transparency Initiative COVID Database.^14^ Furthermore, we reviewed all our current data sources for relevance and incorporated new data sources, such as the US Government foreign assistance website and World Bank Support for Country Access to COVID-19 Vaccines, as appropriate and relevant. We obtained up-to-date data for 38 agencies for generating COVID-19 development assistance estimates, mainly bilateral and UN agencies. Programme areas for COVID-19 development assistance were decided on the basis of WHO's 2021 COVID-19 Strategic Preparedness and Response Plan (SPRP 2021),^15^ and we also added research and development initiatives for vaccines and other therapeutics from development agencies. Our keyword approach only captured projects that were explicitly targeted to the COVID-19 health-related response efforts. Other support that might have been relevant but did not explicitly include “COVID-19” in the project description was not captured in this study. Detailed explanations of the methods have been published previously and are reproduced in the appendix (p 61).^16^, ^17^, ^18^, ^19^, ^20^, ^21^, ^22^

We defined development assistance for pandemic preparedness and response as any development assistance contribution targeted towards preparedness planning and response activities for outbreaks, epidemics, and pandemics. Such support could include assistance in the development of national response plans, training for laboratory staff, provision of personal protective equipment, and resources for hiring surge staff. This study improved upon previous research by including new keywords for pandemic preparedness and response. We also refined our methodology for estimating assistance disbursed through WHO by adjusting our disbursement to include only 76% of its assessed contributions, on the basis of a new report from the OECD Development Assistance Committee:^23^ This rescale adjustment of development assistance provided through WHO was necessary because, although the organisation provides support to all member countries through its headquarters, not all member countries that receive support are LMICs, and therefore this support falls outside of what we define as eligible for development assistance. The adjustment accounts for this distinction.^23^ The keywords used to isolate relevant pandemic preparedness projects include “pandemic preparedness”, “epidemic alert”, “outbreak response”, “pandemic influenza”, “epidemiological investigation”, “contact management”, “preparedness and response plan”, “biosafety measure”, “health security preparedness”, “rapid response strategy”, “pandemic planning”, and “international health regulation”. These keywords were informed by literature.^24^, ^25^ To further disaggregate funding for pandemic preparedness into global, regional, or country levels, we used the geographical information provided in the various databases on the recipient of the funds.

### Estimating total domestic health spending, 1995–2019

For domestic health spending sources, including government, out-of-pocket, and prepaid private health spending, we obtained data from the GHED in current national currency units for all available countries and territories. For the few countries not reported on in the GHED, data were extracted from the country's ministry of health or finance website. We adjusted these estimates for inflation, modelled estimates to ensure consistency over time and completeness across locations, and estimated uncertainty (appendix pp 46–59).

For each of the three domestic financing sources, we assessed the reliability of data extracted from the GHED by evaluating metadata provided by WHO. Using natural language processing techniques, we assigned a data quality score of 0 to 5 (in which 0 represents the least reliable data and 5 represents the most reliable data) to each downloaded datapoint according to metadata reporting data source, data type, methods of estimation, and completeness (methods are detailed in appendix pp 46–50). Using these scores to weight the raw data, we then applied a spatiotemporal Gaussian process model to generate a complete time series of estimates from 1995 to 2019 for each country, and calculated 95% uncertainty intervals (UIs).

### Estimating future health spending, 2020–26

We estimated GDP; total government spending across all sectors; and health spending for each of four financing sources from 2020 to 2026 (the end of the 5-year term for which HLIP's recommended need estimates exist), drawing heavily from the methods used in our previous research to predict future health spending. Projections were generated using ensemble modelling techniques taking the mean of 500 estimated projections from a broad set of linear mixed-effects models (appendix p 165). Model selection was defined by country-specific and year-specific out-of-sample validation. Projections were generated subsequently, allowing us to use previously projected values as covariates. We forecasted GDP per working-age population, defined as ages 20–64 years, from 2025 to 2026, with retrospective estimates between 1970 and 2019. GDP estimates from 2022 to 2026 drew on short-term forecasts from several data sources that estimated the economic effects of COVID-19 (appendix p 8). General government spending per gross domestic product was forecasted from 2022 to 2026, with retrospective estimates between 1980 and 2019. We estimated DAH from 2022 to 2026 as a proportion of the donor country's general government spending, and for private donors using AutoRegressive Integrated Moving Average modelling techniques.^26^, ^27^

Total DAH was aggregated across donors, and we used a subsequent ensemble model to project the proportion of total DAH expected to be received by each recipient from 2021 to 2026 with smoothed retrospective data extending to 2019. Models were fit on smoothed DAH estimates in order to capture broad trends, rather than idiosyncratic DAH shocks. Because high-income countries are, by definition, not eligible to receive DAH, we also modelled when countries are expected to transition to high income according to the World Bank high-income cutoff and our GDP forecasts. Government health spending as a share of general government spending was projected from 2020 to 2026, with retrospective data extending to 2019. To capture increases in government spending in response to COVID-19, we evaluated the 2020 and 2021 estimates generated through our projections against the International Monetary Fund's (IMF's) database of Country Fiscal Measures in Response to the COVID-19 Pandemic, which was published in October of 2020 and October of 2021. If the year-over-year spending increase in 2020 or 2021 shown in our estimates was less than the increase reported by the IMF for 2020 or 2021, we adjusted our estimates upwards by the difference to better reflect the most recent available observed data. We also projected both prepaid private health spending and out-of-pocket health spending as a share of GDP from 2020 to 2026.

Model uncertainty was propagated using ensemble modelling techniques, and parameter uncertainty was propagated by taking draws of the variance–covariance matrix for each estimated model. A random-walk residual was added to each projection to propagate fundamental uncertainty, and we generated 95% UIs by taking the 2·5th and 97·5th percentile of the 500 random draws. Importantly, our projections are based on past trends and relationships of observed retrospective data and do not capture the effects of possible future shocks such as natural disasters, conflicts, or policy changes that were not observed in the past. Moreover, dramatic changes to policy that fall outside of the patterns observed in the past and captured in our covariates are also not included in these projections, such that they should be interpreted as a baseline projection of the spending that can be expected by extrapolating trends and relationships with key covariates.

### Reporting

All results are reported in inflation-adjusted 2021 US$ unless otherwise specified. To report the DAH estimate in 2021 inflation-adjusted dollars, we took disbursements in nominal US$ in the year of disbursements and used US GDP deflators from the IMF World Economic Outlook database (WEO) to convert the series to constant 2021 US$. To adjust for inflation for the historical and future global health spending estimates, we used country-specific exchange rate data and deflator series from IMF WEO to convert the series to constant 2021 US$. We also converted these estimates into 2021 purchasing-power-parity-adjusted US$ to assess spending relative to local prices (even though a portion of DAH might have been spent elsewhere).

We reported all spending estimates by GBD 2019 super-region and 2020 World Bank income group categories as assigned in 2020. For the global aggregate, as well as for the region-specific and income-specific aggregates, we reported estimates for each group as a whole instead of using the mean of the countries included in that group. For all tables and figures, the country income classifications were held constant at the 2021 reported level, irrespective of whether they changed groups.

To calculate the extent to which the currently available funding falls short of what is proposed, we extracted estimates of need from the HLIP report.^28^ The panel report provides an estimate of the additional funding needed to provide effective pandemic prevention and preparedness from both a domestic and donor perspective. The estimate focuses on the additional funding needed in three core global public goods areas: robust surveillance and detection networks, building resilience in health systems, and supply chains for medical countermeasures; we report on the total need. We completed all the analyses using Stata (version 15.1), R (versions 3.6 and 4.0), and Python (versions 3.6, 3.7, and 3.10). For a complete list of packages used in the analysis, refer to the appendix (p 41).

### Role of the funding source

The funder of this study had no role in study design, data

collection, data analysis, data interpretation, or writing of

the report.

## Results

In 2019, at the onset of the COVID-19 pandemic, global health spending worldwide was $9·2 trillion (95% UI 9·1–9·3). Global spending on health per person was $1183 (95% UI 1171–1195) and is projected to reach $1366 (1345–1387) in 2026 (table 1). Per-person health spending in 2019 was 2·0% (95% UI 0·8–3·3) higher than in 2018 and 17·8% (16·4–19·2) higher than in 2010 (appendix pp 6–7). However, this total hides the substantial variation in spending across countries. In 2019, per-person health spending in the USA (the country with highest health spending per person) was $11 583 (95% UI 11 373–11 828); almost 10 times the global spending per person, and 1761 times the spending in the country with lowest health spending per person (Somalia; $7 per person (95% UI 6–7). High-income countries spent $7·3 trillion (95% UI 7·2–7·4) in 2019; 293·7 times the $24·8 billion (95% UI 24·3–25·3) spent by low-income countries in 2019. In 2026, these gaps are projected to persist, with spending in the USA being $13 821 per person (95% UI 13 398–14 300); 1717 times the $8 per person (95% UI 7–10) health spending in Somalia and approximately 10 times the $1363 (95% UI 1363 (1342–1385) global spending per person. In 2019, government spending, measured as a fraction of the total health spending, was highest in Greenland (100·0% [95% UI 100·0–100·0]) and Brunei (94·2% [93·7–95·1]) and lowest in Cameroon (5·2% [4·4–6·2]) and Afghanistan (5·5% [4·6–6·5]; appendix pp 19–38). Out-of-pocket spending, measured as a fraction of the total health spending, was highest in Armenia (84·4% [95% UI 83·1–85·7]) and Afghanistan (81·3% [79·8–82·6]) and lowest in Greenland (0·0% [0·0–0·0]) and Botswana (3·0% [2·7–3·4]; appendix pp 19–38). The country that received the most development assistance in 2019 was the Democratic Republic of the Congo ($1005·4 million; appendix pp 19–38), and the country that received the most per person was the Marshall Islands ($203). Donor spending, measured as a fraction of the total health spending, was as high as 69·6% (95% UI 67·0–72·3) in South Sudan and 66·2% (61·8–70·7) in Somalia (appendix pp 19–38).

**Table 1:** Health spending in 2019 and 2026

		**Health spending per person (2021 US$)**		**Health spending per person (2021 purchasing-power parity-adjusted US$)**		**Total health spending per gross domestic product**		**Government health spending per person (2021 US$); 2019**	**Prepaid private spending per person (2021 US$); 2019**	**Out-of-pocket spending per person (2021 US$); 2019**	**Development assistance for health per person (2021 US$); 2019**
		2019	2026	2019	2026	2019	2026				
**Global**											
Total		1183 (1171–1195)	1366 (1345–1387)	1518 (1505–1531)	1769 (1744–1796)	9·7% (9·6–9·8)	10·2% (10·0–10·4)	708 (700–715)	254 (247–262)	215 (210–221)	6 (6–6)
**World Bank income groups**											
High income		5938 (5876–6004)	6978 (6863–7101)	6469 (6405–6535)	7600 (7483–7725)	12·4% (12·2–12·5)	13·4% (13·1–13·6)	3682 (3645–3717)	1441 (1395–1485)	815 (785–851)	..
Upper-middle income		575 (561–590)	773 (743–807)	1085 (1062–1108)	1426 (1378–1480)	5·7% (5·6–5·9)	6·2% (5·9–6·5)	322 (309–336)	65 (59–70)	187 (180–194)	1 (1–1)
Lower-middle income		117 (114–121)	140 (136–145)	300 (291–309)	362 (349–375)	4·1% (3·9–4·2)	4·1% (4·0–4·3)	47 (45–48)	12 (11–14)	55 (53–58)	3 (3–3)
Low-income		37 (36–38)	41 (40–44)	145 (140–150)	153 (146–160)	4·9% (4·7–5·2)	5·0% (4·7–5·3)	9 (8–9)	2 (2–2)	16 (16–17)	11 (11–11)
**Country**											
Central Europe, Eastern Europe, and Central Asia		649 (642–657)	713 (700–727)	1514 (1496–1534)	1644 (1612–1680)	6·1% (6·0–6·2)	6·3% (6·2–6·5)	420 (413–426)	24 (23–26)	204 (200–207)	1 (1–1)
	Albania (UM)	339 (317–363)	423 (392–458)	851 (796–911)	1063 (984–1149)	5·6% (5·2–6·0)	5·6% (5·2–6·1)	184 (163–205)	0 (0–0)	152 (142–162)	4 (4–4)
	Armenia (UM)	514 (483–548)	596 (560–635)	1602 (1508–1708)	1858 (1747–1980)	10·5% (9·5–11·3)	10·5% (9·7–11·2)	64 (61–68)	9 (6–12)	434 (405–466)	7 (7–7)
	Azerbaijan (UM)	206 (188–224)	217 (197–237)	605 (553–658)	637 (581–696)	3·8% (3·5–4·2)	3·8% (3·4–4·1)	61 (56–67)	0 (0–0)	144 (127–160)	1 (1–1)
	Belarus (UM)	413 (386–443)	443 (411–478)	1227 (1146–1317)	1316 (1221–1420)	5·8% (5·4–6·2)	6·1% (5·6–6·6)	288 (267–311)	15 (11–20)	107 (95–122)	2 (2–2)
	Bosnia and Herzegovina (UM)	579 (554–606)	757 (721–795)	1439 (1378–1508)	1881 (1792–1978)	8·9% (8·4–9·5)	9·7% (9·0–10·5)	404 (384–424)	2 (1–3)	171 (155–186)	1 (1–1)
	Bulgaria (UM)	840 (806–871)	1009 (943–1070)	1862 (1788–1932)	2238 (2091–2373)	7·3% (7·0–7·6)	7·2% (6·7–7·7)	492 (468–517)	16 (10–23)	332 (312–351)	0 (0–0)
	Croatia (H)	1072 (1035–1111)	1350 (1247–1462)	2100 (2027–2176)	2644 (2443–2864)	6·6% (6·3–6·8)	6·9% (6·3–7·5)	882 (845–919)	72 (63–82)	118 (107–130)	NA
	Czechia (H)	2117 (2061–2172)	2414 (2331–2498)	3513 (3421–3605)	4008 (3869–4146)	7·8% (7·6–8·0)	8·0% (7·7–8·3)	1732 (1681–1784)	85 (77–95)	300 (287–314)	NA
	Estonia (H)	1793 (1767–1820)	2210 (2108–2323)	2764 (2724–2806)	3407 (3250–3581)	6·9% (6·8–7·0)	7·1% (6·8–7·5)	1330 (1311–1348)	28 (25–32)	435 (417–452)	NA
	Georgia (UM)	352 (334–374)	459 (426–501)	1186 (1124–1259)	1548 (1434–1688)	7·4% (6·8–8·1)	7·4% (6·7–8·4)	141 (127–157)	37 (28–50)	158 (151–164)	16 (16–16)
	Hungary (H)	1194 (1167–1222)	1464 (1407–1523)	2337 (2285–2392)	2866 (2755–2981)	6·4% (6·3–6·6)	6·5% (6·2–6·8)	818 (794–842)	47 (43–51)	329 (316–342)	NA
	Kazakhstan (UM)	296 (276–317)	329 (303–357)	842 (787–903)	936 (861–1017)	2·9% (2·7–3·1)	2·9% (2·7–3·2)	177 (160–194)	17 (11–24)	101 (91–112)	1 (1–1)
	Kyrgyzstan (LM)	73 (64–82)	73 (64–83)	302 (265–338)	301 (265–341)	5·2% (4·6–5·8)	5·4% (4·7–6·1)	33 (27–39)	0 (0–0)	34 (28–42)	6 (6–6)
	Latvia (H)	1300 (1260–1339)	1603 (1523–1682)	2193 (2126–2259)	2704 (2568–2837)	6·5% (6·3–6·7)	6·7% (6·2–7·2)	785 (755–813)	29 (22–38)	486 (459–510)	NA
	Lithuania (H)	1541 (1507–1572)	1924 (1852–1994)	2818 (2756–2876)	3518 (3387–3647)	6·9% (6·7–7·0)	7·1% (6·6–7·6)	1010 (980–1036)	35 (32–38)	495 (479–512)	NA
	Moldova (UM)	234 (217–254)	316 (290–344)	683 (631–742)	922 (845–1004)	5·6% (4·2–8·3)	5·7% (4·3–8·2)	137 (122–154)	3 (2–4)	91 (81–100)	4 (4–4)
	Mongolia (LM)	195 (186–204)	239 (225–255)	553 (528–578)	677 (637–724)	4·1% (3·9–4·3)	4·5% (4·1–4·8)	108 (101–115)	6 (4–9)	62 (58–67)	19 (19–19)
	Montenegro (UM)	811 (767–856)	934 (878–997)	1957 (1852–2067)	2255 (2120–2407)	8·3% (7·8–8·8)	8·6% (8·1–9·2)	483 (447–520)	7 (4–10)	318 (296–344)	2 (2–2)
	North Macedonia (UM)	555 (531–579)	639 (609–667)	1487 (1423–1550)	1712 (1631–1788)	8·1% (7·8–8·5)	8·2% (7·7–8·6)	322 (301–346)	3 (2–5)	229 (223–234)	1 (1–1)
	Poland (H)	1096 (1075–1116)	1400 (1351–1452)	2324 (2279–2367)	2969 (2864–3079)	6·4% (6·2–6·5)	6·7% (6·4–7·0)	783 (763–799)	89 (84–93)	225 (216–233)	NA
	Romania (H)	830 (807–852)	1040 (978–1101)	1908 (1856–1958)	2391 (2248–2531)	5·8% (5·6–5·9)	5·9% (5·5–6·4)	666 (645–687)	8 (7–10)	156 (152–162)	NA
	Russia (UM)	668 (650–688)	673 (639–712)	1690 (1644–1740)	1702 (1616–1800)	5·6% (5·4–5·8)	5·9% (5·6–6·3)	406 (389–422)	16 (11–21)	247 (238–256)	0 (0–0)
	Serbia (UM)	560 (538–583)	742 (706–777)	1321 (1267–1374)	1750 (1664–1831)	6·6% (6·3–6·9)	6·8% (6·4–7·1)	331 (311–352)	12 (9–18)	212 (202–221)	5 (5–5)
	Slovakia (H)	1482 (1432–1532)	1780 (1704–1860)	2494 (2410–2579)	2995 (2868–3130)	6·9% (6·7–7·2)	7·3% (6·9–7·7)	1175 (1131–1220)	31 (24–41)	276 (249–304)	NA
	Slovenia (H)	2434 (2373–2497)	2955 (2857–3050)	3672 (3580–3768)	4459 (4311–4602)	8·5% (8·3–8·7)	8·9% (8·6–9·3)	1761 (1704–1823)	388 (372–405)	285 (275–295)	NA
	Tajikistan (LM)	56 (51–60)	64 (59–70)	274 (252–298)	318 (289–347)	6·8% (6·1–7·5)	6·8% (6·0–7·5)	15 (14–16)	0 (0–0)	36 (31–41)	5 (5–5)
	Turkmenistan (UM)	714 (669–762)	744 (697–793)	1227 (1150–1310)	1279 (1198–1364)	8·3% (5·7–13·6)	8·2% (5·8–13·0)	133 (119–147)	37 (28–47)	543 (504–587)	1 (1–1)
	Ukraine (LM)	326 (317–336)	221 (211–231)	969 (940–997)	657 (626–686)	7·1% (6·7–7·5)	7·2% (6·2–8·3)	149 (141–157)	9 (6–13)	166 (161–170)	3 (3–3)
	Uzbekistan (LM)	108 (104–112)	134 (126–142)	461 (445–480)	575 (540–608)	5·7% (5·5–6·1)	5·9% (5·5–6·4)	43 (41–45)	1 (0–1)	61 (58–65)	3 (3–3)
GBD high-income region		6531 (6463–6606)	7649 (7515–7789)	6971 (6902–7047)	8145 (8008–8285)	12·9% (12·7–13·0)	14·0% (13·7–14·2)	4025 (3985–4066)	1614 (1563–1664)	892 (859–932)	NA
	Andorra (H)	2805 (2669–2943)	2906 (2741–3081)	3913 (3723–4106)	4053 (3823–4298)	6·3% (6·0–6·6)	6·6% (6·2–7·1)	1931 (1796–2067)	502 (466–540)	372 (352–393)	NA
	Argentina (UM)	1038 (999–1079)	1145 (1075–1219)	2299 (2211–2388)	2535 (2380–2699)	9·4% (9·1–9·8)	9·8% (9·2–10·5)	644 (613–676)	106 (88–127)	286 (271–302)	2 (2–2)
	Australia (H)	6421 (6293–6527)	7476 (7256–7701)	5701 (5588–5796)	6638 (6443–6838)	10·5% (10·0–11·3)	11·3% (10·5–12·2)	4566 (4480–4646)	810 (753–870)	1044 (1008–1081)	NA
	Austria (H)	5741 (5672–5817)	6369 (6144–6615)	6425 (6348–6510)	7128 (6876–7402)	10·4% (10·3–10·6)	11·0% (10·6–11·5)	4188 (4126–4250)	451 (430–474)	1102 (1074–1132)	NA
	Belgium (H)	5581 (5481–5695)	6205 (6015–6421)	6140 (6030–6265)	6827 (6617–7064)	10·7% (10·5–10·9)	11·3% (10·9–11·7)	4288 (4191–4394)	280 (268–293)	1012 (983–1039)	NA
	Brunei (H)	1010 (933–1084)	1136 (980–1311)	1506 (1392–1617)	1694 (1461–1955)	2·2% (2·1–2·4)	2·2% (1·9–2·6)	956 (880–1032)	0 (0–0)	54 (45–65)	NA
	Canada (H)	5968 (5893–6040)	6445 (6263–6623)	6073 (5998–6147)	6560 (6374–6740)	11·2% (11·0–11·3)	11·6% (11·3–12·0)	4188 (4129–4247)	890 (853–929)	890 (868–916)	NA
	Chile (H)	1545 (1516–1573)	1850 (1800–1900)	2568 (2521–2614)	3076 (2992–3158)	9·7% (9·5–9·9)	10·6% (9·9–11·4)	788 (769–808)	250 (239–263)	506 (492–520)	NA
	Cyprus (H)	1426 (1364–1492)	1658 (1575–1740)	2080 (1991–2177)	2419 (2298–2539)	5·1% (4·2–6·7)	5·5% (4·6–7·1)	719 (676–767)	188 (173–204)	519 (480–563)	NA
	Denmark (H)	6708 (6581–6831)	7702 (7406–8029)	6330 (6210–6447)	7269 (6989–7577)	10·0% (9·8–10·2)	10·5% (10·1–11·0)	5592 (5470–5718)	169 (162–175)	946 (925–966)	NA
	Finland (H)	4906 (4828–4985)	5555 (5393–5730)	4883 (4805–4961)	5529 (5367–5703)	9·2% (9·0–9·3)	9·6% (9·2–9·9)	3918 (3848–3988)	127 (119–136)	862 (837–887)	NA
	France (H)	4976 (4903–5047)	5528 (5392–5657)	5700 (5616–5781)	6332 (6177–6480)	11·1% (10·7–11·4)	11·7% (11·3–12·1)	3761 (3709–3810)	752 (703–805)	463 (443–485)	NA
	Germany (H)	5887 (5829–5941)	6628 (6471–6788)	6767 (6701–6830)	7619 (7439–7804)	11·4% (11·3–11·5)	12·0% (11·7–12·3)	4575 (4522–4630)	558 (548–568)	754 (737–770)	NA
	Greece (H)	1670 (1591–1761)	1913 (1821–2023)	2657 (2532–2802)	3044 (2898–3220)	8·1% (7·7–8·5)	8·2% (7·7–8·8)	828 (765–894)	247 (223–272)	595 (557–635)	NA
	Greenland (H)	6700 (5922–7459)	8853 (7862–9868)	6013 (5315–6694)	7945 (7056–8856)	11·1% (9·8–12·5)	12·2% (10·6–13·9)	6699 (5922–7458)	0 (0–1)	0 (0–1)	NA
	Iceland (H)	6528 (6297–6797)	7301 (6734–7921)	5654 (5454–5887)	6324 (5833–6860)	8·8% (8·2–9·3)	9·5% (8·4–10·6)	5403 (5179–5673)	109 (104–113)	1016 (971–1059)	NA
	Ireland (H)	5733 (5511–5968)	8063 (7662–8451)	6510 (6259–6777)	9157 (8702–9598)	6·8% (6·5–7·1)	7·2% (6·8–7·7)	4266 (4063–4486)	791 (730–862)	675 (630–724)	NA
	Israel (H)	3565 (3500–3629)	4166 (4043–4293)	3172 (3114–3230)	3707 (3598–3820)	7·0% (6·5–7·3)	7·2% (6·8–7·6)	2356 (2308–2406)	441 (421–461)	768 (734–801)	NA
	Italy (H)	3114 (3052–3177)	3442 (3322–3561)	4054 (3973–4136)	4481 (4325–4636)	8·6% (8·5–8·9)	9·0% (8·6–9·3)	2301 (2241–2360)	86 (82–90)	727 (707–748)	NA
	Japan (H)	4317 (4214–4425)	4772 (4621–4936)	4909 (4792–5033)	5427 (5255–5613)	10·7% (10·4–11·0)	11·2% (10·7–11·6)	3620 (3515–3729)	140 (136–145)	557 (544–569)	NA
	Luxembourg (H)	7114 (6738–7505)	7865 (7299–8429)	6862 (6499–7239)	7586 (7040–8130)	5·3% (5·0–5·6)	5·5% (5·1–5·9)	6164 (5809–6543)	230 (199–269)	720 (634–814)	NA
	Malta (H)	3169 (3075–3257)	3520 (3339–3694)	4715 (4575–4845)	5237 (4968–5495)	8·9% (7·9–9·6)	8·9% (8·0–10·0)	2010 (1941–2087)	71 (66–77)	1088 (1038–1139)	NA
	Monaco (H)	3696 (3490–3910)	3975 (3715–4258)	3821 (3607–4042)	4109 (3840–4402)	1·7% (1·5–2·0)	1·8% (1·6–2·1)	3156 (2951–3362)	261 (210–317)	279 (265–294)	NA
	Netherlands (H)	5956 (5831–6095)	6786 (6496–7092)	6419 (6284–6568)	7314 (7001–7643)	10·2% (9·9–10·5)	10·8% (10·3–11·3)	3917 (3818–4026)	1408 (1334–1481)	632 (594–666)	NA
	New Zealand (H)	4615 (4526–4711)	5269 (5110–5432)	4441 (4355–4532)	5069 (4916–5226)	9·4% (8·8–9·8)	10·0% (9·3–10·7)	3481 (3399–3567)	561 (532–589)	572 (544–602)	NA
	Norway (H)	9180 (8983–9407)	10650 (10210–11145)	7298 (7141–7478)	8467 (8117–8860)	10·5% (10·3–10·8)	11·2% (10·6–11·8)	7880 (7678–8106)	30 (26–34)	1270 (1225–1314)	NA
	Portugal (H)	2317 (2249–2389)	2578 (2439–2740)	3521 (3418–3630)	3917 (3707–4163)	9·1% (8·9–9·4)	9·4% (8·8–10·0)	1411 (1351–1469)	204 (176–235)	703 (677–730)	NA
	San Marino (H)	3462 (3331–3591)	3766 (3622–3922)	4478 (4309–4645)	4872 (4686–5073)	6·7% (6·5–7·0)	7·1% (6·8–7·4)	2837 (2710–2961)	37 (34–39)	589 (553–625)	NA
	Singapore (H)	2795 (2708–2877)	3796 (3484–4093)	4470 (4331–4601)	6071 (5573–6546)	4·2% (4·0–4·3)	4·7% (4·2–5·4)	1414 (1342–1483)	548 (514–585)	833 (798–875)	NA
	South Korea (H)	2674 (2632–2714)	3671 (3545–3788)	3732 (3673–3789)	5125 (4948–5287)	7·9% (7·8–8·0)	9·3% (8·9–9·7)	1590 (1556–1624)	268 (259–277)	815 (800–831)	NA
	Spain (H)	3019 (2957–3082)	3337 (3238–3439)	4195 (4109–4283)	4637 (4499–4779)	9·3% (9·1–9·5)	9·9% (9·5–10·3)	2129 (2071–2193)	229 (214–243)	660 (644–676)	NA
	Sweden (H)	6553 (6335–6778)	7328 (6984–7681)	6436 (6222–6657)	7198 (6860–7544)	10·9% (10·4–11·3)	11·4% (10·7–12·0)	5563 (5342–5788)	82 (78–85)	909 (878–939)	NA
	Switzerland (H)	10 407 (10 241–10 573)	11 894 (11 537–12 248)	8717 (8578–8856)	9962 (9664–10 259)	11·1% (10·9–11·3)	12·2% (11·8–12·5)	3319 (3251–3393)	4378 (4251–4492)	2710 (2611–2808)	NA
	UK (H)	4892 (4834–4951)	5349 (5207–5484)	5221 (5159–5285)	5709 (5557–5854)	10·0% (9·9–10·2)	10·5% (10·2–10·8)	3888 (3837–3940)	170 (163–179)	833 (799–868)	NA
	USA (H)	11 583 (11 373–11 828)	13 821 (13 398–14 300)	11 583 (11 373–11 828)	13 821 (13 398–14 300)	16·9% (16·6–17·3)	18·6% (17·9–19·3)	5887 (5769–6009)	4386 (4218–4553)	1310 (1201–1449)	NA
	Uruguay (H)	1547 (1529–1566)	1764 (1665–1870)	2238 (2211–2265)	2551 (2408–2704)	9·1% (8·7–9·8)	9·6% (8·8–10·5)	1111 (1096–1126)	178 (170–187)	259 (252–264)	NA
Latin America and Caribbean		563 (547–580)	626 (606–647)	1198 (1164–1234)	1342 (1300–1387)	7·2% (7·0–7·4)	7·7% (7·5–8·0)	289 (277–300)	110 (101–118)	163 (156–170)	2 (2–2)
	Antigua and Barbuda (H)	885 (844–926)	880 (825–931)	1182 (1127–1238)	1176 (1103–1244)	4·9% (4·7–5·1)	5·1% (4·8–5·4)	522 (484–561)	147 (141–153)	216 (194–238)	NA
	Barbados (H)	1161 (1105–1217)	1197 (1135–1259)	1017 (967–1065)	1047 (994–1102)	6·0% (5·7–6·3)	5·9% (5·4–6·3)	520 (474–568)	90 (85–95)	552 (527–578)	NA
	Belize (UM)	284 (267–301)	273 (248–298)	443 (417–469)	426 (386–465)	5·7% (5·3–6·2)	6·0% (5·3–6·7)	195 (178–211)	15 (13–18)	62 (59–65)	12 (12–12)
	Bermuda (H)	8602 (7363–9879)	10 906 (9269–12 638)	5956 (5098–6840)	7551 (6418–8751)	6·7% (5·2–9·2)	7·7% (5·6–10·8)	2427 (2072–2795)	5312 (4155–6563)	862 (672–1090)	NA
	Bolivia (LM)	249 (240–257)	269 (254–283)	668 (646–691)	722 (684–761)	7·0% (6·7–7·2)	7·3% (6·9–7·8)	174 (168–180)	10 (6–14)	57 (52–61)	8 (8–8)
	Brazil (UM)	683 (646–720)	778 (733–823)	1460 (1380–1538)	1662 (1567–1758)	9·0% (8·5–9·5)	9·8% (9·2–10·4)	289 (262–317)	222 (201–243)	172 (158–186)	1 (1–1)
	Colombia (UM)	490 (470–512)	629 (593–668)	1305 (1251–1363)	1675 (1580–1778)	8·0% (7·7–8·4)	8·9% (8·4–9·5)	352 (334–374)	65 (59–70)	73 (66–81)	0 (0–0)
	Costa Rica (UM)	994 (958–1038)	1272 (1217–1339)	1805 (1738–1884)	2308 (2210–2430)	8·1% (7·8–8·4)	9·3% (8·8–9·8)	715 (678–749)	52 (40–66)	214 (196–232)	14 (14–14)
	Cuba (UM)	1357 (1272–1440)	1513 (1409–1616)	2948 (2763–3127)	3287 (3060–3509)	12·0% (10·9–13·2)	13·2% (12·0–14·7)	1211 (1128–1294)	0 (0–0)	145 (138–152)	1 (1–1)
	Dominica (UM)	467 (444–491)	517 (481–558)	780 (741–819)	862 (802–931)	5·5% (5·3–5·8)	5·7% (5·2–6·2)	285 (266–302)	6 (4–8)	149 (134–166)	28 (28–28)
	Dominican Republic (UM)	492 (464–520)	636 (584–689)	1181 (1115–1250)	1528 (1403–1655)	5·8% (5·4–6·2)	6·1% (5·5–6·7)	221 (204–239)	52 (39–67)	215 (196–234)	3 (3–3)
	Ecuador (UM)	492 (461–524)	527 (481–571)	967 (905–1029)	1035 (945–1122)	7·7% (7·2–8·2)	8·2% (7·6–8·9)	305 (283–328)	34 (24–46)	152 (136–172)	1 (1–1)
	El Salvador (LM)	311 (294–329)	356 (334–380)	695 (659–736)	798 (748–850)	7·2% (6·8–7·6)	7·6% (7·1–8·0)	196 (187–205)	23 (16–31)	89 (77–102)	3 (3–3)
	Grenada (UM)	604 (573–634)	621 (587–653)	1024 (971–1075)	1052 (995–1108)	5·5% (5·2–5·8)	5·5% (5·2–5·8)	230 (209–254)	23 (18–28)	312 (288–333)	39 (39–39)
	Guatemala (UM)	323 (313–334)	366 (349–383)	633 (613–654)	717 (684–751)	7·0% (6·5–7·4)	7·2% (6·7–7·7)	122 (116–126)	18 (12–25)	181 (175–188)	3 (3–3)
	Guyana (UM)	285 (276–295)	1063 (982–1155)	724 (701–751)	2703 (2498–2936)	4·7% (4·3–5·3)	4·8% (3·8–5·8)	170 (166–175)	9 (6–13)	95 (88–102)	11 (11–11)
	Haiti (LM)	60 (56–64)	60 (56–65)	102 (96–110)	103 (96–112)	3·1% (2·9–3·3)	3·3% (3·0–3·6)	9 (7–11)	4 (3–6)	35 (31–38)	12 (12–12)
	Honduras (LM)	199 (182–217)	220 (200–241)	443 (406–484)	491 (446–537)	7·0% (6·5–7·7)	7·2% (6·5–7·9)	79 (72–88)	12 (8–16)	106 (93–121)	2 (2–2)
	Jamaica (UM)	356 (339–373)	403 (364–439)	699 (665–733)	791 (715–861)	6·3% (5·8–6·8)	6·9% (6·2–7·6)	232 (217–248)	59 (56–61)	58 (52–65)	8 (8–8)
	Mexico (UM)	580 (549–617)	594 (556–638)	1194 (1130–1270)	1224 (1146–1313)	5·5% (5·2–5·8)	5·6% (5·2–6·0)	288 (266–308)	49 (38–61)	243 (223–265)	0 (0–0)
	Nicaragua (LM)	179 (175–183)	212 (203–222)	532 (521–544)	631 (602–659)	8·9% (8·7–9·1)	9·3% (8·7–9·8)	105 (102–109)	3 (3–4)	58 (57–60)	12 (12–12)
	Panama (H)	1208 (1178–1236)	1393 (1323–1467)	2638 (2573–2699)	3042 (2888–3203)	7·6% (7·4–7·8)	8·1% (7·5–8·5)	793 (763–821)	77 (73–81)	338 (333–343)	NA
	Paraguay (UM)	365 (341–394)	457 (424–495)	961 (898–1038)	1204 (1119–1304)	7·0% (6·5–7·5)	7·8% (7·2–8·5)	168 (151–185)	43 (32–57)	152 (137–169)	2 (2–2)
	Peru (UM)	333 (309–357)	376 (346–406)	696 (647–745)	785 (723–849)	4·9% (4·5–5·3)	5·0% (4·6–5·5)	208 (192–226)	30 (22–41)	94 (82–109)	1 (1–1)
	Puerto Rico (H)	1199 (1055–1370)	1388 (1217–1584)	1332 (1172–1522)	1542 (1352–1760)	3·4% (2·9–4·0)	3·7% (3·1–4·3)	894 (768–1043)	48 (32–70)	258 (198–334)	NA
	Saint Kitts and Nevis (H)	1005 (957–1053)	998 (937–1058)	1457 (1387–1526)	1447 (1358–1533)	4·9% (4·4–5·4)	4·9% (4·5–5·4)	481 (453–511)	48 (42–53)	477 (438–518)	NA
	Saint Lucia (UM)	538 (518–560)	561 (523–598)	787 (757–819)	820 (764–874)	4·7% (4·5–4·9)	4·9% (4·6–5·2)	244 (238–250)	28 (26–30)	250 (231–270)	16 (16–16)
	Saint Vincent and the Grenadines (UM)	374 (349–397)	456 (421–489)	661 (617–701)	804 (744–864)	4·6% (4·2–5·2)	5·0% (4·5–5·5)	236 (216–255)	11 (7–16)	102 (88–116)	26 (26–26)
	Suriname (UM)	517 (487–550)	464 (427–504)	1787 (1683–1902)	1603 (1475–1741)	8·8% (8·1–9·7)	9·2% (8·3–10·3)	364 (336–396)	57 (48–68)	91 (84–100)	5 (5–5)
	The Bahamas (H)	1940 (1882–2003)	2127 (2037–2228)	2452 (2380–2532)	2689 (2575–2817)	6·1% (5·8–6·3)	6·8% (6·4–7·2)	1020 (968–1072)	412 (387–437)	507 (492–523)	NA
	Trinidad and Tobago (H)	1152 (1098–1207)	1193 (1118–1271)	2031 (1937–2128)	2103 (1972–2241)	6·9% (6·5–7·2)	7·0% (6·5–7·5)	550 (513–586)	79 (73–86)	522 (483–561)	NA
	Venezuela (UM)	243 (220–270)	196 (171–222)	368 (333–409)	297 (259–337)	5·2% (4·0–6·9)	5·8% (4·6–7·3)	117 (102–137)	70 (56–87)	56 (46–66)	0 (0–0)
	Virgin Islands (H)	1390 (1175–1658)	1585 (1329–1914)	1390 (1175–1658)	1585 (1329–1914)	3·4% (2·8–4·1)	3·7% (3·0–4·6)	1034 (836–1270)	52 (34–77)	304 (239–396)	NA
North Africa and Middle East		448 (437–460)	524 (508–540)	963 (944–983)	1130 (1098–1163)	5·5% (5·3–5·6)	5·9% (5·7–6·2)	253 (245–261)	55 (51–59)	138 (132–145)	2 (2–2)
	Afghanistan (L)	84 (79–89)	78 (73–83)	340 (321–362)	315 (296–336)	14·1% (11·4–16·6)	13·7% (11·3–16·1)	5 (4–5)	0 (0–0)	68 (64–74)	11 (11–11)
	Algeria (LM)	246 (228–266)	253 (231–277)	803 (747–870)	826 (757–905)	6·4% (5·9–6·9)	6·7% (6·1–7·3)	161 (146–178)	4 (2–5)	80 (68–93)	0 (0–0)
	Bahrain (H)	1118 (1060–1176)	1179 (1101–1257)	2285 (2167–2404)	2409 (2250–2568)	4·5% (4·0–4·8)	4·7% (4·1–5·3)	661 (614–706)	125 (113–138)	332 (305–362)	NA
	Egypt (LM)	179 (162–196)	214 (193–234)	617 (559–674)	737 (666–806)	5·0% (4·3–5·6)	5·0% (4·3–5·6)	51 (43–60)	17 (12–23)	109 (95–123)	2 (2–2)
	Iran (LM)	1082 (1018–1144)	1293 (1209–1368)	1090 (1026–1153)	1303 (1218–1378)	6·7% (6·3–7·1)	7·3% (6·8–7·7)	518 (475–564)	154 (131–182)	409 (371–453)	0 (0–0)
	Iraq (UM)	250 (230–273)	242 (211–279)	526 (483–574)	509 (443–586)	4·2% (3·8–4·6)	4·3% (3·7–5·0)	113 (96–129)	0 (0–0)	136 (121–151)	1 (1–1)
	Jordan (UM)	309 (286–339)	360 (330–398)	767 (709–840)	893 (817–988)	6·9% (6·4–7·6)	7·2% (6·5–7·9)	153 (136–171)	49 (38–63)	97 (84–110)	9 (9–9)
	Kuwait (H)	1844 (1726–1978)	2064 (1794–2377)	2901 (2715–3112)	3246 (2822–3739)	5·5% (4·9–6·3)	6·1% (5·2–7·2)	1609 (1494–1745)	22 (20–25)	213 (193–232)	NA
	Lebanon (LM)	562 (534–588)	462 (430–497)	1572 (1495–1646)	1293 (1202–1390)	10·1% (9·6–10·6)	10·8% (9·7–12·3)	276 (257–297)	94 (87–100)	188 (174–204)	4 (4–4)
	Libya (UM)	294 (254–339)	380 (313–459)	1028 (888–1184)	1326 (1094–1605)	5·0% (3·1–8·0)	4·9% (3·4–7·4)	208 (172–249)	8 (5–11)	76 (61–96)	1 (1–1)
	Morocco (LM)	189 (172–208)	222 (201–245)	444 (404–488)	520 (471–574)	5·1% (4·6–5·7)	5·6% (5·0–6·2)	78 (66–91)	17 (12–25)	91 (79–104)	3 (3–3)
	Oman (H)	817 (759–878)	839 (744–946)	1441 (1338–1548)	1480 (1312–1668)	4·5% (3·9–5·1)	4·6% (4·0–5·2)	711 (654–772)	54 (50–59)	52 (48–57)	NA
	Palestine (LM)	410 (374–442)	422 (385–458)	208 (189–224)	214 (195–232)	10·5% (9·4–11·6)	11·0% (9·9–12·2)	164 (148–185)	65 (49–83)	171 (151–195)	9 (9–9)
	Qatar (H)	1938 (1808–2099)	2885 (2352–3487)	2957 (2759–3204)	4403 (3590–5322)	3·0% (2·8–3·2)	3·9% (3·2–4·9)	1430 (1293–1581)	284 (259–308)	223 (196–253)	NA
	Saudi Arabia (H)	1362 (1287–1441)	1654 (1494–1821)	2862 (2703–3026)	3475 (3139–3825)	5·5% (5·2–5·9)	6·4% (5·8–7·0)	958 (885–1028)	189 (169–210)	216 (201–229)	NA
	Sudan (L)	47 (42–52)	49 (44–56)	258 (231–290)	273 (244–310)	4·7% (3·3–6·4)	5·0% (3·6–6·5)	10 (9–11)	2 (1–2)	31 (26–37)	4 (4–4)
	Syria (L)	31 (27–34)	31 (28–35)	1372 (1207–1543)	1399 (1236–1582)	2·7% (2·3–3·2)	2·9% (2·5–3·4)	14 (11–17)	1 (0–1)	14 (11–17)	2 (2–2)
	Tunisia (LM)	279 (268–292)	301 (286–315)	826 (791–862)	888 (845–931)	6·7% (6·3–7·2)	7·0% (6·6–7·4)	158 (152–165)	13 (11–16)	106 (97–115)	1 (1–1)
	Turkey (UM)	378 (358–398)	519 (483–554)	1377 (1307–1451)	1893 (1762–2022)	4·4% (4·2–4·6)	4·9% (4·6–5·3)	292 (273–310)	20 (14–28)	64 (61–66)	1 (1–1)
	United Arab Emirates (H)	1983 (1906–2057)	2290 (2131–2461)	3401 (3269–3529)	3928 (3656–4221)	4·5% (4·3–4·7)	4·9% (4·5–5·3)	1042 (982–1107)	693 (666–723)	247 (213–284)	NA
	Yemen (L)	34 (29–39)	38 (33–44)	94 (82–108)	107 (92–121)	4·8% (3·5–7·2)	5·3% (4·2–6·9)	4 (3–5)	0 (0–0)	24 (19–29)	6 (6–6)
South Asia		64 (59–69)	85 (79–91)	206 (192–221)	273 (253–292)	3·0% (2·8–3·2)	3·1% (2·8–3·3)	20 (18–22)	7 (5–9)	36 (33–40)	1 (1–1)
	Bangladesh (LM)	50 (46–55)	69 (63–76)	130 (118–142)	179 (162–196)	2·5% (2·3–2·8)	2·5% (2·2–2·8)	9 (8–11)	1 (1–2)	37 (32–41)	3 (3–3)
	Bhutan (LM)	126 (115–139)	137 (124–152)	467 (428–515)	509 (459–562)	3·6% (3·3–4·0)	3·6% (3·2–4·0)	84 (75–96)	2 (1–2)	18 (14–23)	22 (22–22)
	India (LM)	69 (64–75)	93 (86–102)	223 (205–243)	300 (276–327)	3·0% (2·7–3·3)	3·1% (2·8–3·4)	22 (20–25)	8 (6–11)	39 (34–44)	1 (1–1)
	Nepal (LM)	52 (46–58)	57 (51–65)	187 (166–211)	207 (183–234)	4·4% (3·8–4·9)	4·3% (3·7–5·0)	12 (11–14)	3 (2–5)	30 (24–37)	6 (6–6)
	Pakistan (LM)	42 (35–49)	49 (41–57)	161 (135–189)	187 (158–219)	2·9% (2·4–3·5)	3·1% (2·5–3·7)	13 (11–16)	3 (2–4)	24 (18–30)	2 (2–2)
Southeast Asia, east Asia, and Oceania		460 (445–478)	674 (640–714)	791 (767–819)	1147 (1094–1210)	5·0% (4·9–5·3)	5·6% (5·3–6·0)	259 (245–274)	41 (35–47)	160 (152–168)	1 (1–1)
	American Samoa (UM)	623 (519–730)	697 (583–817)	623 (519–730)	697 (583–817)	4·9% (4·0–5·9)	5·0% (4·0–6·1)	479 (385–582)	21 (14–31)	124 (93–159)	0 (0–0)
	Cambodia (LM)	104 (98–111)	123 (116–132)	313 (293–333)	370 (347–395)	6·4% (5·8–6·9)	6·3% (5·7–6·8)	26 (22–31)	4 (3–6)	67 (62–71)	8 (8–8)
	China (UM)	594 (570–621)	901 (847–964)	925 (888–968)	1404 (1320–1503)	5·3% (5·0–5·6)	5·9% (5·5–6·3)	334 (312–357)	50 (41–60)	210 (198–221)	0 (0–0)
	Cook Islands (H)	815 (750–881)	750 (661–847)	996 (916–1076)	917 (808–1035)	3·4% (3·1–3·7)	3·3% (2·9–3·8)	689 (623–756)	5 (3–6)	51 (46–56)	NA
	Federated States of Micronesia (LM)	176 (163–190)	161 (142–181)	157 (146–170)	144 (127–161)	4·6% (4·1–5·0)	4·2% (3·6–4·8)	143 (130–157)	0 (0–0)	11 (9–12)	22 (22–22)
	Fiji (UM)	228 (211–245)	253 (230–280)	533 (493–574)	593 (539–656)	3·8% (3·3–4·2)	4·2% (3·7–4·8)	149 (136–165)	37 (31–42)	30 (28–34)	11 (11–11)
	Guam (H)	931 (795–1098)	926 (794–1088)	931 (795–1098)	926 (794–1088)	2·3% (1·9–2·7)	2·4% (2·0–3·0)	618 (489–772)	57 (36–87)	256 (197–325)	NA
	Indonesia (LM)	125 (119–131)	163 (150–175)	375 (357–393)	491 (452–528)	2·9% (2·7–3·0)	3·1% (2·9–3·3)	61 (58–65)	19 (17–21)	43 (40–47)	1 (1–1)
	Kiribati (LM)	244 (226–264)	224 (199–250)	292 (270–316)	268 (238–299)	14·0% (13·0–15·2)	12·9% (11·5–14·5)	154 (139–169)	7 (6–9)	49 (38–64)	34 (34–34)
	Laos (LM)	62 (56–67)	70 (63–76)	208 (190–227)	235 (214–258)	2·4% (2·2–2·7)	2·4% (2·2–2·7)	22 (18–27)	0 (0–0)	28 (25–31)	11 (11–11)
	Malaysia (UM)	475 (452–498)	585 (553–621)	1237 (1177–1297)	1523 (1439–1617)	4·0% (3·8–4·2)	4·4% (4·1–4·7)	247 (231–266)	65 (53–82)	163 (158–168)	0 (0–0)
	Maldives (UM)	976 (909–1058)	1212 (1102–1326)	1944 (1810–2106)	2413 (2194–2641)	7·9% (5·9–9·5)	8·6% (7·0–10·2)	757 (689–833)	40 (29–53)	171 (151–193)	8 (8–8)
	Marshall Islands (UM)	658 (630–690)	628 (568–695)	574 (550–603)	548 (496–607)	14·6% (13·5–15·6)	13·6% (12·2–15·1)	332 (307–362)	31 (22–42)	91 (82–100)	203 (203–203)
	Mauritius (UM)	612 (597–628)	694 (666–723)	1554 (1515–1595)	1762 (1690–1835)	6·2% (6·0–6·4)	6·5% (6·2–6·8)	288 (277–300)	42 (40–45)	282 (271–294)	0 (0–0)
	Myanmar (LM)	65 (56–76)	61 (52–70)	239 (205–278)	221 (191–257)	4·4% (3·7–5·2)	4·7% (4·0–5·7)	11 (9–13)	0 (0–0)	51 (42–61)	4 (4–4)
	Nauru (H)	1311 (1214–1412)	1205 (1057–1384)	1329 (1231–1432)	1222 (1072–1404)	10·2% (6·0–15·1)	8·9% (5·4–13·6)	1032 (950–1128)	92 (76–110)	186 (146–238)	NA
	Niue (H)	2642 (2370–2932)	1704 (1355–2152)	1917 (1720–2128)	1237 (983–1562)	12·5% (10·9–14·6)	6·8% (5·2–8·7)	1355 (1088–1645)	62 (39–93)	231 (177–298)	NA
	North Korea (L)	39 (33–46)	40 (34–47)	23 (20–27)	24 (20–28)	5·7% (4·7–6·9)	5·9% (4·7–7·2)	23 (18–28)	1 (0–1)	15 (12–20)	0 (0–0)
	Northern Mariana Islands (H)	451 (378–528)	452 (375–550)	451 (378–528)	452 (375–550)	2·1% (1·7–2·6)	2·5% (2·0–3·2)	288 (226–352)	24 (16–38)	139 (103–183)	NA
	Palau (UM)	1885 (1804–1965)	2200 (2035–2383)	1855 (1776–1934)	2166 (2003–2346)	11·6% (10·9–12·3)	13·5% (12·4–14·6)	1179 (1103–1261)	414 (389–441)	291 (277–304)	0 (0–0)
	Papua New Guinea (LM)	71 (67–76)	74 (67–81)	93 (88–99)	97 (88–106)	2·3% (2·1–2·4)	2·3% (2·1–2·6)	46 (42–50)	0 (0–0)	6 (5–7)	19 (19–19)
	Philippines (LM)	156 (140–173)	194 (173–214)	401 (359–443)	498 (445–549)	4·1% (3·7–4·5)	4·4% (3·9–4·9)	61 (52–71)	18 (12–24)	76 (64–88)	2 (2–2)
	Samoa (LM)	274 (257–293)	266 (246–289)	391 (366–418)	379 (350–412)	6·1% (5·6–6·5)	6·2% (5·7–6·7)	189 (173–206)	2 (1–3)	27 (22–34)	56 (56–56)
	Seychelles (H)	695 (672–718)	845 (778–914)	1490 (1440–1539)	1811 (1667–1959)	4·7% (4·4–5·0)	5·0% (4·5–5·7)	509 (496–523)	12 (10–14)	174 (156–194)	NA
	Solomon Islands (LM)	114 (106–124)	124 (101–153)	119 (110–128)	129 (104–159)	4·5% (4·2–4·9)	5·3% (4·1–6·5)	80 (71–89)	0 (0–0)	4 (3–4)	31 (31–31)
	Sri Lanka (LM)	154 (140–169)	148 (134–164)	584 (531–640)	563 (509–623)	4·0% (3·6–4·4)	4·2% (3·8–4·7)	70 (63–78)	10 (6–14)	71 (59–83)	3 (3–3)
	Taiwan (province of China) (H)	1556 (1468–1652)	2161 (2028–2309)	2880 (2717–3058)	4002 (3754–4274)	5·1% (4·8–5·5)	5·7% (5·3–6·2)	1196 (1148–1243)	58 (38–85)	302 (234–386)	NA
	Thailand (UM)	310 (304–315)	398 (384–414)	811 (797–825)	1043 (1005–1085)	4·0% (3·9–4·1)	4·7% (4·5–4·9)	220 (216–223)	60 (58–62)	30 (26–33)	1 (1–1)
	Timor-Leste (LM)	117 (106–131)	127 (110–150)	193 (174–216)	209 (181–246)	5·1% (4·6–5·7)	4·9% (4·2–5·7)	80 (70–93)	6 (4–10)	11 (9–15)	19 (19–19)
	Tokelau (UM)	2055 (1865–2255)	2133 (1885–2404)	2684 (2436–2945)	2786 (2462–3139)	22·9% (20·6–25·5)	19·6% (17·2–22·2)	743 (598–914)	63 (41–93)	383 (289–495)	867 (867–867)
	Tonga (UM)	209 (200–218)	214 (201–226)	270 (259–283)	277 (261–293)	4·2% (4·0–4·5)	4·1% (3·7–4·6)	140 (134–146)	9 (7–12)	25 (20–31)	35 (35–35)
	Tuvalu (UM)	969 (928–1004)	1209 (1034–1432)	885 (848–918)	1105 (945–1308)	18·5% (16·9–19·9)	19·4% (16·2–22·9)	755 (719–788)	0 (0–0)	84 (64–106)	130 (130–130)
	Vanuatu (LM)	106 (95–118)	92 (76–113)	94 (85–105)	82 (68–101)	3·0% (2·7–3·4)	2·9% (2·3–3·6)	66 (55–78)	4 (3–6)	10 (8–12)	25 (25–25)
	Vietnam (LM)	180 (162–200)	254 (225–287)	558 (501–618)	788 (698–888)	5·5% (4·4–7·2)	5·8% (4·7–7·5)	80 (69–93)	20 (13–28)	79 (66–93)	2 (2–2)
Sub-Saharan Africa		82 (80–83)	86 (83–89)	193 (188–197)	205 (198–212)	4·8% (4·6–5·0)	4·9% (4·7–5·1)	33 (32–34)	15 (14–16)	23 (22–24)	10 (10–10)
	Angola (LM)	68 (60–78)	66 (57–75)	203 (179–230)	195 (170–222)	2·7% (2·3–3·1)	2·8% (2·4–3·3)	29 (23–34)	12 (8–17)	24 (19–30)	3 (3–3)
	Benin (LM)	33 (29–37)	38 (34–43)	89 (79–99)	103 (91–117)	2·4% (2·2–2·7)	2·4% (2·1–2·7)	7 (6–8)	2 (1–3)	14 (11–18)	11 (11–11)
	Botswana (UM)	503 (474–534)	559 (511–608)	1205 (1134–1280)	1340 (1225–1456)	6·5% (6·1–7·0)	6·7% (6·2–7·4)	389 (364–415)	67 (52–84)	15 (14–17)	32 (32–32)
	Burkina Faso (L)	49 (46–52)	57 (53–62)	137 (128–145)	158 (146–171)	5·7% (5·4–6·1)	5·8% (5·3–6·2)	19 (17–22)	3 (2–5)	15 (14–16)	12 (12–12)
	Burundi (L)	32 (30–34)	36 (32–42)	93 (88–99)	106 (93–123)	10·7% (9·2–12·0)	12·0% (9·9–14·4)	7 (6–8)	4 (3–5)	5 (5–6)	16 (16–16)
	Cameroon (LM)	50 (48–53)	58 (55–62)	124 (118–130)	144 (135–153)	3·0% (2·9–3·2)	3·2% (3·0–3·5)	3 (2–3)	5 (3–6)	36 (34–39)	7 (7–7)
	Cape Verde (LM)	231 (217–248)	262 (234–291)	468 (439–502)	530 (475–589)	5·9% (5·6–6·4)	6·3% (5·6–6·9)	127 (112–143)	5 (4–7)	50 (48–53)	49 (49–49)
	Central African Republic (L)	43 (41–45)	47 (42–52)	83 (79–87)	91 (82–101)	8·0% (7·6–8·3)	8·2% (7·4–9·1)	4 (3–4)	0 (0–1)	21 (19–23)	17 (17–17)
	Chad (L)	34 (32–36)	33 (30–35)	77 (73–81)	75 (70–80)	4·3% (4·0–4·7)	4·4% (4·0–4·8)	5 (5–6)	2 (1–2)	19 (17–21)	7 (7–7)
	Comoros (LM)	108 (105–112)	147 (135–165)	244 (235–251)	332 (303–372)	7·1% (6·2–7·8)	9·4% (7·8–11·1)	13 (11–16)	4 (3–6)	58 (55–61)	33 (33–33)
	Congo (Brazzaville) (LM)	60 (53–67)	59 (50–67)	96 (86–107)	95 (81–108)	2·1% (1·8–2·5)	2·4% (2·0–2·8)	21 (18–25)	4 (3–6)	26 (21–32)	8 (8–8)
	Côte d'Ivoire (LM)	78 (70–86)	104 (93–116)	184 (166–203)	245 (221–274)	3·1% (2·8–3·5)	3·4% (3·0–3·8)	23 (22–24)	16 (10–23)	30 (25–35)	9 (9–9)
	DR Congo (L)	25 (24–27)	29 (26–32)	50 (48–53)	57 (52–64)	4·2% (4·0–4·4)	4·1% (3·7–4·6)	3 (3–4)	2 (1–2)	9 (8–10)	12 (12–12)
	Djibouti (LM)	60 (59–62)	72 (66–78)	104 (101–107)	124 (114–134)	1·7% (1·7–1·8)	1·7% (1·5–1·8)	28 (27–29)	1 (1–1)	13 (12–15)	18 (18–18)
	Equatorial Guinea (UM)	306 (277–338)	241 (218–268)	608 (551–672)	480 (434–533)	3·0% (2·7–3·3)	3·3% (2·9–3·9)	58 (52–64)	7 (6–10)	228 (198–261)	12 (12–12)
	Eritrea (L)	16 (14–18)	21 (18–24)	49 (44–55)	64 (56–73)	2·5% (2·3–2·8)	3·1% (2·7–3·5)	3 (2–3)	0 (0–0)	7 (5–8)	7 (7–7)
	Eswatini (LM)	282 (269–296)	356 (318–399)	665 (636–700)	840 (752–941)	6·9% (6·6–7·4)	8·3% (7·3–9·3)	138 (131–146)	37 (28–48)	29 (23–36)	77 (77–77)
	Ethiopia (L)	25 (24–27)	31 (29–33)	80 (75–85)	98 (91–106)	3·1% (2·6–3·5)	2·9% (2·5–3·4)	7 (6–8)	2 (1–3)	10 (9–11)	7 (7–7)
	Gabon (UM)	335 (317–355)	395 (372–419)	618 (585–655)	728 (686–772)	3·7% (3·5–4·0)	4·2% (3·8–4·6)	186 (171–200)	48 (46–50)	71 (59–84)	31 (31–31)
	Ghana (LM)	76 (71–81)	90 (84–97)	193 (181–206)	229 (213–246)	3·6% (2·9–4·8)	3·6% (3·0–4·6)	30 (27–34)	9 (9–10)	28 (25–32)	8 (8–8)
	Guinea (L)	52 (45–60)	67 (57–78)	117 (102–135)	152 (130–177)	3·9% (3·2–5·0)	4·2% (3·4–5·1)	8 (7–10)	4 (3–6)	31 (24–38)	8 (8–8)
	Guinea-Bissau (L)	68 (63–72)	80 (74–87)	199 (185–213)	236 (217–254)	8·4% (7·4–9·5)	9·1% (7·7–10·5)	4 (3–5)	2 (1–3)	39 (34–43)	23 (23–23)
	Kenya (LM)	91 (83–100)	113 (102–125)	227 (207–250)	283 (255–314)	4·4% (3·9–5·0)	4·7% (4·1–5·4)	40 (33–49)	11 (8–14)	22 (18–27)	17 (17–17)
	Lesotho (LM)	132 (127–138)	153 (141–167)	313 (301–327)	364 (334–396)	10·5% (10·1–11·0)	12·0% (10·7–13·4)	70 (65–76)	1 (0–1)	21 (20–22)	40 (40–40)
	Liberia (L)	60 (56–64)	88 (78–100)	136 (128–145)	200 (176–226)	9·3% (7·3–12·8)	12·4% (9·3–16·7)	7 (6–9)	3 (2–5)	27 (24–31)	22 (22–22)
	Madagascar (L)	20 (18–23)	22 (19–24)	66 (60–74)	71 (63–79)	3·8% (3·4–4·2)	3·9% (3·5–4·4)	9 (8–10)	1 (1–2)	6 (5–8)	4 (4–4)
	Malawi (L)	44 (42–45)	53 (48–58)	116 (113–120)	140 (128–155)	8·0% (6·3–10·5)	8·9% (6·7–11·8)	11 (10–12)	2 (2–3)	6 (5–6)	25 (25–25)
	Mali (L)	32 (30–34)	33 (30–36)	86 (80–92)	87 (81–95)	3·1% (2·4–3·7)	3·0% (2·3–3·7)	11 (10–12)	1 (0–1)	11 (9–12)	10 (10–10)
	Mauritania (LM)	72 (69–76)	83 (78–88)	212 (202–223)	245 (228–260)	3·6% (3·1–4·0)	3·7% (3·1–4·3)	26 (24–29)	4 (3–6)	32 (30–34)	9 (9–9)
	Mozambique (L)	31 (28–34)	39 (35–43)	83 (76–90)	104 (94–116)	5·9% (5·4–6·4)	6·7% (6·0–7·5)	9 (7–11)	3 (2–4)	4 (3–5)	15 (15–15)
	Namibia (UM)	486 (458–518)	489 (456–528)	977 (920–1042)	984 (917–1062)	9·0% (8·5–9·6)	9·6% (8·9–10·3)	226 (209–247)	193 (173–216)	40 (32–50)	26 (26–26)
	Niger (L)	37 (35–39)	43 (40–46)	81 (78–85)	94 (88–101)	6·0% (5·7–6·3)	5·6% (5·1–6·1)	12 (10–13)	1 (1–2)	16 (15–17)	8 (8–8)
	Nigeria (LM)	62 (57–68)	63 (57–69)	163 (148–177)	165 (149–182)	2·9% (2·6–3·1)	2·9% (2·6–3·2)	10 (8–12)	1 (0–1)	47 (43–53)	4 (4–4)
	Rwanda (L)	56 (52–61)	63 (57–71)	166 (154–181)	187 (168–210)	6·7% (6·2–7·4)	6·2% (5·4–7·1)	20 (17–25)	8 (6–10)	6 (5–7)	22 (22–22)
	Senegal (LM)	71 (63–79)	86 (77–97)	166 (149–186)	202 (181–227)	4·4% (4·0–5·0)	4·3% (3·8–4·9)	17 (14–21)	4 (3–6)	36 (29–43)	13 (13–13)
	Sierra Leone (L)	45 (43–48)	67 (60–76)	159 (149–169)	235 (209–266)	8·5% (7·9–9·0)	11·5% (10·2–13·0)	5 (4–7)	1 (0–1)	24 (22–27)	15 (15–15)
	Somalia (L)	7 (6–7)	8 (7–10)	18 (17–19)	22 (18–26)	4·2% (3·8–4·6)	5·5% (4·6–6·8)	1 (0–1)	0 (0–0)	2 (1–2)	4 (4–4)
	South Africa (UM)	631 (605–656)	676 (639–711)	1307 (1253–1359)	1400 (1324–1472)	9·0% (8·3–10·0)	9·9% (9·1–10·8)	367 (355–378)	219 (197–242)	36 (34–38)	9 (9–9)
	South Sudan (L)	41 (39–42)	36 (32–41)	94 (91–98)	83 (74–95)	9·8% (4·5–15·8)	9·6% (2·9–24·9)	4 (3–5)	2 (1–2)	7 (6–8)	28 (28–28)
	São Tomé and Príncipe (LM)	189 (177–202)	275 (249–306)	339 (318–362)	493 (447–549)	8·2% (7·7–8·8)	11·2% (10·1–12·5)	60 (50–71)	2 (1–3)	22 (18–28)	104 (104–104)
	Tanzania (LM)	42 (41–44)	49 (44–54)	111 (107–116)	128 (116–142)	3·8% (3·6–4·0)	3·8% (3·4–4·2)	18 (17–20)	0 (0–1)	10 (9–11)	14 (14–14)
	The Gambia (L)	42 (40–44)	57 (50–63)	125 (118–132)	169 (149–189)	5·0% (4·7–5·4)	6·1% (5·4–6·8)	9 (7–11)	1 (1–2)	7 (6–9)	24 (24–24)
	Togo (L)	59 (55–64)	75 (69–81)	141 (131–152)	179 (165–194)	6·8% (5·5–8·9)	7·2% (5·8–9·2)	9 (7–11)	5 (3–7)	37 (34–41)	8 (8–8)
	Uganda (L)	41 (38–44)	46 (42–51)	112 (104–122)	127 (116–139)	4·2% (3·7–4·8)	4·3% (3·8–4·8)	7 (6–8)	2 (1–2)	15 (12–18)	18 (18–18)
	Zambia (L)	60 (55–66)	67 (58–77)	199 (184–219)	223 (194–255)	5·2% (4·6–5·7)	5·9% (5·1–6·7)	23 (19–28)	4 (2–5)	6 (5–8)	28 (28–28)
	Zimbabwe (LM)	54 (50–59)	59 (53–65)	60 (56–66)	65 (59–73)	2·5% (2·3–2·7)	2·5% (2·2–2·8)	12 (10–14)	11 (8–15)	11 (9–14)	20 (20–20)

Estimates in parentheses are 95% uncertainty intervals. Venezuela estimates are presented in 2014 US$. Spending reported in 2021 inflation-adjusted US$. GBD=Global Burden of Diseases, Injuries, and Risk Factors Study. GDP=gross domestic product. H=high-income. UM=upper-middle income. LM=lower-middle income. L=low-income. NA=not applicable due to country not receiving development assistance.

**Figure 1:**
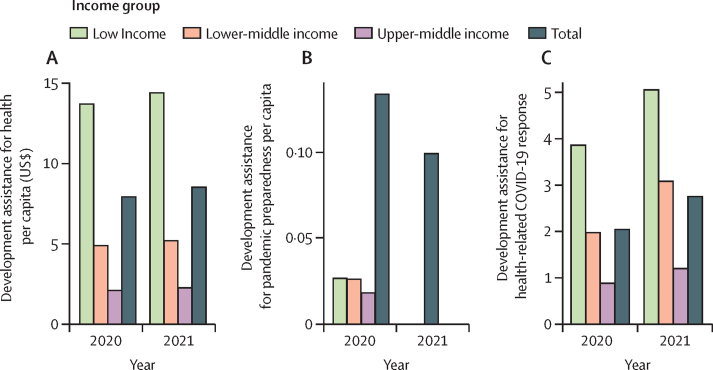
Per-person development assistance for health (A), per-person development assistance for pandemic preparedness (B), and per-person COVID-19 development assistance for health spending (C), by World Bank income groups Spending reported in 2021 inflation-adjusted US$.

Across income groups, development assistance available for pandemic preparedness, health-related COVID-19 response, and health in general differs substantially (figure 1). For low-income countries in 2020 (the last year with disaggregated pandemic preparedness data), average per-person assistance for pandemic preparedness was $0·04, for the COVID-19 health response was $4, and for total DAH was $14. For upper-middle-income countries, average per-person assistance for pandemic preparedness was $0·01, for COVID-19 was $1, and for overall development assistance was $2.

**Figure 2:**
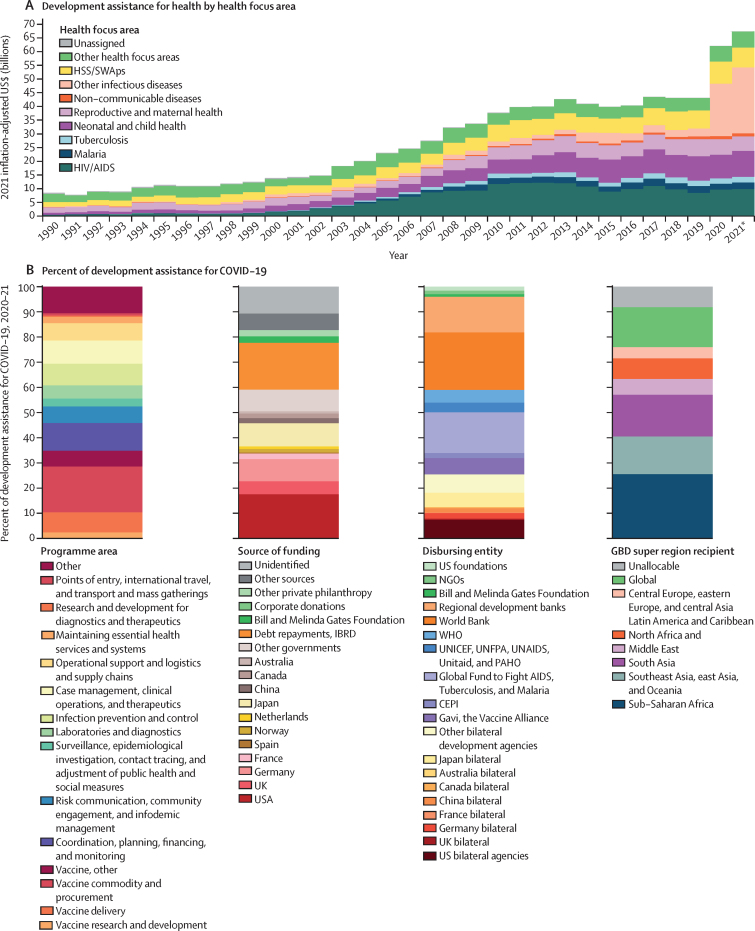
Development assistance for health, by health focus area, 1990–2021 (A), and percentage of development assistance for COVID-19, 2020–21 (B) In A, Other health focus areas captures development assistance for health for which we have health focus area information but is not identified as being allocated to any of the health focus areas listed. Health assistance for which we have no health focus area information is designated as Unassigned. In B, Other sources captures development assistance for health for which we have source information but which is not identified as originating within any of the sources listed. Other governments includes Afghanistan, Angola, Argentina, Austria, Azerbaijan, Bangladesh, Belgium, Bhutan, Brazil, Brunei, Bulgaria, Côte d'Ivoire, Cameroon, Central African Republic, Chad, Colombia, Croatia, Czechia, DR Congo, Denmark, Egypt, Estonia, Ethiopia, Finland, Gabon, Greece, Guinea, Hungary, Iceland, India, Indonesia, Iran, Iraq, Ireland, Italy, Jamaica, Jordan, Kenya, Kuwait, Latvia, Lebanon, Libya, Lithuania, Luxembourg, Madagascar, Malaysia, Malta, Monaco, Myanmar, New Zealand, Nigeria, Oman, Pakistan, Palestine, Peru, Poland, Portugal, Qatar, Romania, Russia, São Tomé and Príncipe, Saudi Arabia, Serbia, Singapore, Slovakia, Slovenia, South Africa, South Korea, South Sudan, Sudan, Sweden, Switzerland, Syria, Taiwan (province of China), Thailand, Togo, Türkiye, Uganda, Ukraine, United Arab Emirates, Yemen, and Zimbabwe. Health assistance for which we have no source information is designated as Unidentified. Regional development banks include the African Development Bank, the Asian Development Bank, and the Inter-American Development Bank. Other bilateral development agencies include Austria, Belgium, Denmark, Finland, Greece, Ireland, Italy, Luxembourg, Netherlands, New Zealand, Norway, South Korea, Spain, Sweden, Switzerland, United Arab Emirates, the European Commission, and the European Economic Area. Argentina is included in the Global Burden of Disease high-income classification but has been included in Latin America and Caribbean because Argentina was considered a middle-income country by the World Bank in 2020 and 2021. Health assistance for which no recipient country of regional information is available is designated as Unallocable. CEPI=Coalition for Epidemic Preparedness Innovations. GBD=Global Burden of Diseases, Injuries, and Risk Factors Study. HSS/SWAps=health system strengthening and sector-wide approaches. IBRD=International Bank for Reconstruction and Development. NGOs=non-governmental organisations. PAHO=Pan American Health Organization. UNFPA=United Nations Population Fund. Global refers to donor resources that were contributed towards goods or activities that are of benefit to the world; these types of goods or activities are more commonly referred to as global public goods. Spending reported in 2021 inflation-adjusted US$. *2021 disbursement estimates are preliminary.

In 2019, $43·1 billion in development assistance was provided to maintain or improve health. The COVID-19 pandemic initiated an unprecedented growth of resources targeted towards global health activities (figure 2A). In 2020 and 2021, $1·8 billion in DAH contributions was provided towards pandemic preparedness in LMICs, and $37·8 billion was provided for the health-related COVID-19 response. After a decade of relative stagnation (a 1·5% annualised growth rate in total DAH from 2010 to 2019), we see an increase in disbursements to $62·1 billion in 2020 (43·9% increase on the $43·1 billion in 2019) and to $67·4 billion in 2021 (8·6% increase from 2020). In 2019, at the onset of the pandemic, the health focus areas with the most disbursements were neonatal and child health, HIV/AIDS, and sector-wide approaches and health systems strengthening. Between 2019 and 2021, development assistance for other infectious diseases has increased from $2·9 billion in 2019 to $24·0 billion in 2021 (733·1%), and for HIV/AIDS has increased from $8·5 billion in 2019 to $9·9 billion in 2021 (16·8%), while development assistance for reproductive and maternal health has decreased from $6·2 billion in 2019 to $5·3 billion in 2021 (14·1%) and for malaria has decreased from $2·5 billion in 2019 to $2·4 billion in 2021 (3·2%).

In 2021, $21·8 billion was provided in development assistance funding for the health response to COVID-19 (figure 2A). This amount is 1·4 times the amount provided in 2020 ($16·0 billion). The main sources of development assistance for the health response for COVID-19 in 2021 were the USA ($5·2 billion), Germany ($1·8 billion), and Japan ($1·5 billion). These funds were disbursed primarily through the World Bank ($5·6 billion), the Global Fund to Fight AIDS, Tuberculosis and Malaria ($5·1 billion), and US bilateral agencies ($2·2 billion; figure 2B). Of the development assistance for COVID-19 response that can be traced to a specific country or region recipient, much was directed towards activities in sub-Saharan Africa (35·7%), south Asia (21·2%), and southeast Asia, east Asia, and Oceania (19·9%).

**Figure 3:**
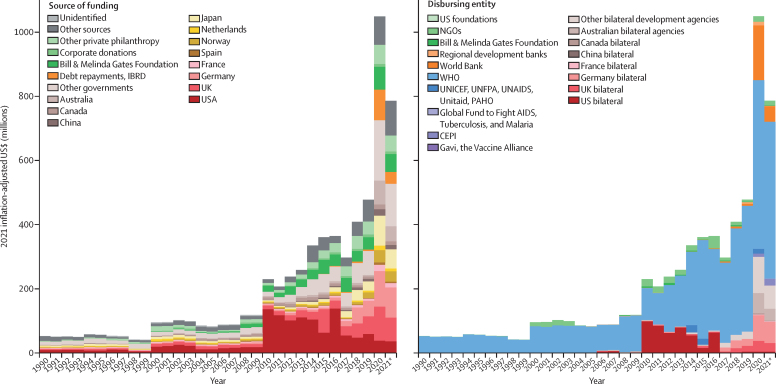
Development assistance for pandemic preparedness by source of funding (A) and disbursing entity (B), 1990–21 Other sources captures development assistance for health for which we have source information but which is not identified as originating within any of the sources listed. Other governments includes Afghanistan, Angola, Argentina, Austria, Azerbaijan, Bangladesh, Belgium, Bhutan, Brazil, Brunei, Bulgaria, Côte d'Ivoire, Cameroon, Central African Republic, Chad, Colombia, Croatia, Czechia, DR Congo, Denmark, Egypt, Estonia, Ethiopia, Finland, Gabon, Greece, Guinea, Hungary, Iceland, India, Indonesia, Iran, Iraq, Ireland, Italy, Jamaica, Jordan, Kenya, Kuwait, Latvia, Lebanon, Libya, Lithuania, Luxembourg, Madagascar, Malaysia, Malta, Monaco, Myanmar, New Zealand, Nigeria, Oman, Pakistan, Palestine, Peru, Poland, Portugal, Qatar, Romania, Russia, São Tomé and Príncipe, Saudi Arabia, Serbia, Singapore, Slovakia, Slovenia, South Africa, South Korea, South Sudan, Sudan, Sweden, Switzerland, Syria, Taiwan (province of China), Thailand, Togo, Türkiye, Uganda, Ukraine, United Arab Emirates, Yemen, and Zimbabwe. Health assistance for which we have no source information is designated as Unidentified. Regional development banks include the African Development Bank, the Asian Development Bank, and the Inter-American Development Bank. Other bilateral development agencies include Austria, Belgium, Denmark, Finland, Greece, Ireland, Italy, Luxembourg, Netherlands, New Zealand, Norway, South Korea, Spain, Sweden, Switzerland, United Arab Emirates, the European Commission, and the European Economic Area. CEPI=Coalition for Epidemic Preparedness Innovations. GBD=Global Burden of Diseases, Injuries, and Risk Factors Study. HSS/SWAps=health system strengthening and sector-wide approaches. IBRD=International Bank for Reconstruction and Development. NGO=non-governmental organisation. PAHO=Pan American Health Organization. UNFPA=United Nations Population Fund. Spending reported in 2021 inflation-adjusted USD. *2021 disbursement estimates are preliminary.

Development assistance contributions towards pandemic preparedness remain small, although they have been increasing over time (figure 3). In 2021, a total of $786·6 million was contributed towards this activity. For perspective, this value was a 1394·5% increase relative to the amount contributed in 1990 ($52·6 million), a 64·8% increase relative to 2019 ($477·4 million), and a 33·4% decrease relative to 2020 ($1049·6 million). Between 2000 and 2021, a cumulative total of $6·0 billion in funds was invested by international donors for pandemic preparedness. We found that the predominant sources of pandemic preparedness and response funds are the USA ($1·3 billion), the UK ($597·3 million), and the Bill & Melinda Gates Foundation ($474·9 million; figure 3A). The international development agencies that have been responsible for disbursing these funds include WHO ($4·7 billion), US bilateral agencies ($480·7 million), and the World Bank ($240·2 million; figure 3B).

A large fraction of the available development assistance for pandemic preparedness was targeted towards global activities in contrast to regional or national activities (appendix p 118). In 2021, $20·7 million (2·6% of the $786·6 million overall funding available for which we were able to determine target destination), was dedicated to global activities. This figure is in comparison with $2·4 million (0·3% of the $786·6 million overall funding) that targeted pandemic preparedness activities in specific countries. Importantly, $763·5 million (97·1%) of the allocable funding did not include the geographical detail that would allow for disaggregating to global, regional, or national activities.

**Figure 4:**
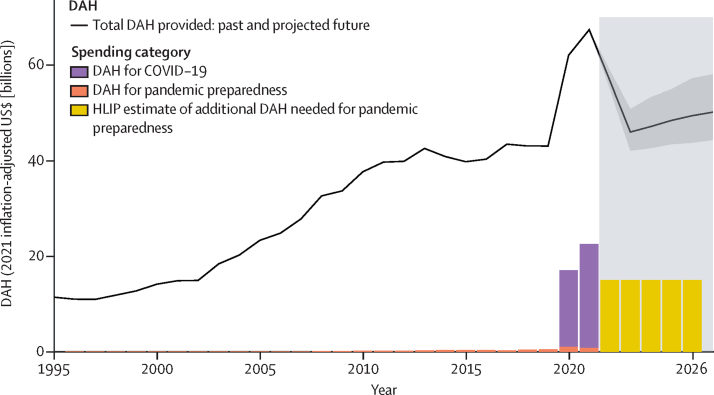
Development assistance for pandemic preparedness and COVID-19, 1995–2026 Projected future pandemic preparedness needs come from the report Financing the Global Commons for Pandemic Preparedness and Response.^10^ Spending reported in 2021 inflation-adjusted US$. DAH=development assistance for health. HLIP=High-Level Independent Panel. LMIC=low-income or middle-income country.

There are substantial gaps between estimated resource needs for adequate preparation against the next pandemic and the resources that are currently allocated to pandemic preparedness. According to the HLIP, an additional $15 billion should be invested by development partners annually, and national governments should dedicate an additional 1% of their country's GDP towards health, inclusive of the tools and surveillance necessary to prevent another pandemic. Our current estimates suggest that in 2021, a total of $22·6 billion in development assistance was provided towards pandemic preparedness and COVID-19 health-related response efforts (figure 4). This amount is 150·5% of the proposed additional need ($15 billion), meaning that the aggregate 2021 development assistance provided for pandemic preparedness and the health-related response to the COVID-19 pandemic more than exceeded the amount that is recommended by the HLIP to be spent on pandemic preparedness investment. Separately, the support for pandemic preparedness is 12·2% of the recommended target by the HLIP while the support provided for the health-related COVID-19 response is 252·2% of the recommended target. Our projections suggest that, of the 137 LMICs included in the study, only 17 (12%) would meet the recommended spending target for national governments in 5 years (table 2).

**Table 2:** Low-income and middle-income countries' projected government health spending in 2026 versus HLIP projected need

	**2021 government health spending per person**	**2026 government health spending per person**	HLIP 2026 government health spending per person recommended target*	**Percentage difference in HLIP recommended target and projected 2026 government health spending per person**†
Afghanistan	6 (6–7)	5 (4–6)	12 (10–13)	42%
Albania	229 (207–254)	236 (205–269)	294 (270–318)	81%
Algeria	157 (142–172)	183 (161–205)	194 (179–209)	94%
American Samoa	502 (403–612)	572 (459–700)	637 (538–750)	90%
Angola	34 (28–39)	30 (24–37)	56 (51–62)	54%
Argentina	661 (631–692)	733 (671–801)	769 (738–800)	95%
Armenia	86 (80–91)	80 (72–91)	133 (127–139)	60%
Azerbaijan	89 (82–97)	67 (60–76)	143 (136–151)	47%
Bangladesh	16 (14–17)	13 (11–16)	37 (36–39)	35%
Belarus	315 (292–340)	308 (283–338)	388 (366–413)	79%
Belize	167 (150–185)	200 (170–227)	210 (192–229)	95%
Benin	18 (17–20)	10 (8–12)	32 (31–34)	30%
Bhutan	113 (100–128)	100 (88–114)	145 (131–161)	69%
Bolivia (Plurinational State of)	169 (164–175)	210 (197–223)	203 (197–209)	104%
Bosnia and Herzegovina	478 (454–500)	533 (503–562)	545 (522–569)	98%
Botswana	386 (362–411)	468 (419–517)	461 (437–486)	102%
Brazil	361 (332–391)	339 (306–375)	436 (407–466)	78%
Bulgaria	589 (557–618)	564 (504–617)	706 (674–735)	80%
Burkina Faso	29 (26–32)	28 (23–32)	37 (35–40)	73%
Burundi	10 (9–11)	8 (7–10)	13 (11–14)	67%
Cabo Verde	144 (129–160)	153 (126–181)	178 (164–194)	86%
Cambodia	34 (30–40)	32 (27–38)	50 (46–56)	63%
Cameroon	4 (4–5)	3 (2–4)	21 (20–21)	15%
Central African Republic	7 (6–8)	4 (3–6)	12 (11–13)	36%
Chad	9 (8–10)	6 (5–8)	16 (15–17)	38%
China	373 (345–399)	539 (482–598)	496 (469–522)	109%
Colombia	376 (358–398)	479 (445–519)	438 (419–460)	110%
Comoros	35 (33–38)	16 (13–19)	50 (47–54)	31%
Congo	18 (14–22)	20 (15–27)	42 (37–47)	49%
Costa Rica	730 (694–765)	965 (910–1025)	853 (819–888)	113%
Cuba	1144 (1059–1221)	1344 (1243–1446)	1246 (1157–1329)	108%
Côte d'Ivoire	32 (30–33)	34 (32–37)	57 (56–59)	60%
Democratic People's Republic of Korea	22 (18–27)	25 (20–30)	29 (25–34)	85%
Democratic Republic of the Congo	3 (3–4)	5 (4–7)	10 (9–10)	53%
Djibouti	43 (42–44)	36 (31–40)	79 (77–80)	45%
Dominica	364 (334–394)	325 (290–363)	439 (409–470)	74%
Dominican Republic	288 (264–312)	308 (262–358)	375 (352–401)	82%
Ecuador	288 (261–316)	356 (316–396)	347 (321–375)	103%
Egypt	55 (47–64)	65 (55–78)	92 (82–101)	71%
El Salvador	229 (218–241)	229 (211–246)	272 (261–284)	84%
Equatorial Guinea	78 (73–85)	51 (42–63)	169 (162–176)	30%
Eritrea	3 (2–4)	4 (3–5)	9 (9–10)	38%
Eswatini	147 (139–155)	157 (139–177)	187 (178–196)	84%
Ethiopia	10 (9–11)	8 (7–10)	19 (17–20)	45%
Fiji	147 (134–162)	169 (148–195)	196 (182–212)	86%
Gabon	246 (230–262)	243 (222–265)	333 (317–349)	73%
Gambia	8 (7–10)	12 (10–15)	16 (15–18)	74%
Georgia	213 (196–232)	189 (160–225)	263 (245–281)	72%
Ghana	45 (41–50)	40 (36–46)	67 (61–74)	60%
Grenada	295 (269–324)	235 (212–258)	394 (368–422)	60%
Guatemala	143 (137–150)	152 (137–167)	191 (183–197)	80%
Guinea	15 (13–17)	12 (10–16)	29 (26–32)	42%
Guinea-Bissau	26 (25–27)	5 (4–7)	34 (32–36)	15%
Guyana	1034 (1027–1042)	687 (615–775)	1138 (1125–1150)	60%
Haiti	12 (11–14)	8 (6–10)	30 (29–32)	27%
Honduras	99 (92–108)	95 (82–108)	127 (119–136)	75%
India	28 (26–30)	33 (30–36)	51 (49–53)	65%
Indonesia	109 (104–114)	87 (75–98)	152 (147–157)	57%
Iran (Islamic Republic of)	508 (458–551)	684 (621–744)	675 (625–720)	101%
Iraq	114 (97–138)	127 (97–163)	165 (147–189)	76%
Jamaica	244 (228–262)	252 (217–286)	297 (281–316)	85%
Jordan	167 (148–187)	195 (168–223)	211 (192–232)	92%
Kazakhstan	232 (211–253)	205 (180–232)	332 (312–354)	62%
Kenya	43 (35–52)	56 (47–68)	63 (56–72)	89%
Kiribati	163 (151–176)	143 (128–163)	180 (168–193)	80%
Kyrgyzstan	32 (26–39)	34 (27–43)	45 (39–52)	76%
Lao People's Democratic Republic	22 (18–27)	26 (21–32)	47 (43–53)	55%
Lebanon	136 (123–149)	237 (208–269)	174 (161–187)	136%
Lesotho	78 (73–83)	73 (63–82)	90 (85–95)	81%
Liberia	10 (8–12)	10 (7–13)	16 (13–19)	58%
Libya	168 (137–202)	273 (208–350)	235 (190–280)	116%
Madagascar	10 (9–11)	10 (8–12)	15 (14–16)	68%
Malawi	13 (12–15)	13 (11–15)	19 (17–21)	68%
Malaysia	258 (241–278)	337 (311–367)	371 (354–391)	91%
Maldives	811 (747–878)	1052 (931–1168)	916 (844–1006)	115%
Mali	14 (13–15)	13 (11–14)	24 (22–27)	53%
Marshall Islands	367 (326–410)	368 (308–434)	409 (368–454)	90%
Mauritania	35 (33–38)	32 (28–36)	54 (51–58)	59%
Mauritius	280 (268–293)	339 (313–364)	368 (356–381)	92%
Mexico	305 (284–326)	310 (283–340)	405 (383–426)	77%
Micronesia (Federated States of)	161 (145–180)	152 (133–172)	197 (181–216)	77%
Mongolia	152 (144–161)	124 (113–135)	197 (189–206)	63%
Montenegro	485 (446–523)	577 (524–630)	578 (539–616)	100%
Morocco	95 (82–109)	102 (86–119)	131 (118–145)	78%
Mozambique	13 (11–15)	11 (8–13)	18 (16–20)	60%
Myanmar	13 (11–15)	11 (9–14)	24 (22–27)	46%
Namibia	231 (213–252)	230 (206–259)	280 (262–301)	82%
Nepal	29 (27–30)	14 (13–16)	40 (38–41)	36%
Nicaragua	118 (113–125)	135 (125–145)	140 (134–146)	97%
Niger	13 (12–15)	19 (16–22)	19 (18–21)	101%
Nigeria	20 (18–22)	12 (8–17)	40 (38–43)	29%
North Macedonia	371 (346–396)	369 (342–397)	439 (413–463)	84%
Pakistan	17 (14–20)	18 (14–22)	31 (28–35)	56%
Palau	1366 (1260–1490)	1406 (1239–1587)	1488 (1381–1612)	94%
Palestine	157 (141–178)	177 (159–202)	193 (177–213)	92%
Papua New Guinea	53 (49–58)	53 (46–60)	83 (79–88)	64%
Paraguay	232 (214–250)	240 (213–267)	284 (266–303)	84%
Peru	259 (242–278)	248 (225–273)	327 (309–346)	76%
Philippines	77 (68–88)	88 (74–103)	113 (104–124)	78%
Republic of Moldova	164 (145–184)	185 (163–210)	211 (183–237)	88%
Russian Federation	450 (425–477)	421 (388–458)	573 (547–600)	74%
Rwanda	31 (27–35)	29 (23–36)	39 (35–44)	74%
Saint Lucia	244 (233–256)	266 (238–295)	340 (328–352)	78%
Saint Vincent and the Grenadines	265 (242–288)	261 (236–286)	342 (320–366)	76%
Samoa	195 (181–211)	191 (173–209)	234 (220–250)	81%
Sao Tome and Principe	67 (55–80)	71 (58–85)	91 (79–103)	79%
Senegal	27 (24–31)	24 (19–29)	44 (40–48)	55%
Serbia	442 (418–465)	434 (402–467)	533 (510–557)	81%
Sierra Leone	9 (8–11)	7 (5–9)	14 (13–16)	47%
Solomon Islands	84 (71–99)	82 (56–111)	107 (94–122)	76%
Somalia	1 (1–1)	1 (0–1)	2 (2–3)	25%
South Africa	389 (376–402)	394 (372–417)	456 (441–471)	86%
South Sudan	3 (3–4)	5 (4–6)	8 (5–12)	68%
Sri Lanka	77 (70–86)	68 (59–79)	115 (107–123)	59%
Sudan	11 (10–12)	13 (10–16)	20 (18–24)	64%
Suriname	231 (209–259)	334 (298–373)	279 (255–307)	120%
Syrian Arab Republic	14 (12–17)	14 (11–18)	25 (23–28)	55%
Tajikistan	26 (24–27)	19 (16–23)	35 (33–36)	55%
Thailand	265 (258–272)	288 (275–302)	338 (332–345)	85%
Timor-Leste	88 (74–105)	93 (74–117)	114 (100–130)	81%
Togo	23 (21–26)	13 (10–16)	32 (28–35)	39%
Tokelau	751 (603–924)	976 (779–1210)	841 (691–1015)	116%
Tonga	196 (190–202)	152 (144–160)	245 (237–253)	62%
Tunisia	170 (163–177)	180 (169–191)	208 (200–215)	86%
Turkey	308 (288–327)	431 (393–463)	402 (383–422)	107%
Turkmenistan	129 (115–144)	158 (139–180)	221 (173–258)	72%
Tuvalu	825 (790–859)	898 (710–1120)	879 (844–914)	102%
Uganda	12 (11–13)	9 (8–11)	22 (20–23)	42%
Ukraine	186 (176–195)	97 (88–105)	232 (222–241)	42%
United Republic of Tanzania	20 (17–22)	24 (20–30)	31 (29–34)	77%
Uzbekistan	57 (54–60)	59 (52–67)	77 (73–80)	77%
Vanuatu	64 (52–76)	65 (48–87)	95 (83–107)	69%
Venezuela (Bolivarian Republic of)	49 (41–58)	102 (83–124)	82 (69–95)	125%
Viet Nam	86 (74–101)	120 (99–143)	121 (104–137)	99%
Yemen	4 (3–5)	4 (3–6)	10 (8–12)	43%
Zambia	25 (20–30)	27 (20–37)	36 (31–41)	75%
Zimbabwe	14 (11–18)	9 (6–12)	36 (32–40)	24%

All currency values are reported in 2021 inflation-adjusted US$. Discrepancies in percentages are due to rounding. Estimates in parentheses are 95% uncertainty intervals. Source data are from the Financing Global Health Database 2021. HLIP=High Level Independent Panel.

* HLIP recommended increase in government health spending between 2021 and 2026 is an additional 1% of GDP for low-income and middle-income countries.

† Calculated as the difference between government health spending change from 2021 to 2026 and HLIP projected need (additional 1% of GDP), divided by HLIP projected need (additional 1% of GDP), for each country.

## Discussion

The study showed that donor funding for pandemic preparedness as a proportion of total DAH has historically been diminutive. Nonetheless, funding raised in support of the COVID-19 health response marked the greatest increase in DAH ever observed and exceeded what was estimated to be needed for pandemic preparedness. However, should development assistance for pandemic preparedness fall back to historical levels, then available funding for pandemic preparedness at both the global and national levels will be insufficient.

There are two main conclusions from this study. First, development assistance for pandemic preparedness has not historically been prioritised. The low levels of support reflect the panic and neglect pattern that has characterised pandemic preparedness financing over the most recent decades.^29^, ^30^ A 2019 analysis detailed the dramatic increase in resources targeted towards pandemic preparedness immediately following the Ebola crisis in 2015 ($1·01 billion) and a similarly dramatic decline in the resources targeting pandemic preparedness by 2017 ($478 million), 2 years later.^31^ Although the pattern of panic and neglect towards pandemics is well documented, existing methods for quantifying the effect of pandemics and epidemics remain at an early stage. The available data suggest that the costs of previous epidemics were high and so will be the cost of the next pandemic.^32^, ^33^ The estimated global economic cost of severe acute respiratory syndrome (SARS) in 2013 was $40 billion.^34^ For the current pandemic, estimates of total financial loss between 2020 and 2024 have been as high as $13·8 trillion.^35^, ^36^ According to the HLIP, investing now can make us better prepared, reduce loss of life, and promote economic wellbeing. Existing estimates of the annual investments required for pandemic preparedness vary. Although this study relied on estimates from the HLIP, reports from other studies range from $1·9 billion annually to $130 billion over the next 2 years and $50 billion annually thereafter.^37^, ^38^ There are also reports of an annual need of about $26·4 billion at the national level and $4·7 billion at the global level.^25^ Although these estimates vary, all are small in relation to the projected costs of a pandemic. Although previous failures to fund pandemic preparedness sustainably after the immediate crisis suggest that support for funding pandemic preparedness will remain relatively low, this time can be different if global health stakeholders and national health system leaders work together to make the necessary investments sustainable.

The second conclusion is that the funds necessary for pandemic preparedness could be available. Based on the magnitude of current total health spending globally, the resources needed to ensure that countries are adequately prepared for any pandemic is a very small fraction of what is currently spent on health. Development assistance for the COVID-19 health response was $16·0 billion in 2020 and $21·8 billion in 2021, and $1049·6 million and $786·6 million was provided for pandemic preparedness. Collectively, this spending suggests that donors could potentially provide the additional $15 billion each year that has been recommended by the HLIP for pandemic preparedness in the short run. The global health community has shown, by the mobilisation of substantial additional resources for health support since the onset of the pandemic, what is possible when political commitment is present.^39^ Development assistance for health will probably be a key means for supporting pandemic preparedness and other global health goals such as the Sustainable Development Goals (SDGs) that aim to leave no one behind, assuming the existing disparities in global health spending persist.

Furthermore, the HLIP also recommends that an additional 1% of GDP be spent by each country's government on health. As indicated earlier, only 17 of the 137 LMICs will meet the recommended target by 2026. These results highlight how challenging an increase of government spending of such a magnitude would be for many countries. Increasing government spending on health by an additional 1% of the GDP would mean 120 (88%) of the 137 LMICs need to increase their spending by an average of at least 54·5% (95% UI 45·7–64·7). Although this remains a feasible task for governments, it is undoubtedly a formidable one.

This study has some limitations. First, due to lags in data availability, our current understanding of global health spending during the COVID-19 pandemic is limited. Nonetheless, with the available data, we can describe global health spending at the start of the pandemic, which still provides very useful context. Second, to obtain the most recent data on DAH support, we leverage various databases that report official development assistance. These databases have different levels of completeness, and although we cross-validated, aggregated, and supplemented sources to ensure an improved level of completeness, we acknowledge this as a limitation to the currently available public data on development assistance. This limitation is highlighted in particular in the analyses that disaggregate the available development assistance for pandemic preparedness and the proportion of funding that is reported as unallocable. Although the current analyses are insightful for understanding the distribution of the recipient-level spending data, they also highlight the paucity of the available data and the urgent need for development agencies to improve upon the reporting standards at the recipient level. Third, the keyword search approach leveraged in the generation of DAH estimates is contingent on the text provided in the project detail, which might or might not accurately reflect what project activities are undertaken in the field. Fourth, we used existing estimates of global investment requirements for pandemic preparedness from the HLIP. These existing need estimates excluded antimicrobial containment measures and thus highlight mainly a conservative estimate of the additional resources required and not a fully comprehensive estimate, suggesting that our estimates of the magnitude of the gap are also conservative. Lastly, the geographical detail was only available for a small number of projects (84 629 [4·2%] of 2 000 816), which resulted in a major portion of the funding for pandemic preparedness being categorised as unallocable and highlights the challenge with the publicly available data on development assistance for pandemic preparedness.

The years 2020 and 2021 have brought an unprecedented scale-up in DAH. The COVID-19 pandemic has raised awareness of the importance of investing in global public goods, such as in infectious diseases tracking and emerging pathogen detection systems for pandemic preparedness. Chronic underfunding of pandemic preparedness could persist into the future without proactive measures to change course. This moment presents a unique opportunity to end the cycle of panic and neglect that has long characterised pandemic preparedness financing efforts and to sustain funding for crucial global health functions.

## Data sharing

Data used for this study were extracted from publicly available sources that are listed in the appendix (pp 62–67, 159). Further details are available on the Global Health Data Exchange website (https://ghdx.healthdata.org/series/financing-global-health-fgh).

## Declaration of interests

S Bhaskar reports support for the present manuscript from leadership or fiduciary roles in board, society, committee or advocacy groups, paid or unpaid with the Rotary Club of Sydney and Global Health and Migration Hub Community, Global Health Hub Germany as a Board Director and Co-Manager. N Fullman reports funding from Gates Ventures, outside of the submitted work. D Holinelli reports grants or contracts from Ministero dell'Università e della Ricerca (Italian Ministry of University and Research, MUR), outside the submitted work. A Guha reports being the recipient of the American Heart Association Strategically Focused Research Network Grant, consulting fees from Myovant and Pfizer, payment or honoraria for lectures, presentations, speakers bureaus, manuscript writing, or educational events from the Association of Community Cancer Centers, Ohio State University, and the University of Kentucky, outside the submitted work. C Herteliu reports grants or contracts from Romanian Ministry of Research Innovation and Digitalization and MCID, outside the submitted work. J J Jozwiak reports personal fees from Novartis and Adamed, outside the submitted work. K Krishan is supported by the UGC Centre of Advanced Study (phase II), awarded to the Department of Anthropology, Panjab University, Chandigarh, India, outside the submitted work. L G Mantovani reports support from the Italian Ministry of Health for the present manuscript. L Monasta received support for the present manuscript from the Italian Ministry of Health (Ricerca Corrente 34/2017), payments made to the Institute for Maternal and Child Health IRCCS Burlo Garofolo. F Obi is an unpaid board member of Health Systems Global, outside the submitted work. J Sanabria reports a patent for a synthetic peptide active on *ATP1A1* signalosome for hepatocellular carcinoma and is a member of multiple scientific and clinical societies and associations, outside the submitted work. L R Silva supported by the project code CENTRO-04-3559-FSE-000162, Fundo Social Europeu. J A Singh reports consulting fees from Crealta/Horizon, Medisys, Fidia, PK Med, Two Labs, Adept Field Solutions, Clinical Care options, Clearview Healthcare Partners, Putnam Associates, Focus Forward, Navigant Consulting, Spherix, MedIQ, Jupiter Life Science, UBM, Trio Health, Medscape, WebMD, Practice Point Communications, the US National Institutes of Health, and the American College of Rheumatology; payment or honoraria for lectures, presentations, speakers bureaus, manuscript writing, or educational events from Simply Speaking; support for attending meetings or travel from the steering committee of OMERACT; unpaid participation on a data safety monitoring board or advisory board with the US Food and Drug Administration Arthritis Advisory Committee; leadership or fiduciary role in board, society, committee or advocacy group, paid or unpaid, with OMERACT as a steering committee member, as Chair of the Veterans Affairs Rheumatology Field Advisory Committee, and as Editor and Director of the UAB Cochrane Musculoskeletal Group Satellite Center on Network Meta-analysis; stock or stock options in TPT Global Tech, Vaxart Pharmaceuticals, Atyu Biopharma, Adaptimmune Therapeutics, GeoVax Labs, Pieris Pharmaceuticals, Enzolytics, Seres Therapeutics, Tonix Pharmaceuticals, and Charlotte's Web Holdings; and previously owning stock options in Amarin, Viking, and Moderna pharmaceuticals. L Zühlke receives support from the National Research Foundation of South Africa, as well as the UK Medical Research Council and the UK Department for International Development under the MRC/DFID Concordat agreement, via the African Research Leader Award (MR/S005242/1). All other authors declare no competing interests.
