# Supplementary material for: Global investments in pandemic preparedness and COVID-19: development assistance and domestic spending on health between 1990 and 2026
Source: Lancet Glob Health. 2023 Jan 24;11(3):e385–413. doi: 10.1016/S2214-109X(23)00007-4 (PMC9998276; doi:10.1016/S2214-109X(23)00007-4)
Supplement: Supplementary appendix [file mmc1.pdf]

THE LANCET
Global Health

Supplementary appendix

This appendix formed part of the original submission and has been peer reviewed. We post it as supplied by the authors.

Supplement to: Global Burden of Disease 2021 Health Financing Collaborator Network. Global investments in pandemic preparedness and COVID-19: development assistance and domestic spending on health between 1990 and 2026. *Lancet Glob Health* 2023; published online Jan 24. https://doi.org/10.1016/S2214-109X(23)00007-4.

SUPPLEMENTARY METHODS ANNEX

Global investments in pandemic preparedness and COVID-19: tracking development assistance and domestic spending on health, 1990-2026

Version: January 12, 2023

Global Burden of Disease 2021 Health Financing Collaborator Network

This appendix provides further methodological detail for "Global investments in pandemic preparedness and COVID-19: tracking development assistance and domestic spending on health, 1990-2050."

Portions of this appendix have been reproduced or adapted from previous Global Burden of Disease Health Financing Collaborator Network publications.[1,2] References are provided for reproduced sections.

---

1) Global Burden of Disease Health Financing Collaborator Network. **Health sector spending and spending on HIV/AIDS, tuberculosis, and malaria, and development assistance for health: progress towards Sustainable Development Goal 3.** *The Lancet.* 23 April 2020. doi:10.1016/S0140-6736(20)30608-5.

2) Global Burden of Disease 2020 Health Financing Collaborator Network. **Tracking development assistance for health and for COVID-19: a review of development assistance, government, out-of-pocket, and other private spending on health for 204 countries and territories, 1990–2050.** *The Lancet.* 22 September 2021. doi: 10.1016/S0140-6736(21)01258-7.

Table of Contents

SECTION 1. METHODS OVERVIEW

S1.1 Overview

S1.2 Estimating domestic health spending: 1995-2019

S1.3 Estimating development assistance for health: 1990-2020

S1.4 Forecasting GDP per capita in the short term to reflect the impacts of COVID-19

S1.5 Reporting and uncertainty analysis

SECTION 2. TRACKING TOTAL HEALTH SPENDING AND ITS COMPONENTS

S2.1 Overview

S2.2 Statistical model to fill missingness in health expenditure variables

S2.3 Currency exchange and deflation

SECTION 3. TRACKING DEVELOPMENT ASSISTANCE FOR HEALTH

S3.1 Overview

S3.2 Tracking development assistance for health from bilateral aid agencies and the European Commission

S3.3 Tracking development assistance for health from the development banks

S3.4 Tracking contributions from the Global Fund and Gavi

S3.5 Tracking expenditure by United Nations Agencies active in the health domain

S3.6 Tracking development assistance for health from private foundations

S3.7 Tracking non-governmental organizations

S3.8 Tracking Development Assistance for health from China

S3.9 Calculating the technical assistance and program support component of development assistance for health from loan-and grant-making channels of assistance

S3.10 Regular development assistance for health References

S3.11 Tracking Development Assistance for Health: incorporating COVID-19

SECTION 4. FORECASTING HEALTH SPENDING FROM 2020 THROUGH 2050

S4.1 Data

S4.2 Covariates

S4.2.1 List of Forecasted Variables
S4.2.2 Covariates Used for Forecasting
S4.3 Ensemble Modeling
S4.3.1 Sub-model Setup
S4.3.2 Covariates
S4.3.3 Convergence to Global Growth Rates
S4.3.4 Specifications
S4.4 Inclusion and Exclusion Criteria
S4.5 Creating the Forecasts
S4.5.1 Ranking Sub-Models
S4.5.2 Uncertainty Estimation
S4.6 Ad-hoc Draws Correlation
S6.6.1 Motivation
S6.6.2 Bivariate Correlated Distributions
S4.7 Future Health Scenarios
S6.7.1 Long-term Growth Regressions
S6.7.2 Forecasting Greater and Lesser Scenarios
S6.7.3 Uncertainty Estimation
S4.8 Package and Architecture
S6.8.1 Architecture
S6.8.2 Template Model Builder
S4.9 Tables and Figures
SECTION 5. AUTHORS' CONTRIBUTIONS STATEMENT

Abbreviations

ADB	Asian Development Bank
AfDB	African Development Bank
BMGF	Bill & Melinda Gates Foundation
CRS	Creditor Reporting System
DAC	Development Assistance Committee
DAH	Development assistance for health
EC	European Commission
GDP	Gross domestic product
Global Fund	Global Fund to fight AIDS, Tuberculosis and Malaria
GGHE	General Government health expenditure
GHED	Global Health Expenditure Database
GHES	Government health spending (Aggregate of "Social insurance contributions" and "Transfers from government domestic revenue (allocated to health purposes)", as defined in GHED)
HSS	Health System Strengthening
IBRD	International Bank for Reconstruction and Development
IDA	International Development Association
IDB	Inter-American Development Bank
LCU	Local currency units
NASA	National AIDS Spending Assessments
NGO	Non-governmental organization
NHA	National Health Account
NPISH	Non-profit institutions serving households
ODA	Official development assistance
OECD	Organisation for Economic Co-operation and Development
OOP	Out-of-pocket health spending (defined in GHED: “Other revenues from households n.e.c")
PAHO	Pan American Regional Office for WHO
PI	Private insurance
PPP	Prepaid private health spending (Aggregate of "Voluntary prepayment", "Other revenues from corporations n.e.c.", "Other revenues from NPISH n.e.c.", and "Compulsory prepayment (Other, and unspecified, than FS.3)", as defined in GHED)

SHA 2011	System of Health Accounts 2011
SWAps	Sector Wide Approaches
TB	Tuberculosis
THE	Total health spending (defined in GHED: “Current health expenditure by revenues of health care financing schemes”)
UNICEF	United Nations Children’s Fund
UNAIDS	Joint United Nations Programme on HIV/AIDS
UNFPA	United Nations Population Fund
USD	US dollars
VolAg	Report of Voluntary Agencies
WHO	World Health Organization

SECTION 1. METHODS OVERVIEW

S1.1 Overview

The methods presented here summarize the various components of the estimation process. “Health spending” is defined as money spent on services, supplies, and basic infrastructure to deliver health care, using the same definition employed by the System of Health Accounts 2011 and the WHO Global Health Expenditure Database (GHED).1,2 Health “financing” and “funding” are used interchangeably to refer to the source, as opposed to the utilization, of financial resources. “Economic development” refers to GDP per capita.

We estimated health spending from four main sources – government, out-of-pocket, prepaid private, and development assistance for health (DAH) – for 204 countries and territories shown in TS1.1.. For brevity, “countries and territories” are referred to only as “countries”, all of which are categorized into four World Bank income groups (high-, upper-middle-, lower-middle-, and low-income) and seven Global Burden of Disease (GBD) super-regions (Central Europe, Eastern Europe, and Central Asia; GBD high income; Latin America and Caribbean; North Arica and Middle East; South Asia; Southeast Asia, East Asia, and Oceania; and sub-Saharan Africa). Data tracking government, out-of-pocket, and prepaid private health spending, which together comprise total domestic health spending, were available from 1995 through 2019 Government health spending includes social health insurance and mandated private health insurance, as well as government public health programmes. Out-of-pocket health spending includes health care spending paid by the patient or his or her household, excluding insurance premiums paid in advance of care. Prepaid private health spending includes voluntary private insurance and non-governmental agency spending on health.

Estimates of DAH, defined as the financial and in-kind contributions from major development agencies to low- and middle-income countries for the purpose of maintaining or improving population health, were generated from 1990 through 2021, although transfers to recipient countries can only be estimated until 2020. In other words, the total amount of DAH, by source, is estimated through 2021, but is not allocated by recipient country for 2021. The sum of domestic health spending and DAH, net of the administrative costs needed to run the development agencies, form the envelope of total health spending for each country and year.

Domestic health spending from each of the three sources was projected for each country from 2020 to 2050, and DAH was projected from 2021 to 2050, by modelling rates of change across time. These models incorporate country-specific time trends that attenuate across time and converge to the global average, consider a broad set of covariates and time-series modelling techniques, and propagate four types of uncertainty - model, data, parameter, and fundamental uncertainty.

S1.1 Health spending by source per person and percent change for 2010, 2018, and 2019

	2019 value per person (2021 US dollars)	2018 value per person (2021 US dollars)	Percent increase between 2018 and 2019	2010 value per person (2021 US dollars)	Percent increase between 2010 and 2019

Total Health spending	1183 (1171 to 1195)	1160 (1151 to 1168)	2·0% (0·8% to 3·3%)	1004 (997 to 1012)	17·8% (16·4% to 19·2%)
Government health spending	708 (700 to 715)	693 (687 to 698)	2·2% (0·9% to 3·4%)	591 (587 to 596)	19·8% (18·2% to 21·4%)
Prepaid private spending	254 (247 to 262)	249 (244 to 255)	2·1% (-1·6% to 5·9%)	220 (215 to 225)	15·6% (11·2% to 20·0%)
Out-of-pocket spending	215 (210 to 221)	212 (208 to 216)	1·6% (-1·7% to 5·2%)	188 (184 to 192)	14·5% (11·1% to 18·2%)
Development assistance for health	6 (6 to 6)	6 (6 to 6)	-1·1% (-1·1% to -1·1%)	5 (5 to 5)	2·5% (2·5% to 2·5%)

S1.2 Estimates of government, out-of-pocket, prepaid private, and development assistance for health, 2019 and 2026

	Health spending per person, 2019 and 2026 (2021 US dollars)		Health spending per person, 2019 and 2026 (2021 Purchasing-power parity-adjusted dollars)		Total health spending per gross domestic product, 2019 and 2026		Government health spending per person (2021 US dollars)	Prepaid private spending per person (2021 US dollars)	Out-of-pocket spending per person (2021 US dollars)	Development assistance for health per person (2021 US dollars)
	2019	2026	2019	2026	2019	2026	2019	2019	2019	2019
Global										
Total	1183 (1171 to 1195)	1366 (1345 to 1387)	1518 (1505 to 1531)	1769 (1744 to 1796)	9·7% (9·6 to 9·8)	10·2% (10·0 to 10·4)	708 (700 to 715)	254 (247 to 262)	215 (210 to 221)	6 (6 to 6)
World Bank income groups										
High-income	5938 (5876 to 6004)	6978 (6863 to 7101)	6469 (6405 to 6535)	7600 (7483 to 7725)	12·4% (12·2 to 12·5)	13·4% (13·1 to 13·6)	3682 (3645 to 3717)	1441 (1395 to 1485)	815 (785 to 851)	-
Upper-middle-income	575 (561 to 590)	773 (743 to 807)	1085 (1062 to 1108)	1426 (1378 to 1480)	5·7% (5·6 to 5·9)	6·2% (5·9 to 6·5)	322 (309 to 336)	65 (59 to 70)	187 (180 to 194)	1 (1 to 1)
Lower-middle-income	117 (114 to 121)	140 (136 to 145)	300 (291 to 309)	362 (349 to 375)	4·1% (3·9 to 4·2)	4·1% (4·0 to 4·3)	47 (45 to 48)	12 (11 to 14)	55 (53 to 58)	3 (3 to 3)
Low-income	37 (36 to 38)	41 (40 to 44)	145 (140 to 150)	153 (146 to 160)	4·9% (4·7 to 5·2)	5·0% (4·7 to 5·3)	9 (8 to 9)	2 (2 to 2)	16 (16 to 17)	11 (11 to 11)
GBD super-region										
Central Europe, Eastern Europe, and Central Asia	649 (642 to 657)	713 (700 to 727)	1514 (1496 to 1534)	1644 (1612 to 1680)	6·1% (6·0 to 6·2)	6·3% (6·2 to 6·5)	420 (413 to 426)	24 (23 to 26)	204 (200 to 207)	1 (1 to 1)
GBD high-income	6531 (6463 to 6606)	7649 (7515 to 7789)	6971 (6902 to 7047)	8145 (8008 to 8285)	12·9% (12·7 to 13·0)	14·0% (13·7 to 14·2)	4025 (3985 to 4066)	1614 (1563 to 1664)	892 (859 to 932)	-

Latin America and Caribbean	563 (547 to 580)	626 (606 to 647)	1198 (1164 to 1234)	1342 (1300 to 1387)	7·2% (7·0 to 7·4)	7·7% (7·5 to 8·0)	289 (277 to 300)	110 (101 to 118)	163 (156 to 170)	2 (2 to 2)
North Africa and Middle East	448 (437 to 460)	524 (508 to 540)	963 (944 to 983)	1130 (1098 to 1163)	5·5% (5·3 to 5·6)	5·9% (5·7 to 6·2)	253 (245 to 261)	55 (51 to 59)	138 (132 to 145)	2 (2 to 2)
South Asia	64 (59 to 69)	85 (79 to 91)	206 (192 to 221)	273 (253 to 292)	3·0% (2·8 to 3·2)	3·1% (2·8 to 3·3)	20 (18 to 22)	7 (5 to 9)	36 (33 to 40)	1 (1 to 1)
Southeast Asia, East Asia, and Oceania	460 (445 to 478)	674 (640 to 714)	791 (767 to 819)	1147 (1094 to 1210)	5·0% (4·9 to 5·3)	5·6% (5·3 to 6·0)	259 (245 to 274)	41 (35 to 47)	160 (152 to 168)	1 (1 to 1)
Sub-Saharan Africa	82 (80 to 83)	86 (83 to 89)	193 (188 to 197)	205 (198 to 212)	4·8% (4·6 to 5·0)	4·9% (4·7 to 5·1)	33 (32 to 34)	15 (14 to 16)	23 (22 to 24)	10 (10 to 10)
Country										
Central Europe, Eastern Europe, and Central Asia										
Albania (UM)	339 (317 to 363)	423 (392 to 458)	851 (796 to 911)	1063 (984 to 1149)	5·6% (5·2 to 6·0)	5·6% (5·2 to 6·1)	184 (163 to 205)	0 (0 to 0)	152 (142 to 162)	4 (4 to 4)
Armenia (UM)	514 (483 to 548)	596 (560 to 635)	1602 (1508 to 1708)	1858 (1747 to 1980)	10·5% (9·5 to 11·3)	10·5% (9·7 to 11·2)	64 (61 to 68)	9 (6 to 12)	434 (405 to 466)	7 (7 to 7)
Azerbaijan (UM)	206 (188 to 224)	217 (197 to 237)	605 (553 to 658)	637 (581 to 696)	3·8% (3·5 to 4·2)	3·8% (3·4 to 4·1)	61 (56 to 67)	0 (0 to 0)	144 (127 to 160)	1 (1 to 1)
Belarus (UM)	413 (386 to 443)	443 (411 to 478)	1227 (1146 to 1317)	1316 (1221 to 1420)	5·8% (5·4 to 6·2)	6·1% (5·6 to 6·6)	288 (267 to 311)	15 (11 to 20)	107 (95 to 122)	2 (2 to 2)
Bosnia and Herzegovina (UM)	579 (554 to 606)	757 (721 to 795)	1439 (1378 to 1508)	1881 (1792 to 1978)	8·9% (8·4 to 9·5)	9·7% (9·0 to 10·5)	404 (384 to 424)	2 (1 to 3)	171 (155 to 186)	1 (1 to 1)
Bulgaria (UM)	840 (806 to 871)	1009 (943 to 1070)	1862 (1788 to 1932)	2238 (2091 to 2373)	7·3% (7·0 to 7·6)	7·2% (6·7 to 7·7)	492 (468 to 517)	16 (10 to 23)	332 (312 to 351)	0 (0 to 0)
Croatia (H)	1072 (1035 to 1111)	1350 (1247 to 1462)	2100 (2027 to 2176)	2644 (2443 to 2864)	6·6% (6·3 to 6·8)	6·9% (6·3 to 7·5)	882 (845 to 919)	72 (63 to 82)	118 (107 to 130)	-
Czechia (H)	2117 (2061 to 2172)	2414 (2331 to 2498)	3513 (3421 to 3605)	4008 (3869 to 4146)	7·8% (7·6 to 8·0)	8·0% (7·7 to 8·3)	1732 (1681 to 1784)	85 (77 to 95)	300 (287 to 314)	-
Estonia (H)	1793 (1767 to 1820)	2210 (2108 to 2323)	2764 (2724 to 2806)	3407 (3250 to 3581)	6·9% (6·8 to 7·0)	7·1% (6·8 to 7·5)	1330 (1311 to 1348)	28 (25 to 32)	435 (417 to 452)	-
Georgia (UM)	352 (334 to 374)	459 (426 to 501)	1186 (1124 to 1259)	1548 (1434 to 1688)	7·4% (6·8 to 8·1)	7·4% (6·7 to 8·4)	141 (127 to 157)	37 (28 to 50)	158 (151 to 164)	16 (16 to 16)
Hungary (H)	1194 (1167 to 1222)	1464 (1407 to 1523)	2337 (2285 to 2392)	2866 (2755 to 2981)	6·4% (6·3 to 6·6)	6·5% (6·2 to 6·8)	818 (794 to 842)	47 (43 to 51)	329 (316 to 342)	-

Kazakhstan (UM)	296 (276 to 317)	329 (303 to 357)	842 (787 to 903)	936 (861 to 1017)	2·9% (2·7 to 3·1)	2·9% (2·7 to 3·2)	177 (160 to 194)	17 (11 to 24)	101 (91 to 112)	1 (1 to 1)
Kyrgyzstan (LM)	73 (64 to 82)	73 (64 to 83)	302 (265 to 338)	301 (265 to 341)	5·2% (4·6 to 5·8)	5·4% (4·7 to 6·1)	33 (27 to 39)	0 (0 to 0)	34 (28 to 42)	6 (6 to 6)
Latvia (H)	1300 (1260 to 1339)	1603 (1523 to 1682)	2193 (2126 to 2259)	2704 (2568 to 2837)	6·5% (6·3 to 6·7)	6·7% (6·2 to 7·2)	785 (755 to 813)	29 (22 to 38)	486 (459 to 510)	-
Lithuania (H)	1541 (1507 to 1572)	1924 (1852 to 1994)	2818 (2756 to 2876)	3518 (3387 to 3647)	6·9% (6·7 to 7·0)	7·1% (6·6 to 7·6)	1010 (980 to 1036)	35 (32 to 38)	495 (479 to 512)	-
Moldova (UM)	234 (217 to 254)	316 (290 to 344)	683 (631 to 742)	922 (845 to 1004)	5·6% (4·2 to 8·3)	5·7% (4·3 to 8·2)	137 (122 to 154)	3 (2 to 4)	91 (81 to 100)	4 (4 to 4)
Mongolia (LM)	195 (186 to 204)	239 (225 to 255)	553 (528 to 578)	677 (637 to 724)	4·1% (3·9 to 4·3)	4·5% (4·1 to 4·8)	108 (101 to 115)	6 (4 to 9)	62 (58 to 67)	19 (19 to 19)
Montenegro (UM)	811 (767 to 856)	934 (878 to 997)	1957 (1852 to 2067)	2255 (2120 to 2407)	8·3% (7·8 to 8·8)	8·6% (8·1 to 9·2)	483 (447 to 520)	7 (4 to 10)	318 (296 to 344)	2 (2 to 2)
North Macedonia (UM)	555 (531 to 579)	639 (609 to 667)	1487 (1423 to 1550)	1712 (1631 to 1788)	8·1% (7·8 to 8·5)	8·2% (7·7 to 8·6)	322 (301 to 346)	3 (2 to 5)	229 (223 to 234)	1 (1 to 1)
Poland (H)	1096 (1075 to 1116)	1400 (1351 to 1452)	2324 (2279 to 2367)	2969 (2864 to 3079)	6·4% (6·2 to 6·5)	6·7% (6·4 to 7·0)	783 (763 to 799)	89 (84 to 93)	225 (216 to 233)	-
Romania (H)	830 (807 to 852)	1040 (978 to 1101)	1908 (1856 to 1958)	2391 (2248 to 2531)	5·8% (5·6 to 5·9)	5·9% (5·5 to 6·4)	666 (645 to 687)	8 (7 to 10)	156 (152 to 162)	-
Russia (UM)	668 (650 to 688)	673 (639 to 712)	1690 (1644 to 1740)	1702 (1616 to 1800)	5·6% (5·4 to 5·8)	5·9% (5·6 to 6·3)	406 (389 to 422)	16 (11 to 21)	247 (238 to 256)	0 (0 to 0)
Serbia (UM)	560 (538 to 583)	742 (706 to 777)	1321 (1267 to 1374)	1750 (1664 to 1831)	6·6% (6·3 to 6·9)	6·8% (6·4 to 7·1)	331 (311 to 352)	12 (9 to 18)	212 (202 to 221)	5 (5 to 5)
Slovakia (H)	1482 (1432 to 1532)	1780 (1704 to 1860)	2494 (2410 to 2579)	2995 (2868 to 3130)	6·9% (6·7 to 7·2)	7·3% (6·9 to 7·7)	1175 (1131 to 1220)	31 (24 to 41)	276 (249 to 304)	-
Slovenia (H)	2434 (2373 to 2497)	2955 (2857 to 3050)	3672 (3580 to 3768)	4459 (4311 to 4602)	8·5% (8·3 to 8·7)	8·9% (8·6 to 9·3)	1761 (1704 to 1823)	388 (372 to 405)	285 (275 to 295)	-
Tajikistan (LM)	56 (51 to 60)	64 (59 to 70)	274 (252 to 298)	318 (289 to 347)	6·8% (6·1 to 7·5)	6·8% (6·0 to 7·5)	15 (14 to 16)	0 (0 to 0)	36 (31 to 41)	5 (5 to 5)
Turkmenistan (UM)	714 (669 to 762)	744 (697 to 793)	1227 (1150 to 1310)	1279 (1198 to 1364)	8·3% (5·7 to 13·6)	8·2% (5·8 to 13·0)	133 (119 to 147)	37 (28 to 47)	543 (504 to 587)	1 (1 to 1)
Ukraine (LM)	326 (317 to 336)	221 (211 to 231)	969 (940 to 997)	657 (626 to 686)	7·1% (6·7 to 7·5)	7·2% (6·2 to 8·3)	149 (141 to 157)	9 (6 to 13)	166 (161 to 170)	3 (3 to 3)
Uzbekistan (LM)	108 (104 to 112)	134 (126 to 142)	461 (445 to 480)	575 (540 to 608)	5·7% (5·5 to 6·1)	5·9% (5·5 to 6·4)	43 (41 to 45)	1 (0 to 1)	61 (58 to 65)	3 (3 to 3)

GBD high-income										
Andorra (H)	2805 (2669 to 2943)	2906 (2741 to 3081)	3913 (3723 to 4106)	4053 (3823 to 4298)	6·3% (6·0 to 6·6)	6·6% (6·2 to 7·1)	1931 (1796 to 2067)	502 (466 to 540)	372 (352 to 393)	-
Argentina (UM)	1038 (999 to 1079)	1145 (1075 to 1219)	2299 (2211 to 2388)	2535 (2380 to 2699)	9·4% (9·1 to 9·8)	9·8% (9·2 to 10·5)	644 (613 to 676)	106 (88 to 127)	286 (271 to 302)	2 (2 to 2)
Australia (H)	6421 (6293 to 6527)	7476 (7256 to 7701)	5701 (5588 to 5796)	6638 (6443 to 6838)	10·5% (10·0 to 11·3)	11·3% (10·5 to 12·2)	4566 (4480 to 4646)	810 (753 to 870)	1044 (1008 to 1081)	-
Austria (H)	5741 (5672 to 5817)	6369 (6144 to 6615)	6425 (6348 to 6510)	7128 (6876 to 7402)	10·4% (10·3 to 10·6)	11·0% (10·6 to 11·5)	4188 (4126 to 4250)	451 (430 to 474)	1102 (1074 to 1132)	-
Belgium (H)	5581 (5481 to 5695)	6205 (6015 to 6421)	6140 (6030 to 6265)	6827 (6617 to 7064)	10·7% (10·5 to 10·9)	11·3% (10·9 to 11·7)	4288 (4191 to 4394)	280 (268 to 293)	1012 (983 to 1039)	-
Brunei (H)	1010 (933 to 1084)	1136 (980 to 1311)	1506 (1392 to 1617)	1694 (1461 to 1955)	2·2% (2·1 to 2·4)	2·2% (1·9 to 2·6)	956 (880 to 1032)	0 (0 to 0)	54 (45 to 65)	-
Canada (H)	5968 (5893 to 6040)	6445 (6263 to 6623)	6073 (5998 to 6147)	6560 (6374 to 6740)	11·2% (11·0 to 11·3)	11·6% (11·3 to 12·0)	4188 (4129 to 4247)	890 (853 to 929)	890 (868 to 916)	-
Chile (H)	1545 (1516 to 1573)	1850 (1800 to 1900)	2568 (2521 to 2614)	3076 (2992 to 3158)	9·7% (9·5 to 9·9)	10·6% (9·9 to 11·4)	788 (769 to 808)	250 (239 to 263)	506 (492 to 520)	-
Cyprus (H)	1426 (1364 to 1492)	1658 (1575 to 1740)	2080 (1991 to 2177)	2419 (2298 to 2539)	5·1% (4·2 to 6·7)	5·5% (4·6 to 7·1)	719 (676 to 767)	188 (173 to 204)	519 (480 to 563)	-
Denmark (H)	6708 (6581 to 6831)	7702 (7406 to 8029)	6330 (6210 to 6447)	7269 (6989 to 7577)	10·0% (9·8 to 10·2)	10·5% (10·1 to 11·0)	5592 (5470 to 5718)	169 (162 to 175)	946 (925 to 966)	-
Finland (H)	4906 (4828 to 4985)	5555 (5393 to 5730)	4883 (4805 to 4961)	5529 (5367 to 5703)	9·2% (9·0 to 9·3)	9·6% (9·2 to 9·9)	3918 (3848 to 3988)	127 (119 to 136)	862 (837 to 887)	-
France (H)	4976 (4903 to 5047)	5528 (5392 to 5657)	5700 (5616 to 5781)	6332 (6177 to 6480)	11·1% (10·7 to 11·4)	11·7% (11·3 to 12·1)	3761 (3709 to 3810)	752 (703 to 805)	463 (443 to 485)	-
Germany (H)	5887 (5829 to 5941)	6628 (6471 to 6788)	6767 (6701 to 6830)	7619 (7439 to 7804)	11·4% (11·3 to 11·5)	12·0% (11·7 to 12·3)	4575 (4522 to 4630)	558 (548 to 568)	754 (737 to 770)	-
Greece (H)	1670 (1591 to 1761)	1913 (1821 to 2023)	2657 (2532 to 2802)	3044 (2898 to 3220)	8·1% (7·7 to 8·5)	8·2% (7·7 to 8·8)	828 (765 to 894)	247 (223 to 272)	595 (557 to 635)	-
Greenland (H)	6700 (5922 to 7459)	8853 (7862 to 9868)	6013 (5315 to 6694)	7945 (7056 to 8856)	11·1% (9·8 to 12·5)	12·2% (10·6 to 13·9)	6699 (5922 to 7458)	0 (0 to 1)	0 (0 to 1)	-
Iceland (H)	6528 (6297 to 6797)	7301 (6734 to 7921)	5654 (5454 to 5887)	6324 (5833 to 6860)	8·8% (8·2 to 9·3)	9·5% (8·4 to 10·6)	5403 (5179 to 5673)	109 (104 to 113)	1016 (971 to 1059)	-
Ireland (H)	5733 (5511 to 5968)	8063 (7662 to 8451)	6510 (6259 to 6777)	9157 (8702 to 9598)	6·8% (6·5 to 7·1)	7·2% (6·8 to 7·7)	4266 (4063 to 4486)	791 (730 to 862)	675 (630 to 724)	-

Israel (H)	3565 (3500 to 3629)	4166 (4043 to 4293)	3172 (3114 to 3230)	3707 (3598 to 3820)	7·0% (6·5 to 7·3)	7·2% (6·8 to 7·6)	2356 (2308 to 2406)	441 (421 to 461)	768 (734 to 801)	-
Italy (H)	3114 (3052 to 3177)	3442 (3322 to 3561)	4054 (3973 to 4136)	4481 (4325 to 4636)	8·6% (8·5 to 8·9)	9·0% (8·6 to 9·3)	2301 (2241 to 2360)	86 (82 to 90)	727 (707 to 748)	-
Japan (H)	4317 (4214 to 4425)	4772 (4621 to 4936)	4909 (4792 to 5033)	5427 (5255 to 5613)	10·7% (10·4 to 11·0)	11·2% (10·7 to 11·6)	3620 (3515 to 3729)	140 (136 to 145)	557 (544 to 569)	-
Luxembourg (H)	7114 (6738 to 7505)	7865 (7299 to 8429)	6862 (6499 to 7239)	7586 (7040 to 8130)	5·3% (5·0 to 5·6)	5·5% (5·1 to 5·9)	6164 (5809 to 6543)	230 (199 to 269)	720 (634 to 814)	-
Malta (H)	3169 (3075 to 3257)	3520 (3339 to 3694)	4715 (4575 to 4845)	5237 (4968 to 5495)	8·9% (7·9 to 9·6)	8·9% (8·0 to 10·0)	2010 (1941 to 2087)	71 (66 to 77)	1088 (1038 to 1139)	-
Monaco (H)	3696 (3490 to 3910)	3975 (3715 to 4258)	3821 (3607 to 4042)	4109 (3840 to 4402)	1·7% (1·5 to 2·0)	1·8% (1·6 to 2·1)	3156 (2951 to 3362)	261 (210 to 317)	279 (265 to 294)	-
Netherlands (H)	5956 (5831 to 6095)	6786 (6496 to 7092)	6419 (6284 to 6568)	7314 (7001 to 7643)	10·2% (9·9 to 10·5)	10·8% (10·3 to 11·3)	3917 (3818 to 4026)	1408 (1334 to 1481)	632 (594 to 666)	-
New Zealand (H)	4615 (4526 to 4711)	5269 (5110 to 5432)	4441 (4355 to 4532)	5069 (4916 to 5226)	9·4% (8·8 to 9·8)	10·0% (9·3 to 10·7)	3481 (3399 to 3567)	561 (532 to 589)	572 (544 to 602)	-
Norway (H)	9180 (8983 to 9407)	10650 (10210 to 11145)	7298 (7141 to 7478)	8467 (8117 to 8860)	10·5% (10·3 to 10·8)	11·2% (10·6 to 11·8)	7880 (7678 to 8106)	30 (26 to 34)	1270 (1225 to 1314)	-
Portugal (H)	2317 (2249 to 2389)	2578 (2439 to 2740)	3521 (3418 to 3630)	3917 (3707 to 4163)	9·1% (8·9 to 9·4)	9·4% (8·8 to 10·0)	1411 (1351 to 1469)	204 (176 to 235)	703 (677 to 730)	-
San Marino (H)	3462 (3331 to 3591)	3766 (3622 to 3922)	4478 (4309 to 4645)	4872 (4686 to 5073)	6·7% (6·5 to 7·0)	7·1% (6·8 to 7·4)	2837 (2710 to 2961)	37 (34 to 39)	589 (553 to 625)	-
Singapore (H)	2795 (2708 to 2877)	3796 (3484 to 4093)	4470 (4331 to 4601)	6071 (5573 to 6546)	4·2% (4·0 to 4·3)	4·7% (4·2 to 5·4)	1414 (1342 to 1483)	548 (514 to 585)	833 (798 to 875)	-
South Korea (H)	2674 (2632 to 2714)	3671 (3545 to 3788)	3732 (3673 to 3789)	5125 (4948 to 5287)	7·9% (7·8 to 8·0)	9·3% (8·9 to 9·7)	1590 (1556 to 1624)	268 (259 to 277)	815 (800 to 831)	-
Spain (H)	3019 (2957 to 3082)	3337 (3238 to 3439)	4195 (4109 to 4283)	4637 (4499 to 4779)	9·3% (9·1 to 9·5)	9·9% (9·5 to 10·3)	2129 (2071 to 2193)	229 (214 to 243)	660 (644 to 676)	-
Sweden (H)	6553 (6335 to 6778)	7328 (6984 to 7681)	6436 (6222 to 6657)	7198 (6860 to 7544)	10·9% (10·4 to 11·3)	11·4% (10·7 to 12·0)	5563 (5342 to 5788)	82 (78 to 85)	909 (878 to 939)	-
Switzerland (H)	10407 (10241 to 10573)	11894 (11537 to 12248)	8717 (8578 to 8856)	9962 (9664 to 10259)	11·1% (10·9 to 11·3)	12·2% (11·8 to 12·5)	3319 (3251 to 3393)	4378 (4251 to 4492)	2710 (2611 to 2808)	-
UK (H)	4892 (4834 to 4951)	5349 (5207 to 5484)	5221 (5159 to 5285)	5709 (5557 to 5854)	10·0% (9·9 to 10·2)	10·5% (10·2 to 10·8)	3888 (3837 to 3940)	170 (163 to 179)	833 (799 to 868)	-

USA (H)	11583 (11373 to 11828)	13821 (13398 to 14300)	11583 (11373 to 11828)	13821 (13398 to 14300)	16·9% (16·6 to 17·3)	18·6% (17·9 to 19·3)	5887 (5769 to 6009)	4386 (4218 to 4553)	1310 (1201 to 1449)	-
Uruguay (H)	1547 (1529 to 1566)	1764 (1665 to 1870)	2238 (2211 to 2265)	2551 (2408 to 2704)	9·1% (8·7 to 9·8)	9·6% (8·8 to 10·5)	1111 (1096 to 1126)	178 (170 to 187)	259 (252 to 264)	-
Latin America and Caribbean										
Antigua and Barbuda (H)	885 (844 to 926)	880 (825 to 931)	1182 (1127 to 1238)	1176 (1103 to 1244)	4·9% (4·7 to 5·1)	5·1% (4·8 to 5·4)	522 (484 to 561)	147 (141 to 153)	216 (194 to 238)	-
Barbados (H)	1161 (1105 to 1217)	1197 (1135 to 1259)	1017 (967 to 1065)	1047 (994 to 1102)	6·0% (5·7 to 6·3)	5·9% (5·4 to 6·3)	520 (474 to 568)	90 (85 to 95)	552 (527 to 578)	-
Belize (UM)	284 (267 to 301)	273 (248 to 298)	443 (417 to 469)	426 (386 to 465)	5·7% (5·3 to 6·2)	6·0% (5·3 to 6·7)	195 (178 to 211)	15 (13 to 18)	62 (59 to 65)	12 (12 to 12)
Bermuda (H)	8602 (7363 to 9879)	10906 (9269 to 12638)	5956 (5098 to 6840)	7551 (6418 to 8751)	6·7% (5·2 to 9·2)	7·7% (5·6 to 10·8)	2427 (2072 to 2795)	5312 (4155 to 6563)	862 (672 to 1090)	-
Bolivia (LM)	249 (240 to 257)	269 (254 to 283)	668 (646 to 691)	722 (684 to 761)	7·0% (6·7 to 7·2)	7·3% (6·9 to 7·8)	174 (168 to 180)	10 (6 to 14)	57 (52 to 61)	8 (8 to 8)
Brazil (UM)	683 (646 to 720)	778 (733 to 823)	1460 (1380 to 1538)	1662 (1567 to 1758)	9·0% (8·5 to 9·5)	9·8% (9·2 to 10·4)	289 (262 to 317)	222 (201 to 243)	172 (158 to 186)	1 (1 to 1)
Colombia (UM)	490 (470 to 512)	629 (593 to 668)	1305 (1251 to 1363)	1675 (1580 to 1778)	8·0% (7·7 to 8·4)	8·9% (8·4 to 9·5)	352 (334 to 374)	65 (59 to 70)	73 (66 to 81)	0 (0 to 0)
Costa Rica (UM)	994 (958 to 1038)	1272 (1217 to 1339)	1805 (1738 to 1884)	2308 (2210 to 2430)	8·1% (7·8 to 8·4)	9·3% (8·8 to 9·8)	715 (678 to 749)	52 (40 to 66)	214 (196 to 232)	14 (14 to 14)
Cuba (UM)	1357 (1272 to 1440)	1513 (1409 to 1616)	2948 (2763 to 3127)	3287 (3060 to 3509)	12·0% (10·9 to 13·2)	13·2% (12·0 to 14·7)	1211 (1128 to 1294)	0 (0 to 0)	145 (138 to 152)	1 (1 to 1)
Dominica (UM)	467 (444 to 491)	517 (481 to 558)	780 (741 to 819)	862 (802 to 931)	5·5% (5·3 to 5·8)	5·7% (5·2 to 6·2)	285 (266 to 302)	6 (4 to 8)	149 (134 to 166)	28 (28 to 28)
Dominican Republic (UM)	492 (464 to 520)	636 (584 to 689)	1181 (1115 to 1250)	1528 (1403 to 1655)	5·8% (5·4 to 6·2)	6·1% (5·5 to 6·7)	221 (204 to 239)	52 (39 to 67)	215 (196 to 234)	3 (3 to 3)
Ecuador (UM)	492 (461 to 524)	527 (481 to 571)	967 (905 to 1029)	1035 (945 to 1122)	7·7% (7·2 to 8·2)	8·2% (7·6 to 8·9)	305 (283 to 328)	34 (24 to 46)	152 (136 to 172)	1 (1 to 1)
El Salvador (LM)	311 (294 to 329)	356 (334 to 380)	695 (659 to 736)	798 (748 to 850)	7·2% (6·8 to 7·6)	7·6% (7·1 to 8·0)	196 (187 to 205)	23 (16 to 31)	89 (77 to 102)	3 (3 to 3)
Grenada (UM)	604 (573 to 634)	621 (587 to 653)	1024 (971 to 1075)	1052 (995 to 1108)	5·5% (5·2 to 5·8)	5·5% (5·2 to 5·8)	230 (209 to 254)	23 (18 to 28)	312 (288 to 333)	39 (39 to 39)
Guatemala (UM)	323 (313 to 334)	366 (349 to 383)	633 (613 to 654)	717 (684 to 751)	7·0% (6·5 to 7·4)	7·2% (6·7 to 7·7)	122 (116 to 126)	18 (12 to 25)	181 (175 to 188)	3 (3 to 3)
Guyana (UM)	285 (276 to 295)	1063 (982 to 1155)	724 (701 to 751)	2703 (2498	4·7% (4·3 to 5·3)	4·8% (3·8 to 5·8)	170 (166 to 175)	9 (6 to 13)	95 (88 to 102)	11 (11 to 11)

				to 2936)						
Haiti (LM)	60 (56 to 64)	60 (56 to 65)	102 (96 to 110)	103 (96 to 112)	3·1% (2·9 to 3·3)	3·3% (3·0 to 3·6)	9 (7 to 11)	4 (3 to 6)	35 (31 to 38)	12 (12 to 12)
Honduras (LM)	199 (182 to 217)	220 (200 to 241)	443 (406 to 484)	491 (446 to 537)	7·0% (6·5 to 7·7)	7·2% (6·5 to 7·9)	79 (72 to 88)	12 (8 to 16)	106 (93 to 121)	2 (2 to 2)
Jamaica (UM)	356 (339 to 373)	403 (364 to 439)	699 (665 to 733)	791 (715 to 861)	6·3% (5·8 to 6·8)	6·9% (6·2 to 7·6)	232 (217 to 248)	59 (56 to 61)	58 (52 to 65)	8 (8 to 8)
Mexico (UM)	580 (549 to 617)	594 (556 to 638)	1194 (1130 to 1270)	1224 (1146 to 1313)	5·5% (5·2 to 5·8)	5·6% (5·2 to 6·0)	288 (266 to 308)	49 (38 to 61)	243 (223 to 265)	0 (0 to 0)
Nicaragua (LM)	179 (175 to 183)	212 (203 to 222)	532 (521 to 544)	631 (602 to 659)	8·9% (8·7 to 9·1)	9·3% (8·7 to 9·8)	105 (102 to 109)	3 (3 to 4)	58 (57 to 60)	12 (12 to 12)
Panama (H)	1208 (1178 to 1236)	1393 (1323 to 1467)	2638 (2573 to 2699)	3042 (2888 to 3203)	7·6% (7·4 to 7·8)	8·1% (7·5 to 8·5)	793 (763 to 821)	77 (73 to 81)	338 (333 to 343)	-
Paraguay (UM)	365 (341 to 394)	457 (424 to 495)	961 (898 to 1038)	1204 (1119 to 1304)	7·0% (6·5 to 7·5)	7·8% (7·2 to 8·5)	168 (151 to 185)	43 (32 to 57)	152 (137 to 169)	2 (2 to 2)
Peru (UM)	333 (309 to 357)	376 (346 to 406)	696 (647 to 745)	785 (723 to 849)	4·9% (4·5 to 5·3)	5·0% (4·6 to 5·5)	208 (192 to 226)	30 (22 to 41)	94 (82 to 109)	1 (1 to 1)
Puerto Rico (H)	1199 (1055 to 1370)	1388 (1217 to 1584)	1332 (1172 to 1522)	1542 (1352 to 1760)	3·4% (2·9 to 4·0)	3·7% (3·1 to 4·3)	894 (768 to 1043)	48 (32 to 70)	258 (198 to 334)	-
Saint Kitts and Nevis (H)	1005 (957 to 1053)	998 (937 to 1058)	1457 (1387 to 1526)	1447 (1358 to 1533)	4·9% (4·4 to 5·4)	4·9% (4·5 to 5·4)	481 (453 to 511)	48 (42 to 53)	477 (438 to 518)	-
Saint Lucia (UM)	538 (518 to 560)	561 (523 to 598)	787 (757 to 819)	820 (764 to 874)	4·7% (4·5 to 4·9)	4·9% (4·6 to 5·2)	244 (238 to 250)	28 (26 to 30)	250 (231 to 270)	16 (16 to 16)
Saint Vincent and the Grenadines (UM)	374 (349 to 397)	456 (421 to 489)	661 (617 to 701)	804 (744 to 864)	4·6% (4·2 to 5·2)	5·0% (4·5 to 5·5)	236 (216 to 255)	11 (7 to 16)	102 (88 to 116)	26 (26 to 26)
Suriname (UM)	517 (487 to 550)	464 (427 to 504)	1787 (1683 to 1902)	1603 (1475 to 1741)	8·8% (8·1 to 9·7)	9·2% (8·3 to 10·3)	364 (336 to 396)	57 (48 to 68)	91 (84 to 100)	5 (5 to 5)
The Bahamas (H)	1940 (1882 to 2003)	2127 (2037 to 2228)	2452 (2380 to 2532)	2689 (2575 to 2817)	6·1% (5·8 to 6·3)	6·8% (6·4 to 7·2)	1020 (968 to 1072)	412 (387 to 437)	507 (492 to 523)	-
Trinidad and Tobago (H)	1152 (1098 to 1207)	1193 (1118 to 1271)	2031 (1937 to 2128)	2103 (1972 to 2241)	6·9% (6·5 to 7·2)	7·0% (6·5 to 7·5)	550 (513 to 586)	79 (73 to 86)	522 (483 to 561)	-
Venezuela (UM)	243 (220 to 270)	196 (171 to 222)	368 (333 to 409)	297 (259 to 337)	5·2% (4·0 to 6·9)	5·8% (4·6 to 7·3)	117 (102 to 137)	70 (56 to 87)	56 (46 to 66)	0 (0 to 0)
Virgin Islands (H)	1390 (1175 to 1658)	1585 (1329 to 1914)	1390 (1175 to 1658)	1585 (1329 to 1914)	3·4% (2·8 to 4·1)	3·7% (3·0 to 4·6)	1034 (836 to 1270)	52 (34 to 77)	304 (239 to 396)	-
North Africa and Middle East										

Afghanistan (L)	84 (79 to 89)	78 (73 to 83)	340 (321 to 362)	315 (296 to 336)	14·1% (11·4 to 16·6)	13·7% (11·3 to 16·1)	5 (4 to 5)	0 (0 to 0)	68 (64 to 74)	11 (11 to 11)
Algeria (LM)	246 (228 to 266)	253 (231 to 277)	803 (747 to 870)	826 (757 to 905)	6·4% (5·9 to 6·9)	6·7% (6·1 to 7·3)	161 (146 to 178)	4 (2 to 5)	80 (68 to 93)	0 (0 to 0)
Bahrain (H)	1118 (1060 to 1176)	1179 (1101 to 1257)	2285 (2167 to 2404)	2409 (2250 to 2568)	4·5% (4·0 to 4·8)	4·7% (4·1 to 5·3)	661 (614 to 706)	125 (113 to 138)	332 (305 to 362)	-
Egypt (LM)	179 (162 to 196)	214 (193 to 234)	617 (559 to 674)	737 (666 to 806)	5·0% (4·3 to 5·6)	5·0% (4·3 to 5·6)	51 (43 to 60)	17 (12 to 23)	109 (95 to 123)	2 (2 to 2)
Iran (LM)	1082 (1018 to 1144)	1293 (1209 to 1368)	1090 (1026 to 1153)	1303 (1218 to 1378)	6·7% (6·3 to 7·1)	7·3% (6·8 to 7·7)	518 (475 to 564)	154 (131 to 182)	409 (371 to 453)	0 (0 to 0)
Iraq (UM)	250 (230 to 273)	242 (211 to 279)	526 (483 to 574)	509 (443 to 586)	4·2% (3·8 to 4·6)	4·3% (3·7 to 5·0)	113 (96 to 129)	0 (0 to 0)	136 (121 to 151)	1 (1 to 1)
Jordan (UM)	309 (286 to 339)	360 (330 to 398)	767 (709 to 840)	893 (817 to 988)	6·9% (6·4 to 7·6)	7·2% (6·5 to 7·9)	153 (136 to 171)	49 (38 to 63)	97 (84 to 110)	9 (9 to 9)
Kuwait (H)	1844 (1726 to 1978)	2064 (1794 to 2377)	2901 (2715 to 3112)	3246 (2822 to 3739)	5·5% (4·9 to 6·3)	6·1% (5·2 to 7·2)	1609 (1494 to 1745)	22 (20 to 25)	213 (193 to 232)	-
Lebanon (LM)	562 (534 to 588)	462 (430 to 497)	1572 (1495 to 1646)	1293 (1202 to 1390)	10·1% (9·6 to 10·6)	10·8% (9·7 to 12·3)	276 (257 to 297)	94 (87 to 100)	188 (174 to 204)	4 (4 to 4)
Libya (UM)	294 (254 to 339)	380 (313 to 459)	1028 (888 to 1184)	1326 (1094 to 1605)	5·0% (3·1 to 8·0)	4·9% (3·4 to 7·4)	208 (172 to 249)	8 (5 to 11)	76 (61 to 96)	1 (1 to 1)
Morocco (LM)	189 (172 to 208)	222 (201 to 245)	444 (404 to 488)	520 (471 to 574)	5·1% (4·6 to 5·7)	5·6% (5·0 to 6·2)	78 (66 to 91)	17 (12 to 25)	91 (79 to 104)	3 (3 to 3)
Oman (H)	817 (759 to 878)	839 (744 to 946)	1441 (1338 to 1548)	1480 (1312 to 1668)	4·5% (3·9 to 5·1)	4·6% (4·0 to 5·2)	711 (654 to 772)	54 (50 to 59)	52 (48 to 57)	-
Palestine (LM)	410 (374 to 442)	422 (385 to 458)	208 (189 to 224)	214 (195 to 232)	10·5% (9·4 to 11·6)	11·0% (9·9 to 12·2)	164 (148 to 185)	65 (49 to 83)	171 (151 to 195)	9 (9 to 9)
Qatar (H)	1938 (1808 to 2099)	2885 (2352 to 3487)	2957 (2759 to 3204)	4403 (3590 to 5322)	3·0% (2·8 to 3·2)	3·9% (3·2 to 4·9)	1430 (1293 to 1581)	284 (259 to 308)	223 (196 to 253)	-
Saudi Arabia (H)	1362 (1287 to 1441)	1654 (1494 to 1821)	2862 (2703 to 3026)	3475 (3139 to 3825)	5·5% (5·2 to 5·9)	6·4% (5·8 to 7·0)	958 (885 to 1028)	189 (169 to 210)	216 (201 to 229)	-
Sudan (L)	47 (42 to 52)	49 (44 to 56)	258 (231 to 290)	273 (244 to 310)	4·7% (3·3 to 6·4)	5·0% (3·6 to 6·5)	10 (9 to 11)	2 (1 to 2)	31 (26 to 37)	4 (4 to 4)
Syria (L)	31 (27 to 34)	31 (28 to 35)	1372 (1207 to 1543)	1399 (1236 to 1582)	2·7% (2·3 to 3·2)	2·9% (2·5 to 3·4)	14 (11 to 17)	1 (0 to 1)	14 (11 to 17)	2 (2 to 2)
Tunisia (LM)	279 (268 to 292)	301 (286 to 315)	826 (791 to 862)	888 (845 to 931)	6·7% (6·3 to 7·2)	7·0% (6·6 to 7·4)	158 (152 to 165)	13 (11 to 16)	106 (97 to 115)	1 (1 to 1)

Turkey (UM)	378 (358 to 398)	519 (483 to 554)	1377 (1307 to 1451)	1893 (1762 to 2022)	4·4% (4·2 to 4·6)	4·9% (4·6 to 5·3)	292 (273 to 310)	20 (14 to 28)	64 (61 to 66)	1 (1 to 1)
United Arab Emirates (H)	1983 (1906 to 2057)	2290 (2131 to 2461)	3401 (3269 to 3529)	3928 (3656 to 4221)	4·5% (4·3 to 4·7)	4·9% (4·5 to 5·3)	1042 (982 to 1107)	693 (666 to 723)	247 (213 to 284)	-
Yemen (L)	34 (29 to 39)	38 (33 to 44)	94 (82 to 108)	107 (92 to 121)	4·8% (3·5 to 7·2)	5·3% (4·2 to 6·9)	4 (3 to 5)	0 (0 to 0)	24 (19 to 29)	6 (6 to 6)
South Asia										
Bangladesh (LM)	50 (46 to 55)	69 (63 to 76)	130 (118 to 142)	179 (162 to 196)	2·5% (2·3 to 2·8)	2·5% (2·2 to 2·8)	9 (8 to 11)	1 (1 to 2)	37 (32 to 41)	3 (3 to 3)
Bhutan (LM)	126 (115 to 139)	137 (124 to 152)	467 (428 to 515)	509 (459 to 562)	3·6% (3·3 to 4·0)	3·6% (3·2 to 4·0)	84 (75 to 96)	2 (1 to 2)	18 (14 to 23)	22 (22 to 22)
India (LM)	69 (64 to 75)	93 (86 to 102)	223 (205 to 243)	300 (276 to 327)	3·0% (2·7 to 3·3)	3·1% (2·8 to 3·4)	22 (20 to 25)	8 (6 to 11)	39 (34 to 44)	1 (1 to 1)
Nepal (LM)	52 (46 to 58)	57 (51 to 65)	187 (166 to 211)	207 (183 to 234)	4·4% (3·8 to 4·9)	4·3% (3·7 to 5·0)	12 (11 to 14)	3 (2 to 5)	30 (24 to 37)	6 (6 to 6)
Pakistan (LM)	42 (35 to 49)	49 (41 to 57)	161 (135 to 189)	187 (158 to 219)	2·9% (2·4 to 3·5)	3·1% (2·5 to 3·7)	13 (11 to 16)	3 (2 to 4)	24 (18 to 30)	2 (2 to 2)
Southeast Asia, East Asia, and Oceania										
American Samoa (UM)	623 (519 to 730)	697 (583 to 817)	623 (519 to 730)	697 (583 to 817)	4·9% (4·0 to 5·9)	5·0% (4·0 to 6·1)	479 (385 to 582)	21 (14 to 31)	124 (93 to 159)	0 (0 to 0)
Cambodia (LM)	104 (98 to 111)	123 (116 to 132)	313 (293 to 333)	370 (347 to 395)	6·4% (5·8 to 6·9)	6·3% (5·7 to 6·8)	26 (22 to 31)	4 (3 to 6)	67 (62 to 71)	8 (8 to 8)
China (UM)	594 (570 to 621)	901 (847 to 964)	925 (888 to 968)	1404 (1320 to 1503)	5·3% (5·0 to 5·6)	5·9% (5·5 to 6·3)	334 (312 to 357)	50 (41 to 60)	210 (198 to 221)	0 (0 to 0)
Cook Islands (H)	815 (750 to 881)	750 (661 to 847)	996 (916 to 1076)	917 (808 to 1035)	3·4% (3·1 to 3·7)	3·3% (2·9 to 3·8)	689 (623 to 756)	5 (3 to 6)	51 (46 to 56)	-
Federated States of Micronesia (LM)	176 (163 to 190)	161 (142 to 181)	157 (146 to 170)	144 (127 to 161)	4·6% (4·1 to 5·0)	4·2% (3·6 to 4·8)	143 (130 to 157)	0 (0 to 0)	11 (9 to 12)	22 (22 to 22)
Fiji (UM)	228 (211 to 245)	253 (230 to 280)	533 (493 to 574)	593 (539 to 656)	3·8% (3·3 to 4·2)	4·2% (3·7 to 4·8)	149 (136 to 165)	37 (31 to 42)	30 (28 to 34)	11 (11 to 11)
Guam (H)	931 (795 to 1098)	926 (794 to 1088)	931 (795 to 1098)	926 (794 to 1088)	2·3% (1·9 to 2·7)	2·4% (2·0 to 3·0)	618 (489 to 772)	57 (36 to 87)	256 (197 to 325)	-
Indonesia (LM)	125 (119 to 131)	163 (150 to 175)	375 (357 to 393)	491 (452 to 528)	2·9% (2·7 to 3·0)	3·1% (2·9 to 3·3)	61 (58 to 65)	19 (17 to 21)	43 (40 to 47)	1 (1 to 1)
Kiribati (LM)	244 (226 to 264)	224 (199 to 250)	292 (270 to 316)	268 (238 to 299)	14·0% (13·0 to 15·2)	12·9% (11·5 to 14·5)	154 (139 to 169)	7 (6 to 9)	49 (38 to 64)	34 (34 to 34)
Laos (LM)	62 (56 to 67)	70 (63 to 76)	208 (190 to 227)	235 (214 to 258)	2·4% (2·2 to 2·7)	2·4% (2·2 to 2·7)	22 (18 to 27)	0 (0 to 0)	28 (25 to 31)	11 (11 to 11)

Malaysia (UM)	475 (452 to 498)	585 (553 to 621)	1237 (1177 to 1297)	1523 (1439 to 1617)	4·0% (3·8 to 4·2)	4·4% (4·1 to 4·7)	247 (231 to 266)	65 (53 to 82)	163 (158 to 168)	0 (0 to 0)
Maldives (UM)	976 (909 to 1058)	1212 (1102 to 1326)	1944 (1810 to 2106)	2413 (2194 to 2641)	7·9% (5·9 to 9·5)	8·6% (7·0 to 10·2)	757 (689 to 833)	40 (29 to 53)	171 (151 to 193)	8 (8 to 8)
Marshall Islands (UM)	658 (630 to 690)	628 (568 to 695)	574 (550 to 603)	548 (496 to 607)	14·6% (13·5 to 15·6)	13·6% (12·2 to 15·1)	332 (307 to 362)	31 (22 to 42)	91 (82 to 100)	203 (203 to 203)
Mauritius (UM)	612 (597 to 628)	694 (666 to 723)	1554 (1515 to 1595)	1762 (1690 to 1835)	6·2% (6·0 to 6·4)	6·5% (6·2 to 6·8)	288 (277 to 300)	42 (40 to 45)	282 (271 to 294)	0 (0 to 0)
Myanmar (LM)	65 (56 to 76)	61 (52 to 70)	239 (205 to 278)	221 (191 to 257)	4·4% (3·7 to 5·2)	4·7% (4·0 to 5·7)	11 (9 to 13)	0 (0 to 0)	51 (42 to 61)	4 (4 to 4)
Nauru (H)	1311 (1214 to 1412)	1205 (1057 to 1384)	1329 (1231 to 1432)	1222 (1072 to 1404)	10·2% (6·0 to 15·1)	8·9% (5·4 to 13·6)	1032 (950 to 1128)	92 (76 to 110)	186 (146 to 238)	-
Niue (H)	2642 (2370 to 2932)	1704 (1355 to 2152)	1917 (1720 to 2128)	1237 (983 to 1562)	12·5% (10·9 to 14·6)	6·8% (5·2 to 8·7)	1355 (1088 to 1645)	62 (39 to 93)	231 (177 to 298)	-
North Korea (L)	39 (33 to 46)	40 (34 to 47)	23 (20 to 27)	24 (20 to 28)	5·7% (4·7 to 6·9)	5·9% (4·7 to 7·2)	23 (18 to 28)	1 (0 to 1)	15 (12 to 20)	0 (0 to 0)
Northern Mariana Islands (H)	451 (378 to 528)	452 (375 to 550)	451 (378 to 528)	452 (375 to 550)	2·1% (1·7 to 2·6)	2·5% (2·0 to 3·2)	288 (226 to 352)	24 (16 to 38)	139 (103 to 183)	-
Palau (UM)	1885 (1804 to 1965)	2200 (2035 to 2383)	1855 (1776 to 1934)	2166 (2003 to 2346)	11·6% (10·9 to 12·3)	13·5% (12·4 to 14·6)	1179 (1103 to 1261)	414 (389 to 441)	291 (277 to 304)	0 (0 to 0)
Papua New Guinea (LM)	71 (67 to 76)	74 (67 to 81)	93 (88 to 99)	97 (88 to 106)	2·3% (2·1 to 2·4)	2·3% (2·1 to 2·6)	46 (42 to 50)	0 (0 to 0)	6 (5 to 7)	19 (19 to 19)
Philippines (LM)	156 (140 to 173)	194 (173 to 214)	401 (359 to 443)	498 (445 to 549)	4·1% (3·7 to 4·5)	4·4% (3·9 to 4·9)	61 (52 to 71)	18 (12 to 24)	76 (64 to 88)	2 (2 to 2)
Samoa (LM)	274 (257 to 293)	266 (246 to 289)	391 (366 to 418)	379 (350 to 412)	6·1% (5·6 to 6·5)	6·2% (5·7 to 6·7)	189 (173 to 206)	2 (1 to 3)	27 (22 to 34)	56 (56 to 56)
Seychelles (H)	695 (672 to 718)	845 (778 to 914)	1490 (1440 to 1539)	1811 (1667 to 1959)	4·7% (4·4 to 5·0)	5·0% (4·5 to 5·7)	509 (496 to 523)	12 (10 to 14)	174 (156 to 194)	-
Solomon Islands (LM)	114 (106 to 124)	124 (101 to 153)	119 (110 to 128)	129 (104 to 159)	4·5% (4·2 to 4·9)	5·3% (4·1 to 6·5)	80 (71 to 89)	0 (0 to 0)	4 (3 to 4)	31 (31 to 31)
Sri Lanka (LM)	154 (140 to 169)	148 (134 to 164)	584 (531 to 640)	563 (509 to 623)	4·0% (3·6 to 4·4)	4·2% (3·8 to 4·7)	70 (63 to 78)	10 (6 to 14)	71 (59 to 83)	3 (3 to 3)
Taiwan (province of China) (H)	1556 (1468 to 1652)	2161 (2028 to 2309)	2880 (2717 to 3058)	4002 (3754 to 4274)	5·1% (4·8 to 5·5)	5·7% (5·3 to 6·2)	1196 (1148 to 1243)	58 (38 to 85)	302 (234 to 386)	-
Thailand (UM)	310 (304 to 315)	398 (384 to 414)	811 (797 to 825)	1043 (1005 to 1085)	4·0% (3·9 to 4·1)	4·7% (4·5 to 4·9)	220 (216 to 223)	60 (58 to 62)	30 (26 to 33)	1 (1 to 1)

Timor-Leste (LM)	117 (106 to 131)	127 (110 to 150)	193 (174 to 216)	209 (181 to 246)	5·1% (4·6 to 5·7)	4·9% (4·2 to 5·7)	80 (70 to 93)	6 (4 to 10)	11 (9 to 15)	19 (19 to 19)
Tokelau (UM)	2055 (1865 to 2255)	2133 (1885 to 2404)	2684 (2436 to 2945)	2786 (2462 to 3139)	22·9% (20·6 to 25·5)	19·6% (17·2 to 22·2)	743 (598 to 914)	63 (41 to 93)	383 (289 to 495)	867 (867 to 867)
Tonga (UM)	209 (200 to 218)	214 (201 to 226)	270 (259 to 283)	277 (261 to 293)	4·2% (4·0 to 4·5)	4·1% (3·7 to 4·6)	140 (134 to 146)	9 (7 to 12)	25 (20 to 31)	35 (35 to 35)
Tuvalu (UM)	969 (928 to 1004)	1209 (1034 to 1432)	885 (848 to 918)	1105 (945 to 1308)	18·5% (16·9 to 19·9)	19·4% (16·2 to 22·9)	755 (719 to 788)	0 (0 to 0)	84 (64 to 106)	130 (130 to 130)
Vanuatu (LM)	106 (95 to 118)	92 (76 to 113)	94 (85 to 105)	82 (68 to 101)	3·0% (2·7 to 3·4)	2·9% (2·3 to 3·6)	66 (55 to 78)	4 (3 to 6)	10 (8 to 12)	25 (25 to 25)
Vietnam (LM)	180 (162 to 200)	254 (225 to 287)	558 (501 to 618)	788 (698 to 888)	5·5% (4·4 to 7·2)	5·8% (4·7 to 7·5)	80 (69 to 93)	20 (13 to 28)	79 (66 to 93)	2 (2 to 2)
Sub-Saharan Africa										
Angola (LM)	68 (60 to 78)	66 (57 to 75)	203 (179 to 230)	195 (170 to 222)	2·7% (2·3 to 3·1)	2·8% (2·4 to 3·3)	29 (23 to 34)	12 (8 to 17)	24 (19 to 30)	3 (3 to 3)
Benin (LM)	33 (29 to 37)	38 (34 to 43)	89 (79 to 99)	103 (91 to 117)	2·4% (2·2 to 2·7)	2·4% (2·1 to 2·7)	7 (6 to 8)	2 (1 to 3)	14 (11 to 18)	11 (11 to 11)
Botswana (UM)	503 (474 to 534)	559 (511 to 608)	1205 (1134 to 1280)	1340 (1225 to 1456)	6·5% (6·1 to 7·0)	6·7% (6·2 to 7·4)	389 (364 to 415)	67 (52 to 84)	15 (14 to 17)	32 (32 to 32)
Burkina Faso (L)	49 (46 to 52)	57 (53 to 62)	137 (128 to 145)	158 (146 to 171)	5·7% (5·4 to 6·1)	5·8% (5·3 to 6·2)	19 (17 to 22)	3 (2 to 5)	15 (14 to 16)	12 (12 to 12)
Burundi (L)	32 (30 to 34)	36 (32 to 42)	93 (88 to 99)	106 (93 to 123)	10·7% (9·2 to 12·0)	12·0% (9·9 to 14·4)	7 (6 to 8)	4 (3 to 5)	5 (5 to 6)	16 (16 to 16)
Cameroon (LM)	50 (48 to 53)	58 (55 to 62)	124 (118 to 130)	144 (135 to 153)	3·0% (2·9 to 3·2)	3·2% (3·0 to 3·5)	3 (2 to 3)	5 (3 to 6)	36 (34 to 39)	7 (7 to 7)
Cape Verde (LM)	231 (217 to 248)	262 (234 to 291)	468 (439 to 502)	530 (475 to 589)	5·9% (5·6 to 6·4)	6·3% (5·6 to 6·9)	127 (112 to 143)	5 (4 to 7)	50 (48 to 53)	49 (49 to 49)
Central African Republic (L)	43 (41 to 45)	47 (42 to 52)	83 (79 to 87)	91 (82 to 101)	8·0% (7·6 to 8·3)	8·2% (7·4 to 9·1)	4 (3 to 4)	0 (0 to 1)	21 (19 to 23)	17 (17 to 17)
Chad (L)	34 (32 to 36)	33 (30 to 35)	77 (73 to 81)	75 (70 to 80)	4·3% (4·0 to 4·7)	4·4% (4·0 to 4·8)	5 (5 to 6)	2 (1 to 2)	19 (17 to 21)	7 (7 to 7)
Comoros (LM)	108 (105 to 112)	147 (135 to 165)	244 (235 to 251)	332 (303 to 372)	7·1% (6·2 to 7·8)	9·4% (7·8 to 11·1)	13 (11 to 16)	4 (3 to 6)	58 (55 to 61)	33 (33 to 33)
Congo (Brazzaville) (LM)	60 (53 to 67)	59 (50 to 67)	96 (86 to 107)	95 (81 to 108)	2·1% (1·8 to 2·5)	2·4% (2·0 to 2·8)	21 (18 to 25)	4 (3 to 6)	26 (21 to 32)	8 (8 to 8)
Côte d'Ivoire (LM)	78 (70 to 86)	104 (93 to 116)	184 (166 to 203)	245 (221 to 274)	3·1% (2·8 to 3·5)	3·4% (3·0 to 3·8)	23 (22 to 24)	16 (10 to 23)	30 (25 to 35)	9 (9 to 9)
DR Congo (L)	25 (24 to 27)	29 (26 to 32)	50 (48 to 53)	57 (52 to 64)	4·2% (4·0 to 4·4)	4·1% (3·7 to 4·6)	3 (3 to 4)	2 (1 to 2)	9 (8 to 10)	12 (12 to 12)

Djibouti (LM)	60 (59 to 62)	72 (66 to 78)	104 (101 to 107)	124 (114 to 134)	1·7% (1·7 to 1·8)	1·7% (1·5 to 1·8)	28 (27 to 29)	1 (1 to 1)	13 (12 to 15)	18 (18 to 18)
Equatorial Guinea (UM)	306 (277 to 338)	241 (218 to 268)	608 (551 to 672)	480 (434 to 533)	3·0% (2·7 to 3·3)	3·3% (2·9 to 3·9)	58 (52 to 64)	7 (6 to 10)	228 (198 to 261)	12 (12 to 12)
Eritrea (L)	16 (14 to 18)	21 (18 to 24)	49 (44 to 55)	64 (56 to 73)	2·5% (2·3 to 2·8)	3·1% (2·7 to 3·5)	3 (2 to 3)	0 (0 to 0)	7 (5 to 8)	7 (7 to 7)
eSwatini (LM)	282 (269 to 296)	356 (318 to 399)	665 (636 to 700)	840 (752 to 941)	6·9% (6·6 to 7·4)	8·3% (7·3 to 9·3)	138 (131 to 146)	37 (28 to 48)	29 (23 to 36)	77 (77 to 77)
Ethiopia (L)	25 (24 to 27)	31 (29 to 33)	80 (75 to 85)	98 (91 to 106)	3·1% (2·6 to 3·5)	2·9% (2·5 to 3·4)	7 (6 to 8)	2 (1 to 3)	10 (9 to 11)	7 (7 to 7)
Gabon (UM)	335 (317 to 355)	395 (372 to 419)	618 (585 to 655)	728 (686 to 772)	3·7% (3·5 to 4·0)	4·2% (3·8 to 4·6)	186 (171 to 200)	48 (46 to 50)	71 (59 to 84)	31 (31 to 31)
Ghana (LM)	76 (71 to 81)	90 (84 to 97)	193 (181 to 206)	229 (213 to 246)	3·6% (2·9 to 4·8)	3·6% (3·0 to 4·6)	30 (27 to 34)	9 (9 to 10)	28 (25 to 32)	8 (8 to 8)
Guinea (L)	52 (45 to 60)	67 (57 to 78)	117 (102 to 135)	152 (130 to 177)	3·9% (3·2 to 5·0)	4·2% (3·4 to 5·1)	8 (7 to 10)	4 (3 to 6)	31 (24 to 38)	8 (8 to 8)
Guinea-Bissau (L)	68 (63 to 72)	80 (74 to 87)	199 (185 to 213)	236 (217 to 254)	8·4% (7·4 to 9·5)	9·1% (7·7 to 10·5)	4 (3 to 5)	2 (1 to 3)	39 (34 to 43)	23 (23 to 23)
Kenya (LM)	91 (83 to 100)	113 (102 to 125)	227 (207 to 250)	283 (255 to 314)	4·4% (3·9 to 5·0)	4·7% (4·1 to 5·4)	40 (33 to 49)	11 (8 to 14)	22 (18 to 27)	17 (17 to 17)
Lesotho (LM)	132 (127 to 138)	153 (141 to 167)	313 (301 to 327)	364 (334 to 396)	10·5% (10·1 to 11·0)	12·0% (10·7 to 13·4)	70 (65 to 76)	1 (0 to 1)	21 (20 to 22)	40 (40 to 40)
Liberia (L)	60 (56 to 64)	88 (78 to 100)	136 (128 to 145)	200 (176 to 226)	9·3% (7·3 to 12·8)	12·4% (9·3 to 16·7)	7 (6 to 9)	3 (2 to 5)	27 (24 to 31)	22 (22 to 22)
Madagascar (L)	20 (18 to 23)	22 (19 to 24)	66 (60 to 74)	71 (63 to 79)	3·8% (3·4 to 4·2)	3·9% (3·5 to 4·4)	9 (8 to 10)	1 (1 to 2)	6 (5 to 8)	4 (4 to 4)
Malawi (L)	44 (42 to 45)	53 (48 to 58)	116 (113 to 120)	140 (128 to 155)	8·0% (6·3 to 10·5)	8·9% (6·7 to 11·8)	11 (10 to 12)	2 (2 to 3)	6 (5 to 6)	25 (25 to 25)
Mali (L)	32 (30 to 34)	33 (30 to 36)	86 (80 to 92)	87 (81 to 95)	3·1% (2·4 to 3·7)	3·0% (2·3 to 3·7)	11 (10 to 12)	1 (0 to 1)	11 (9 to 12)	10 (10 to 10)
Mauritania (LM)	72 (69 to 76)	83 (78 to 88)	212 (202 to 223)	245 (228 to 260)	3·6% (3·1 to 4·0)	3·7% (3·1 to 4·3)	26 (24 to 29)	4 (3 to 6)	32 (30 to 34)	9 (9 to 9)
Mozambique (L)	31 (28 to 34)	39 (35 to 43)	83 (76 to 90)	104 (94 to 116)	5·9% (5·4 to 6·4)	6·7% (6·0 to 7·5)	9 (7 to 11)	3 (2 to 4)	4 (3 to 5)	15 (15 to 15)
Namibia (UM)	486 (458 to 518)	489 (456 to 528)	977 (920 to 1042)	984 (917 to 1062)	9·0% (8·5 to 9·6)	9·6% (8·9 to 10·3)	226 (209 to 247)	193 (173 to 216)	40 (32 to 50)	26 (26 to 26)
Niger (L)	37 (35 to 39)	43 (40 to 46)	81 (78 to 85)	94 (88 to 101)	6·0% (5·7 to 6·3)	5·6% (5·1 to 6·1)	12 (10 to 13)	1 (1 to 2)	16 (15 to 17)	8 (8 to 8)

Nigeria (LM)	62 (57 to 68)	63 (57 to 69)	163 (148 to 177)	165 (149 to 182)	2·9% (2·6 to 3·1)	2·9% (2·6 to 3·2)	10 (8 to 12)	1 (0 to 1)	47 (43 to 53)	4 (4 to 4)
Rwanda (L)	56 (52 to 61)	63 (57 to 71)	166 (154 to 181)	187 (168 to 210)	6·7% (6·2 to 7·4)	6·2% (5·4 to 7·1)	20 (17 to 25)	8 (6 to 10)	6 (5 to 7)	22 (22 to 22)
Senegal (LM)	71 (63 to 79)	86 (77 to 97)	166 (149 to 186)	202 (181 to 227)	4·4% (4·0 to 5·0)	4·3% (3·8 to 4·9)	17 (14 to 21)	4 (3 to 6)	36 (29 to 43)	13 (13 to 13)
Sierra Leone (L)	45 (43 to 48)	67 (60 to 76)	159 (149 to 169)	235 (209 to 266)	8·5% (7·9 to 9·0)	11·5% (10·2 to 13·0)	5 (4 to 7)	1 (0 to 1)	24 (22 to 27)	15 (15 to 15)
Somalia (L)	7 (6 to 7)	8 (7 to 10)	18 (17 to 19)	22 (18 to 26)	4·2% (3·8 to 4·6)	5·5% (4·6 to 6·8)	1 (0 to 1)	0 (0 to 0)	2 (1 to 2)	4 (4 to 4)
South Africa (UM)	631 (605 to 656)	676 (639 to 711)	1307 (1253 to 1359)	1400 (1324 to 1472)	9·0% (8·3 to 10·0)	9·9% (9·1 to 10·8)	367 (355 to 378)	219 (197 to 242)	36 (34 to 38)	9 (9 to 9)
South Sudan (L)	41 (39 to 42)	36 (32 to 41)	94 (91 to 98)	83 (74 to 95)	9·8% (4·5 to 15·8)	9·6% (2·9 to 24·9)	4 (3 to 5)	2 (1 to 2)	7 (6 to 8)	28 (28 to 28)
São Tomé and Príncipe (LM)	189 (177 to 202)	275 (249 to 306)	339 (318 to 362)	493 (447 to 549)	8·2% (7·7 to 8·8)	11·2% (10·1 to 12·5)	60 (50 to 71)	2 (1 to 3)	22 (18 to 28)	104 (104 to 104)
Tanzania (LM)	42 (41 to 44)	49 (44 to 54)	111 (107 to 116)	128 (116 to 142)	3·8% (3·6 to 4·0)	3·8% (3·4 to 4·2)	18 (17 to 20)	0 (0 to 1)	10 (9 to 11)	14 (14 to 14)
The Gambia (L)	42 (40 to 44)	57 (50 to 63)	125 (118 to 132)	169 (149 to 189)	5·0% (4·7 to 5·4)	6·1% (5·4 to 6·8)	9 (7 to 11)	1 (1 to 2)	7 (6 to 9)	24 (24 to 24)
Togo (L)	59 (55 to 64)	75 (69 to 81)	141 (131 to 152)	179 (165 to 194)	6·8% (5·5 to 8·9)	7·2% (5·8 to 9·2)	9 (7 to 11)	5 (3 to 7)	37 (34 to 41)	8 (8 to 8)
Uganda (L)	41 (38 to 44)	46 (42 to 51)	112 (104 to 122)	127 (116 to 139)	4·2% (3·7 to 4·8)	4·3% (3·8 to 4·8)	7 (6 to 8)	2 (1 to 2)	15 (12 to 18)	18 (18 to 18)
Zambia (L)	60 (55 to 66)	67 (58 to 77)	199 (184 to 219)	223 (194 to 255)	5·2% (4·6 to 5·7)	5·9% (5·1 to 6·7)	23 (19 to 28)	4 (2 to 5)	6 (5 to 8)	28 (28 to 28)
Zimbabwe (LM)	54 (50 to 59)	59 (53 to 65)	60 (56 to 66)	65 (59 to 73)	2·5% (2·3 to 2·7)	2·5% (2·2 to 2·8)	12 (10 to 14)	11 (8 to 15)	11 (9 to 14)	20 (20 to 20)

S1.3

T1.1.3 Estimates of 2019 health expenditure by source as percent of total health expenditure, and absolute development assistance for health in millions of USD

	Government health spending per total health spending, 2019 (2021 US dollars)	Out-of-pocket spending per total health spending, 2019 (2021 US dollars)	Prepaid private health spending per total health spending, 2019 (2021 US dollars)	Development assistance for health spending per total health spending, 2019 (2021 US dollars)	Development assistance for health, 2019 (millions 2021 US dollars)
Global					
Total	59.8% (59.8% to 59.8%)	18.2% (17.9% to 18.5%)	21.5% (21.1% to 21.9%)	0.5% (0.5% to 0.5%)	43117.6 (43117.6 to 43117.6)
World Bank income groups					
High-income	62.0% (62.0% to 61.9%)	13.7% (13.4% to 14.2%)	24.3% (23.7% to 24.7%)	-	-
Upper-middle-income	56.0% (55.1% to 56.9%)	32.6% (32.2% to 32.9%)	11.3% (10.6% to 11.9%)	0.2% (0.2% to 0.1%)	2194.1 (2194.1 to 2194.1)
Lower-middle-income	39.9% (39.6% to 40.2%)	47.3% (46.5% to 48.1%)	10.6% (9.8% to 11.4%)	2.2% (2.3% to 2.2%)	8733.3 (8733.3 to 8733.3)
Low-income	23.0% (22.5% to 23.4%)	43.8% (43.0% to 44.6%)	4.7% (4.4% to 5.1%)	28.5% (29.1% to 28.0%)	7070.2 (7070.2 to 7070.2)
GBD super-region					
Central Europe, Eastern Europe, and Central Asia	64.7% (64.4% to 64.8%)	31.4% (31.2% to 31.6%)	3.8% (3.5% to 4.0%)	0.2% (0.2% to 0.2%)	602.6 (602.6 to 602.6)
GBD high-income	61.6% (61.7% to 61.5%)	13.7% (13.3% to 14.1%)	24.7% (24.2% to 25.2%)	-	0.0 (0.0 to 0.0)
Latin America and Caribbean	51.3% (50.7% to 51.8%)	28.9% (28.5% to 29.3%)	19.5% (18.5% to 20.4%)	0.3% (0.3% to 0.3%)	962.4 (962.4 to 962.4)

North Africa and Middle East	56.4% (56.0% to 56.9%)	30.9% (30.3% to 31.5%)	12.2% (11.7% to 12.8%)	0.5% (0.5% to 0.5%)	1469.3 (1469.3 to 1469.3)
South Asia	30.9% (30.4% to 31.7%)	56.9% (55.3% to 58.9%)	10.6% (8.7% to 12.9%)	1.6% (1.7% to 1.5%)	1818.8 (1818.8 to 1818.8)
Southeast Asia, East Asia, and Oceania	56.3% (55.0% to 57.3%)	34.7% (34.2% to 35.1%)	8.8% (7.8% to 9.8%)	0.2% (0.2% to 0.2%)	1836.8 (1836.8 to 1836.8)
Sub-Saharan Africa	40.2% (40.1% to 40.4%)	28.6% (27.8% to 29.3%)	18.4% (17.5% to 19.6%)	12.8% (13.1% to 12.5%)	11307.7 (11307.7 to 11307.7)
Country					
Central Europe, Eastern Europe, and Central Asia					
Albania (UM)	54.1% (51.5% to 56.5%)	44.7% (44.8% to 44.6%)	0.0% (0.0% to 0.1%)	1.1% (1.2% to 1.0%)	10.2 (10.2 to 10.2)
Armenia (UM)	12.5% (12.6% to 12.5%)	84.5% (83.7% to 85.1%)	1.7% (1.2% to 2.3%)	1.3% (1.4% to 1.3%)	21.0 (21.0 to 21.0)
Azerbaijan (UM)	29.7% (29.8% to 29.8%)	69.8% (67.5% to 71.5%)	0.0% (0.0% to 0.0%)	0.4% (0.5% to 0.4%)	8.8 (8.8 to 8.8)
Belarus (UM)	69.9% (69.1% to 70.3%)	26.0% (24.6% to 27.5%)	3.7% (2.9% to 4.4%)	0.4% (0.5% to 0.4%)	16.7 (16.7 to 16.7)
Bosnia and Herzegovina (UM)	69.9% (69.3% to 70.0%)	29.5% (28.0% to 30.7%)	0.4% (0.3% to 0.6%)	0.2% (0.2% to 0.2%)	4.3 (4.3 to 4.3)
Bulgaria (UM)	58.6% (58.1% to 59.4%)	39.5% (38.7% to 40.3%)	1.9% (1.3% to 2.6%)	0.0% (0.0% to 0.0%)	0.3 (0.3 to 0.3)
Croatia (H)	82.2% (81.6% to 82.7%)	11.0% (10.3% to 11.7%)	6.7% (6.1% to 7.4%)	-	-

Czechia (H)	81.8% (81.6% to 82.1%)	14.2% (13.9% to 14.5%)	4.0% (3.7% to 4.4%)	-	-
Estonia (H)	74.2% (74.2% to 74.1%)	24.3% (23.6% to 24.8%)	1.6% (1.4% to 1.8%)	-	-
Georgia (UM)	40.2% (38.1% to 42.0%)	44.8% (45.3% to 43.9%)	10.5% (8.5% to 13.5%)	4.4% (4.7% to 4.2%)	56.9 (56.9 to 56.9)
Hungary (H)	68.5% (68.0% to 68.9%)	27.6% (27.1% to 28.0%)	3.9% (3.7% to 4.2%)	-	-
Kazakhstan (UM)	59.9% (57.7% to 61.2%)	34.1% (32.8% to 35.2%)	5.6% (4.1% to 7.6%)	0.4% (0.4% to 0.4%)	21.3 (21.3 to 21.3)
Kyrgyzstan (LM)	44.8% (42.2% to 47.6%)	46.7% (43.1% to 50.8%)	0.0% (0.0% to 0.1%)	8.4% (9.6% to 7.5%)	41.0 (41.0 to 41.0)
Latvia (H)	60.4% (59.9% to 60.7%)	37.4% (36.4% to 38.1%)	2.3% (1.7% to 2.9%)	-	-
Lithuania (H)	65.6% (65.0% to 65.9%)	32.1% (31.8% to 32.6%)	2.3% (2.1% to 2.4%)	-	-
Moldova (UM)	58.5% (56.4% to 60.4%)	38.6% (37.3% to 39.4%)	1.1% (0.8% to 1.5%)	1.8% (2.0% to 1.7%)	15.6 (15.6 to 15.6)
Mongolia (LM)	55.2% (54.1% to 56.5%)	31.9% (31.0% to 33.1%)	3.3% (2.3% to 4.6%)	9.5% (10.0% to 9.1%)	59.9 (59.9 to 59.9)
Montenegro (UM)	59.6% (58.2% to 60.8%)	39.3% (38.6% to 40.2%)	0.8% (0.6% to 1.2%)	0.3% (0.3% to 0.3%)	1.3 (1.3 to 1.3)
North Macedonia (UM)	58.1% (56.7% to 59.8%)	41.2% (42.0% to 40.4%)	0.6% (0.4% to 0.8%)	0.1% (0.1% to 0.1%)	1.4 (1.4 to 1.4)

Poland (H)	71.4% (71.0% to 71.5%)	20.5% (20.1% to 20.9%)	8.1% (7.8% to 8.4%)	-	-
Romania (H)	80.2% (79.8% to 80.6%)	18.8% (18.8% to 19.0%)	1.0% (0.8% to 1.1%)	-	-
Russia (UM)	60.7% (59.9% to 61.3%)	36.9% (36.6% to 37.3%)	2.3% (1.7% to 3.0%)	0.0% (0.0% to 0.0%)	0.0 (0.0 to 0.0)
Serbia (UM)	59.0% (57.9% to 60.4%)	37.9% (37.6% to 38.0%)	2.2% (1.6% to 3.1%)	0.9% (0.9% to 0.8%)	44.4 (44.4 to 44.4)
Slovakia (H)	79.3% (79.0% to 79.7%)	18.6% (17.4% to 19.8%)	2.1% (1.7% to 2.7%)	-	-
Slovenia (H)	72.4% (71.8% to 73.0%)	11.7% (11.6% to 11.8%)	15.9% (15.7% to 16.2%)	-	-
Tajikistan (LM)	26.2% (26.5% to 25.9%)	64.4% (61.7% to 67.3%)	0.2% (0.1% to 0.2%)	9.3% (10.1% to 8.6%)	49.9 (49.9 to 49.9)
Turkmenistan (UM)	18.6% (17.8% to 19.3%)	76.1% (75.3% to 77.1%)	5.1% (4.1% to 6.1%)	0.2% (0.2% to 0.2%)	6.0 (6.0 to 6.0)
Ukraine (LM)	45.6% (44.6% to 46.7%)	50.8% (50.9% to 50.7%)	2.6% (1.8% to 3.7%)	1.0% (1.0% to 1.0%)	145.0 (145.0 to 145.0)
Uzbekistan (LM)	39.8% (39.3% to 40.3%)	56.7% (55.6% to 57.8%)	0.7% (0.4% to 0.9%)	2.8% (2.9% to 2.7%)	98.6 (98.6 to 98.6)
GBD high-income					
Andorra (H)	68.8% (67.3% to 70.2%)	13.3% (13.2% to 13.4%)	17.9% (17.5% to 18.3%)	-	-
Argentina (UM)	62.0% (61.4% to 62.7%)	27.6% (27.1% to 28.0%)	10.2% (8.8% to 11.8%)	0.2% (0.2% to 0.2%)	94.2 (94.2 to 94.2)

Australia (H)	71.1% (71.2% to 71.2%)	16.3% (16.0% to 16.6%)	12.6% (12.0% to 13.3%)	-	-
Austria (H)	73.0% (72.7% to 73.1%)	19.2% (18.9% to 19.5%)	7.9% (7.6% to 8.1%)	-	-
Belgium (H)	76.8% (76.5% to 77.2%)	18.1% (17.9% to 18.3%)	5.0% (4.9% to 5.1%)	-	-
Brunei (H)	94.7% (94.2% to 95.1%)	5.3% (4.8% to 6.0%)	0.0% (0.0% to 0.0%)	-	-
Canada (H)	70.2% (70.1% to 70.3%)	14.9% (14.7% to 15.2%)	14.9% (14.5% to 15.4%)	-	-
Chile (H)	51.0% (50.7% to 51.4%)	32.7% (32.5% to 33.0%)	16.2% (15.8% to 16.7%)	-	-
Cyprus (H)	50.4% (49.5% to 51.4%)	36.4% (35.2% to 37.8%)	13.2% (12.6% to 13.6%)	-	-
Denmark (H)	83.4% (83.1% to 83.7%)	14.1% (14.1% to 14.1%)	2.5% (2.5% to 2.6%)	-	-
Finland (H)	79.8% (79.7% to 80.0%)	17.6% (17.3% to 17.8%)	2.6% (2.5% to 2.7%)	-	-
France (H)	75.6% (75.7% to 75.5%)	9.3% (9.0% to 9.6%)	15.1% (14.3% to 16.0%)	-	-
Germany (H)	77.7% (77.6% to 77.9%)	12.8% (12.6% to 13.0%)	9.5% (9.4% to 9.6%)	-	-
Greece (H)	49.6% (48.1% to 50.7%)	35.6% (35.0% to 36.1%)	14.8% (14.0% to 15.5%)	-	-

Greenland (H)	100.0% (100.0% to 100.0%)	0.0% (0.0% to 0.0%)	0.0% (0.0% to 0.0%)	-	-
Iceland (H)	82.8% (82.2% to 83.5%)	15.6% (15.4% to 15.6%)	1.7% (1.7% to 1.7%)	-	-
Ireland (H)	74.4% (73.7% to 75.2%)	11.8% (11.4% to 12.1%)	13.8% (13.2% to 14.5%)	-	-
Israel (H)	66.1% (65.9% to 66.3%)	21.6% (21.0% to 22.1%)	12.4% (12.0% to 12.7%)	-	-
Italy (H)	73.9% (73.4% to 74.3%)	23.4% (23.2% to 23.5%)	2.8% (2.7% to 2.8%)	-	-
Japan (H)	83.9% (83.4% to 84.3%)	12.9% (12.9% to 12.8%)	3.2% (3.2% to 3.3%)	-	-
Luxembourg (H)	86.6% (86.2% to 87.2%)	10.1% (9.4% to 10.8%)	3.2% (3.0% to 3.6%)	-	-
Malta (H)	63.4% (63.1% to 64.1%)	34.3% (33.8% to 35.0%)	2.3% (2.1% to 2.4%)	-	-
Monaco (H)	85.4% (84.6% to 86.0%)	7.5% (7.6% to 7.5%)	7.1% (6.0% to 8.1%)	-	-
Netherlands (H)	65.8% (65.5% to 66.1%)	10.6% (10.2% to 10.9%)	23.6% (22.9% to 24.3%)	-	-
New Zealand (H)	75.4% (75.1% to 75.7%)	12.4% (12.0% to 12.8%)	12.2% (11.8% to 12.5%)	-	-
Norway (H)	85.8% (85.5% to 86.2%)	13.8% (13.6% to 14.0%)	0.3% (0.3% to 0.4%)	-	-

Portugal (H)	60.9% (60.1% to 61.5%)	30.3% (30.1% to 30.6%)	8.8% (7.8% to 9.8%)	-	-
San Marino (H)	81.9% (81.4% to 82.4%)	17.0% (16.6% to 17.4%)	1.1% (1.0% to 1.1%)	-	-
Singapore (H)	50.6% (49.6% to 51.5%)	29.8% (29.5% to 30.4%)	19.6% (19.0% to 20.3%)	-	-
South Korea (H)	59.5% (59.1% to 59.8%)	30.5% (30.4% to 30.6%)	10.0% (9.9% to 10.2%)	-	-
Spain (H)	70.5% (70.0% to 71.2%)	21.9% (21.8% to 21.9%)	7.6% (7.2% to 7.9%)	-	-
Sweden (H)	84.9% (84.3% to 85.4%)	13.9% (13.9% to 13.9%)	1.2% (1.2% to 1.3%)	-	-
Switzerland (H)	31.9% (31.7% to 32.1%)	26.0% (25.5% to 26.6%)	42.1% (41.5% to 42.5%)	-	-
UK (H)	79.5% (79.4% to 79.6%)	17.0% (16.5% to 17.5%)	3.5% (3.4% to 3.6%)	-	-
USA (H)	50.8% (50.7% to 50.8%)	11.3% (10.6% to 12.3%)	37.9% (37.1% to 38.5%)	-	-
Uruguay (H)	71.8% (71.7% to 71.9%)	16.7% (16.5% to 16.9%)	11.5% (11.1% to 12.0%)	-	-
Latin America and Caribbean					
Antigua and Barbuda (H)	59.0% (57.3% to 60.5%)	24.4% (23.0% to 25.7%)	16.6% (16.8% to 16.5%)	-	-
Barbados (H)	44.7% (42.9% to 46.7%)	47.5% (47.7% to 47.5%)	7.7% (7.7% to 7.8%)	-	-

Belize (UM)	68.5% (66.7% to 70.3%)	22.0% (22.2% to 21.7%)	5.4% (4.9% to 5.9%)	4.1% (4.4% to 3.9%)	4.8 (4.8 to 4.8)
Bermuda (H)	28.2% (28.1% to 28.3%)	10.0% (9.1% to 11.0%)	61.8% (56.4% to 66.4%)	-	-
Bolivia (LM)	70.0% (69.9% to 69.9%)	22.8% (21.7% to 23.9%)	3.9% (2.6% to 5.4%)	3.4% (3.5% to 3.2%)	97.3 (97.3 to 97.3)
Brazil (UM)	42.3% (40.6% to 44.1%)	25.1% (24.5% to 25.9%)	32.4% (31.2% to 33.7%)	0.1% (0.1% to 0.1%)	197.0 (197.0 to 197.0)
Colombia (UM)	71.8% (71.0% to 73.0%)	14.9% (14.0% to 15.9%)	13.2% (12.6% to 13.7%)	0.1% (0.1% to 0.1%)	15.3 (15.3 to 15.3)
Costa Rica (UM)	71.9% (70.8% to 72.1%)	21.5% (20.5% to 22.4%)	5.2% (4.1% to 6.4%)	1.4% (1.5% to 1.4%)	66.2 (66.2 to 66.2)
Cuba (UM)	89.3% (88.7% to 89.9%)	10.7% (10.8% to 10.5%)	0.0% (0.0% to 0.0%)	0.1% (0.1% to 0.1%)	10.2 (10.2 to 10.2)
Dominica (UM)	61.0% (59.8% to 61.7%)	31.9% (30.1% to 33.8%)	1.2% (0.8% to 1.6%)	5.9% (6.2% to 5.7%)	1.9 (1.9 to 1.9)
Dominican Republic (UM)	45.0% (44.0% to 46.0%)	43.7% (42.3% to 45.1%)	10.5% (8.4% to 12.8%)	0.7% (0.7% to 0.6%)	36.1 (36.1 to 36.1)
Ecuador (UM)	61.9% (61.4% to 62.6%)	30.9% (29.5% to 32.8%)	6.9% (5.3% to 8.8%)	0.2% (0.3% to 0.2%)	20.8 (20.8 to 20.8)
El Salvador (LM)	63.2% (63.5% to 62.4%)	28.6% (26.1% to 30.9%)	7.3% (5.3% to 9.6%)	0.9% (0.9% to 0.8%)	17.5 (17.5 to 17.5)
Grenada (UM)	38.1% (36.6% to 40.0%)	51.6% (50.4% to 52.5%)	3.8% (3.2% to 4.4%)	6.4% (6.8% to 6.1%)	4.0 (4.0 to 4.0)

Guatemala (UM)	37.6% (37.2% to 37.8%)	56.0% (55.9% to 56.2%)	5.5% (3.8% to 7.5%)	0.8% (0.9% to 0.8%)	42.3 (42.3 to 42.3)
Guyana (UM)	59.7% (60.1% to 59.3%)	33.4% (31.9% to 34.7%)	3.0% (2.1% to 4.3%)	3.8% (4.0% to 3.7%)	8.4 (8.4 to 8.4)
Haiti (LM)	15.2% (13.2% to 17.2%)	57.9% (56.0% to 59.6%)	6.8% (4.6% to 9.0%)	20.1% (21.5% to 18.7%)	149.1 (149.1 to 149.1)
Honduras (LM)	39.7% (39.3% to 40.4%)	53.4% (50.9% to 55.8%)	5.9% (4.4% to 7.5%)	1.1% (1.2% to 1.0%)	20.5 (20.5 to 20.5)
Jamaica (UM)	65.1% (64.0% to 66.4%)	16.3% (15.3% to 17.5%)	16.5% (16.6% to 16.3%)	2.1% (2.3% to 2.0%)	21.3 (21.3 to 21.3)
Mexico (UM)	49.6% (48.5% to 49.9%)	42.0% (40.7% to 43.1%)	8.4% (6.9% to 10.0%)	0.0% (0.0% to 0.0%)	19.4 (19.4 to 19.4)
Nicaragua (LM)	58.7% (58.2% to 59.5%)	32.6% (32.4% to 32.9%)	1.9% (1.6% to 2.2%)	6.8% (6.9% to 6.6%)	78.7 (78.7 to 78.7)
Panama (H)	65.6% (64.7% to 66.4%)	28.0% (28.3% to 27.7%)	6.4% (6.2% to 6.6%)	-	-
Paraguay (UM)	46.0% (44.2% to 47.1%)	41.8% (40.2% to 42.8%)	11.7% (9.5% to 14.4%)	0.5% (0.6% to 0.5%)	14.4 (14.4 to 14.4)
Peru (UM)	62.4% (62.0% to 63.4%)	28.3% (26.6% to 30.5%)	9.1% (7.0% to 11.5%)	0.3% (0.3% to 0.2%)	30.5 (30.5 to 30.5)
Puerto Rico (H)	74.5% (72.8% to 76.2%)	21.5% (18.8% to 24.4%)	4.0% (3.0% to 5.1%)	-	-
Saint Kitts and Nevis (H)	47.8% (47.3% to 48.5%)	47.4% (45.7% to 49.2%)	4.7% (4.4% to 5.0%)	-	-

Saint Lucia (UM)	45.3% (45.9% to 44.6%)	46.5% (44.7% to 48.2%)	5.2% (5.0% to 5.4%)	3.0% (3.1% to 2.9%)	2.8 (2.8 to 2.8)
Saint Vincent and the Grenadines (UM)	62.9% (61.8% to 64.2%)	27.2% (25.1% to 29.1%)	2.9% (2.0% to 3.9%)	7.0% (7.5% to 6.6%)	3.0 (3.0 to 3.0)
Suriname (UM)	70.3% (69.0% to 72.0%)	17.7% (17.2% to 18.1%)	11.0% (9.9% to 12.3%)	0.9% (1.0% to 0.9%)	2.8 (2.8 to 2.8)
The Bahamas (H)	52.6% (51.4% to 53.5%)	26.2% (26.1% to 26.1%)	21.2% (20.6% to 21.8%)	-	-
Trinidad and Tobago (H)	47.8% (46.7% to 48.6%)	45.4% (44.0% to 46.5%)	6.9% (6.6% to 7.1%)	-	-
Venezuela (UM)	48.2% (46.5% to 50.7%)	23.0% (21.0% to 24.6%)	28.7% (25.5% to 32.1%)	0.1% (0.1% to 0.0%)	3.6 (3.6 to 3.6)
Virgin Islands (H)	74.4% (71.2% to 76.6%)	21.9% (20.3% to 23.9%)	3.7% (2.9% to 4.7%)	-	-
North Africa and Middle East					
Afghanistan (L)	5.5% (4.9% to 6.1%)	81.3% (80.3% to 82.5%)	0.0% (0.0% to 0.0%)	13.2% (14.0% to 12.4%)	396.0 (396.0 to 396.0)
Algeria (LM)	65.7% (64.1% to 66.9%)	32.7% (29.9% to 34.9%)	1.5% (1.1% to 2.0%)	0.1% (0.1% to 0.1%)	11.3 (11.3 to 11.3)
Bahrain (H)	59.1% (57.9% to 60.0%)	29.7% (28.8% to 30.7%)	11.2% (10.6% to 11.8%)	-	-
Egypt (LM)	28.5% (26.5% to 30.8%)	60.9% (58.7% to 62.9%)	9.5% (7.2% to 11.9%)	1.1% (1.2% to 1.0%)	204.3 (204.3 to 204.3)
Iran (LM)	47.9% (46.6% to 49.3%)	37.8% (36.4% to 39.6%)	14.3% (12.9% to 15.9%)	0.0% (0.0% to 0.0%)	4.7 (4.7 to 4.7)

Iraq (UM)	45.0% (41.8% to 47.4%)	54.4% (52.7% to 55.5%)	0.0% (0.0% to 0.0%)	0.5% (0.6% to 0.5%)	54.1 (54.1 to 54.1)
Jordan (UM)	49.6% (47.4% to 50.6%)	31.4% (29.4% to 32.5%)	16.0% (13.2% to 18.5%)	3.0% (3.3% to 2.8%)	108.1 (108.1 to 108.1)
Kuwait (H)	87.3% (86.5% to 88.2%)	11.5% (11.2% to 11.7%)	1.2% (1.1% to 1.3%)	-	-
Lebanon (LM)	49.2% (48.0% to 50.4%)	33.5% (32.6% to 34.7%)	16.7% (16.4% to 17.0%)	0.7% (0.7% to 0.6%)	21.2 (21.2 to 21.2)
Libya (UM)	70.9% (67.8% to 73.4%)	25.9% (24.1% to 28.5%)	2.7% (2.0% to 3.4%)	0.5% (0.6% to 0.4%)	10.6 (10.6 to 10.6)
Morocco (LM)	41.0% (38.4% to 43.6%)	48.3% (45.8% to 50.2%)	9.2% (6.8% to 11.8%)	1.5% (1.6% to 1.4%)	103.5 (103.5 to 103.5)
Oman (H)	87.0% (86.2% to 88.0%)	6.4% (6.3% to 6.5%)	6.7% (6.6% to 6.7%)	-	-
Palestine (LM)	40.1% (39.5% to 41.8%)	41.8% (40.3% to 44.1%)	15.8% (13.1% to 18.8%)	2.3% (2.5% to 2.1%)	46.8 (46.8 to 46.8)
Qatar (H)	73.8% (71.5% to 75.3%)	11.5% (10.9% to 12.0%)	14.7% (14.3% to 14.7%)	-	-
Saudi Arabia (H)	70.3% (68.8% to 71.4%)	15.8% (15.6% to 15.9%)	13.9% (13.2% to 14.6%)	-	-
Sudan (L)	21.6% (20.9% to 21.7%)	66.5% (63.1% to 69.8%)	3.4% (2.5% to 4.4%)	8.6% (9.6% to 7.6%)	167.6 (167.6 to 167.6)
Syria (L)	45.1% (42.1% to 48.0%)	44.5% (40.9% to 48.3%)	2.4% (1.7% to 3.1%)	8.1% (9.2% to 7.2%)	43.5 (43.5 to 43.5)

Tunisia (LM)	56.7% (56.6% to 56.6%)	38.0% (36.4% to 39.5%)	4.8% (4.2% to 5.4%)	0.5% (0.5% to 0.5%)	16.6 (16.6 to 16.6)
Turkey (UM)	77.5% (76.3% to 77.9%)	16.9% (17.1% to 16.7%)	5.3% (3.9% to 7.1%)	0.3% (0.3% to 0.3%)	96.0 (96.0 to 96.0)
United Arab Emirates (H)	52.6% (51.5% to 53.8%)	12.5% (11.2% to 13.8%)	35.0% (34.9% to 35.1%)	-	-
Yemen (L)	12.1% (11.1% to 13.0%)	70.1% (65.2% to 74.0%)	0.9% (0.7% to 1.2%)	16.9% (19.4% to 14.7%)	185.2 (185.2 to 185.2)
South Asia					
Bangladesh (LM)	18.8% (17.5% to 20.1%)	73.2% (71.2% to 75.2%)	2.8% (2.0% to 3.7%)	5.2% (5.7% to 4.7%)	423.3 (423.3 to 423.3)
Bhutan (LM)	66.9% (64.6% to 69.1%)	14.4% (12.2% to 16.4%)	1.2% (0.9% to 1.7%)	17.5% (19.1% to 15.9%)	16.8 (16.8 to 16.8)
India (LM)	31.9% (31.2% to 32.5%)	55.6% (53.5% to 58.0%)	11.7% (9.5% to 14.2%)	0.7% (0.8% to 0.7%)	705.4 (705.4 to 705.4)
Nepal (LM)	23.9% (24.3% to 23.4%)	58.5% (53.2% to 62.9%)	6.4% (4.6% to 8.5%)	11.3% (12.7% to 10.0%)	177.2 (177.2 to 177.2)
Pakistan (LM)	32.0% (30.7% to 33.3%)	56.2% (51.4% to 60.8%)	6.6% (5.0% to 8.1%)	5.2% (6.2% to 4.4%)	496.0 (496.0 to 496.0)
Southeast Asia, East Asia, and Oceania					
American Samoa (UM)	76.8% (74.2% to 79.8%)	19.8% (17.9% to 21.8%)	3.3% (2.6% to 4.3%)	0.0% (0.0% to 0.0%)	0.0 (0.0 to 0.0)
Cambodia (LM)	25.0% (22.3% to 27.8%)	63.9% (63.9% to 64.0%)	3.7% (2.7% to 5.1%)	7.4% (7.9% to 6.9%)	127.4 (127.4 to 127.4)

China (UM)	56.2% (54.7% to 57.4%)	35.3% (34.7% to 35.6%)	8.4% (7.2% to 9.6%)	0.0% (0.0% to 0.0%)	326.5 (326.5 to 326.5)
Cook Islands (H)	84.5% (83.1% to 85.8%)	6.2% (6.2% to 6.3%)	0.6% (0.4% to 0.7%)	-	-
Federated States of Micronesia (LM)	81.4% (79.7% to 82.7%)	6.0% (5.8% to 6.3%)	0.0% (0.0% to 0.0%)	12.6% (13.6% to 11.7%)	2.3 (2.3 to 2.3)
Fiji (UM)	65.7% (64.4% to 67.4%)	13.4% (13.1% to 13.9%)	16.1% (14.8% to 17.3%)	4.8% (5.2% to 4.5%)	10.0 (10.0 to 10.0)
Guam (H)	66.4% (61.5% to 70.3%)	27.5% (24.8% to 29.6%)	6.1% (4.5% to 7.9%)	-	-
Indonesia (LM)	49.3% (48.5% to 49.9%)	34.4% (33.2% to 36.1%)	15.5% (14.6% to 16.4%)	0.9% (0.9% to 0.8%)	290.7 (290.7 to 290.7)
Kiribati (LM)	63.2% (61.8% to 64.1%)	20.0% (16.9% to 24.2%)	2.9% (2.6% to 3.3%)	13.8% (15.0% to 12.8%)	4.0 (4.0 to 4.0)
Laos (LM)	36.3% (31.5% to 40.6%)	45.2% (44.7% to 45.7%)	0.2% (0.1% to 0.3%)	18.3% (20.1% to 16.8%)	81.1 (81.1 to 81.1)
Malaysia (UM)	52.0% (51.0% to 53.5%)	34.2% (34.9% to 33.6%)	13.7% (11.7% to 16.4%)	0.0% (0.0% to 0.0%)	6.5 (6.5 to 6.5)
Maldives (UM)	77.5% (75.8% to 78.8%)	17.6% (16.6% to 18.3%)	4.1% (3.2% to 5.0%)	0.8% (0.9% to 0.7%)	3.8 (3.8 to 3.8)
Marshall Islands (UM)	50.5% (48.8% to 52.4%)	13.9% (13.1% to 14.4%)	4.8% (3.5% to 6.1%)	30.8% (32.2% to 29.4%)	11.4 (11.4 to 11.4)
Mauritius (UM)	47.0% (46.5% to 47.8%)	46.1% (45.5% to 46.7%)	6.9% (6.7% to 7.1%)	0.0% (0.0% to 0.0%)	0.0 (0.0 to 0.0)

Myanmar (LM)	16.7% (16.8% to 16.5%)	77.6% (74.4% to 80.0%)	0.0% (0.0% to 0.0%)	5.7% (6.6% to 4.9%)	201.6 (201.6 to 201.6)
Nauru (H)	78.7% (78.2% to 79.9%)	14.2% (12.0% to 16.8%)	7.0% (6.3% to 7.8%)	-	-
Niue (H)	51.3% (45.9% to 56.1%)	8.8% (7.5% to 10.2%)	2.3% (1.7% to 3.2%)	-	-
North Korea (L)	58.5% (55.0% to 60.7%)	39.3% (35.8% to 44.3%)	2.0% (1.5% to 2.4%)	0.2% (0.3% to 0.2%)	2.4 (2.4 to 2.4)
Northern Mariana Islands (H)	63.8% (59.8% to 66.6%)	30.8% (27.1% to 34.7%)	5.4% (4.3% to 7.1%)	-	-
Palau (UM)	62.6% (61.1% to 64.2%)	15.4% (15.3% to 15.5%)	22.0% (21.6% to 22.5%)	0.0% (0.0% to 0.0%)	0.0 (0.0 to 0.0)
Papua New Guinea (LM)	63.9% (62.0% to 66.2%)	8.8% (8.1% to 9.6%)	0.0% (0.0% to 0.0%)	27.2% (28.9% to 25.6%)	192.4 (192.4 to 192.4)
Philippines (LM)	38.9% (36.9% to 41.1%)	48.4% (46.0% to 51.2%)	11.4% (8.9% to 14.0%)	1.3% (1.5% to 1.2%)	228.4 (228.4 to 228.4)
Samoa (LM)	68.8% (67.3% to 70.2%)	10.0% (8.5% to 11.7%)	0.8% (0.5% to 1.0%)	20.5% (21.9% to 19.1%)	11.7 (11.7 to 11.7)
Seychelles (H)	73.3% (73.8% to 72.8%)	25.0% (23.1% to 27.0%)	1.7% (1.4% to 2.0%)	-	-
Solomon Islands (LM)	69.5% (67.2% to 71.5%)	3.2% (2.9% to 3.5%)	0.0% (0.0% to 0.0%)	27.3% (29.4% to 25.2%)	20.5 (20.5 to 20.5)
Sri Lanka (LM)	45.7% (45.2% to 46.4%)	46.1% (42.2% to 49.4%)	6.3% (4.5% to 8.5%)	1.9% (2.1% to 1.8%)	65.5 (65.5 to 65.5)

Taiwan (province of China) (H)	76.9% (78.2% to 75.2%)	19.4% (15.9% to 23.3%)	3.7% (2.6% to 5.1%)	-	-
Thailand (UM)	70.9% (70.9% to 70.9%)	9.6% (8.7% to 10.5%)	19.3% (19.0% to 19.7%)	0.2% (0.2% to 0.2%)	43.4 (43.4 to 43.4)
Timor-Leste (LM)	68.4% (65.8% to 70.9%)	9.6% (8.3% to 11.1%)	5.5% (4.0% to 7.4%)	16.5% (18.3% to 14.7%)	26.0 (26.0 to 26.0)
Tokelau (UM)	36.1% (32.1% to 40.5%)	18.6% (15.5% to 22.0%)	3.1% (2.2% to 4.1%)	42.2% (46.5% to 38.4%)	1.2 (1.2 to 1.2)
Tonga (UM)	67.1% (67.0% to 67.0%)	12.0% (9.9% to 14.4%)	4.2% (3.3% to 5.3%)	16.6% (17.4% to 15.9%)	3.7 (3.7 to 3.7)
Tuvalu (UM)	77.9% (77.5% to 78.5%)	8.7% (6.9% to 10.6%)	0.0% (0.0% to 0.0%)	13.4% (14.0% to 12.9%)	1.6 (1.6 to 1.6)
Vanuatu (LM)	62.4% (58.2% to 66.6%)	9.5% (8.9% to 10.2%)	4.0% (3.1% to 5.2%)	24.1% (26.8% to 21.6%)	7.6 (7.6 to 7.6)
Vietnam (LM)	44.3% (42.9% to 46.4%)	43.9% (41.0% to 46.8%)	10.8% (8.0% to 13.9%)	0.9% (1.1% to 0.9%)	166.9 (166.9 to 166.9)
Sub-Saharan Africa					
Angola (LM)	41.9% (38.9% to 43.9%)	35.6% (31.8% to 38.4%)	17.7% (13.7% to 22.2%)	4.8% (5.4% to 4.2%)	102.7 (102.7 to 102.7)
Benin (LM)	20.9% (19.2% to 23.1%)	41.6% (36.6% to 47.9%)	5.3% (3.8% to 7.0%)	32.2% (36.1% to 28.8%)	133.7 (133.7 to 133.7)
Botswana (UM)	77.4% (76.9% to 77.6%)	3.0% (2.9% to 3.1%)	13.3% (10.9% to 15.7%)	6.4% (6.8% to 6.0%)	74.8 (74.8 to 74.8)
Burkina Faso (L)	39.1% (35.8% to 42.4%)	30.4% (30.4% to 30.5%)	6.6% (4.6% to 9.0%)	23.9% (25.5% to 22.5%)	253.8 (253.8 to 253.8)

Burundi (L)	21.9% (20.0% to 23.6%)	16.7% (15.4% to 18.2%)	11.5% (8.6% to 14.8%)	49.9% (52.5% to 46.8%)	194.6 (194.6 to 194.6)
Cameroon (LM)	5.2% (4.7% to 5.8%)	72.3% (71.6% to 73.1%)	9.2% (7.3% to 11.2%)	13.3% (14.0% to 12.6%)	200.8 (200.8 to 200.8)
Cape Verde (LM)	54.8% (51.6% to 57.7%)	21.8% (22.1% to 21.5%)	2.1% (1.6% to 2.6%)	21.3% (22.7% to 19.8%)	27.0 (27.0 to 27.0)
Central African Republic (L)	8.3% (7.4% to 9.4%)	50.0% (48.0% to 51.9%)	0.8% (0.5% to 1.1%)	40.8% (42.9% to 39.1%)	94.2 (94.2 to 94.2)
Chad (L)	16.2% (15.1% to 17.3%)	56.8% (54.8% to 59.0%)	5.0% (3.9% to 6.4%)	22.0% (23.3% to 20.8%)	122.6 (122.6 to 122.6)
Comoros (LM)	12.4% (10.7% to 14.3%)	53.3% (52.9% to 54.2%)	3.6% (2.6% to 5.0%)	30.7% (31.8% to 29.8%)	23.8 (23.8 to 23.8)
Congo (Brazzaville) (LM)	36.0% (34.6% to 36.9%)	43.7% (39.5% to 48.1%)	7.2% (5.1% to 9.4%)	13.2% (14.7% to 11.7%)	41.2 (41.2 to 41.2)
C√¥te d'Ivoire (LM)	29.3% (31.0% to 27.8%)	38.9% (36.1% to 41.0%)	20.1% (14.5% to 26.7%)	11.7% (12.9% to 10.6%)	241.8 (241.8 to 241.8)
DR Congo (L)	13.1% (11.6% to 14.5%)	34.8% (32.8% to 36.5%)	6.5% (5.1% to 8.5%)	45.6% (47.8% to 43.5%)	1005.4 (1005.4 to 1005.4)
Djibouti (LM)	46.6% (45.9% to 47.2%)	21.5% (19.7% to 23.4%)	1.5% (1.1% to 2.0%)	30.4% (31.3% to 29.6%)	22.1 (22.1 to 22.1)
Equatorial Guinea (UM)	18.9% (18.8% to 19.0%)	74.7% (71.7% to 77.2%)	2.4% (2.0% to 2.9%)	3.9% (4.3% to 3.6%)	17.3 (17.3 to 17.3)
Eritrea (L)	16.9% (15.0% to 18.7%)	40.9% (36.1% to 47.3%)	0.0% (0.0% to 0.0%)	42.2% (46.6% to 37.5%)	43.4 (43.4 to 43.4)

eSwatini (LM)	49.0% (48.7% to 49.1%)	10.3% (8.7% to 12.3%)	13.2% (10.4% to 16.1%)	27.5% (28.7% to 26.1%)	88.4 (88.4 to 88.4)
Ethiopia (L)	25.9% (24.2% to 27.9%)	39.0% (37.5% to 40.3%)	7.2% (5.2% to 9.4%)	27.9% (29.7% to 26.2%)	740.3 (740.3 to 740.3)
Gabon (UM)	55.4% (53.8% to 56.3%)	21.0% (18.6% to 23.6%)	14.4% (14.6% to 14.2%)	9.2% (9.7% to 8.6%)	54.4 (54.4 to 54.4)
Ghana (LM)	39.8% (37.9% to 42.0%)	37.3% (35.3% to 39.5%)	12.4% (12.1% to 12.8%)	10.5% (11.2% to 9.8%)	255.2 (255.2 to 255.2)
Guinea (L)	16.2% (14.9% to 17.4%)	59.5% (54.2% to 63.9%)	8.2% (6.1% to 10.7%)	16.1% (18.5% to 13.9%)	106.2 (106.2 to 106.2)
Guinea-Bissau (L)	6.2% (5.3% to 7.1%)	57.2% (54.3% to 59.6%)	2.9% (2.1% to 3.9%)	33.7% (36.2% to 31.5%)	44.9 (44.9 to 44.9)
Kenya (LM)	44.6% (40.3% to 48.6%)	24.5% (22.1% to 27.0%)	11.8% (10.0% to 13.6%)	19.2% (21.1% to 17.4%)	845.9 (845.9 to 845.9)
Lesotho (LM)	53.2% (51.2% to 55.0%)	16.0% (15.9% to 16.0%)	0.5% (0.4% to 0.7%)	30.3% (31.6% to 29.1%)	75.0 (75.0 to 75.0)
Liberia (L)	12.3% (10.8% to 13.9%)	45.5% (42.9% to 48.3%)	5.6% (3.9% to 8.1%)	36.6% (39.1% to 34.5%)	109.3 (109.3 to 109.3)
Madagascar (L)	41.9% (41.1% to 42.6%)	31.7% (26.9% to 36.2%)	7.1% (4.9% to 9.4%)	19.3% (21.4% to 17.3%)	107.5 (107.5 to 107.5)
Malawi (L)	24.7% (23.0% to 26.2%)	12.8% (11.7% to 13.9%)	5.4% (4.0% to 7.1%)	57.2% (59.0% to 55.3%)	462.2 (462.2 to 462.2)
Mali (L)	33.8% (32.6% to 34.6%)	33.2% (29.9% to 36.3%)	1.8% (1.2% to 2.4%)	31.3% (33.4% to 29.3%)	227.1 (227.1 to 227.1)

Mauritania (LM)	36.7% (35.4% to 37.8%)	44.7% (44.0% to 45.3%)	5.5% (3.9% to 8.1%)	13.1% (13.7% to 12.5%)	39.3 (39.3 to 39.3)
Mozambique (L)	29.4% (25.6% to 33.5%)	13.0% (10.8% to 15.1%)	8.6% (6.1% to 11.6%)	49.0% (53.2% to 45.0%)	445.5 (445.5 to 445.5)
Namibia (UM)	46.5% (45.6% to 47.6%)	8.2% (7.1% to 9.7%)	39.8% (37.8% to 41.7%)	5.5% (5.8% to 5.1%)	63.2 (63.2 to 63.2)
Niger (L)	31.7% (29.4% to 34.0%)	43.1% (42.3% to 43.7%)	3.4% (2.3% to 4.7%)	21.8% (22.8% to 20.9%)	189.3 (189.3 to 189.3)
Nigeria (LM)	16.2% (14.4% to 18.1%)	76.3% (75.1% to 78.0%)	0.9% (0.6% to 1.2%)	6.6% (7.3% to 6.1%)	905.0 (905.0 to 905.0)
Rwanda (L)	36.2% (32.4% to 40.1%)	10.9% (10.6% to 11.2%)	14.4% (11.9% to 16.7%)	38.5% (41.6% to 35.4%)	274.3 (274.3 to 274.3)
Senegal (LM)	24.5% (21.9% to 26.7%)	50.2% (45.7% to 54.2%)	6.2% (4.4% to 8.0%)	19.0% (21.2% to 16.9%)	205.2 (205.2 to 205.2)
Sierra Leone (L)	12.1% (10.3% to 14.1%)	53.7% (51.0% to 56.0%)	1.4% (1.0% to 1.9%)	32.9% (35.1% to 31.0%)	125.0 (125.0 to 125.0)
Somalia (L)	7.7% (6.5% to 9.0%)	26.0% (20.9% to 31.0%)	0.2% (0.1% to 0.2%)	66.1% (70.7% to 61.8%)	89.5 (89.5 to 89.5)
South Africa (UM)	58.2% (58.7% to 57.7%)	5.7% (5.6% to 5.8%)	34.7% (32.6% to 36.9%)	1.4% (1.5% to 1.4%)	500.1 (500.1 to 500.1)
South Sudan (L)	10.2% (8.7% to 11.6%)	16.5% (14.1% to 18.9%)	3.7% (2.5% to 5.4%)	69.6% (72.3% to 67.0%)	274.9 (274.9 to 274.9)
S√£o Tom√© and Pr√≠ncipe (LM)	31.8% (28.0% to 35.2%)	11.9% (10.1% to 14.0%)	1.2% (0.8% to 1.6%)	55.2% (58.8% to 51.7%)	21.7 (21.7 to 21.7)

Tanzania (LM)	42.8% (41.1% to 44.4%)	22.7% (21.8% to 23.8%)	1.0% (0.6% to 1.4%)	33.5% (34.9% to 32.1%)	789.0 (789.0 to 789.0)
The Gambia (L)	21.9% (18.8% to 25.1%)	17.8% (15.8% to 19.8%)	3.3% (2.2% to 4.6%)	57.1% (60.3% to 54.0%)	54.4 (54.4 to 54.4)
Togo (L)	15.1% (13.3% to 17.2%)	63.2% (61.2% to 64.4%)	8.5% (6.2% to 11.4%)	13.2% (14.2% to 12.2%)	62.3 (62.3 to 62.3)
Uganda (L)	17.0% (15.5% to 18.5%)	36.3% (31.8% to 40.9%)	3.8% (2.7% to 5.0%)	43.0% (46.4% to 39.6%)	728.6 (728.6 to 728.6)
Zambia (L)	38.0% (34.1% to 43.3%)	10.0% (8.4% to 11.6%)	5.9% (4.0% to 7.8%)	46.1% (49.9% to 41.9%)	520.1 (520.1 to 520.1)
Zimbabwe (LM)	21.4% (19.3% to 23.2%)	20.6% (17.5% to 23.7%)	21.0% (16.6% to 25.7%)	37.0% (40.1% to 34.0%)	304.5 (304.5 to 304.5)

S1.2 Estimating domestic health spending: 1995–2019

We extracted data on gross domestic product (GDP) per capita from five leading sources of these estimates (World Bank, International Monetary Fund [IMF], United Nations, Penn World Tables, and The Maddison Project) and built from methods described by James and colleagues to generate a single series of GDP per capita using Gaussian processes.[3-7] This method incorporated data from all five GDP series and also propagated uncertainty through the estimates.[8] The resulting series spans 204 countries from 1950 to 2021, and larger uncertainty intervals highlight countries where the input data from the five sources was discordant or estimates were missing.

We extracted data from the WHO's Global Health Expenditure Database (GHED) on government domestic revenue transfers allocated for health, compulsory prepayment, voluntary prepayment, social insurance contributions, and other domestic revenue from households, corporations, and non-profit institutions serving households.[9] Data from GHED excludes spending on major investments such as hospital construction, health worker education and training, and research and development. Countries report these data and the data source to WHO; when possible, these are based on National Health Accounts, but alternative sources are used in many cases. Health spending estimates were extracted in current national currency units, deflated to 2021 national currency units, and exchanged to 2021 US dollars. Deflator series and exchanges rates used were based on those reported in the IMF World Economic Outlook.[5]

We estimated domestic government spending on health by aggregating transfers from government domestic revenue for health purposes, compulsory prepayment, and social insurance contributions. Then, to estimate domestic prepaid private health spending, we aggregated voluntary prepayment, other domestic revenues from non-profit institutions serving the household, and other domestic revenues from corporations. All payments by households other than social insurance contributions were designated as out-of-pocket spending. To generate domestic health spending estimates in purchasing-power parity-adjusted (PPP) dollars, we multiplied the health spending fractions by GDP per capita measured in 2021 PPP dollars.

The extracted data were evaluated for quality. Some of the extracted data points were not tied to an underlying data source and were estimated using a diverse set or poorly defined methods. Furthermore, for a given country, some data varied substantially across time. To overcome these concerns, we used a spatiotemporal Gaussian process regression model to estimate health spending across time, country, and spending category.[10] To prevent data with insufficiently described estimation methods or without data source identification from substantially influencing our model estimation, each data point was assessed and assigned a weight between one and five using the point-specific metadata provided in the GHED. We based weights on metadata completeness, documented source information, and documented methods for estimation. The distribution of data points assigned to each of the five data quality scores, by country income group, is presented in the appendix. Our guidelines for assessing the metadata, a detailed description of how the weights were created, and additional information about the spatiotemporal Gaussian process regression model can also be found in the appendix.

S1.3 Estimating development assistance for health: 1990–2020

To track DAH, we relied on revenue data in financial statements, annual reports, budget documents, and project disbursement records reported by international development agencies such as the Organisation for Economic Co-operation and Development (OECD) Creditor Reporting System, the World Bank, the Global Fund, and major philanthropic entities like the Bill & Melinda Gates Foundation, Susan Thompson Buffet Foundation, and Rotary International.[11-20] We used budget and commitment data to generate estimates for recent years for which disbursement data were not yet available; for most channels data extends to 2019 or 2020 so that at most two years of estimation were needed.

Our DAH estimates tracked disbursements from the originating donor (called the source); to the development agency responsible for disbursing the funds to the recipient country (called the channel); and to the recipient country. We used revenue and disbursement data to count the transfers between development agencies only once to limit double counting. In addition to reporting the source, channel, and recipient of DAH, we disaggregated spending into nine major health focus areas (as well as more detailed programme areas) by searching project titles and descriptions for keywords, as well as using other indicators in the data. Health focus areas included HIV/AIDS; malaria; tuberculosis; reproductive and maternal health; newborn and child health; non-communicable diseases; other infectious diseases; sector-wide approaches and health system strengthening; and other. The "Other" category captured projects such as general support for a conference on the Sustainable Development Goals that were not allocated to any of the eight specific health focus areas; remaining funds for which no project descriptions were available were classified as "unallocable." The list of keywords used to isolate relevant health projects for each of the health focus areas are included in the supplemental appendix Table S3.4.

While the majority of the methods used for tracking DAH have been described previously, the current estimates of DAH incorporated improvements in methodology such as leveraging additional project level descriptions from the Creditor Reporting System for the allocation of disbursements channeled through NGOs and ongoing refinement of our keyword search list.[21-25] For all DAH tracking, we include funds that

were transferred through major development agencies included in our research, as well as private foundation and non-governmental agencies for whom we have data. This definition of DAH excludes spending on basic bench science which may have down-stream effects on the development of new drugs.

Spending type	Main sources of data	Data type
Development Assistance	• Organisation for Economic Co-operation and Development (OECD) Creditor Reporting System<br>• the World Bank,<br>• the Global Fund<br>• Bill & Melinda Gates Foundation,<br>• Susan Thompson Buffet Foundation, and<br>• Rotary International	• Financial statements<br>• Annual reports<br>• Budget documents<br>• Project disbursement records
Total Domestic Health Spending (Government Spending, Out-of-pocket Spending, Prepaid Private Spending)	• World Bank<br>• International Monetary Fund (IMF)<br>• United Nations<br>• Penn World Tables<br>• The Maddison Project<br>• WHO's Global Health Expenditure Database (GHED)	• Gross domestic product (GDP) per capita<br>• Government domestic revenue transfers allocated for:<br>○ Health<br>○ Compulsory prepayment<br>○ Voluntary prepayment<br>○ Social insurance contributions<br>○ Other domestic revenue from households, corporations, and non-profit institutions serving households

S1.4 Forecasting GDP per capita in the short term to reflect the impacts of COVID-19

In light of the COVID-19 pandemic, we estimated GDP per capita in 2022–2027 using a similar set of methods as described in Section S1.2. We obtained real GDP growth rates from several sources which provide short-term economic forecasts, including the World Bank, International Monetary Fund [IMF], and United Nations.[5, 30-32] We applied these growth rates to each respective data source's GDP estimates in 2021 (expressed in 2021 inflation-adjusted dollars) to estimate GDP per capita in 2022–2027. We then generated a single series of GDP per capita using Gaussian processes, incorporating data from all three GDP series and propagating uncertainty through the estimates. For 2025–2027, we used a weighted average of forecasts generated by the Gaussian process regression, and forecasts generated for these years by our long-term forecasting methods as described in Section 4.

S1.5 Reporting and uncertainty analysis

All inflation-adjusted health spending estimates are reported using 2021 prices. Future values are not discounted. We report health spending per capita in US dollars and purchasing-power parity-adjusted dollars and as a fraction of GDP. When not otherwise indicated, estimates are reported in 2021 US dollars. We report country spending estimates using 2019 Global Burden of Disease super-regions and 2020 World Bank income groups, regardless of whether a country changed, or is projected to change, income groups during the study period.[28,29] GBD regions can be seen in Figure S1.5 below. Rates were calculated to reflect each group, rather than the average of countries within the group, such that spending per capita estimates for an income group or region more heavily reflect rates in more populous countries. The uncertainty interval around each estimate was computed using the 2.5th and 97.5th percentiles of the 500 draws. All analyses of domestic health spending were performed using R (version 3.6), Stata (version 15.1), and Python (version 3.7). All analyses of development assistance for health were performed using R (versions 3.6 and 4.0), Stata (version 15.1), and Python (versions 3.6 and 3.10). Table S1.5 presents all packages used for the analyses in this paper.

Table S1.4 Programming packages used for analyses

Packages used within R (versions 3.6 and 4.0)					Packages used within Python (versions 3.6, 3.7, 3.10)	Packages used within Stata (version 15.1)
abind	dplyr	highcharter	readstata13	TMB	bs4.BeautifulSoup	egenmore
AFModel	DT	influence.ME	readxl	tools	csv	findname
argparse	feather	iterators	reshape2	wbstats	datetime	grc1leg
arm	foreach	lme4	reticulate	xlsx	joblib	xfill
bit64	forecast	lmtest	rhdf5	yaml	json	
boot	foreign	MASS	rjson	zip	numpy	
Cairo	frontier	matrix	RMySQL	zoo	os	
car	GauProMod	matrixStats	scales		pandas	
chron	ggalluvial	micEcon	shiny		requests	

compiler	ggplot2	openxlsx	shinythemes		selenium.webdriver	
crayon	ggpubr	pacman	stringi		sklearn	
cowplot	ggrepel	parallel	stringr		string	
data.table	grid	plotly	testthat		sys	
devtools	gridExtra	plyr	tidyr		time	
doParallel	haven	RColorBrewer	tidyverse		xarray	

Figure S1.5 Global Burden of Disease super-regions and regions (source www.healthdata.org)

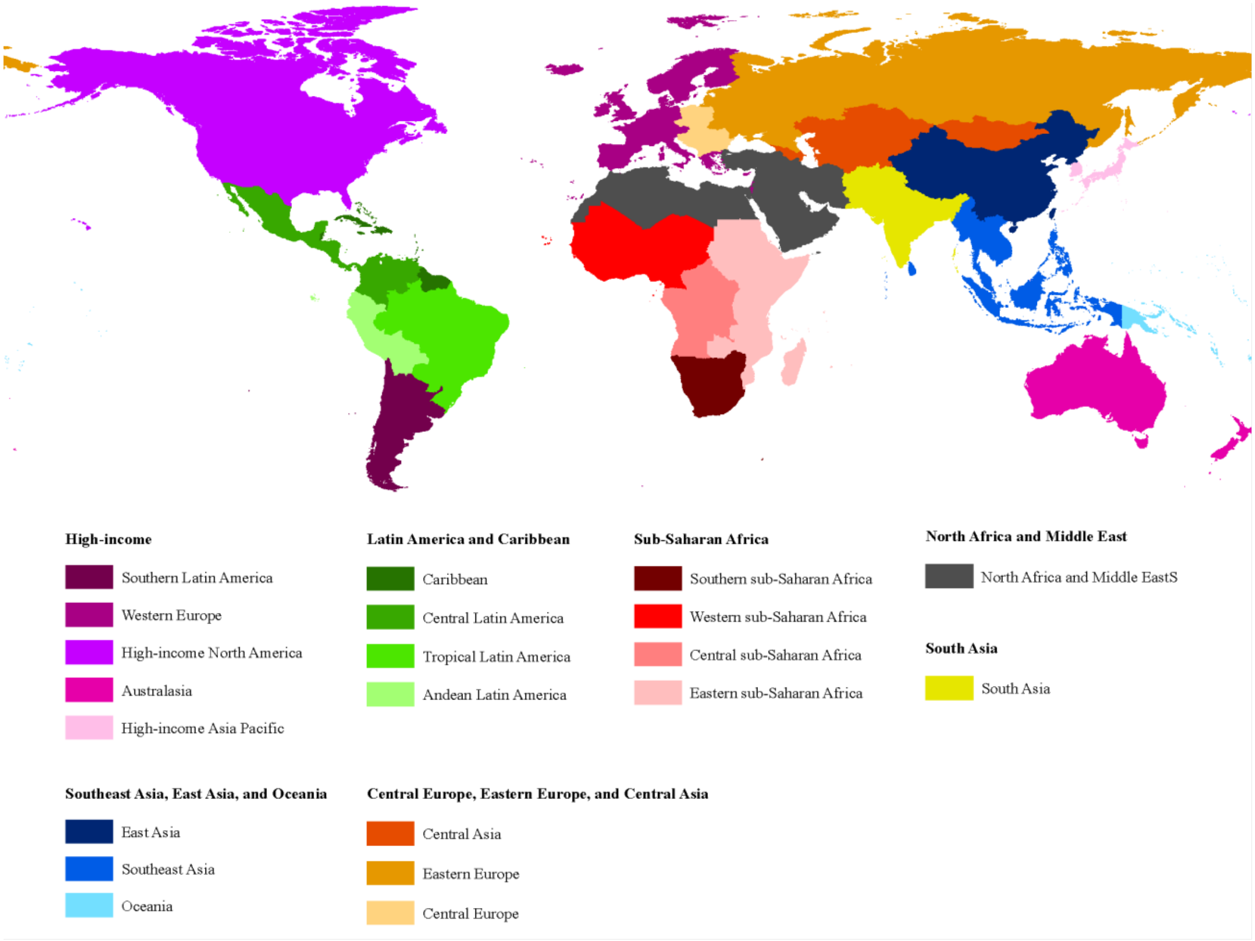

References

1WHO | Global Health Expenditure Database. WHO. http://www.who.int/health-accounts/ghed/en/ (accessed Feb 4, 2022).

2OECD, Eurostat and World Health Organization. A System of Health Accounts 2011: Revised edition. Paris: OECD Publishing, 2017 https://read.oecd-ilibrary.org/social-issues-migration-health/a-system-of-health-accounts-2011_9789264270985-en (accessed March 7, 2019).

3The Database | Penn World Table version 10.0. Groningen Growth and Development Centre: Faculty of Economics and Business. https://www.rug.nl/ggdc/productivity/pwt/ (accessed Feb 4, 2022).

4DataBank | The World Bank. The World Bank. http://databank.worldbank.org/data/home.aspx (accessed Feb 4, 2022).

5International Monetary Fund. World Economic Outlook, April 2022: War Sets Back the Global Recovery. International Monetary Fund, 2022. https://www.imf.org/en/Publications/WEO/Issues/2022/04/19/world-economic-outlook-april-2022 (accessed April June 8, 2022).

6Maddison Project Database 2020. Groningen Growth and Development Centre: Faculty of Economics and Business. https://www.rug.nl/ggdc/historicaldevelopment/maddison/releases/maddison-project-database-20 (accessed Feb 4, 2022).

7National Accounts Main Aggregates Database. United Nations Statistics Division - National Health Accounts. https://unstats.un.org/unsd/snaama/ (accessed Feb 4, 2022).

8James SL, Gubbins P, Murray CJ, Gakidou E. Developing a comprehensive time series of GDP per capita for 210 countries from 1950 to 2015. *Population Health Metrics* 2012; **10**: 12.

9Global Health Expenditure Database. World Health Organization. http://apps.who.int/nha/database (accessed Feb 4, 2022).

10GBD 2015 Risk Factors Collaborators. Global, regional, and national comparative risk assessment of 79 behavioural, environmental and occupational, and metabolic risks or clusters of risks, 1990–2015: a systematic analysis for the Global Burden of Disease Study 2015. The Lancet 2016; 388: 1659–724.

11Find a Grant. https://data-service.theglobalfund.org/ (accessed Oct 26, 2021).

12OECD Statistics. https://stats.oecd.org/ (accessed Dec 22, 2018).

13Malaria Operational Plans (MOPs) | PMI. https://www.pmi.gov/resource-library/mops/ (accessed Dec 22, 2018).

14Key figures: donor contributions & pledges. https://www.gavi.org/investing/funding/donor-profiles/ (accessed Jan 13, 2022).

15Trustees reports and financial statements - International Finance Facility for Immunisation. https://iffim.org/investor-centre/trustee-reports-financial-statements (accessed Dec 22, 2018).

16Financial reports. https://www.gavi.org/news-resources/document-library/financial-reports (accessed Jan 11, 2022).

17Disbursements and commitments. https://www.gavi.org/results/disbursements/ (accessed Dec 22, 2018).

18 UNAIDS. PCB Archive. http://www.unaids.org/en/aboutunaids/unaidsprogrammecoordinatingboard/pcbmeetingarchive (accessed Jan 26, 2022).

19 Bill and Melinda Gates Foundation Financial statements https://www.gatesfoundation.org/about/financials/annual-reports (accessed Oct 26, 2021).

20 GB | Governing Body Documentation. http://apps.who.int/gb/ (accessed Dec 7, 2022).

21Ravishankar N, Gubbins P, Cooley RJ, et al. Financing of global health: tracking development assistance for health from 1990 to 2007. The Lancet 2009; 373: 2113–24.

22Leach-Kemon K, Chou DP, Schneider MT, et al. The Global Financial Crisis Has Led To A Slowdown In Growth Of Funding To Improve Health In Many Developing Countries. Health Affairs 2012; 31: 228–35.

23Dieleman JL, Graves C, Johnson E, et al. Sources and Focus of Health Development Assistance, 1990–2014. JAMA 2015; 313: 2359–68.

24Dieleman JL, Graves CM, Templin T, et al. Global health development assistance remained steady in 2013 but did not align with recipients' disease burden. Health Aff (Millwood) 2014; 33: 878–86.

25Dieleman JL, Templin T, Sadat N, et al. National spending on health by source for 184 countries between 2013 and 2040. The Lancet 2016; 387: 2521–35.

26Micah AE, Zhao Y, Chen CS, et al. Tracking development assistance for health from China, 2000–2018. BMJ Global Health under review.

27Das Gupta P. Standardization and Decompostion of Rates: A User's Manual. U.S. Bureau of the Census, 1993 https://www.census.gov/content/dam/Census/library/publications/1993/demo/p23-186.pdf (accessed Dec 21, 2018).

28World Bank Country and Lending Groups – World Bank Data Help Desk. The World Bank. https://datahelpdesk.worldbank.org/knowledgebase/articles/906519-world-bank-country-and-lending-groups (accessed Oct 11, 2021).

29GBD 2019 Demographics Collaborators. Global age-sex-specific fertility, mortality, healthy life expectancy (HALE), and population estimates in 204 countries and territories, 1950–2019: a comprehensive demographic analysis for the Global Burden of Disease Study 2019. The Lancet. 17 October 2020. doi:10.1016/S0140-6736(20)30977-6.

30Global Economic Prospects, June 2022. The World Bank. The World Bank. https://www.worldbank.org/en/publication/global-economic-prospects (accessed June 8, 2022).

31World Economic Situation and Prospects Report 2022. United Nations Department of Economic and Social Affairs. https://www.un.org/development/desa/dpad/publication/world-economic-situation-and-prospects-2022/ (accessed Feb 4, 2022).

32 World Economic Situation and Prospects as of mid-2022. United Nations Department of Economic and Social Affairs. https://www.un.org/development/desa/dpad/publication/world-economic-situation-and-prospects-as-of-mid-2022/ (accessed June 8, 2022).

SECTION 2. TRACKING TOTAL HEALTH SPENDING AND ITS COMPONENTS

List of Tables and Figures

Table S2.1 Rules for assigning weight values to metadata

Table S2.2 Countries and income groups, number of assigned weights

Figure S2.1 Comparison of WHO GHED extracted and IHME ST-GPR THE in per capita space, 2000-2019

Figure S2.2 Comparison of WHO GHED extracted and IHME ST-GPR THE in per GDP space, 2000-2019

Figure S2.3 Currency conversion process diagram

S 2.1 Overview of GHED data cleaning process

We used Global Health Expenditure Database (GHED) data from the World Health Organization (WHO) to generate our estimates.1 From the GHED, we extracted "Current health expenditure by revenues of health care financing schemes" for total health expenditure (THE), "Other revenues from households n.e.c" for out-of-pocket (OOP), "Gross Domestic Product" for GDP. We summed "Social insurance contributions" and "Transfers from government domestic revenue (allocated to health purposes)" for government health spending (GHE). We summed "Voluntary prepayment", "Other revenues from corporations n.e.c.", "Compulsory prepayment (Other, and unspecified, than FS.3)", and "Other revenues from NPISH n.e.c." for pre-paid private (PPP).

To ensure we used the best possible data from the GHED, we evaluated the metadata also provided by GHED to establish the reliability of the data. To do so, we downloaded the metadata from the GHED website for each data point for the five indicators. We used the metadata to decide how each given data point should be weighted, from 0 to 5, with 0 meaning drop, and 1 through 5 meaning keep and treated these weights as inverse variance weights.

To assign the weights, we established guidelines for the metadata that informed how the underlying data points should be weighted. We gave priority to factors such as complete, documented source information and penalized factors such as having been derived or estimated. Table S2.1 below describes the guidelines we created; any metadata that did not meet any of the disqualifying factors were given a value of 5 to reflect highest reliability.

In order to assign weights to the latest 2021 GHED estimates, we trained a linear SVM (Support Vector Machine) model supported by an NLP (Natural Language Processing) pipeline that learned from the weights that were manually assigned (based on the aforementioned guidelines) to metadata downloaded in the year 2017. A linear SVM model using the Sklearn library was chosen since its strengths aligned well with our task and the model performed well overall. The NLP pipeline consisted of a count vectorizer to transform the text metadata matrix into a matrix of token counts that can easily be interpreted by any machine learning algorithm. The count vectorizer also helped us bring in the possibility of simple words as well as combinations of simple words (i.e., word n-grams) to help with predicting the optimum reliability weight for each data point. The final model was tuned to achieve close resemblance for predicted weights when juxtaposed against the manually assigned weights for data points downloaded in the year 2017; and then used to predict weights for data points downloaded in 2021.

We used the four primary metadata variables from the GHED database: data type, method of estimation, comments, and sources. We applied the guidelines to each unique set of metadata across these four variables. For a subset of data points, the metadata indicated that the data point was the sum of other data points. In these cases, if the indicator was a sub-indicator of GHES, PPP, or OOP, we assigned the data point a value of 2 to reflect that even though we could not determine if the sub-components existed, as they are not reported in GHED, we did not feel that being a sum warranted dropping the data. We assigned the summed GHES, PPP, and OOP indicators the lowest value of its sub-indicators. If the summed data point was THE, however, we assigned the data point the lowest value of its sub-components, the summed GHES, PPP, and OOP indicators.

After designating each of the 5,079 unique sets of metadata a value weight, we applied these weights to the underlying data points. In total, we had 33,344 data points, as multiple data points shared the same unique set of metadata. Once the weights were applied to the data, we reassigned all high-income country data points that were a 0 based on the metadata to 3. We made this change to reflect that high-income countries have higher quality data and thus should not be dropped, but should also not be given the highest weight value. The high-income classification comes from the World Bank.[2] For all countries across all years of data their relative weighting scores as well as the percent completeness of underlying cited data can be seen in Table S2.2.

Each component of health expenditure was aggregated for all component-year-country combinations by taking component values (downloaded in current national unit); dividing by GHED reported GDP for the corresponding year-country (also in current national unit) to convert each component into its fraction of GDP; and finally multiplying those fractions by IHME GDP series measured in USD 2021. (Note: though we harvested and cleaned metadata for THE and GDP, we do not model THE (we model GHE, PPP, OOP and then aggregate) or use the WHO's GDP data since we have our own methods with multiple sources for determination of our value).

Each component of health expenditure was then converted into three spending metrics for the time period between 2000-2019, measured in purchasing power parity (PPP): health spending per capita ($), annualized rate of change in health spending per capita 2000-2019 (%), and annualized rate of change in health spending 2000-2019 (%). Then, each component of health expenditure was aggregated and converted into six spending metrics for the time period between 2020-2050, measured in PPP: health spending per capita ($), annualized rate of change in health spending per capita 2020-2050 (%), annualized rate of change in health spending 2020-2050 (%), Government health spending per capita ($), difference between GHEpc (government health expenditure per capita) reference scenario and greater scenario 1, 2050 ($), and difference between GHEpc reference scenario and greater scenario 2, 2050 ($).

Formula for calculating AROC (Annualized rate of change):

If you apply this growth rate n times (where n is the number of steps from starting to ending year) to the value in the starting year, you will get the value in the ending year. For example –

$$\underset{1990\rightarrow 2019}{\text{AROC}} = \left(\frac{value\ in\ 2019}{value\ in\ 1990}\right)^{\frac{1}{2019-1990}} - 1$$

Table S2.1 Rules for assigning weight values to metadata

Data type	Methods of estimation	Sources and/or Comments	Weight
Blank			0
Estimated			0
	Derived by applying the sum of the components		Lowest weight of the components
	Interpolated but with additional information		2
	Method description is unclear or provides very little information		0
	Time trend interpolation		1
	Uses data from other countries		0
		Abstract that's not from something documented	1 or method (whichever is bigger)
		Adjusted	0 or method (whichever is bigger)
		Adjusted using something	2 or method (whichever is bigger)
		Any suggestion that the WHO is unclear or unsure about some aspect of the data point's metadata	0 or method (whichever is bigger)
		Approximation	0 or method (whichever is bigger)
		Assumption	0 or method (whichever is bigger)
		Both blank	0 or method (whichever is bigger)
		Both with no intelligible information	0 or method (whichever is bigger)
		Budget address	1 or method (whichever is smaller)
		Calculation was used to generate the estimate	1 or method (whichever is bigger)
		Consultation/contact (without an additional documented source)	1 or method (whichever is smaller)
		Consultations with additional source, but no specifics and just consult is documented	1 or method (whichever is bigger)
		Currency conversion	1 or method (whichever is bigger)
		Data delivered/provided/reported	1 or method (whichever is smaller)

Data type	Methods of estimation	Sources and/or Comments	Weight
		by (a non-documented source)	
		Data provided but not clear by whom, with an additional source if additional source is not documented	1 or method (whichever is smaller)
		Derived	0 or method (whichever is bigger)
		Estimated based on	1 or method (whichever is bigger)
		Estimation	0 or method (whichever is bigger)
		Excludes (if it excludes what we do want)	0 (supersedes method)
		Extrapolated	0 or method (whichever is bigger)
		Forecasted	0 or method (whichever is bigger)
		Government department, no explicit documented source	1 or method (whichever is bigger)
		Government ministry, but no explicit documented source	1 or method (whichever is bigger)
		Includes (if it includes what we don't want)	0 (supersedes method)
		Inferred	0 or method (whichever is bigger)
		Interpolation	0 or method (whichever is bigger)
		Missing (if missing something that should be included)	0 (supersedes method)
		Modified	0 or method (whichever is bigger)
		Modified from something/modified using something	2 or method (whichever is bigger)
		Needs assessment	1 or method (whichever is smaller)
		Needs discussion	1 or method (whichever is smaller)
		Needs validation	1 or method (whichever is smaller)
		Needs verification	1 or method (whichever is smaller)
		Only provides hint of a source	1 or method (whichever is smaller)
		Projected	0 or method (whichever is bigger)
		Provides only a vague term that does not provide adequate information to infer or determine what the source is	0 or method (whichever is bigger)
		Reply	1 or method (whichever is smaller)
		Response	1 or method (whichever is smaller)
		Speech	1 or method (whichever is smaller)

Data type	Methods of estimation	Sources and/or Comments	Weight
		Sum of	2 (except for THE, which is lowest weight of components)
		Total of	3 (except for THE, which is lowest weight of components)
		Underestimated	0 (supersedes method)
		Unpublished	1 or method (whichever is smaller)
		Validated figures, but without specifics	2 or method (whichever is bigger)
		Weights	0 or method (whichever is bigger)

Table S2.2 Countries and income groups, number of assigned weights and data completeness

Country	0	1	2	3	4	5	Completeness (%)
Afghanistan	88	60	0	0	0	32	51
Albania	118	0	0	0	0	42	23
Algeria	142	1	0	0	0	37	21
Andorra	0	0	0	120	0	0	66
Angola	153	1	0	0	0	26	15
Antigua and Barbuda	39	0	3	91	0	7	56
Argentina	106	0	33	19	0	22	41
Armenia	80	13	46	0	0	41	55
Australia	0	0	0	180	0	0	100
Austria	0	0	0	180	0	0	100
Azerbaijan	71	0	20	0	0	29	27
Bahamas	0	0	0	180	0	0	100
Bahrain	6	1	0	171	0	2	96
Bangladesh	66	3	14	0	0	97	63
Barbados	16	0	4	144	0	16	91
Belarus	111	3	1	0	0	25	16
Belgium	0	0	0	180	0	0	100
Belize	38	0	20	0	0	102	67
Benin	101	17	19	0	0	23	32
Bhutan	129	0	0	0	3	48	28
Bolivia Plurinational States of	113	0	0	0	0	67	37
Bosnia and Herzegovina	146	2	2	0	0	30	18
Botswana	84	11	0	0	0	85	53
Brazil	123	0	0	0	0	57	31
Brunei Darussalam	0	0	0	120	0	0	66
Bulgaria	156	0	3	0	1	20	13
Burkina Faso	101	2	4	0	9	64	43
Burundi	93	10	3	0	0	74	48
Cabo Verde Republic of	116	3	0	0	0	61	35
Cambodia	95	11	4	0	0	70	47
Cameroon	93	11	1	0	0	75	48
Canada	0	0	0	180	0	0	100
Central African Republic	73	0	0	0	0	107	59
Chad	96	0	0	0	1	63	35
Chile	67	24	0	72	0	17	62
China	42	2	9	0	0	127	76
Colombia	54	49	3	0	0	74	70
Comoros	47	61	16	0	1	55	73
Congo	86	29	16	0	0	49	52

**Cook Islands**	120	0	0	0	0	60	33
**Costa Rica**	126	16	0	0	0	38	30
**Cote d'Ivoire**	110	11	13	0	11	35	38
**Croatia**	52	0	8	99	8	13	71
**Cuba**	81	11	0	0	0	68	43
**Cyprus**	0	0	0	180	0	0	100
**Czech Republic**	36	0	0	126	0	18	80
**Democratic Republic of the Congo**	94	0	18	0	0	68	47
**Denmark**	0	0	0	180	0	0	100
**Djibouti**	96	10	0	0	0	74	46
**Dominica**	117	0	0	0	0	63	35
**Dominican Republic**	125	0	19	0	0	36	30
**Ecuador**	146	0	0	0	0	34	18
**Egypt**	137	4	7	0	0	32	23
**El Salvador**	105	38	12	0	2	23	41
**Equatorial Guinea**	22	27	13	56	1	21	65
**Eritrea**	122	21	0	0	0	17	21
**Estonia**	36	0	0	126	0	18	80
**Eswatini**	100	10	5	0	0	65	44
**Ethiopia**	59	18	8	0	5	90	67
**Fiji**	146	6	0	0	0	28	18
**Finland**	0	0	0	180	0	0	100
**France**	0	0	0	180	0	0	100
**Gabon**	41	0	15	0	0	124	77
**Gambia**	79	0	18	0	0	83	56
**Georgia**	109	0	0	0	0	51	28
**Germany**	0	0	0	180	0	0	100
**Ghana**	50	6	15	0	0	109	72
**Greece**	0	0	0	180	0	0	100
**Grenada**	97	0	11	0	0	32	23
**Guatemala**	72	0	19	0	0	89	60
**Guinea**	123	4	19	0	0	34	31
**Guinea-Bissau**	123	0	32	0	0	25	31
**Guyana**	96	7	0	0	0	77	46
**Haiti**	144	1	0	0	0	35	20
**Honduras**	109	0	29	0	17	25	39
**Hungary**	66	0	6	99	0	9	63
**Iceland**	0	0	0	180	0	0	100
**India**	21	0	0	0	0	159	88
**Indonesia**	24	2	0	0	0	154	86
**Iran (Islamic Republic of)**	119	1	1	0	0	59	33

**Iraq**	83	21	0	0	10	66	53
**Ireland**	0	0	0	180	0	0	100
**Israel**	0	0	0	180	0	0	100
**Italy**	0	0	0	160	0	0	88
**Jamaica**	72	0	0	0	0	108	60
**Japan**	0	0	0	180	0	0	100
**Jordan**	116	5	11	0	0	48	35
**Kazakhstan**	70	0	0	0	0	90	50
**Kenya**	73	59	3	1	1	43	59
**Kiribati**	80	35	0	0	2	63	55
**Kuwait**	0	0	0	180	0	0	100
**Kyrgyzstan**	124	0	2	0	0	34	20
**Lao People's Democratic Republic**	104	15	20	0	1	20	31
**Latvia**	44	22	0	81	0	33	75
**Lebanon**	113	2	0	0	0	65	37
**Lesotho**	73	1	0	0	0	46	26
**Liberia**	136	13	0	0	0	31	24
**Libya**	137	23	0	0	0	20	23
**Lithuania**	93	0	0	72	0	15	48
**Luxembourg**	0	0	0	180	0	0	100
**Madagascar**	113	1	0	0	0	46	26
**Malawi**	71	0	0	0	0	109	60
**Malaysia**	102	0	1	0	0	77	43
**Maldives**	111	9	0	0	0	60	38
**Mali**	43	27	0	0	0	110	76
**Malta**	3	1	1	171	0	4	98
**Marshall Islands**	100	22	0	0	0	58	44
**Mauritania**	130	3	0	0	0	47	27
**Mauritius**	97	11	19	9	0	44	46
**Mexico**	89	29	18	0	0	44	50
**Micronesia (Federated States of)**	98	51	0	0	0	31	45
**Monaco**	0	0	0	120	0	0	66
**Mongolia**	41	0	0	0	0	139	77
**Montenegro**	74	0	31	0	1	34	36
**Morocco**	141	0	19	0	0	20	21
**Mozambique**	122	5	13	0	0	20	21
**Myanmar**	111	0	0	1	0	68	38
**Namibia**	155	2	0	0	0	23	13
**Nauru**	128	0	0	18	0	34	28
**Nepal**	108	33	3	0	0	36	40
**Netherlands**	0	0	0	180	0	0	100

**New Zealand**	0	0	0	180	0	0	100
**Nicaragua**	34	33	24	0	3	86	81
**Niger**	51	0	18	0	0	111	71
**Nigeria**	86	0	0	0	0	74	41
**Niue**	105	34	21	0	0	20	41
**Norway**	0	0	0	180	0	0	100
**Oman**	33	0	0	117	0	30	81
**Pakistan**	145	0	1	0	1	33	19
**Palau**	124	0	0	36	0	20	31
**Panama**	119	0	0	24	0	17	22
**Papua New Guinea**	116	3	5	0	6	50	35
**Paraguay**	119	0	19	0	0	42	33
**Peru**	105	16	38	0	1	20	41
**Philippines**	123	3	24	0	0	30	31
**Poland**	38	12	10	99	0	21	78
**Portugal**	0	0	0	180	0	0	100
**Qatar**	0	0	0	180	0	0	100
**Republic of Korea**	8	0	0	171	0	1	95
**Republic of Moldova**	89	20	3	1	0	67	50
**Romania**	99	3	0	9	1	68	45
**Russian Federation**	46	0	0	27	0	107	74
**Rwanda**	90	21	0	0	1	68	50
**Saint Kitts and Nevis**	88	0	0	81	0	11	51
**Saint Lucia**	62	57	0	0	0	61	65
**Saint Vincent and the Grenadines**	106	16	0	0	0	58	41
**Samoa**	123	3	19	1	0	34	31
**San Marino**	0	0	0	120	0	0	66
**Sao Tome and Principe**	132	20	8	0	0	20	26
**Saudi Arabia**	28	0	4	144	0	4	84
**Senegal**	139	0	2	0	0	39	22
**Serbia**	92	6	0	0	0	82	48
**Seychelles**	108	0	0	54	0	18	40
**Sierra Leone**	140	11	0	0	0	29	22
**Singapore**	0	0	0	180	0	0	100
**Slovakia**	48	8	0	117	0	7	73
**Slovenia**	0	0	0	180	0	0	100
**Solomon Islands**	64	29	0	0	0	47	42
**South Africa**	103	24	0	0	0	33	31
**South Sudan**	115	12	0	0	0	53	36
**Spain**	0	0	0	180	0	0	100
**Sri Lanka**	136	0	15	0	0	29	24

**Sudan**	126	20	14	0	0	20	30
**Suriname**	109	7	0	0	0	64	39
**Sweden**	0	0	0	180	0	0	100
**Switzerland**	0	0	0	180	0	0	100
**Syrian Arab Republic**	83	50	2	0	14	31	53
**Tajikistan**	106	0	1	0	0	53	30
**Thailand**	43	0	0	0	0	137	76
**The Republic of North Macedonia**	59	0	0	0	0	101	56
**Timor-Leste**	122	3	2	0	7	46	32
**Togo**	65	2	2	0	0	111	63
**Tonga**	20	20	0	0	0	140	88
**Trinidad and Tobago**	29	13	0	126	6	6	83
**Tunisia**	116	20	7	0	0	37	35
**Turkey**	128	0	1	0	0	51	28
**Turkmenistan**	96	1	40	0	0	43	46
**Tuvalu**	129	0	0	0	0	11	6
**Uganda**	128	6	0	0	2	44	28
**Ukraine**	94	27	0	0	0	59	47
**United Arab Emirates**	0	0	0	180	0	0	100
**United Kingdom**	0	0	0	180	0	0	100
**United Republic of Tanzania**	91	0	1	0	0	88	49
**United States of America**	0	0	0	180	0	0	100
**Uruguay**	80	3	0	72	1	24	55
**Uzbekistan**	0	20	0	0	0	160	100
**Vanuatu**	97	0	0	0	0	63	35
**Venezuela (Bolivarian Republic of)**	19	0	0	8	0	133	78
**Viet Nam**	105	0	15	0	0	60	41
**Yemen**	80	0	6	0	0	34	22
**Zambia**	147	0	0	0	0	33	18
**Zimbabwe**	120	18	20	0	0	2	22
**High income**	0	0	0	8478	0	0	78
**Low income**	4170	493	290	1	38	2316	64
**Lower-middle income**	5090	513	348	2	58	2931	43
**Upper-middle income**	4535	382	281	2	20	2730	35
**GBD High-income**	264	28	34	5645	0	0	90

Note: Values represent the number of raw datapoints classified as belonging to the respective data quality score of 0-5 in each column.

S2.2 Statistical model to fill missingness in health expenditure variables

After the cleaning up of the data previously described above, we used Spatiotemporal Gaussian process regression (ST-GPR) in order to predict and fill out the missingness that existed in the resulting health expenditure dataset. ST-GPR is a stochastic modeling technique designed to detect signals amidst noisy data. It also serves as a powerful tool for interpolating non-linear trends. Unlike classical linear models that assume that the trend underlying data follows a definitive functional form, GPR assumes that the specific trend of interest follows a Gaussian process, where each point can be estimated with a mean and covariance function.

The first step to implementing ST-GPR is to identify relevant covariates that would be helpful in predicting each health expenditure variable of interest. Using the following set of covariates, we estimated the first stage of the process (space-time) in order to predict and fill up the dependent variables. The covariates used are:

a) All-sector government expenditure per capita, logged
b) Lag Distributed Income, logged
c) Healthcare Access and Quality Index, logged
d) Proportion of total population above the age of 65, logit transformed.

The dependent variables were logs of GHE per capita, PPP per capita and OOP per capita. We also run these models sequentially so that the following model can include the previous component as a covariate. GHE per capita uses only the above covariates, while PPP per capita additionally included GHE per capita as a covariate, and OOP per capita additionally uses GHE per capita and OOP per capita as covariates.

Given the weight of data, we were able to adjust the weight of each data point that contributed to the likelihood function of the Gaussian process, by inflating the pointwise variance for data points with lower weights. For missing data points, the resulting uncertainty was determined by region specific estimates. The final resulting dataset was a complete set of GHE, PPP and OOP per capita estimates for 204 countries from 1995 through 2019, where the uncertainty around each point was constructed by simulating from a normal distribution. Goodness-of-fit was determined using out-of-sample validation and choosing the models with the best performing set of hyperparameters. Overall, ST-GPR is a stochastic modeling technique that performs well at detecting signals among datasets that are noisy. It deviates from classic linear models that assume trend of its underlying data must follow a definitive functional form. Instead, GPR assumes the trend must follow a Gaussian Process form, having a covariance function and a pointwise mean. For the distribution of the Gaussian process, we used a Matérn covariance matrix. GPRs were run repeatedly to ensure that every country-year estimate per metric had 500 draws. This ST-GPR process follows the same form of that found in the Global Burden of Disease Study 2017 appendix.3

Figure S2.1 below shows the comparison of WHO GHED extracted THE data and IHME ST-GPR THE in per capita space from 2000-2019. Figure S2.2 shows the comparison of WHO GHED extracted THE data and IHME ST-GPR THE in per GDP space from 2000-2019.

Figure S2.1 Comparison of WHO GHED extracted and IHME ST-GPR THE in per capita space, 2000-2019

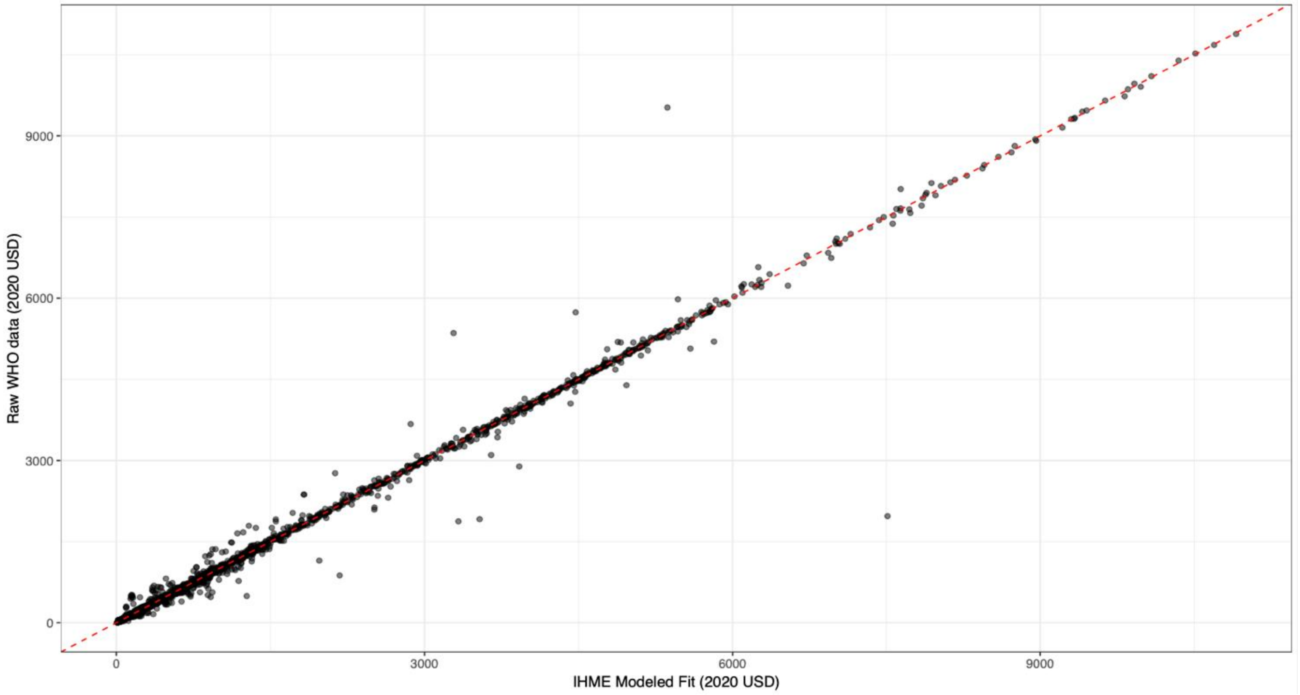

Figure S2.2 Comparison of WHO GHED extracted and IHME ST-GPR THE in per GDP space, 2000-2019

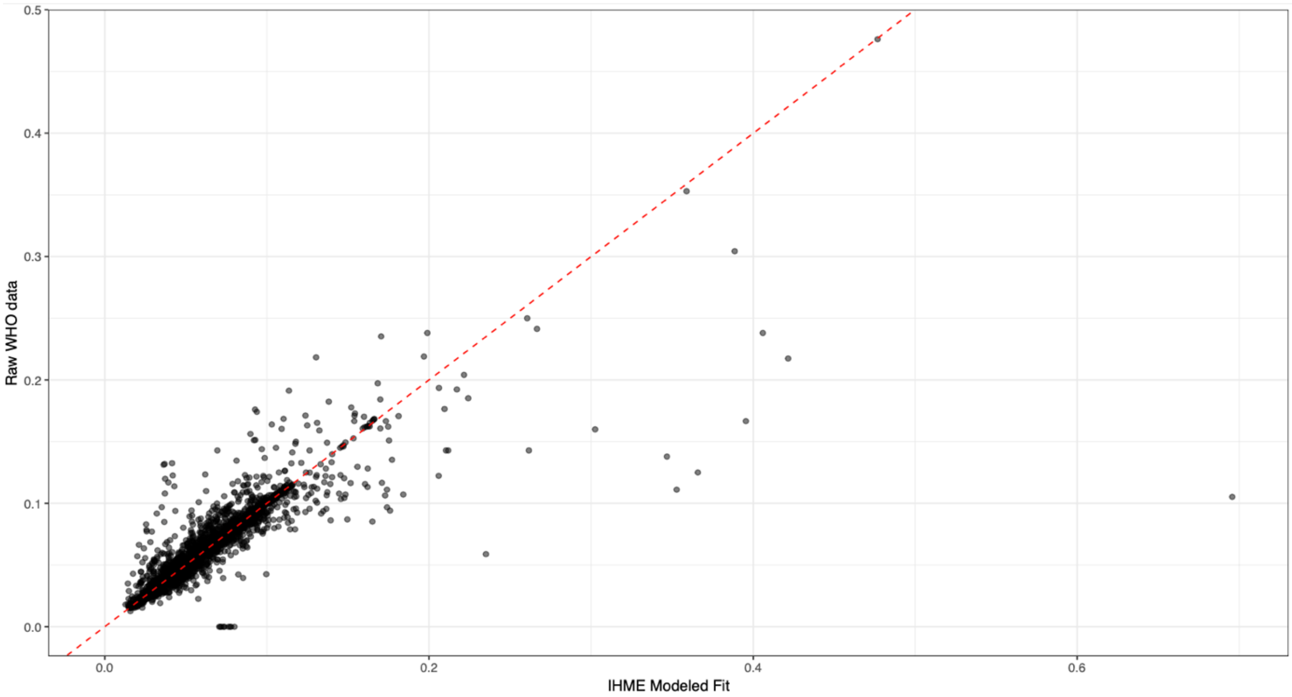

S2.3 Currency exchange and deflation

To convert a metric from real 2021 USD to real 2021 PPP, the following steps were taken. Real USD was exchanged to real LCU (local currency units) by multiplying with country-year specific USD to LCU exchange rates. Then, values were converted from real LCU to real PPP (purchasing-power parity-adjusted dollars) using the country-year specific LCU to PPP conversion series. Any currency deflation from nominal to real currency units or between real currency units in different base years were performed after converting either USD or PPP to LCU, and used country-specific deflator series. All deflators and exchange rates were extracted from the World Bank[4], International Monetary Fund[5], Penn World Tables[6], the United Nations National Accounts[7], and the World Health Organization[1], and were imputed to provide a complete series for each of the variables between 1950 and 2021. We then used several models including ordinary least-squares regression and mixed effects models, to complete each source series from 1950 to 2021, holding constant the last country specific observed data point backwards in time.

Figure S2.3 shows the currency conversion process used in this work.

Figure S2.3 Currency conversion process diagram

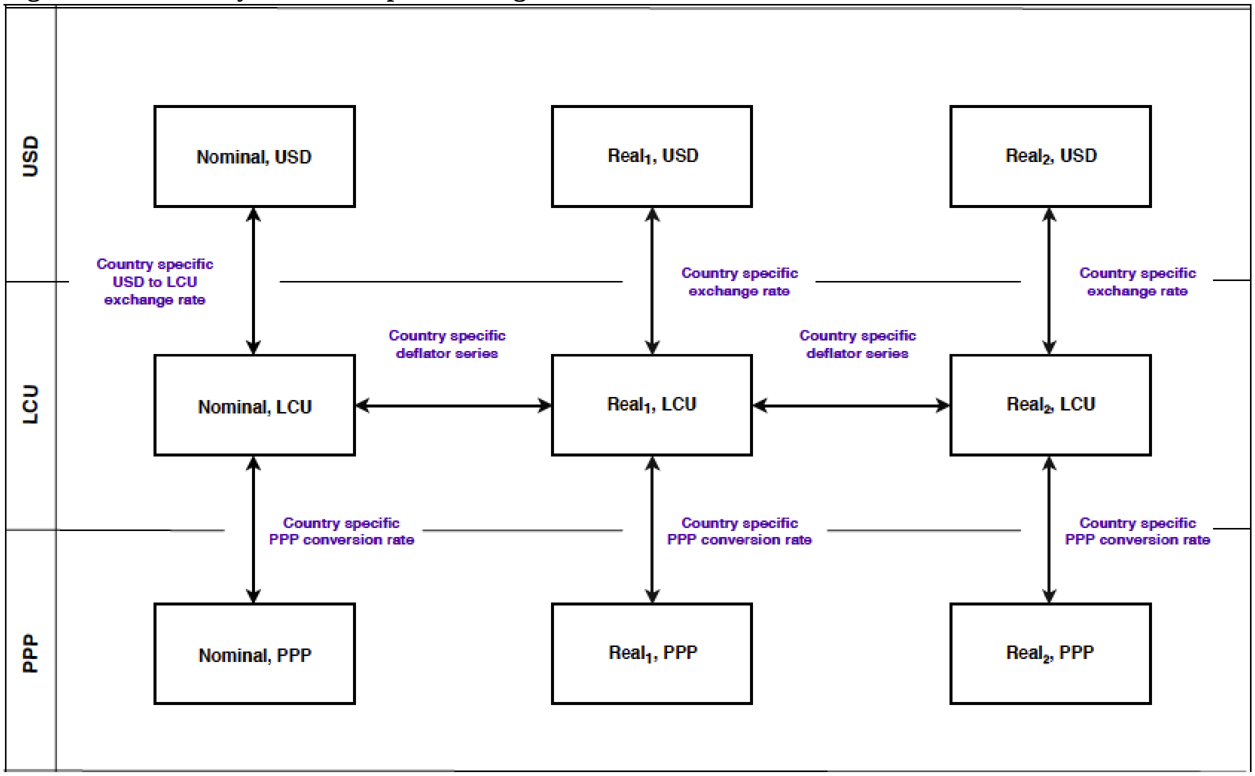

References

1 Global Health Expenditure Database. World Health Organization. http://apps.who.int/nha/database (accessed Feb 4, 2022).

2 World Bank Country and Lending Groups – World Bank Data Help Desk. World Bank. https://datahelpdesk.worldbank.org/knowledgebase/articles/906519-world-bank-country-and-lending-groups (accessed Feb 4, 2022).

3 Stanaway JD, Afshin A, Gakidou E, et al. Global, regional, and national comparative risk assessment of 84 behavioural, environmental and occupational, and metabolic risks or clusters of risks for 195 countries and territories, 1990–2017: a systematic analysis for the Global Burden of Disease Study 2017. The Lancet 2018; 392: 1923–94.

4 World Development Indicators (WDI) | Data Catalog. https://datacatalog.worldbank.org/dataset/world-development-indicators (accessed Feb 4, 2022).

5 World Economic Outlook Database October 2021. https://www.imf.org/en/Publications/WEO/weo-database/2021/October (accessed Feb 4, 2022).

6 Feenstra, Robert C., Robert Inklaar and Marcel P. Timmer (2015), "The Next Generation of the Penn World Table" American Economic Review, 105(10), 3150-3182, available for download at www.ggdc.net/pwt DOI:10.15141/S5Q94M.

7 National Accounts - Analysis of Main Aggregates (AMA). https://unstats.un.org/unsd/snaama/Basic (accessed Feb 4, 2022).

SECTION 3. TRACKING DEVELOPMENT ASSISTANCE FOR HEALTH FROM TRADITIONAL DONORS

List of Figures

Figure S3.1 Comparing CRS commitments, CRS disbursements, and DAC commitments

Figure S3.2 CRS disbursement to commitment ratio and cutoff points by donor agency

Figure S3.3 One- to six-year disbursement schedules for bilateral channels

Figure S3.4 DAH as a percentage of corresponding budget data by bilateral agency

Figure S3.5 Malaria DAH to program areas as assigned by keyword search and PMI reports

Figure S3.6 Comparison of world Bank IDA Replenishment and DAC

Figure S3.7 Disbursements by the African Development Bank

Figure S3.8 Disbursements by Asian Development Bank

Figure S3.9 Disbursements by Inter-American Development Bank

Figure S3.10 Contributions received by the Global Fund to Fight AIDS, Tuberculosis and Malaria

Figure S3.11 The Global Fund to Fight AIDS, Tuberculosis and Malaria's commitments and disbursements

Figure S3.12 Gavi's income and disbursements

Figure S3.13 Total revenue received by non-governmental organizations

Figure S3.14 Expenditure by non-governmental organizations

Figure S3.15 In-kind contributions by loan- and grant-making DAH channels of assistance

List of Tables

Table S3.1. Summary of primary data sources and databases

Table S3.2. Data sources received via personal correspondence

Table S3.3. Additional data sources, databases and model choices used for preliminary estimates of DAH

Table S3.4. Terms for keyword searches

Table S3.5. Additional health focus area categorizations

Table S3.6. World Bank's health sector and theme codes

Table S3.7 Summary of data sources for the regional development banks

Table S3.8 Summary of US non-governmental organizations in the study

Table S3.9 Summary of data sources for calculating in-kind contributions

Box 3.1. EXAMPLE - Australia's primary and additional data sources

Box 3.2. EXAMPLE. Post-keyword search weighting

Box 3.3. EXAMPLE. Australia's CRS disbursement to commitment ratio and cutoff year

Box 3.4. EXAMPLE. Australia's one- to six-year disbursement schedules

Box 3.5. EXAMPLE. Australia's DAH as a percentage of corresponding budget data

S3.1 Overview

We summarize the original methodology as well as updates for this year's estimates below. A more detailed description of the original methodology used to obtain the estimates can be found in Dieleman et al.[1] All known, systematically reported, available data on health-related disbursements and expenditures were extracted, as well as income and revenue from existing project databases, annual reports, and audited financial statements. The channels included and the corresponding data sources are summarized in Table S3.1. Data sources obtained via personal correspondence are summarized in Table S3.2.

DAH for bilateral agencies included all health-related disbursements from bilateral donor agencies, excluding funds that they transferred to any of the other channels we tracked in order to avoid double-counting. This information was extracted from the Creditor Reporting System (CRS) and Development Assistance Committee (DAC) databases of the Development Assistance Committee of the Organisation for Economic Co-operation and Development (OECD-DAC). In some cases, donor agencies did not report disbursement data to the CRS. A method for predicting disbursements from commitment data was implemented to address this challenge. For detailed description of this method see Tracking Development Assistance for Health from Bilateral Aid Agencies and the European Commission section below as well as in Dieleman et al. [1]

For other grant- and loan-making institutions, annual disbursements on health grants and loans were similarly included, excluding transfers to any other channels and ignoring any repayments on outstanding debts. For a more detailed description of this process see Dieleman et al.[1] The annual disbursements for grant- and loan-making institutions only reflect the financial transfers made by these agencies. Therefore, in-kind transfers from these institutions in the form of staff time for providing technical assistance and the costs of managing programs were estimated separately.[1]

Estimates of DAH for the United Nations (UN) agencies included annual expenditures on health both from their core budgets and from voluntary contributions. Calculating DAH for the United Nations Children's Fund (UNICEF) involved estimating the fraction of its total expenditure spent on health prior to 2001.[1]

Non-governmental organizations' (NGOs) DAH estimates utilized data from US government sources, GuideStar Research Fundamental Plus dataset and a survey of health expenditure for a sample of NGOs to estimate DAH from US-based and internationally based NGOs receiving support from the US government. We were unable to include other NGOs due to the lack of audited and comparable data.

The database also included an analysis of the composition of health funding by recipient country, health focus area and program area. In this round of updates to the database we incorporated improvements in methodology such as leveraging additional project level descriptions from the Creditor Reporting System for the allocation of disbursements channeled through NGOs and ongoing refinement of our keyword search list. All methodological updates made are detailed in their relevant channel sub-section below. The improvements to our health focus area keyword search terms are detailed in Table S3.4.

For many channels, reporting-time lags prevent primary disbursement data for the most recent year(s). For those years, the values of DAH were predicted, using channel-specific time trends. The methods employed to obtain these predictions are summarized in Table S3.3. In general, these methods depend on data availability. The estimates are based on channel-specific budget, commitment, and appropriations data, and in many cases assume the most recent disbursement patterns persist. Due to the lack of more detailed disaggregated data, estimates for the most recent year are not provided for recipient countries.

We predicted ebola funding in 2020-2021 for bilateral sources and the European Commission by assuming that 2020 and 2021 ebola funding was equal to 2019 ebola funding.

In addition, for SWAp/HSS funding, we allocated SWAP/HSS projects with multiple health focus areas identified by a proportional allocation based on the relative proportions of the project going to the various health focus areas.

For projects that list a recipient as a region, we split DAH among the low-middle income countries that are eligible to receive DAH within that region based on known proportion of DAH. We do not split projects targeted at global initiatives and label the recipient as "global". For example, a project for vaccine development targeted at no specified recipient country would be classified as global.

For countries that only began existing in certain years, we backcasted DAH in years before their existence as follows. For countries that split off from parent countries, we calculated a three-year average ratio of child country DAH received to parent country DAH received. In years before the child country split off, DAH received by the parent country would have included DAH received in the region that would split off to become the child country. Therefore, we reallocated funding from the parent country, in all years before the child country split off, adding this proportion of the parent country's DAH to the child country's DAH and subtracting out this value from the parent country's DAH. By this method, total annual DAH between the parent and child country do not change, but the allocation of funding between the parent country and child country change. For any country that ceased to exist (such as former Yugoslavia and former USSR) and that had observed DAH received in certain years, we split the funding equally among its new constituent countries.

S3.1.1 Currency exchange and deflation

All results are presented in real 2021 US dollars. All disbursement sequences are converted into real 2021 US dollars by taking disbursements in nominal US dollars in the year of disbursement and adjusting these sequences into real 2021 US dollars using US gross domestic product (GDP) deflators[99]. All disbursement sequences in nominal currencies other than US dollars are converted into real 2021 US dollars by adjustment using country-year specific rates from the OECD's exchange rate series from OECD.Stat. If the current year is not yet available, the previous year's deflator value is utilized. OECD does not include an exchange series for the United Arab Emirates. IHME deflator estimates for the United Arab Emirates are leveraged for deflation. Analyses are conducted in Stata (versions 13.1 or 15.1) or R (version 3.6.3).

Table S3.1. Summary of primary data sources and databases

Channel	*Source*
Bilateral agencies	OECD-DAC and CRS databases[2]
European Commission	OECD-DAC and CRS databases[3]
Joint United Nations Programme on HIV/AIDS (UNAIDS)	Financial reports, audited financial statements, and budget[4]
United Nations Children's Fund (UNICEF)	Financial reports and audited financial statements[5–7]
United Nations Population Fund (UNFPA)	Financial reports and audited financial statements[8]
UNITAID	Project level data from IATI database shared through correspondence, financial reports and audited financial statements[9]
Pan American Health Organization (PAHO)	Financial reports and audited financial statements[10]
World Health Organization (WHO)	Financial reports and audited financial statements[11]
World Bank	Online project database and correspondence[12,13]
African Development Bank (AfDB)	Correspondence, replenishment reports, and compendium of statistics[14,15]
Asian Development Bank (ADB)	Online project database and correspondence[81,98]
Inter-American Development Bank (IDB)	Online project database and correspondence[16,17]

Gavi, the Vaccine Alliance	Online project database, cash received database, International Finance Facility for Immunisation (IFFIm) annual reports, Advance Market Commitment for Pneumococcal Vaccines (AMC) annual reports, correspondence and annual reports[18–21]
The Global Fund to Fight AIDS, Tuberculosis and Malaria (The Global Fund)	Online grant database, contributions report and annual reports[22–24]
Coalition for Epidemic Preparedness and Innovation (CEPI)	Project level data through correspondence
NGOs registered in the US	Candid Research Funadmentals PLUS datset, United States Agency for International Development (USAID) Report of Voluntary Agencies (VolAg), tax filings, annual reports, financial statements, RED BOOK Expanded Database, and WHO's Model List of Essential Medicines, Guidestar Research Fundamentals Plus dataset[25–29]
Bill & Melinda Gates Foundation (BMGF)	Online grant database, IRS 990 tax forms, and correspondence[30,31]
Other private US foundations	Foundation Center's grants database[32]
Wellcome Trust	Project level data through correspondence
European Economic Area	Online project database[33] and correspondence[34]
China	Financial reports and yearbooks

Table S3.2. Data sources received via personal correspondence

Channel	*Data received*
World Bank	Health project-level disbursement data, 1990 – 2021[13]
BMGF	Health disbursement data, 2019, 2020[31]
Gavi	Health disbursement data, 2021
CEPI	Health disbursement data, 2021
IDB	Health project-level loan disbursement data, 2015-2021[17]
AfDB	Health project-level disbursement data, 1990 – December 2021[35]
ADB	Health project-level disbursement data, 1990 – 2 February 2022[98]
UNITAID	Health project-level disbursement data, 2007 – 2021[36]
UAE bilateral	UAE Foreign Assistance in Health 1990 - 2008[37]
European Economic Area	Health project-level disbursement data, 2007-2021

Table S3.3. Additional data sources, databases and model choices used for preliminary estimates of DAH

Channel	*Data source*	*Variables used*	*Years of budget data used for modeling**	*Years underlying DAH data not available; thus modeled**	*Model used*
National agencies					

Australia	Australia's International Development Assistance (2008-2021); Australia's Overseas Aid Program (1998-2008)[38,39]	Health official development assistance (ODA): International development assistance budget	1998-2020	2020-2021	Weighted average of actual DAH/budgeted DAH
Austria	Austria Federal Ministry of Finance budget[40]	General ODA: Federal ODA budget	2007-2020	2021	Weighted average of DAH/budgeted ODA
Belgium	Project Budget General – general expenses[41]	General ODA: Foreign affairs, foreign trade development and cooperation	2000-2020	2021	Weighted average of DAH/budgeted ODA
Canada	Canadian International Development Agency – Report on Plans and Priorities[42]	General ODA: Financial summary – planned spending	1996-2020	2021	Weighted average of DAH/budgeted ODA
Denmark	Danish Ministry of Foreign Affairs Budget[43]	General ODA: Budgeted expenditures on overseas development assistance	2000-2020	2021	Weighted average of DAH/budgeted ODA
European Commission	General budget[44]	Data not used as they were inconsistent with disbursements	–	2021	Based on weighted average of trends in member countries
Finland	Document Assembly in budget years 1998-2021[45]	General ODA: Ministry of Foreign Affairs' administrative appropriations, international development	2002-2020	2021	Weighted average of DAH/budgeted ODA
France	Budget and Financial documents[46,47]	General ODA: aggregated project data; Total ODA	2009-2020	2021	Weighted average of DAH/budgeted ODA
Germany	Plan of the Federal Budget[48]	General ODA: Development expenditure	2001-2020	2021	Weighted average of DAH/budgeted ODA
Greece	Ministry of Finance Budget (2013-	General ODA; ODA commitments	1996-2020	2021	Weighted average of

	2018); OECD Data (1996-2020)[2,49,50]				DAH/budgeted ODA
Ireland	Department of Finance – budget 2000-2004; Estimates for Public Services and Summary Public Capital Programme, 2005-2021[51]	General ODA: Summary of adjustments to gross current estimates – international co-operation	2002-2020	2021	Weighted average of DAH/budgeted ODA
Italy	The Italian Agency for Development Cooperation[52]	General ODA: Net development corporation	2007-2020	2021	Weighted average of DAH/budgeted ODA
Japan	Highlights of the Budget for FY1999-2021[53]	General ODA: Major budget expenditures	2003-2020	2021	Weighted average of DAH/budgeted ODA
Korea, South	ODA Korea comprehensive implementation plan[54]	General ODA: Plan for international development cooperation	2008-2020	2021	Weighted average of DAH/budgeted ODA
Luxembourg	State Budget[55]	General ODA: Ministry of Foreign Affairs – budgeted international development cooperation and humanitarian aid	2001-2020	2021	Weighted average of DAH/budgeted ODA
Netherlands	Netherlands International Cooperation Budget (2001-2021)	General ODA: Total annual official development assistance expenditure	2001-2020	2021	Weighted average of DAH/budgeted ODA
New Zealand	Vote Foreign Affairs and Trade (1998-2001); VOTE Official Development Assistance (2002-2021)[56]	General ODA: Total annual official development assistance expenditure	1998-2020	2021	Weighted average of DAH/budgeted ODA
Norway	Norwegian Ministry of Finance National Budget (2014-2021); Correspondence (2000-2013)[57,58]	General ODA: ODA budget	2000-2020	2021	Weighted average of DAH/budgeted ODA
Portugal	Ministry of Finance and Public Administration	General ODA: Integrated service	2003-2020	2021	Weighted average of

	State Budget 2003-2021[59]	expenditure – external cooperation budget			DAH/budgeted ODA
Spain	Annual Plans of Spanish International Cooperation[60]	General ODA: Spanish total development cooperation	2003-2020	2021	Weighted average of DAH/budgeted ODA
Sweden	Correspondence (2000-2010); Ministry of Foreign Affairs Budget (2010-2021)[61]	General ODA: Ministry for Foreign Affairs budgets for expenditure – international development cooperation	2000-2020	2021	Weighted average of DAH/budgeted ODA
Switzerland	Foreign Affairs (2000-2006); Budget – Further Explanations and Statistics (2007-2021)	General ODA: Direction of development and cooperation (2000-2006); foreign affairs – international cooperation, development aid (in the South and East) (2007-2016)	2000-2020	2021	Weighted average of DAH/budgeted ODA
United Kingdom	IATA (Department for International Development (DFID)) [62,63]	General ODA: assistance for international development; Sum (revised) - aggregated project data	1998-2020	2021	Weighted average of DAH/budgeted ODA
United States	Foreign Assistance Dashboard (2006-2021); Budget of the US Government (2005-2021)[64,65]	Global health ODA: Planned foreign assistance for health; Department of Health and Human Services global health budget	2005-2020	2021	Weighted average of actual DAH/budgeted DAH
UN agencies					
WHO	Programme budget[66]	DAH budget: Programme budget	2002-2020	2021	Weighted average of DAH/budget

UNAIDS	Unified Budget and Workplan, bienniums 2002-2017, 2018-2019, 2020-2021[67,68,69]	DAH budget: Unified Budget and Workplan	2002-2020	2021	Weighted average of DAH/Core Budget
UNICEF	Financial report and audited financial statements[7,69,70]	Total expenditure; Total health expenditure	1990-2020	2021	Weighted average of DAH/budget
UNFPA	Audited Financial report, statistical and financial review, contributions report, annual report, and Structured Funding dialogue [71,72]	Total health expenditure	2002-2020	2021	Weighted average of DAH/budget
PAHO	Proposed program budget[10]	Total regular budget, estimated voluntary contributions	2000-2020	2021	Weighted average of DAH/budget
NGOs	VolAg (1990-2014), GuideStar (2015-2018), sample of top NGOs 2[5,26]	Revenue breakdowns for: US public, non-US public, private, in-kind, BMGF; total overseas expenditures	1990-2018	2019-2021	Regression on DAH, US GDP, and USAID and private voluntary organization (PVO) revenue

* Years of budget data used for modeling versus years underlying DAH data unavailable thus modeled: The data used to estimate DAH by channel vary across channels. Table S3.1 reports our primary data used for each channel. Due to reporting lags there are some years we need to estimate disbursement using additional data sources. These additional data sources, the years in which the primary data is modeled, the years the additional data is available, and the methods for this estimating these modeled years are reported in Table S3.3. Years of budget data used for modeling are the years of additional data available to us. We rely on historic trends to inform our estimates so we rely on many years of additional data despite only modeling a few years of primary data. Years underlying DAH data unavailable thus modeled are the years the primary data is incomplete and thus estimated using additional data. See example below for more details for Australia.

Box 3.1. EXAMPLE - Australia's primary and additional data sources

Project-level data for health-related projects funded by Australia's bilateral aid agencies are available from the OECD's CRS database through 2020. This is the primary data source used to estimate DAH channeled by Australian aid agencies, as described in Table S3.1. 2021 is incomplete because of lags in reporting. To estimate DAH disbursed for 2021, additional data are available from Australia's International Development Assistance budget (2008-2021) and Australia's Overseas Aid Program budget (1998-2008), as described in Table S3.3. These sources provide health-specific official development assistance (ODA) budgeted by Australia, 1998-2021. We convert countries' budgeted ODA, as given in nominal local currency units, to nominal US dollars using the OECD's currency exchange rate series based on USD monthly averages. To estimate DAH disbursed in 2021, we

calculated the ratio of disbursed DAH (from the CRS database) relative to budgeted DAH (from the International Development Assistance and Overseas Aid Program budgets) for 1998-2020. We combine the most recent three ratios into a single estimate by taking a weighted average, weighting substantially higher the most recent year. We multiply this ratio – the estimated disbursed DAH to budgeted DAH – by the 2021 budgeted DAH to estimate disbursed DAH in that years. These methods are described more fully in Dieleman et al.[1]

S3.1.2 DISAGGREGATING BY HEALTH FOCUS AREA

Similar to our previous work, the analysis of health focus areas included assessments of development assistance for HIV/AIDS, tuberculosis (TB), malaria, reproductive and maternal health, newborn and child health, other infectious diseases (including COVID-19), non-communicable diseases, and SWAps and health system strengthening by using keyword searches within descriptive fields. These were chosen as the areas of focus because of their relevance to current policy debates about global health financing and data availability.

In effect, DAH was disaggregated into nine health focus areas: HIV/AIDS, tuberculosis, malaria, reproductive and maternal health, newborn and child health, non-communicable diseases, SWAps/health sector support, other infectious diseases, and other. For most data sources, project-level data were available only through 2020. Methods to estimate health focus area allocations for 2020 are described in more detail below. Keyword searches were performed for a subset of global health channels that provide project-level data with project titles or descriptions. These sources include the bilateral development assistance agencies from 24 DAC member countries, one DAC participant country, THE GLOBAL FUND, the World Bank, Asian Development Bank, African Development Bank, Inter-American Development Bank, Bill & Melinda Gates Foundations, Non-government Organizations, US foundations, Wellcome Trust and the European Economic Area. The keywords used are outlined in Table S3.4 below. Descriptive fields were adjusted so that they were all in capitalized letters, special characters were modified, and search terms with multiple words were put between quotation marks. All keywords were translated into nine major languages (English, Spanish, French, Portuguese, Italian, Dutch, German, Norwegian, and Swedish) used in the OECD CRS, checked for double meanings across all languages, and adjusted accordingly.

Total DAH was split across the health focus areas using weighted averages based on the number of keywords present in each project's descriptive variables. If, for example, three keywords suggested the project focused on HIV/AIDS and two keywords related to tuberculosis, three-fifths of the project's total DAH was allocated to HIV/AIDS and two-fifths was allocated to tuberculosis. To account for the sensitivity of this method, several checks were implemented after the keyword searches to ensure the project was accurately categorized. First, projects that were tagged as child and newborn vaccines and other infectious diseases were categorized as child and newborn vaccines only. Second, projects that were tagged as one of the three major infectious diseases (HIV/AIDS, tuberculosis, or malaria) and other infectious diseases were categorized under only HIV/AIDS, tuberculosis, or malaria.

Box 3.2. EXAMPLE. Post-keyword search weighting

A project in the CRS database had a value of $1,000 of DAH. A keyword search conducted on this project's title and description tagged five keywords: 3 keywords related to HIV/AIDs and 2 keywords related to tuberculosis. Therefore, $600, or 3/5 of total DAH, was allocated to HIV/AIDS, while $400, or 2/5 of total DAH, was allocated to tuberculosis.

In addition to keyword searches, funds were allocated to health focus areas based on characteristics of the channel or additional channel variables. For the bilateral agencies and the EC, purpose codes from the CRS were used to supplement keyword searches. For the World Bank-IDA and -IBRD, health focus areas were also determined by the project sector codes and theme codes, which included percentages of health funds that targeted each theme. All funds from Gavi were allocated to child and newborn vaccines, health system strengthening and non-communicable diseases and all funds from UNICEF to HIV/AIDS prevention, newborn and child health, ebola, and reproductive

and maternal health – other (plus a very small amount being unallocable). Funds from THE GLOBAL FUND were distributed to malaria, HIV/AIDS, TB, and health sector support based on disease components. Within each disease component, keyword searches on programmatic budget data and project descriptions were conducted to distribute among program areas. Funds from UNAIDS were allocated to HIV/AIDS and TB with specific program areas determined by budget information. UNITAID funds were allocated based on assignments made by correspondence. UNFPA funds were allocated to specific health focus areas based on project expenditure data from its financial review report. PAHO and WHO funds were allocated to specific health focus areas based on project expenditure data from correspondence budget data and their proposed programme budgets. For all channels, projects listed as HIV/TB were distributed evenly among the two health focus categories. See Table S3.5 below for more details on these categorizations.

Table S3.4. Terms for keyword searches

Health focus area level	*Program area*	*Keywords*
HIV/AIDS	HIV envelope/other	" HUMANIMMUNODEFVIRUS " " SIDA " " OVC " " H I V " " HIV " " AIDS " " HUMAN IMMUNODEFICIENCY " " REVERSE TRANSCRIPTASE INHIBITOR " " ACQUIRED IMMUNE DEFICIENCY SYNDROME " " ACQUIRED IMMUNNODEFICIENCY " "RETROVIRAL " " VCT " " MALE CIRCUMCISION" " ART " " ARV " " CD4 COUNT " " HAART " " PMTCT " " MOTHER TO CHILD TRANSMISSION" " MOTHER TO CHILD AIDS TRANSMISSION" " PARENT TO CHILD TRANSMISSION" " PRESIDENT S EMERGENCY PLAN FOR AIDS RELIEF " " PEPFAR " " THREE DISEASES FUND " " 3 DISEASES FUND " " EMTCT " " 90 90 90 " " MOTHERTOCHILD"
	Care and Support	" CAREANDSUPPORT " " CARE ACTIVIT" " PAIN RELIEF " " SYMPTOM RELIEF " " PSYCHO SOCIAL SUPPORT " " CHRONICALLY ILL " " CLINICAL MONITORING " " CARE AND SUPPORT " " PSYCHOLOGICAL SERVICE" " PSYCHOLOGICAL SUPPORT " " PSYCHOSOCIAL SUPPORT " " PSYCHOSOCIAL SERVICE" " MATERIAL SUPPORT " " HOME BASED CARE " " PALLIATIVE CARE "
	Counseling and Testing	" COUNSELING " " TESTING " " VCT " " COUNSELLING " " COUNSELINGANDTESTING " " DIAGNOS"
	Orphans and Vulnerable Children	" VULNERABLECHILD" " OVC " " ORPHAN" " VULNERABLE CHILD" " INFECTED CHILD"
	Prevention of mother-to-child transmission (PMTCT)	" MOTHERTOCHILD" " MOTHER TO CHILD" " PARENT TO CHILD" " PMTCT " " EMTCT " " OPTION B "
	Prevention	" CONDOM" " PREVENT" " HIV EDUCATION " " AIDS EDUCATION " " REDUCING THE TRANSMISSION OF HIV " " REDUCE THE TRANSMISSION OF HIV " " MALE CIRCUMCISION" " SAFE BLOOD SUPPL" " SAFE INJECTION" " ABSTINENCE " " BLOOD SAFETY " " MICROBICIDE" " HARM REDUCTION " " PREP " " PEP " " PROPHYLAXIS " " ABCD " " BE FAITHFUL " " EARLY DETECTION "
	Treatment	"RETROVIRAL " " TREAT" " ART " " ARV " " CD4 COUNT " " HAART " " VIRAL LOAD " " VIRAL BURDEN " " VIRAL TITER " " DRUG REGIMEN" " FIRST LINE " " REVERSE TRANSCRIPTASE INHIBITOR "
	Antimicrobial resistance	" ANTIMICROBIAL RESISTAN" " ANTI MICROBIAL RESISTAN" " ANTIBIOTIC RESISTAN" " AMR " "DRUG

Health focus area level	Program area	Keywords
		RESISTAN" " MDR " " XDR " " RESISTANCE TESTING " " DRUG SUSCEPTIBILITY TESTING " " DST " " SECOND LINE "
Tuberculosis	Tuberculosis envelope/other	" TUBERCULOSIS " " TB " " TBC " " TUBERCULAR" " DOTS " " DIRECTLY OBSERVED TREATMENT " " RIFAMPICIN " " ISONIAZID " " THREE DISEASES FUND " " 3 DISEASES FUND " " RIFAMPIN "
	Treatment	" TREAT" " DOTS " " FIRST LINE " " DRUGS " " RIFAMPICIN " " RIFAMPIN " " ISONIAZID " " INH " " PYRAZINAMIDE " " PZA " " ETHAMBUTOL " " EMB " " STREPTOMYCIN " " SM " " STM " " PATIENT KIT " " INJECTABLE AGENT" " FLUOROQUINOLONES " " REGIMEN" " CASE MANAGEMENT " " ANTIMICROBIAL THERAPY " " DRUG SUSCEPTIBLE " " DRUG SENSITIVE "
	Diagnosis	" MICROSCOPY " " SPUTUM " " SMEAR " " CULTURE " " BACTERIOLOGICALLY CONFIRMED " " LABORATORY CONFIRMED " " GENEXPERT " " XPERT MTB RIF " " CHEST X RAY" " DIAGNOS" " LINE PROBE ASSAY "
	Antimicrobial resistance	" ANTIMICROBIAL RESISTAN" " ANTI MICROBIAL RESISTAN" " ANTIBIOTIC RESISTAN" " AMR " "DRUG RESISTAN" " MDR " " XDR " " RESISTANCE TESTING " " DRUG SUSCEPTIBILITY TESTING " " DST " " SECOND LINE "
Malaria	Malaria envelope/other	" MALARIA " " FALCIPARUM " " ANOPHELES " " ARTEMISININ " " PRIMAQUINE " " INDOOR RESIDUAL SPRAY" " INDOORRESIDUALSPRAY" " IRS " "VIVAX " " BEDNET" " BED NET" " SMITN " " ITN " " LLIN " " INSECTICIDAL NET" " INSECTICIDE TREATED NET" " THREE DISEASES FUND " " 3 DISEASES FUND " " CHLOROQUINE " " ANTI MALARIAL " " ANTIMALARIAL "
	Diagnosis	" DIAGNOS" " CASE DETECTION " " MICROSCOPY " " BLOOD SURVEY" " BIOLOGICAL TESTING " " EDT " " LAMP " " RDT "
	Community outreach	" COMMUNITYOUTREACH " " OUTREACH " " COMMUNITY MOBILIZATION" " AWARE" " COMMUNICATION STRATEGY " " SOCIAL COMMUNICATION " " PARTNERSHIP" " ACTIVITIES NEAR COMMUNITIES " " BCC " " BEHAVIORAL CHANGE COMMUNICATION " " BEHAVIOURAL CHANGE COMMUNICATION " " BEHAVIOR CHANGE COMMUNICATION " " BEHAVIOUR CHANGE COMMUNICATION " " SOCIAL MOBILIZATION "
	Vector control: bednets	" BEDNET" " BED NET" " SMITN " " ITN " " LLIN " " INSECTICIDAL NET" " INSECTICIDE TREAT"
	Vector control: irs	" INDOORRESIDUALSPRAY" " IRS " " REDUCE THE PARASITE RESERVOIR " " FOGGING " " COILS " " LARVICID" " LARVACID" " VECTOR CONTROL" "RESIDUAL SPRAY" " RESIDUALSSPRAY " "INDOOR SPRAY" " INDOORSPRAY "
	Vector control: other than bednets and irs	" PREVENT" " IPT " " SMC " " SEASONAL MALARIA CHEMOPREVENTION "
	Treatment	" ARTEMISININ " " PRIMAQUINE " " ACT " " DRUG" " TREAT" " CASE MANAGEMENT " " COMBINATION THERAPY " " ANTI MALARIAL " " ANTIMALARIAL " " CHLOROQUINE "
	Antimicrobial resistance	" ANTIMICROBIAL RESISTAN" " ANTI MICROBIAL RESISTAN" " ANTIBIOTIC RESISTAN" " AMR " "DRUG RESISTAN" " MDR " " XDR " " RESISTANCE TESTING " " DRUG SUSCEPTIBILITY TESTING " " DST " " SECOND LINE "

Health focus area level	*Program area*	*Keywords*
Reproductive and maternal health	envelope/other	" FERTILITY " " FAMILY PLANNING " " FP " " BIRTH" " WOMEN HEALTH " " WOMEN S HEALTH " " WOMENS HEALTH " " CONTRACEP" " IPPF " " PLANNED PARENTHOOD " " ABORTION" " UNFPA " " POSTPARTUM " " POST PARTUM " " MATERNAL " " MATERNITY " " MOTHER" " SBA " " ANTENATAL " " PRENATAL " " PERINATAL " " POSTNATAL " " FETUS" " FETAL" " IPTP " " REPRODUCTIVE HEALTH " " OBSTETRIC" " PREGNANC" " RH " " REPROD " " RHCS " " SEXUAL HEALTH " " SYPHILIS " " FISTULA " " ANEMI" " ANAEMI" " FOETUS" " FOETAL " " FGM " " FEMALE GENITAL MUTILATION " " FEMALE GENITAL CUTTING " " FEMALE CIRCUMCISION " " SBAS " " OBSTRUCTED LABOR " " OBSTRUCTED LABOUR " " MNCH" " RNCH " " RCH " " RNH " " MNH " " MCH " " EMAS " " MCNH " " PMNCH " "ECLAMPSIA " " PRETERM " " ANC " " WCAH " " TBA " " TBAS " " BIRTH ATTENDANT" " CESAREAN" " CAESAREAN" " C SECTION" " STI " " STD " " SEXUALLY TRANSMITTED " " CHILD MARRIAGE " " EARLY MARRIAGE " " FORCED MARRIAGE " " INFORMAL MARRIAGE "
	Family planning	" FERTILITY " " FAMILY PLANNING " " FP " " BIRTH SPACING " " CONTRACEPT" " FAMILY SIZE" " IPPF " " PLANNED PARENTHOOD " " ABORTION" " BIRTH CONTROL " " CONDOM" " IUD " " VASECTOMY " " TUBULAR LIGATION"
	Maternal health	" POSTPARTUM " " POST PARTUM " " MATERNAL HEALTH " " MATERNAL MORTALITY " " MATERNAL DEATH" " SAFE MOTHERHOOD " " BIRTH ATTENDANT" " SBA " " ANTENATAL " " PRENATAL " " PERINATAL " " POSTNATAL " " FETUS" " FETAL" " IPTP " " MATERNITY " " OBSTETRIC" " PREGNANC" " FISTULA " " SEPSIS " " SEPTICEMIA " " ANEMI" " ANAEMI" " FOETUS" " FOETAL " " SBAS " " OBSTRUCTED LABOR " " OBSTRUCTURED LABOUR " " DELIVERY ROOM" " CHILD DELIVERY " " MIDWIV" " MIDWIFE" "ECLAMPSIA " " PRETERM " " ANC " " TBA " " TBAS " " CESAREAN" " CAESAREAN" " C SECTION" " MNH " " MCH " " MNCH " " MCNH "
Newborn and child health	envelope/other	" NEONATAL " " PERINATAL " " POSTNATAL " " MALNUTRITION " " VITAMIN A " " BREAST FE" " BREASTFE" " MICRONUTRIENT" " FORTIFICATION " " STUNT" " WASTING " " BABY FRIENDLY HOSPITAL INITIATIVE " " BREASTMILK " " BREAST MILK " " IODINE " " IODIZED " " IODIZATION " " VAD " " LACTAT" " FOLIC ACID " " FOLAT" " VACCIN" " IMMUNIZ" " POLIO " " DIPHTHERIA " " TETANUS " " PERTUSSIS " " DTP " " HIB " " ROTAVIRUS " " MEASLES " " IMMUNIS" " HEPB " " INJECTION SAFETY " " RUBELLA " " MENINGITIS " " PENTA " " PENTAVALENT " " PNEUMONIA " " PNEUMOCOCC" " HAEMOPHILUS INFLUENZAE " " TETRA " " GAVI " " CHILDHEALTH " " CHILD HEALTH " " CHILDREN " " INFANT" " NEWBORN" " CHILD MORTALITY " " UNDER FIVE MORTALITY " " CHILD SURVIVAL " " CHILDHOOD ILLNESS" " LRI " " RESPIRATORY INFECTION" " DIARRHEA" " DIARRHOEA" " ORAL REHYDRATION " " ORT " " ORS " " UNICEF " " MNCH" " RNCH " " RCH " " RNH " " MNH " " MCH " " EMAS " " MCNH " " POLIOVIRUS " " LRTI " " GPEI " " GLOBAL POLIO ERADICATION INITIATIVE "

Health focus area level	*Program area*	*Keywords*
	Child/newborn nutrition	" NUTRITION" " MALNUTRITION " " BIRTH WEIGHT " " BIRTHWEIGHT " " VITAMIN A " " BREAST FE" " BREASTFE" " FEEDING " " MICRONUTRIENT" " ZINC " " FORTIFICATION " " STUNT" " WASTING " " UNDERWEIGHT " " BABY FRIENDLY HOSPITAL INITIATIVE " " BREASTMILK " " BREAST MILK " " IODINE " " IODIZED " " IODIZATION " " VAD " " LACTAT" " FOLIC ACID " " FOLAT" " IRON " " DEWORMING "
	Child/newborn vaccines	" POLIO " " VACCIN" " IMMUNIZ" " DIPHTHERIA " " TETANUS " " PERTUSSIS " " DTP " " HIB " " ROTAVIRUS " " MEASLES " " IMMUNIS" " HEPB " " INJECTION SAFETY " " RUBELLA " " MENINGITIS " " PENTA " " PENTAVALENT " " PNEUMONIA " " PNEUMOCOCC" " HAEMOPHILUS INFLUENZAE " " TETRA " " GAVI " " POLIOVIRUS " " DPT " " PCV " " GVAP " " VACCINE ACTION PLAN " " IA2030 " " GPEI " " GLOBAL POLIO ERADICATION INITIATIVE "
Non-communicable diseases	envelope/other	" TOBACCO" " SMOK" "CIGAR" " FCTC " " TFI " " SCHIZOPHRENIA " " MENTAL HEALTH " " NEUROTIC " " NEUROSIS " " NEUROSES " " NEUROLOGICAL" " PSYCHOLOG" " PSYCHIATR" " EMOTIONAL DISORDER" " OBSESSIVE COMPULSIVE " " OCD " " PTSD " " POST TRAUMATIC " " POSTTRAUMATIC " " ALCOHOL DEPENDEN" " ALCOHOL ABUSE " " ADDICTION " " BEHAVIORAL DISORDER" " DRUG ABUSE " " SUBSTANCE ABUSE " " OPIOID " " COCAINE " " AMPHETAMIN" " DEPRESSIVE DISORDER" " DEPRESSION " " DYSTHYMIA " " BIPOLAR " " ANXIETY " " EATING DISORDER " " PHOBIA " " DRUG DEPENDEN" " ATTENTION DEFICIT HYPERACTIVITY DISORDER " " ADHD " " PANIC DISORDER" " SELF HARM " " STRESS DISORDER" " SUBSTANCE USE DISORDER" " DRUG USE DISORDER" " MENTAL ILLNESS" " MENTAL DISORDER" " PSYCHOSOCIAL " " PSYCHO SOCIAL " " HEROIN " " OXYCODONE " " ATTENTION DEFICIT DISORDER " " SUICIDE PREVENTION" " HEADACHE " " ANOREXIA " " BULIMIA " " HYPERKINETIC DISORDER" " PERSONALITY DISORDER" " FETAL ALCOHOL SYNDROME" " PSYCHOSIS " " DOWN SYNDROME " " DOWN S SYNDROME " " DOWNS SYNDROME " " AUTISM " " ASPERGER " " DEVELOPMENTAL DISORDER" " CONDUCT DISORDER" " INTELLECTUAL DISABILIT" " MENTAL DISAB" " MENTAL RETARDATION " " ALZHEIMER" " DEMENTIA" " EPILEPSY " " MIGRAINE " " PARKINSON" " MENTALLY DISAB" " NERVOUS SYSTEM" " SYNAPSE" " NON COMMUNICABLE " " NONCOMMUNICABLE " " CANCER" " CHEMOTHERAPY " " RADIATION " " NEOPLAS" " TUMOR " " LEUKEMIA " " LYMPHOMA " " MYELOMA " " HPV " " HUMAN PAPILLOMA VIRUS " " HEP C " " HEPATITIS C " " DIABET" " INSULIN " " ENDOCRINE " " RHEUMAT" " ISCHAEMIC " " ISCHEMIC " " CIRCULATORY " " CIRRHOSIS " " DIGESTIVE DISEASE" " OTHER DIGESTIVE " " PEPTIC " " APPENDICITIS " " GASTRITIS " " GENITOURINARY " " UROGENITAL " " MUSCULOSKELETAL " " GOUT " " BACK PAIN " " MACULAR " " HEARING " " AUDIOLOG" " PERIODONTAL " " CARIES " " CONGENITAL " " OBESITY " " OVERWEIGHT " " GLAUCOMA " " HYPERTENSI" " HERNIA " "ARTHRITIS " " CLEFT LIP" "

Health focus area level	*Program area*	*Keywords*
		CLEFT PALATE" " PHENYLKETONURIA " " SICKLE CELL" " DREPANOCYTOSIS " " HEMOPHILIA " " HAEMOPHILIA " " THALASSEMIA " " GENETIC DISORDER" " HEART DISEASE" " CHRONIC RESPIRATORY " " COPD " " STROKE " " CATARACT" " CHRONIC OBSTRUCTIVE PULMONARY DISEASE" " ASTHMA " " SKIN DISEASE" " DERMATITIS " " PSORIASIS " " SCABIES " " PHYSICAL DISAB" " DENTAL " " ORAL HEALTH " " CVD " " IHD " " CKD " " KIDNEY DISEASE" " MSK " " EYE " " CEREBROVASCULAR " " VASCULAR " " BLOOD PRESSURE " " ACUTE GLOMERULONEPHRITIS " " ALOPECIA AREATA " " ANEURYSM " " ANGINA " " ARTERY " " ATHEROSCLEROSIS " " ATRIAL FIBRILLATION" " ATRIAL FLUTTER " " BENIGN PROSTATIC HYPERPLASIA " "BLASTOMA" " BLIND " " PREVENTABLE BLINDNESS " " AVOIDABLE BLINDNESS " " BLOOD DISORDER" " BRONCHITI" " CARCINOMA " " CARDIAC " " CARDIO" " CELLULITIS " " CEREBRAL " " CORONARY " " DEAF" " DECUBITUS ULCER " "DIALYSIS" " DUODENITIS " " ECZEMA " " EKZEMA " " EDENTULISM " " ENDOCARDITIS " " FIBROSIS " " G6PD DEFICIENCY " " GALL BLADDER " " BILE DUCT " " GLYCEMI" " GLYCAEMI" " HEMOGLOBINOPATH" " HEMOLYTIC ANEMIA " " HODGKIN" " INSOMNIA " " INTERSTITIAL LUNG DISEASE" " INTESTINAL OBSTRUCTION" " LEUKAEMIA " " MELANOMA" " MULTIPLE SCLEROSIS " " MYOCARD" " NCD " " NECK PAIN " " NEPHRITIS " " NEPHROSIS " " NEURAL TUBE DEFECT" " NEURODEGENERATIVE " " INFLAMMATORY BOWEL" " ONCOLOG" " OPTICAL " " OSTEOMYELITIS " " OTITIS MEDIA " " PANCREATITIS " " PARALYTIC ILEUS " " PERITONEAL " " PNEUMOCONIOSIS " " PROSTATE " " PRURITUS " " SARCOIDOSIS " " PYELONEPHRITIS " " REFRACTIVE ERROR" " RENAL " " RETINA " " SARCOMA " " SUBCUTANEOUS DISEASE" " URINARY DISEASE" " URINARY TRACT INFECTION" " UROLITHIASIS " " URTICARIA " " VENTRICULAR " " VISION LOSS " " ACCOMODATION DISORDER" " SENSE ORGAN " " GUILLAIN BARRE SYNDROME" " IMPETIGO " " LOSE WEIGHT " " BIRTH DEFECT" "PAPILLOMAVIRUS" " GENE DEFECT" " PHYSICALLY DISAB" " TUMOUR" " BRAIN INJUR" " MAMMOGRA" " ANTITUMOR " " ANTITUMOUR " " BARIATRIC" " FATTY LIVER" " IMMUNOTHERAPY " " CHROMOSOMAL ABERRATION" " PERIODONTITIS " " OSTEOPOROSIS " "MALIGNANC" " NEURON"
	Mental health	" SCHIZOPHRENIA " " MENTAL HEALTH " " NEUROTIC " " NEUROSIS " " NEUROSES " " NEUROLOGICAL" " PSYCHOLOG" " PSYCHIATR" " EMOTIONAL DISORDER" " OBSESSIVE COMPULSIVE " " OCD " " PTSD " " POST TRAUMATIC " " POSTTRAUMATIC " " ALCOHOL DEPENDEN" " ALCOHOL ABUSE " " ADDICTION " " BEHAVIORAL DISORDER" " DRUG ABUSE " " SUBSTANCE ABUSE " " OPIOID " " COCAINE " " AMPHETAMIN" " DEPRESSIVE DISORDER" " DEPRESSION " " DYSTHYMIA " " BIPOLAR " " ANXIETY " " EATING DISORDER " " PHOBIA " " DRUG DEPENDEN" " ATTENTION DEFICIT HYPERACTIVITY

Health focus area level	Program area	Keywords
		DISORDER " " ADHD " " PANIC DISORDER" " SELF HARM " " STRESS DISORDER" " SUBSTANCE USE DISORDER" " DRUG USE DISORDER" " MENTAL ILLNESS" " MENTAL DISORDER" " PSYCHOSOCIAL " " PSYCHO SOCIAL " " HEROIN " " OXYCODONE " " ATTENTION DEFICIT DISORDER " " SUICIDE PREVENTION" " HEADACHE " " ANOREXIA " " BULIMIA " " HYPERKINETIC DISORDER" " PERSONALITY DISORDER" " FETAL ALCOHOL SYNDROME" " PSYCHOSIS " " DOWN SYNDROME " " DOWN S SYNDROME " " DOWNS SYNDROME " " AUTISM " " ASPERGER " " DEVELOPMENTAL DISORDER" " CONDUCT DISORDER" " INTELLECTUAL DISABILIT" " MENTAL DISAB" " MENTAL RETARDATION " " ALZHEIMER" " DEMENTIA" " EPILEPSY " " MIGRAINE " " PARKINSON" " MENTALLY DISAB" " NERVOUS SYSTEM" " SYNAPSE"
	Tobacco	" TOBACCO" " SMOK" "CIGAR" " FCTC " " TFI "
SWAps/ Health sector support	envelope/other	" SWAP" " TRAINING " " CAPACIT" " DATA SYSTEM" " SECTOR WIDE APPROACH" " HEALTH SYSTEM" " SECTOR PROGRAM" " BUDGET SUPPORT" " SECTOR SUPPORT " " HSS " " TRACKING PROGRESS " " SKILLED WORKER" " HEALTH WORKER" " SKILLED STAFF " " HEALTH PROFESSIONAL" " FACILITIES " " ESSENTIAL MEDICINES " " POLICY DEVELOPMENT" " TRAINING FACILIT" " TRAINING EQUIPMENT" " TRAINING EQMT " " INSTITUTIONAL STRENGTHENING " " HSPSP " " M&E " " M & E " " MONITORING " " SURVEILLANCE " " GOVERNANCE " " HUMAN RESOURCE" " HUMAN CAPITAL " " SCALING UP " " REALLOCATE RESOURCES " " STRATEGIES AND PROGRAM" " HIV STRATEG" " PROGRAM IN COUNTRY ACTIVITIES " " STRATEGIC INFORMATION " " PROCUREMENT " " EVIDENCE BASED " " CASE REPORTING " " MEDICAL WORKER" " HEALTH CARE PERSONNEL " " OPERATIONAL RESEARCH " " SUPPORTIVE ENVIRONMENT " " INFORMATION SYSTEM" " WORKFORCE " " INFRASTRUCTUR" " MEDICAL EDUCATION " " CASE NOTIFICATION " " CASE FINDING " " LABORATORY STRENGTHENING " " LABORATORY QUALITY " " LABORATORY NETWORK" " CONTROL SERVICES " " INFECTION CONTROL " " CONTROL PROGRAM" " SCALE UP" " STOP TB STRATEGY " " HEALTH EDUCATION " " CONTINUING EDUCATION " " SUPPLY " " HEALTH MANAGEMENT" " HEALTH POLICY " " MANAGEMENT AND COORDINATION " " ADMINISTRATIVE MANAGEMENT " " MANAGEMENT AND ADMINISTRATION " " COLD CHAIN" " HEALTH PROMOTION " " TECHNICAL ASSISTANCE " " DSS " " DISTRIBUTION SYSTEMS " " SERVICE DELIVERY " " HEALTH FACILIT" " CONSTRUCT" " MEDICAL SCHOOL"

Health focus area level	*Program area*	*Keywords*
		"CENTERS OF EXCELLENCE" "NURSE" "DOCTOR" "PHYSICIAN" "MEDICAL LABORATORY SCIENTIST" "SURGEON" "SPECIALIST" "PHARMACIST" "HEALTH LABOR" "LABOR MARKET" "PERSONNEL" "MEDICAL PRACTIONER" "DENTAL PRACTIONER" "TASK SHIFTING"
	Human resources	" INFRASTRUCTUR" " MEDICAL EQUIPMENT" " SURGICAL EQUIPMENT" " HOSPITAL EQUIPMENT" " HOSPITAL EQMT " " BUILDINGS " " HEALTH FACILIT" " CONSTRUCT" " MEDICAL SCHOOL" "CENTERS OF EXCELLENCE" " TRAINING " " CAPACIT" " SKILLED WORKER" " HEALTH WORKER" " SKILLED STAFF " " HEALTH PROFESSIONAL" " HUMAN RESOURCE" " HUMAN CAPITAL " " MEDICAL WORKER" " WORKFORCE " " MEDICAL EDUCATION " " HEALTH EDUCATION " " CONTINUING EDUCATION " " HEALTH MANAGEMENT" " MANAGEMENT AND COORDINATION " " ADMINISTRATIVE MANAGEMENT " " MANAGEMENT AND ADMINISTRATION " "NURSE" "DOCTOR" "PHYSICIAN" "MIDWIFE" "MIDWIVES" "MEDICAL LABORATORY SCIENTIST" "SURGEON" "SPECIALIST" "PHARMACIST" "HEALTH LABOR" "LABOR MARKET" "PERSONNEL" "MEDICAL PRACTIONER" "DENTAL PRACTIONER" "TASK SHIFTING" " TASK SHARING”
	Pandemic preparedness	" PANDEMIC PREPAREDNESS " " PANDEMIC RESPONSE" " PANDEMIC ALERT" " EPIDEMIC ALERT" " EPIDEMIC RESPONSE" " EPIDEMIC PREPAREDNESS " " OUTBREAK RESPONSE" " OUTBREAK ALERT" " OUTBREAK PREPAREDNESS " " PANDEMIC INFLUENZA " " EPIDEMIOLOGICAL INVESTIGATION" " CONTACT MANAGEMENT " " PREPAREDNESS AND RESPONSE PLAN" " PREPAREDNESS & RESPONSE PLAN" " BIOSAFETY MEASURE" "EARLY WARNING " " HEALTH SECURITY PREPAREDNESS " " HEALTH SECURITY RISK ASSESSMENT " " RAPID RESPONSE STRATEG" " CONTACT TRAC" " PREPAREDNESS AND RESPONSE " " PANDEMIC PLANNING " " INTERNATIONAL HEALTH REGULATION " " IHR "
	Monitoring and evaluation	“ M E “ “ M E “ “ MONITORING “ “ EVALUATION “ “ SURVEILLANCE “
Other infectious diseases	envelope/other	" INFECTIOUS " " COMMUNICABLE " " TRICHURIASIS " " YELLOW FEVER " " WHIPWORM " " TRACHOMA " " SCHISTOSOMIASIS " " BILHARZIA " " SNAIL FEVER " " KAYAYAMA FEVER " " RABIES " " ONCHOCERCIASIS " " RIVER BLINDNESS " " ROBLES DISEASE" " LYMPHATIC FILARIASIS " " ELEPHANTIASIS " " LEISHMANIASIS " " LEISHMANIOSIS " " HOOKWORM " " FOOD BORNE " " FOODBORNE " " ECHINOCOCCOSIS " " HYDATID DISEASE" " HYDATIDOSIS " " DENGUE " " CYSTICERCOSIS " " CHAGAS " " TRYPANOSOMIASIS " " SLEEPING SICKNESS " " ASCARIASIS " " TROPICAL DISEASE" " CHOLERA " " DYSENTERY " " PARASITE DISEASE" " FAO " " NEGLECTED TROPICAL DISEASE" "TYPHOID" " LEPROSY " " BURULI ULCER " " EBOLA" " EBOV " " EVD " " ZIKA " " ZIKV " "

Health focus area level	Program area	Keywords
		GUINEA WORM " " DRACUNCULIASIS " " FILARIASIS " " HEPATITIS E" " ENCEPHALITIS " " VARICELLA" " INFLUENZA" " FLU " " NTD " " HEPATITIS A " " HEPATITIS D " " HEP A " " HEP D " " ROUNDWORM" " RINGWORM" " TAPEWORM" " FLATWORM" " CHIKUNGUNYA " " LASSA " " MERS " " NIPAH " " MARBURG " " SARS "
	Ebola	" EBOLA" " EBOV " " EVD "
	Zika	" ZIKA " " ZIKV "
	Antimicrobial resistance	" ANTIMICROBIAL RESISTAN" " ANTI MICROBIAL RESISTAN" " ANTIBIOTIC RESISTAN" " AMR " "DRUG RESISTAN" " MDR " " XDR " " RESISTANCE TESTING " " DRUG SUSCEPTIBILITY TESTING " " DST " " SECOND LINE "

Table S3.5. Additional health focus area categorizations

Channel	*Allocation criteria*	*Health focus area*
Bilaterals and the EC	CRS purpose code 13030, family planning	Family planning
	CRS purpose code 13020, reproductive health care	Maternal health, non-family planning
	CRS purpose code 12240, basic nutrition	Child and newborn nutrition
	CRS purpose code 12250, infectious disease control and the keywords “child” or “vaccine” present in descriptive variables	Child and newborn vaccines
	CRS purpose code 13040, STD control including HIV/AIDS	HIV/AIDS
	CRS purpose code 12262, malaria control	Malaria, unspecified
	CRS purpose code 12250, infectious disease control and no other keywords present in the descriptive variables	Other infectious diseases
	CRS purpose code 12263, tuberculosis control	Tuberculosis
	CRS purpose code 12230, basic health infrastructure	SWAPs/health system strengthening - other
	CRS purpose code 12281, health personnel development	SWAPs/health system strengthening - Human resources
	CRS purpose code 12281, medical education/training	SWAPs/health system strengthening - Human resources
World Bank IDA and IBRD	Theme code reproductive and maternal health	Reproductive and maternal health, other
	Theme code adolescent health	Reproductive and maternal health, other
	Theme code tuberculosis	Tuberculosis, other
	Theme code child health	Newborn and child health, other
	Theme code HIV/AIDS	HIV/AIDS, other
	Theme code malaria	Malaria, other
	Theme code non-communicable diseases	Non-communicable diseases, other
	Theme code nutrition	Newborn and child health, nutrition
	Theme code neglected tropical diseases	Other infectious diseases, other
	Theme code health system strengthening	SWAPs/health systems strengthening, other

Channel	Allocation criteria	Health focus area
	Theme code health finance	SWAPs/health systems strengthening, other
	Theme code health service delivery	SWAPs/health systems strengthening, other
	Theme code private sector delivery in health	SWAPs/health systems strengthening, other
	Theme code pandemic response	SWAPs/health systems strengthening, pandemic prepareness
UNFPA	Family planning, population and development strategies, population and development, population dynamics<br>Output 02. Integrated sexual and reproductive health services	Family planning
	Reproductive health, maternal and newborn health, young people's SRH and sexuality education, HIV and STI prevention services, sexual and reproductive health, sexuality education<br>Output 02. Integrated sexual and reproductive health services	Maternal health
	Output 02. Integrated sexual and reproductive health services	Split equally between Family planning and Maternal health, unspecified, and HIV other
	HIV and STI prevention services, HIV and AIDS	HIV prevention
	Advocacy, Multisector, Gender equality and women's empowerment, reproductive rights, program coordination and assistance, adolescents and youth, civil society and rights for all, ending harmful practices, marginalized girls, protection rights.<br>Output 01. Sexual and reproductive health policies for those furthest behind<br>Output 02. Integrated sexual and reproductive health services<br>Output 06. Adolescents and youth skills and capabilities<br>Output 07. Adolescents and youth policies<br>Output 08. Youth leadership and participation<br>Output 12. Elimination of harmful practices	Reproductive and maternal health - other
	Health workforce capacity	Reproductive and maternal health HSS - human resources
	Data and policies, Data availability and analysis, Data production and dissemination, Monitoring and evaluation, National population data system, Organizational adaptability, Organizational effectiveness and efficiency, Population data analysis, Population dynamics, Program coordination and assistance, Programme effectiveness, Resources management.<br>Output 04. Delivery of sexual and reproductive health commodities<br>Output 05. Accountability for sexual and reproductive health	Reproductive and maternal health HSS - other

Channel	Allocation criteria	Health focus area
	Output 09. Gender equality laws and policies<br>Output 10. Gender and sociocultural norms<br>Output 11. Prevention and addressing of gender-based violence<br>Output 13. Population data systems<br>Output 14. Demographic intelligence<br>OEE1. Improved programming for results<br>OEE2. Optimized management of resources<br>OEE3. United Nationals coordination and coherence<br>OEE4. Communication, resources mobiliztion and partnerships	
UNICEF	HIV/AIDS results	HIV/AIDS – prevention, treatment, care, PMTCT, OVC, and other
	Nutrition results	Newborn and child health – nutrition
	Health results	Reproductive and maternal health – other<br>Newborn and child health – HSS, vaccines, other
	Ebola (starting in 2014)	Other infectious diseases - Ebola
	The keyword search was run on budget information for years 2008-2019<br>Program components in budget documents from 1998 to 2007	All program areas under HIV/AIDS and TB
UNAIDS	Assigned in database received through correspondence	HIV/AIDS (care and strengthening, counseling and testing, orphans and vulnerable children, PMTCT, prevention, treatment, other) , TB (diagnosis, treatment and other), HSS
GLOBAL FUND	Disease components for Malaria, HIV/AIDS, TB, TB/HIV, and Other (health systems strengthening)<br>Keyword search on program service delivery areas	All program areas under Malaria, TB, HIV and Swap/HSS
GAVI	Reproductive, maternal, newborn, child, and adolescent health (divided by 2); Research in human reproduction	Maternal health, unspecified, other non-communicable diseases, maternal newborn child vaccines, health system strengthening
WHO	Nutrition	Child and newborn nutrition
	Vaccine-preventable diseases	Child and newborn vaccines
	Reproductive, maternal, newborn, child and adolescent health (divided by 2)	Child and newborn health, unspecified
	Aging and health;	Maternal, newborn, and child health, unspecified
	HIV/AIDS	HIV/AIDS
	Malaria	Malaria
	Tuberculosis	Tuberculosis
	Mental health and substance abuse	Non-communicable diseases, mental health
	Disabilities and rehabilitation; Non-communicable diseases; Violence and injuries	Non-communicable diseases, unspecified
	Neglected tropical diseases; Tropical disease research; Outbreak and crisis response (50%); Alert and response capacities (50%); Polio eradication	Other infectious diseases

Channel	Allocation criteria	Health focus area
	Health system information and evidence; Integrated people-centered health services; National health policies, strategies and plans; Access to medicines and health technologies and strengthening regulatory capacity; health emergency information and risk assessment (50%); Improved access to quality essential health services; Reduced number of people suffering financial hardships; Improved access to essential medicines, vaccines, diagnostics and devices for primary health care; Determinants of health addressed; Risk factors reduced through multisectoral action; Healthy settings and Health in All Policies promoted; Strengthen country capacity in data and innovation; Strengthen leadership, governance and advocacy for health; Financial, human and administrative resources managed in an efficient, effective, results-oriented and transparent manner	SWAps/health system strengthening
	Country health emergency preparedness and the International Health Regulations; health emergency information and risk assessment(50%); Emergency operations; Emergency core services; Outbreak and crisis response(50%); Epidemic- and pandemic-prone diseases; Alert and response capacities(50%); Infectious hazard management; Countries prepared for health emergencies; Epidemics anf pandemics prevented; Health emergencies rapidly detected and responded to	SWAps/health system strengthening, pandemic preparedness
	Social determinants for health; Health and the environment; Food safety; Antimicrobial resistance; gender, equity and human rights mainstreaming	Other
	HIV/AIDS and STls; HIV/AIDS, TB and malaria (33%)	HIV/AIDS, unspecified
PAHO	Tuberculosis; HIV/AIDS, TB and malaria (33%)	Tuberculosis, unspecified
	HIV/AIDS, TB and malaria (33%); Malaria and other Vector-Borne Diseases (50%);	Malaria, unspecified
	Communicable diseases; Malaria and other Vector-Borne Diseases (50%); Neglected Tropical and zoonotic diseases; Response capacity for communicable diseases; Risk factors for communicable diseases; Elimination of communicable diseases	Other infectious diseases
	Nutrition; Nutrition food safety and food security; Malnutrition	Child and newborn nutrition
	Vaccíne-Preventable Diseases	Child and newborn vaccines
	Women, maternal, newborn, child, and adolescent and adult health	Maternal and child health, unspecified
	Mental Health and psychoactive substance use disorders; Intersectoral action on mental health;	Non-communicable diseases, mental health

Channel	Allocation criteria	Health focus area
	Access to services for NCDs and mental health conditions (50%)	
	Non Communicable Diseases and Risk Factors; Chronic noncommunicable diseases; Access to services for NCDs and mental health conditions (50%); Risk factors for NCDs	Non-communicable diseases, unspecified
	Health systems leadership and governance; Human resources for health; Social protection and financing; Health systems information and evidence; Health services; People-centered integrated health services; Access to medical products and strengthening regulatory capacity; Health governance and financing, national health policies, strategies and plans; Access to comprehensive and quality health services; Access to health technologies; Strengthened stewardship and governance; Increased public financing for health; Strengthened financial protection; Health promotion and intersectoral action; Integrated information systems for health; Data, information, knowledge, and evidence; Research, ethics, and innovation for health; Leadership and governance; Management and administration	Swap/health system strengthening, unspecified
	Health emergencies preparedness and risk reduction; Epidemic and pandemic prevention and control; Health Emergencies Detection and Response	Swap/health system strengthening, pandemic preparedness
	Violence and Injuries; Disabilities and Rehabilitation; Antimicrobial resistance; Aging and health; Gender, equity, human rights and ethnicity; Social determinants of health; Health and the environment; Strategic communications; Management and administration; Flexible and learning organization; Health throughout the life course; Quality care for older people; Response capacity for violence and injuries	Other

S3.1.3 Disaggregating preliminary estimates by health focus area

Estimates by health focus area for years in which descriptive data were not available (usually 2020) were obtained by modeling channel-specific DAH per health focus area as a function of time. Out-of-sample validation was used to test the predictive accuracy of a large suite of models, estimating the models using 1990-2010 data and predicting 2011 and 2012. The potential models included fractional multinomial logit regression, OLS regression, autoregressive integrated moving average (ARIMA) models, Epanechnikov kernel-weighted local polynomial smoothing, and multivariable fractional polynomial models. For each model, time was modeled linearly, with splines, and by including lag-dependent variables. Other methodologies considered included modeling health-focus-area-specific DAH as a dollar amount and as a fraction of the channel-specific total DAH. Lastly, models that involved transforming the dependent variable in natural log and logit transformed space were considered. In order to accommodate zero values in the logit transformation, the transformation described in Smithson and Verkuilen were applied.[73] Over 40 models and specifications were evaluated in total.

Each of the potential model and specification described above were estimated using data from 1990 through 2010, and then the estimated model was used to predict DAH by health focus area for 2011 and 2012. Since we have DAH estimates for 2011 and 2012, we compared the modeled estimates and the observed estimates and calculated average percent deviation and average total absolute deviation for each model and specification across all the channels and health focus areas. A variant of the Epanechnikov kernel-weighted local polynomial smoothing had the smallest average percent deviations and average total absolute error. In this model and specification, health focus area-specific DAH fractions were independently estimated at the channel level after they were logit transformed. Time was the only independent variable included in the model. The health focus area-specific DAH estimates were adjusted so the sum of the channel's health focus area disbursements totaled channel-specific DAH envelope. Our preferred model, the Epanechnikov kernel-weighted local polynomial smoothing, minimized both the average percent deviation and the total absolute error out of sample, predicting two years ahead. See Dieleman et al. for a table that demonstrates the performance of four models, each with their optimal specification (as determined by the out-of-sample average percent deviation and total absolute error).[1]

S3.2 Tracking development assistance for health from bilateral aid agencies and the European Commission

OECD-DAC maintains two databases on aid flows: 1) the DAC annual aggregates database, which provides summaries of the total volume of flows from different donor countries and institutions, and 2) the CRS, which contains project- or activity-level data.[3] This year, we used the DAC databases to track health ODA from 24 OECD-DAC members (Austria, Australia, Belgium, Canada, Denmark, Finland, France, Germany, Greece, Ireland, Italy, Japan, Luxembourg, the Netherlands, New Zealand, Norway, Portugal, South Korea, Spain, Sweden, Switzerland, the United Kingdom, the United States, and the EC), and one DAC Participant country, United Arab Emirates, for the years 1990 to 2020. Observed data for the DAC members was available from 1990 to 2020, and observed data for the United Arab Emirates was available from 2009 to 2020. United Arab Emirates bilateral health ODA from 1990 to 2008 was obtained through personal correspondence. We determined the observed 2019 data for the United States was incomplete after consulting several USAID data tools and other data experts in the field. Therefore, we imputed 2019 and predicted 2021 for the United States.

These two DAC databases track the following types of resource flows:

Official development assistance (ODA), defined as "flows of official financing administered with the promotion of the economic development and welfare of developing countries as the main objective"[2] is tracked from its 30 members (Austria, Australia, Belgium, Canada, Czech Republic, Denmark, Finland, France, Germany, Greece, Hungary, Iceland, Ireland, Italy, Japan, Luxembourg, the Netherlands, New Zealand, Norway, Poland, Portugal, Slovakia, Slovenia, South Korea, Spain, Sweden, Switzerland, the United Kingdom, the United States, and the EC). The CRS also now includes some private ODA, such as that funded by BMGF and the Global Fund to Fight AIDS, Tuberculosis and Malaria (THE GLOBAL FUND), as well as assistance from a number of non-DAC countries such as the United Arab Emirates and Kuwait.

ODA includes:

• Bilateral ODA, which is given directly by DAC members as aid to recipient governments, core contributions to NGOs and public-private partnerships, and earmarked funding to international organizations.

• Multilateral ODA, which includes core contributions to multilateral agencies such as WHO, UNFPA, THE GLOBAL FUND, Gavi, UNAIDS, UNICEF, PAHO, the World Bank, and other regional development banks. Only regular budgetary contributions to these institutions can be reported to the OECD-DAC; hence, extrabudgetary funds, including earmarked contributions that donors can report as bilateral ODA, are not included as multilateral ODA. Only 70% of core contributions to WHO can be counted as multilateral ODA.

a. Official development finance (ODF), which includes grants and loans made by multilateral agencies.

b. Other official flows (OOF), which refers to transactions that “do not meet the conditions for eligibility as Official Development Assistance or Official Aid, either because they are not primarily aimed at development, or because they have a Grant Element of less than 25 percent.”

The DAC aggregate tables include all multilateral development banks, THE GLOBAL FUND, operational activities of UN agencies and funds, and a few other multilateral agencies. The project-level data in the CRS cover a smaller subset of multilateral institutions, including UNAIDS, UNFPA, UNICEF, public-private partnerships including Gavi and THE GLOBAL FUND, some development banks, and BMGF, but do not reflect the core-funded operational activities of WHO prior to 2009, disbursements by Gavi prior to 2007 and BMGF prior to 2009, or all loans from the World Bank.

This research utilized the CRS as the principal source for tracking bilateral DAH. This is because the DAC aggregate tables do not report detailed project-level information about the recipient country and health focus area. The OECD sector codes for general health (121), basic health (122), and population programs (130) were used to identify health flows in the CRS. Only ODA related flows are used in our analysis, including OECD flow codes corresponding to ODA grants (11), ODA grant-like (12), ODA loans (13), and equity investment (19). In addition, we included select projects with OECD sector code for emergency aid (720) in order to capture additional funding for ebola and zika outbreaks. Projects with purpose code emergency food aid (72040) were excluded, and the remaining project descriptive variables were parsed for the keywords “EBOLA” and “ZIKA” to identify emergency aid projects targeting these diseases. Based on our definition of DAH, we also excluded any remaining projects containing any of the following keywords: " WATER " " SANITATION " " FOOD " " EARTHQUAKE " " HURRICANE " " TERREMOTO " " HURACAN " " FAMILY CARE STRUCTURES ".

To avoid double-counting, all identifiable earmarked commitments and disbursements between one disbursing agency to another were tagged and removed before calculating final DAH estimates. Income statements, expenditure statements, and keyword searches in the descriptive project fields were used to identify potential sources of double-counting.

In multilateral agencies, public-private partnerships, development banks, and foundations, when expenditure by recipient agency information was available, double counting was tagged and dropped in the disbursing agency from whom the resources was transferred. In the absence of detailed expenditure data in the disbursing agency that transferred resources, the income statement of the receiving disbursing agency that reflected the disbursement was used to flag the disbursement in the initial disbursing agency. Preliminary estimates were generated based on the annual disbursed envelope exclusive of resources flagged as transfers to other agencies.

Channel codes in the CRS data were used to track DAH to international and donor-country-based non-government organizations. The names of NGOs that were captured in IHME’s NGO data (as detailed in section 3.7 titled “Tracking non-governmental organizations”) were searched for in the CRS descriptive variables and tagged as double-counting. Research funds for HIV/AIDS channeled by the US government through the National Institutes for Health (NIH) were also removed from the total since they do not meet the definition of DAH as contributions from institutions whose primary purpose is development assistance. Official development finance (ODF) from the CRS was not counted because these expenditures were included elsewhere, either in the analysis of multilateral institutions relevant to the study or in the assessment of health spending by BMGF, the data for which were obtained via correspondence and from their annual reports, audited financial statements, and project databases. To avoid double-counting, only health assistance flows from multilateral institutions to low- and middle-income countries were counted, and not transfers to multilateral institutions. Also, for regional projects the disbursements are split amongst all countries in the specified OECD region. For example, a project allocated to recipient “North of Sahara, regional” would have its disbursements split equally between all the countries in the corresponding OECD region: Algeria, Egypt, Libya, Morocco, and Tunisia.

Allocation of funding to health focus areas was assigned as described in the section “DISAGGREGATING BY HEALTH FOCUS AREA”, based on a keyword search of five descriptive variables in the CRS: project title, short

description, long description, channel name, and channel reported name. Additional adjustments were made based on CRS purpose codes, as detailed in Table S3.5, in order to ensure that the specified purpose corresponded to the highest-weighted **bilateral** health focus area.

S3.2.1 Estimating disbursements for the 24 channels and the EC

Both the DAC tables and the CRS rely on information reported by DAC members and other institutions to the OECD-DAC. Hence, the quality of the data varies considerably over time and across donors. Three variables were used to estimate yearly donor disbursements: CRS commitments, CRS disbursements, and DAC commitments. There were two main challenges in using the data from the CRS for this research:

1. underreporting of aid activity to the CRS compared to what is reported to the DAC, and

2. underreporting of disbursement data to the CRS compared to commitment data reported to the CRS.

These issues are highlighted in Figure S3.1. Methods developed to account for both these challenges are discussed below. Details on how we estimated the cost of providing technical assistance and program support for these institutions are highlighted below in the section titled calculating the 'Technical assistance and program support component of development assistance for health from loan-and grant-making channels of assistance'.

To address these two challenges, we determined a cutoff point for each channel. We defined this channel-specific cutoff year as when the ratio of total CRS disbursements to commitments was greater than 50% and did not drop subsequently below 30%. Figure S3.2 below shows each donor's CRS disbursement to commitment ratio in green, and the estimated cutoff year is marked with a vertical red line. For years after the cutoff year, DAH is measured using the unadjusted disbursement data. For the time prior to the cutoff year, it was determined that the disbursement data are not of high enough quality, and adjusted commitments were used instead.

Two adjustments were made to commitments to estimate disbursements before each donor-specific cutoff point:

I. The first adjustment addressed underreporting of aid activity to the CRS (relative to the DAC). To address this challenge, all CRS commitments for the health sector were adjusted upward using the DAC commitment to CRS commitment coverage ratio. The coverage ratio of the CRS was well below 10% before 1996 but has improved steadily over time.

II. The second adjustment addressed underreporting of disbursements data to the CRS (relative to commitments reported to the CRS). To address this challenge, we pooled completed projects in the CRS that have disbursement data for each channel and computed yearly project disbursement rates (the fraction of total commitments disbursed for each year of a project) and overall project disbursement rates (the fraction of total commitments disbursed over the life of each project) by project length. Yearly disbursement schedules were calculated for projects with lengths of one, two, three, four, five, and six years. When an observed project length was more than six years, all expenditure after the sixth year was aggregated and assumed to be expended in the sixth year. This does not happen often. Yearly disbursement rates were the median of these shares, averaged across projects for every donor in each project year. The sum of these averages equals one, so that all the disbursements were expended over the lifetime of a project. We also adjusted donor disbursement schedules by the ratio of disbursements to commitments in the CRS in order to reflect the fact that not all commitments are typically disbursed. The product of these donor-specific yearly disbursement rates, the donor-specific overall disbursement rates, and the donor-specific disbursement to commitment ratios produced the donor-specific disbursement schedules. The donor-specific disbursement schedules were applied to project-level DAC-adjusted commitments reported in the CRS. Figure S3.3 shows the yearly disbursement rates and overall disbursement rates for projects with one- to six-year lifespans for each of the 24 member countries and the EC.

Lastly, to address the challenge of underreporting of aid activity to the CRS compared to the DAC for all years, the difference between each donor's aggregate DAC health commitments and CRS health disbursements was added to

each donor’s yearly DAH. Since only aggregate commitments are reported to the DAC, several adjustments were made, based on more detailed CRS data:

I. First, each donor’s yearly median project length was calculated by applying the donor-specific disbursement schedules described above to CRS projects that had disbursement in order to get adjusted DAC commitments.
II. Commitments for projects that have not opened yet were then subtracted, based on the open date reporting in the CRS. This ensured that future disbursements were not captured.
III. The donor-specific ratio of disbursements to commitments was applied to adjusted DAC commitments in order to account for the fact that not all commitments are usually disbursed.
IV. Lastly, these DAC-adjusted commitments were compared to CRS disbursements, inclusive of transfers that were later dropped as double-counting.

In addition to tracking disbursements from the EC, gross disbursements from the DAC were used to compile data on the sources of funding for the EC.

Figure S3.1 Comparing CRS commitments, CRS disbursements, and DAC commitments

This figure compares commitments and disbursements from the Creditor Reporting System (CRS) and Development Assistance Committee (DAC) databases of the Development Assistance Committee of the Organisation for Economic Co-operation and Development (OECD-DAC) from 1990 to 2020. CRS disbursements are usually underreported when compared to both CRS and DAC commitments data, especially in earlier years. Because of this gap between CRS and DAC, CRS disbursements data were adjusted to fit DAC commitments data. Drop in 2019 across all commitments and disbursements is due to incomplete USA bilateral data for that year.

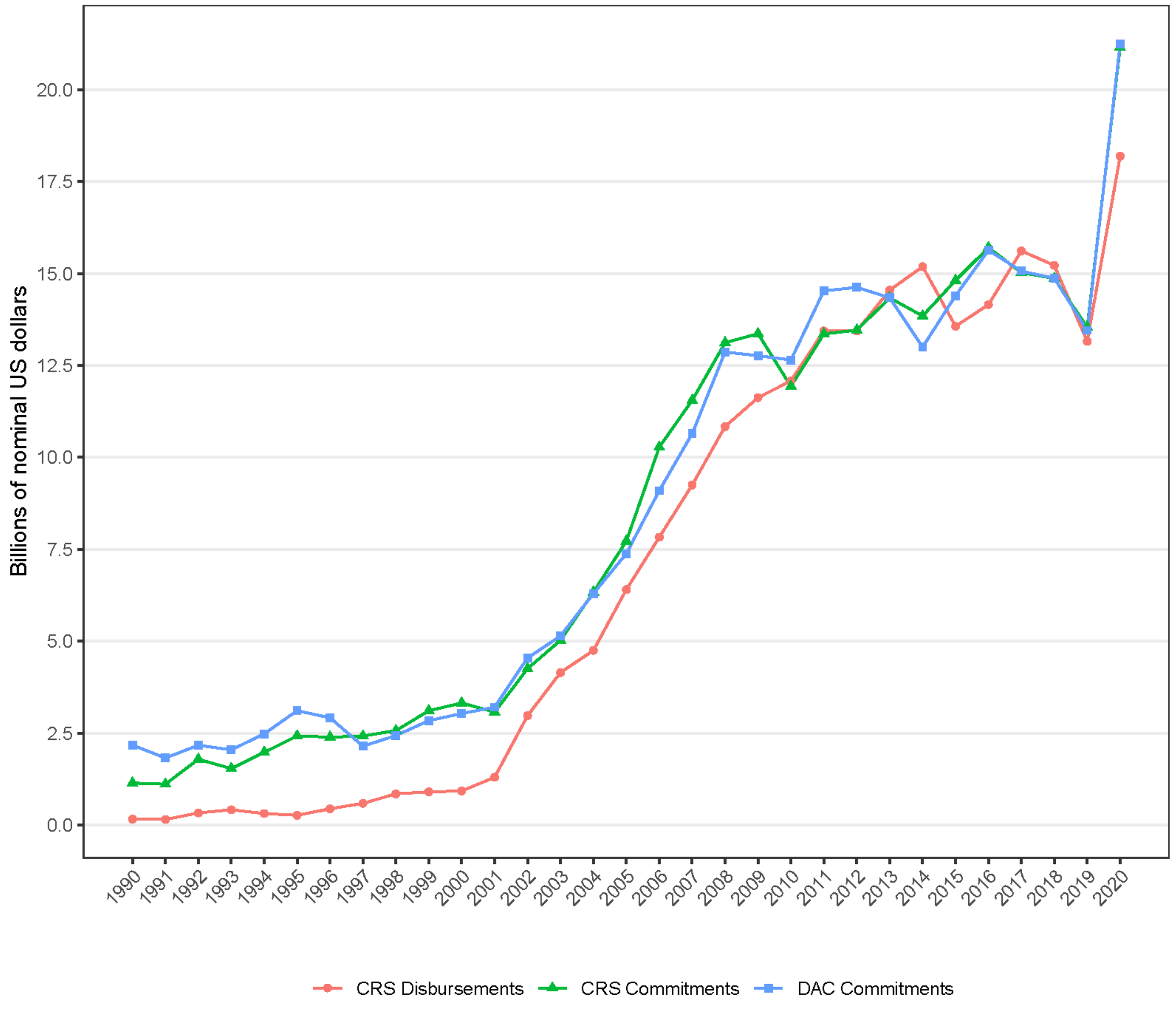

Source: OECD-DAC and OECD Creditor Reporting System

Figure S3.2 CRS disbursement to commitment ratio and cutoff points by donor agency

This figure shows the channel-specific cutoff year. Before this year, we adjust CRS commitments using disbursement schedules. After this cutoff we rely on CRS-reported disbursements. The total CRS disbursements to commitments ratio is in black, and the cutoff year is marked with a vertical red line. The cutoff year is determined to be when the ratio goes above 50% and does not fall back below 30%. The vertical axis represents the CRS disbursement to commitment ratio as a percentage. ARE = United Arab Emirates, AUS = Australia, AUT = Austria, BEL = Belgium, CAN = Canada, CHE = Switzerland, DEU = Germany, DNK = Denmark, EC = European Commission, ESP = Spain, FIN = Finland, FRA = France, GBR = Great Britain, GRC = Greece, IRL = Ireland, ITA = Italy, JPN = Japan, KOR = South Korea, LUX = Luxembourg, NLD = the Netherlands, NOR = Norway, NZL = New Zealand, PRT = Portugal, SWE = Sweden, USA = United States of America.

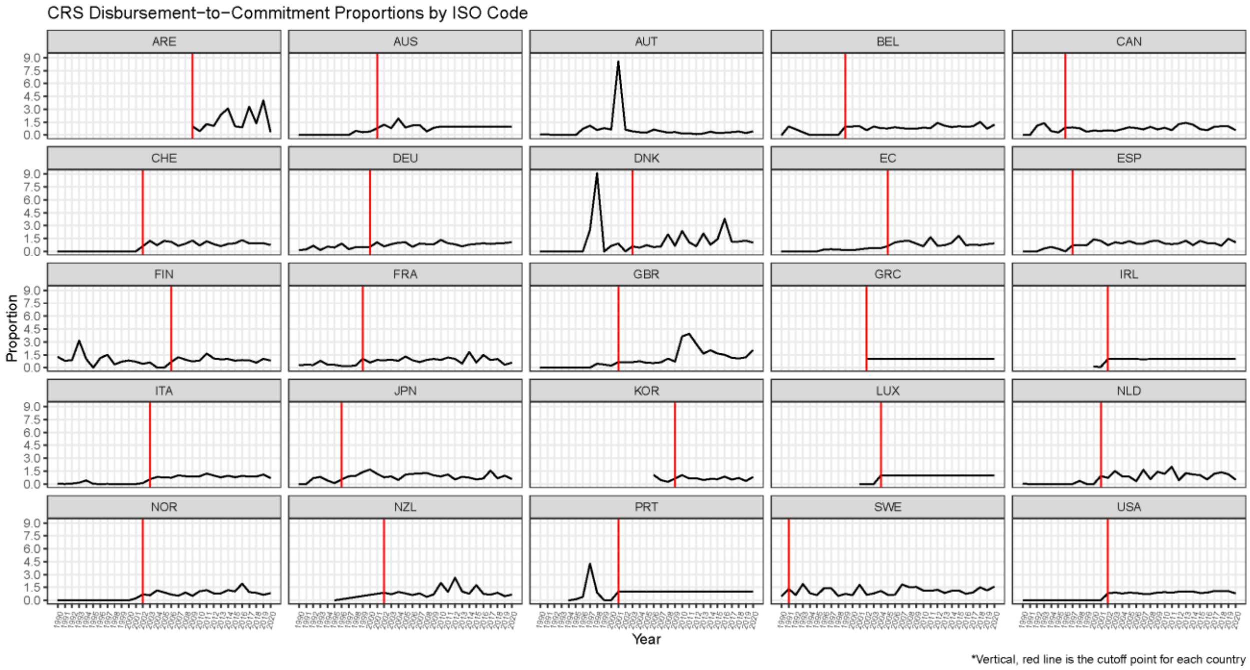

Source: OECD Creditor Reporting System

Box 3.3. EXAMPLE. Australia's CRS disbursement to commitment ratio and cutoff year

The black line shows the ratio of Australia's disbursements to commitments, as reported in the CRS. Prior to 2001, the ratio was always below 50%. In 2001, the ratio rose above 50%; it did not fall below 30% in subsequent years, thereby defining 2001 as the cutoff year. Thus, for Australia, before 2001 DAH is based on adjusted CRS commitment data. These data are adjusted using disbursements schedules (Figure S3.3) and data from the DAC. After 2001, Australia's DAH is based on the disbursements reported in the CRS.

Figure S3.3 One- to six-year disbursement schedules for bilateral channels

This figure shows the estimated disbursement schedules for bilateral channels. Before the channel-specific cutoff year, we rely on commitment data to inform our estimates of DAH. Commitment data are adjusted to reflect disbursements over time using schedules estimated from projects in the CRS that have both commitment and disbursement data. The vertical axis represents the percentage of the commitment disbursed. ARE = United Arab Emirates, AUS = Australia, AUT = Austria, BEL = Belgium, CAN = Canada, CHE = Switzerland, DEU = Germany, DNK = Denmark, EC = European Commission, ESP = Spain, FIN = Finland, FRA = France, GBR = Great Britain, GRC = Greece, IRL = Ireland, ITA = Italy, JPN = Japan, KOR = South Korea, LUX = Luxembourg, NLD = the Netherlands, NOR = Norway, NZL = New Zealand, PRT = Portugal, SWE = Sweden, USA = United States of America

1−Year Disbursement Schedules by ISO Code

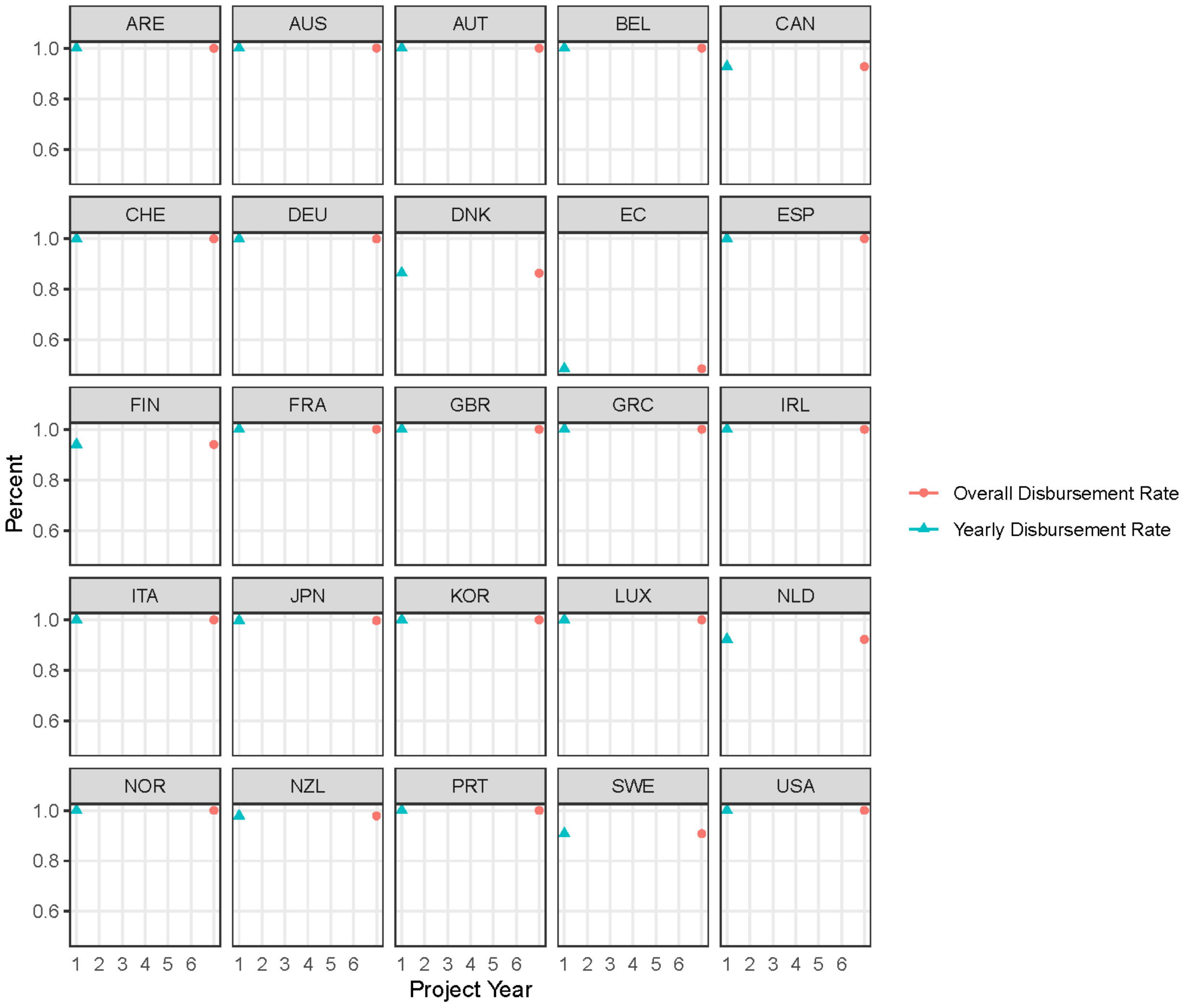

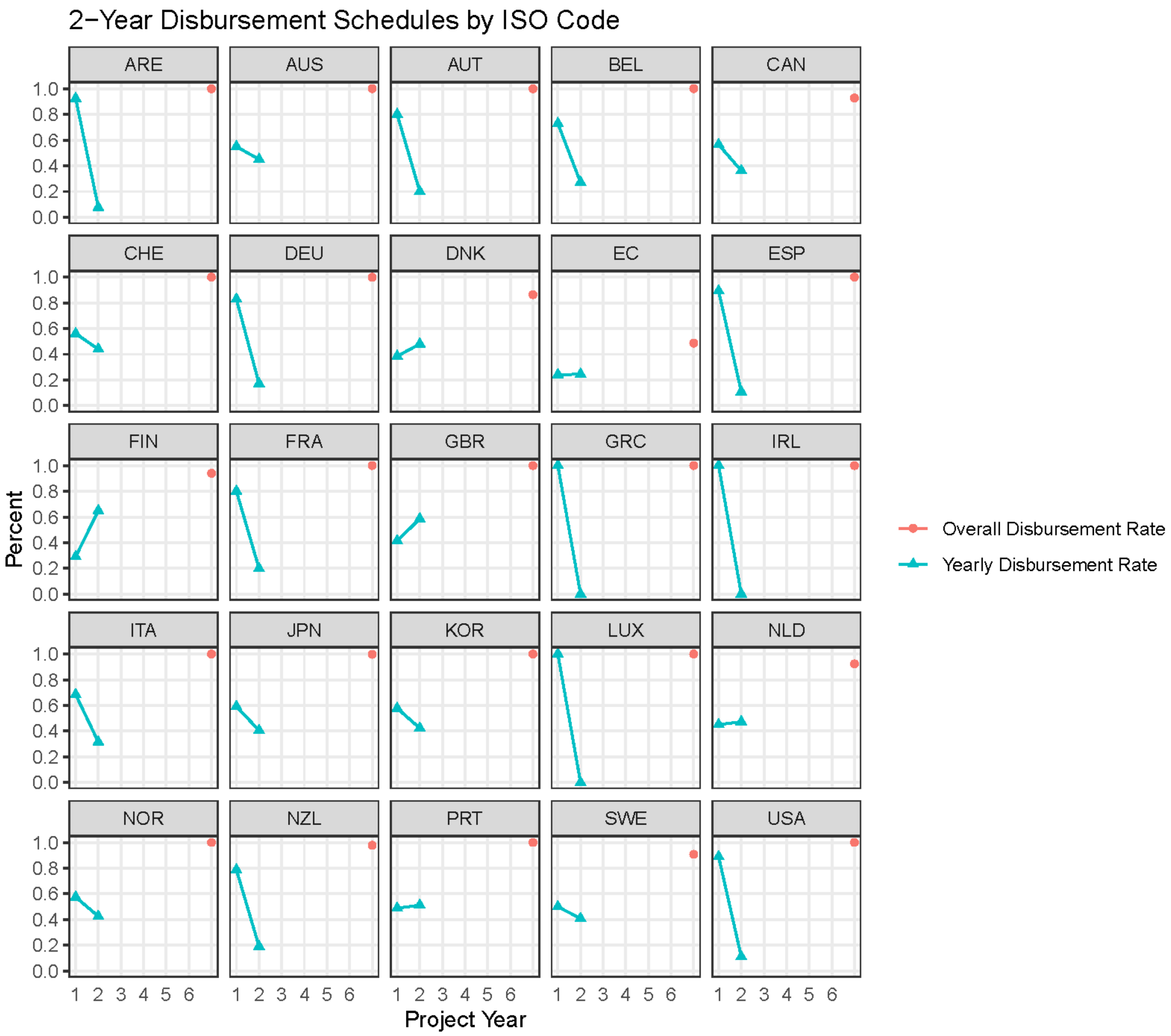
2−Year Disbursement Schedules by ISO Code
ARE
AUS
AUT
BEL
CAN
CHE
DEU
DNK
EC
ESP
FIN
FRA
GBR
GRC
IRL
ITA
JPN
KOR
LUX
NLD
NOR
NZL
PRT
SWE
USA
Percent
0.0 0.2 0.4 0.6 0.8 1.0
1 2 3 4 5 6
Project Year
Overall Disbursement Rate
Yearly Disbursement Rate

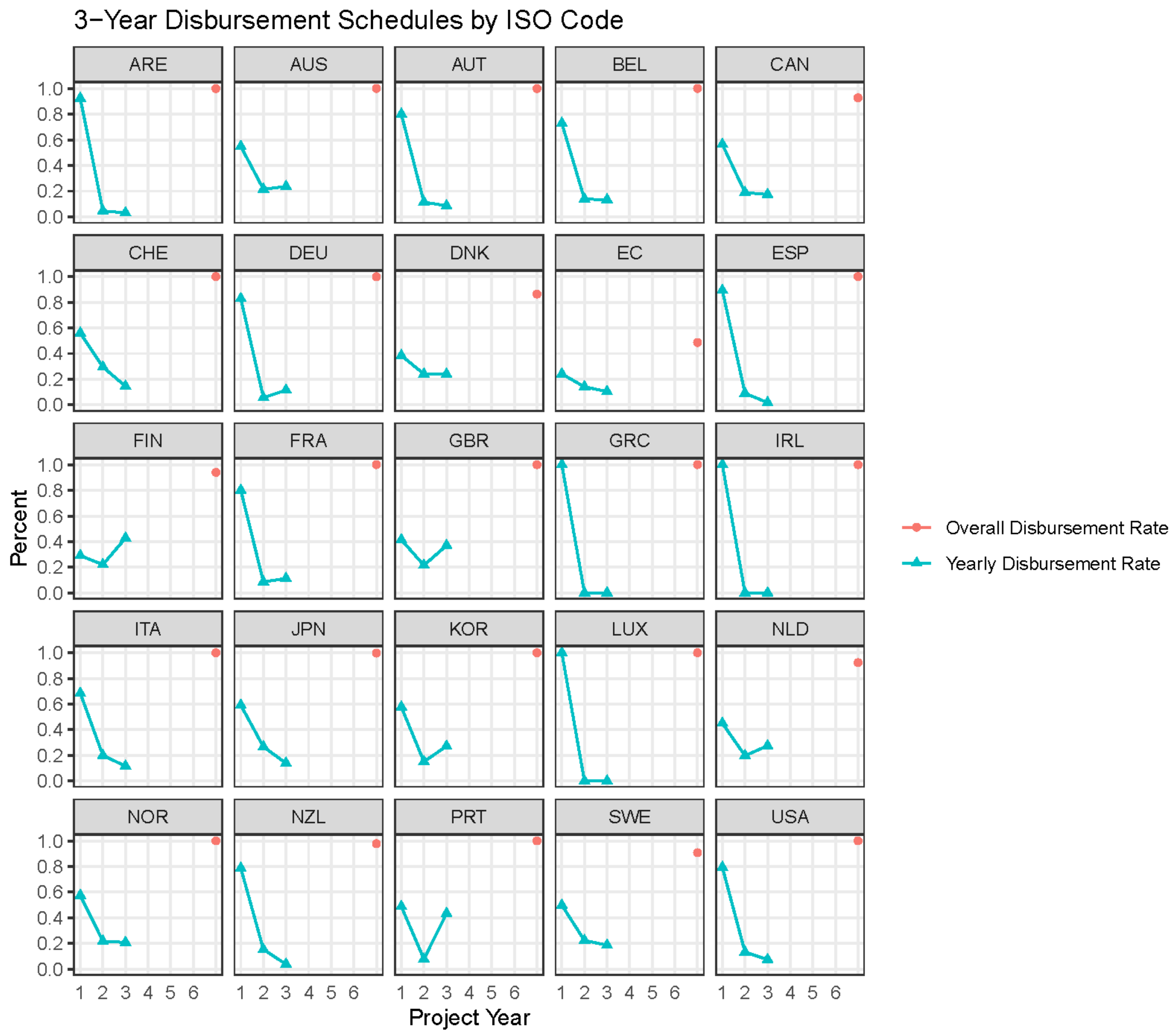
3−Year Disbursement Schedules by ISO Code
ARE
AUS
AUT
BEL
CAN
CHE
DEU
DNK
EC
ESP
FIN
FRA
GBR
GRC
IRL
ITA
JPN
KOR
LUX
NLD
NOR
NZL
PRT
SWE
USA
Percent
1.0
0.8
0.6
0.4
0.2
0.0
1 2 3 4 5 6
Project Year
Overall Disbursement Rate
Yearly Disbursement Rate

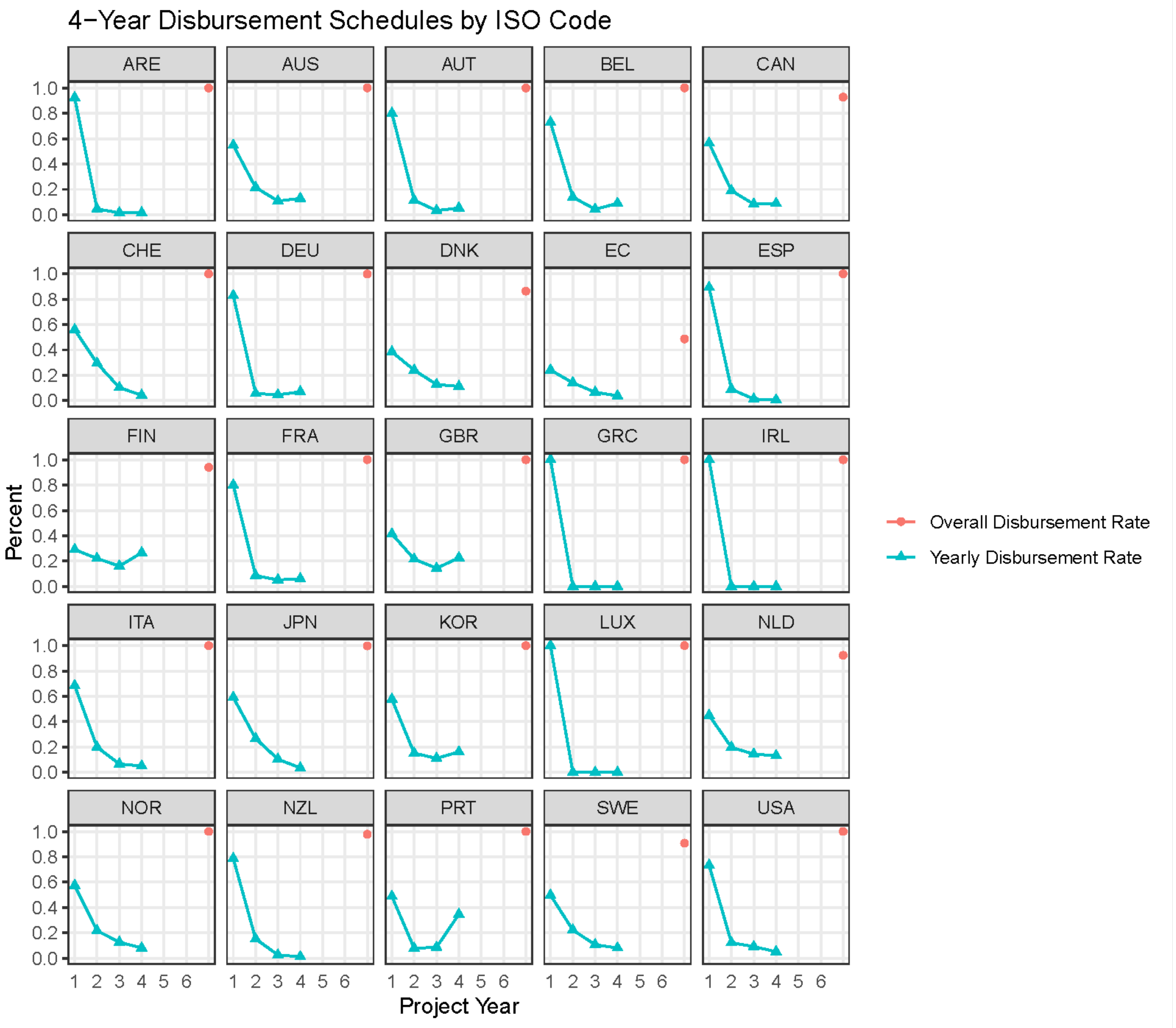
4−Year Disbursement Schedules by ISO Code
ARE
AUS
AUT
BEL
CAN
CHE
DEU
DNK
EC
ESP
FIN
FRA
GBR
GRC
IRL
ITA
JPN
KOR
LUX
NLD
NOR
NZL
PRT
SWE
USA
Percent
1.0
0.8
0.6
0.4
0.2
0.0
1 2 3 4 5 6
Project Year
Overall Disbursement Rate
Yearly Disbursement Rate

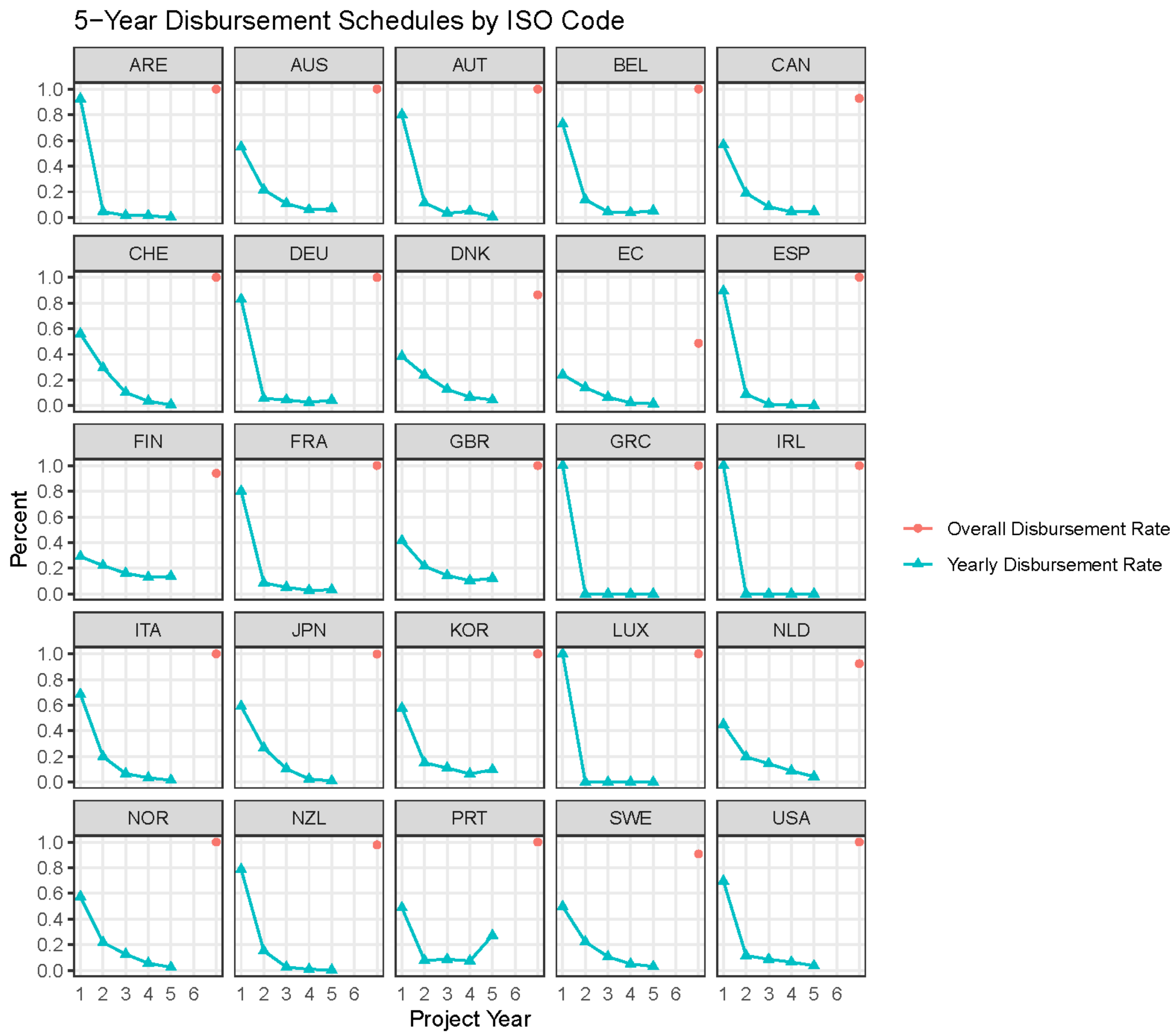
5−Year Disbursement Schedules by ISO Code
ARE
AUS
AUT
BEL
CAN
CHE
DEU
DNK
EC
ESP
FIN
FRA
GBR
GRC
IRL
ITA
JPN
KOR
LUX
NLD
NOR
NZL
PRT
SWE
USA
Percent
1.0
0.8
0.6
0.4
0.2
0.0
1 2 3 4 5 6
Project Year
Overall Disbursement Rate
Yearly Disbursement Rate

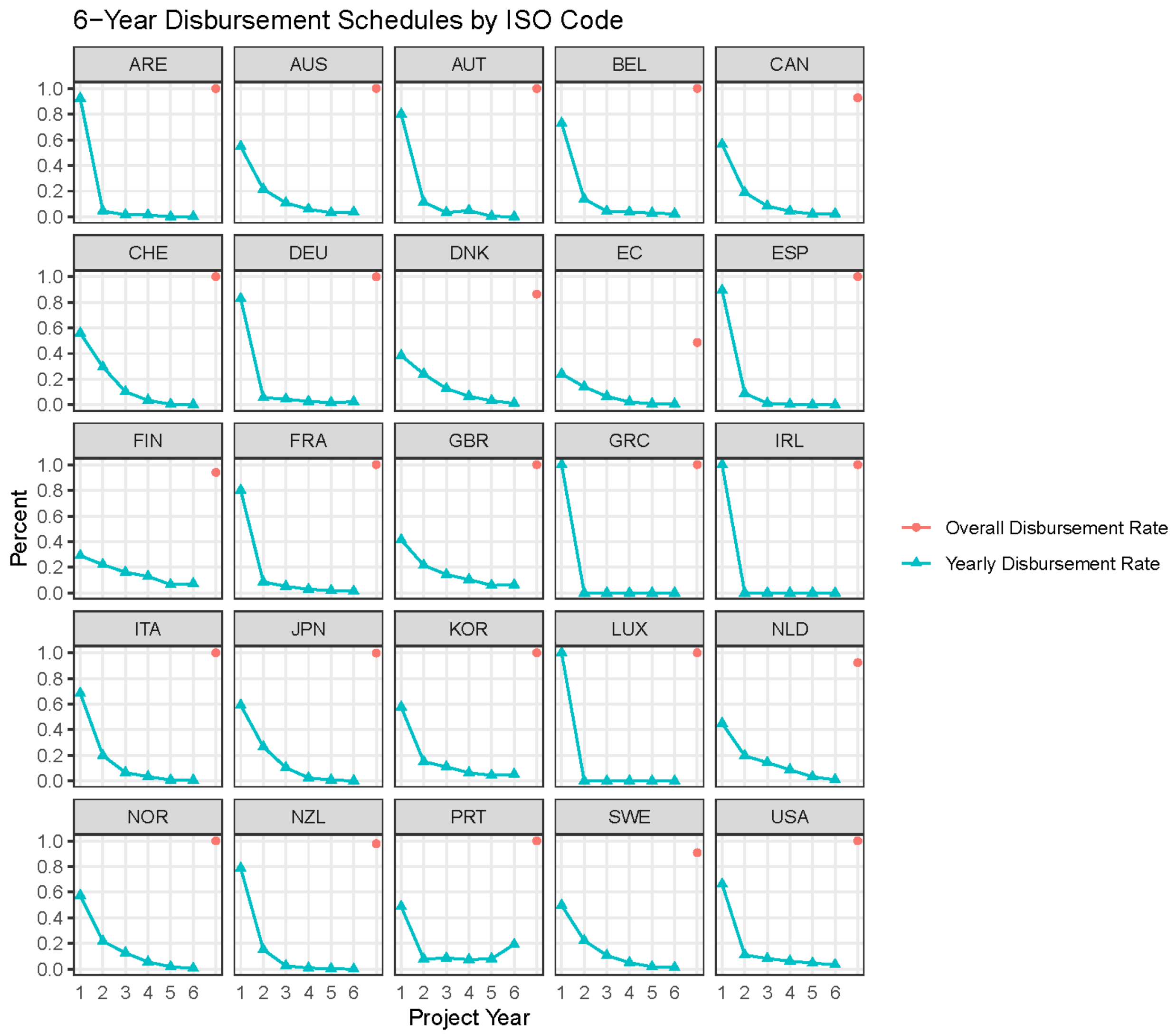
6−Year Disbursement Schedules by ISO Code
ARE
AUS
AUT
BEL
CAN
CHE
DEU
DNK
EC
ESP
FIN
FRA
GBR
GRC
IRL
ITA
JPN
KOR
LUX
NLD
NOR
NZL
PRT
SWE
USA
Percent
1.0
0.8
0.6
0.4
0.2
0.0
1 2 3 4 5 6
Project Year
Overall Disbursement Rate
Yearly Disbursement Rate

Box 3.4. EXAMPLE. Australia's one- to six-year disbursement schedules

To estimate disbursements using commitment data, we rely on disbursement schedules derived from CRS data that include both commitments and disbursements. Disbursement schedules are specific for each channel and the length of a project. These schedules also take into consideration the average amount of commitments for each channel that lead to disbursements. Across all Australian projects in the CRS with complete disbursements data, Australia disbursed 100% of the funds that it committed, as shown by the solid red dot on the right-hand side of the Australia panel (upper left corner of the first panel of Figure S3.3). In projects with a length of one year, Australia disbursed 100% of the funds that it committed in that year. For two-year projects, Australia disbursed 50% of total disbursements in year one and 50% of total disbursements in year two. In projects with lengths of three years, Australia disbursed about 50% of total disbursements in year one and 23% and 27% of total disbursements in years two and three, respectively. This is estimated for projects ranging from one to six years. The disbursement schedules were applied to commitment data from the CRS to estimate disbursements for years prior to the cutoff year, which is 2001 for Australia.

To predict DAH for the recent years not reported in the CRS, budget data were extracted from a variety of sources. These data are listed in Table S3.3. Global health budgetary data were utilized whenever possible, but these detailed data were available as a complete time series only for Australia and the United States. For all other bilateral channels, general ODA budgets were used. In order to predict DAH for 2021 for 24 bilateral agencies, the budget ratio for each donor was calculated by dividing DAH estimates by the corresponding budget data (ODA or global health). Budget ratios were projected using a weighted average of the previous three years (placing one-half weight on the one-year lagged ratio, one-third weight on the two-year lagged ratio, and one-sixth weight on the three-year lagged ratio), and this ratio was multiplied by the observed budgeted DAH for those same years. Figure S3.4 plots the budget ratio for each bilateral channel. Budget data for the EC were inconsistent and did not match the disbursement series. Instead, DAH for 2021 was estimated based on trends in DAH for EC member countries. A weighted average was applied to the percent change in DAH from 2018-2020 for all EC member countries. The weighting was based on each country's total national contributions to the EC. These data were collected from the EC's financial statements.[74] The weighted average was then applied to the EC's 2020 DAH to forecast 2021.

Figure S3.4 DAH as a percentage of corresponding budget data by bilateral agency

This figure shows the trend of the ratio of DAH measured as a share of budget data. Green dots indicate that a donor provided global-health-specific budget data, so in these cases the denominator is all global-health-specific budgeted data. The numerator is estimated DAH. Red dots indicate that a donor did not have global-health-specific budget data, so overall ODA budget data were used in calculating the DAH to budget ratios. The vertical axis represents estimated DAH as a fraction of corresponding budget data. ARE = United Arab Emirates, AUS = Australia, AUT = Austria, BEL = Belgium, CAN = Canada, CHE = Switzerland, DEU = Germany, DNK = Denmark, ESP = Spain, FIN = Finland, FRA = France, GBR = Great Britain, GRC = Greece, IRL = Ireland, ITA = Italy, JPN = Japan, KOR = South Korea, LUX = Luxembourg, NLD = the Netherlands, NOR = Norway, NZL = New Zealand, PRT = Portugal, SWE = Sweden, USA = United States of America.

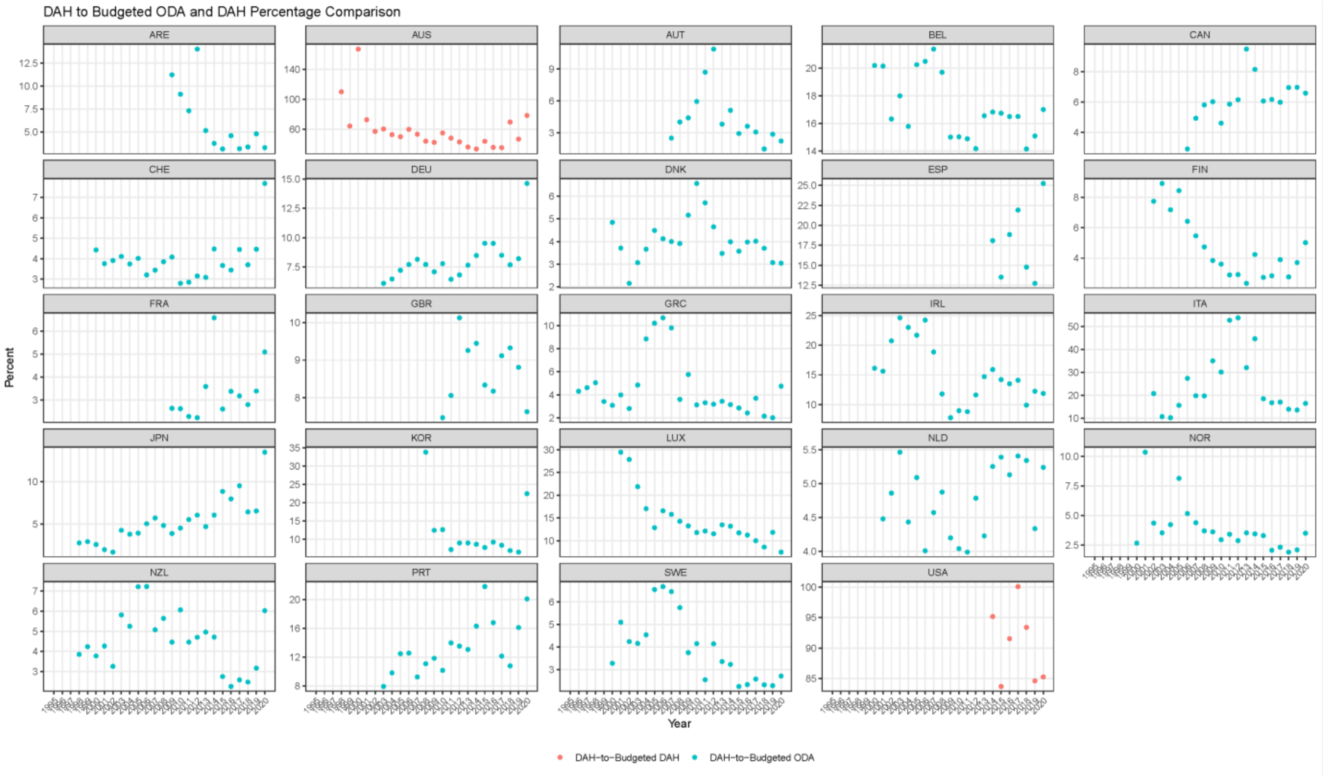

Source: IHME DAH Database (2021) and corresponding bilateral ODA/DAH budget documents outlined in Table S3.1 and 3.3.

Box 3.5. EXAMPLE. Australia's DAH as a percentage of corresponding budget data

Australia provided global-health-specific budget data for 1998-2021 through its International Development Assistance and Overseas Aid Program budgets. For 1998-2020, health ODA and observed DAH were used to create DAH to budget ratios. These budget ratios were then applied to 2020-2021 health ODA budget data to project DAH in 2021, using a weighted average:

$$(\boldsymbol{Total\ DAH_t}) = \left(\frac{\mathbf{1}}{\mathbf{2}}\right)(\boldsymbol{Budget\ ratio_{t-1}})\,(\boldsymbol{Budgeted\ GHE_t}) + \left(\frac{\mathbf{1}}{\mathbf{3}}\right)(\boldsymbol{Budget\ ratio_{t-2}})(\boldsymbol{Budgeted\ GHE_t}) + \left(\frac{\mathbf{1}}{\mathbf{6}}\right)(\boldsymbol{Budget\ ratio_{t-3}})(\boldsymbol{Budgeted\ GHE_t})$$

where t = year to be modeled.

To supplement our estimates of development assistance for health to HIV/AIDS and malaria program areas for the United States, we used additional available data from the President's Emergency Plan for AIDS Relief (PEPFAR) and the President's Malaria Initiative (PMI). We downloaded data on all planned funding by PEPFAR by recipient country, year, and program area from 2004 to 2021.[75] All PEPFAR projects were assigned to our eight HIV/AIDS program areas using PEPFAR budget codes, splitting out overhead costs equally to all other program areas. We then created country-year specific HIV/AIDS program area fractions out of total annual HIV/AIDS DAH, which we applied to all United States HIV/AIDS projects in the CRS from 2004 to 2020 by country-year. To inform malaria

funding by program areas, we downloaded the most recently available malaria funding tables from malaria operational plans for all countries and years.[76] We used a keyword search to assign each line item in these tables to our eight malaria program areas, and then created fractions for the malaria program areas out of the total annual malaria DAH specific to each country-year. These fractions were applied to all United States malaria projects in the CRS from 2006 to 2018 by country-year.

Figure S3.5 Malaria DAH to program areas as assigned by keyword search and PMI reports

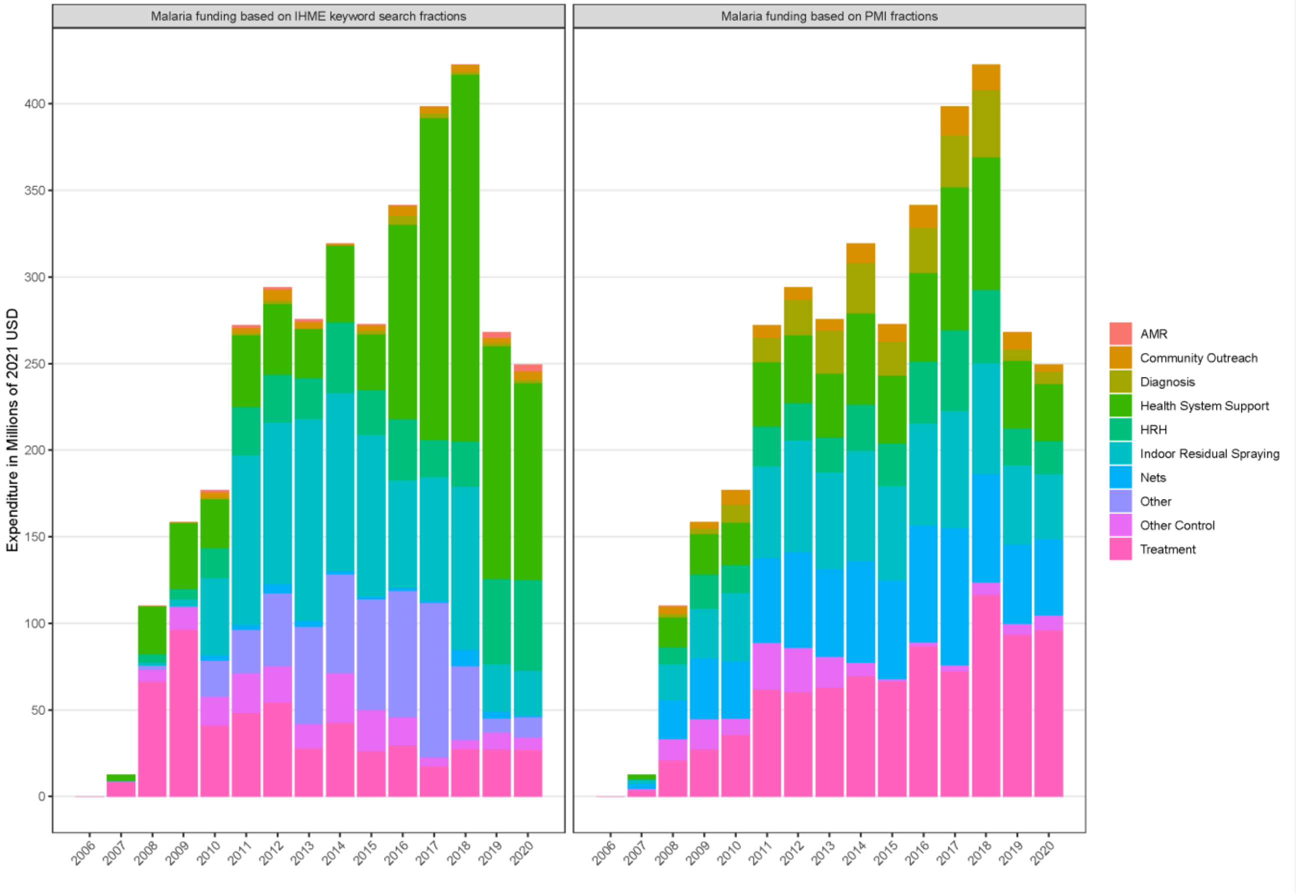

Source: IHME DAH Database (2021) and PMI malaria operational plans

This figure outlines the assignment of funding to malaria program areas for United States projects from the OECD CRS from 2006 onwards. The figure on the left shows how malaria funding is broken out based on keyword search. The figure on the right shows the breakdown of funding to malaria program areas based on PMI malaria funding tables. Using the data from PMI reduces the amount of funding allocated to the "other" program area. As such, in as often as more disaggregated information on project allocation is available, IHME uses such project information available in project budget documents or other project documents to disaggregate into program areas.

S3.3 Tracking development assistance for health from the development banks

The World Bank project-level health disbursement data for 1990 through December 2021 were obtained through correspondence with Miyuki T. Parris, .[77] The World Bank recently underwent a recoding process for their

disbursements. This recoding affected health disbursements, however the recoding was not completed for projects with disbursements prior to 2001. To create a comparable dataset adjustments had to be made. Regression analysis to predict health disbursements were explored, however, in the end the average percent change between project-level health disbursements before and after recoding was used to adjust health disbursements prior to 2001. It was observed that on average, between 2001 and 2005 (inclusive) the recoding process decreased health disbursements by 0.22%. This number was used to adjust all project-level health disbursements prior to 2001.[78] Health disbursements included all health projects as well as other sector projects with a health sector code. In addition, data were collected from the World Bank online loans database in order to fill in descriptive information for loans from the two arms of the World Bank: the International Development Association (IDA) and the International Bank for Reconstruction and Development (IBRD).[79] Along with keyword searches, health theme codes were used to allocate disbursements by health focus area. The online database contains up to five sector codes and five theme codes that can be assigned to each project. Sector codes represent economic, political, and social subdivisions, while theme codes represent the goals or objectives of World Bank activities. The codes are summarized in Table S3.6. Emergency recovery loans were excluded since they do not fit the definition of DAH.

Table S3.6 World Bank's health sector and theme codes

Health sector codes<br>*Sector codes represent economic, political, or social subdivisions within society. World Bank projects are classified by up to five sectors.*	*Health theme codes*<br>*Theme codes represent the goals or objectives of World Bank activities.*
Historic (prior to 2001):	Current:
(1)*Basic health*	(1) HIV/AIDS
(2)*Other population health and nutrition*	(2) Malaria
(3)*Targeted health*	(3) Tuberculosis
(4)*Primary health, including reproductive health, child health, and health promotion*	(4) Other communicable diseases<br>(5) Population and reproductive health
	(6) Child health
Current (as of 2001):	(7) Nutrition and food security
(1) Health	(8) Injuries and non-communicable diseases
(2) Compulsory health finance	(9) Health system performance
(3) Public administration – health	(10) Social analysis and monitoring
(4) Noncompulsory health finance	
(5) Health facilities and construction	

Data on yearly government contributions were obtained from the DAC statistics in order to disaggregate IDA flows by source. Because China does not report to DAC, we generated contribution estimates using replenishment data. We split the 3-year replenishment amount over the three years of the 16th-19th replenishment, to obtain China's contributions to the World Bank from 2008-2018. We validated our use of replenishment data by extracting China's and other countries' contributions to the World Bank International Development Association (IDA) 16th, 17th and 18th replenishment and comparing the number with the contribution we extracted from the OECD Creditor's Reporting System (CRS)[80]. The trend of contribution from major donors was similar, we therefore used China's replenishment contribution as a proxy of the contribution to World Bank IDA. We further disaggregated the DAH contribution to World Bank IDA from China by multiplying China's contributing proportion over World Bank IDA's total health envelope.

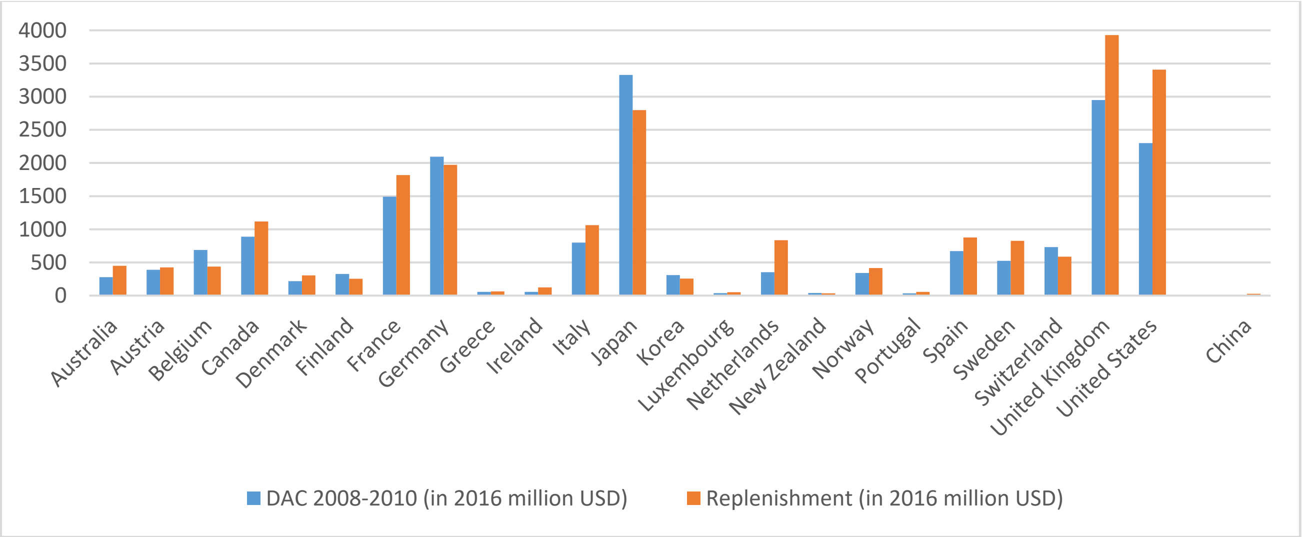

Panel a. Comparison of World Bank International Development Association 16th Replenishment

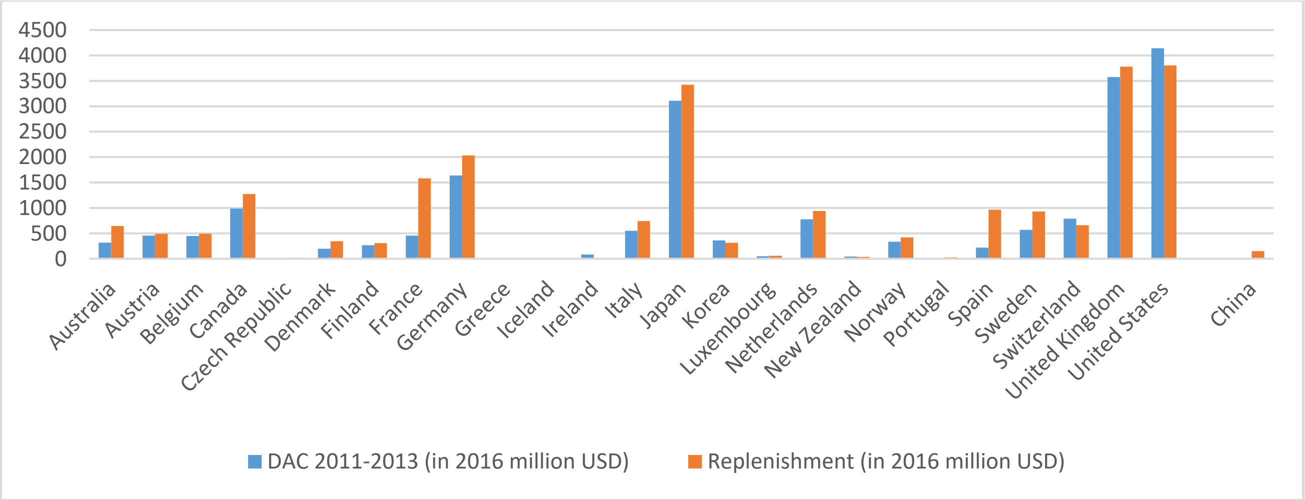

Panel b. Comparison of World Bank International Development Association 17th Replenishment

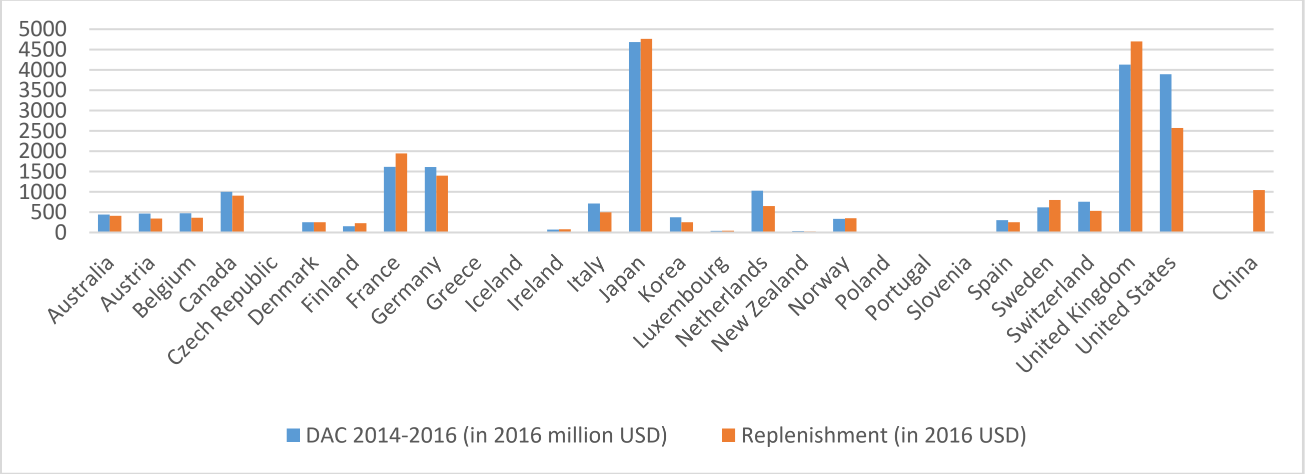

Panel c. Comparison of World Bank International Development Association 18th Replenishment

Figure S3.6 Comparison of World Bank IDA Replenishment and DAC

The three figures show the comparison of country commitment to World Bank IDA replenishment rounds, and yearly government contributions reported to DAC statistics. The orange line shows the country commitment to each IDA replenishment round. The blue line shows the sum of yearly reported contribution during the three-year replenishment round. Panel a is contributions and commitments from replenishment round 16; panel b is contributions and commitments from replenishment round 17; panel c is contributions and commitments from replenishment round 18.

Details on how we estimated the cost of providing technical assistance and program support for these institutions are highlighted below in the section titled "Calculating the technical assistance and program support component of development assistance for health from loan-and grant-making channels of assistance."

S3.3.1 Regional development banks

The Asian Development Bank (ADB) and Inter-American Development Bank (IDB) maintain their own loan databases, which we used to estimate disbursements.[15,16,81] To estimate health disbursements from the African Development Bank (AfDB), data was received via correspondence with Mr. Yomi Ayodabo (Division Manager, Loan accounting) .[35] To estimate health disbursement from the Asian Development Bank (ADB), data were webscraped from the online project database Table S3.7 provides a summary of the data sources used across the regional banks. Furthermore, Figure S3.6 displays the disbursements for AfDB from 1990 to 2021 and eFigures 3.7, and 3.8 display commitments and disbursements from 1990 to 2021 for ADB and IDB.

For AfDB, we received project level disbursement data from 2001 through 2021. For pre-2001 estimates, data from the Compendium of Statistics were used. Donor information for projects funded by the African Development Fund was based on subscription information from the 9th to 14th replenishment reports[82]. The sole donor to projects implemented by the Nigeria Trust Fund was Nigeria, and all income for African Development Bank projects was attributable to other sources.

For ADB, we received project-level disbursement data from 1990 through 2021, including recipient, project title and description and annual disbursement. A keyword search was performed to identify health focus areas of the projects. For income source of ADB, data for country donations were extracted from the replenishment reports of Asian Development Fund and we manually divide the four or five years' replenishment into annual contributions[83]. We extracted the total revenue of Ordinary Capital Resources and Asian Development Fund from the Financial reports of ADB to generate the total envelope of income sources and subtracted the country contribution to provide the estimate for resources coming from the bank itself. Using the fraction for the Bank's own resources and country contribution, we provided the estimate of income source for each project.

The IDB's project database also provided commitments for all projects. The same methods were used for estimating annual disbursements from the IDB as were used for the ADB. Through correspondence, 2021 IDB health loan disbursements were obtained in January 2021. These were used for the 2021 estimates. However, since these channels have so few new projects each year, we assume that smoothing disbursements over time for reported projects captures the majority of total disbursements for 2021. The contribution quotas by donor for the Fund for Special Operations from 1990-2016 were extracted from Annual Financial reports for the IDB. The ratio of each donor's contribution to total contribution was applied to total disbursements to assign income sources. In 2017, the Fund for Special Operations was transferred to the Ordinary Capital. Donor assignments from 2017 onwards are unallocable.

Table S3.7 Summary of data sources for the regional development banks

This figure indicates the data available and used to estimate DAH. (X) indicates that project-level data are present in the dataset. (-) indicates that project-level data are not present in the dataset.

Institution	*Data source*	*Commitments*	*Cumulative disbursements*	*Yearly disbursement*	*Notes*
African Development Bank (AfDB)	Compendium of Statistics	X	-	(Aggregate - not at the project level)	The Compendium of Statistics was not available for 1990-1993, 1995, 1998-1999, or after 2002; we estimated yearly disbursements using the average of neighboring disbursements
	Correspondence	-	-	X	Annual loan disbursements from 2001 through December 2021
Asian Development Bank (ADB)	Correspondence	X	X	X	Annual grant loan disbursements from 1990 through 2 February 2022
InterAmerican Development Bank (IDB)	Online projects database	-	X	-	As yearly disbursement amounts are not provided in the online database, we estimated yearly disbursements by allocating cumulative disbursements over each year of the project.
	Correspondence	-	-	X	Loan disbursements from January through December 2021

Figure S3.7 Disbursements by the African Development Bank

The orange line with squares shows estimated disbursements based on the Compendium of Statistics from 1990 through 2001 and actual disbursements received from 2001 onwards.

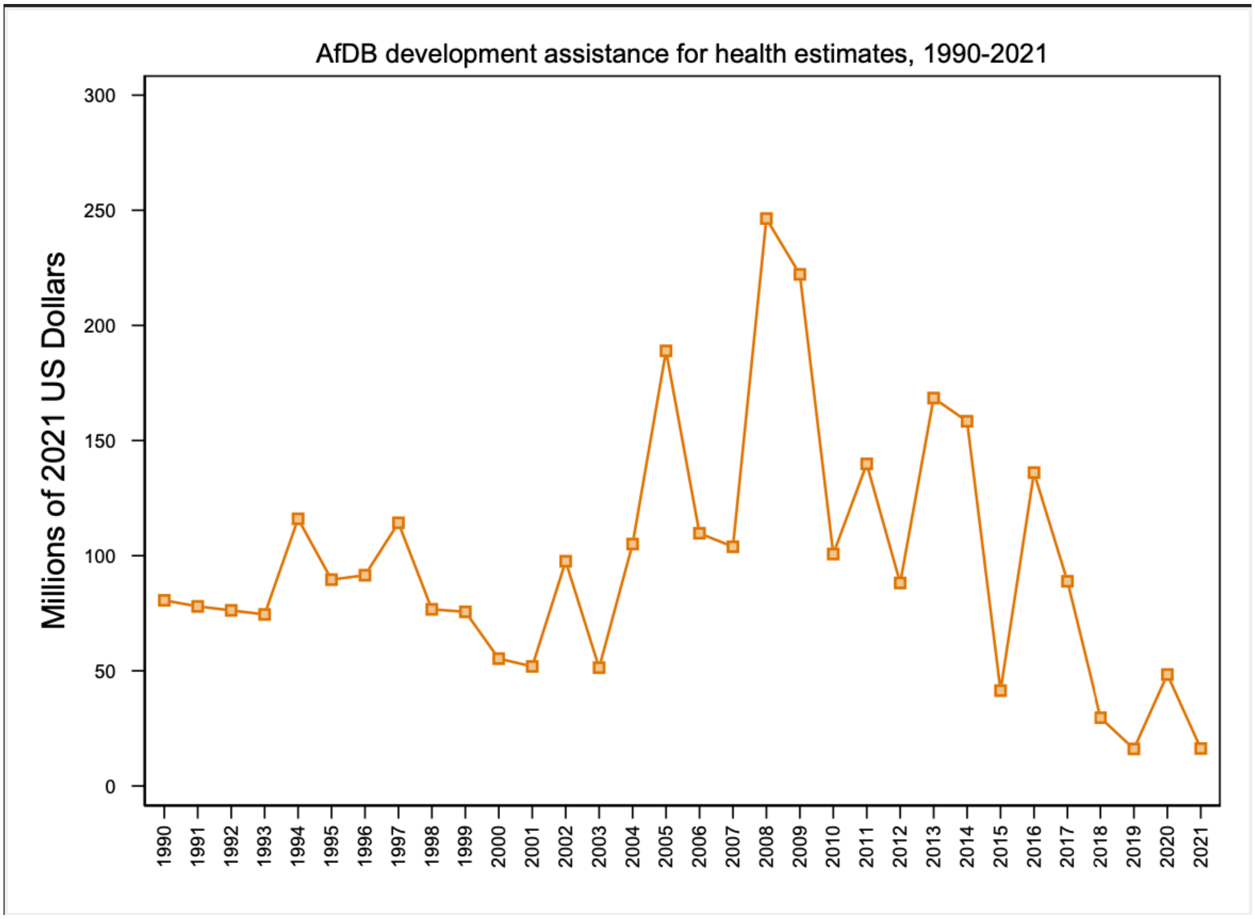

Source: IHME DAH Database (2021) and African Development Bank Compendium of Statistics.

Figure S3.8 Disbursements by Asian Development Bank

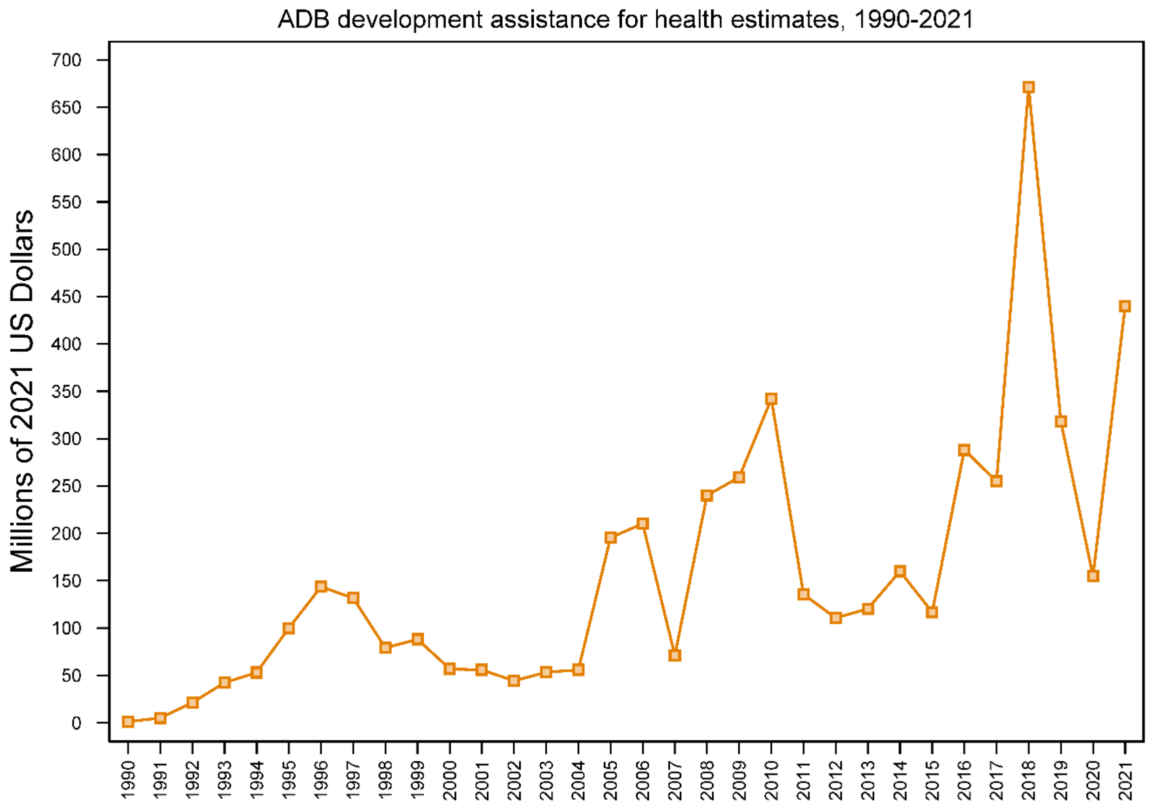

Source: IHME DAH Database (2021)

Figure S3.9 Disbursements by Inter-American Development Bank

The black line shows total disbursements from the Inter-American Development Bank's (IDB) online projects database. The blue line shows disbursements excluding administrative expenses. This data comes from the online projects database and from correspondence for 2021.

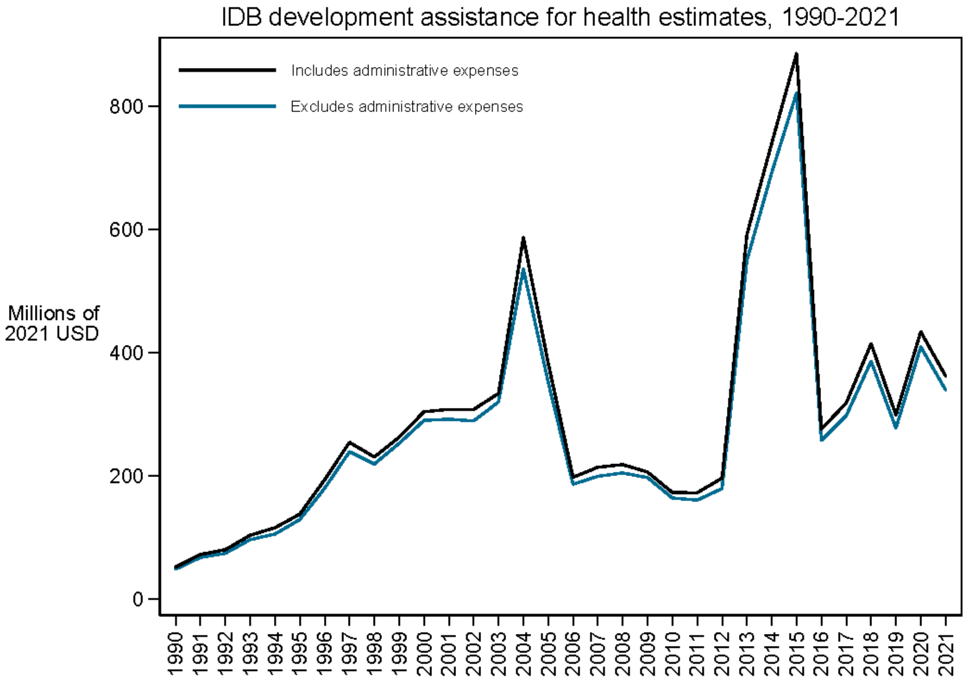

Source: IHME DAH Database (2021) and correspondence

S3.4 Tracking contributions from THE GLOBAL FUND and GAVI

The Global Fund to Fight AIDS, Tuberculosis and Malaria

The grants database made available online by the Global Fund to Fight AIDS, Tuberculosis and Malaria (THE GLOBAL FUND) provides grant-level commitments and annual disbursements from its inception in 2002 to the present year.[22] In addition, sources of funding were compiled from the THE GLOBAL FUND contributions dataset and annual reports, all downloaded from the THE GLOBAL FUND website.[23,24] Regional grants were split evenly between all countries identified in the regional grant documents found on the THE GLOBAL FUND website. Figure S3.10 shows THE GLOBAL FUND's annual contributions received from public and private sources. Figure S3.10 shows THE GLOBAL FUND's annual commitments and disbursements from its project database from 2002 through 2021.

Figure S3.10 Contributions received by the Global Fund to Fight AIDS, Tuberculosis and Malaria

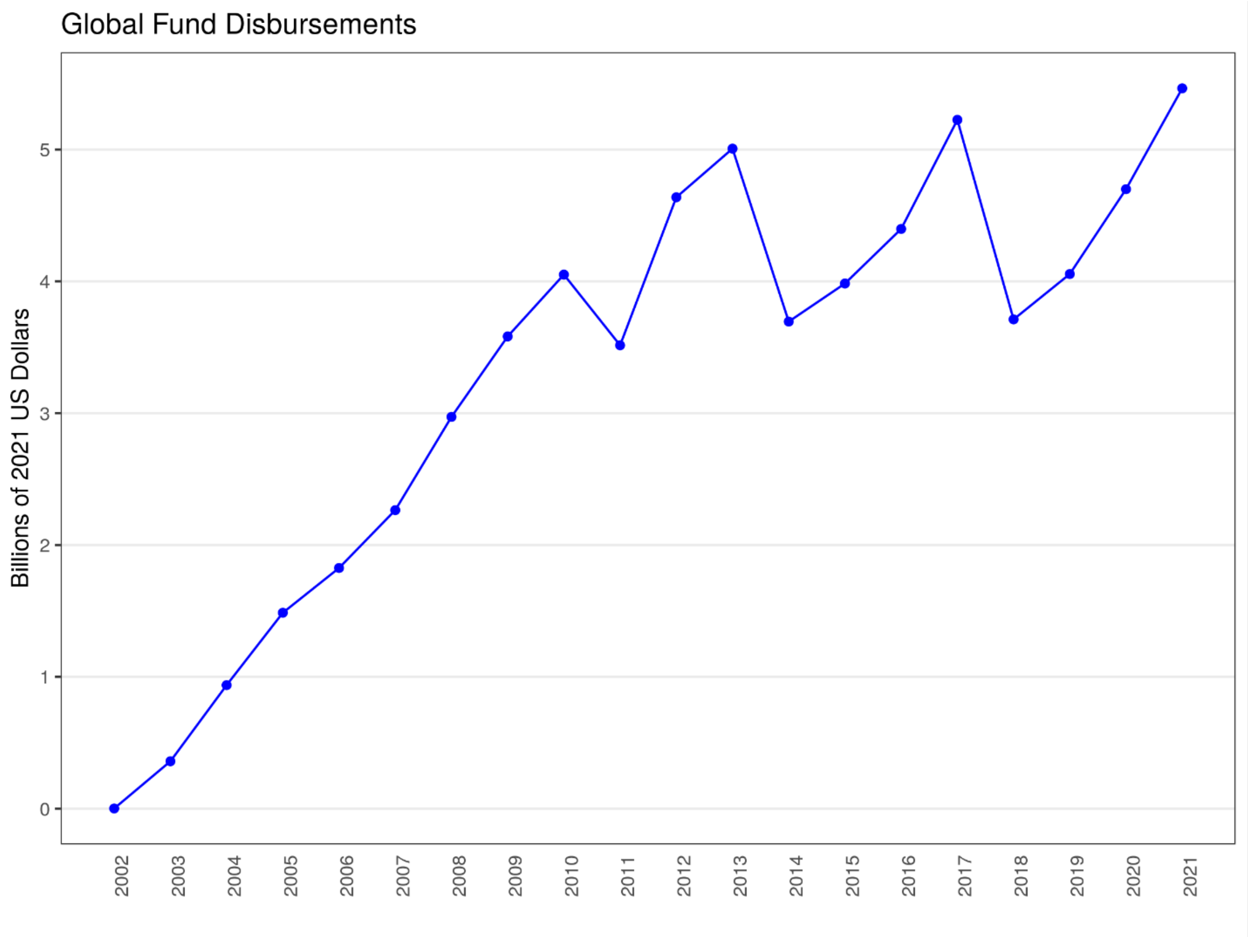

Source: THE GLOBAL FUND pledges and contributions 2021

Figure S3.11 The Global Fund to Fight AIDS, Tuberculosis and Malaria's commitments and disbursements

The dashed green line shows commitments from the Global Fund to Fight AIDS, Tuberculosis and Malaria's (THE GLOBAL FUND) online grants database. The orange line shows disbursements from the online grants database.

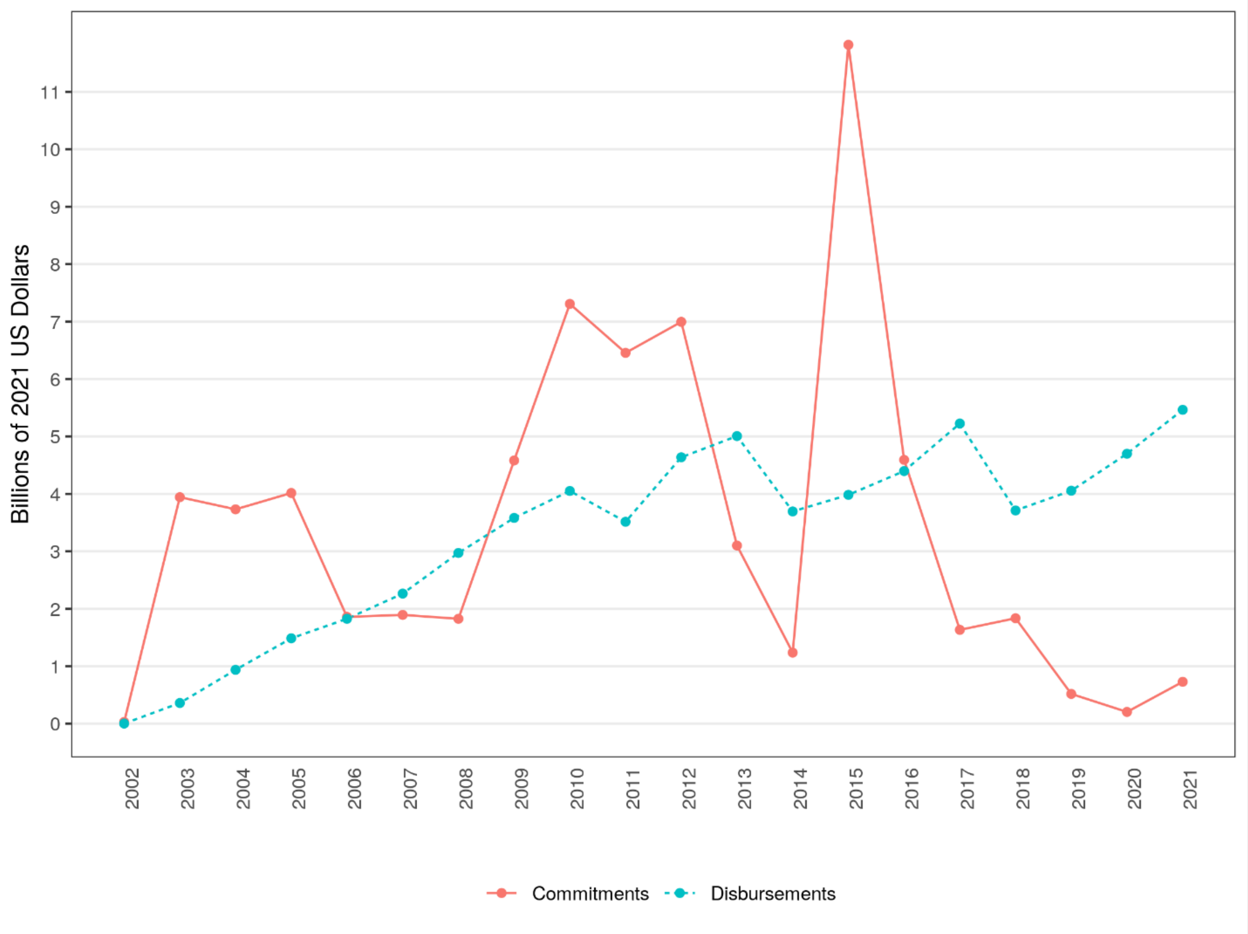

Source: IHME DAH Database (2021)

S3.4.1 Gavi, the Vaccine Alliance

Gavi provided publicly available project-level data on commitments, disbursements, and investment cases from 2000 through the present.[18,21] Gavi's annual DAH was defined as the sum of (1) project-level disbursements by year paid; (2) investment cases (one-time investments in disease prevention and control); and (3) administrative and work plan costs. Data from Gavi's online databases include expenditure for (1) and (2), but not (3). However, project level data from the CRS for 2007-2012 did include administrative and work plan costs, so disbursements data from the online database were adjusted to match the CRS in those years. The average fraction of administrative and work plan costs was added to total disbursements in 2000-2006 and 2013-2015, the years in which the CRS did not include these data. Contributions data from Gavi's website as well as annual reports from the International Finance Facility for Immunisation (IFFIm) and Advance Market Commitment for Pneumococcal Vaccines were used to determine Gavi's annual income.[19,20,84]

All of the data sources used for Gavi estimates were complete through 2021. Donor contributions received and outstanding pledges data were available on Gavi's website. Gavi disbursements were assigned to health focus areas including child and newborn vaccines, HSS, and non-communicable disease as documented in Table S3.7 above. Of note, we reclassified all GAVI health system strengthening projects as maternal, newborn, child health specific health system strengthening disbursements

Figure S3.12 Gavi's income and disbursements

The dashed orange line shows commitments from Gavi's online database. The dashed blue line shows the disbursements from Gavi's online database, which are the sum of project-level disbursements and investment cases. These data are adjusted using Gavi expenditure data reported to the Creditor Reporting System (CRS) to add administrative and work plan costs to the total. Adjusted disbursements are shown by the solid orange line.

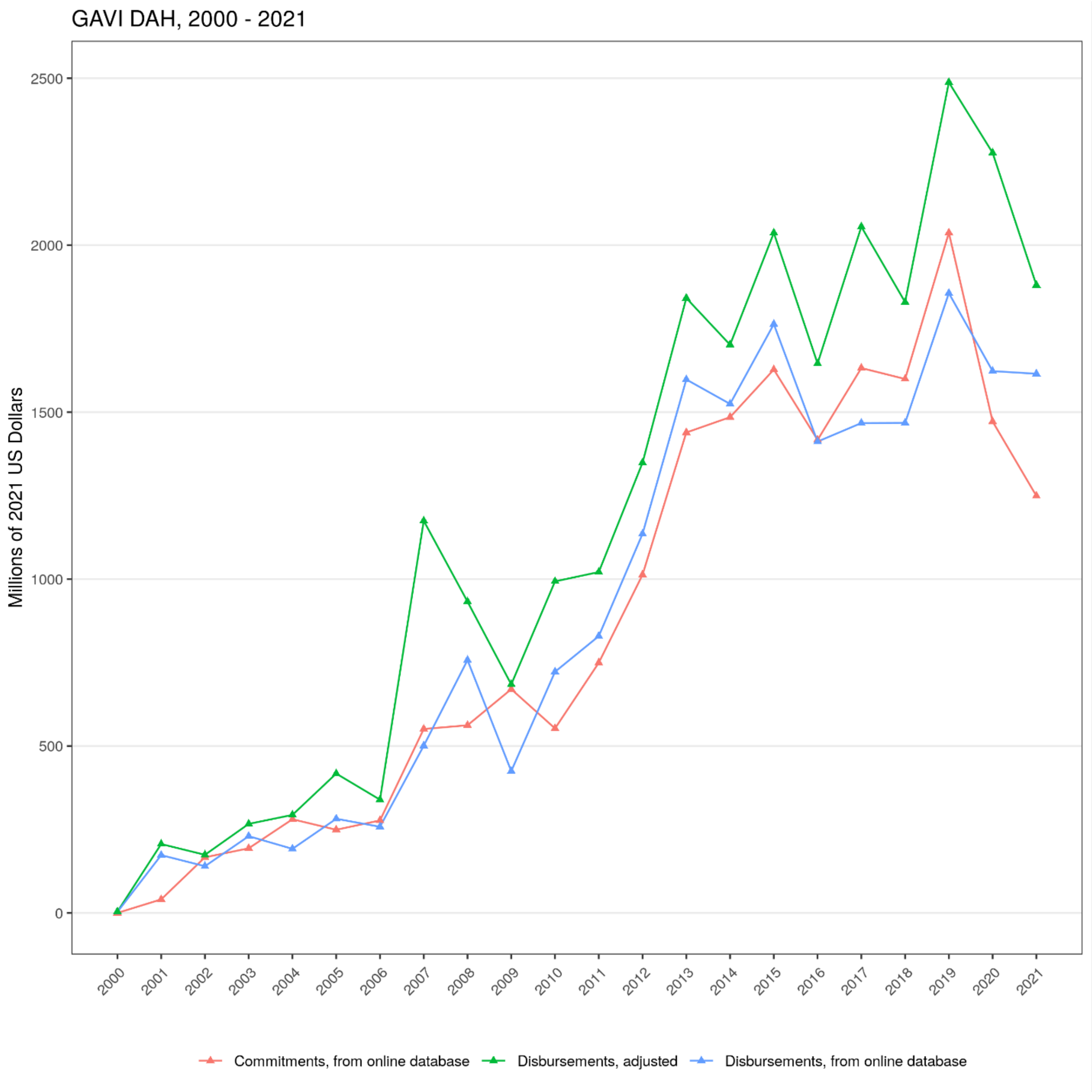

Source: IHME DAH Database (2021)

S3.4.2 Coalition for Epidemic Preparedness and Innovation

CEPI provided a complete dataset of all disbursed funding by disease from 2017-2021 and all income received from donors from 2018-2021. Funding data was provided per target vaccine and were classified between the other infectious diseases health focus area and the health systems strengthening other infectious diseases health focus area.

S3.5 Tracking expenditure by United Nations Agencies active in the health domain

Data on income and expenditures were collected for six UN agencies: WHO, UNICEF, UNFPA, UNAIDS, UNITAID and PAHO. The data sources and calculations for each are described in detail below. Similar to the bilateral channels, we extracted budget data for the UN agencies to predict DAH for years for which we did not have health expenditure data. Model choices and budget measures for UN agencies are presented in Table S3.3.

S3.5.1 World Health Organization
Data on WHO's budgetary and extrabudgetary income and expenditure were compiled from annual reports and audited financial statements released by WHO.[85] Income data were extracted from WHO's assessed and voluntary contributions, while expenditure data were extracted from both budgetary and extrabudgetary spending reports. For financial statements representing activities over a two-year period, both income and expenditure data were divided by two, in order to approximate yearly amounts, and dollars were deflated using the US GDP deflator specific to the reporting year. Expenditures from trust funds, regional offices tracked separately, and associated entities not part of WHO's program of activities, such as UNAIDS and THE GLOBAL FUND trust funds were excluded. Expenditures from supply
services funds were also excluded, as these expenditures pertain to services provided by WHO but paid for by recipient countries. Additionally, WHO projects tracked as paid Ebola expenditure were extracted from the UNOCHA Financial Tracking System database and included as WHO health expenditure.

Disbursement data were not available for WHO in 2020 and 2021. Much like the bilateral agencies, the ratio of DAH to the
total program budget was estimated for 1990-2019 and then predicted for 2020 and 2021 using a three year weighted average of 2017-2019. The predicted ratio was then multiplied by the observed program budget for 2020 and 2021 to get the estimates of DAH.
Assessed Contributions have been adjusted to 76% of reported values to follow the research conducted and published by OECD - Review of the ODA coefficients of select health-related organisations on Annex 2 and the Single Table of the Converged Statistical Reporting Directives for the Creditor Reporting System (CRS) and the annual DAC Questionnaire [100] . OECD estimate is negotiated with WHO as the HQ "has a partly normative function and provides support to all member countries, some of which are not ODA-eligible" [100].

S3.5.2 United Nations Population Fund

Data on income and expenditure were extracted for UNFPA from its audited financial statements.[72] As the 1990-2005 statements represent activities over a two-year period, income and expenditure data were divided by two in order to approximate yearly amounts.

Income and expenditures associated with procurement and cost-sharing activities were excluded from estimates of health assistance because UNFPA uses cost-sharing accounts when a donor contributes to UNFPA for a project to be conducted in the donor's own country. Since this money can be considered domestic spending that goes through UNFPA before being returned to the country in the form of a UNFPA program, it is not included in calculations of total DAH. UNFPA's additional expenditures for these projects come from trust funds or regular resources and are therefore captured in our estimates. To estimate disbursements by health focus areas, UNFPA's total health expenditure was multiplied by the proportion of funding reported for each program area from annual reports from 1997 through 2013, from the UNFPA transparency portal for 2014 through 2018, from the 2018-2019 Statistical and Financial Review for 2019-2020, and from the 2019-2020 Statistical and Financial Review for 2020-2021. Maternal

and child health spending classified as “other” was split equally between the maternal and child health program areas. Health spending in 2018 that was classified as towards “Integrated sexual and reproductive health services” was split equally between maternal health, family planning, other reproductive health and other HIV/AIDS program areas. Additionally, UNFPA projects tracked as paid Ebola expenditure were extracted from the UNOCHA Financial Tracking System database and added to UNFPA health expenditure.

The disbursement data for UNFPA were available through 2020. For year 2020 – 2021, the ratio of DAH and income was estimated for 1990-2020 and then predicted for 2021 using a three-year weighted average of 2018-2020. The predicted ratio was then multiplied by observed income to get the estimates of DAH.

S3.5.3 United Nations Children’s Fund

Data on income and expenditure for UNICEF were extracted from its audited financial statements.[69] As these statements represent activities over a two-year period from 1990-2011, income and expenditure data were divided by two in order to approximate yearly amounts. The audited financial statements from 2012 onwards are produced on an annual basis.

Since UNICEF’s activities are not limited to the health sector, the fraction of UNICEF’s expenditure that was for health was estimated using either financial data from correspondence (2001-2013 observed data was used to estimate 1990 through 2000 expenditure) or a combination of annual reports and annual results reports from 2014 through 2020. The annual results reports provide the proportion of funding for each program area and the average of 2014 through 2016 proportions was used to estimate the spending proportion for the years 1990 through 2013. In the annual results report, HIV/AIDS funding and Nutrition funding (as of 2014) was reported separately from health funding so the percentages spent on each health program were proportioned based on total spending for Health. Furthermore, UNICEF projects tracked as paid Ebola expenditure were extracted from the UNOCHA Financial Tracking System and added to estimates for UNICEF’s health expenditure. Since 2020 data was not project level we scaled down the total envelope for this year by the 2020 UNICEF DAH for COVID envelope in order to avoid double counting.

The product of observed program budget and the three-year weighted average of the DAH to budget was used to predict DAH in 2021.

S3.5.4 Joint United Nations Programme on HIV/AIDS

UNAIDS income and expenditure data for both its core and noncore budgets were extracted from its audited financial statements.[67] As financial data are provided on a biennial basis in all years prior to 2012, those values were divided by two to obtain yearly amounts. Dollars were deflated using the US GDP deflator specific to the reporting year.

For UNAIDS, budget measures were available only for a subset of reported total disbursements. UNAIDS reported total expenditure, which combined Unified Budget and Workplan (UBW) and non-UWB components, but only UBW budget data were available.[68] To predict DAH for UNAIDS in 2021, disbursements for that year were estimated by multiplying the observed UBW budget by the three-year weighted average of the ratio of DAH to the UWB budget. UNAIDS disbursements were assigned to HIV/AIDS and TB program areas as documented in Table S3.7 above. Since 2020 data was not project level we scaled down the total envelope for this year by the 2020 UNAIDS DAH for COVID envelope in order to avoid double counting.

S3.5.5 UNITAID

Data on project level disbursement was obtained through correspondence with UNITAID. Income data was extracted from the annual financial statements downloaded from UNITAID’s website. The project level data

provided covered project disbursements from 2007 through 2021 and project budget commitments was provided for 2021.

S3.5.6 Pan American Health Organization

The Pan American Regional Office for WHO, or PAHO, reports its income and expenditure in its biennial financial report.[10,86] The funds transferred through the “Rotating Fund” were excluded because developing countries fund this procurement of health commodities which are then used within that funding country , and it therefore does not fit the definition of DAH.

As the financial data are provided on a biennial basis (with the exception of 2010 through 2016, where single-year financial reports were available), the quantities were divided by two to obtain yearly amounts. Dollars were deflated using the US GDP deflator specific to the reporting year.

Correspondence with PAHO revealed that data from the financial statements include both Program and nonProgram funds. The latter include funds that countries provide PAHO, so that PAHO can reinvest these funds into the countries’ national health systems. These funds should not be included as development assistance for health, and PAHO provided corrected disbursement numbers for 2008 to 2013. The corresponding disbursement numbers for 2014 and 2015 were identified in the PAHO End-of-Biennium Assessment 2014-2015. These funds were provided as biennial disbursements, so they were divided by two to obtain yearly disbursements. The ratio of Program disbursements numbers provided by PAHO and the sum of Program and non-Program funds collected from financial statements was taken for the years 2008 to 2015. The average ratio was calculated, and this ratio was multiplied through disbursement numbers collected from financial statements from earlier years. In this way, Program and non-Program funds collected from audited statements from earlier years were adjusted to estimate DAH.

For PAHO, disbursement data were not available for 2021. PAHO provided budget information along with disbursements for 2008 to 2020. The average ratio between spending and budget was calculated over the years 2008 to 2020, and this ratio was used to estimate 2021 disbursements.

S3.6 Tracking development assistance for health from private foundations

Previous studies on foundations outside the US have documented the severe paucity of reliable time series data and lack of comparability across countries.[87] Hence, this research focused efforts on tracking only US foundations.

S3.6.1 US Foundations

The Foundation Center maintains a database of all grants of $10,000 or more awarded by over 1,000 US foundations. The Foundation Center has coded each grant by sector and international focus and therefore is able to identify global health grants. IHME purchased a customized dataset with cross-border health grants and health grants to US-based international programs from 1992 to2019 from the Foundation Center.[32] Grants from BMGF, which were tracked separately, were excluded. Additionally, grants to channels that this research already tracks were excluded.

The Foundation Center adopted a new classification methodology as of FGH 2016. The Foundation Center was able to provide historic data based on the new classification system from 2002 to 2012. In order to obtain the series from 1992 to 2001, we multiplied a weighted fraction calculated based on both old and new classification data values from 2002 through 2004 by the old data series (1992 to 2001) we had previously obtained.

$$
\begin{aligned}
&(Weighted\ fraction) \\
&\quad = \left(\frac{1}{2}\right)\left(DAH_{new\ classification}\right)/\left(DAH_{old\ classification}\right)2002 \\
&\quad + \left(\frac{1}{3}\right)\left(DAH_{new\ classification}\right)/\left(DAH_{old\ classification}\right)2003 \\
&\quad + \left(\frac{1}{6}\right)\left(DAH_{new\ classification}\right)/\left(DAH_{old\ classification}\right)2004
\end{aligned}
$$

$$(DAH\ Estimate_t) = (Weighted\ fraction)(DAH\ Observed_t)$$

where DAH Observed is the old data values for the series 1992 through 2001

To estimate total health grants in 1990-1991 and 2020-2021, natural log of US foundation DAH was regressed on the lagged natural log of US GDP per capita and year using ordinary least squares estimation. The missing years of data were predicted based on estimated regression coefficients from the equation. Exponents of the predicted values were used as final estimates

$$(ln\ Foundation_t) = \alpha + 1.\beta_1(\ln US\ GDP\ per\ capita_t) + \beta_2(year_t) + \varepsilon$$

Details on how we estimated the cost of providing technical assistance and program support for these US foundations are highlighted below in the section titled calculating the technical assistance and program support component of development assistance for health from loan-and grant-making channels of assistance.

S3.6.2 Bill & Melinda Gates Foundation

BMGF has been the single largest grant-making institution in the health domain since 2000; hence, additional research was undertaken to accurately capture its annual disbursements. BMGF's IRS 990PF filings for years 1999-2008, which report all global health grants disbursed per year, were downloaded from the BMGF website. Additionally, disbursement data for years 2009-2021 were collected from the BMGF online grants database, the OECD CRS and personal correspondence. The OECD CRS data was used to identify NGOs that are double-counted from other data sources.

An ordinary least squares linear regression model was used to predict the disbursement proportion by healthcare focus area for BMGF for 2021. Since there is a strong correlation between market trends and BMGF annual disbursements, market data including lagged US GDP, lagged yearly average of Berkshire stock returns, lagged yearly average of the Russell Index, and lagged total assets of the BMGF Trust were utilized to predict the total disbursement for year 2020.

$$
\begin{aligned}
&(BMGF\ total\ disbursement_t) \\
&\quad = \alpha + \beta_1\left(US\ GDP\ per\ capita_{t-2}\right) + \beta_2(Berkshire\ stock\ returns_{t-2}) \\
&\quad + \beta_3(Russell\ Index_{t-2}) + \beta_4(BMGF\ total\ asset_{t-2}) + \varepsilon
\end{aligned}
$$

BMGF's predicted DAH was adjusted to account for in-kind DAH and double-counting, and the grants database was used to adjust the 2021 predicted envelope based on the yearly trend in overall commitments for global health projects from 2020 to 2021. The difference between BMGF's final DAH and DAH without in-kind added and double-counting removed from 2003-2019 was regressed using ordinary least squares on DAH without in-kind

added and double-counting removed and year. The predicted difference was then subtracted from the predicted DAH from the previous regression for 2020.

S3.6.3 European Economic Area

DAH estimates for the European Economic Area were based on two downloadable project datasets available online. IHME DAH data for 2014 to 2018 was obtained from projects in the 2009-2014 grant period dataset with programme area "public health initiatives", and 2007-2012 data was obtained from projects in the 2004-2009 grant period dataset with sector "health and childcare". Childcare projects not related to health were identified and excluded based on manual screening and keyword search for the following terms: "NURSERY" "ORPHANAGE" "RECREATION" "FOSTER" " CARE HOME " " CHILDREN S HOME " " CHILD DEVELOPMENT CENTRE " " YOUTH CENTER" " YOUTH CAMPS " " RESIDENTIAL CENTRE" " RESIDENTIAL CARE FACILITIES " "CHILDCARE" " INFORMAL EDUCATION FOR CHILDREN " " PREVENT INSTITUTIONALISATION " " SPORTS FIELD" " SPORT FIELD" " SPORTS FACILIT" " SPORTS ACTIVIT" " SPORTS INFRASTRUCTURE" " REINTEGRATION OF JUVENILE OFFENDERS "

We determined that no health projects to low- and middle-income countries were implemented by the EEA prior to the 2004-2009 grant period, based on the report on the Financial Instrument 1999-2003 and the Financial Mechanism 1994-1998.[88,89] Project summary variables were obtained through correspondence, and were used together with project titles to assign DAH to health focus areas using a keyword search. To assign annual funding for projects that were implemented across multiple years, project grants were split based on the average daily spending across the duration of the project and the proportion of the year that the project was in implementation. The relative contributions of donors to the EEA grants (Norway, Iceland, and Liechtenstein) were obtained as constant ratios based on the 2016-2017 Annual Report.[90]

An open call for projects in the EEA's third grant period occurred in 2014, the first projects of which began disbursing in 2019 and are set to conclude in 2021. Data for the 2019-2021 grants came from personal correspondence. For 2021, our correspondence reported only grants for high income countries and therefore we recorded no DAH for EEA this year.

S3.7 Tracking non-governmental organisations

Currently, there are no centralized, easily accessible databases for tracking program expenses of the thousands of NGOs based in high-income countries that are active in providing development assistance and humanitarian relief worldwide. This study relied on CRS data and the only comprehensive data source identified for a large subset of these NGOs, namely the United States Agency for International Development's Report of Voluntary Agencies (USAID's VolAg report).[27] The report, which includes both US-based and international NGOs that received funding from the US government, provides data from 1990 to 2014 on domestic and overseas expenditures for these NGOs as well as their revenue from US and other public sources, private contributions, and in-kind. Total revenue and expenditure data obtained from the NGOs' IRS tax forms, accessed through the GuideStar online database, were also used in tracking NGOs incorporated in the US.[26] The GuideStar Research Fundamentals Plus dataset was used to obtain information for US-based NGOs in fiscal years 2015-2018, including domestic, overseas, and total expenses, and revenues from all public and private sources.

First, in order to track disbursements from OECD donor countries to NGOs, we utilized channel codes present in the CRS database. The code 21000 identified international NGOs and the code 22000 identified donor-country-based NGOs. In order to remove double-counting, we conducted a keyword search on channels where the donor country was the United States to exclude NGOs present in the USAID VolAg report and the GuideStar dataset. Allocation of funding to health focus areas for NGOs tracked through the CRS was assigned as described in the section "DISAGGREGATING BY HEALTH FOCUS AREA", based on a keyword search of five descriptive variables in the CRS: project title, short description, long description, channel name, and channel reported name. For NGOs tracked in the USAID VolAg report and the GuideStar dataset, allocation of funding to health focus areas was

assigned as described in the section “DISAGGREGATING BY HEALTH FOCUS AREA”, based on a keyword search of the NGO’s description given in the VolAg report. Due to CRS projects containing more complete descriptions than the VolAg report descriptions, the health focus area allocations from CRS were prioritized over those from the VolAg report for US NGO agencies that appeared in both sources.

In order to use the USAID VolAg data and the GuideStar dataset, several challenges were overcome. We outline these challenges here and discuss below the methods employed to estimate a consistent series of DAH channeled through NGOs despite these challenges. First, with the exception of BMGF, it was impossible to track the amount of funding from US foundations routed through US NGOs, which may have led to double-counting in estimates of total health assistance. The second challenge relates to the incompleteness of the universe of NGOs captured through the VolAg report and the GuideStar Research Fundamentals dataset. The VolAg report provides data on NGOs that received funding from the US government. While this covers many of the largest NGOs, it is not a comprehensive list. A related problem is that the VolAg report and GuideStar dataset only include NGOs that received funds in a given year. While many of the largest NGOs are consistently funded by the US government and are therefore in the dataset every year, not all NGOs are reported across all years. Third, health sector-specific expenditure is not reported in the VolAg or systematically reported in IRS tax forms. The VolAg does report overseas expenditure but does not disaggregate this expenditure by sector. Fourth, complete data are lacking in several time periods. The 2016 VolAg provided data through 2014. For NGOs incorporated in the US, IRS tax forms were obtained. Furthermore, prior to 1998 the VolAg report did not include international NGOs. Attempts were made to compile other data on the health expenditures of the top international NGOs, in terms of overseas expenditure, by searching other websites for financial documents and contacting these organizations directly. Getting reliable time series data before 2000 proved to be extremely difficult for even this small sample of international NGOs.

Estimates of the share of overseas expenditure spent on health-related projects drew upon a sample of NGOs for which such data were available. Collecting financial data on health expenditures for each NGO would have been prohibitively time-consuming. Therefore, a sample of NGOs was drawn from the list for each year; the sample included the top 30 NGOs in terms of overseas expenditure and 20 randomly selected US-based NGOs from the remaining pool, with the probability of being selected set proportional to overseas expenditure. Next, health expenditure data were collected for each NGO in this sample by seeking out annual reports, audited financial statements, 990 tax forms, and data from NGO websites. Health expenditure was carefully reviewed to ensure that expenditures on food aid, food security, disaster relief, and water and sanitation projects were not included. Table S3.8 summarizes the number of NGOs included each year in the USAID report, the number of NGOs in the sample by year, and the number of NGOs for which health expenditure data were successfully compiled.

Table S3.8 Summary of US non-governmental organizations in the study

Year	*Number of US NGOs in VolAG report*	*Number of international NGOs in VolAG report*	*Number of US NGOs in IHME sample*	*Number of US NGOs from sample for which data on health expenditure were found*
1990	267	-	16	9
1991	334	-	19	14
1992	385	-	18	15
1993	411	-	17	12
1994	424	-	17	10
1995	416	-	16	12
1996	423	-	21	14
1997	425	-	23	18
1998	435	42	24	22
1999	438	-	33	28

2000	433	50	34	28
2001	442	51	33	26
2002	486	58	33	27
2003	507	54	42	32
2004	508	55	47	33
2005	494	59	45	36
2006	536	67	50	38
2007	556	68	50	40
2008	565	78	58	48
2009	580	90	57	45
2010	579	94	69	57
2011	595	112	73	63
2012	579	94	69	60
2013	519	113	69	52
2014	485	106	73	54
2015	-	-	67	53
2016	-	-	115	98
2017	-	-	81	69
2018	-	-	109	93

A random effects regression model was fit to predict health expenditure as a fraction of total expenditure using the data for the sampled NGOs. A random effects model was chosen because the sample included observations for several NGOs for multiple years. A random effects model allows for the effect of each type of NGO to be captured distinctly. This model was used to predict the fraction of expenditure spent on health for the remaining NGOs. To ensure that the predicted health fractions were bounded between zero and one, the regression utilized the logit-transformed health fraction as the dependent variable. Since several NGOs in the sample were observed for multiple years, the regression included a random effect that varied by NGO. Five of the nine variables used to predict the health fraction were drawn from the VolAg reports and the GuideStar dataset. They were (1) fraction of revenue from in-kind donations, (2) fraction of revenue from the US government, (3) fraction of revenue from private financial contributions, (4) overseas expenditure as a fraction of total expenditure, and (5) calendar year. The remaining four variables used to predict the health fraction were binary indicators that were constructed based on keyword searches on the NGO name and NGO description found in the VolAg. For both the NGO name and description, a keyword search was conducted to indicate whether the name or description was sufficiently health-related. Another keyword search was conducted independently on the NGO names and descriptions for keywords that indicated if the NGOs might focus on something other than health. These four indicators proved excellent predictors of health fractions.

$$\begin{aligned} logit(\,NGO - specific\ DAH_{it}) &= \alpha + \beta_1(Inkind\ contributions\ fraction_{it}) \\ &+ \beta_2(US\ government\ contributions\ fraction\ _{it}) \\ &+ \beta_3(Private\ financial\ contributions\ fractions_{it}) \\ &+ \beta_4(Overseas\ expenditure\ as\ a\ fraction\ of\ total\ expenditure_{it}) \\ &+ \beta_5(Health - related\ name_{it}) + \beta_6(Non - health - related\ name_{it}) \\ &+ \beta_7(Health - related\ description_{it}) + \beta_8(Non - health - related\ desciption_{it}) + U_i + \varepsilon \end{aligned}$$

Overseas health expenditure was calculated for individual NGOs in each year by multiplying the estimated health fraction and total overseas expenditure. For the NGOs that were sampled, the observed health fraction acquired through data collection was used. For the unsampled NGOs, the fitted fraction from the previously described random effects regression was used. Total overseas expenditure, reported in the VolAg, was not available for 2015-

2020. For US-based NGOs, the 2018 NGO overseas fraction was calculated by regressing the logit transformed observed overseas fraction on a linear time trend using ordinary least squares, for each NGO independently. For these cases, the overseas health fraction was calculated as the product of estimated overseas fraction, estimated health fraction, and total expenditure found in the IRS 990 forms.

$$logit(Observed\ overseas\ health\ expenditure_i) = \alpha + \beta_i(year_t) + U_i + \varepsilon$$

At this point three reasons remained why the overseas health expenditure for some NGOs remained unknown. First, if an observation was non-US-based, then IRS tax forms were not available and total overseas expenditure could not be calculated. Second, for 2019 to 2021, no data were available. Finally, if an NGO was reported in the VolAg in multiple years but not for an intermittent year, no NGO-specific data were available for the gap year. This would be the case if an NGO received support from the US government one year and then again in a nonconsecutive year. For all three of these scenarios, a panel-based hierarchical linear regression model was used to fill in the overseas health expenditure gaps. Total overseas health expenditure (measured at the NGO-year level) was regressed on US GDP per capita and US bilateral DAH disbursed. Because the US government funds many of these NGOs, US bilateral DAH was an excellent predictor of NGO DAH. A flexible model was employed to allow both the GDP and US government DAH coefficients to vary randomly across NGOs, such that each NGO employed a unique (but not independent) relationship between overseas health expenditure, GDP, and US government DAH. A random intercept was also included to capture the significant unobserved heterogeneity present in our set of NGOs. Once fit, this model was used to predict overseas health expenditure for all remaining gaps.

$$(\ NGO\ DAH_{it}) = \alpha + \beta_{1i}(US\ GDP\ per\ capita_t) + \beta_{2i}(US\ bilateral\ DAH\ per\ capita_t) + U_i + \varepsilon$$

Expenditures financed from each revenue source were then calculated by multiplying overseas health expenditure by NGO-specific revenue fractions. Expenditures from in-kind sources were deflated by a constant fraction. This was determined by comparing the federal upper limit and average wholesale price valuations of drugs on the WHO's Model List of Essential Medicines from the RED BOOK Expanded Database.[28,29] Figure S3.13 and Figure S3.14 show the income and estimated overseas health expenditure, respectively, of the NGOs in the universe of US- and non-US-based NGOs that were tracked in this study from 1990 to 2018 in constant 2020 US dollars.

Figure S3.13 Total revenue received by non-governmental organizations

The orange line shows total revenue for all sources, both public and private, received by NGOs. The green line shows estimates of private financial contributions to NGOs, while the blue line shows private in-kind donations to NGOs.

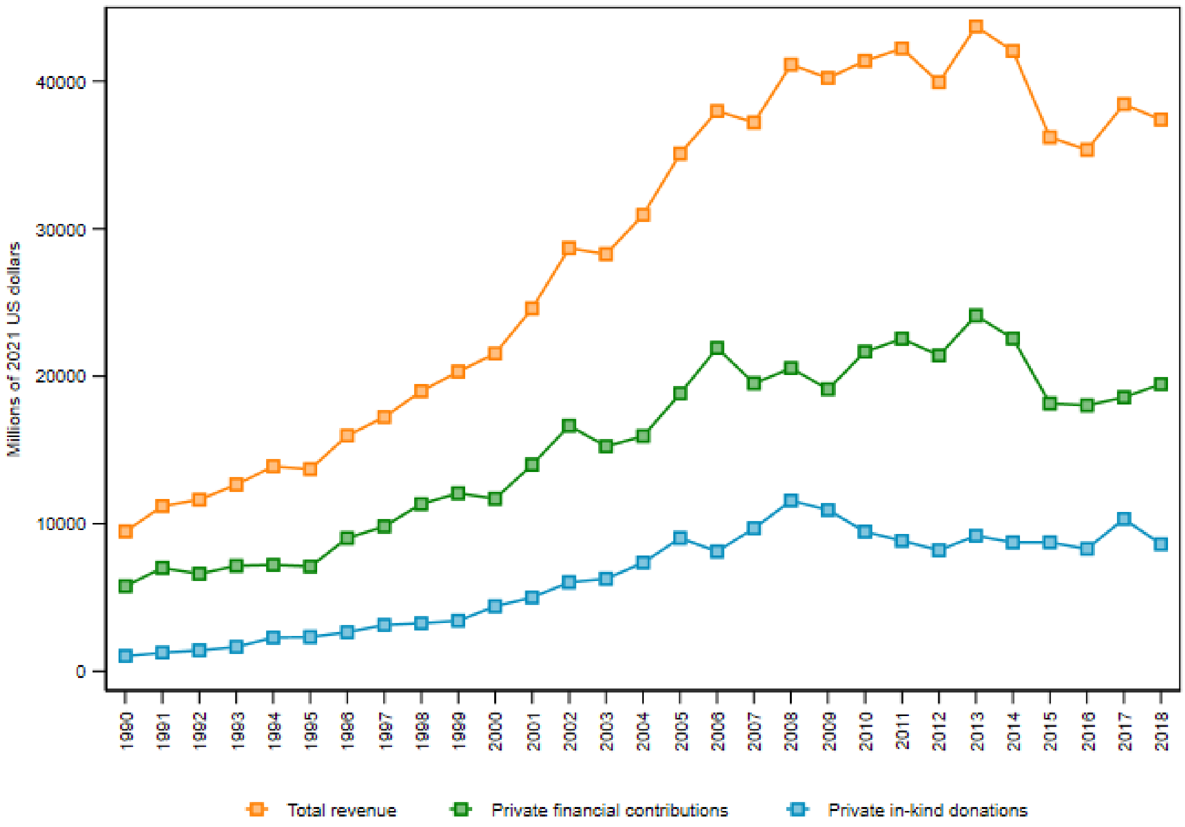

Source: IHME DAH Database (2021)

Figure S3.14 Expenditure by non-governmental organizations

The orange line illustrates total overseas expenditure by NGOs, regardless of sector. The green line shows overseas expenditure by NGOs to health-specific recipients, or DAH.

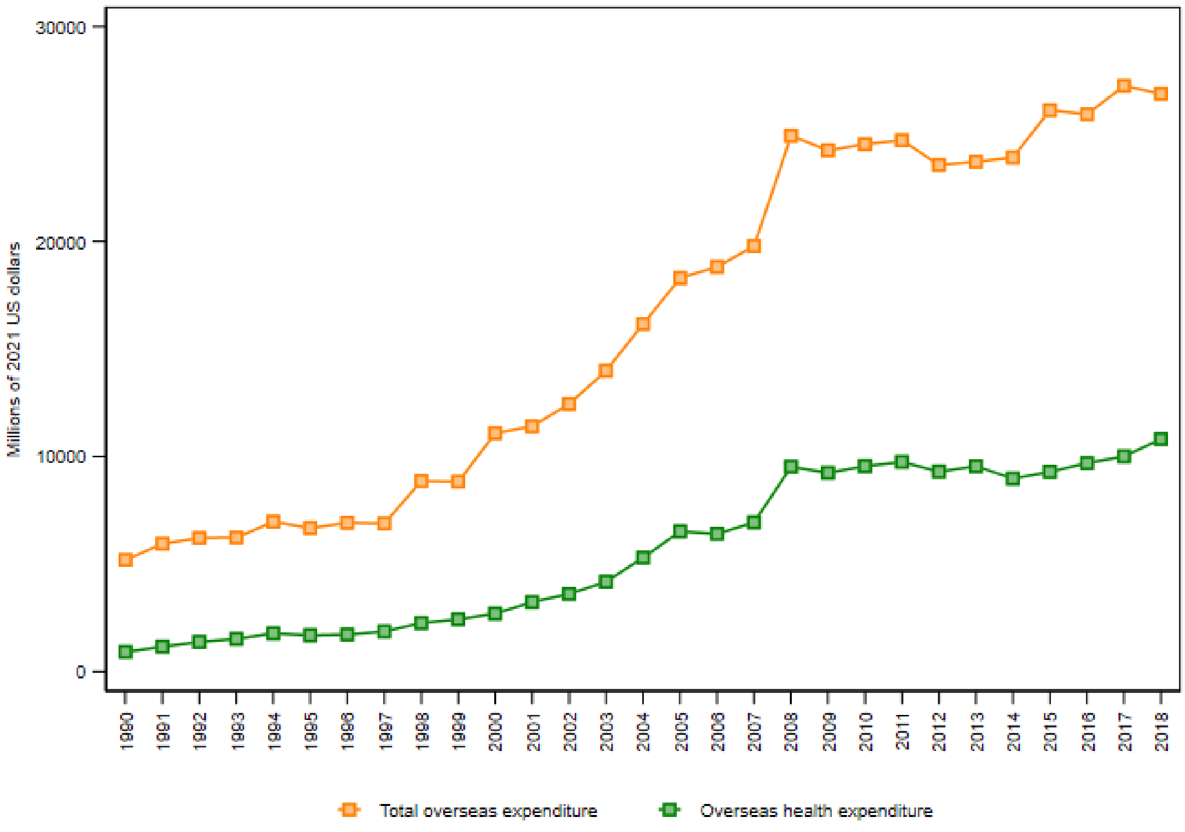

Source: IHME DAH Database (2021)

S3.8 Tracking development assistance for health from China

The estimates of DAH contributions from China is based on data from diverse sources including government reports and international development agencies databases. We generate estimates for bilateral and multilateral contributions from China separately and aggregate to obtain the total DAH contributions from China. We use project descriptions and reports where available to determine health focus areas of the contributions. For years with limited data, we use various methods inclusive of interpolation and modeling to generate estimates.

Following the addition of the China International Development Cooperation Agency (CIDCA) into our estimates last year, we now include five Chinese agencies in our bilateral DAH envelope: the National Health Commission, the Ministry of Commerce, the Ministry of Education, the Export-Import Bank of China, and CIDCA.

Detailed description of the methods for generating the estimates are reported in Micah AE, Zhao Y, Chen CS, et al[97].

S3.9 Calculating the technical assistance and program support component of development assistance for health from loan-and grant-making channels of assistance

The following methods were used to estimate the costs incurred by loan- and grant-making institutions for

administering and supporting health sector loans and grants, which includes costs related to staffing and program management.

Data on the total administrative costs were compiled for institutions in our universe for which these data were readily available: IDA, IBRD, ADB, AfDB, IDB, BMGF, the Wellcome Trust, US Foundations, CEPI, THE GLOBAL FUND, Gavi, UNICEF, UNFPA, WHO, UNITAID, PAHO, UNAIDS, USAID, and the UK Department for International Development (DFID), Japan International Cooperation Agency (JICA), Norwegian Aid Agency (NORAD) and the Swedish International Development Agency (SIDA). The sources of data for the institutions in this sample are summarized in Table S3.9. The ratio of total administrative costs to total grants and loans was calculated for each source by year. It was assumed that the percentage of operating and administrative costs devoted to health would be equal to the percentage of grants and loans that were for health. In other words, if 20% of a foundation's grants were for health, the model assumed that 20% of administrative costs of the foundation were spent on facilitating these health grants. Given this assumption, the ratios of the observed administrative costs to grants/loans were used to estimate the in-kind contribution made by each of these organizations toward maintaining their health grants and loans. For the institutions not in this sample, the ratio from the institution most similar to it was used to arrive at an estimate of in-kind contributions. For example, for US foundations we selected the top 10 and bottom 10 US foundations based on total disbursements across the entire time period. For each foundation, we calculated the ratio of the "cash basis" column of total operating and administrative expenses to grants paid. We then used the average of these top 10 and bottom 10 foundations ratio as the inkind ratio across time. Total in-kind contributions from all grant- and loan-making global health institutions are shown in Figure S3.14.

Table S3.9 Summary of data sources for calculating in-kind contributions

Organization	*Source*	*Notes*
BMGF	990 tax returns (1999-2006)<br>BMGF Trust financial statements (2007-2020)	Used "cash basis" column to calculate ratio of total operating and administrative expenses to grants paid.<br>Used "grants expenditure" statement to calculate ratio of administrative expenditure to grants/program expenditure.
US Foundations	990 tax returns	Calculated the average across time of the ratio of the "cash basis" column of total operating and administrative expenses to grants paid.
Wellcome Trust	Annual report	Calculated ratio of support costs to grants awarded by the trust
CEPI	Board of directors report, 2016-2019	Calculated ratio of operating expenses to total expenditure.
THE GLOBAL FUND	Annual report financial statements	Calculated ratio of operating expenses to grants disbursed.
Gavi	Annual report financial statements	Calculated ratio of management, general, and fundraising expenses to program expenses.
UNICEF	Annual report financial statements	Calculated ratio of programme support to total expenditure (1990-2011)<br>Calculated ratio of institutional budget to total programme and emergency budgets (2012-2020)
UNFPA	Annual financial review	Calculated ratio of institutional budget to total expenses

WHO	Financial report and annual financial statements	Calculated ratio of general operating expenses to total expenses
UNITAID	Financial statement	Calculated ratio of operating expenses to program expenses
USAID	US government budget database	Used outlays spreadsheet to calculate ratio of total outlays for USAID operating account to sum of outlays for bilateral accounts.
DFID	Annual report expense summary	Calculated ratio of DFID's administration expenses to DFID's bilateral program expenses from 2002 onward.
JICA	Statement of income in annual report	Calculated ratio of general administrative expenses to operating expenses less depreciation
SIDA and other Swedish agencies disbursing aid	Swedish OpenAid website[91]	Calculated ratio of administrative costs to the sum of all program expenses
NORAD and other Norwegian agencies disbursing aid	Norwegian Aid statistics website[92]	Calculated ratio of general administrative expenses to operating expenses less depreciation
IDA	World Bank audited financial statements	Calculated ratio of management fee charged by IBRD to development credit disbursements.
IBRD	World Bank audited financial statements	Calculated ratio of administrative expenses to loan disbursements.
AfDB	Income statements in annual reports	Calculated ratio of administrative expenses to the sum of loan and grant disbursements
ADB	Annual report	Calculated ratio of administrative expenses to the sum of loan and grant disbursements
IDB	Annual report and financial statement	Calculated the ratio of administrative expenses to total loan disbursements
EEA	Administrative budget data[93]	Calculated ratio of donor states' management budget and donor programme partners' participation to Net allocation to beneficiary countries
PAHO	Audited financial statements	Calculated ratio of general operating expenses and contractual services to total expenses.
UNAIDS	Audited financial statements	Calculated ratio of general operating expenses to total expenses.

Figure S3.15 In-kind contributions by loan- and grant-making DAH channels of assistance

This figure illustrates the proportions of financial and in-kind DAH disbursed by loan- and grant-making institutions. The proportion of in-kind DAH varies, based on the channel. The overall proportion of in-kind DAH received across all channels has grown over time.

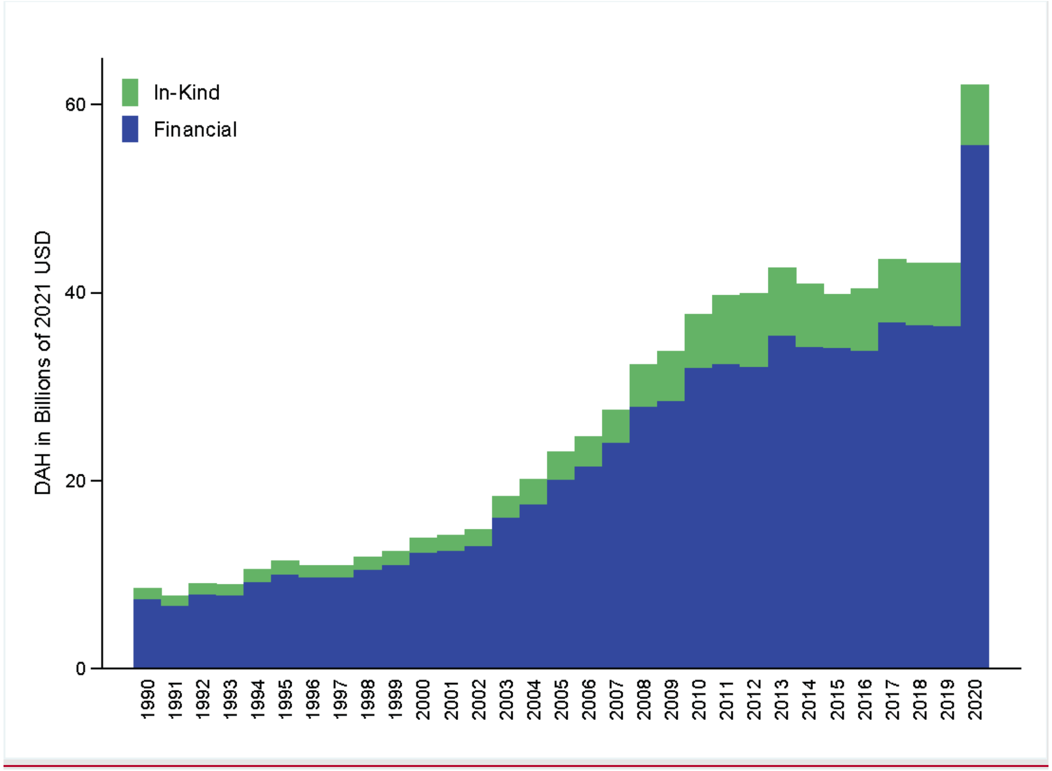

Source: IHME DAH Database (2021)

Amount and percent of development assistance for pandemic preparedness disaggregated by recipient of funds, 1990-2021

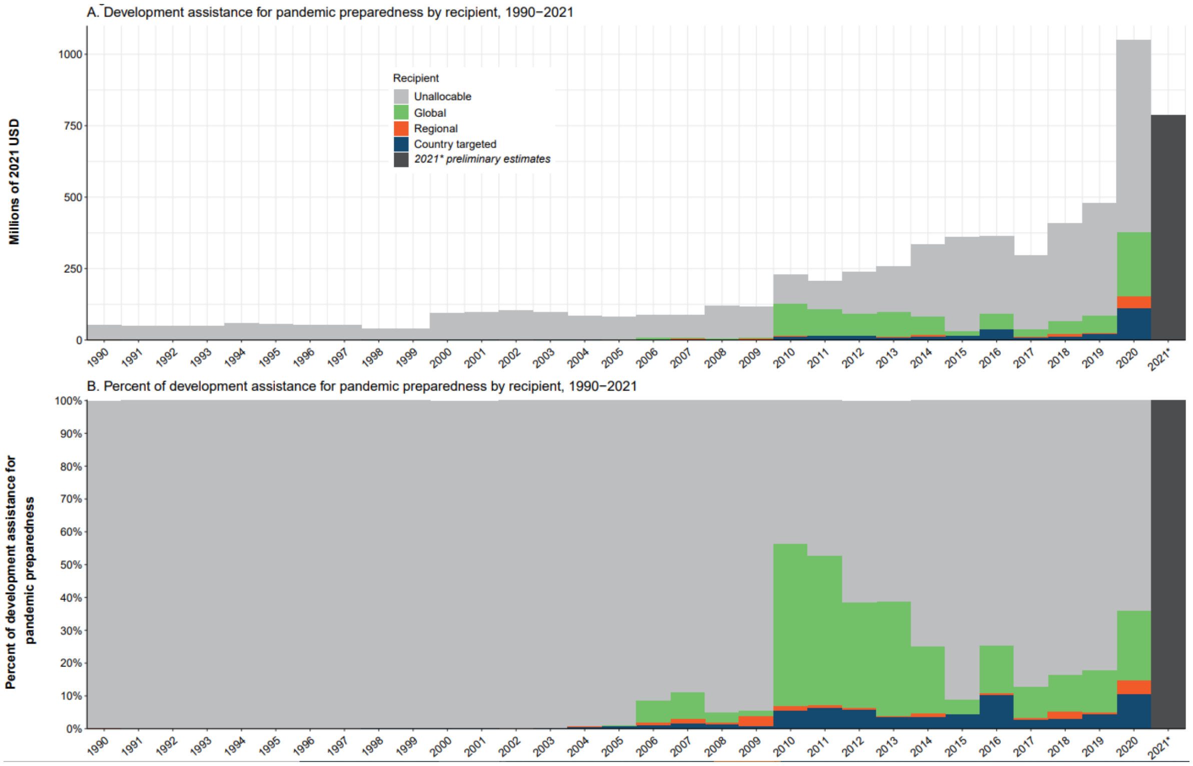

S3.11 REFERENCES

1 Dieleman JL, Schneider MT, Haakenstad A, *et al*. Development assistance for health: past trends, associations, and the future of international financial flows for health. *The Lancet* 2016; **387**: 2536–44.

2 OECD. International Development Statistics (IDS) online databases. https://www.oecd.org/development/stats/idsonline.htm (accessed October 10, 2020).

3 European Commission. Annual reports - international cooperation and development. Int. Coop. Dev. /europeaid/annual-reports_en (accessed November 2, 2020).

4 UNAIDS. PCB Archive. http://www.unaids.org/en/aboutunaids/unaidsprogrammecoordinatingboard/pcbmeetingarchive (accessed Nov 9, 2021).

5 UNICEF. UNICEF Strategic Plan: updated financial estimates, 2019-2022. 2020. https://www.unicef.org/about/execboard/files/2019-ABL5-SP_financial_estimates_2019-2022-EN-ODS.pdf

6 UNICEF. United Nations Children's Fund Financial report and audited financial statements for the year ended 31 December 2017 and Report of the Board of Auditors. United Nations: New York, 2018. https://www.unicef.org/about/execboard/files/A-73-5-Add3-BOA_report-ODS-EN.pdf

7 UNICEF. Annual Report 2020. UNICEF. https://www.unicef.org/reports/annual-report-2020 (accessed Nov, 23, 2021).

8 United Nations Population Fund. Annual Report 2020. http://www.unfpa.org/annual-report (accessed Oct 13, 2021).

9 UNITAID. Audited Financial Statements (2007-2020). Unitaid. https://unitaid.eu/publications/ (accessed Nov 22, 2021).

10 Pan American Health Organization, World Health Organization. Executive Committee Session. 2020; published online Dec 20. http://iris.paho.org/xmlui/handle/123456789/34210 (accessed Dec 10, 2021).

11 World Health Organization. Financial report and audited financial statement, 2019. World Health Organization, 2019 http://apps.who.int/gb/ebwha/pdf_files/WHA68/A68_38-en.pdf.

12 The World Bank. Projects & Operations. http://projects.worldbank.org/ (accessed Jan 13, 2021).

13 Paris M. Project database 1990-2020 obtained through personal correspondence. 2021.

14 African Development Bank. Compendium of statistics on bank group operations. Tunis, Tunisia: Statistics Department, African Development Bank.

15 African Development Bank. Financial report – Financial Management and Financial Statements Year Ended 31 December 2020. (accessed Feb 9, 2022).

15 African Development Bank. Online project database. https://www.afdb.org/en/projects-and-operations/project-portfolio/ (accessed Jan 30, 2017).

16 Inter-American Development Bank. Annual Report – The Year in Review 2020. https://www.iadb.org/en/about-us/annual-reports (accessed Oct 28, 2021).

16 Inter-American Development Bank. Projects database. https://www.iadb.org/en/projects-search(accessed Mar 9, 2022).

17 Neret M. health disbursement data. 2021; accessed Feb 7, 2022.

18 Gavi, the Vaccine Alliance. Disbursements and commitments. http://www.gavi.org/results/disbursements/ (accessed Oct 9, 2020).

19 Gavi, the Vaccine Alliance. Pneumococcal AMC. https://www.gavi.org/investing-gavi/innovative-financing/pneumococcal-amc / (accessed Oct 9, 2020).

20 Gavi, the Vaccine Alliance. Financial reports. http://www.gavi.org/funding/financial-reports/ (accessed Oct 9, 2020).

21 Gavi, the Vaccine Alliance. Cash Received database. http://www.gavi.org/funding/donor-contributions-pledges/cash-receipts/ (accessed Oct 9, 2020).

22 The Global Fund to fight AIDS, Tuberculosis and Malaria. Grants in detail and Disbursements. https://data-service.theglobalfund.org/downloads (accessed Jan 15, 2022).

23 The Global Fund to fight AIDS, Tuberculosis and Malaria. THE GLOBAL FUND annual reports. http://www.theglobalfund.org/en/archive/annualreports/ (accessed Jan 15, 2022).

24 The Global Fund to fight AIDS, Tuberculosis and Malaria. THE GLOBAL FUND pledges & contributions report. https://data-service.theglobalfund.org/downloads (accessed Jan 15, 2022).

25 GuideStar USA. Income tax filings. http://www2.guidestar.org/Home.aspx (accessed Jan 27, 2022).

26 United States Agency for International Development. VolAg report: report of voluntary agencies engaged in overseas relief and development. https://www.usaid.gov/pvo/volag-report (accessed Jan 30, 2017).

27 Thomson Reuters. Red book expanded database. New York: Thomson Reuters, 2009.

28 World Health Organization. WHO | Essential medicines. WHO. http://www.who.int/topics/essential_medicines/en/ (accessed Feb 15, 2017).

29 GuideStar. Data Sets for Research. https://learn.guidestar.org/products/data-sets-for-research (accessed November 24, 2020).

30 Bill & Melinda Gates Foundation. Audited Financial statements. Seattle, WA: Bill & Melinda Gates Foundation http://www.gatesfoundation.org/Who-We-Are/General-Information/Financials (accessed Oct 12, 2020).

31 Chan S. Foundation Awards and Payments. 2016; published online Aug 16.

32 Foundation Center. Grants database. Candid. https://candid.org/ (accessed Jan 13, 2021).

33 EEA Grants-Norway Grants, Financial Mechanism Office. EEA Grants project portal. https://eeagrants.org/resources?title=&field_resource_type_target_id=109 (accessed Sept 17, 2020).

34 Popic A. EEA project data obtained through personal correspondence. Brussels, Belgium. EEA and Norway Grants. 2020.

35 Fofana A. AfDB development support for health projects enquiry. 2019; accessed Dec 19.

36 Rouzinova R. Incorporating UNITAID into Financing Global Health Landscape. 2017; published online Aug 21.

37 Al Anood Al Abdool., United Arab Emirates Ministry of Foreign Affairs and International Cooperation. UAE Foreign Assistance in Health 1990-2008 through personal correspondence. 2018; published online Jan 24.

38 Australian Government. Department of Foreign Affairs and Trade. Aid budget and statistical information. Dep. Foreign Aff. Trade. http://dfat.gov.au/aid/aid-budgets-statistics/Pages/default.aspx (accessed Dec 11, 2020).

39 Department of Foreign Affairs and Trade. Annual reports. Dep. Foreign Aff. Trade. http://dfat.gov.au/about-us/publications/corporate/annual-reports/pages/annual-reports.aspx (accessed Nov 15, 2020).

40 Austria Federal Ministry of Finance. Federal budget. https://www.bmf.gv.at/budget/das-budget/das-budget.html (accessed Nov 24, 2020).

41 Belgium House of Representatives. Project budgets. https://www.lachambre.be/kvvcr/showpage.cfm?section=/pri/budget&language=fr&rightmenu=right_pri&story=2019-budget.xml (accessed Nov 15, 2020).

42 Government of Canada. Planning and performance. GAC. http://international.gc.ca/gac-amc/publications/plans/index.aspx?lang=eng#rpp (accessed Nov 15, 2020).

43 Danish Ministry of Foreign Affairs. Foreign affairs budget. http://www.oes-cs.dk/bevillingslove/ (accessed Nov 15, 2020).

44 European Commission. General Budget - budget on-line. https://ec.europa.eu/budget/graphs/revenue_expediture.html (accessed Nov 15, 2020).

45 Ministry of Finance Finland. State budget bills [in Finnish]. http://budjetti.vm.fi/indox/index.jsp (accessed Nov 15, 2020).

46 Ministry of Foreign Affairs and International Development. Politique francaise en faveur du developpement. Ministry of Foreign Affairs and International Development, 2019 https://www.performance-publique.budget.gouv.fr/sites/performance_publique/files/files/documents/dpt-2019/DPT2019_politique_developpement-W.pdf(accessed Nov 15, 2020).

47 Legifrance. Republique Francaise. Budget and financial documents. https://www.legifrance.gouv.fr/initRechTexte.do (accessed Nov 15, 2020).

48 German Federal Ministry of Economic Cooperation and Development. Plan of the federal budget. Fed. Minist. Econ. Coop. Dev. http://www.bmz.de/en/ministry/InDetail/budget/index.html (accessed Nov 15, 2020).

49 Greece Standing Committee on Economic Affairs. The state budget and budgets for certain special funds and services, 2013 and 2014 [in Greek]. 2014. http://www.hellenicparliament.gr/UserFiles/7b24652e-78eb-4807-9d68-e9a5d4576eff/Proyp2014-prak.pdf.

50 Greece Standing Committee on Economic Affairs. Ratification of the State Budget for the financial year 2017. http://www.hellenicparliament.gr/Nomothetiko-Ergo/Anazitisi-Nomothetikou-Ergou?law_id=76e87dd3-8cae-433f-963f-a6c50141de97 (accessed Feb 13, 2018).

51 Department of Finance, Government of Ireland. The Budget. http://www.budget.gov.ie/Budgets/2019/2019.aspx (accessed Nov 15, 2020).

52 Ministry of Foreign Affairs and International Cooperation. Stato di previsione del ministero degli affari esteri e della cooperazione internazionale. 2020. https://www.esteri.it/mae/resource/doc/2019/01/tabella_6_del_2019.pdf (accessed Nov 15, 2020).

53 Ministry of Finance Japan. Budget. http://www.mof.go.jp/english/budget/budget/index.html (accessed Nov 15, 2020).

54 Korea Official Development Assistance. Comprehensive Implementation plan for international development cooperation by year. http://www.odakorea.go.kr/hz.blltn2.YearPlanSlPL.do?brd_seq=3&blltn_div=oda (accessed Nov 15, 2020).

55 Ministry of Finance Luxembourg. State Budget[in French]. http://www.igf.etat.lu/ (accessed Nov 15, 2020).

56 New Zealand Treasury. Vote budget data - budgets of the New Zealand Government. 2019; published online May 30. http://www.treasury.govt.nz/budget (accessed Nov 15, 2020).

57 Ministry of Foreign Affairs Norway. Email correspondences. April 18, 2011, February 13, 2012, and August 14, 2013.

58 Norwegian Ministry of Finance. The National Budget 2019: a summary. https://www.statsbudsjettet.no/english/ (accessed Nov 15, 2020).

59 Ministry of Finance Portugal. State budget report. http://www.dgo.pt/Paginas/default.aspx (accessed Nov 15, 2020).

60 Ministry of Finance and Public Function Spain. State General Budget [in Spanish] http://www.congreso.es/docu/pge2019/pge_2019-tomos/PGE-ROM/doc/L_19_A_A4.PDF (accessed Nov 15, 2020).

61 Ministry of Foreign Affairs Sweden. International aid budget. http://www.regeringen.se/ (accessed Nov 15, 2020).

62 UK - Department for International Development. IATI Dashboard. http://dashboard.iatistandard.org/publisher/dfid.html (accessed Nov 15, 2020).

63 Her Majesty's Treasury United Kingdom. Budget. https://www.gov.uk/government/publications/spring-budget-2018-documents (accessed Nov 15, 2020).

64 Executive Office of the President of the United States. Budget of the United States Government. https://www.gpo.gov/fdsys/browse/collection.action?collectionCode=BUDGET&browsePath=Fiscal+Year+2014&isCollapsed=true&leafLevelBrowse=false&isDocumentResults=true&ycord=0 (accessed Nov 20, 2020).

65 US Foreign Assistance Dashboard. Foreign assistance by category, health-planned stage. https://www.foreignassistance.gov/explore (accessed Nov 20, 2020).

66 World Health Organization. Proposed programme budget 2020-2021. https://apps.who.int/iris/handle/10665/274693 (accessed October 23, 2020).

67 Joint United Nations Programme on HIV/AIDS. Financial report and audited financial statement. https://www.unaids.org/en/aboutunaids/unaidsprogrammecoordinatingboard/pcbmeetingarchive (accessed Nov 9, 2020).

68 Joint United Nations Programme on HIV/AIDS. Unified budget and workplan. https://www.unaids.org/en/aboutunaids/unaidsprogrammecoordinatingboard/pcbmeetingarchive/ (accessed Nov 9, 2020).

69 UNICEF. United Nations Children's Fund Financial report and audited financial statements for the year ended 31 December 2018 and Report of the Board of Auditors. United Nations: New York, 2019. https://digitallibrary.un.org/record/3824598?ln=en

70 Sabbah L. Health expenditure data - UNICEF. 2015; published online Sept 29.

71 UNFPA. Report on contributions by member states and others to UNFPA and revenue projections. https://executiveboard.unfpa.org/ (accessed Aug 23, 2018).

72 UNFPA. Statistical and financial review, 2020. https://executiveboard.unfpa.org/ (accessed Oct 13, 2021).

73 Smithson M, Verkuilen J. A better lemon squeezer? Maximum-likelihood regression with beta-distributed dependent variables. *Psychol Methods* 2006; **11**: 54–71.

74 Office of the European Union. EU budget 2018 Financial report. Luxembourg: Office of the European Union, 2015 https://ec.europa.eu/info/publications/eu-budget-2018-financial-report_en (accessed Nov 15, 2020).

75 President's Emergency Plan for AIDS Relief/ US Government. PEPFAR Dashboards- PEPFAR OU Budgets by Budget code FY04-FY19. 2019. https://data.pepfar.gov/dashboards (accessed October 11, 2020).

76 President's Malaria Initiative/US Government. Malaria Operational Plans (MOPs). 2019. https://www.pmi.gov/resource-library/mops (accessed December, 2020).

77Parris M. IHME-WB FGH 2021 data request. 2022.

78 Projects & Operations - Sectors. http://projects.worldbank.org/sector (accessed Jan 13, 2021).

79 Miyuki Parris. Project database 1990-2020 obtained through personal correspondence. Washington D.C.: The World Bank, 2021.

80 World Bank Group - International Development Association. IDA18 Replenishment. Int. Dev. Assoc. - World Bank. 2016; published online Feb 16. http://ida.worldbank.org/financing/replenishments/ida18-replenishment (accessed Oct 10, 2020).

81 Asian Development Bank. Online project database. https://www.adb.org/projects (accessed Feb 2, 2022).

82 African Development Bank Group. Resource Mobilization and Partnerships. https://frmb.afdb.org/?page=adf&subpage=adf-ar (accessed Dec 21, 2018).

83 Asian Development Bank. Asian Development Fund 12 Donors' Report; published online May 2016. https://www.adb.org/site/adf/replenishments (accessed Oct 12, 2018).

84 Gavi, the Vaccine Alliance. Annual financial reports. https://www.gavi.org/search?s=financial&f%5B0%5D=type%3Adocument&page=0 (accessed Oct 9, 2020).

85 World Health Organization. WHO Audited Financial Statements for the year ended 31 December 2019. Geneva, Switzerland: World Health Organization, 2019 http://apps.who.int/gb/ebwha/pdf_files/WHA72/A72_36-en.pdf (accessed October, 2020).

86 Pan American Health Organization. Financial Report of the Director and Report of the External Auditor for 2019. 1 January 2019 - 31 December 2019 https://www.paho.org/hq/index.php?option=com_content&view=article&id=15194:official-document-357&Itemid=40453&lang=en er 2018. Washington D.C.: Pan American Health Organization, 2019. (accessed October, 2020)

87 Schlutter A, Volker T, Walkenhorst P. Foundations in Europe: International Reference Book on Society, Management and Law. Gutersloh, Germany; Washington, DC: Bertelsmann Stiftung; Brookings Institution Press, 2001.

88 European Economic Area. EEA grants report. Financial Instrument 1999-2003. Final report. http://eeagrants.org/content/download/10037/139187/version/3/file/141219+FI99-03+final+report.pdf.

89 European Economic Area. EEA report - Financial Mechanism 1994-1998. Final report. http://eeagrants.org/content/download/5915/65422/version/1/file/Financial+Mechanism+1994-1998+Final+Report.pdf.

90 European Economic Area. EEA Annual Report - 2016-2017. https://eeagrants.org/content/download/13045/175890/version/1/file/Annual+Report+2016-2017.pdf.

91 Swedish International Development Agency. Sweden's aid to the world for all sectors in. Openaid.se. https://openaid.se/aid/ (accessed December, 2020).

92 NORAD. Norwegian Aid Statistics. NoradDev. https://norad.no/en/front/toolspublications/norwegian-aid-statistics/ (accessed Dec 21, 2020).

93 European Economic Area. Administrative budget - EEA Grants. https://eeagrants.org/Who-we-are/How-we-work/Administrative-budget (accessed Oct 27, 2020).

94 UNFPA. UNFPA Report on the Structured Funding Dialogue 2019-2020. https://www.unfpa.org/pcm/node/19087 (accessed Oct 28, 2020).

95 World Health Organization. Status of collection of assessed contributions, including Member States in arrears in the payment of their contributions to an extent that would justify invoking Article 7 of the Constitution. Geneva, Switzerland: World Health Organization, 2020 http://apps.who.int/gb/ebwha/pdf_files/WHA72/A72_37-en.pdf (accessed October, 2020).

96 World Health Organization. Voluntary contributions by fund and by contributor, 2019. Geneva, Switzerland: World Health Organization, 2020. http://apps.who.int/gb/ebwha/pdf_files/WHA72/A72_INF5-en.pdf (accessed Oct 26, 2020).

97 Micah AE, Zhao Y, Chen CS, et al. Tracking development assistance for health from China, 2007–2017BMJ Global Health 2019;4:e001513.

98 Lavado R.F. ADB development support for health projects enquiry. 2019; accessed Dec 19.

99 International Monetary Fund. World Economic Outlook Database: October 2019. https://www.imf.org/external/pubs/ft/weo/2019/02/weodata/index.aspx (accessed Oct 20, 2019).

100 Organisation for Economic Co-operation and Development. Review of the ODA coefficients of select health-related organisations on Annex 2 and the Single Table of the Converged Statistical Reporting Directives for the Creditor Reporting System (CRS) and the annual DAC Questionnaire. chrome-extension://efaidnbmnnnibpcajpcglclefindmkaj/viewer.html?pdfurl=https%3A%2F%2Fwww.oecd.org%2Fofficialdocuments%2Fpublicdisplaydocumentpdf%2F%3Fcote%3DDCD%2FDAC%2FSTAT(2021)34%26docLanguage%3DEn&clen=596929&pdffilename=DCD-DAC-STAT(2021)34.pdf (accessed Jan 20, 2022)

S3.11 Tracking Development Assistance for Health: incorporating COVID-19

COVID Keyword Search

To further our COVID DAH analysis, we developed a COVID-specific keyword search. This keyword search takes the detailed information supplied by grants' project descriptions and categorizes COVID spending into either vaccine related COVID spending or spending not related to vaccines. The keywords used to flag a project as vaccine related were " VACCIN", " IMMUNIZ", " IMMUNIS", and " DOSE". The program areas not related to vaccines are : 1) Coordination, planning, financing and monitoring, 2) Risk communication, community engagement and infodemic management, 3) Surveillance, epidemiological investigation, contact tracing and adjustment of public health and social measures , 4)Laboratories and diagnostics, 5) Infection prevention and control, 6) Case management, clinical operations and therapeutics, 7) Operational support and logistics, and supply chains, 8) Maintaining other essential health services and systems, 9) Research & Development for diagnostics and therapeutics from development agencies, 10) Points of entry, international travel and transport and mass gatherings and 11) Other.

The keyword search first separates vaccine projects which are then processed using the vaccine specific keywords. All projects not tagged as vaccine are processed using the non-vaccine keyword list and program areas. Each program area is defined by a set of keywords (in English, Spanish, Portuguese, French, Italian, Dutch, German, Norwegian, and Swedish) which identify words and phrases in each grants' project description and proportion the grant total amount into each respective bucket. Grants which do not tag any keywords are automatically placed into the Other program area. Grant project descriptions are cleaned of special characters, made uppercase, void of excessive mid-line spacing, and padded with beginning and ending spaces, before passing through our keyword search. Table S3.11.1. presents the keywords in English for the ten defined program areas.

Table S3.11.1. List of keywords used to define program areas

Program Area	Keywords
Coordination, planning, financing and monitoring	" COORDINAT", " PLAN", " MONITOR", " PREPARED", " EMERGENCY RESPONSE", " NATIONAL PLAN", " EMERGENCY OPERATIONS CENT", " EOC ", " RISK ASSESS", " EVALUAT", " COMMUNICAT "
Risk communication, community engagement and infodemic management	" COMMUNICAT", " CELL CENT", " COMMUNIT", " VULNERABL", " AWARE", " BEHAVIOR", " BEHAVIOUR", " MARGINALIZED ", " RISK"
Surveillance, epidemiological investigation, contact tracing and adjustment of public health and social measures	" SURVEIL", " RAPID RESPONSE TEAM", " CASE INVESTIGATION ", " CONTACT TRAC", " DETECT", " CLOSE CONTACT", "PHONE"
Laboratories and diagnostic	" LAB", " TEST", " SEROLOGICAL ", " PCR ", " ANTIBODY ", " DIAGNOS"

Infection prevention and control	" INFECTION PREVENT", " IPC ", " PERSONAL PROTECTIVE EQUIP", " PPE ", " MASK", " HAND SANITIZ", " HYGIENE ", " WASH ", " SANITAT", " ISOLATION ", " NOSOCOMIAL ", " DISINFECT", " SOAP ", " FACE COVER", " SHIELD ", " PREVENT"
Case management, clinical operations and the therapeutics	" CASE MANAG", " INTENSIVE CARE ", " CLINIC", " DIAGNOS"," TREAT", " TRIAGE ", " REFERRAL ", " THERAP", " VENTILAT", " OXYGEN ", " GUIDELINE", " REMDESIVIR ", " DEXAMETHASONE ", " CONVALESCENT PLASMA ", " TOCILIZUMAB ", " ACTEMRA "
Supply chain and logistics	" OPERATIONAL SUPPORT ", " LOGISTIC", " STOCK ", " SUPPL", " PROCURE", " EQUIP", " DEPLOY", " DELIVERY SYSTEM"
Maintaining other essential health services and systems	" ESSENTIAL HEALTH SERVICE", " HEALTH WORKFORCE PLAN", " BASIC HEALTH SERVICE", " BASIC SERVICE", " ROUTINE HEALTH SERVICE", " ROUTINE SERVICE", " HIV ", " AIDS ", " TUBERCULOSIS ", " IMMUNIZATION ", " MALARIA ", " MATERNAL ", " CHILD HEALTH ", " NEWBORN ", " REPRODUCTIVE HEALTH ", " SRH ", " NON COMMUNICABLE DISEASE", " NCD ", " NONCOMMUNICABLE DISEASE", " ESSENTIAL HEALTHCARE SERVICE", " TB "
Research & Development for diagnostics and therapeutics from development agencies	" DIAGNOSTICS MANUFACTUR", " THERAPEUTICS MANUFACTUR", " DIAGNOSTICS DEVELOP", " THERAPEUTICS DEVELOP", " MODEL", " INNOVAT", " RESEARCH", " R D ", " R AND D "
Points of entry, international travel and transport and mass gatherings	" BORDER", " AIRPORT", " TRAVEL ", " POINT OF ENTRY ", " FLIGHT"
Other	Anything not categorized by the above keywords

Table S3.11.2. List of keywords used to define vaccination program areas

Program Area	Keywords
Research & development for vaccines	" RESEARCH", " R D ", " R AND D "
Commodity and procurement	" PROCURE", " COMMODITY", " PURCHASE", " DOSE"
Demand creation and social mobilization	" COMMUNICAT", " COMMUNIT", " AWARE", " BEHAVIOR", " BEHAVIOUR", " BCC ", " IEC ", " CONFIDENCE", " SENSITIZATION ", " VULNERABL", " INFORMATION CAMPAIGN", " VACCINATION

	CAMPAIGN", " BUILDING TRUST ", " COVID 19 VACCINE UPTAKE ", " COVID VACCINE UPTAKE ", " PUBLIC PERCEPTION"
Supply chain	" SUPPLY CHAIN", " COLD CHAIN "
Hygiene and PPE	" INFECTION PREVENT", " IPC ", " PERSONAL PROTECTIVE EQUIP", " PPE ", " MASK", " HAND SANITIZ", " HYGIENE ", " WASH ", " SANITAT", " NOSOCOMIAL ", " DISINFECT", "SOAP BAR", " FACE COVER", " SHIELD "
Waste management	" WASTE ", " SAFETY BOX", " DISPOS", " VIAL"
Supply chain and logistics	" OPERATIONAL SUPPORT ", " LOGISTIC", " STOCK ", " SUPPL", " PROCURE", " EQUIP", " DEPLOY", " DELIVERY SYSTEM"
Human resources	" MEDICAL SCHOOL", " CENTERS OF EXCELLENCE ", " TRAINING ", "CAPACIT", " SKILLED WORKER", " HEALTH WORKER", " HEALTHCARE WORKER ", " HEALTH CARE WORKER", " HCW", " HEALTHCARE PROFESSIONAL", " HEALTH CARE PROFESSIONAL", " HCP", " SKILLED STAFF ", " HEALTH PROFESSIONAL", " HUMAN RESOURCE", " HUMAN CAPITAL ", " MEDICAL WORKER", " WORKFORCE ", " MEDICAL EDUCATION ", " HEALTH EDUCATION ", " CONTINUING EDUCATION ", "NURSE", "DOCTOR", "PHYSICIAN", "MIDWIFE", "MIDWIVES", "MEDICAL LABORATORY SCIENTIST", "SURGEON", "SPECIALIST", "PHARMACIST", "HEALTH LABOR", "LABOR MARKET", "PERSONNEL", "MEDICAL PRACTITIONER", "DENTAL PRACTITIONER", "TASK SHIFTING", "TASK SHARING", " HRH "
Technical assistance	" TECHNICAL ASSISTANCE ", " TECHNICAL SUPPORT ", " CONSULTANT ", " TA "
Planning and coordination	" COORDINAT", " PLAN", " MONITOR", " PREPARED", " EMERGENCY RESPONSE", " NATIONAL PLAN"
Delivery	" DELIVERY", " DISTRIBUT", " OUTREACH", " PER DIEM", " TRANSPORT"
Safety	" SAFETY", " PHARMACOVIGILANCE ", " SIDE EFFECT", " ADVERSE EVENT", " AEFI", " AESI", " VIGILANCE"
Monitoring	" M E ", " M AND E ", " MONITORING ", " EVALUATION ", " SURVEILLANCE ", " DATA SYSTEM"
Vaccine Other	Anything not categorized by the above vaccine keywords

Notes:

1. Proportions of vaccine projects tagged as "Hygiene and PPE" were included in the "Infection prevention and control" category.

Example Project Going Through COVID Keyword Search
Example project description: "COVID 19 Response: Distributing PPE and PCR Tests to Frontline Workers"<br><br>Example project amount: $900<br><br>Passing through our COVID keyword search, this project would be tagged by National labs and testing twice (for " PCR " and " TEST") and once by Infection prevention and personal protective equipment (for " PPE "). The resulting program area keyword counts would then proportion the grant amount 2/3rd into National labs and testing and 1/3rd into Infection prevention and personal protective equipment, attributing $600 to the former and $300 to the latter.

Table S3.11.2. Summary of COVID data sources by channel

Channel	IATI	UNOCHA	Correspondence	channel Website	Other website
AfDB			X	X	
AsDB			X	X	
Bilateral, Australia	X				
Bilateral, Austria				X	
Bilateral, Belgium	X				

Bilateral, Canada	X				
Bilateral, Switzerland	X	X			
Bilateral, Germany	X				
Bilateral, Denmark	X				
Bilateral, Spain	X				
Bilateral, Finland	X				
Bilateral, France	X				
Bilateral, UK	X				
Bilateral, USA	X				
Bilateral, UAE		X			
Bilateral, South Korea	X				
Bilateral, Italy	X	X			
Bilateral, Netherlands	X				
Bilateral, Norway	X				
Bilateral, New Zealand	X	X			
Bilateral, Sweden	X				
Bilateral, China		X			X
NGOs		X			
US foundations					X
UNICEF		X	X		
UNFPA		X			
UNAIDs			X		
UNITAID			X		
PAHO				X	
WHO				X	
IDB			X	X	
Gavi			X	X	X
The Global Fund				X	
Gates Foundation				X	

World Bank			X	X	
CEPI			X		
EC	X				

Bilateral agencies and European Commission

To track bilateral agencies and the European Commission we used IATI as the main source and supplemented information for bilaterals that do not report to IATI by using UNOCHA, correspondence for Japan, and the data retrieved from the Austrian Development Agency website[1-3]. The bilaterals not reporting COVID-19 projects to IATI include Austria, , Japan, and the UAE.

For IATI, we accessed data through the d-portal by first selecting their COVID-19 filter followed by selecting sector groups "Basic health (122)", "Health, general (121)", "Population policies / programmes and reproductive health (130)", and "Emergency response (720)". IATI's COVID-19 filter includes projects where "COVID-19" is included in the activity title, activity description, or transaction description or where COVID-19-specific codes are included in humanitarian scope (globally recognised codes) or tag ("COVID-19" code) data fields[4]. To keep only bilateral government and EC projects we kept only the reporting organizations listed in the table above. Not all of the reporting organizations were present in the resulting downloaded dataset. From the d-portal we downloaded the csv files of "Ended projects" and the csv of "Projects ending soon". These csv files contained project title, reporting organization, total commitment, total disbursement, and a link to the more detailed project page on the d-portal. To get recipient country, date information, and project descriptions we used a web-scraper and the unique project links to download the information.

We also downloaded all Covid projects from United States foreign assistance website for 2020 and 2021. The projects were compared to those in AITI for the bilateral partner. After checking for duplicates, only 66 projects were added to the final dataset.

Table S3.11.3. Bilateral agency and EC data sources and commitment disbursement ratio breakdown.

Country	Data Source	Reporting organizations	Disbursement commitment median ratio
Australia	IATI	Australia – Department of Foreign Affairs and Trade	1
Austria	ADA website		N/A (no missing disbursement data)
Belgium	IATI	Belgian development agency; Belgian Development Cooperation	1
Canada	IATI	Canada – Global Affairs Canada; Canada – International Development Research Centre; Canada – Department of Finance	1
Denmark	IATI	Denmark Ministry of Foreign Affairs, Danida	1
Finland	IATI	Finland Ministry of Foreign Affairs	1
France	IATI	Agence Francaise de Developpement; France – Ministry for Europe and Foreign Affairs	0.964
Germany	IATI	Germany – Federal Foreign Office; Germany – Ministry for Economic Cooperation and Development	1
Greece	No data		N/A
Ireland	IATI	Ireland – Department of Foreign Affairs and Trade	N/A
Italy	IATI, UNOCHA	AICS – Agenzia Italiana per la Cooperazione allo Sviluppo / Italian Agency for Cooperation and Development	1
Japan	correspondence	Japan International Cooperation Agency; Ministry of Foreign Affairs of Japan	1
Luxembourg	No data		N/A
Netherlands	IATI	Netherlands – Ministry of Foreign Affairs	1
New Zealand	IATI, UNOCHA	New Zealand – Ministry of Foreign Affairs and Trade	1
Norway	IATI	Norad – Norwegian Agency for Development Cooperation	1
Portugal	No data		N/A

South Korea	IATI	Republic of Korea	1
Spain	IATI	Spain – Ministry of Foreign Affairs and Cooperation; Spanish Agency for International Development Cooperation (AECID)	1
Sweden	IATI	Sweden, through Swedish International Development Cooperation	0.949
Switzerland	IATI, UNOCHA	Switzerland – Swiss Agency for Development and Cooperation	1
United Arab Emirates	UNOCHA	Ministry of Foreign Affairs – UAE	1
United Kingdom	IATI	UK – Foreign, Commonwealth and Development Office; UK – Department for Business, Energy and Industrial Strategy (BEIS); UK – Department for Environment, Food and Rural Affairs	1
United States	IATI/USAID	United States Agency for International Development (USAID)	0.848
European Commission	IATI	European Commission – Directorate-General for International Cooperation and Development; European Commission – Humanitarian Aid and Civil Protection; European Commission – Neighborhood and enlargement negotiations; European Commission – Service for foreign policy instruments	1

We processed IATI data by removing non-DAH projects, filling in missing disbursement information, disaggregating multi-year projects, and tagging repurposed projects. To remove non-health projects we searched project titles and descriptions for non-health and health keywords. We dropped any projects that contained a non-health keyword (education, food assistance, economic relief/recovery, cash transfer, etc.) and no health keywords. To account for projects missing disbursement information, we calculated disbursement to commitment ratios and used the median for each bilateral donor to calculate project disbursement. The median was chosen since several donors had outlier ratios that resulted in means above 1. Multiple year projects were divided by using a start and end date to calculate the total number of days in a project and determine the disbursement per day. We then multiplied this by the days in each year. The data does not contain a variable specifying repurposed or new money. We assumed that all projects starting prior to 2020 were repurposed in 2020.

To supplement this data we downloaded all columns and rows available in the UNOCHA dataset and isolated health sector-specific projects where the donor agency was one of the 24 bilateral governments

we track. To avoid double counting we dropped UNOCHA projects if the bilateral agency and recipient country were the same as any IATI projects since we believe IATI to be a more comprehensive data source. The data contains separate rows for commitments, disbursements, and pledges from the same project. We match these projects with multiple rows to avoid double counting. We used a 1:1 commitment to disbursement ratio to fill in missing disbursement information. Projects that went to recipient agencies classified in the data as NGOs or UN agencies were tagged as double counting and therefore removed from final dataset.

We received correspondence data for Japan bilateral projects from Shuhei Nomura, Associate Profession at the Graduate School of Medicine, The University of Tokyo, Japan, which included project title, links to project descriptions, date, recipient country, aid type, and commitment. We copied over project descriptions from the Japan MOFA website. Several loan descriptions included social and economic relief in addition to COVID-19 relief. We used a 1:1 disbursement to commitment ratio to determine disbursement. Lastly, we downloaded project level data from the Austrian Development Agency website, which included project title, description, recipient country, CRS sector, date, and disbursement information. We searched these projects for COVID-19 keywords to pull out projects for COVID-19. These supplementary projects for Japan and Austria bilateral were all classified as new money based on the date and descriptions of the projects.

We combined this data and ran our keyword search on project descriptions and titles. In order to avoid double-counting spending we have reallocated any money identified as repurposed from our traditional CRS data at the most granular level possible by donor country. After the reallocation we added our COVID data to the top of our reduced traditional envelope in order to obtain the full envelope.

UNAIDS

For UNAIDS, we received data from our contact Jose Antonio, at the agency, on repurposed spending for COVID-19 for recipient countries for 2020. These data were used to inform COVID-19 related spending for the agency, and the repurposed money was removed from the total DAH envelope we estimated for 2020 to avoid double-counting. No keyword search was used on this data; all money was manually assigned to ‘Maintaining other essential health services and systems’, since UNAIDS COVID spending was on maintaining existing programming in the face of the COVID-19 pandemic.

We did not have source information for this channel, and so categorized the source as “Unallocable”.

We did not receive 2021 data for UNAIDS and did not find 2021 disbursements through web scrapping either. Therefore we assumed there was no COVID related disbursements through UNAIDS.

UNICEF

For the UNICEF channel, we identified UNOCHA as the most comprehensive location for UNICEF grant-specific data related to Covid-19[2]. We downloaded all columns and rows available in the database where UNICEF is the destination organization (Figure S3.10.1). To process these data, we subset on the Sector column to isolate “Health”. From there, we checked for grants reporting multiple commitments or disbursements. If a grant had multiple rows reporting commitments and disbursements, we merged the rows and preferred disbursements over commitments. We then cleaned up the donor and recipient

country names to merge before passing the cleaned and formatted data through our COVID keyword search.

In UNOCHA's data there is a column reporting whether the grant is funded through new or repurposed money – repurposed money being "left-over" amounts intended for another use that has been re-directed to support the pandemic. In our existing data we assume that we have already tracked all repurposed money, so in order to avoid double-counting we reduced the envelope of our traditional UNICEF data by reallocating the health focus area assignments at the most granular level possible – in this case by donor country. After this adjustment, we are able to directly add the UNICEF COVID total amount onto our existing estimates where the repurposed-tagged money now exactly fills the reduction we made by donor country.

We also received correspondence data on vaccine delivery from UNICEF. This data was cleaned and incorporated into our model following the process detailed above.

Figure S3.10.1: Sample of UNOCHA's UNICEF Health Data[2]

3	Boundary	Source org.	Destination org.	Sector	Amount (US$)
85	Incoming	Asian Development Bank	United Nations Children's Fund	Health	$10,000,000
89	Incoming	Canada, Government of	United Nations Children's Fund	Health	$73,684
90	Incoming	Canada, Government of	United Nations Children's Fund	Health	$74,072
101	Incoming	World Bank	United Nations Children's Fund	Health	$3,869,071
102	Incoming	World Bank	United Nations Children's Fund	Health	$1,500,090
104	Incoming	World Bank	United Nations Children's Fund	Health	$195,300
105	Incoming	World Bank	United Nations Children's Fund	Health	$4,715,074
106	Incoming	World Bank	United Nations Children's Fund	Health	$884,258
109	Incoming	Ireland, Government of	United Nations Children's Fund	Health	$396,902
161	Incoming	United States of America, Government of	United Nations Children's Fund	Health	$389,194
250	Incoming	United States of America, Government of	United Nations Children's Fund	Health	$243,409
270	Incoming	Saudi Arabia (Kingdom of), Government of	United Nations Children's Fund	Health	$4,000,000
288	Incoming	World Bank	United Nations Children's Fund	Health	$384,894
315	Incoming	Yemen Humanitarian Fund	United Nations Children's Fund	Health	$15,999,547
317	Incoming	Japan, Government of	United Nations Children's Fund	Health	$1,084,166
320	Incoming	Ethiopia Humanitarian Fund	United Nations Children's Fund	Health	$25,920
329	Incoming	Canada, Government of	United Nations Children's Fund	Health	$102,799
398	Incoming	Ukraine Humanitarian Fund	United Nations Children's Fund	Health	$139,997
420	Incoming	United States of America, Government of	United Nations Children's Fund	Health	$1,000,000
542	Incoming	Japan, Government of	United Nations Children's Fund	Health	$2,712,528

UNFPA

For the channel UNFPA, we also used the UNOCHA Financial Tracking Service database to access data on the 'COVID-19 Global Humanitarian Response Plan' for grant-specific data related to COVID-19[2]. We downloaded all the grants available in the database, selecting only those in the health sector where UNFPA is the destination organization. Both commitment and disbursement data were available – where there was grant overlap between the two, disbursement data only has been included, to avoid double-counting any grants. Where only commitment data was available, a 1:1 commitment to disbursement ratio was used to estimate disbursement spending. Source information was also available in the dataset.

Following the same strategy as for UNICEF, the value of the repurposed COVID-19 has been removed from our estimates for general UNFPA spending for 2020 and 2021.

We then used the keyword search described above to categorize each of the grants into the 11 COVID-19 subcategories.

UNITAID

For UNITAID, we received data from correspondence with Nargiza, Mazhidova, a Manager, Impact and Value for Money. This dataset included UNITAID grants, which had a variable tagging those that were COVID related. We followed the same data processing as our regular Unitaid channel pipeline. We split COVID funding into three program areas based on disease area assignments in the data and information from the Unitaid website regarding their contributions to the Access to COVID-19 Tools Accelerator: "Maintaining essential health services and systems", "Case management and treatment", and "National labs and testing"[5]. Grants were counted as new money.

PAHO

We downloaded total contributions made to PAHO since the beginning of the Covid-19 pandemic from the PAHO COVID-19 Response Fund[6]. We extracted outbreak and crisis response expenditures from the 2020 financial report for the same year. We downloaded the Pan American Health Organization response to Covid-19 in the Americas for both 2020 and 2021 to extract priority areas of the pandemic response strategy[7]. We used 2020 outbreak and crisis response expenditures as 2020 disbursement and subtracted it from total contributions made to the PAHO Covid-19 fund to estimate the amount that remained for 2021. We estimated the program areas portions by running PAHO priority areas and descriptions through our keyword search. We then multiply the proportions by the amount disbursed in each year to estimate the amount that was targeted to each of the program areas.

WHO

For WHO, we downloaded the underlying data for the Contributions to WHO for COVID-19 visualizations, which reports detailed information on the donors and partners of WHO's COVID-19 response (Figure S3.11.2.)[8]. This data contained donor information, distributed organization, pandemic flag (COVID-ACT vaccine specific or COVID General) and assigned Strategic Preparedness and Response Plans (SPRPs) that we used for assigning program areas. We pulled recipient country information from the distributed organization variable, which combines the code for the WHO regional office as well as the ISO code of the recipient country. We dropped contributions where the source country was the same as the recipient country as we considered those domestic disbursements. We assigned funds going to headquarters and specific offices that did not indicate a recipient country as "Global" projects. To avoid double counting WHO disbursements, we subtracted disbursements where the source was another one of the channels we track in the aggregation step of our COVID pipeline. For example, the African Development Bank provided money to WHO, which we later took out of our African development bank channel. We used "Pandemic flag" variable to separate projects that were COVID vaccine focused with the label "COVID-ACT" from those in "COVID-General". We used "distributed" variable as our commitment value and "award budget" as our disbursement value. We matched commitments and disbursements by donor, contributor, award ID, award type, and distributed organization since the data contained individual rows for commitments and disbursements. Once we cleaned the data, we ran our COVID keyword search on the SPRP variable. We manually adjusted several keyword assignments. The SPRP "Pandemic supply chain coordination" was changed from being split between "Supply chain and logistics" and "Country-level coordination" to "Supply chain and logistics". "Technical expertise and guidance" was manually assigned to go to "Country-level coordination". "Epidemiological analysis and forecasting" was manually assigned to "Surveillance, rapid-response

teams, and case investigation". "Points of entry" was manually assigned to "Country-level coordination". All WHO 2021 COVID disbursements was treated as new money.

S3.11.2: Sample of WHO's underlying COVID contributions data

Parent Donor: All | Contributor: All | Award: All | SPRP: All

Underlying Data for COVID Awards and Implementation

Parent Donor	Contributor	Award	Pandemic Flag	Form of Award	Award Classification	Distributed Office	Distributed Organization	SPRP Code	SPRP
Afghanistan	Afghanistan	70360	COVID	In Cash	Standard				N/A
Afghanistan	Afghanistan	70360	COVID	In Cash	Standard				
Afghanistan	Afghanistan	70360	COVID	In Cash	Standard	HQ	HQ/OCC		N/A
Afghanistan	Afghanistan	70360	COVID	In Cash	Standard	HQ	HQ/OCC	A1	A1 - Partner coordi
Afghanistan	Afghanistan	70360	COVID	In Cash	Standard	HQ	HQ/OCC	A1	A1 - Partner coordi
Afghanistan	Afghanistan	70360	COVID	In Cash	Standard	EM	EM_AFG		N/A
Afghanistan	Afghanistan	70360	COVID	In Cash	Standard	EM	EM_AFG		N/A
Afghanistan	Afghanistan	70360	COVID	In Cash	Standard	EM	EM_AFG	B5	B5 - National labor
Afghanistan	Afghanistan	70360	COVID	In Cash	Standard	EM	EM_AFG	B5	B5 - National labor
Afghanistan	Afghanistan	70360	COVID	In Cash	Standard	EM	EM_AFG	B5	B5 - National labor
Afghanistan	Afghanistan	70360	COVID	In Cash	Standard	EM	EM_AFG	B5	B5 - National labor
Afghanistan	Afghanistan	70360	COVID	In Cash	Standard	EM	EM_AFG	B5	B5 - National labor
Afghanistan	Afghanistan	70360	COVID	In Cash	Standard	EM	EM_AFG	B8	B8 - Operational su
Afghanistan	Afghanistan	70360	COVID	In Cash	Standard	EM	EM_AFG	B8	B8 - Operational su
Afghanistan	Afghanistan	70360	COVID	In Cash	Standard	EM	EM_AFG	B8	B8 - Operational su
Afghanistan	Afghanistan	70360	COVID	In Cash	Standard	EM	EM_AFG	B8	B8 - Operational su
Afghanistan	Afghanistan	70360	COVID	In Cash	Standard	EM	EM_AFG	B8	B8 - Operational su
Afghanistan	Afghanistan	70697	COVID	In Cash	Standard				N/A
Afghanistan	Afghanistan	70697	COVID	In Cash	Standard				
Afghanistan	Afghanistan	70697	COVID	In Cash	Standard	HQ	HQ/BOS		N/A
Afghanistan	Afghanistan	70697	COVID	In Cash	Standard	EM	EM/BOS		N/A
Afghanistan	Afghanistan	70697	COVID	In Cash	Standard	EM	EM/BOS		N/A
Afghanistan	Afghanistan	70697	COVID	In Cash	Standard	EM	EM/BOS	A7	A7 - Pandemic sup
Total									

Contributors | Financial Flow (SPRP) | SPRP Pillars | Country SPRP Funding | Pooled Funds Report | Raw Data

World Bank

We received health-related disbursement data from correspondence with Miyuki Parris at the World Bank. This dataset included funding from both the IDA and IBRD branches for COVID-19 specific projects. We followed the same initial data processing as the general World Bank channel workflow. This included disaggregating projects with multiple recipients equally, dropping high-income countries, and disaggregating funding from projects with commitments from IBRD and IDA proportionately. COVID-19 related projects were isolated for separate data processing.

We applied the keyword search described previously to each project's respective individual component descriptions obtained from the World Bank Project Database where available[9]. We extracted the component descriptions from Project Information Documents (PAD) or Project Paper documents from the World Bank Project Database (Figure S3.11.3). We allocated funding to these components according to the proportion of the project's funding committed to each component. After disaggregating money by program area for each component, we aggregated the components together for each project. This process ensured that the disaggregation by program area best reflected the funding priorities in each project. Where individual component descriptions were not available, we ran the keyword search on the project development objective. We obtained information regarding commitments, disbursements, grant and loan disaggregation for IDA projects from Project Appraisal Documents as well (Figure X2.). We assigned all IBRD funding as loans. We used this webpage to disaggregate between new and repurposed money[10]. We assigned projects listed as, "benefiting from the dedicated COVID-19 Fast-Track Facility" as new money. We assigned projects listed as "benefiting from other forms of finance/redeploying of existing projects" as repurposed money.

To assign donor countries for IDA, we used a weighted average of historic contributions (2016 to 2018) to IDA from countries and from IBRD. This is unnecessary for IBRD as the branch receives its income entirely from debt repayments. Through information from correspondence, we were unable to disaggregate funding as new or repurposed money.

We further tracked projects from the World Bank Support for Country Access to COVID-19 Vaccines [24] to allocated appropriate updates to funding of existing projects or track projects primarily focused on COVID-19 Vaccine efforts. Similar to general COVID projects, we extracted component information from Project Information Documents (PID) and Project Papers to allocated the Vaccine funding to their respective areas.

Figure S3.11.3

38. **The project will comprise the following components**:

39. **Component 1. Case detection and management [US$8.5 million]:** Activities supported by this component include: establishing and upgrading laboratory, isolation and treatment centers and equipping them with medical supplies and furniture and network installation[8]. NIPH will be upgraded; diagnostic capacity of the four provincial laboratories as well as laboratories attached to the 21 provincial referral hospitals will be built[9]; and isolation and treatment centers in all 25 municipal/provincial referral hospitals will be upgraded.

40. **Component 2. Medical Supplies and Equipment [US$6.5 million]**: This component will finance the procurement of medical supplies and equipment needed for activities outlined in the COVID-19 Master Plan, including business continuity of essential services, such as (i) case management; and (ii) infection prevention and control. Specifically, items procured will include drugs and medical supplies for case management and infection prevention. This component will also allow for flexibility to allocate resources for the purchasing of essential pharmaceutical (medicines and vaccines) and medical supplies as the availability in the country becomes reduced due to the economic impact of the pandemic and the existing mechanisms are insufficient to address the critical health system needs.

Figure S3.11.4.

IDA Resources (in US$, Millions)

	Credit Amount	Grant Amount	Guarantee Amount	Total Amount
**Cambodia**	20.00	0.00	0.00	20.00
National PBA	20.00	0.00	0.00	20.00
**Total**	**20.00**	**0.00**	**0.00**	**20.00**

Expected Disbursements (in US$, Millions)

WB Fiscal Year	2020	2021	2022	2023
Annual	3.00	14.00	2.70	0.30
Cumulative	3.00	17.00	19.70	20.00

AfDB

For the African Development Bank (AfDB), we received data from contacts, Abayomi Ayodabo, at AfDB, on all loans and grants–distributed in 2021. Any loans or grants which reference COVID-19 in their title or description were selected. All of these loans and grants were classified by AfDb as "Multi-sector" and had both commitment and disbursement information. Reviewing the project description for each project (available on the website)[11,12], we attributed between 0% and 66% of each project's spending to health spending. Based on detailed project descriptions on their COVID-related projects-website, we classified each project as a loan or grant, and extracted descriptions of the health programs to use in the keyword search. [11,12]
We then used the keyword search described above to categorize each of the grants into COVID-19 program areas.
For the source information, we used replenishment data from the general DAH pipeline to inform proportions, and then applied those proportions to the reported COVID-19 health spending to estimate country-specific donations.

ADB

To estimate health disbursement from the Asian Development Bank (ADB), data were webscraped from the online project database. Any loans or grants which reference COVID-19 in their title or description are removed from the general ADB spending pipeline, and a separate dataset is created using only COVID-19 loans and grants. For these grants and loans, we evaluated the proportion of each which was designated for health sector spending in the "Report and Recommendation of the President" for each project on the ADB website[13], and scaled the total loan or grant quantity down by this proportion. This is so that we only include health spending in our COVID-19 estimates. Both commitment and disbursement data are available for this dataset. Both commitment and disbursement data are available for all projects in this dataset. For the purposes of estimating COVID-19 spending, we relied on the disbursement information.

We then use health program descriptions in the "Report and Recommendation of the President" for each grant and loan to run the keyword search described above. This categorizes each of the grants/loans into the 19 COVID-19 program areas.

For the source information, we used data from the general DAH pipeline to inform proportions, and then applied those proportions to the reported COVID-19 health spending to estimate country-specific donations.

IDB

For the Inter-American Development Bank (IDB), we received data from our contact Matilde Neret, Contract and Budget Officer at Inter-American Development Bank, on all health-related loans and grants disbursed. Any loans or grants which reference COVID-19 in their title or description are removed from the general IDB spending pipeline, and a separate dataset is created using only COVID-19 loans and grants.

We then used the keyword search described above to categorize each of the grants into the nine COVID-19 subcategories.

GAVI

For the GAVI channel, we used a three-pronged approach to estimating COVID DAH contributions for 2020. First, we utilized data we received through correspondence with Alain McLaren, a Senior Analyst at GAVI, on prepayments and firm order commitments (FOCs) to estimate COVID Health System Strengthening and COVAX non-country specific totals. This dataset includes the two aforementioned programs, a note about their global nature, and the amount of money going towards each. Second, we manually extracted country-specific repurposed money totals from GAVI Situation Reports published monthly on their website[14]. Third, we manually extracted country-specific COVAX commitments from a regularly published document on GAVI's website titled "COVAX AMC Donors Table"[15]. For each of these sources we used distinct methods based on available information on each.

For the prepayments and FOCs data the dataset attests that the reported funds are new money and that the recipients of these prepayments have yet to be determined – which we classify as global. For the GAVI Situation Reports, the Situation Report documents note that the "amount approved for support" column which we extract is entirely repurposed money so we treated it as such and reallocated as necessary (details below). For the COVAX Advanced Market Commitment table, we tagged all reported spending as new money according to COVAX not reporting otherwise. Finally, we appended these three datasets and passed the data through our COVID keyword search.

For GAVI 2021 estimates, we relied on 2021 disbursement data received through correspondence from Alain McLaren and additional disbursement data on vaccine delivery provided in curated databases. All disbursement data was available at the recipient level. To get income data we downloaded an update of the COVAX AMC Donors table described above. This data reported cumulative commitments from 2021-2025. We used this data to create proportions for each donor

For 2021 disbursement data received from correspondence we canceled out negative disbursements by recipient. We combined the disbursement data and multiplied this data by the income proportions created. We then ran the COVID keyword search on the description and/or program type variables that were available depending on the source. Based on information from correspondence and the GAVI website, we assigned all 2021 data as new money and grants. Finally, we appended the 2020 and 2021 data to have a complete dataset. Similar to the UNICEF channel and others, in order avoid double-counting spending we have reallocated any money identified as repurposed from our traditional GAVI data at the most granular level possible – in this case by donor country. After the reallocation, we added our COVID data to the top of our reduced traditional envelope in order to obtain the full envelope.

The Global Fund

For the Global Fund, we directly pulled country-specific disbursements and repurposed resources information from the "Funding Approved for COVID-19 Response" report published on the GFATM website[16]. This dataset contains tables on country-specific new money disbursements awarded as well as country-specific repurposed money utilized.

Little formatting was needed for this source other than splitting regional and multi-country observations, where money was divided evenly between all countries represented in each region – country lists in each region were found using the Global Fund's data explorer[17]. For example, the Multicountry Americas (Andean) region was evenly split between Colombia, Ecuador, Peru, and Venezuela.

Like other channels, we made adjustments to our traditional Global Fund data to account for the repurposed money identified in our data source. The reallocation occurred at the recipient country level, and was again filled by the tagged repurposed money after appending our Global Fund COVID dataset.

CEPI

For the CEPI channel, we received COVID-19 data through correspondence with Tuva Stolen and Mads Hogholen, the Head of Controlling and Director of Finance and Operations at CEPI, respectively. The dataset we received includes donor-specific COVID disbursement amounts which we confirmed with our contacts are all new money observations. After thoroughly reviewing CEPI's reported activities in 2020-2021 we deemed all COVID-19 spending as going to our R&D COVID program area because CEPI as an organization is focused on R&D, and lead on the COVAX facility on development of the vaccine. We then run our COVID-19 keyword search.

NGOs

For non-governmental organizations, we compiled data from UNOCHA's Financial Tracking System (FTS), which reports continuously updated data on humanitarian funding flows[2]. They receive report contributions from government donors, UN agencies, NGOs and other humanitarian actors[2]. The data includes project level information, which included source organization, source type, recipient country, and whether the money was new or repurposed and year. We kept records where the listed recipient agency type included "NGO" and sectors that included "health", "multi-sector", "gender", "covid", "unspecified", or "child protection" in the sector title after reviewing projects from these sectors and determining they were DAH. If there were multiple sectors in a project, commitment and disbursements amounts were split equally among all sectors. The data contains separate rows for commitments, disbursements, and pledges from the same project. We match these projects with multiple rows to avoid double counting. We used a 1:1 commitment to disbursement ratio to fill in missing disbursement information.

We then used the keyword search described above to categorize NGO projects into COVID program areas.

In order to avoid double-counting spending we have reallocated any money identified as repurposed from our traditional data at the most granular level possible by NGO agency. After the reallocation, we added our COVID data to the top of our reduced traditional envelope in order to obtain the full envelope.

Gates Foundation

For the Bill and Melinda Gates Foundation, we pulled the full grants data found on their website. After subsetting to just 2020 and 2021 projects, cleaning project description, title, and location strings, we applied our COVID keyword search on all the project title, description, and categories. Upon reviewing the documentation of their grant database, we identified all extracted projects as new money according to how they sort year-specific results in the tool.

China

For the China Bilateral channel, we used a three-tiered approach to estimating COVID contributions in 2020 and 2021. First, we pull data from UNOCHA where the Source is the Chinese Government[2]. UNOCHA tags new money and repurposed commitment and disbursement observations, and grants with multiple observations of commitments or disbursements have been matched in an identical fashion to the UNCIEF channel. Second, we identified our regular channel National Health Commission estimates as being entirely repurposed for COVID relief; this assumption based on the "White paper on China's action combating COVID-19 which said 56 medical teams that are abroad have assisted in covid-19 response, including providing technical assistance and health education, and delivered over 400 training sessions[19]. recommendation coming from an external FGH collaborator, Yingxi Zhao, who has worked with us previously on our estimation of China's bilateral contributions[20]. Finally, we calculated a back of the envelope estimate of the Chinese government's global PPE, medical equipment and additional medical team contributions using official press releases and the Duke University's Launch and Scale Speedometer, which we consider to be new money.

To format the UNOCHA data, we began by subsetting on the Sector column to isolate "Health". From there, we reformatted column data types to make manipulations simpler, and checked for grants reporting multiple commitments or disbursements. If a grant had multiple rows reporting commitments and disbursements, we merged the rows and preferred disbursements over commitments as to not double-count spending. We then run our COVID keyword search.

To process the NHC data, we extracted the value of DAH contribution from our regular channel data and append it to our COVID data, and then passed it through our COVID keyword search. All NHC contributions were assumed to be medical workforce support hence the reallocation as COVID-related contributions.

To calculate our back of the envelope estimate of the Chinese government's global PPE, medical equipment, vaccine donation and medical team contributions, we began by reviewing press releases from various Chinese ministries to gauge the number of batches of PPE, vaccine and the number of medical teams sent globally. For 2020, we estimated that 34 additional medical teams were dispatched and 200 out of 283 batches of PPE were send to LMICs in 2020 [21, 22]. We then estimated a round unit cost per PPE/medical team batch, assuming that each batch contained roughly the same equipment, volume, and staffing. Finally, we multiplied the estimated number of batches by the estimated unit cost and report $60,000,000 as the total PPE, medical equipment and medical team contributions. For 2021, we estimated that additional 3 medical teams were dispatched. As for vaccine donation, we estimated that 80 millions of vaccines have been delivered as of Oct 26, 2021[25]. This number was similar to Duke university's Launch and Scale Speedometer data which suggested that China's vaccine donation for LMICs in 2021 was 71 million doses[28]. Vaccine's cost was based on official press release of China's donation to Laos where 2.5 million does of vaccines were estimated worth of 80 million RMB – roughly 32 RMB per dose/5 USD per dose[26]. This is a lower estimate as compared with the official market price from UNICEF COVID-19 Vaccine Market Dashboard which reported the market price for China's vaccine around 20 USD per dose[27]. We multiplied the estimated number of doses with the unit cost estimate from Laos and report this as the cost of the Chinese government's vaccine donation contributions and labeled this as Vaccine commodity. As for recipient information of vaccine donation, we incorporated data from Duke university's Launch and Scale Speedometer data and allocated the remaining 9 million doses as "unallocable".

To account for repurposed money, we performed a similar reallocation of traditional channel money as noted in other channels – here by Chinese agency. We completely reallocated the 2020 and 2021 NHC traditional channel amount to COVID, and otherwise reallocated UNOCHA's reported repurposed money also by donor agency.

US Foundations

We utilized purchased dataset from CANDID[23] and isolated USA originating grants funding for COVID-19 related purposes for 2020 and 2021. We removed grants without health-related codes from the dataset. If a grant had an intended beneficiary, we labeled this as the recipient country or countries. If there was no intended beneficiary, we labeled the direct recipient country of the grant as the recipient country. If the grant identified more than one country as the recipient, we divided the funding equally between the recipients. We excluded high-income recipient countries.

We flagged recipient agencies that we track through other channels to avoid double counting this funding. We flagged descriptions that specify any repurposing of funding as repurposed money. We applied the keyword search to descriptions included with each individual grant to categorize projects into Covid-19 program areas.

Table S3.11.4. Top recipients of DAH for COVID-19

Recipient	World Bank Income Group	GBD super region	Development assistance received for COVID-19 (millions USD)	Precent of DAH for COVID-19
Philippines	LM	Southeast Asia East Asia and Oceania	2568·6	94·0
India	LM	South Asia	2511·9	73·1
Bangladesh	LM	South Asia	2229·3	87·2
Indonesia	LM	Southeast Asia East Asia and Oceania	1656·8	79·3
Argentina	UM	Latin America and Caribbean	1325·4	75·8
Ethiopia	L	Sub-Saharan Africa	1182·9	68·9
Nigeria	LM	Sub-Saharan Africa	1117·2	56·0
Pakistan	LM	South Asia	1017·6	57·3

Sri Lanka	LM	Southeast Asia East Asia and Oceania	789·4	93·0
Democratic Republic of the Congo	L	Sub-Saharan Africa	708·7	49·0
South Africa	UM	Sub-Saharan Africa	628·3	63·5
Peru	UM	Latin America and Caribbean	546·1	96·3
Myanmar	LM	Southeast Asia East Asia and Oceania	544·9	69·4
Kenya	LM	Sub-Saharan Africa	535·1	61·4
Mongolia	LM	Central Europe Eastern Europe and Central Asia	499·9	88·2
Mozambique	L	Sub-Saharan Africa	498·2	49·9
Ecuador	UM	Latin America and Caribbean	494·4	96·5
Uganda	L	Sub-Saharan Africa	494·4	47·8
Papua New Guinea	LM	Southeast Asia East Asia and Oceania	484·1	84·5
Ghana	LM	Sub-Saharan Africa	473·9	67·3
Nepal	LM	South Asia	419·0	89·9
Cambodia	LM	Southeast Asia East Asia and Oceania	400·5	82·9
Cote d'Ivoire	LM	Sub-Saharan Africa	375·6	66·6
Mauritius	UM	Southeast Asia East Asia and Oceania	354·2	99·4

Ukraine	LM	Central Europe Eastern Europe and Central Asia	329·2	62·3
Zimbabwe	LM	Sub-Saharan Africa	302·7	40·9
Malawi	L	Sub-Saharan Africa	298·3	41·9
Tanzania	LM	Sub-Saharan Africa	289·7	31·6
Afghanistan	L	North Africa and Middle East	281·3	69·0
Senegal	LM	Sub-Saharan Africa	276·9	72·9
Cameroon	LM	Sub-Saharan Africa	251·7	57·2
Iraq	UM	North Africa and Middle East	242·6	96·4
Tunisia	LM	North Africa and Middle East	240·4	98·0
Colombia	UM	Latin America and Caribbean	238·0	57·0
Mali	L	Sub-Saharan Africa	229·7	68·9
Jordan	UM	North Africa and Middle East	221·4	81·9
Madagascar	L	Sub-Saharan Africa	219·0	75·3
Zambia	LM	Sub-Saharan Africa	217·0	38·4
Burkina Faso	L	Sub-Saharan Africa	200·8	46·7
Niger	L	Sub-Saharan Africa	193·8	66·1
Angola	LM	Sub-Saharan Africa	190·9	60·2

Yemen	L	North Africa and Middle East	188·8	80·9
Honduras	LM	Latin America and Caribbean	185·9	87·8
Sudan	L	North Africa and Middle East	185·5	40·8
Togo	L	Sub-Saharan Africa	182·2	65·9
Rwanda	L	Sub-Saharan Africa	181·8	45·2
Somalia	L	Sub-Saharan Africa	181·7	83·3
Central African Republic	L	Sub-Saharan Africa	171·1	69·2
South Sudan	L	Sub-Saharan Africa	169·2	72·9
Chad	L	Sub-Saharan Africa	168·4	60·1
Guinea	L	Sub-Saharan Africa	160·4	75·1
Syria	L	North Africa and Middle East	159·6	91·8
Georgia	UM	Central Europe Eastern Europe and Central Asia	150·0	92·4
Kazakhstan	UM	Central Europe Eastern Europe and Central Asia	132·1	79·7
Benin	LM	Sub-Saharan Africa	125·1	57·4
Burundi	L	Sub-Saharan Africa	124·0	52·7
Vietnam	LM	Southeast Asia East Asia and Oceania	123·4	41·8
Iran	LM	North Africa and Middle East	121·0	87·7

Turkey	UM	North Africa and Middle East	120·2	88·5
Lebanon	UM	North Africa and Middle East	110·1	86·1
Palestine	LM	North Africa and Middle East	109·7	99·8
Belarus	UM	Central Europe Eastern Europe and Central Asia	106·2	82·4
Fiji	UM	Southeast Asia East Asia and Oceania	104·9	100·0
Haiti	LM	Latin America and Caribbean	102·7	83·1
Tajikistan	LM	Central Europe Eastern Europe and Central Asia	101·7	67·0
Mauritania	LM	Sub-Saharan Africa	101·1	80·8
Sierra Leone	L	Sub-Saharan Africa	98·4	56·0
Moldova	UM	Central Europe Eastern Europe and Central Asia	94·5	87·9
Serbia	UM	Central Europe Eastern Europe and Central Asia	91·1	92·3
Paraguay	UM	Latin America and Caribbean	86·4	90·0
El Salvador	LM	Latin America and Caribbean	83·8	66·2
Nicaragua	LM	Latin America and Caribbean	81·5	41·5
Egypt	LM	North Africa and Middle East	80·9	55·0
Liberia	L	Sub-Saharan Africa	74·8	53·2

Lesotho	LM	Sub-Saharan Africa	73·7	62·0
Kyrgyzstan	LM	Central Europe Eastern Europe and Central Asia	70·0	67·6
Bosnia and Herzegovina	UM	Central Europe Eastern Europe and Central Asia	68·4	100·0
Maldives	UM	Southeast Asia East Asia and Oceania	65·8	100·0
North Macedonia	UM	Central Europe Eastern Europe and Central Asia	64·1	100·0
Thailand	UM	Southeast Asia East Asia and Oceania	61·4	52·1
Uzbekistan	LM	Central Europe Eastern Europe and Central Asia	61·2	37·8
Algeria	LM	North Africa and Middle East	57·1	96·1
Congo	LM	Sub-Saharan Africa	56·4	54·2
Libya	UM	North Africa and Middle East	55·5	99·8
The Gambia	L	Sub-Saharan Africa	55·4	60·6
China	UM	Southeast Asia East Asia and Oceania	54·4	23·7
Laos	LM	Southeast Asia East Asia and Oceania	53·1	55·4
Dominican Republic	UM	Latin America and Caribbean	51·9	53·4

Solomon Islands	LM	Southeast Asia East Asia and Oceania	49·0	92·4
Swaziland	LM	Sub-Saharan Africa	43·1	55·0
Djibouti	LM	Sub-Saharan Africa	43·0	76·1
Morocco	LM	North Africa and Middle East	41·9	58·1
Guinea-Bissau	L	Sub-Saharan Africa	40·1	49·2
Guatemala	UM	Latin America and Caribbean	38·7	59·0
Cuba	UM	Latin America and Caribbean	36·7	68·8
Timor-Leste	LM	Southeast Asia East Asia and Oceania	36·5	75·2
Cape Verde	LM	Sub-Saharan Africa	36·1	88·6
Gabon	UM	Sub-Saharan Africa	29·7	82·4
Namibia	UM	Sub-Saharan Africa	29·6	54·5
Bolivia	LM	Latin America and Caribbean	28·0	23·4
Albania	UM	Central Europe Eastern Europe and Central Asia	26·5	83·2
Panama	UM	Latin America and Caribbean	26·0	28·7
Malaysia	UM	Southeast Asia East Asia and Oceania	25·6	90·7
Turkmenistan	UM	Central Europe Eastern Europe and Central Asia	23·8	78·4

Brazil	UM	Latin America and Caribbean	23·4	9·4
Bhutan	LM	South Asia	22·8	67·9
Jamaica	UM	Latin America and Caribbean	22·6	64·4
Armenia	UM	Central Europe Eastern Europe and Central Asia	22·3	65·2
Sao Tome and Principe	LM	Sub-Saharan Africa	21·1	75·0
Azerbaijan	UM	Central Europe Eastern Europe and Central Asia	20·8	61·4
Comoros	LM	Sub-Saharan Africa	17·9	71·6
Eritrea	L	Sub-Saharan Africa	17·5	31·8
Botswana	UM	Sub-Saharan Africa	17·0	45·5
Mexico	UM	Latin America and Caribbean	15·0	99·5
Vanuatu	LM	Southeast Asia East Asia and Oceania	13·4	77·5
Samoa	LM	Southeast Asia East Asia and Oceania	11·2	85·0
Montenegro	UM	Central Europe Eastern Europe and Central Asia	10·7	88·1
Guyana	UM	Latin America and Caribbean	10·0	68·6
Costa Rica	UM	Latin America and Caribbean	9·4	8·0
Tonga	UM	Southeast Asia East Asia and Oceania	6·4	45·9

Federated States of Micronesia	LM	Southeast Asia East Asia and Oceania	6·0	100·0
Marshall Islands	UM	Southeast Asia East Asia and Oceania	5·3	100·0
Dominica	UM	Latin America and Caribbean	5·0	97·6
Kiribati	LM	Southeast Asia East Asia and Oceania	5·0	85·9
Russia	UM	Central Europe Eastern Europe and Central Asia	4·5	75·1
Romania	UM	Central Europe Eastern Europe and Central Asia	4·3	8·4
Suriname	UM	Latin America and Caribbean	4·0	47·7
Tuvalu	UM	Southeast Asia East Asia and Oceania	4·0	85·5
Grenada	UM	Latin America and Caribbean	3·5	96·5
Saint Lucia	UM	Latin America and Caribbean	2·8	95·6
Saint Vincent and the Grenadines	UM	Latin America and Caribbean	2·8	95·6
Equatorial Guinea	UM	Sub-Saharan Africa	2·5	94·0
Belize	LM	Latin America and Caribbean	2·2	53·4
North Korea	L	Southeast Asia East Asia and Oceania	1·7	99·1
Niue	H	Southeast Asia East Asia and Oceania	1·3	100·0

Tokelau	UM	Southeast Asia East Asia and Oceania	0·9	100·0
Bulgaria	UM	Central Europe Eastern Europe and Central Asia	0·1	100·0
Global		Global	0·0	0·0
Saint Helena		Latin America and Caribbean	0·0	0·0
		Unallocable	10029·3	36·0
INKIND	INKIND	INKIND	-1·1	-0·0
Global		Global	0·0	0·0

References:

0. development-portal.org. http://d-portal.org/ctrack.html#view=search (accessed Jan 14, 2022).
1. Financial Tracking Service. https://fts.unocha.org/ (accessed Feb 2, 2022).
2. Austrian Development Agency. https://www.entwicklung.at/en/projects/all-projects (accessed Mar 01, 2022).
3. Version 1 Guidance Accessing and Using COVID-19 Data. Google Docs. https://docs.google.com/document/u/1/d/1hV_Bk2_VEQmrbSPbp3vE0Rho-gZtOJBSpLkOYInTnOY/edit?usp=embed_facebook (accessed Mar 18, 2022).
4. UNITAID. Unitaid and COVID-19 - Unitaid. https://unitaid.org/covid-19/#en (accessed Feb 2, 2022).
5. PAHO/WHO. COVID-19 Situation Reports. https://www.paho.org/en/covid-19-situation-reports (accessed Nov 10, 2021).
6. PAHO response to COVID-19 situation reportshttps://www.paho.org/en/topics/coronavirus-infections/coronavirus-disease-covid-19-pandemic (accessed Nov 17, 2021).
7. WHO. Contributions to WHO for COVID-19. https://app.powerbi.com/view?r=eyJrIjoiNzNmNTRkMWEtNmZjMS00NzdjLWEyMDYtYWExYzA4NzVhZGQwIiwidCI6ImY2MTBjMGI3LWJkMjQtNGIzOS04MTBiLTNkYzI4MGFmYjU5MCIsImMiOjh9 (accessed Jan 22, 2021).
8. The World Bank. Projects & Operations. World Bank. https://projects.worldbank.org/en/projects-operations/projects-home (accessed Jan 22, 2021).
9. The World Bank. World Bank Group’s Operational Response to COVID-19 (coronavirus) – Projects List. World Bank. https://www.worldbank.org/en/about/what-we-do/brief/world-bank-group-operational-response-covid-19-coronavirus-projects-list (accessed Jan 22, 2021).

10. African Development Bank. AfDB Data Portal. https://projectsportal.afdb.org/dataportal/ (accessed Feb 09, 2022).
11. African Development Bank. Our COVID-19 response to date. African Development Bank - Building today, a better Africa tomorrow. 2020; published online June 24. https://www.afdb.org/en/news-events/our-covid-19-response-date (accessed Jan 22, 2021).
12. Asian Development Bank. Projects and Tenders. Asian Development Bank. https://www.adb.org/projects (accessed Jan 22, 2021).
13. Gavi, The Vaccine Alliance. COVID-19 situation reports. https://www.gavi.org/news-resources/document-library/covid-19-situation-reports (accessed Jan 22, 2021).
14. Gavi, The Vaccine Alliance. COVAX Facility. https://www.gavi.org/covax-facility (accessed Jan 22, 2021).
15. The Global Fund. COVID-19 Operational Response. https://www.theglobalfund.org/en/covid-19/ (accessed Jan 31, 2022).
16. The Global Fund. Map - The Global Fund Data Explorer. https://data.theglobalfund.org/investments/home (accessed Jan 22, 2021).
17. Bill & Melinda Gates Foundation. Grantmaking - Awarded Grants. https://www.gatesfoundation.org/How-We-Work/Quick-Links/Grants-Database (accessed Jan 22, 2021).
18. The State Council, The People's Republic of China. China's action against the new coronavirus epidemic. http://www.gov.cn/zhengce/2020-06/07/content_5517737.htm (accessed Jan 22, 2021).
19. Micah AE, Zhao Y, Chen CS, et al. Tracking development assistance for health from China, 2007–2017BMJ Global Health 2019;4:e001513.
20. Xinhuanet. Xinhua International Current Review: The World Reverberation of the Great Anti-epidemic Spirit. http://www.xinhuanet.com/2020-09/09/c_1126473118.htm (accessed Jan 22, 2021).
21. People's Daily. Fully demonstrate China's conscious responsibility as a large responsible country (by everyone). http://paper.people.com.cn/rmrb/html/2020-09/24/nw.D110000renmrb_20200924_2-09.htm (accessed Jan 22, 2021).
22. Candid. Candid. https://candid.org/ (accessed Jan 10, 2022).
23. The World Bank. World Bank Support for Country Access to COVID-19 Vaccines – Projects List. World Bank. https://www.worldbank.org/en/who-we-are/news/coronavirus-covid19/world-bank-support-for-country-access-to-covid-19-vaccines (accessed Feb 3, 2022).Science and technology daily. 2021. Understanding China's global response to the pandemic. http://www.stdaily.com/index/kejixinwen/2021-10/26/content_1228365.shtml
24. Science and technology daily. 2021. Understanding China's global response to the pandemic. http://www.stdaily.com/index/kejixinwen/2021-10/26/content_1228365.shtml
25. People's Government of Yunnan Province. China's donation of COVID vaccine and other materials to Laos. http://www.yn.gov.cn/ztgg/yqfk/fkyw/202111/t20211126_230887.html
26. UNICEF. Covid-19 vaccine market dashboard. https://www.unicef.org/supply/covid-19-vaccine-market-dashboard
27. Duke University. Duke Global Health Innovation Center. https://launchandscalefaster.org/covid-19/vaccinedonations

Forecasting Health Spending from 2020 through 2050

Supplementary Appendix

Global Burden of Disease Financing Global Health Collaborator Network

Updated: 8 November, 2022

Contents

S4.1 Data **2**
S4.1.1 Summary of Data Sources . . . 2
S4.1.2 World Health Organization's Global Health Expenditure Database . . . 2
S4.1.3 WB WDI, IMF WEO, PWT, and Maddison . . . 4
S4.1.4 Institute for Health Metrics and Evaluation's Development Assistance for Health Database . . . 4
S4.1.5 Institute for Health Metrics and Evaluation's Global Burden of Disease Study 2019 . . . 4

S4.2 Covariates **6**
S4.2.1 List of Forecasted Variables . . . 6
S4.2.2 Covariates Used for Forecasting . . . 6

S4.3 Ensemble Modeling **8**
S4.3.1 Sub-model Setup . . . 8
S4.3.2 Covariates . . . 9
S4.3.3 Convergence to Global Growth Rates . . . 9
S4.3.4 Specifications . . . 10

S4.4 Inclusion and Exclusion Criteria **12**

S4.5 Creating the Forecasts **13**
S4.5.1 Ranking Sub-Models . . . 13
S4.5.2 Uncertainty Estimation . . . 13

S4.6 Ad-hoc Draws Correlation **15**
S4.6.1 Motivation . . . 15
S4.6.2 Bivariate Correlated Distributions . . . 15

S4.7 Future Health Scenarios **15**
S4.7.1 Long-term Growth Regressions . . . 16
S4.7.2 Forecasting Greater and Lesser Scenarios . . . 16
S4.7.3 Uncertainty Estimation . . . 17

S4.8 Package and Architecture **18**
S4.8.1 Architecture . . . 18
S4.8.2 Template Model Builder . . . 18

References **19**

S4.9 Tables and Figures **21**

Introduction

The objective of this study is to provide data on future health spending patterns that can guide decision-makers. These analyses produced comprehensive and comparable set of gross domestic product and all-sector government spending estimates, followed by all the compo-nents of a country's total health expenditure. Additionally, using observed past trends, we predicted the possible trajectories of the covariates of interest conditional on following an optimistic and pessimistic pattern based on global rates of change.

The purpose of this appendix is to describe in detail the methodology used in our analyses. Subsequent sections contain information on all data sources, ensemble forecasting strategies, inclusion criteria, and uncertainty estimation used to generate our estimates, as well as our guidelines on how our future health scenarios analyses were conducted.

While our manuscript's focus includes only five years of forecasts through 2026 to focus more clearly on COVID-19, pandemic preparedness and the resource requirements recommended by the G20 High-Level Independent Panel (HLIP), as suggested by reviewers, we have included our forecasts through 2050 in our appendix.

SECTION 4. FORECASTING HEALTH SPENDING FROM 2020 through 2050

S4.1 Data

S4.1.1 Summary of Data Sources

We used data from seven sources for the analyses:

- World Health Organization's (WHO) Global Health Expenditure Database
- International Monetary Fund's Database of Country Fiscal Measures in Response to the COVID-19 Pandemic published in October of 2020 and 2021
- World Bank (WB) World Development Indicators (WDI) July 2022 database
- International Monetary Fund's (IMF) World Economic Outlook (WEO) April 2022 database
- United Nations Statistics Division National Accounts Main Aggregates Database 2021 (UN)
- Penn World Tables 10.0 (PWT)
- Angus Maddison Project Database 2020 (Maddison)
- Institute for Health Metrics and Evaluation's Development Assistance for Health Database 2021 (IHME)
- Institute for Health Metrics and Evaluation's Global Burden of Disease Study 2019 (IHME GBD 2019)

Specifically, we collected health expenditure information on all available sources that is comparable across countries and complete for most countries from WHO and IHME, and demographic data from the WPP, while the underlying data for producing gross domestic product (GDP) and general government expenditure (GGE) were extracted from the IMF, WB, UN, PWT, and Maddison. Table S4.1 presents the definitions for the various health expenditure sources.

S4.1.2 World Health Organization's Global Health Expenditure Database

We used Global Health Expenditure Database (GHED) data from the WHO to generate our estimates, which spans 192 countries between 2000 and 2019 (in the December 2021 version). From the GHED, we pulled the following variables:

(i) Compulsory prepayment (Other, and unspecified, than FS.3)

(ii) Other revenues from NPISH n.e.c.

(iii) Other revenues from corporations n.e.c.

(iv) Other revenues from households n.e.c.

(v) Social insurance contributions

(vi) Transfers from government domestic revenue (allocated to health purposes)

(vii) Gross Domestic Product

(viii) Voluntary prepayment

To ensure we were using the best possible data, we downloaded the metadata for each data point for all of the indicators from the GHED website. We used the metadata to decide how each given data point should be weighted, from 1 to 5, being applied as inverse variance weights. We established guidelines for the metadata that informed how the underlying data points should be weighted, giving priority to factors such as complete, documented source information and penalizing factors such as having been derived or estimated. We adjusted these data by converting them from current local currency to 2021 US dollars. Details of the weighting guidelines and classification are explained in the supplementary appendix our retrospective analysis paper.

Once we have an incomplete set of data points for the health expenditure variables we are interested in forecasting, we used Spatiotemporal Gaussian process regression (ST-GPR) to model the full time-series for each variable across a total of 204 countries. ST-GPR is a stochastic modeling technique that is designed to detect signals amidst noisy data. Unlike classical linear models that assume that the trend underlying data follows a definitive functional form, GPR assumes that the specific trend of interest follows a Gaussian Process, existing with some pointwise mean and covariance function [1]. The covariates that were used in order to determine the initial fit of our health expenditure variables are: lag-distributed income, all-sector government expenditure per capita, and proportion of total population

over the age of 64; using a Matérn covariance function for the distribution of the Gaussian process. Once we determined an initial prediction of our dependent variables, using the variability of data across regions, Gaussian process regressions (GPR) were run in order to estimate 500 draws of each country-year estimate per metric.

We also supplemented government health spending data using the IMF's Database of Country Fiscal Measures in Response to the COVID-19 Pandemic, in order to capture additional government spending in response to the pandemic.

S4.1.3 WB WDI, IMF WEO, UN, PWT, and Maddison

The WDI Database provides data on a wide range of development related variables, including data on GDP and GDP per capita. Data series in this database begin in 1960 and span through the present. The IMF's WEO Database provides data on various macroeconomic indicators. Macroeconomic series data are available from 1980 to present. The PWT is a database that provides real national accounts data for 183 countries and territories from 1950 to present. The Maddison Project database provides historical GDP, GDP per capita, and population data dating as far back as Roman times. The UN Statistics Division also provides data on GDP for 220 countries from 1970 to present. We utilized GDP per capita as a primary covariate to produce forecasts. GDP per capita from 1950 through 2021 was constructed using the method described in *James et al* [2]. The method utilized extracted data from a number of sources (IMF, WB, UN, PWT and Maddison), and used multiple random effects models to estimate a mean GDP per capita series to be used in our analysis. Similarly, we used the same methodology to produce a mean general government expenditure (GGE) per GDP series, from 1980 through 2021.

S4.1.4 Institute for Health Metrics and Evaluation's Development Assistance for Health Database

Development assistance for health estimates were obtained from the Institute for Health Metrics and Evaluation's Development Assistance for Health Database. To generate these estimates, IHME collected audited budgets, annual reports, and project records from the primary development agencies providing assistance for the health sector. These records are augmented by information acquired via correspondence, and are standardized and compiled to provide a comprehensive perspective on international financial flows for health. These estimates are tracked backward to the source of the funds and forward to the country recipient, and are available from 1990 through 2021 and 2020, respectively.

S4.1.5 Institute for Health Metrics and Evaluation's Global Burden of Disease Study 2019

Covariates used in forecasting health spending, such as populations and educational attainment per capita [3, 4], were obtained from the Institute for Health Metrics and Evaluation's Global Burden of Disease Study 2019. Population forecasts used were from IHME's Future Health Scenarios work, which uses a cohort component method of projection (CCMP [5]), and takes forecasts of age-specific fertility rates, mortality, migration, and the sex ratio at birth as inputs [6]. Age-specific fertility forecasts were projected by modeling completed cohort fertility as a function of met need for contraception and maternal education. Completed cohort fertility forecasts were then converted back to age-specific fertility rates. Net migration rates were forecasted as a function of the sociodemographic index, the crude population growth rate, and mortality due to conflict, war and natural disasters. Total (all-cause) mortality was derived from forecasting cause-specific mortality from GBD 2019, using the three-component model described in Foreman et al [4]. Cause-specific mortality was then aggregated up the GBD 2019 cause hierarchy to generate total mortality forecasts, which were utilized to compute future life tables. Using past population from GBD 2019, the CCMP model advanced populations forward and produced country-age-sex specific estimates for single future calendar years.

S4.2 Covariates

S4.2.1 List of Forecasted Variables

The following are the list of variables which are forecasted in the manuscript:

- GDP: Gross Domestic Product (national income of a country)
- GGE: General Government Expenditure (all sector government expenditure in a country)
- DAH_d: Development Assistance for Health donated
- DAH_r: Development Assistance for Health received
- GHE: Government Health Expenditure
- OOP: Out-of-pocket Private Expenditure
- PPP: Prepaid Private Expenditure
- THE: Total Health Expenditure

S4.2.2 Covariates Used for Forecasting

The following covariates are used as predictors, or independent variables, in our models:

- Total population of a country (1950 - 2050)
- Proportion of total population below the age of 15 (1950 - 2050)
- Proportion of total population above the age of 64 (1950 - 2050)
- Educational attainment per capita above the age of 15 (1980 - 2050)
- An indicator variable used to denote the anomalous disbursement of DAH in our DAH_d forecasts (1 for years in 2000 - 2010, 0 otherwise)
- An indicator variable used to denote the Ebola crisis in Guinea, Sierra Leone and Liberia in our DAH_r forecasts (1 for years in 2014 and 2015 for the three aforementioned countries, 0 otherwise).

Additionally, we also use the forecasted GDP and GGE per capita as covariates to predict the health expenditure variables. Figure S4.1 in the Tables and Figures section shows our full pathway for forecasting all our endogenous variables and how each of those variable fed into a succeeding model.

In order to forecast GDP per capita, we first convert GDP per capita to GDP per working population in the following manner:

$$\text{GDP}_{\text{working population}} = \text{GDP}_{\text{total population}} \times \frac{\text{Working population (20-64 years old)}}{\text{Total Population}}$$

We defined working age population as ages 20-64; this age group was chosen because it yielded the best model fit in tests, which also considered a minimum working age of 15 years and a maximum working age of 70 years. We forecast GDP per working population from 2022-2050 and convert back to GDP per capita for further analysis. The out-of-sample root-mean-squared-error (RMSE) based on using GDP per working population was smaller than forecasting with GDP per capita.

S4.3 Ensemble Modeling

The purpose of ensemble modeling is to make sure that we capture the most out of what we have in our arsenal in terms of covariates and model specifications. We are agnostic about one model being the sole predictor of the future, and allow an ensemble of beliefs about predicting off of the past trends. 'Ensembling', in simple terms, is a way of pooling a number of sub-models, where the space of sub-models span different inclusions and combinations of predictors, and/or different econometric specifications.

S4.3.1 Sub-model Setup

Our basic sub-model is a linear mixed effect model of the following form, for country i and time t:

$$\Delta_t Y_{i,t} = (\alpha + \alpha_i) + \left(\sum_{p=1}^{3} \rho_p \Delta_t Y_{i,t-p} \right) + \Delta_t X' \beta + \varepsilon_{i,t} \tag{1}$$

where

$$\left(\alpha, \vec{\beta}\right) \quad \text{(Fixed effects)} \tag{2}$$

$$\alpha_i \sim N(0, \sigma_a^2) \quad \text{(Country specific random intercept)} \tag{3}$$

$$\varepsilon_{i,t} \sim N\left(\sum_{m=1}^{3} \phi_m \varepsilon_{i,t-m}, \sigma_i^2 \right) \quad \text{(Autocorrelated residuals as time random effects)} \tag{4}$$

$$\left(\sum_{p=1}^{3} \rho_p \Delta_t Y_{i,t-p} \right) \quad \text{(Autoregressive terms)} \tag{5}$$

'Fixed effect' is the equivalent of a non-random coefficient in a linear regression setting, and is estimated globally across all countries and time periods. The likelihood function for the data were all set to be Gaussian distributions for all of our metrics and models. Due to the instability of generating long-term forecasts using nonlinear models, we assumed linearity in the submodels, however, we incorporated nonlinear effects in our ensemble of submodels through features such as ARIMA terms, convergence terms, non-linear transformations, and forecasting in first-difference space. We checked for the stationarity of our retrospective GDP

data to ensure that the time-series is not non-stationarity and to inform our forecast model specifications. We employed panel unit root tests sourced from a time-tested R library "plm". Two standard Unit Root tests for panel data were used: Levin-Lin-Chu and Maddala-Wu and we specified that the exogeneous variables were individual intercepts and time trends. The former yielded a p-value of 9.234e-4 and the latter yielded a p-value of less than 2.2e-16. These test results indicate that a rejection of the null hypothesis of non-stationarity.

S4.3.2 Covariates

Using our linear baseline model as defined in section S4.3.1, we created our set of ensemble sub-models by using all combinations of each of the covariates in Table S4.2. For example, if we were predicting GGE per GDP with Educational attainment and Population as the predictors, then we would get a possible combination of four specifications to use (including one with no fixed effects). All covariates were included as fixed effects, and all specifications had the random effect on country intercepts. The proportion of submodels used that contain particular submodel specifications are described in Table S4.3.

S4.3.3 Convergence to Global Growth Rates

We believe that the very-long-term trajectory of a variable forecasted for each country will gradually taper to the global growth rates. The way that we implemented this growth rate convergence is in the following manner:

1) Suppose that we are forecasting $Y_{i,t}$, which includes an intercept at the global level and the country level. Let us also assume that there were $\widetilde{T}$ time periods of Y observed in the past for each country i.

2) We start the tapering of the growth rate to start from 10 years before $\widetilde{T}$ into the future starting from the present, converging to the global value 10 years after $\widetilde{T}$. Therefore, if the present time period is H, then our decyaing scheme will start off from the year $h + \widetilde{T} - 10$ and end at $h + \widetilde{T} + 10$.

3) Our main objective is to slowly taper all the countries' random intercepts to decay to zero, so that they are all following a global intercept. In a first-difference model, since the intercepts represent the linear time trend, this scheme will slowly converge the linear time trends of all countries to decay to the global time trend.

4) Starting from the first year of decay, we will transform the country specific intercepts as

such:

$$\hat{\alpha}_i = (\hat{\alpha}_i) \times \frac{20 - t}{20}$$

where t are all the time periods between $h + \widetilde{T} - 10$ and $h + \widetilde{T} + 10$.

5) By the time t reaches $h + \widetilde{T} + 10$, the value of $\hat{\alpha}_i$ will have decayed to zero, and therefore, all the countries will have reached a global linear time trend estimate.

S4.3.4 Specifications

1) **ARIMA (Autoregressive Integrated Moving Average [7]) terms**: We allowed up to three degrees of lags in the model (traditional auto-regressive terms), where each degree of AR term will include itself and all other lower degrees of lags. For example, a GDP per capita model with AR(3) specification (predicting log of GDP per capita) will include once, twice and thrice lagged log GDP per capita term. These were included as fixed effects.

   Additionally, in order to predict the best set of fixed effect coefficients, we test and include auto-correlated residuals in our models (traditional moving-average terms in an ARIMA setup). This basically means that we allow our models to estimate the residuals with an autoregressive process of their own. These were included as random effects, and we allowed this to exist at the country-year level.

   All of the variables we forecasted used the first-differences transformation (across time) as the dependent variable, except DAH received which was forecasted in non-differenced space, because of the presence of very high noise, thereby making the differenced series very unstable to forecast.

2) **Recency weights**: One of the other specifications we included in our sub-models was the option of weighting the recent years higher. This is particularly helpful for countries like Ethiopia and Nigeria, where they had rapid economic growth in recent years, and we believe that is a better predictor of the GDP forecasts than the further past. The weighting function was defined as such:

$$Weight = f(\tau, t) = (T - t + 1)^{\tau} \tag{6}$$

   where T is the final year of in-sample data we have, and t is the year at that data point. This is an exponential decaying weighting function, where the degree of decaying is determined by τ, and we test and include a set of values of τ ranging between 0 and

0.5, where $\tau = 0$ refers to equal weights (all time periods are weighed equally at 100). Given that $\left.\frac{df(\tau,t)}{dt}\right|_{\tau>0} < 0$, this allows to weight our data with higher contributions to the likelihood from the recent past, and slowly decaying as we move further into the past towards the first observed time period. These recency weights were multiplied with the pointwise data variance parameter in order to weight the data points.

3) **Convergence Term**: We also allowed for the inclusion of a 'convergence term' in the list of sub-models. A convergence term is the one-year lag of the non-differenced dependent variable, and gets updated as each year is forecasted in the future. If a convergence term was co nsidered in a sub-model, then we only included that sub-model if the coefficient on the convergence term was estimated to be negative (and statistically significant at 10% level).

S4.4 Inclusion and Exclusion Criteria

After we ran all possible combination of our sub-models and created a mean set of forecasts, we only want to keep the best possible set of sub-models. Hence, we implied the following set of inclusion and exclusion criteria in order to filter out the 'unrealistic' sub-models:

(1) All of the estimated coefficients must be **statistically significant at 10% level of significance**. For the fixed effects, we took the mean and the standard deviation of the posterior estimates, and filtered out the sub-model if the absolute z-score is below 1.645 (the absolute value of the one-sided 95th quantile of a standard normal distribution).

For the random effects, we look at the whether the measure of variance is statistically significant or not. The model outputs the mean and standard deviation of the *precision of the random effects*, and therefore allowed us to exclude the specification if the precisions are not statistically significant at 10% level.

(2) If there were any estimated coefficient that **defied a prior belief we have on the direction of the value**, then we dropped that sub-model from consideration. For example, we strongly believe that as a donor country's (high income countries) income (GDP per capita) grows, they will be able to donate more DAH to lower income countries, and so, if we ran a sub-model predicting DAH_d and get a negative coefficient on GDP per capita, we dropped that sub-model from consideration.

Our prior beliefs on the covariates for each dependent variable are listed in Table S4.2.

(3) The forecasted trajectory growth **must not exceed observed growth rates**. We believe that a country will not grow faster than how much it has grown in the past trend. In order to come up with the bounds, we run a stochastic frontier analysis (SFA) of the change in the predicted variable against the level value of the predicted variable. SFA is just like an ordinary least squares specification, except with the addition of an additional 'inefficiency' term with a half-normal distribution. This allows us to estimate (for example, for GDP per capita): conditional of a country's income, how much growth rate did we see in the country's GDP. We ran this analysis across all of the observed data points, and derived a relationship binding the growth rates of GDP against the absolute values of GDP.

S4.5 Creating the Forecasts

S4.5.1 Ranking Sub-Models

In order to find out with sub-models would be able to predict a country's future the best, we ran out-of-sample predictive validity (OOS-PV) tests [8]. Simply put: we took each sub-model that passed the criteria in section S4.4, and instead of running it on all of the past data, we left out some number of recent most years. Following that, we ran the sub-model on the truncated past, and forecasted those years left out. For example, our GDP data extends from 1970 through 2021; we left out 15 years of data, and reran a sub-model from 1970 - 2005, and use the results of that sub-model to forecast GDP for the out-of-sample years (2006 - 2021).

This gave us essentially two trajectories between 2006 through 2021: the truth and the out-of-sample predictions. For each year, we computed the squared error (the difference) between these two lines, and averaged these errors for each of the neighboring years. So for example, we had squared errors for 15 data points between 2005 and 2021, and so the first mean squared error sum was just the squared error at 2006 and 2007, the second was the mean of the sum of squared errors for 2006, 2007 and 2008, and so on. We then took the square root of this new series to get the running root mean squared errors (RMSE) for a given country.

We looked at a country's 2006 RMSE values for each sub-models ran, and listed out the best 10% of the sub-models (that is, the lowest 10% RMSE values), and we did so for every year out-of-sample. For a single country, we may potentially have completely different set of sub-models for each of the OOS years. Then, for the 10% of the sub-models selected in the *first* year OOS, we only used those models to predict the *first* year of forecast for each country; the set of 10% of the sub-models selected in the *second* year OOS were used to predict the *second* year of forecast for each country, and so on, until the last year OOS model selections are used to compute the forecast the remaining years. This allowed us to narrow down every country's trajectory with the best performing OOS-PV sub-models for each year.

S4.5.2 Uncertainty Estimation

To estimate the uncertainty intervals (UI), we reran the selected, ranked sub-models from section S4.5.1 and simulated draws instead of just getting a mean estimate of the future. There are four types of uncertainty we implemented in forecasting:

(i) **Model Uncertainty** : This type of uncertainty comes from having more than one

type of specification to create forecasts, and therefore we included a set of sub-models in the ensemble to incorporate for this uncertainty (which are ranked within each country-year).

(ii) **Data Uncertainty** : If our covariates themselves had draws of the future data (for example, when we forecasted GDP as a covariate to forecast PPP), then we picked randomly from the draws of the independent variable when predicting a sub-model's trajectory, if that covariate was included.

(iii) **Parameter Uncertainty** : This type of uncertainty is due to the variance for the posterior distributions. Once we have run a sub-model, we simulated from each of the estimated posterior distribution to create a set of simulated coefficients. This is done by simulating from the joint precision matrix of all the parameters estimated, and therefore produces a correlated set of coefficients.

(iv) **Fundamental Uncertainty** : The in-sample data and the fitted line in the past will never line up perfectly: there will always be errors from the model fit. We needed to reflect this level of uncertainty in our forecasts as well. By extracting these empirical residuals produced by a sub-model, we forecasted future country-specific residuals by using a random walk process, where the variance of the process is the variance of the residuals from the model fit σ_{ε}^2:

$$\hat{\varepsilon}_{i,t} \sim N(\varepsilon_{i,\hat{t}-1}, \sigma_{\varepsilon,t}^2) \tag{7}$$

A random walk is an AR(1) process with the coefficient equal to 1: in other words, the current value is independent of last year's value (except for the starting position), and will propagate forward with a random Gaussian noise of $\sigma_{\varepsilon,t}^2$ variance.

All of the above were used to simulate 500 forecasts (or draws), and so in order to construct our UIs, we took the mean and the values of the 2.5th and 97.5th quantiles to estimate 95% UIs.

Table S4.4 shows all of the retrospective and prospective estimates of our health spending outcomes in 2021 US dollars and 2021 purchasing-power parity-adjusted dollars in per capita space.

S4.6 Ad-hoc Draws Correlation

S4.6.1 Motivation

Given our current setup of compiling draws for each single year, there is no way of enforcing a temporal correlation across the draws right from the ensemble architecture. For example, the sub-models used in the first 15 years of GDP per capita forecasts were independently constructed and only depended on OOS-PV fits, while the 16th year onwards all draw from the same set of sub-models. This section details on the method used to generate the same correlation in the first 15 years of that example, drawing from the existing correlation from the 16th year onwards.

S4.6.2 Bivariate Correlated Distributions

Using the GDP per capita example: following from uncertainty estimation, once we generated approximately 500 forecast draws for a country and year for any of the covariates, we used the following strategy to achieve consistent temporal correlation across all time periods in the future:

(a) For each country, we recorded country-specific Spearman's correlation coefficient across all draws between 2036 and 2050, which gave us a country-specific correlation vector.

(b) For each value of correlation in step (a), we simulated a bivariate uniform distribution for each country and year (2022 through 2036). This simulated distribution was ranked in such a way that the marginal distributions in the joint distribution were correlated with that value of correlation coefficient we supplied (this joint distribution is known as a *copula*).

(c) We recorded the ranks of the copula, and sorted our draws (within each country) using those temporal ranks, and therefore we ended up with a complete time-series data for all draws, such that each country and year will follow the same rank correlation structure that exists between 2036 and 2050.

(d) Finally, we calculated our final set of uncertainty intervals by taking the 2.5th and 97.5th percentiles of these correlated draws.

We used this method at the end of forecasting every metric, since one metric fed into the other sequentially.

S4.7 Future Health Scenarios

We established the trajectories that our health expenditures are expected to take through 2050 using our ensemble models (from hereby referred to as the 'reference' case). The reference forecasts were built upon the basis of each country's past trends and expected future trends from covariates. We additionally also predicted what the possible trajectories for each country would look like if they were to follow the possible optimistic and pessimistic growth rates observed globally (referred to as 'greater' and 'lesser' cases, respectively).

S4.7.1 Long-term Growth Regressions

In order to determine what the possible greater and lesser growth rates for each country would be, we ran long-term growth regressions with the following specification:

$$Y_{i,T} - Y_{i,t} = \alpha + \beta Y_{i.t} + \epsilon_{i,t} \tag{8}$$

where the dependent variable represented the long term growth rate of Y for country i, which was computed either as logarithmic or logistic growth rates (for fractions).

The only independent variable we used ($Y_{i,t}$) was the value of Y_i at time t, and it served as a convergence term in this regression. This allowed us to predict the long-term growth rates of Y, conditional on a country's level of Y at time t.

S4.7.2 Forecasting Greater and Lesser Scenarios

In order to estimate what the future greater and lesser trajectories would be for each country, we followed these steps (assuming that we are forecasting from 2020 through 2050, with observed data between 1995 and 2019):

(i) We computed the 85th and 15th percentiles of the empirical residuals ϵ, as $Q_{0.85}(\hat{\epsilon_{i,t}})$ and $Q_{0.15}(\hat{\epsilon_{i,t}})$ respectively, where $Q_p(\cdot)$ is a quantile function for a percentile p.

(ii) We computed the starting annualized growth rate from the fitted scenario regression, such that, for country i:

$$\text{Greater growth rate} = \exp(\hat{\alpha}) \times \exp(Q_{0.85}(\hat{\epsilon_{i,t}})) \times (Y_{i,2019}^{\hat{\beta}})^{(1/(2019-1995))} \tag{9}$$

$$\text{Lesser growth rate} = \exp(\hat{\alpha}) \times \exp(Q_{0.15}(\hat{\epsilon_{i,t}})) \times (Y_{i,2019}^{\hat{\beta}})^{(1/(2019-1995))} \tag{10}$$

(iii) Finally, once we have established the growth rates as a function of the convergence term, we recursively created greater and lesser trajectories, conditional on the updated

growth rates every year, such that:

$$Y_{i,t+1} = Y_{i,t} \times \exp(\hat{\alpha}) \times \exp(Q_{0.85}(\hat{\epsilon_{i,t}})) \times (Y_{i,t}^{\hat{\beta}}) \tag{11}$$

where $\exp(\hat{\alpha}) \times \exp(Q_{0.85}(\hat{\epsilon_{i,t}})) \times (Y_{i,t}^{\hat{\beta}})$ was the conditional growth rate for a single year.

One condition that we imposed for the computed scenarios is: the greater projection cannot be lower than the reference projection, and the lesser projection cannot be higher than the reference projection. For countries with wide forecasts where this case did happen, we moved the greater and lesser forecasts down and up to overlap on top of the reference line respectively.

S4.7.3 Uncertainty Estimation

The uncertainty intervals around a scenario were expected to take the same shape as the uncertainty around our reference forecasts. Therefore, once we have propagated a mean set of greater and lesser forecasts in section S4.7.2, we created the draws around our scenarios in the following way:

(i) We took our reference forecast's mean line and the 500 draws around that line.

(ii) We computed the deviation of the mean from each of the draws (in logarithmic or logistic transformation, depending on the space of the covariate).

(iii) We took each of the scenario mean lines and added the deviations from the previous step to the mean lines, giving us 500 draws of the scenario projections.

Figure set S4.3 visualizes the future health spending scenarios in per capita space (in 2021 US dollars).

S4.8 Package and Architecture

S4.8.1 Architecture

All analysis and forecasting were done on a parallel computing cluster with a CentOS interface. We compiled R [9] version 3.6.0 from source code on a Docker based on Debian OS, which was deployed as a Singularity container with all the necessary compilers and binaries (GCC, G++ and Fortran 8.2.0).

S4.8.2 Template Model Builder

We used the R library **TMB** [10] to run our baseline mixed effects models. TMB stands for Template Model Builder, and it's a powerful method of approximating the integral of the Gaussian probability distribution function (which doesn't exist in closed form) by using a Laplace Approximation, which gives very precise results, and is relatively faster than other approximation packages which exist like Stan or Bugs. TMB uses automatic differentiation on the negative log-likelihood function in order to optimize the model parameters, and also returns a sparse joint precision matrix (inverse of the variance-covariance matrix), from which we can simulate posterior parameter draws.

References

[1] GBD 2019 Risk Factors Collaborators. Global burden of 87 risk factors in 204 countries and territories, 1990–2019: a systematic analysis for the Global Burden of Disease Study 2019. *The Lancet*, 396(10258):1223–1249, oct 2020. doi: 10.1016/S0140-6736(20)30752-2. URL `https://doi.org/10.1016/S0140-6736(20)30752-2`.

[2] Spencer L James, Paul Gubbins, Christopher JL Murray, and Emmanuela Gakidou. Developing a comprehensive time series of gdp per capita for 210 countries from 1950 to 2015. *Population health metrics*, 10(1):12, 2012. URL `https://pophealthmetrics.biomedcentral.com/articles/10.1186/1478-7954-10-12`.

[3] Emmanuela Gakidou, Krycia Cowling, Rafael Lozano, and Christopher JL Murray. Increased educational attainment and its effect on child mortality in 175 countries between 1970 and 2009: a systematic analysis. *The Lancet*, 376(9745):959–974, 2010. URL `https://doi.org/10.1016/S0140-6736(10)61257-3`.

[4] GBD 2019 Demographics Collaborators. Global age-sex-specific fertility, mortality, healthy life expectancy (hale), and population estimates in 204 countries and territories, 1950–2019: a comprehensive demographic analysis for the global burden of disease study 2019. *The Lancet*, 396(10258):1160–1203, 2020.

[5] Spencer Preston, Patrick Heuveline, and Michel Guillot. *Demography: measuring and modeling population processes*. Wiley-Blackwell, 1 edition. Malden, MA, USA, 2000.

[6] Stein Emil Vollset et al. Fertility, mortality, migration, and population scenarios for 195 countries and territories from 2017 to 2100: a forecasting analysis for the global burden of disease study. *The Lancet*, 396(10258):1285–1306, 2020. URL `https://doi.org/10.1016/S0140-6736(20)30677-2`.

[7] Vijay Kotu and Bala Deshpande. *Data Science: Concepts and Practice*. Morgan Kaufmann Publishers, 2nd edition. Cambridge, MA, USA, 2019.

[8] Kyle J Foreman, Rafael Lozano, Alan D Lopez, and Christopher JL Murray. Modeling causes of death: an integrated approach using codem. *Population health metrics*, 10(1):1, 2012.

[9] R Core Team. *R: A Language and Environment for Statistical Computing*. R Foundation for Statistical Computing, Vienna, Austria, 2017. URL `https://www.R-project.org/`. Accessed: 2017-12-20.

[10] Kasper Kristensen, Anders Nielsen, Casper W Berg, Hans Skaug, and Brad Bell. Tmb: automatic differentiation and laplace approximation. *arXiv preprint arXiv:1509.00660*, 2015.

S4.9 Tables and Figures

Table S4.1: Definitions of health expenditure sources

Health Expenditure Type	Definition
Development assistance for health	Financial and in-kind contributions from global health channels that aim to improve or maintain health in low- or middle-income countries.
Government health expenditure as source	Comprises domestically financed government expenditure on health, including public health spending.
Out-of-pocket expenditure	Amount paid by individuals for health services.
Prepaid private health expenditure	Includes voluntary and compulsory prepayment, private insurance, and funding from non-governmental organizations.

Figure S4.1: State Space Diagram of Forecasting Components

This diagram details the order in which forecasting components are estimated, and which forecasting components are used

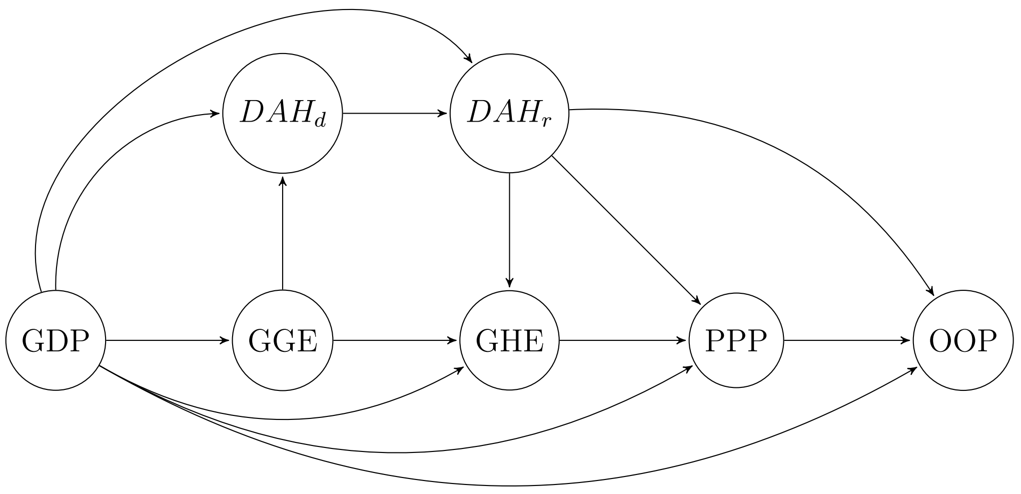

as inputs in others.

Table S4.2: Prediction and Covariates Map with Transformations

	**Predicted Variables**	**Covariates**	**Extra Specifications**
1	GDP per labour (log)		ARIMA(2-3, 1, 0)
2	GGE per GDP (logit)	GDPpc^{+}, Pop >64^{+}, Education^{+}	ARIMA(0-3, 1, 0-3)
3	DAH donated per GGE (logit)	GDPpc^{+}, GGE/GDP^{+}, Bush Era Dummy^{+}	ARIMA(0-3, 1, 0-3)
4	DAH received per total DAH donated (logit)	GDPpc^{-}, Pop <15^{+}, Ebola dummy^{+}, total DAH envelope	ARIMA(0, 0, 0)
5	GHE per GGE (logit)	GDPpc^{+}, Pop >64^{+}, GGE/GDP^{+}, DAH/GDP^{-}	ARIMA(0-3, 1, 0-3)
6	PPP per GDP (logit)	GDPpc^{+}, Pop >64^{+}, GGE/GDP. DAH/GDP, GHE/GDP,	ARIMA(0-3, 1, 0-3)
7	OOP per GDP (logit)	GDPpc^{-}, Pop >64^{+}, GGE/GDP, DAH/GDP, GHE/GDP, PPP/GDP	ARIMA(0-3, 1, 0-3)

Health Spending estimates from 1995-2050

All estimates produced for this publication can be found online in the Global Health Data Exchange (GHDx) at `https://ghdx.healthdata.org/series/financing-global-health-fgh`. The data included shows all of our health spending outcomes for every country and year from 1995 through 2050, and include 95% uncertainty intervals. The variables are in per capita measures, and for currencies 2021 US dollars and 2021 purchasing-power parity-adjusted dollars,.

Figure S4.2: Process diagram for estimating future GDP, all-sector government spending, and health spending by source

This diagram details the inputs and outputs of the ensemble modeling process.

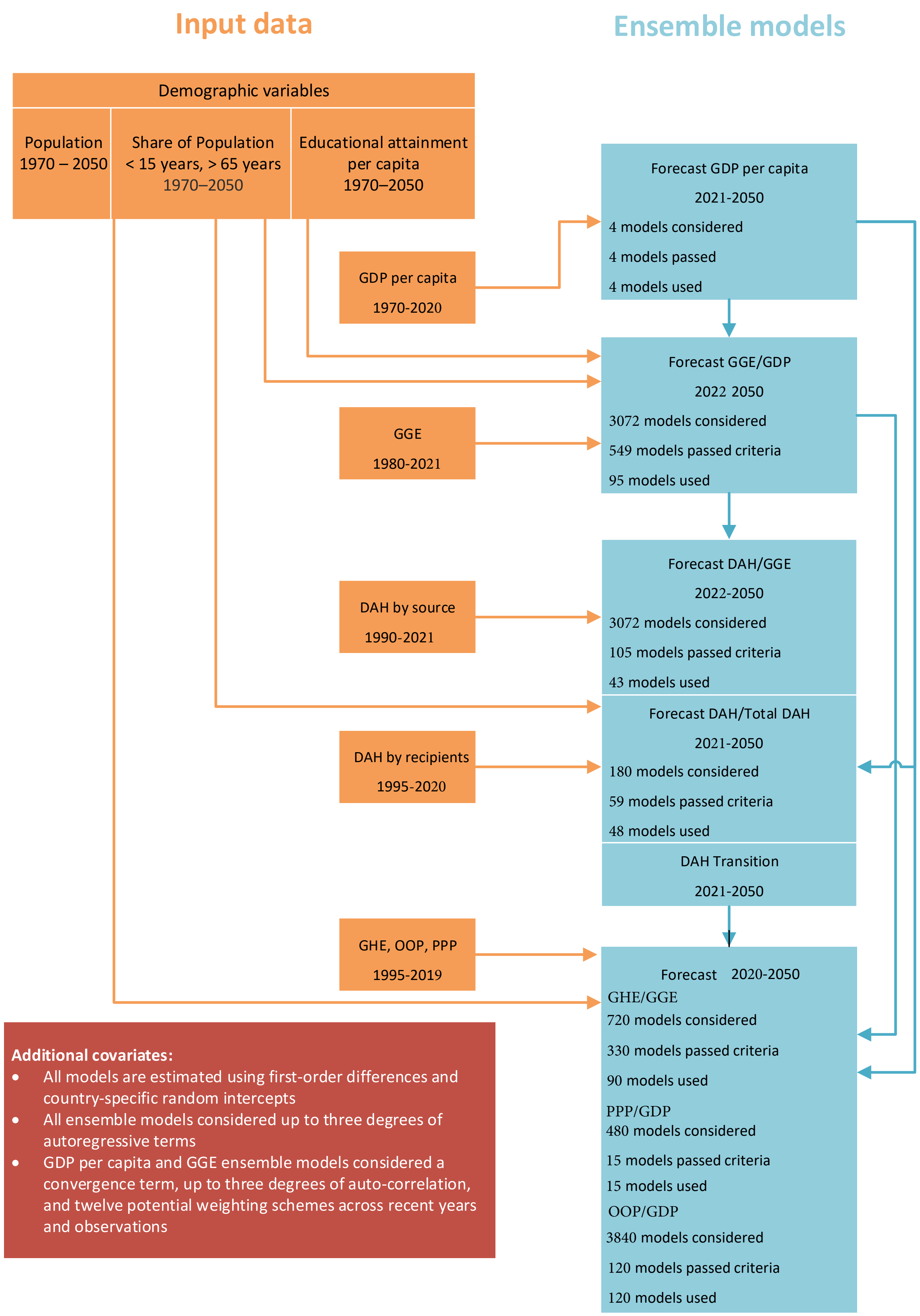
Input data
Ensemble models
Demographic variables
Population
1970 – 2050
Share of Population
< 15 years, > 65 years
1970–2050
Educational attainment
per capita
1970–2050
GDP per capita
1970-2020
GGE
1980-2021
DAH by source
1990-2021
DAH by recipients
1995-2020
GHE, OOP, PPP
1995-2019
Forecast GDP per capita
2021-2050
4 models considered
4 models passed
4 models used
Forecast GGE/GDP
2022 2050
3072 models considered
549 models passed criteria
95 models used
Forecast DAH/GGE
2022-2050
3072 models considered
105 models passed criteria
43 models used
Forecast DAH/Total DAH
2021-2050
180 models considered
59 models passed criteria
48 models used
DAH Transition
2021-2050
Forecast 2020-2050
GHE/GGE
720 models considered
330 models passed criteria
90 models used
PPP/GDP
480 models considered
15 models passed criteria
15 models used
OOP/GDP
3840 models considered
120 models passed criteria
120 models used
Additional covariates:
All models are estimated using first-order differences and country-specific random intercepts
All ensemble models considered up to three degrees of autoregressive terms
GDP per capita and GGE ensemble models considered a convergence term, up to three degrees of auto-correlation, and twelve potential weighting schemes across recent years and observations

Table S4.3: Ensemble submodel specifications used in forecasts

This table shows all submodel specifications considered in the ensemble, and the percentage of models with given specifications used in the forecasts.

	GDP		GGE		DAH Provided		DAH Received		GHE		PPP		OOP	
Number of out-of-sample years included	15		15		15		10		15		15		15	
Number of countries included	204		204		24		135		204		204		204	
Number of top models used per country-year	4		24		15		15		15		15		18	
Total number of country-year-models used	12240		73440		5400		20250		45900		45900		55080	
Retrospective data years used for model fitting	1970-2019		1980-2019 (for forecasted years >2021); 1980-2020 (for forecasted years < 2022)		1990-2019		1990-2019 (for forecasted years >2022)		1995-2019		1995-2019		1995-2019	
Covariate(s)	Value	Percentage of models	Value	Percentage of models	Value	Percentage of models	Value	Percentage of models	Value	Percentage of models	Value	Percentage of models	Value	Percentage of models
Autoregressive terms	3	50.0%	3	16.2%	3	0.0%	0	100.0%	2	23.3%	1	100.0%	3	0.0%
	2	50.0%	2	15.3%	2	0.0%			1	38.7%			2	100.0%
			1	21.9%	1	0.0%			0	38.0%				
			0	46.6%	0	100.0%								
Moving average terms	0	100.0%	3	11.7%	3	0.0%	0	100.0%	0	100.0%	0	100.0%	0	100.0%
			2	18.2%	2	53.2%								
			1	34.0%	1	46.8%								
			0	36.1%	0	0.0%								
Scaled level convergence term	Included	50.0%	Included	24.9%	Included	100.0%	Not included	100.0%	Not included	100.0%	Not included	100.0%	Not included	100.0%
	Not included	50.0%	Not included	75.1%	Not included	0.0%								
Recency weights (Values represent the tau value used in our recency weighting function described on page 10 of this appendix. Higher values indicate a stronger weight placed on recent years' data.)	0	100.0%	0.7	13.1%	0.7	12.4%	0.8	5.1%	0.2	7.0%	0.2	6.7%	0.2	7.4%
			0.636363636	13.5%	0.636363636	12.0%	0.77241	5.0%	0.185714286	7.0%	0.185714286	6.7%	0.185714286	6.8%
			0.57273	11.7%	0.57273	10.7%	0.74483	4.7%	0.17143	7.0%	0.17143	6.7%	0.17143	6.5%
			0.50909	11.6%	0.50909	8.8%	0.71724	5.2%	0.15714	6.8%	0.15714	6.7%	0.15714	6.1%
			0.44545	11.7%	0.44545	7.6%	0.68966	3.1%	0.14286	6.8%	0.14286	6.7%	0.14286	5.8%
			0.38182	10.8%	0.38182	10.0%	0.66207	3.1%	0.12857	6.7%	0.12857	6.7%	0.12857	5.8%
			0.31818	10.3%	0.31818	8.4%	0.63448	2.9%	0.11429	6.6%	0.11429	6.7%	0.11429	6.0%
			0.25455	3.4%	0.25455	6.8%	0.60690	5.0%	0.10000	6.6%	0.10000	6.7%	0.10000	6.3%
			0.19091	3.1%	0.19091	5.7%	0.57931	4.9%	0.08571	6.6%	0.08571	6.7%	0.08571	6.3%
			0.12727	3.3%	0.12727	5.7%	0.55172	5.2%	0.07143	6.5%	0.07143	6.7%	0.07143	6.6%
			0.06364	3.6%	0.06364	5.7%	0.52414	5.1%	0.05714	6.5%	0.05714	6.7%	0.05714	6.6%
			0.00000	4.1%	0.00000	6.1%	0.49655	4.4%	0.04286	6.5%	0.04286	6.7%	0.04286	6.7%
							0.46897	4.6%	0.02857	6.4%	0.02857	6.7%	0.02857	7.2%
							0.44138	4.5%	0.01429	6.4%	0.01429	6.7%	0.01429	7.7%
							0.41379	4.5%	0.00000	6.5%	0.00000	6.7%	0.00000	8.1%
							0.38621	3.5%						
							0.35862	3.3%						
							0.33103	3.3%						
							0.30345	1.8%						
							0.27586	1.8%						
							0.24828	1.8%						
							0.22069	3.4%						
							0.1931	3.0%						
							0.16552	2.9%						
							0.13793	1.5%						
							0.11034	1.4%						
							0.08276	1.4%						
							0.05517	1.3%						
							0.02759	1.3%						
							0	1.3%						
Proportion of population under age 15							Included	8.2%						
							Not included	91.8%						
Proportion of population over age 65			Included	100.0%					Included	56.0%	Included	0.0%	Included	0.0%
			Not included						Not included	44.0%	Not included	100.0%	Not included	100.0%
GDP per capita			Included	0.0%	Included	39.6%	Included	100.0%	Included	0.0%	Included	0.0%		
			Not included	100.0%	Not included	60.4%	Not included	0.0%	Not included	100.0%	Not included	100.0%		
GGE per GDP					Included	0.0%			Included	0.0%				
					Not included	100.0%			Not included	100.0%				
DAH Received per GDP									Included	0.0%	Included	0.0%	Included	52.4%
									Not included	100.0%	Not included	100.0%	Not included	47.6%
GHE per GDP											Included	0.0%	Included	0.0%
											Not included	100.0%	Not included	100.0%
PPP per GDP													Included	55.2%
													Not included	44.8%
Educational attainment per capita			Included	2.8%										
			Not included	97.2%										

Figure S4.3: Future Health Spending Scenarios

This set of figures included show fix panels of the dependent variables that we forecasted, starting with DAH, GHE, PPP, OOP and THE per capita. Each variable contains the respective reference, greater and lesser scenarios. The currency is 2021 US dollars.

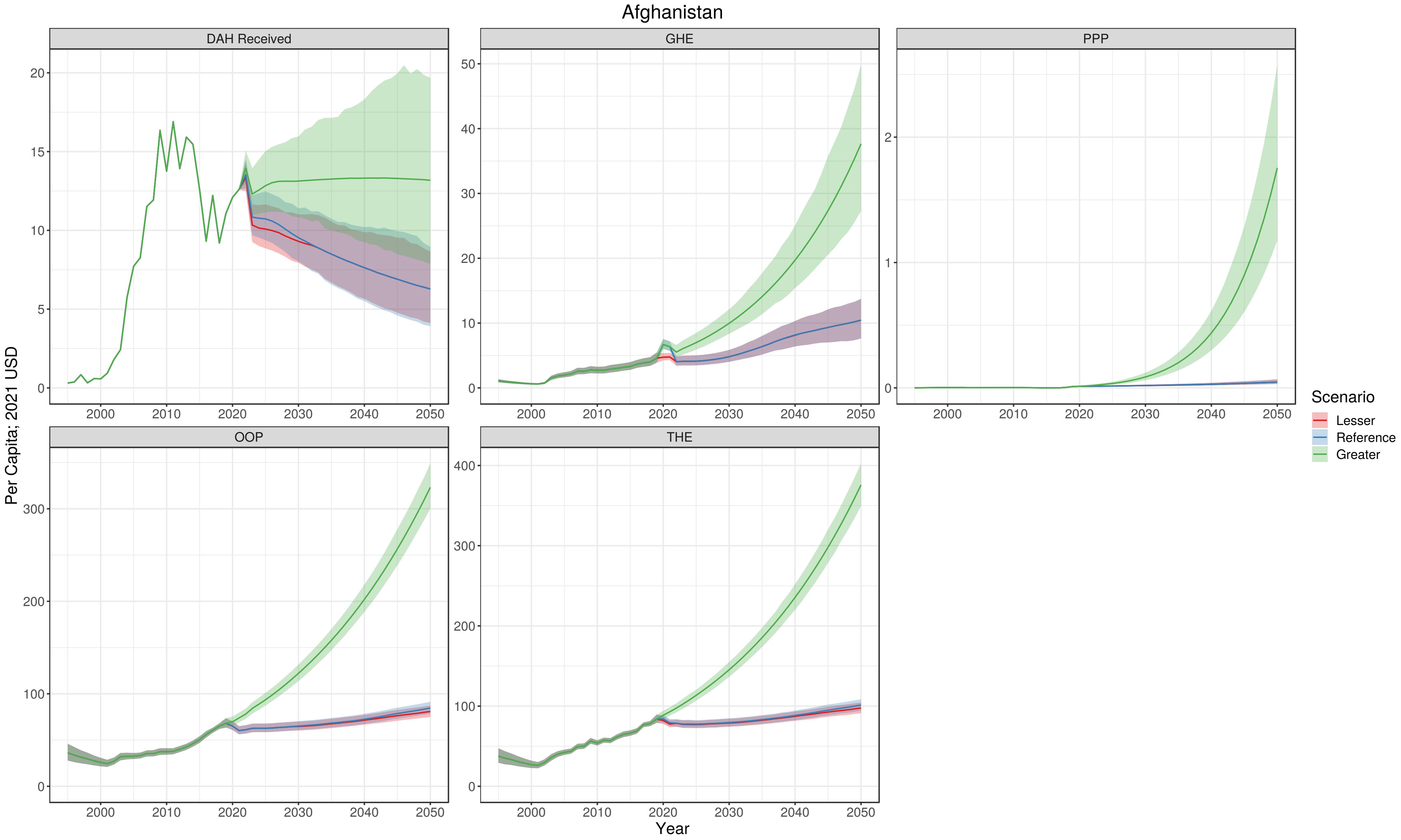

Afghanistan
DAH Received
GHE
PPP
OOP
THE
Per Capita; 2021 USD
Year
Scenario
Lesser
Reference
Greater

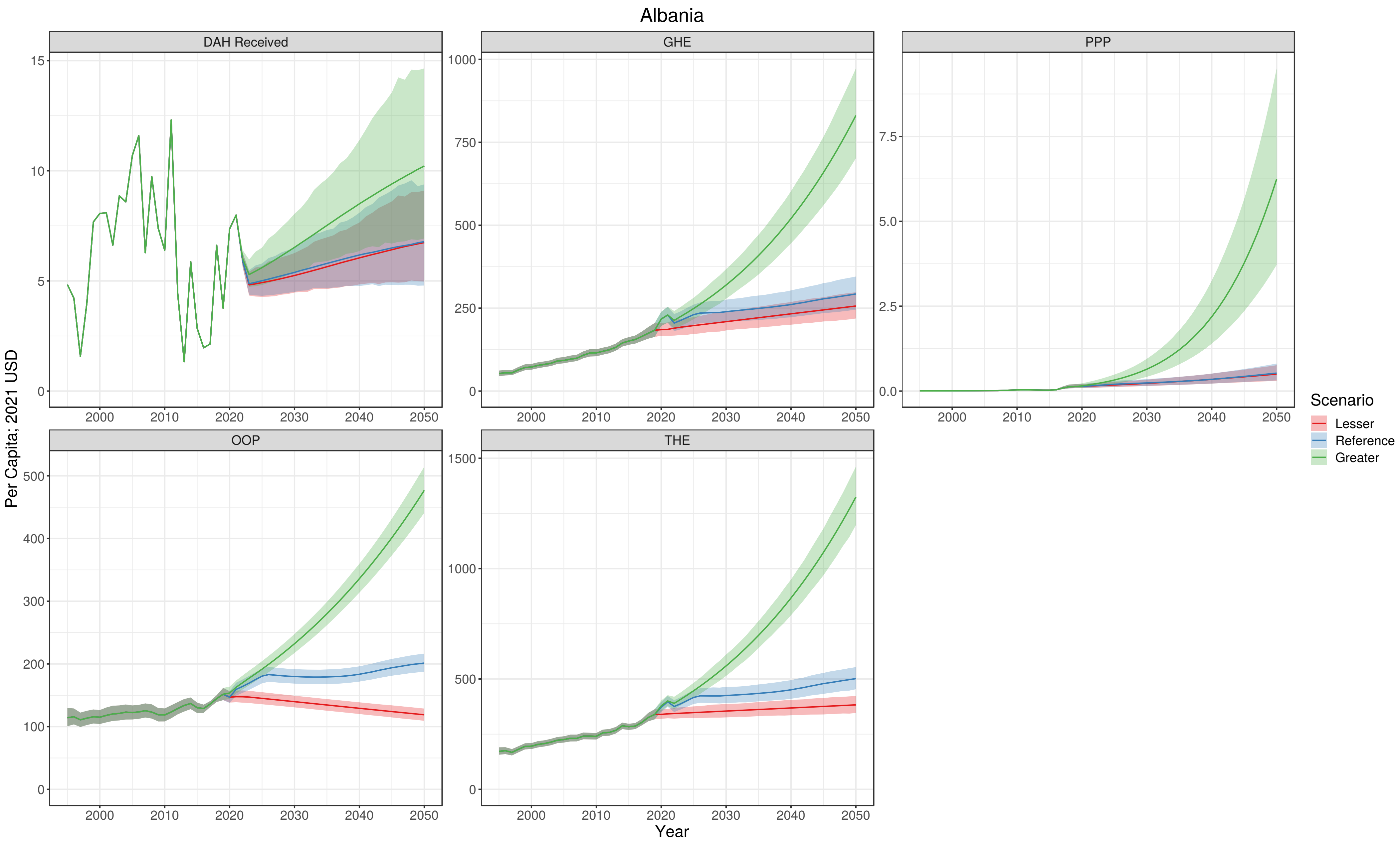

Albania
DAH Received
GHE
PPP
OOP
THE
Per Capita; 2021 USD
Year
Scenario
Lesser
Reference
Greater

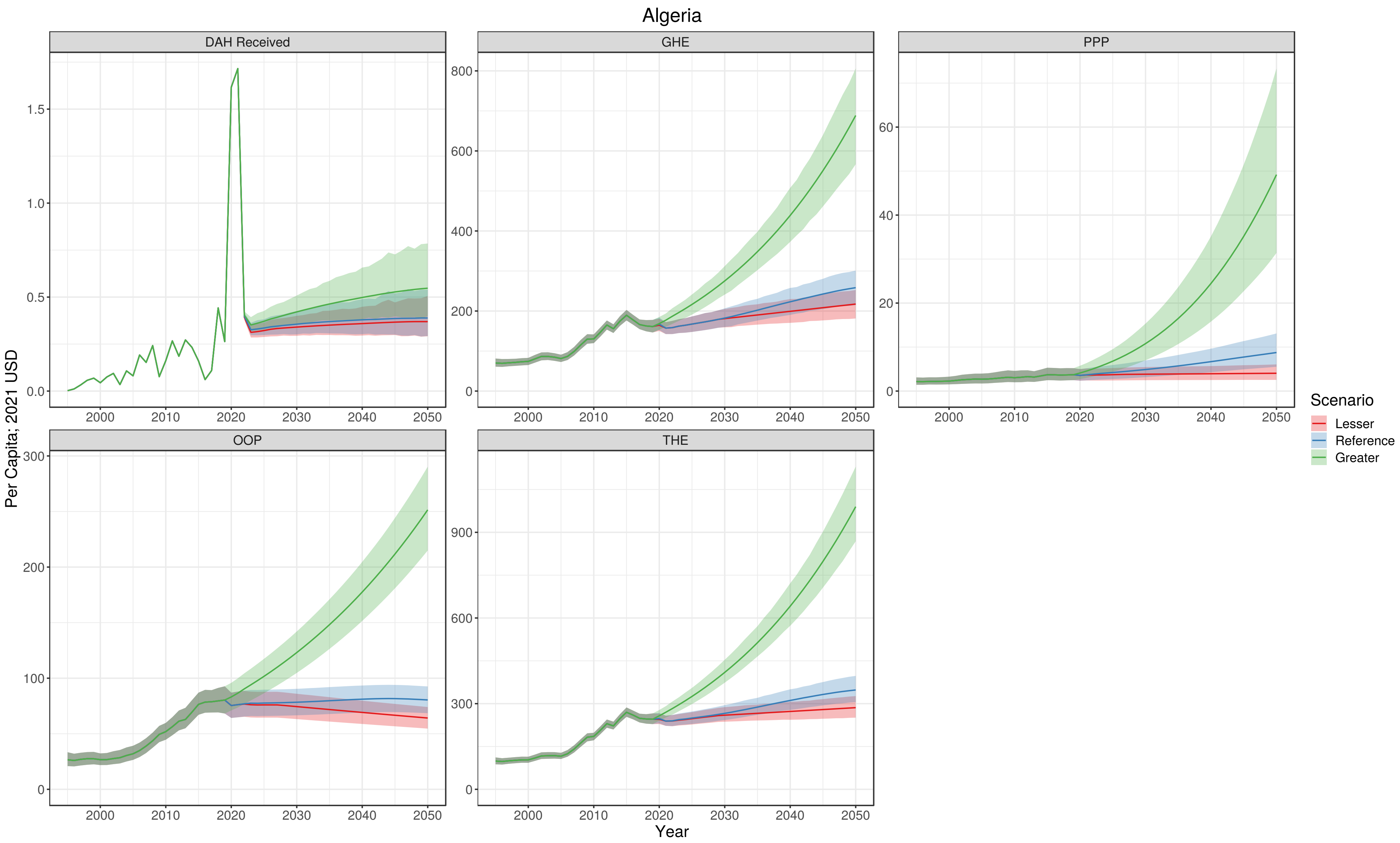

Algeria
DAH Received
GHE
PPP
OOP
THE
Per Capita; 2021 USD
Year
Scenario
Lesser
Reference
Greater
0.0
0.5
1.0
1.5
0
200
400
600
800
0
20
40
60
0
100
200
300
0
300
600
900
2000
2010
2020
2030
2040
2050

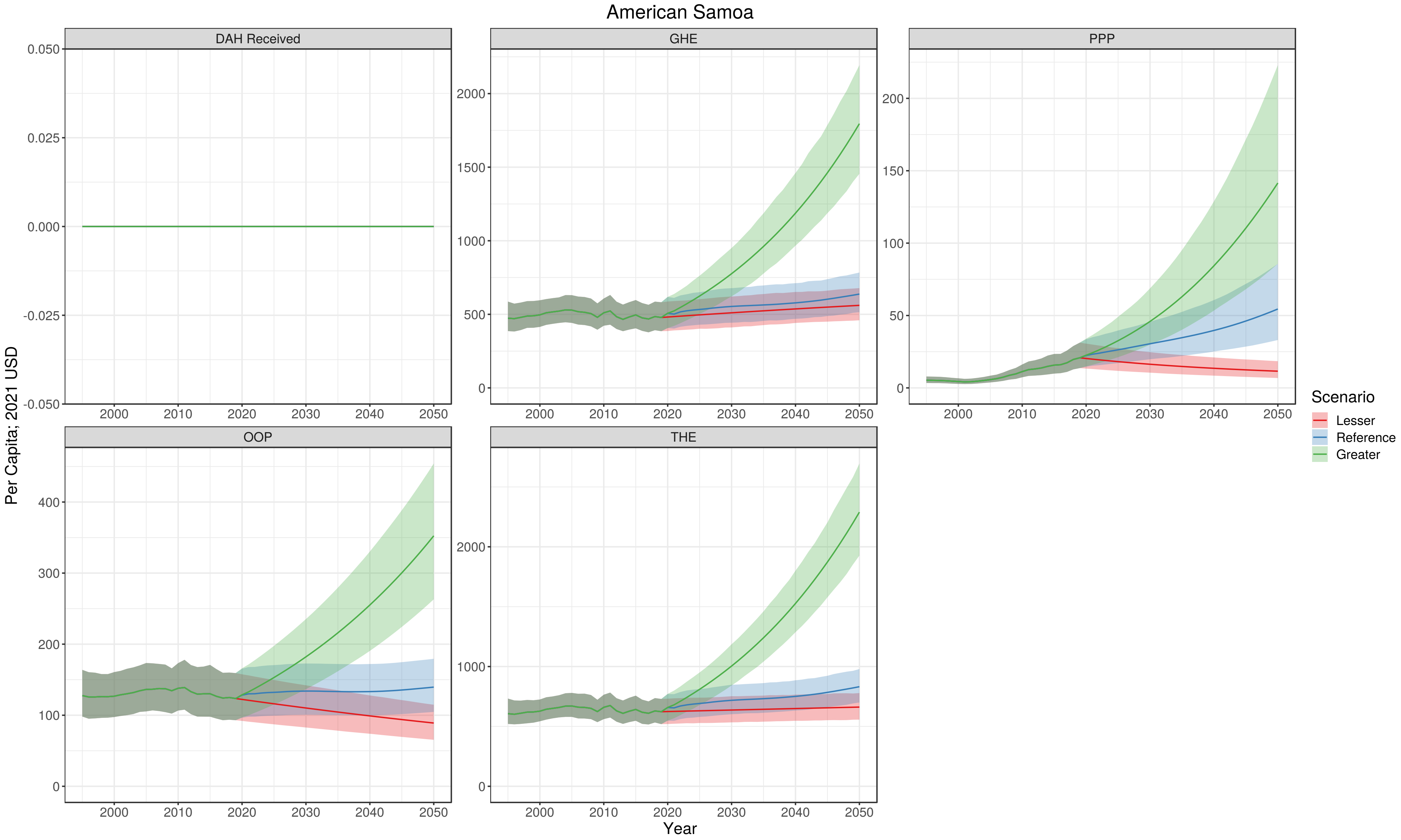

American Samoa
DAH Received
GHE
PPP
OOP
THE
Per Capita; 2021 USD
Year
Scenario
Lesser
Reference
Greater

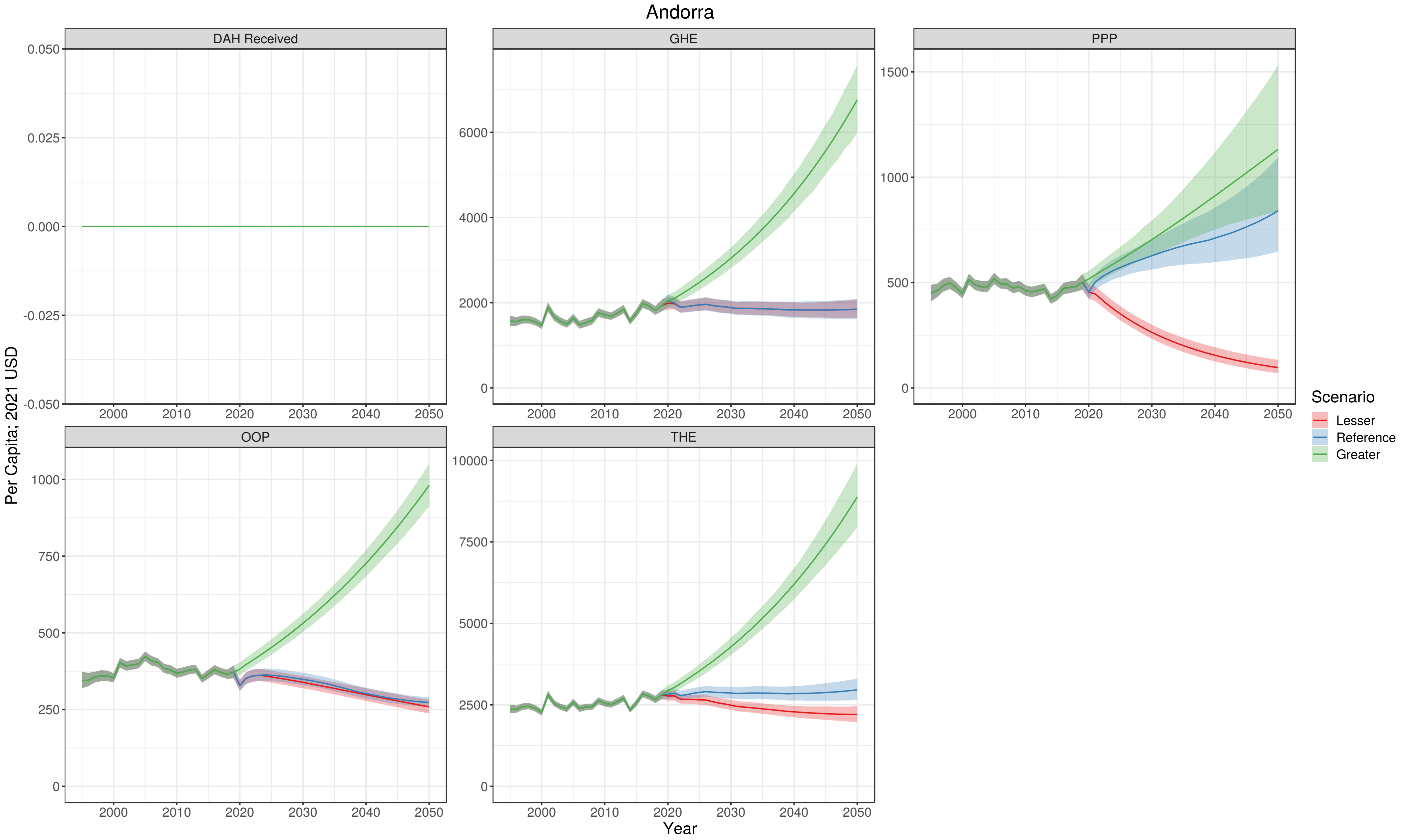
Andorra
DAH Received
GHE
PPP
OOP
THE
Per Capita; 2021 USD
Year
Scenario
Lesser
Reference
Greater
0.050
0.025
0.000
-0.025
-0.050
6000
4000
2000
0
1500
1000
500
1000
750
500
250
10000
7500
5000
2500
2000
2010
2020
2030
2040
2050

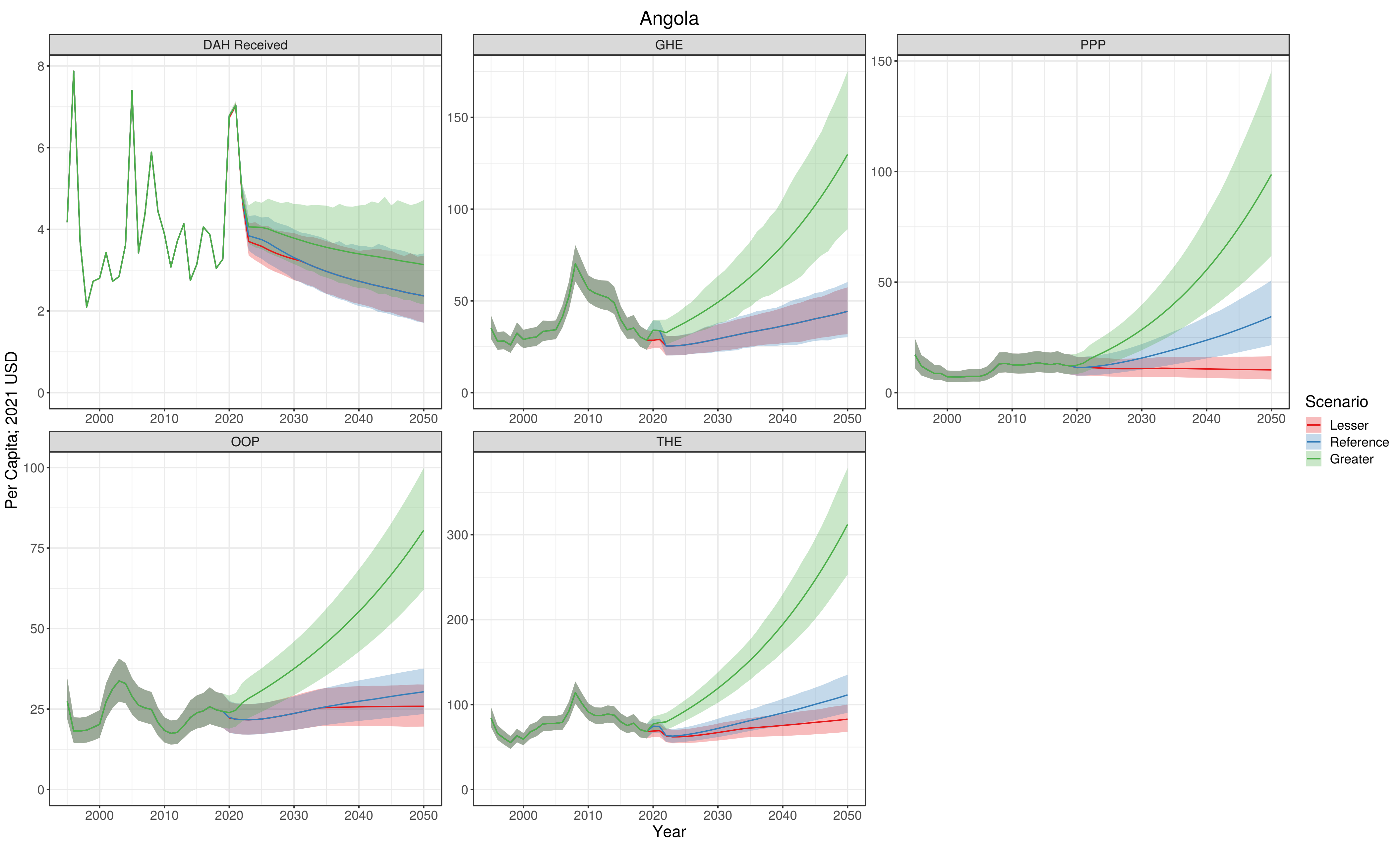

Angola
DAH Received
GHE
PPP
OOP
THE
Per Capita; 2021 USD
Year
Scenario
Lesser
Reference
Greater

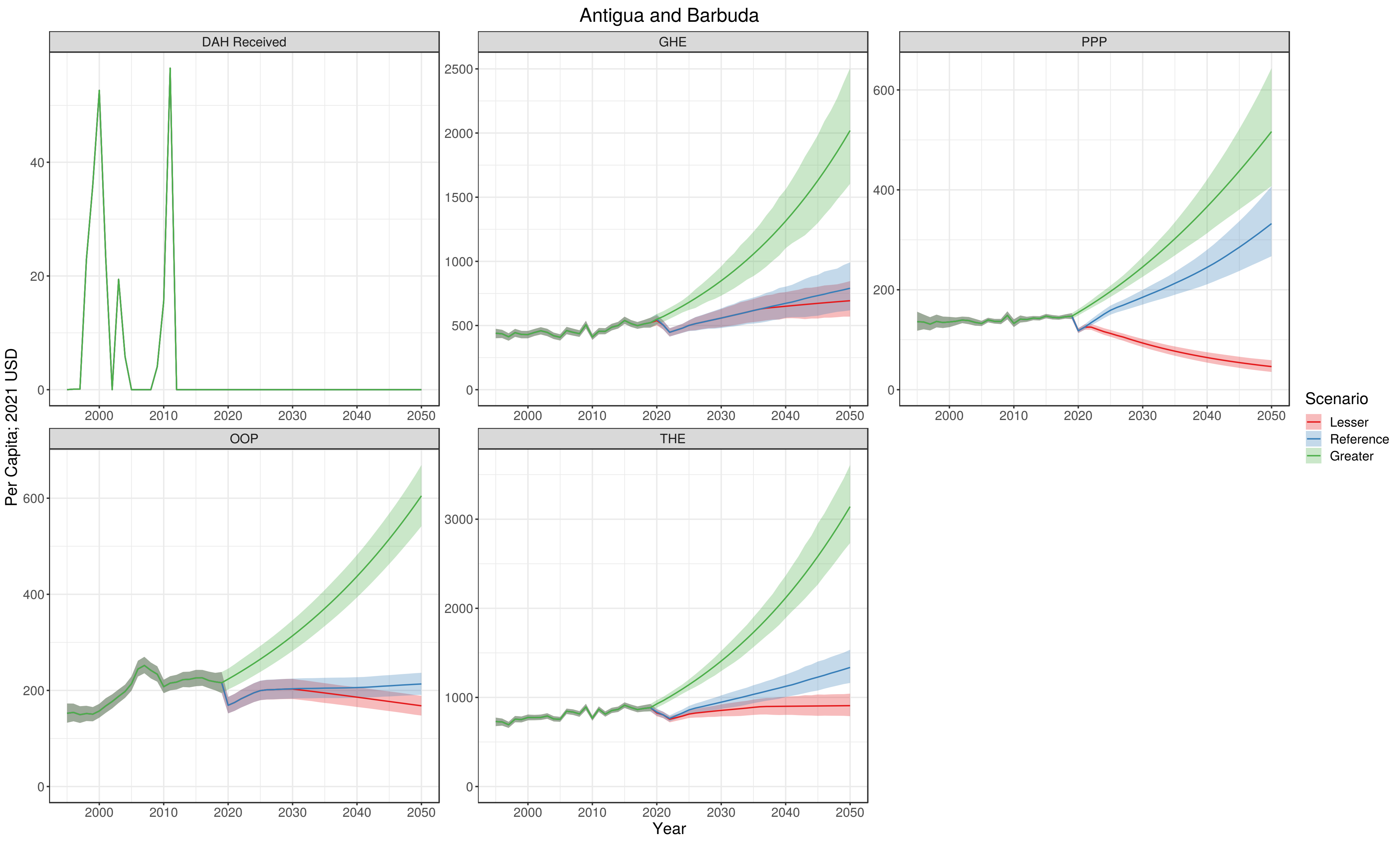

Antigua and Barbuda
DAH Received
GHE
PPP
OOP
THE
Per Capita; 2021 USD
Year
Scenario
Lesser
Reference
Greater
0
20
40
500
1000
1500
2000
2500
200
400
600
3000
2000
2010
2020
2030
2040
2050

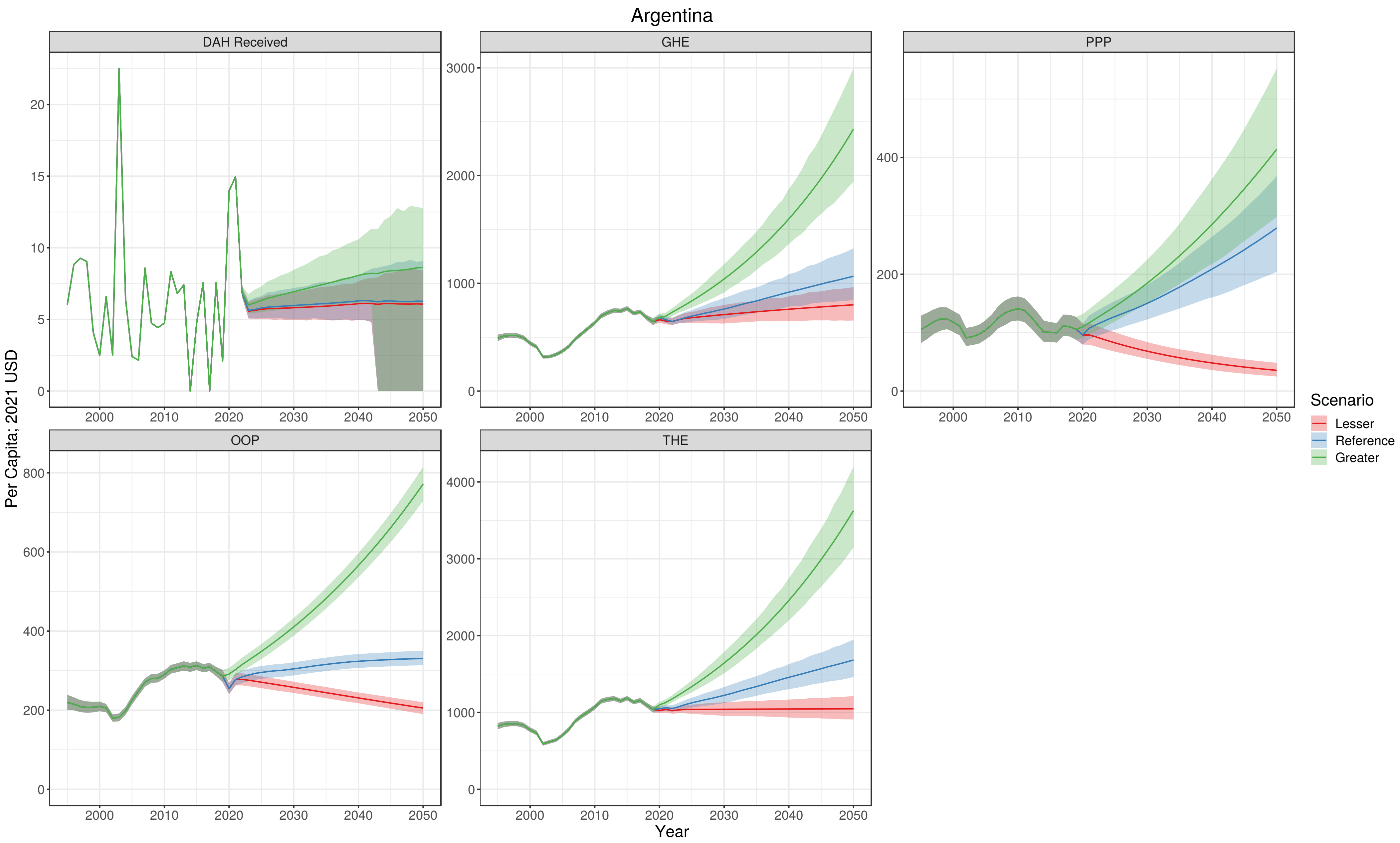

Argentina
DAH Received
GHE
PPP
OOP
THE
Per Capita; 2021 USD
Year
Scenario
Lesser
Reference
Greater

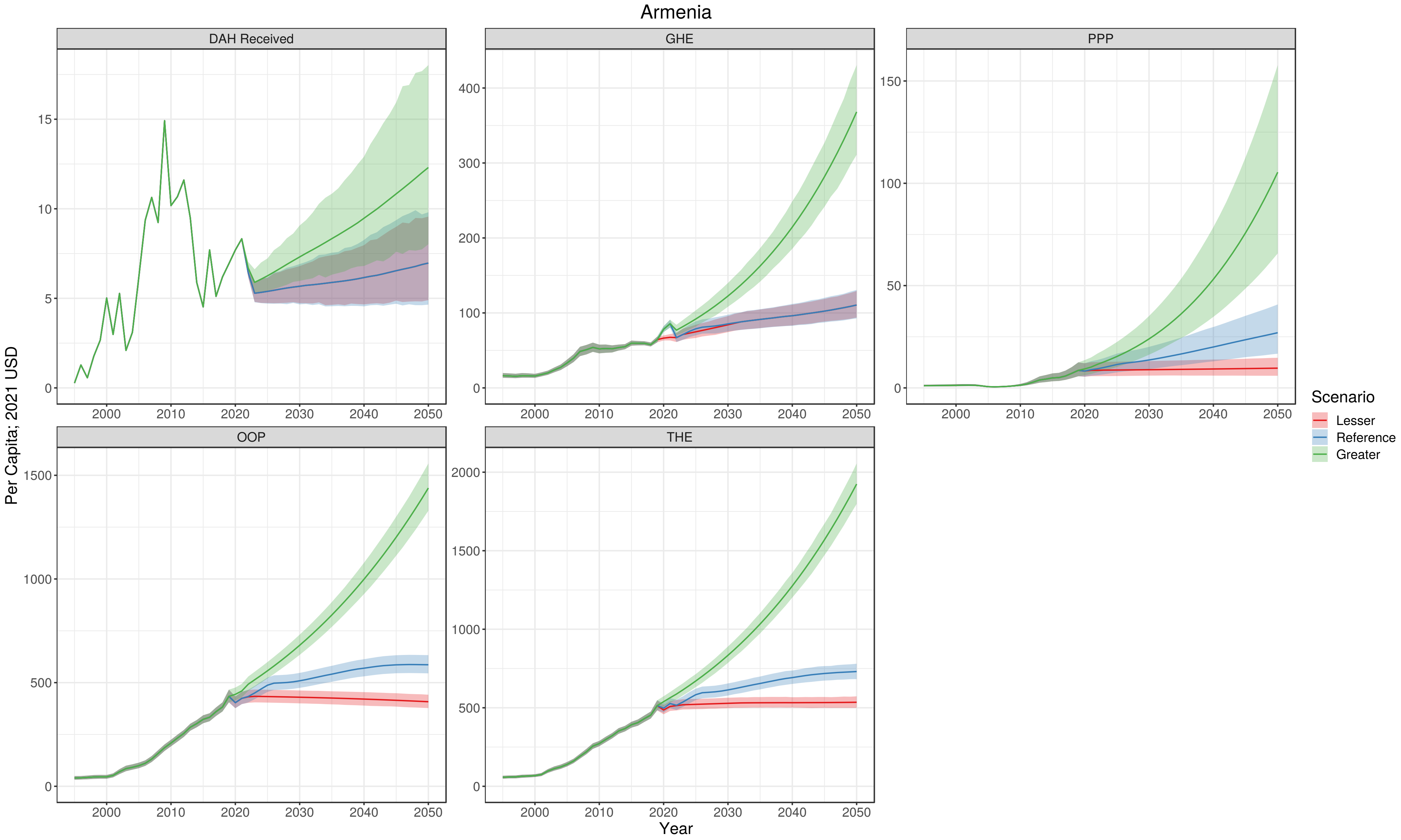

Armenia
DAH Received
GHE
PPP
OOP
THE
Per Capita; 2021 USD
Year
Scenario
Lesser
Reference
Greater

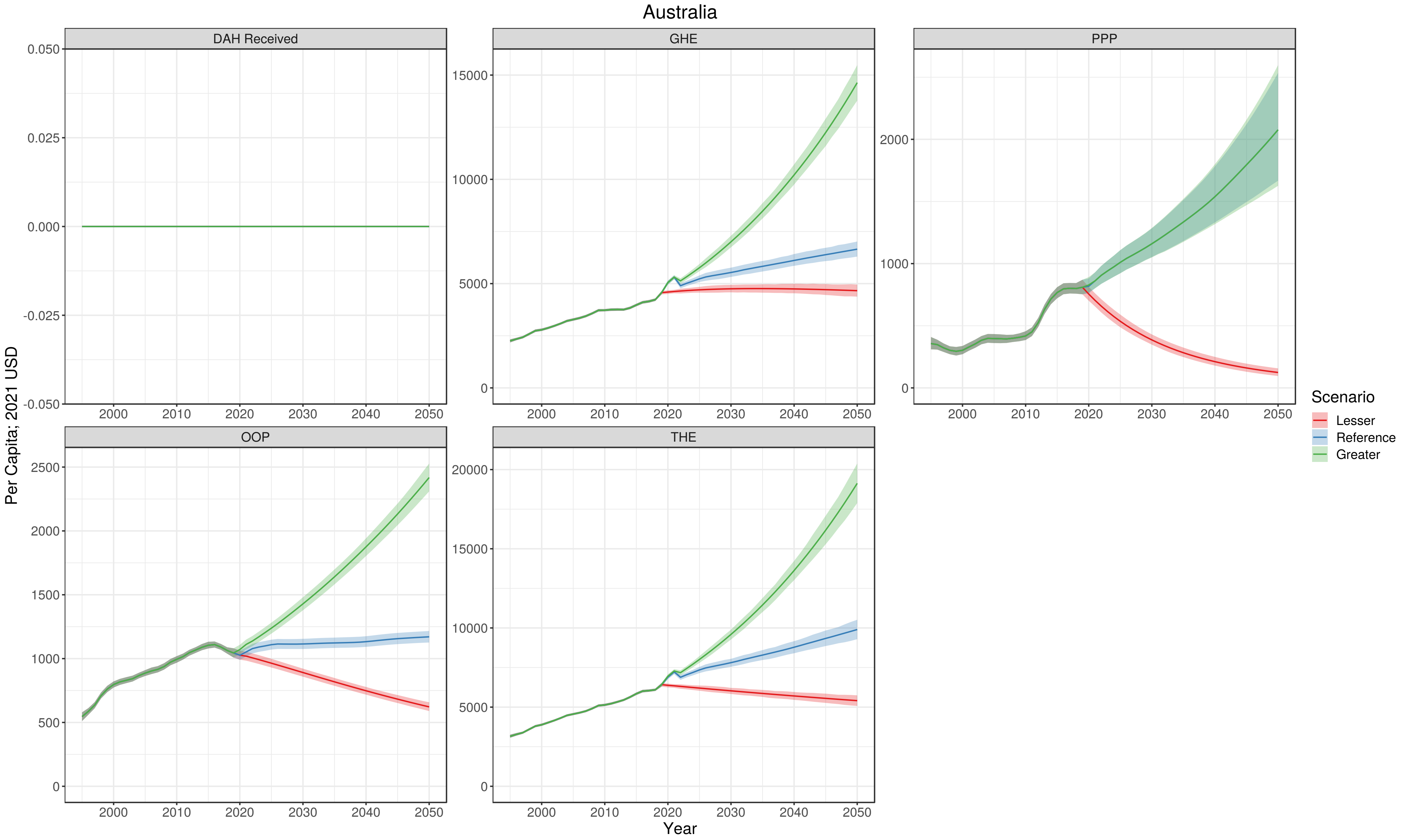

Australia
DAH Received
GHE
PPP
OOP
THE
Per Capita; 2021 USD
Year
Scenario
Lesser
Reference
Greater
0.050
0.025
0.000
-0.025
-0.050
15000
10000
5000
0
2000
1000
2500
1500
500
20000
2000
2010
2020
2030
2040
2050

Austria

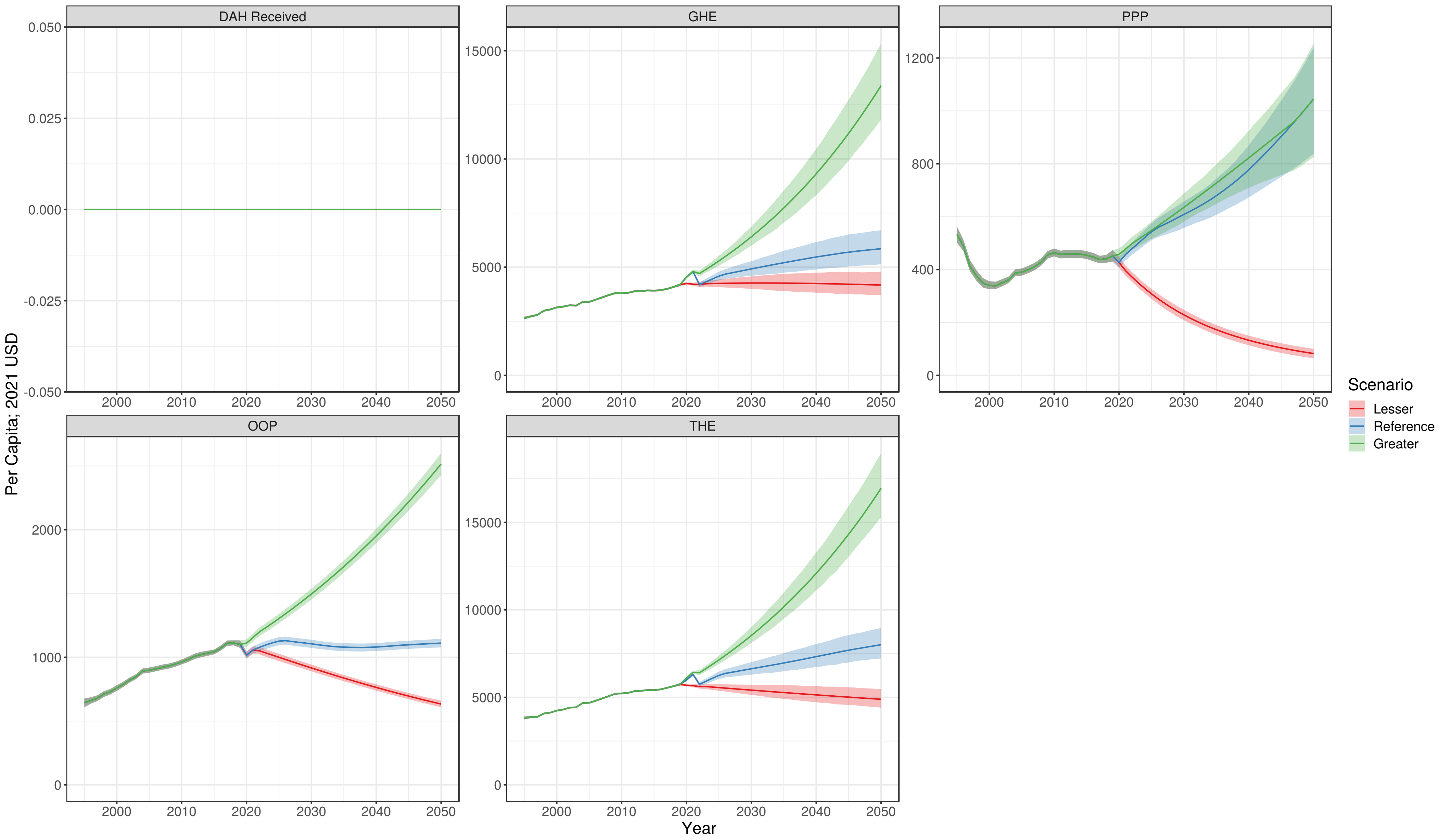

Austria
DAH Received
GHE
PPP
OOP
THE
Per Capita; 2021 USD
Year
0.050
0.025
0.000
-0.025
-0.050
15000
10000
5000
0
1200
800
400
2000
1000
2000
2010
2020
2030
2040
2050
Scenario
Lesser
Reference
Greater

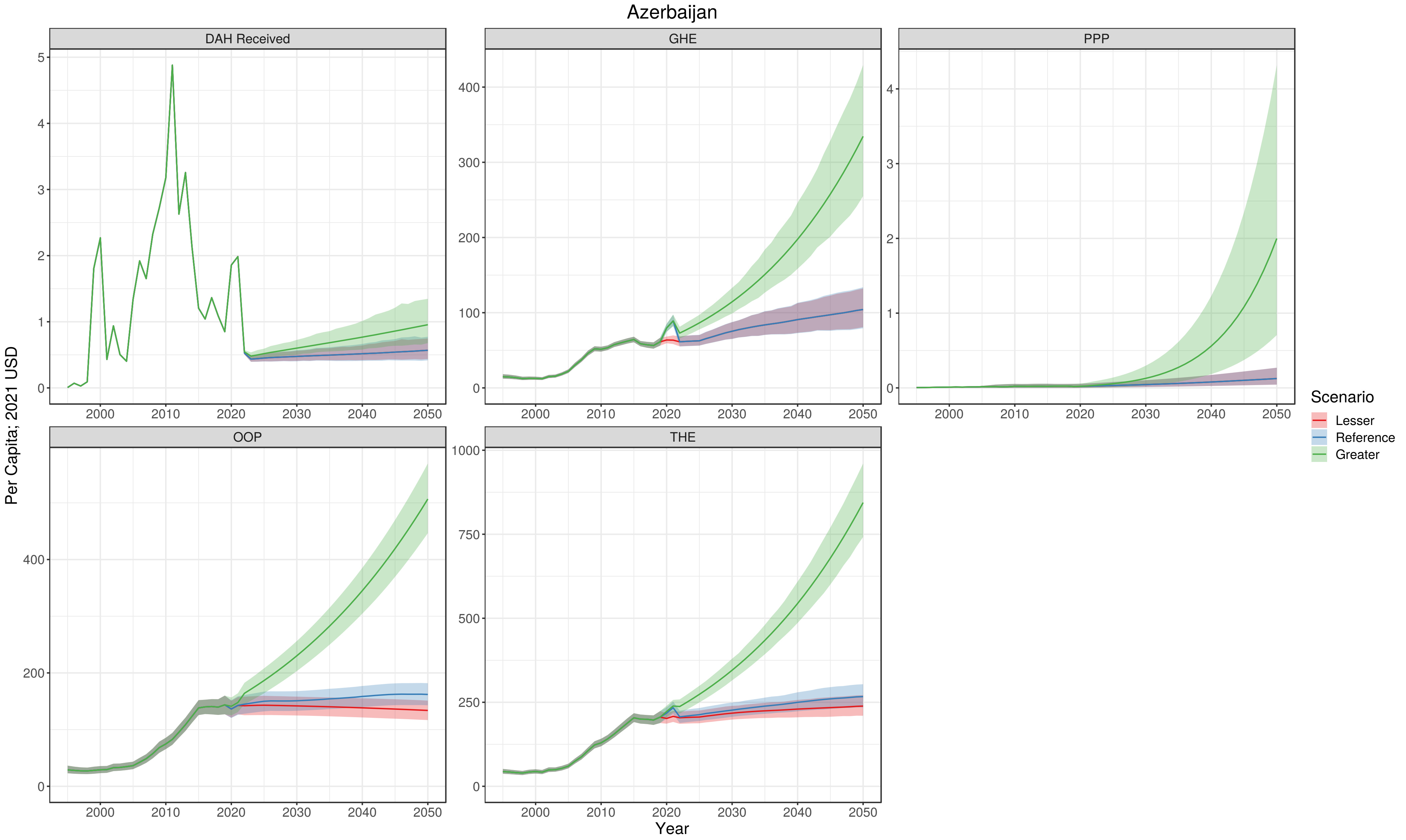

Azerbaijan
DAH Received
GHE
PPP
OOP
THE
Per Capita; 2021 USD
Year
Scenario
Lesser
Reference
Greater

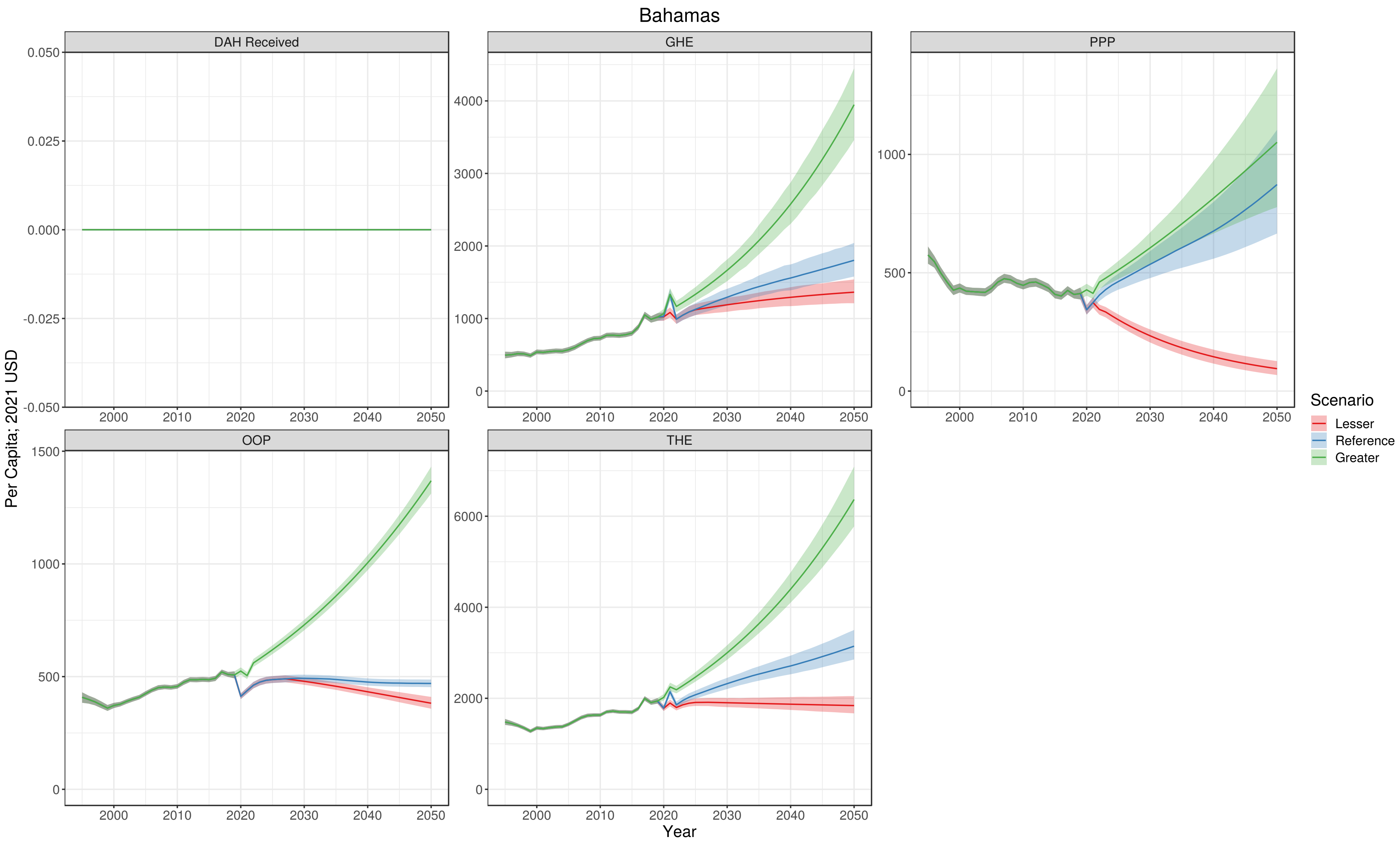

Bahamas
DAH Received
GHE
PPP
OOP
THE
Per Capita; 2021 USD
Year
Scenario
Lesser
Reference
Greater
0.050
0.025
0.000
-0.025
-0.050
4000
3000
2000
1000
0
1000
500
1500
6000
2000
2010
2020
2030
2040
2050

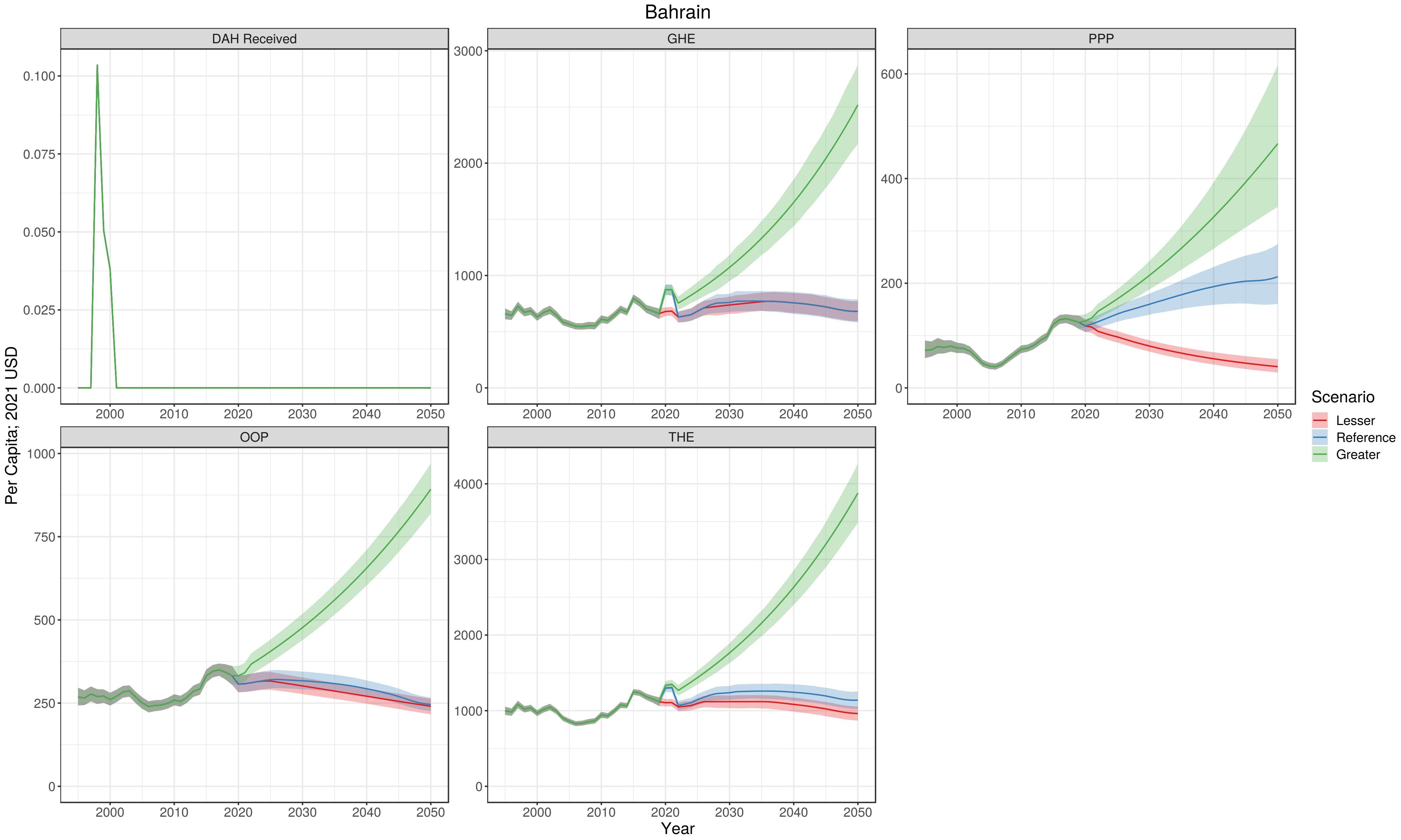
Bahrain
DAH Received
GHE
PPP
OOP
THE
Per Capita; 2021 USD
Year
Scenario
Lesser
Reference
Greater
0.000
0.025
0.050
0.075
0.100
0
1000
2000
3000
200
400
600
250
500
750
4000
2000
2010
2020
2030
2040
2050

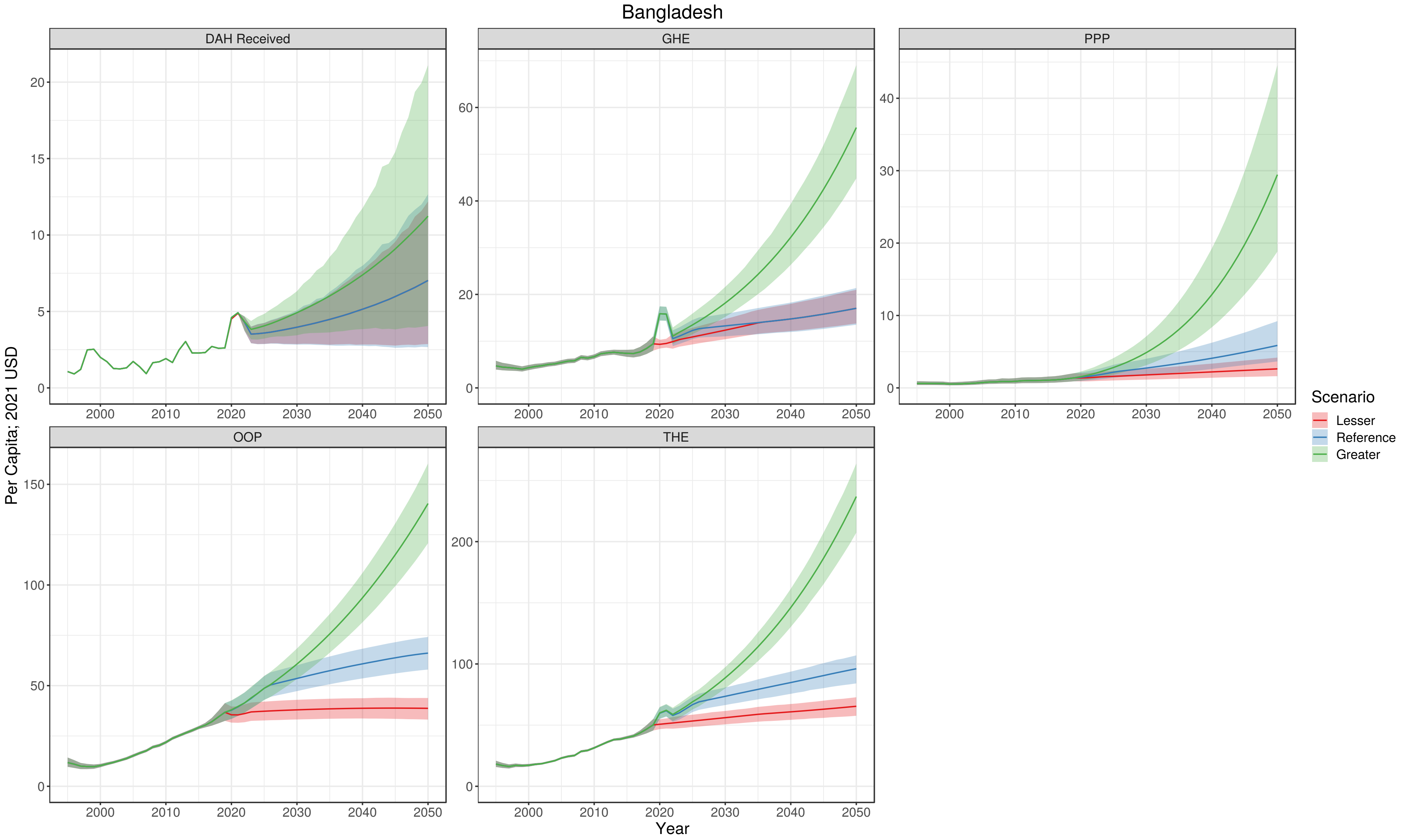

Bangladesh
DAH Received
GHE
PPP
OOP
THE
Per Capita; 2021 USD
Year
Scenario
Lesser
Reference
Greater
0
5
10
15
20
20
40
60
30
40
50
100
150
200
2000
2010
2020
2030
2040
2050

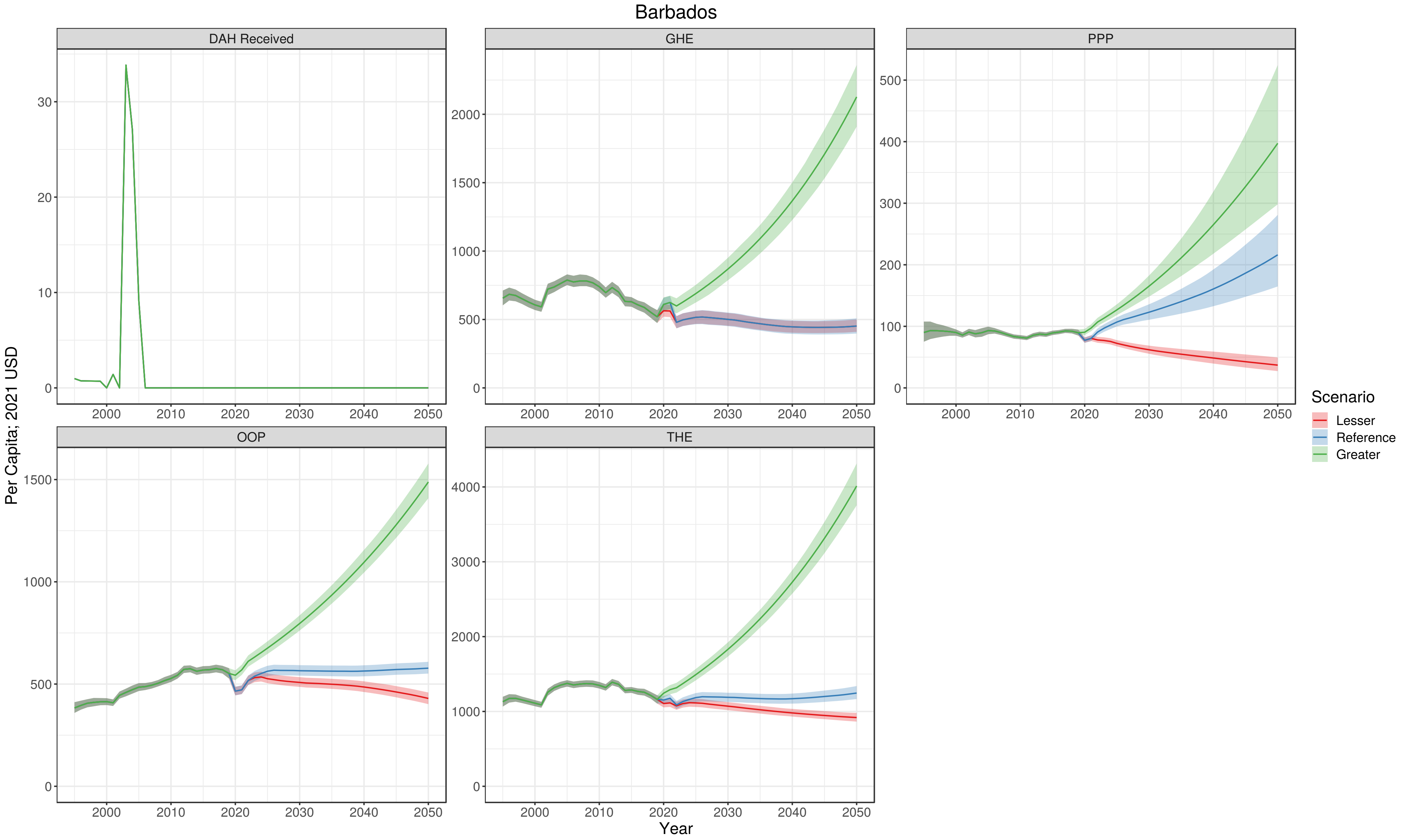

Barbados
DAH Received
GHE
PPP
OOP
THE
Per Capita; 2021 USD
Year
Scenario
Lesser
Reference
Greater
30
20
10
0
2000
1500
1000
500
500
400
300
200
100
2000
2010
2020
2030
2040
2050
4000
3000

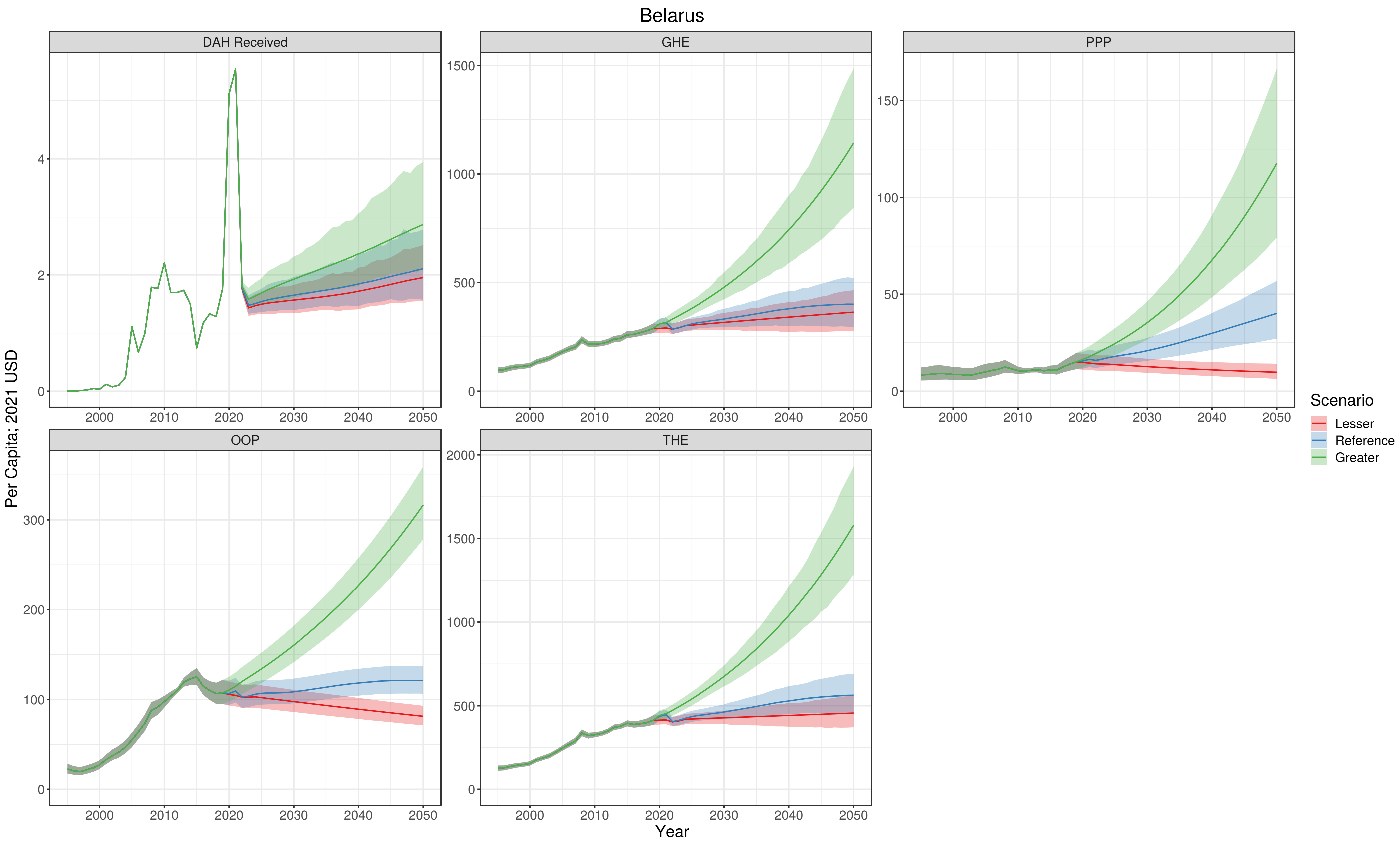

Belarus
DAH Received
GHE
PPP
OOP
THE
Per Capita; 2021 USD
Year
Scenario
Lesser
Reference
Greater
0
2
4
0
500
1000
1500
0
50
100
150
0
100
200
300
0
500
1000
1500
2000
2000
2010
2020
2030
2040
2050

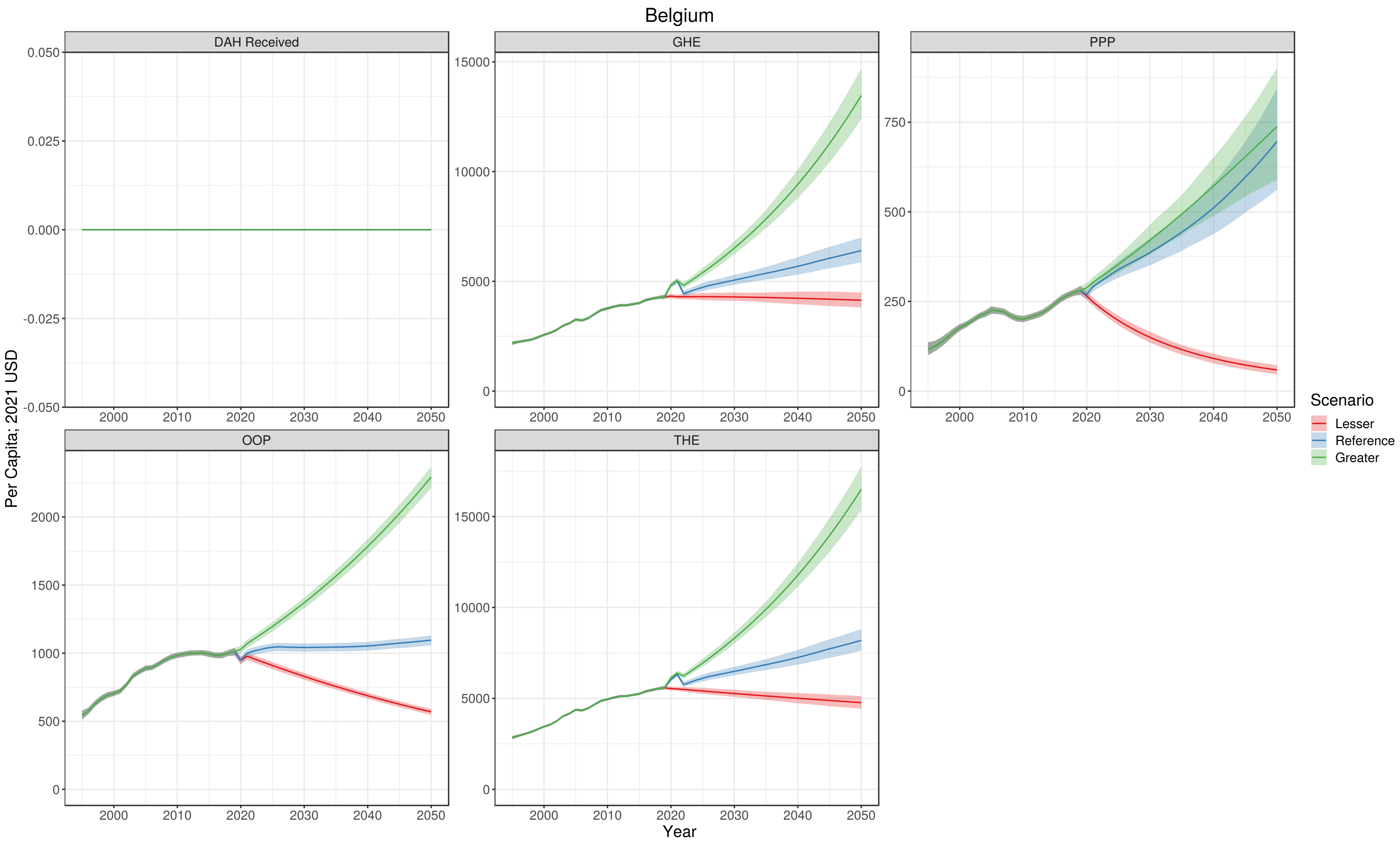

Belgium
DAH Received
GHE
PPP
OOP
THE
0.050
0.025
0.000
-0.025
-0.050
15000
10000
5000
0
750
500
250
2000
1500
1000
500
2000
2010
2020
2030
2040
2050
Per Capita; 2021 USD
Year
Scenario
Lesser
Reference
Greater

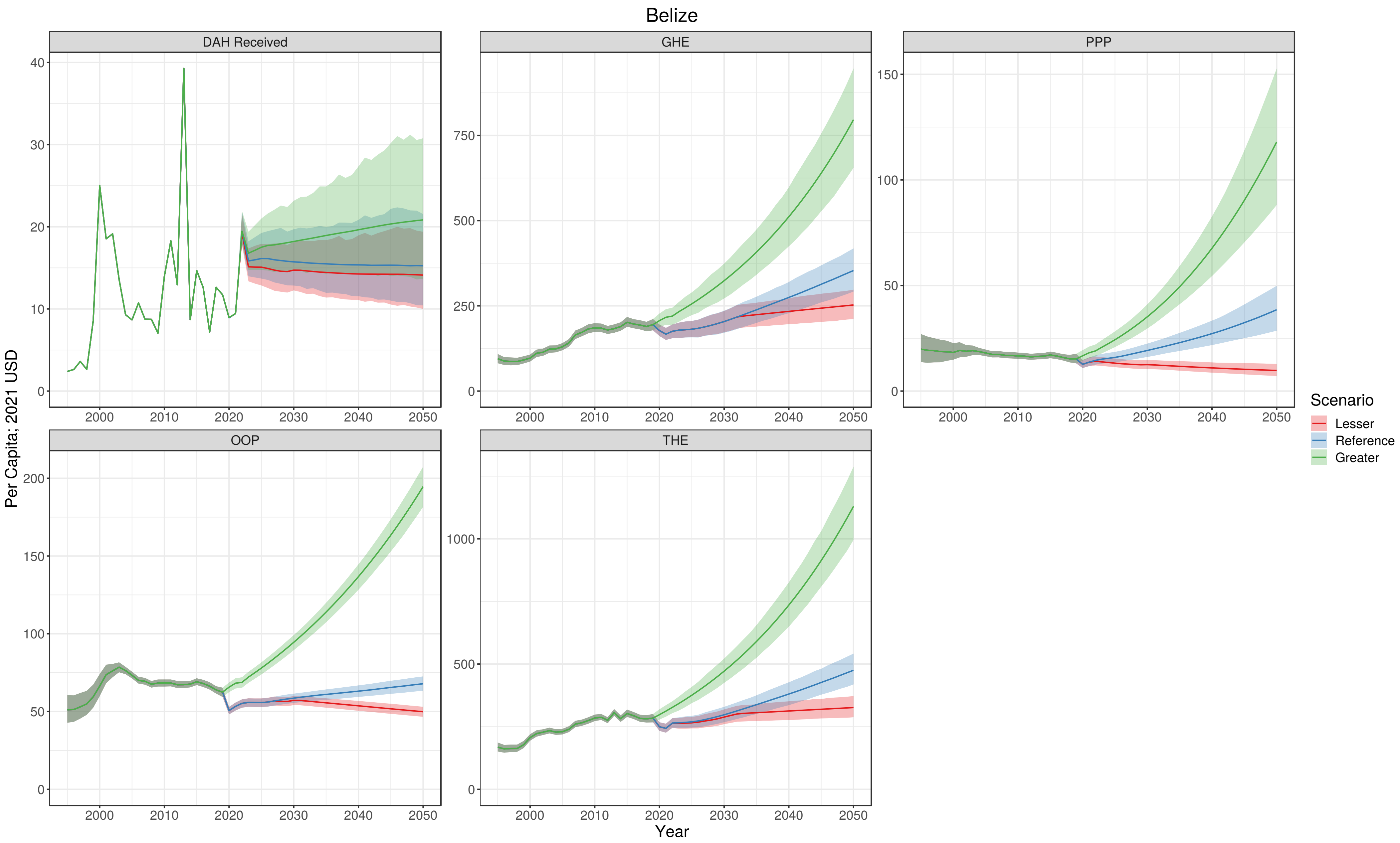

Belize
DAH Received
GHE
PPP
OOP
THE
Per Capita; 2021 USD
Year
Scenario
Lesser
Reference
Greater
0
10
20
30
40
250
500
750
50
100
150
200
1000
2000
2010
2020
2030
2040
2050

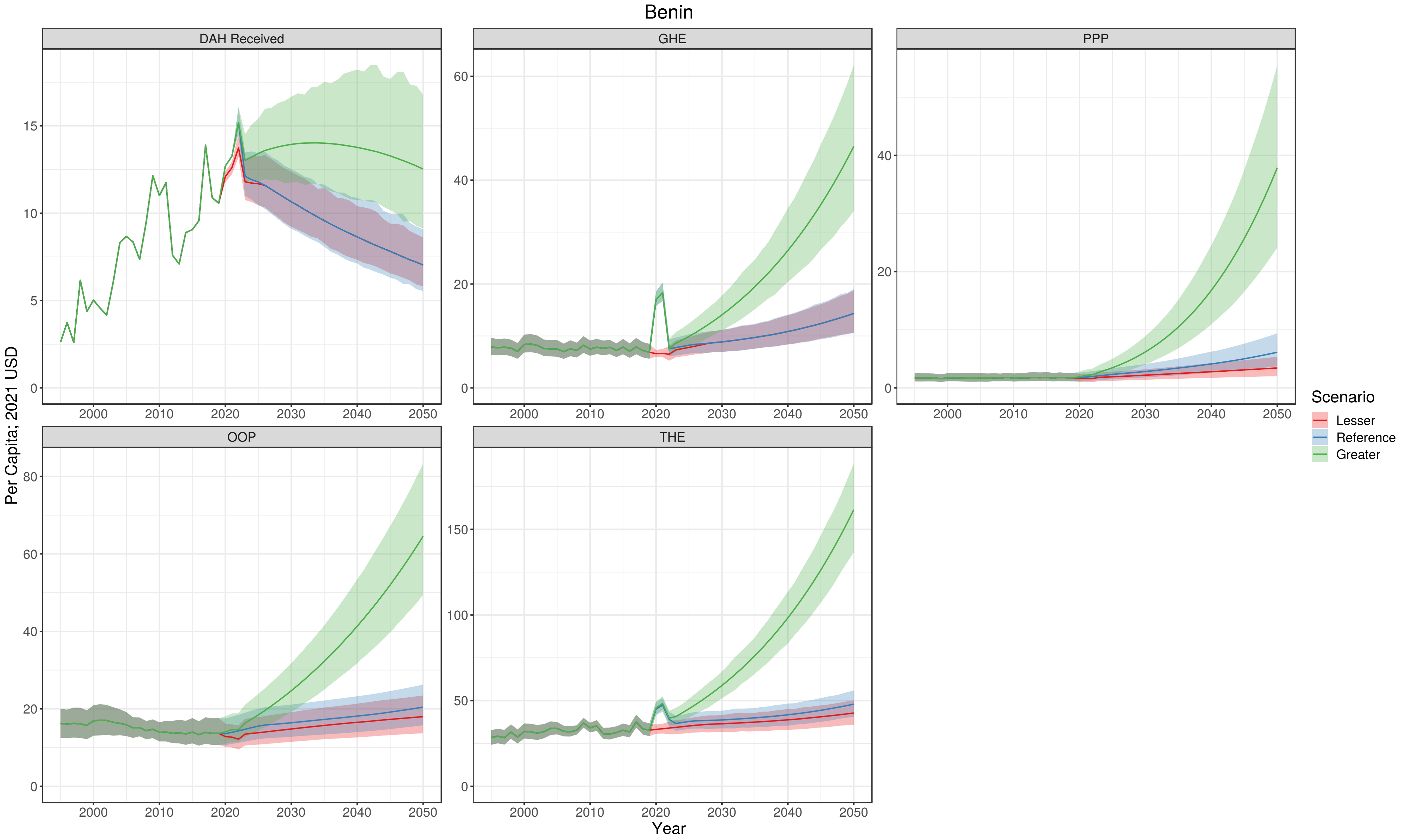
Benin
DAH Received
GHE
PPP
OOP
THE
Per Capita; 2021 USD
Year
Scenario
Lesser
Reference
Greater
2000
2010
2020
2030
2040
2050

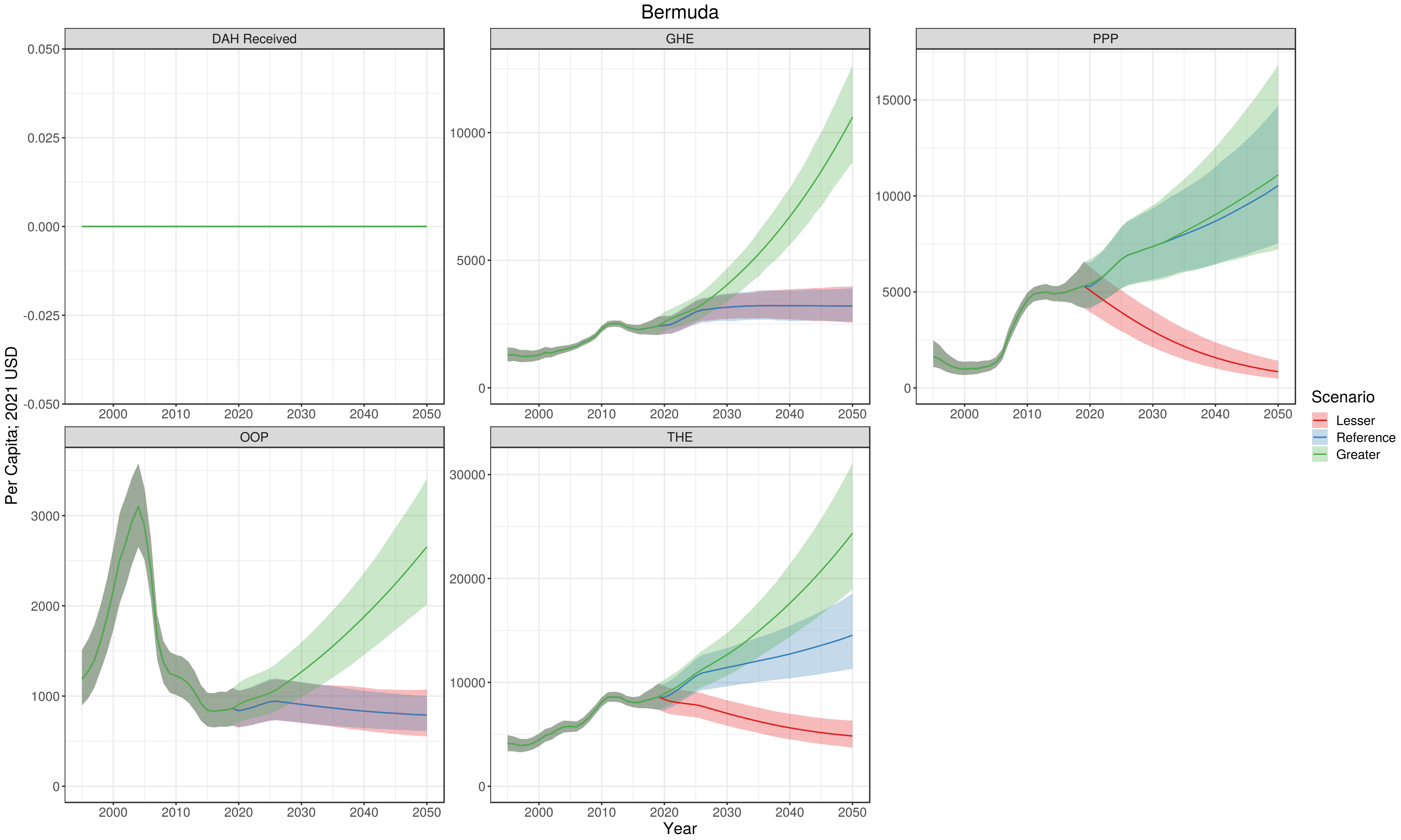

Bermuda
DAH Received
GHE
PPP
OOP
THE
Per Capita; 2021 USD
Year
Scenario
Lesser
Reference
Greater
0.050
0.025
0.000
-0.025
-0.050
10000
5000
0
15000
3000
2000
1000
30000
20000
2000
2010
2020
2030
2040
2050

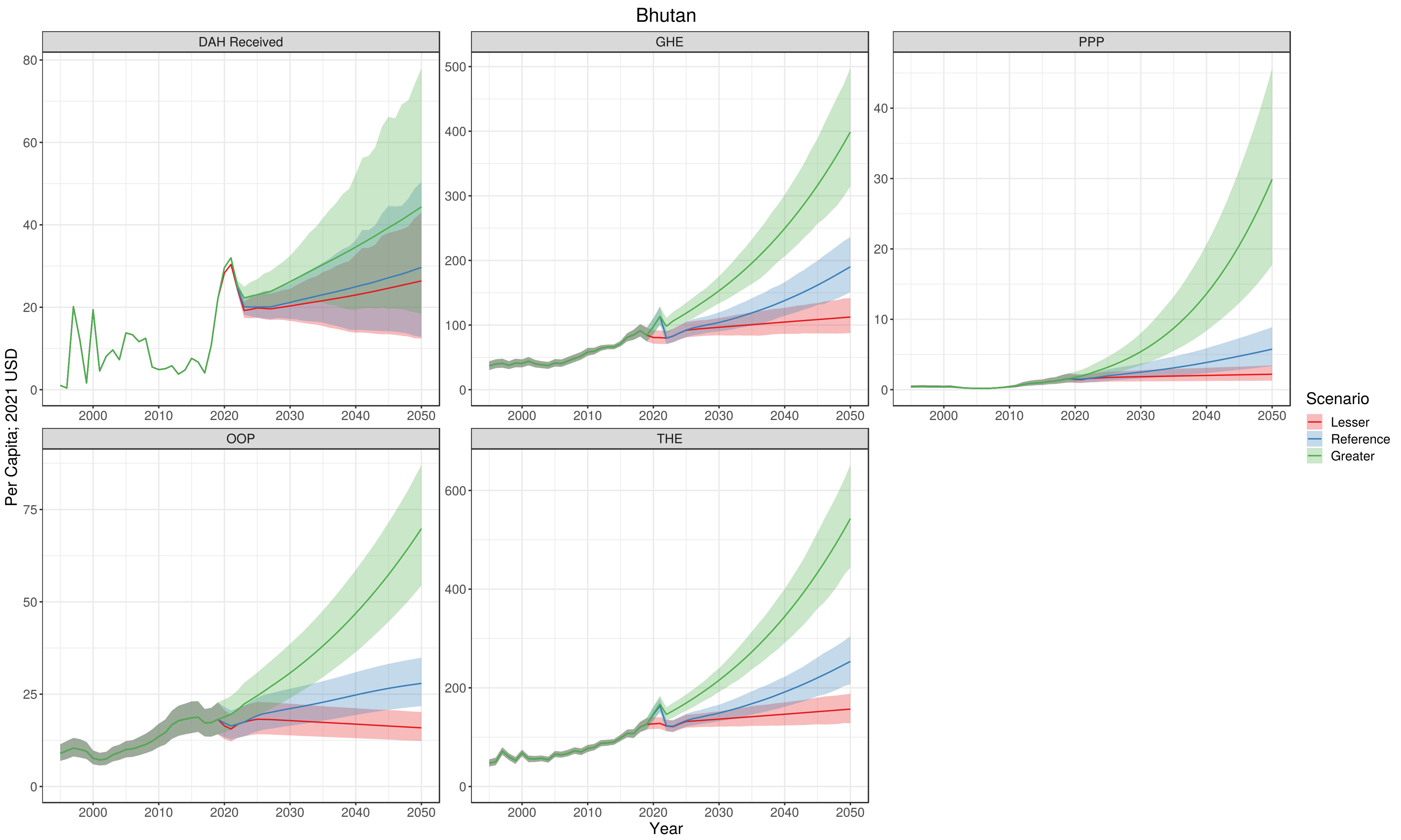

Bhutan
DAH Received
GHE
PPP
OOP
THE
Per Capita; 2021 USD
Year
Scenario
Lesser
Reference
Greater

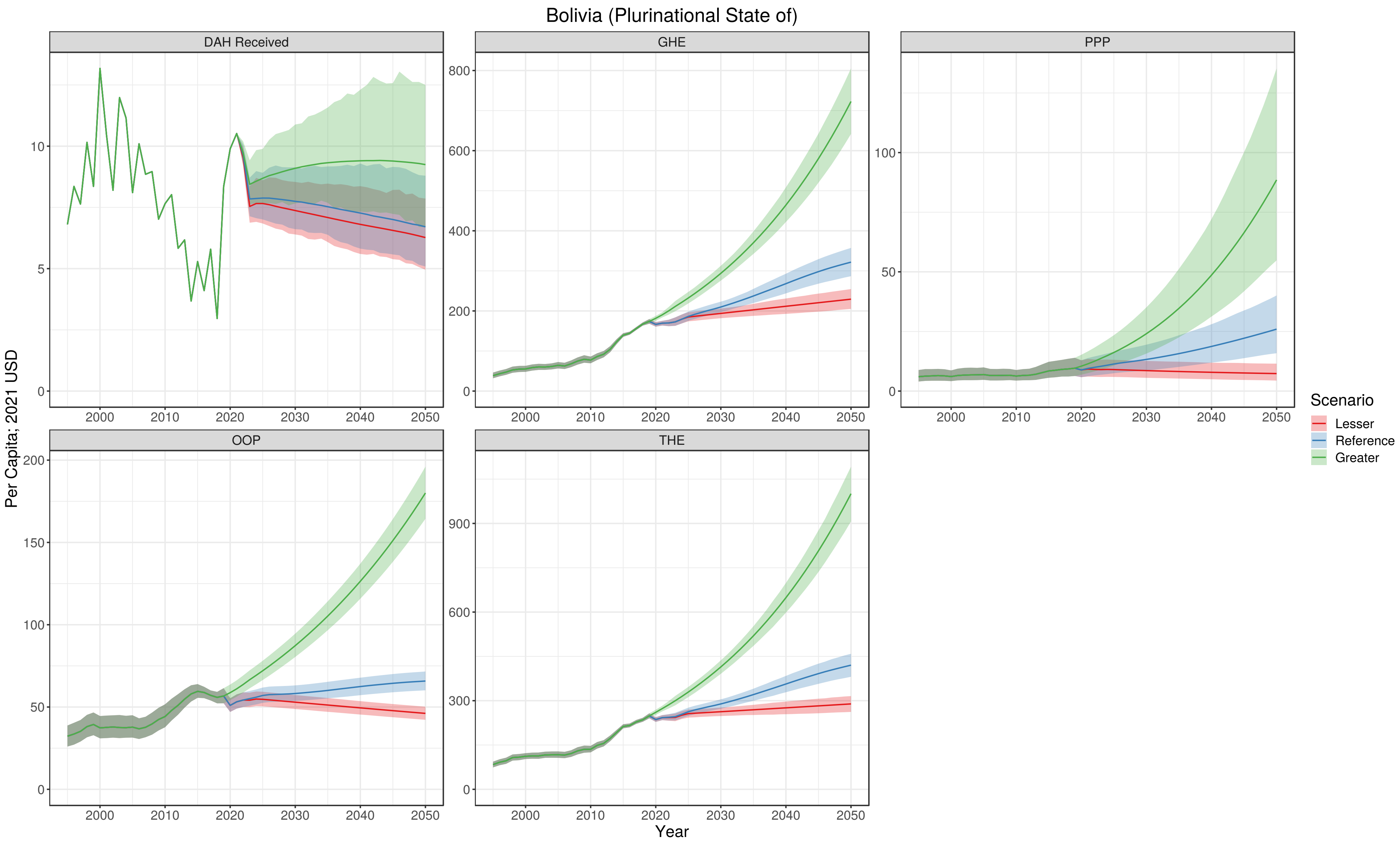

Bolivia (Plurinational State of)
DAH Received
GHE
PPP
OOP
THE
Per Capita; 2021 USD
Year
Scenario
Lesser
Reference
Greater

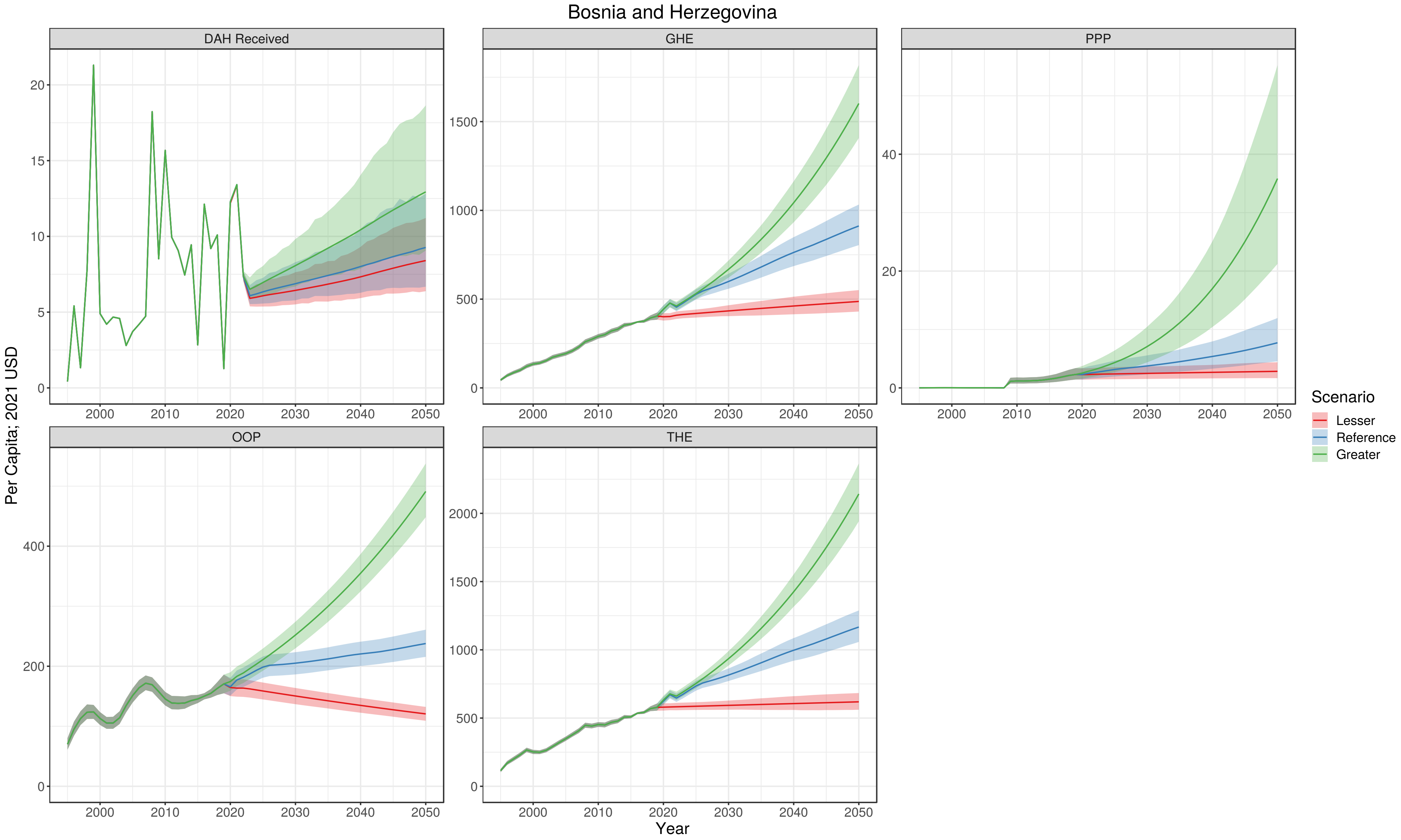

Bosnia and Herzegovina
DAH Received
GHE
PPP
OOP
THE
Per Capita; 2021 USD
Year
Scenario
Lesser
Reference
Greater

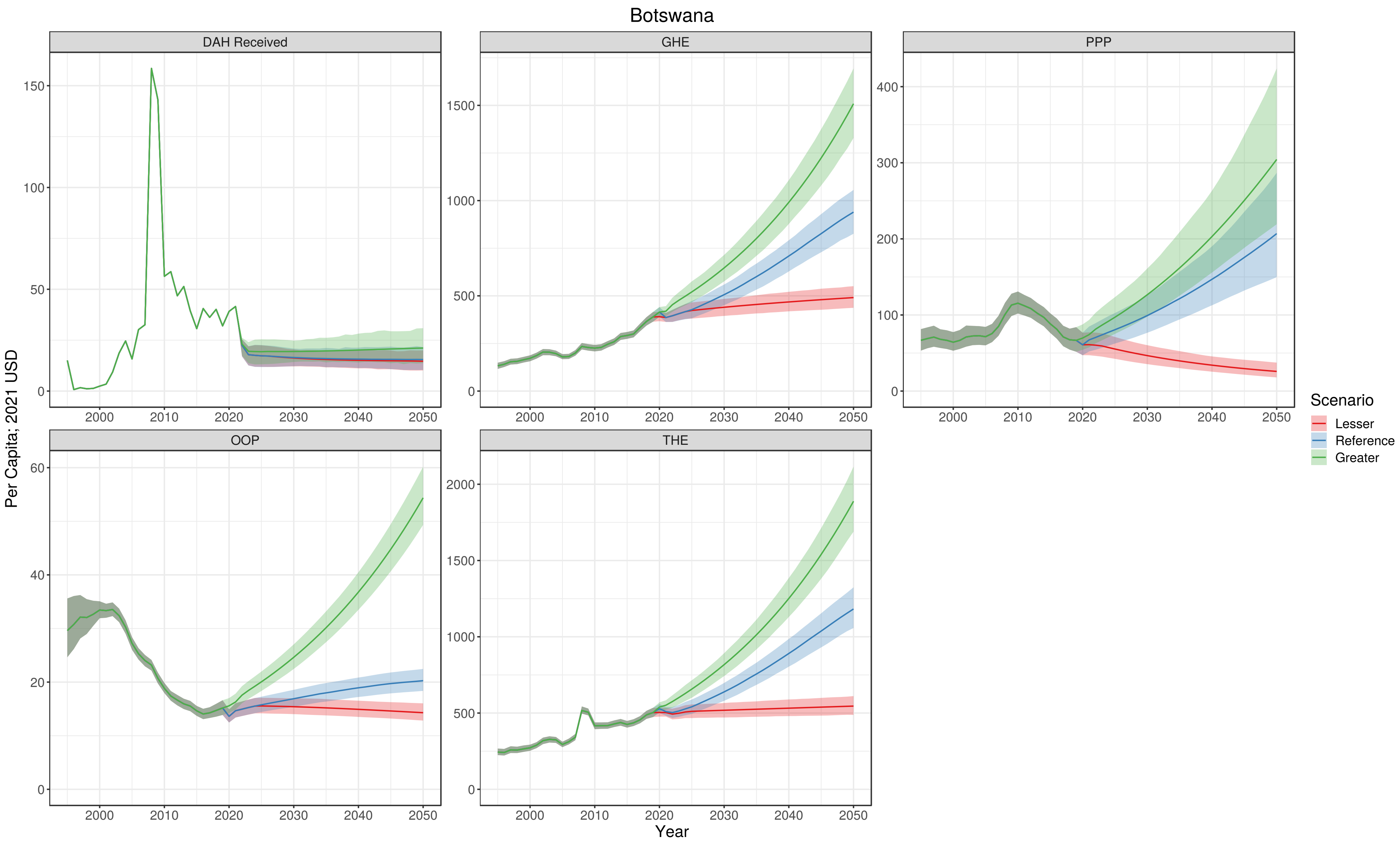

Botswana
DAH Received
GHE
PPP
OOP
THE
Per Capita; 2021 USD
Year
Scenario
Lesser
Reference
Greater

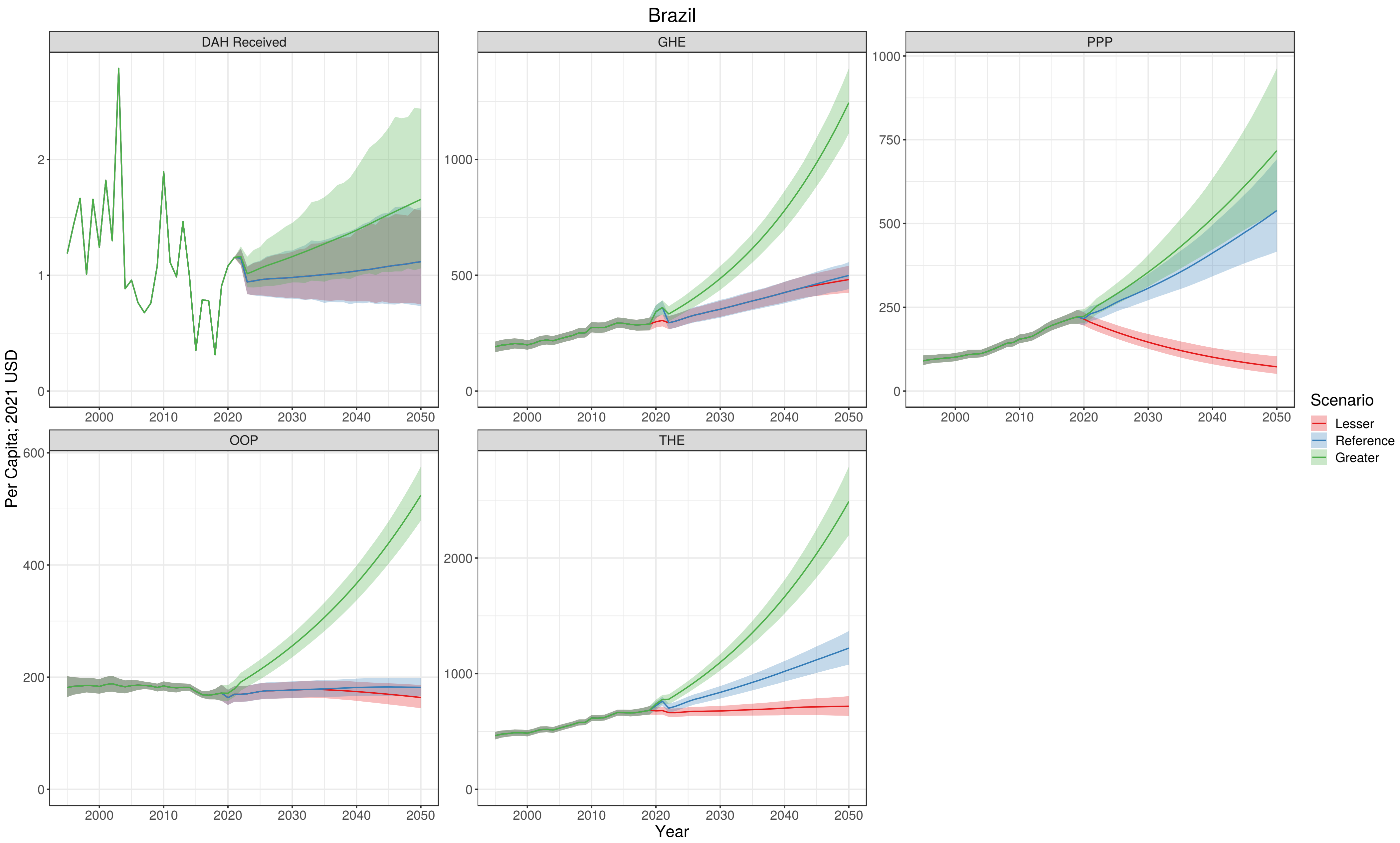

Brazil
DAH Received
GHE
PPP
OOP
THE
Per Capita; 2021 USD
Year
Scenario
Lesser
Reference
Greater

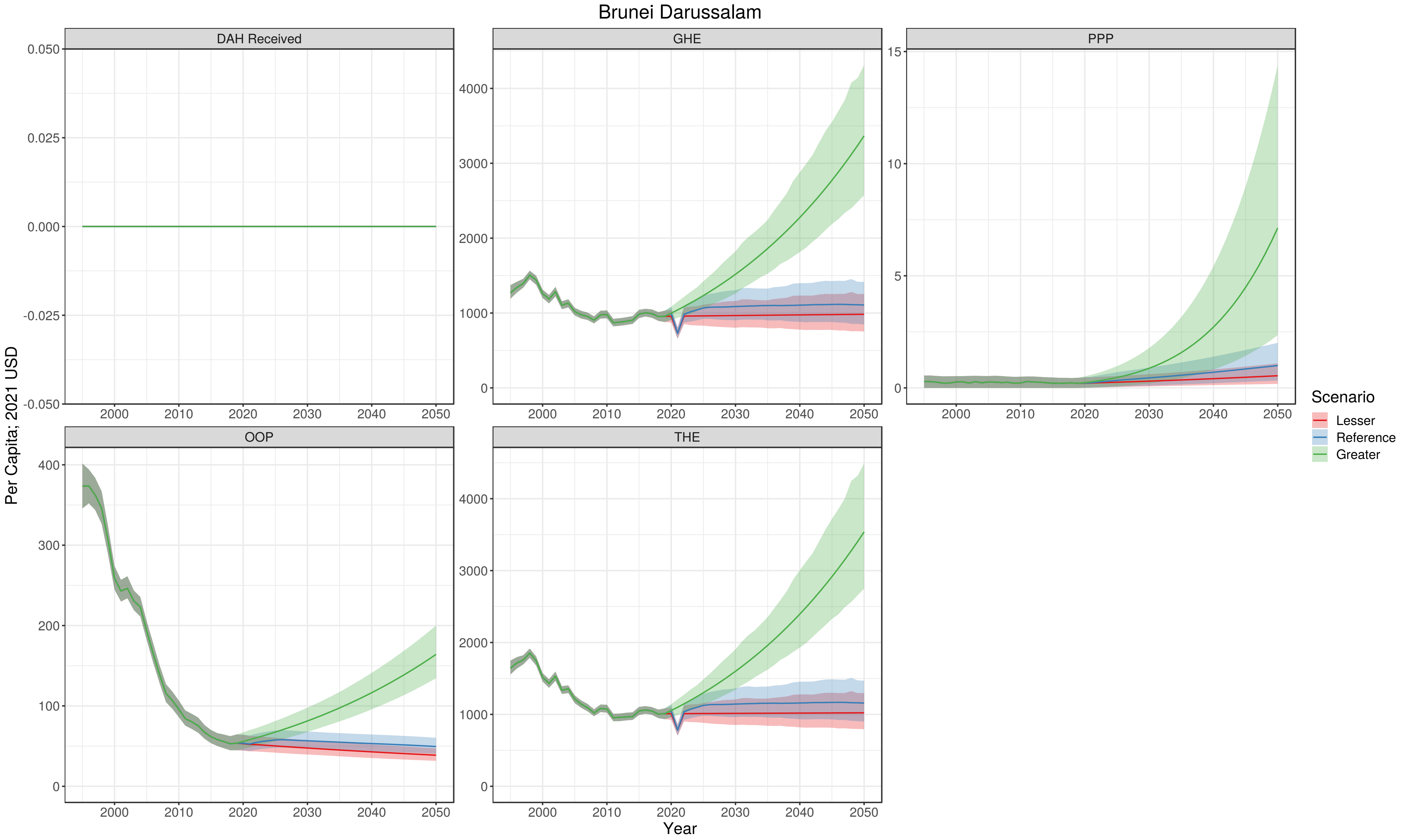
Brunei Darussalam
DAH Received
GHE
PPP
OOP
THE
Per Capita; 2021 USD
Year
Scenario
Lesser
Reference
Greater
0.050
0.025
0.000
-0.025
-0.050
4000
3000
2000
1000
0
15
10
5
400
300
200
100
2000
2010
2020
2030
2040
2050

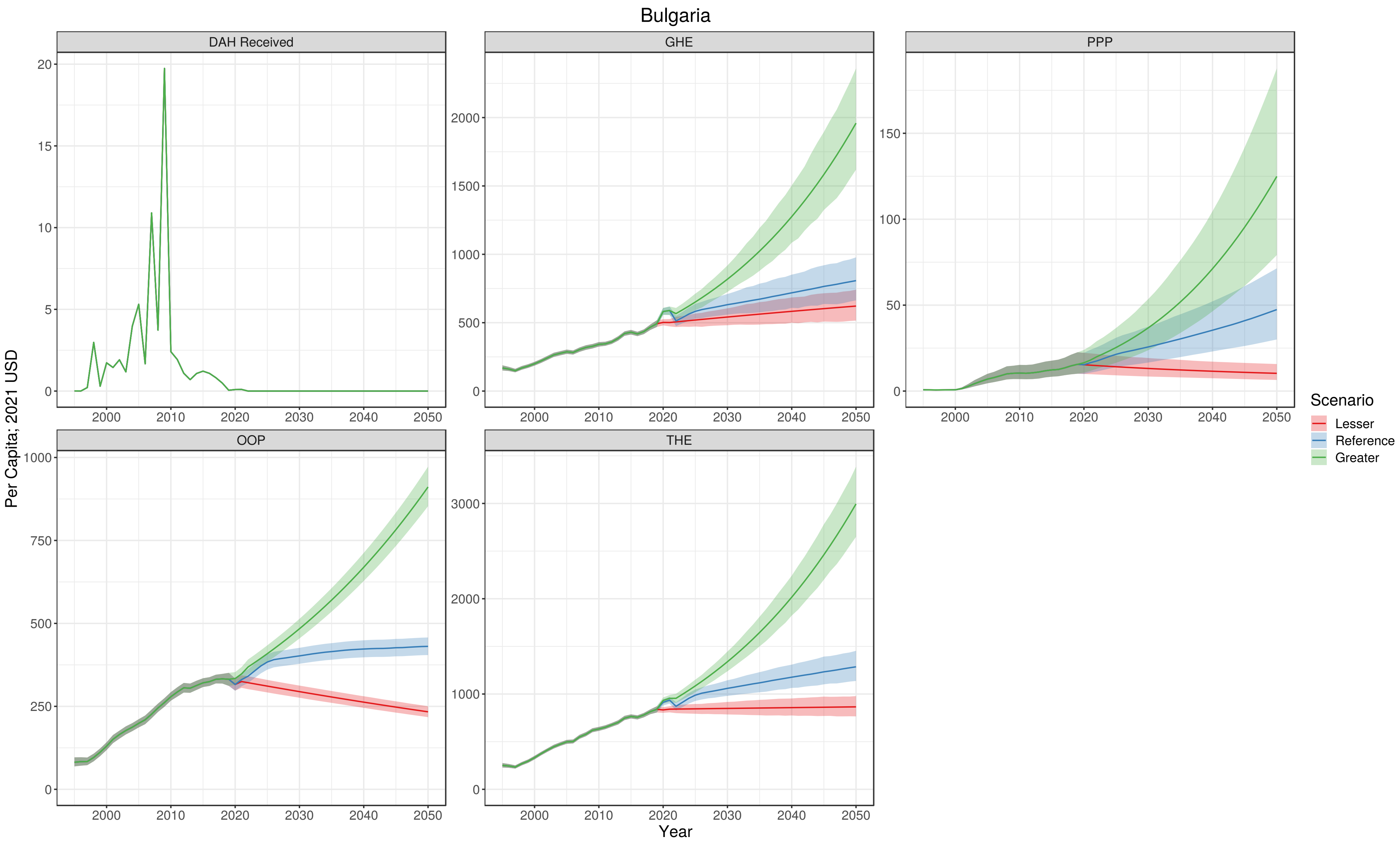

Bulgaria
DAH Received
GHE
PPP
OOP
THE
Per Capita; 2021 USD
Year
Scenario
Lesser
Reference
Greater
20
15
10
5
0
2000
2010
2020
2030
2040
2050
1500
1000
500
150
100
50
750
250
3000

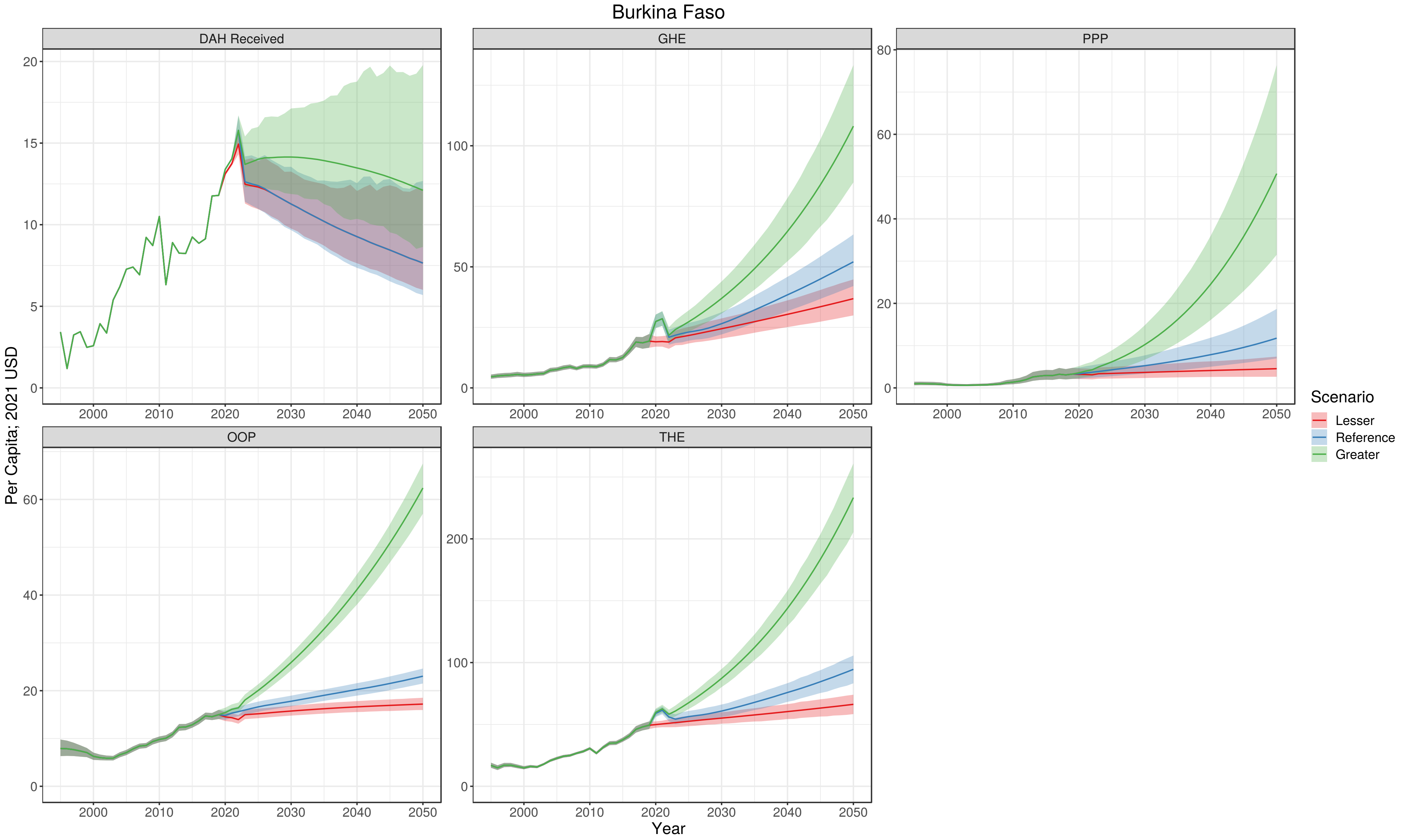

Burkina Faso
DAH Received
GHE
PPP
OOP
THE
Per Capita; 2021 USD
Year
0
5
10
15
20
50
100
40
60
80
200
2000
2010
2020
2030
2040
2050
Scenario
Lesser
Reference
Greater

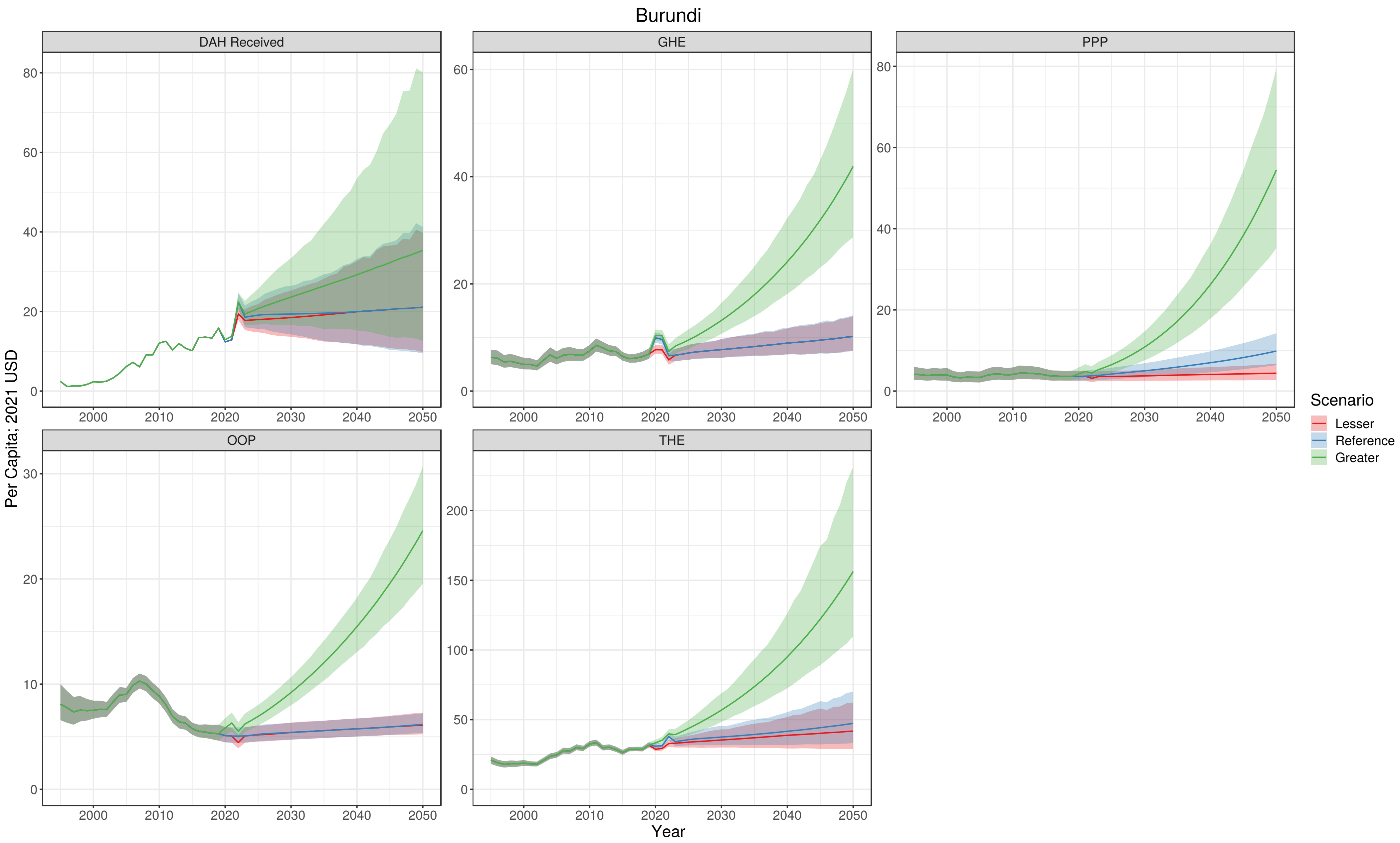
Burundi
DAH Received
GHE
PPP
OOP
THE
Per Capita; 2021 USD
Year
Scenario
Lesser
Reference
Greater

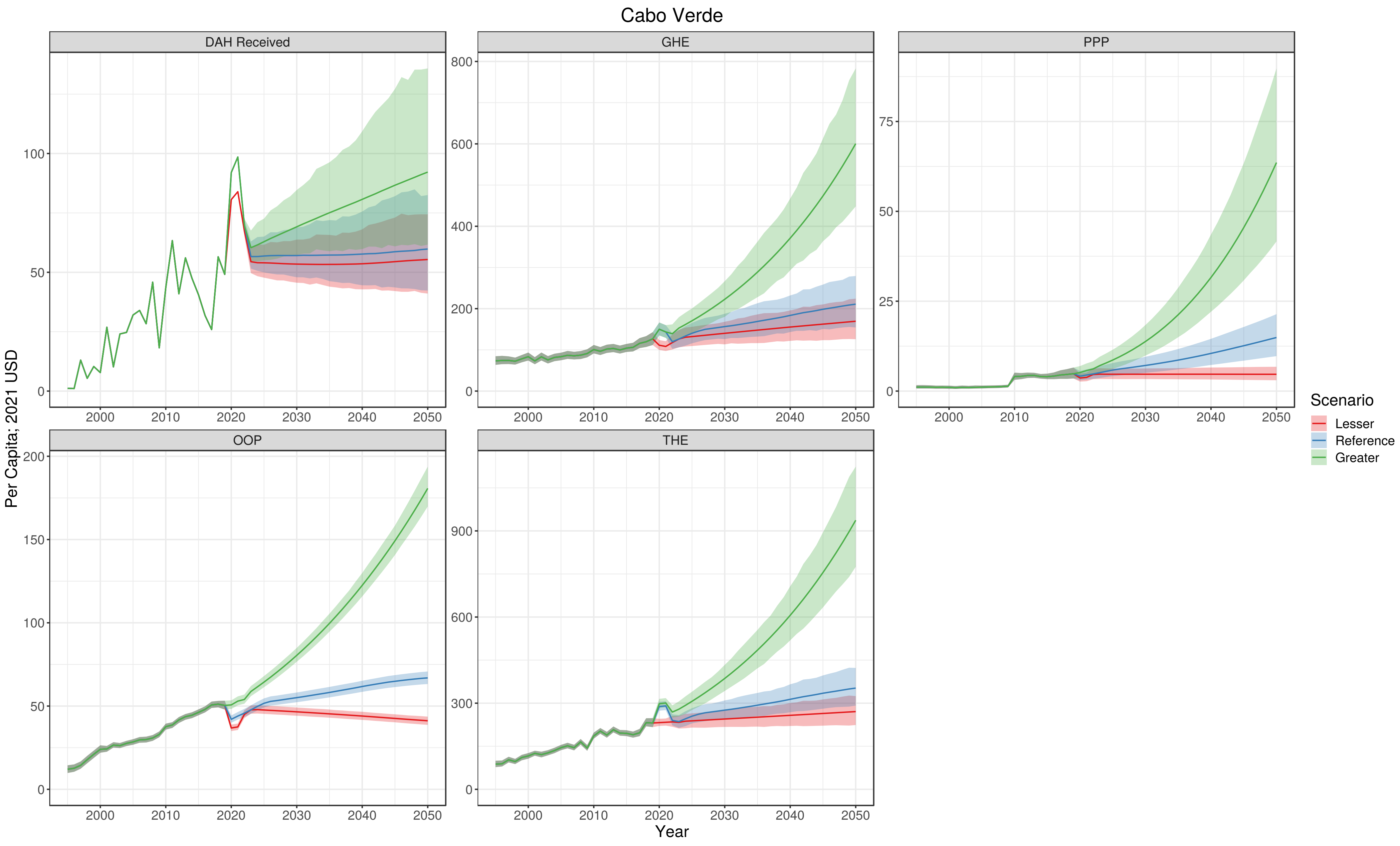

Cabo Verde
DAH Received
GHE
PPP
OOP
THE
Per Capita; 2021 USD
Year
Scenario
Lesser
Reference
Greater

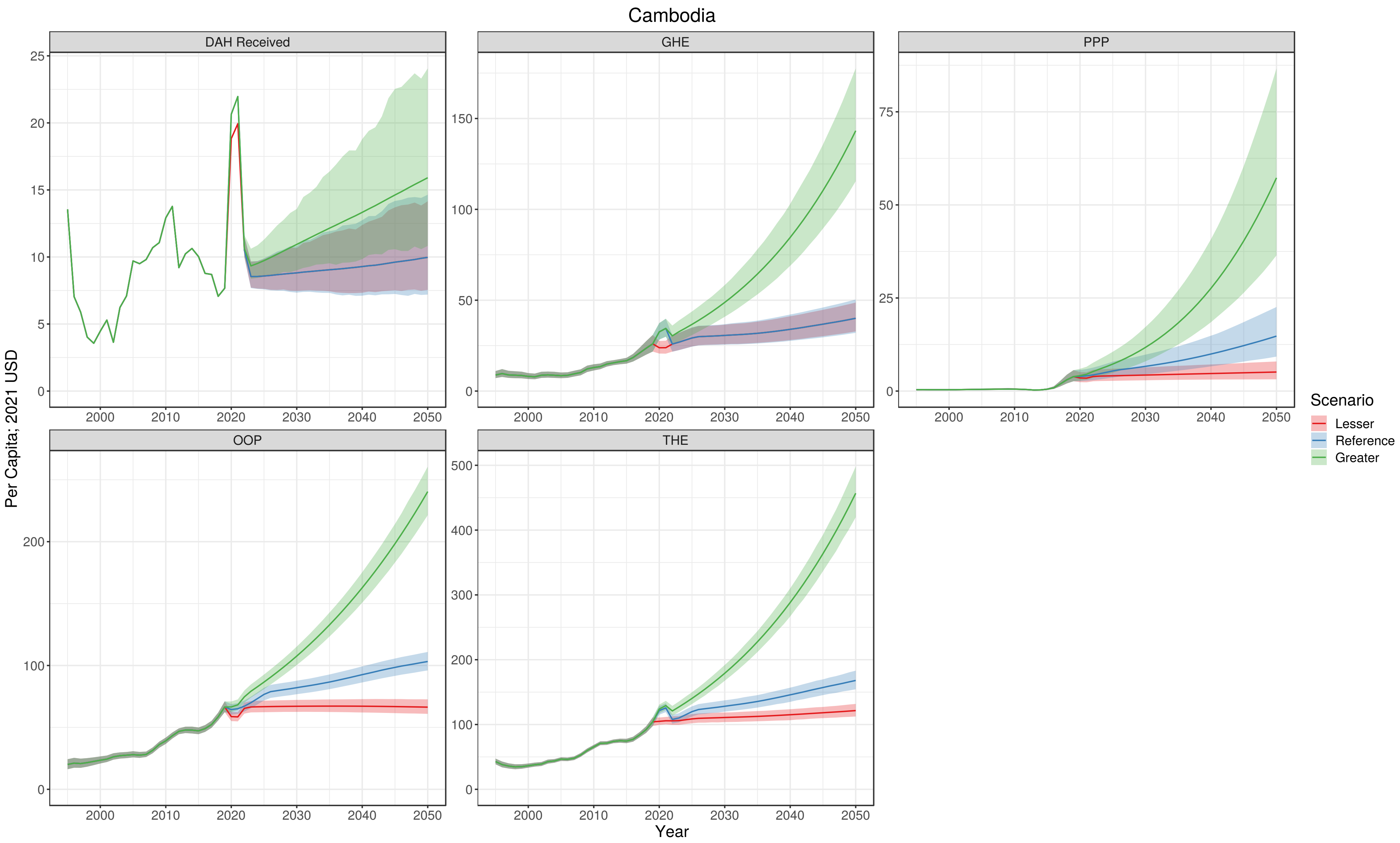

Cambodia
DAH Received
GHE
PPP
OOP
THE
Per Capita; 2021 USD
Year
Scenario
Lesser
Reference
Greater

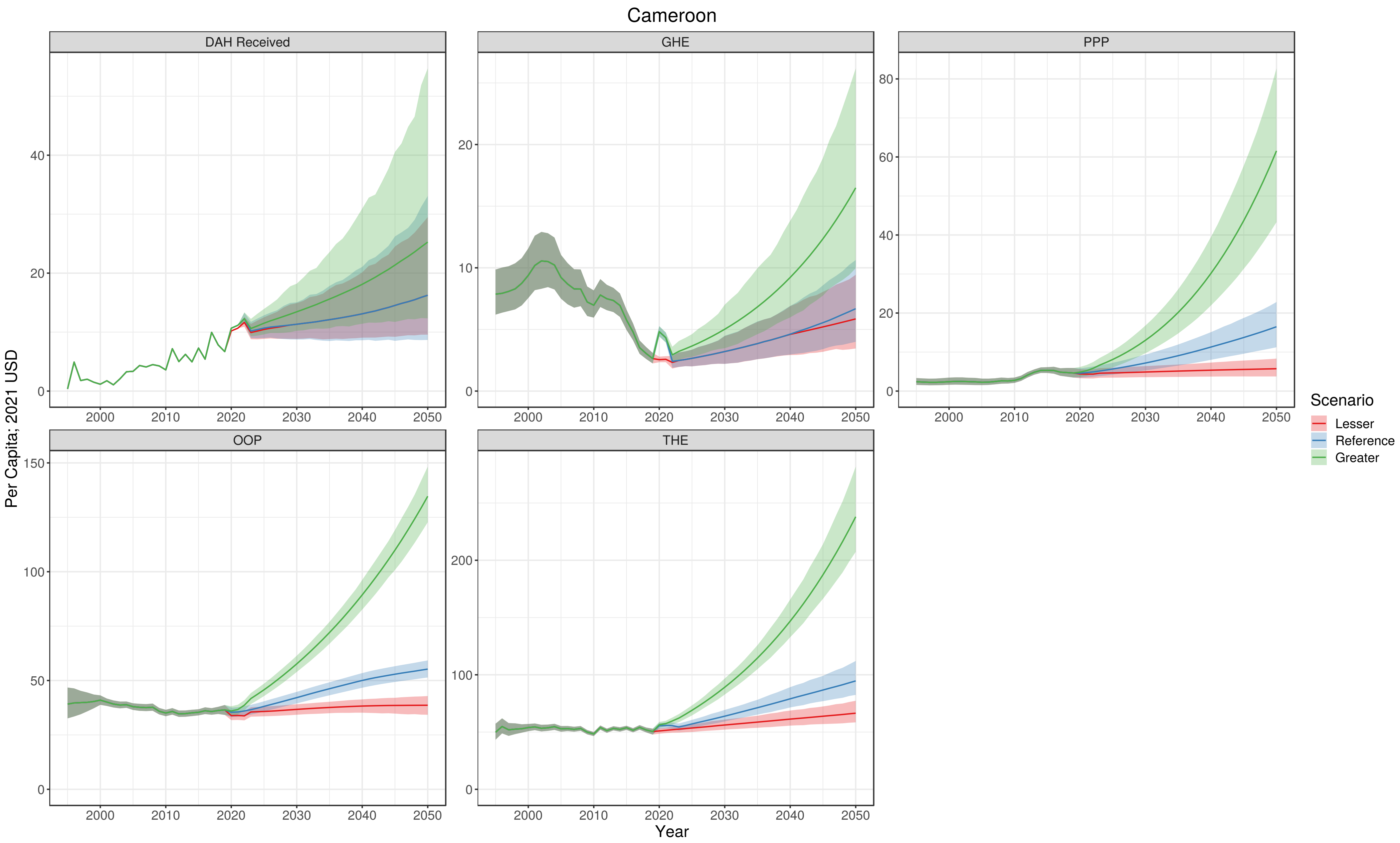
Cameroon
DAH Received
GHE
PPP
OOP
THE
Per Capita; 2021 USD
Year
Scenario
Lesser
Reference
Greater

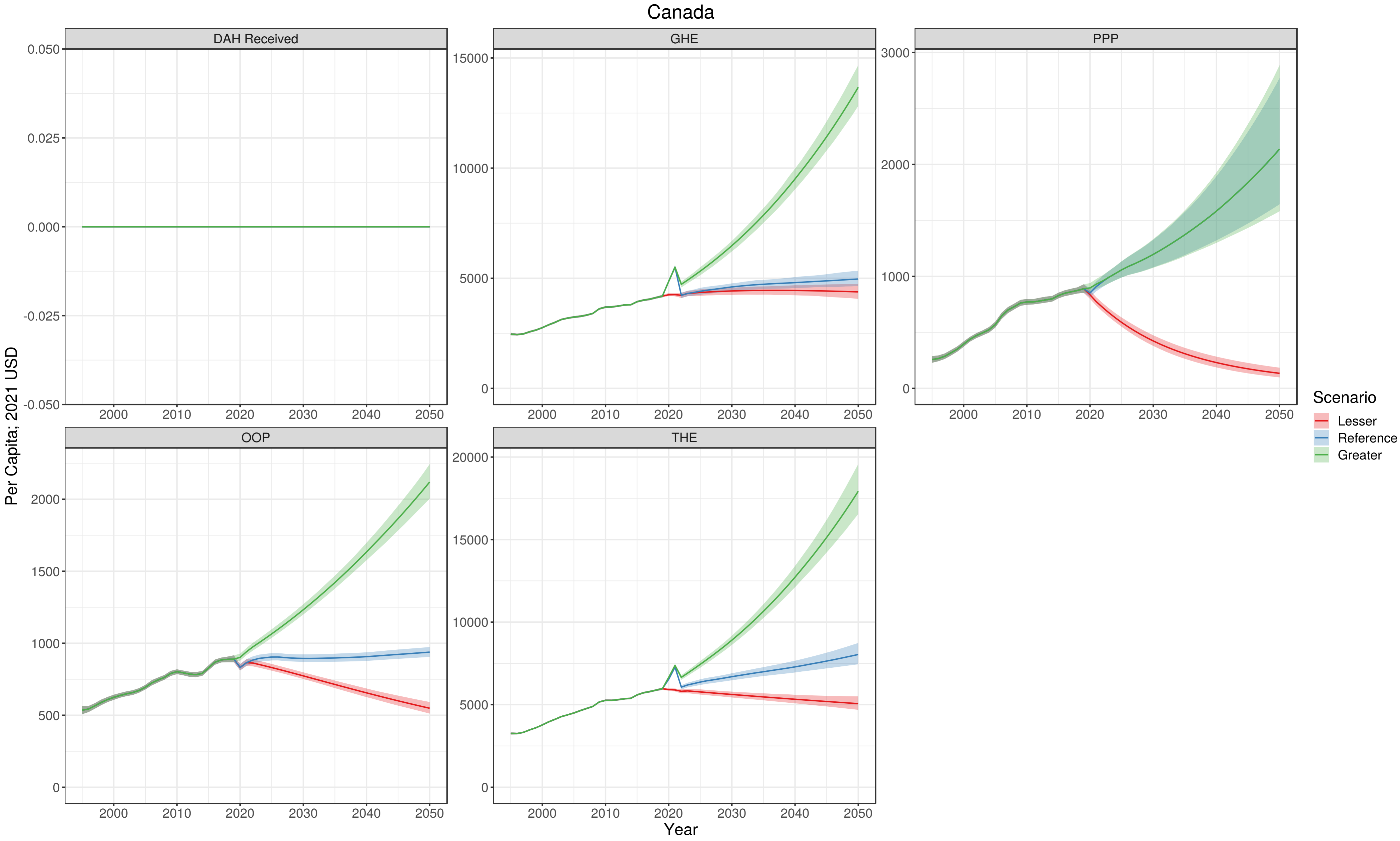

Canada
DAH Received
GHE
PPP
OOP
THE
Per Capita; 2021 USD
Year
Scenario
Lesser
Reference
Greater
0.050
0.025
0.000
-0.025
-0.050
15000
10000
5000
0
3000
2000
1000
2000
1500
1000
500
20000
2000
2010
2020
2030
2040
2050

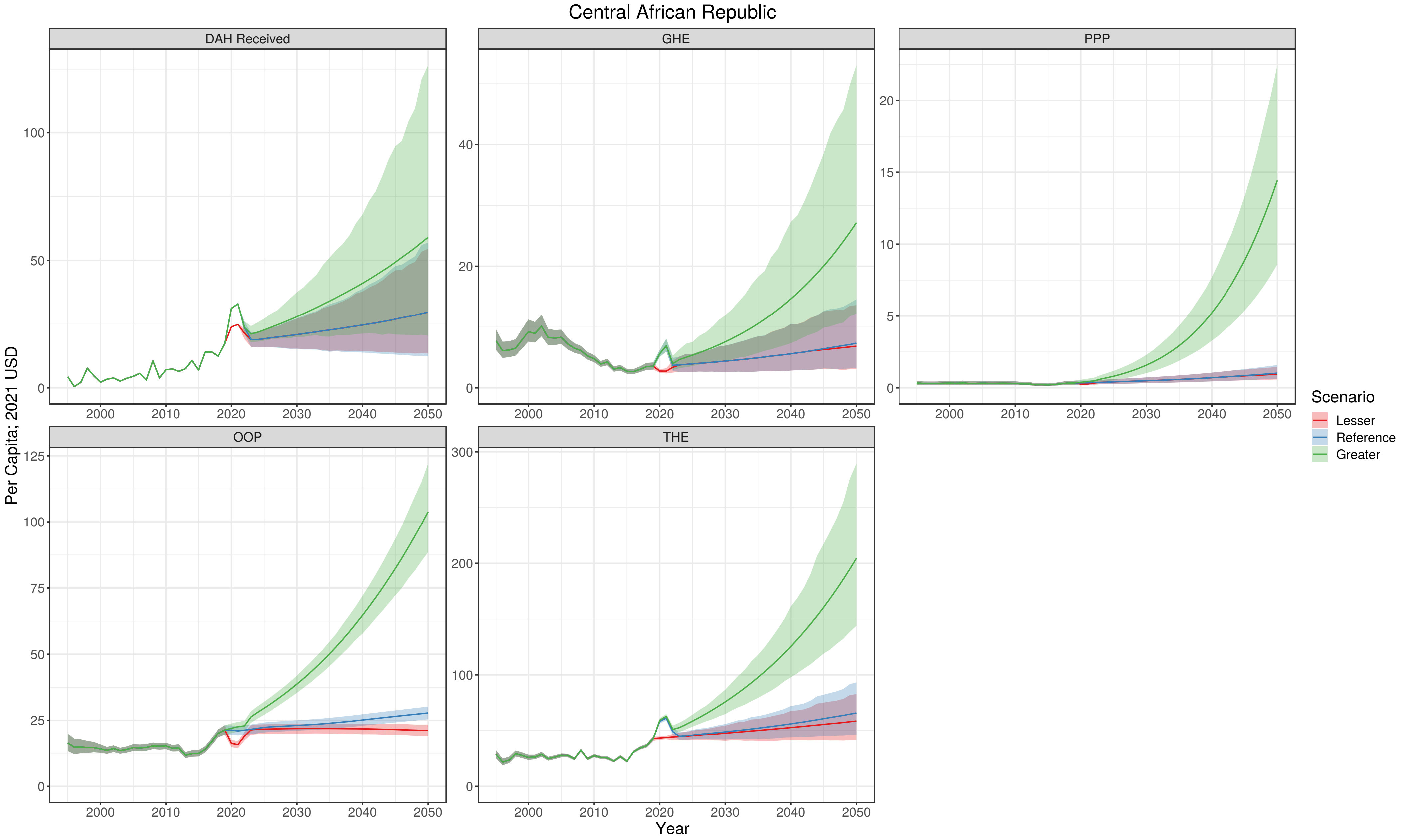

Central African Republic
DAH Received
GHE
PPP
OOP
THE
Per Capita; 2021 USD
Year
Scenario
Lesser
Reference
Greater

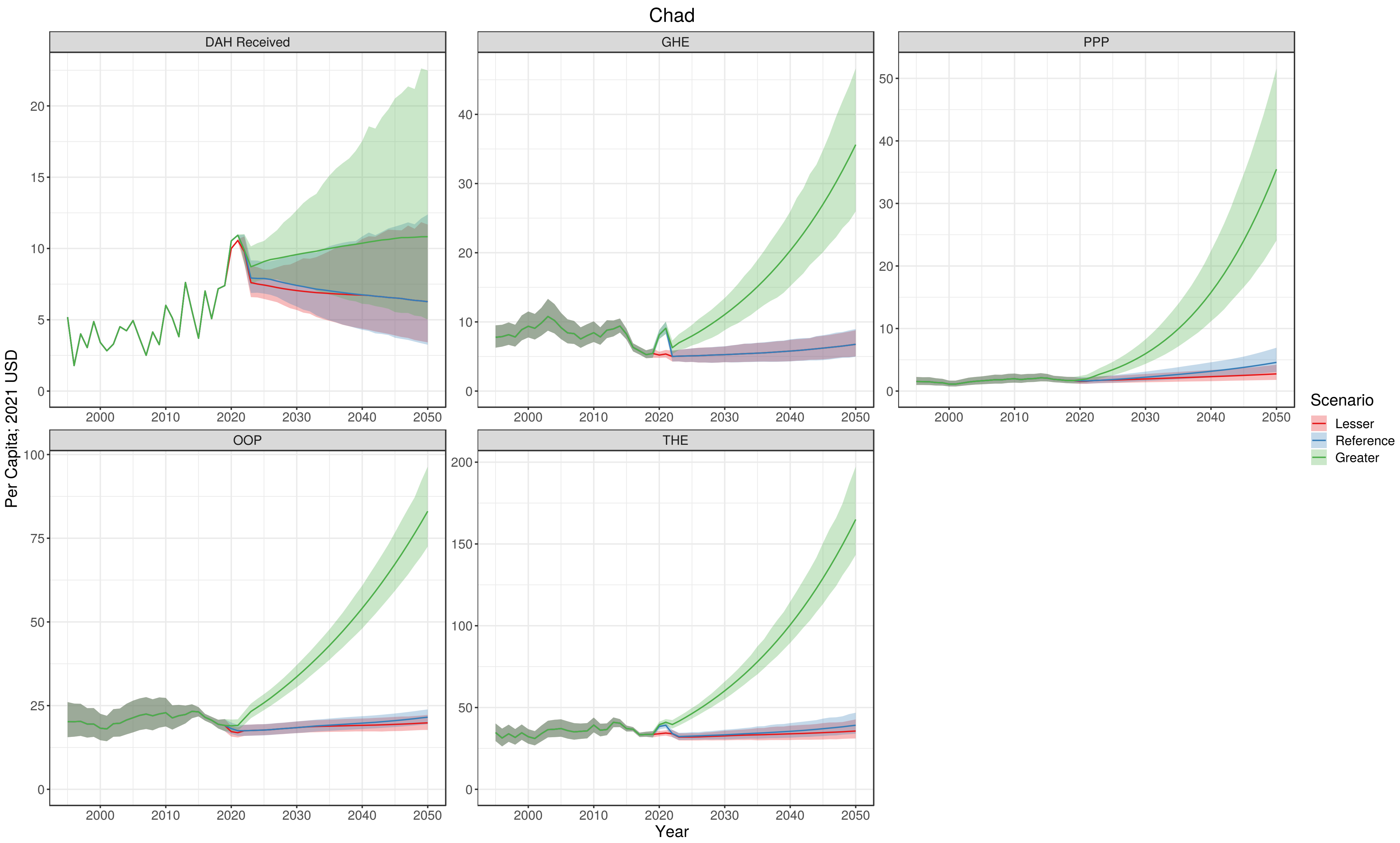

Chad
DAH Received
GHE
PPP
OOP
THE
Per Capita; 2021 USD
Year
Scenario
Lesser
Reference
Greater

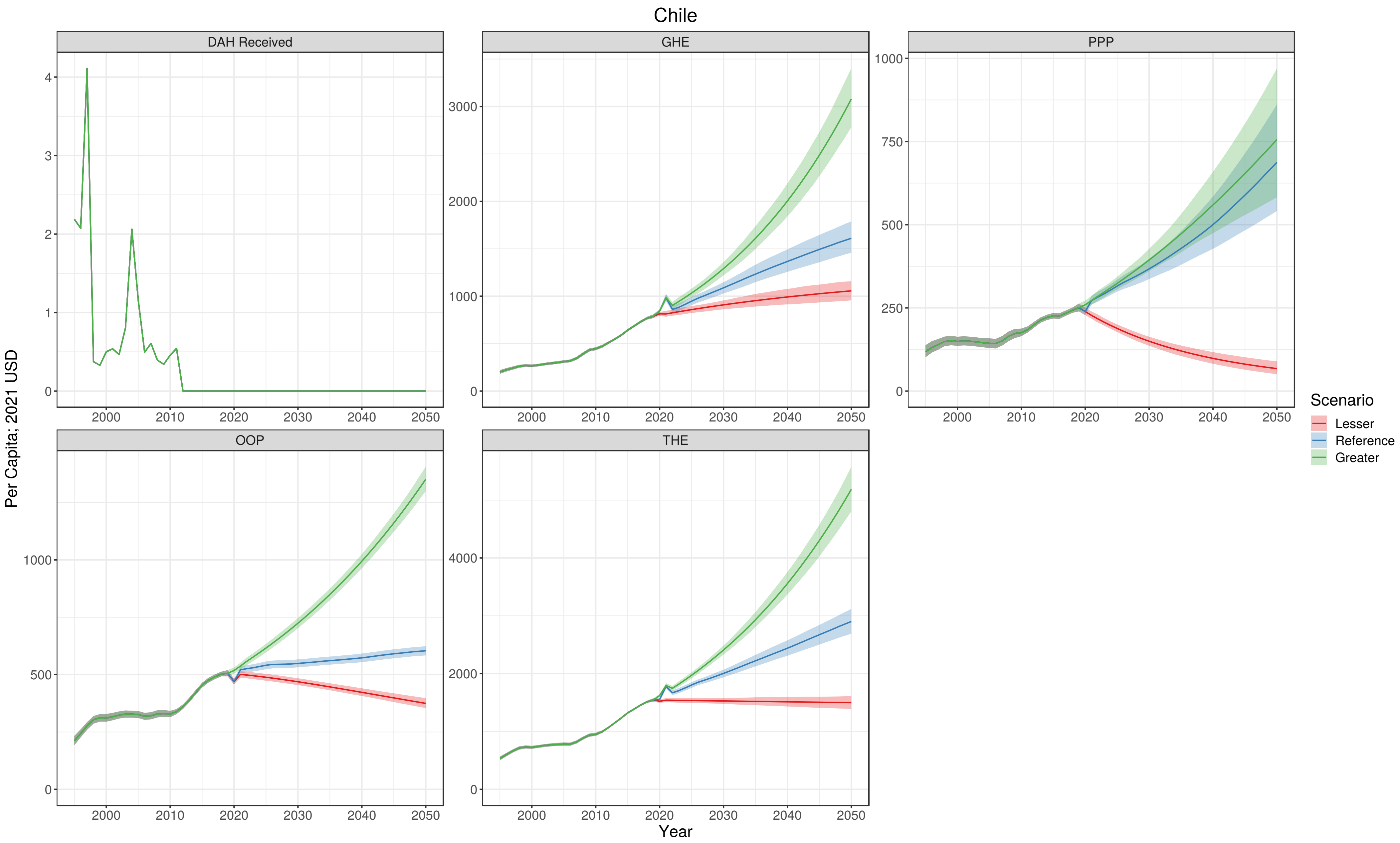

Chile
DAH Received
GHE
PPP
OOP
THE
Per Capita; 2021 USD
Year
Scenario
Lesser
Reference
Greater
0
1
2
3
4
1000
2000
3000
250
500
750
500
4000
2000
2010
2020
2030
2040
2050

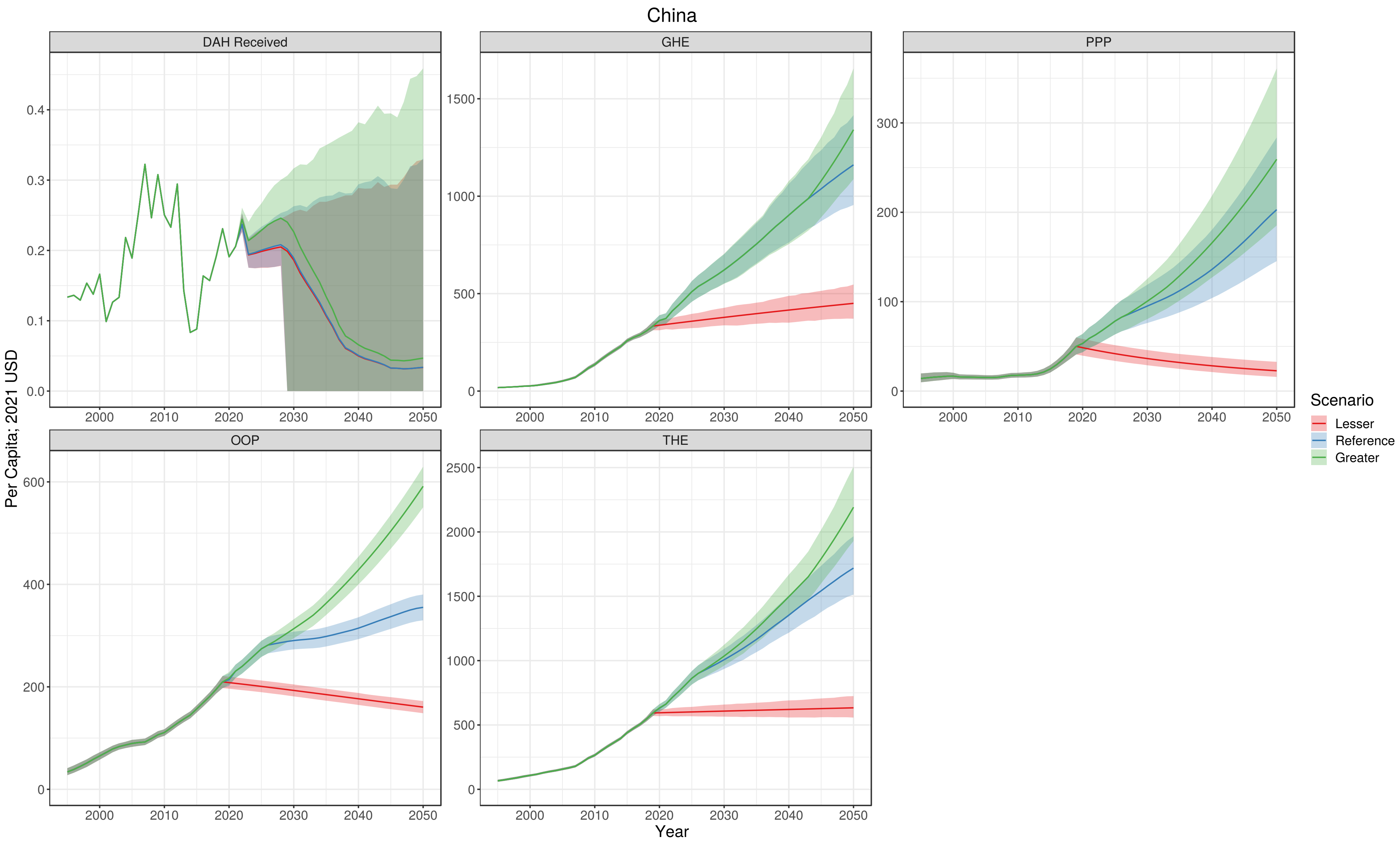
China
DAH Received
GHE
PPP
OOP
THE
Per Capita; 2021 USD
Year
Scenario
Lesser
Reference
Greater
0.0
0.1
0.2
0.3
0.4
0
500
1000
1500
100
200
300
400
600
2000
2500
2000
2010
2020
2030
2040
2050

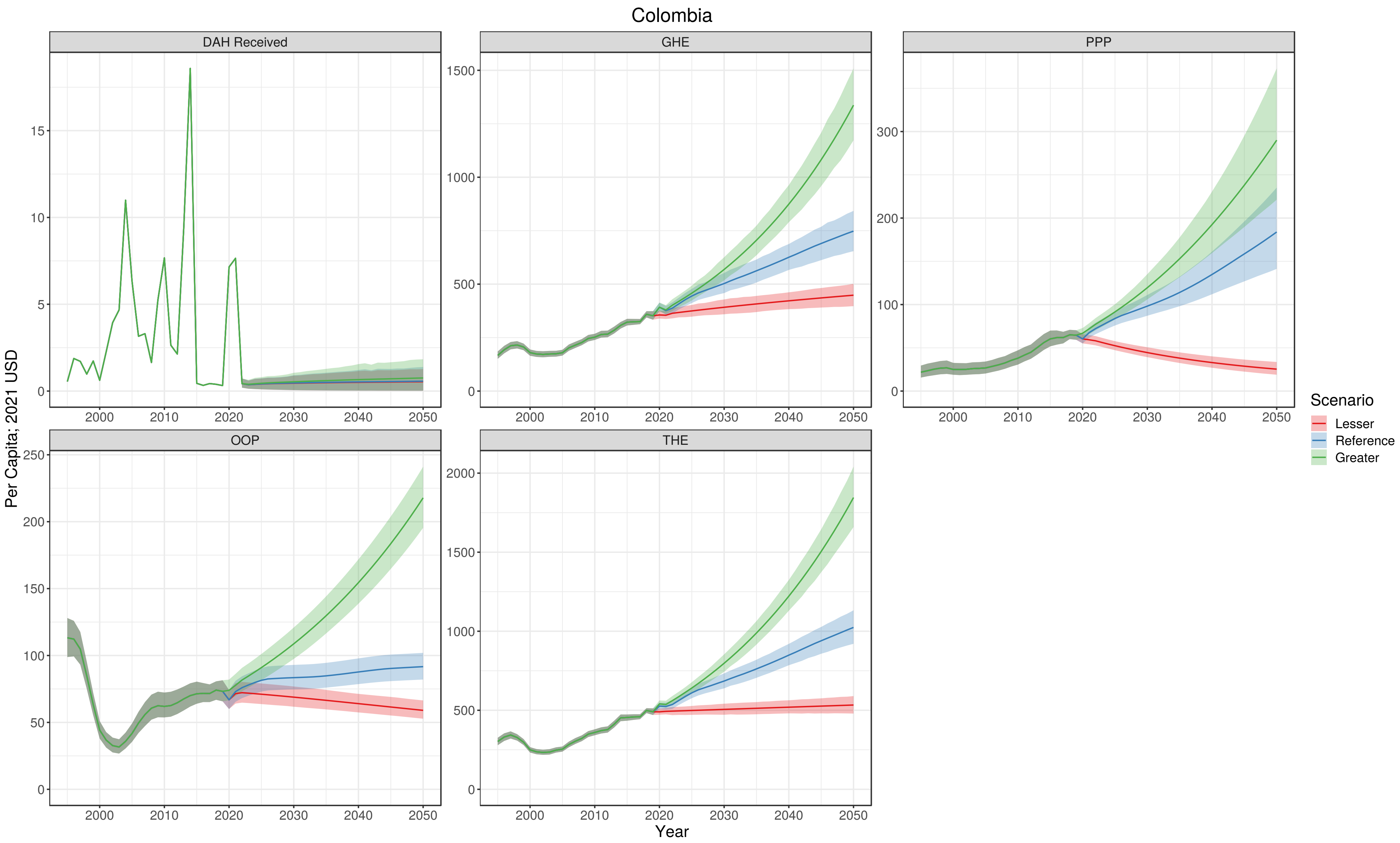

Colombia
DAH Received
GHE
PPP
OOP
THE
Per Capita; 2021 USD
Year
Scenario
Lesser
Reference
Greater

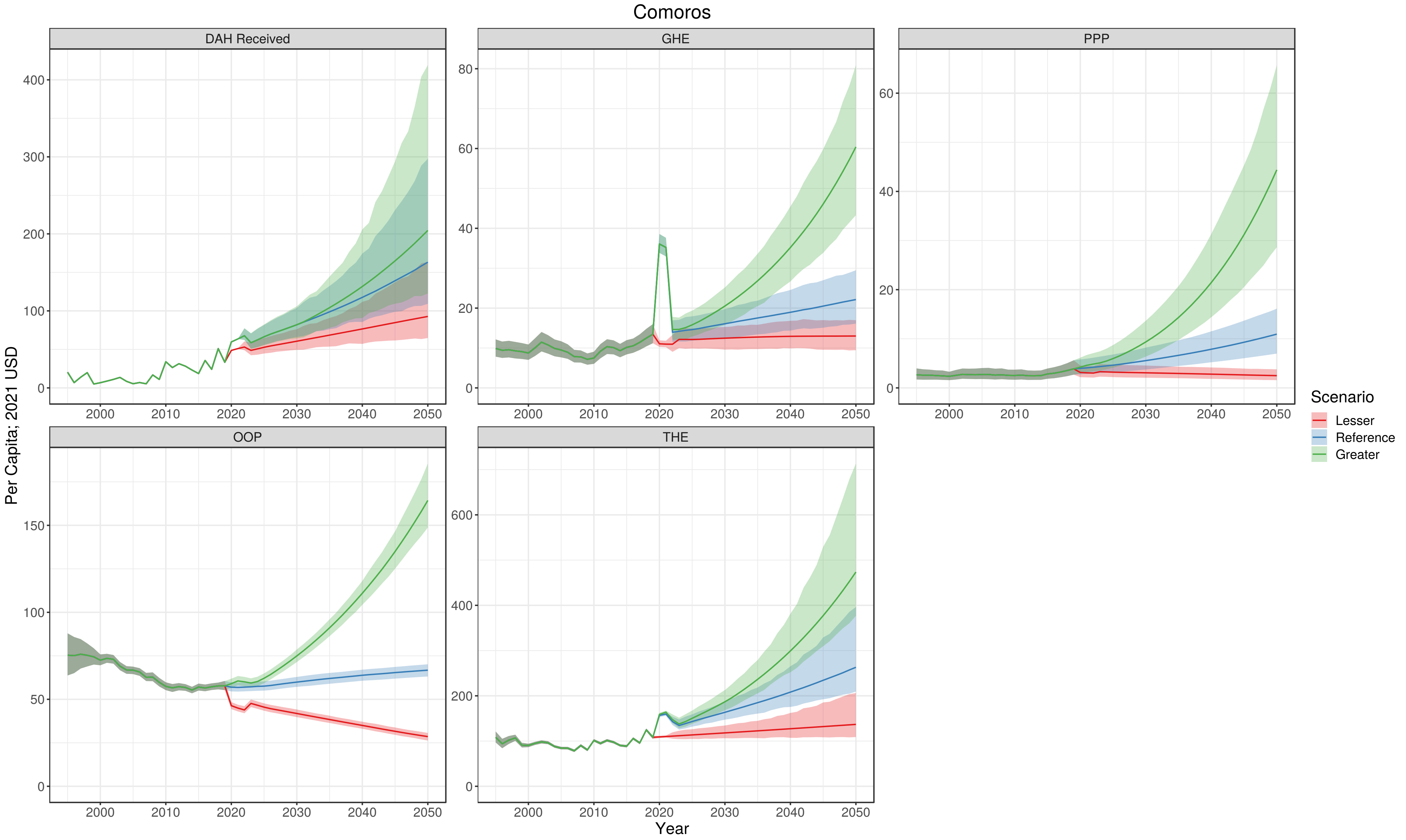

Comoros
DAH Received
GHE
PPP
OOP
THE
Per Capita; 2021 USD
Year
Scenario
Lesser
Reference
Greater

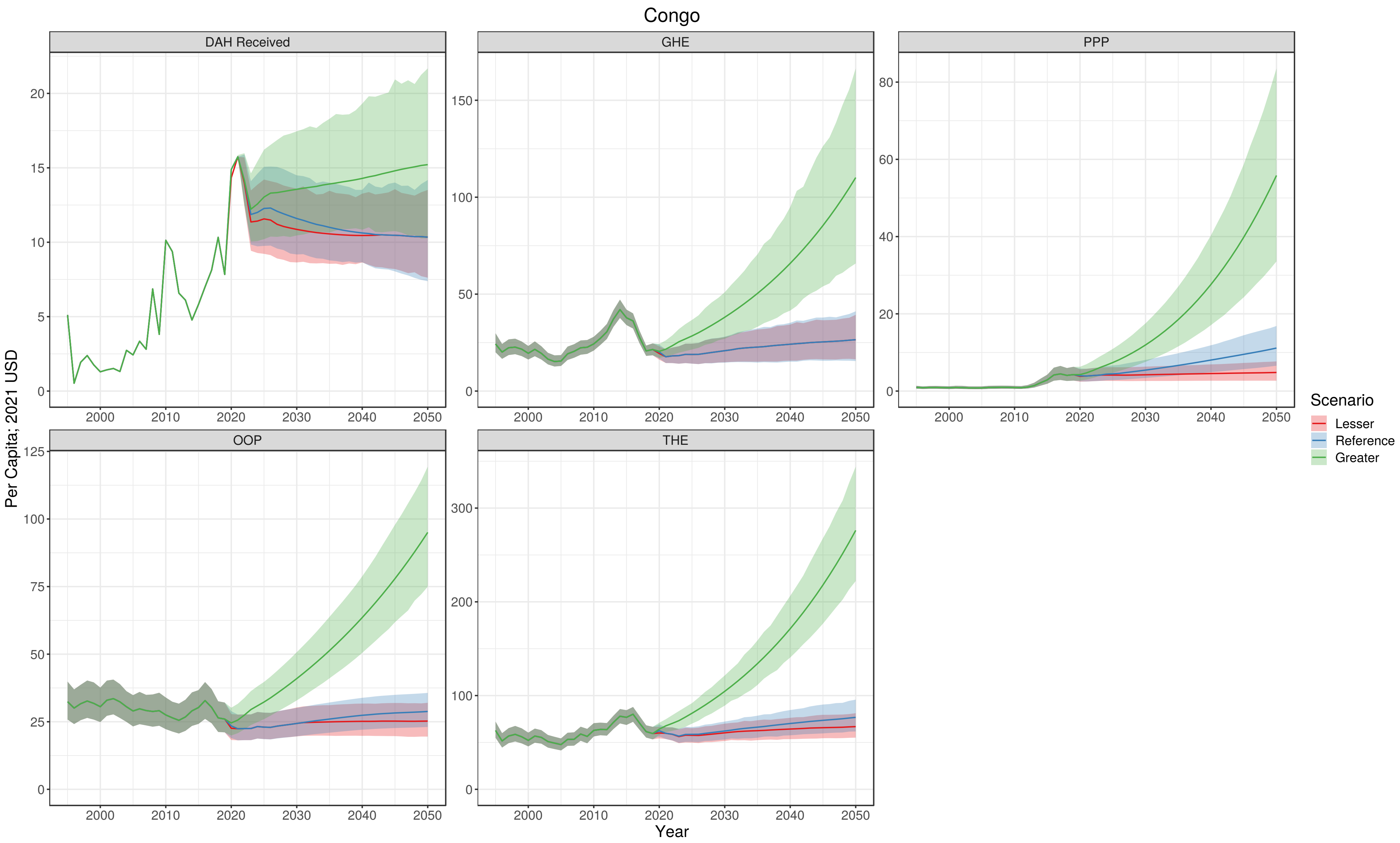

Congo
DAH Received
GHE
PPP
OOP
THE
Per Capita; 2021 USD
Year
Scenario
Lesser
Reference
Greater
0
5
10
15
20
50
100
150
20
40
60
80
25
50
75
100
125
200
300
2000
2010
2020
2030
2040
2050

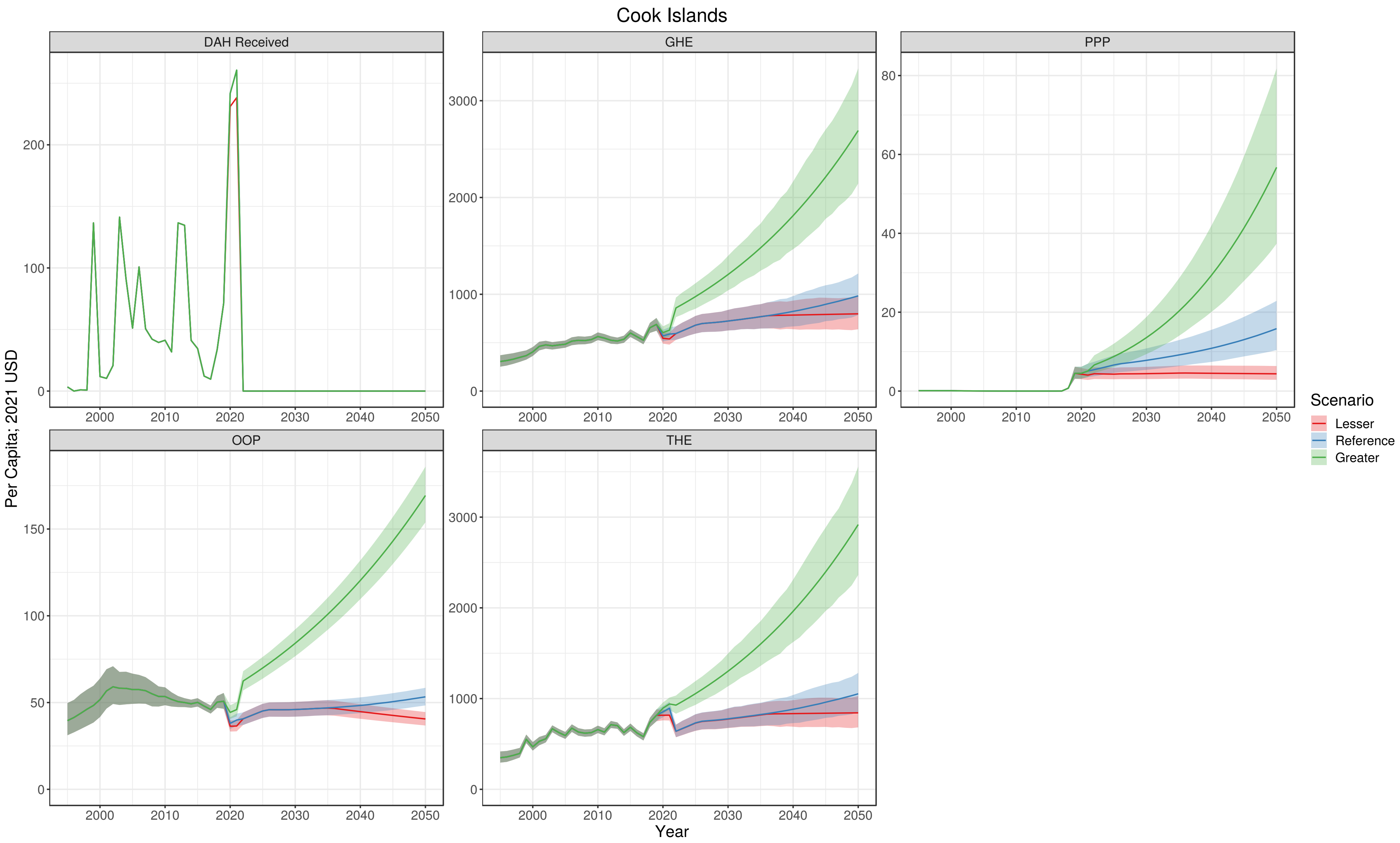

Cook Islands
DAH Received
GHE
PPP
OOP
THE
Per Capita; 2021 USD
Year
Scenario
Lesser
Reference
Greater

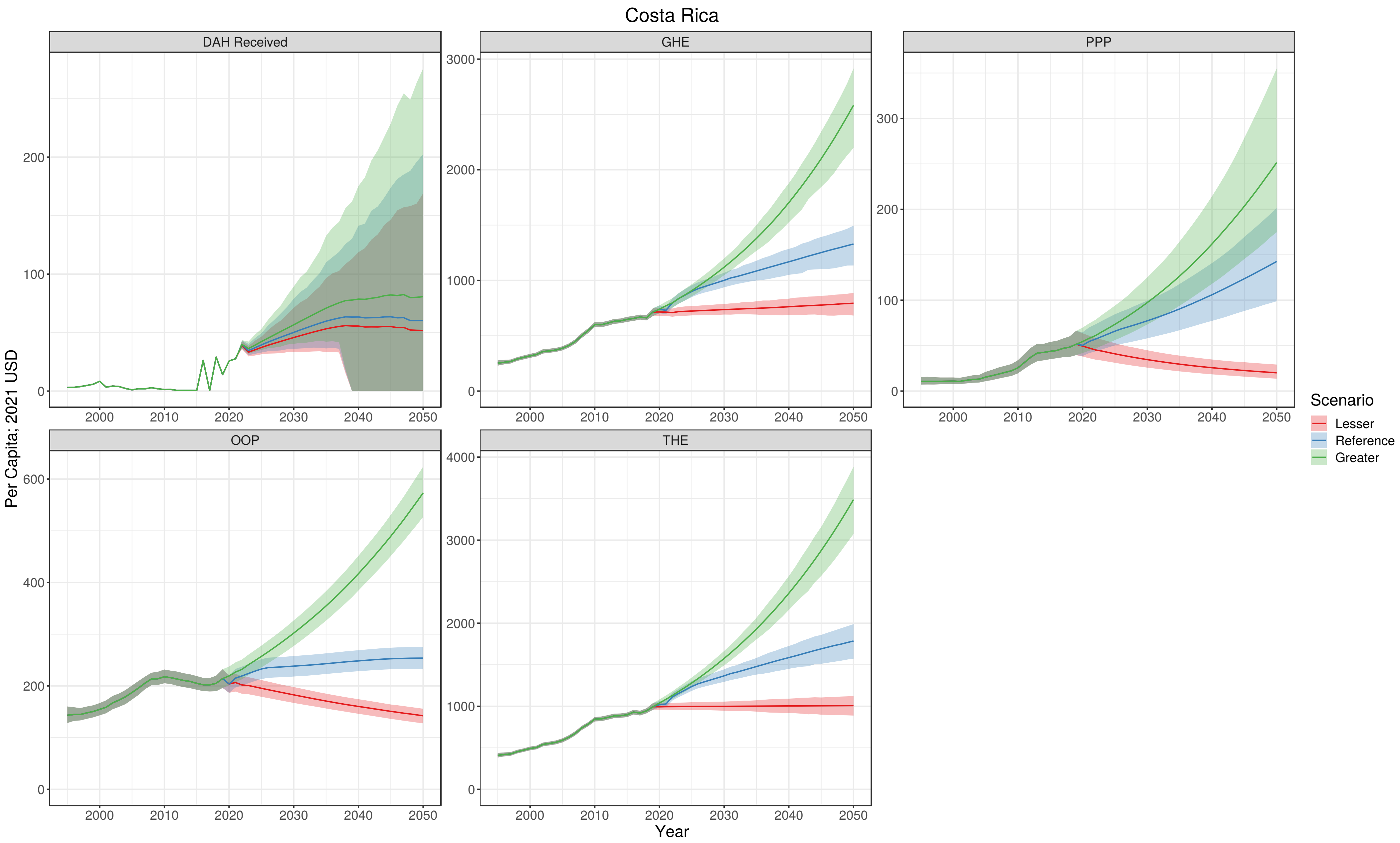
Costa Rica
DAH Received
GHE
PPP
OOP
THE
Per Capita; 2021 USD
Year
Scenario
Lesser
Reference
Greater
0
100
200
1000
2000
3000
300
400
600
4000
2000
2010
2020
2030
2040
2050

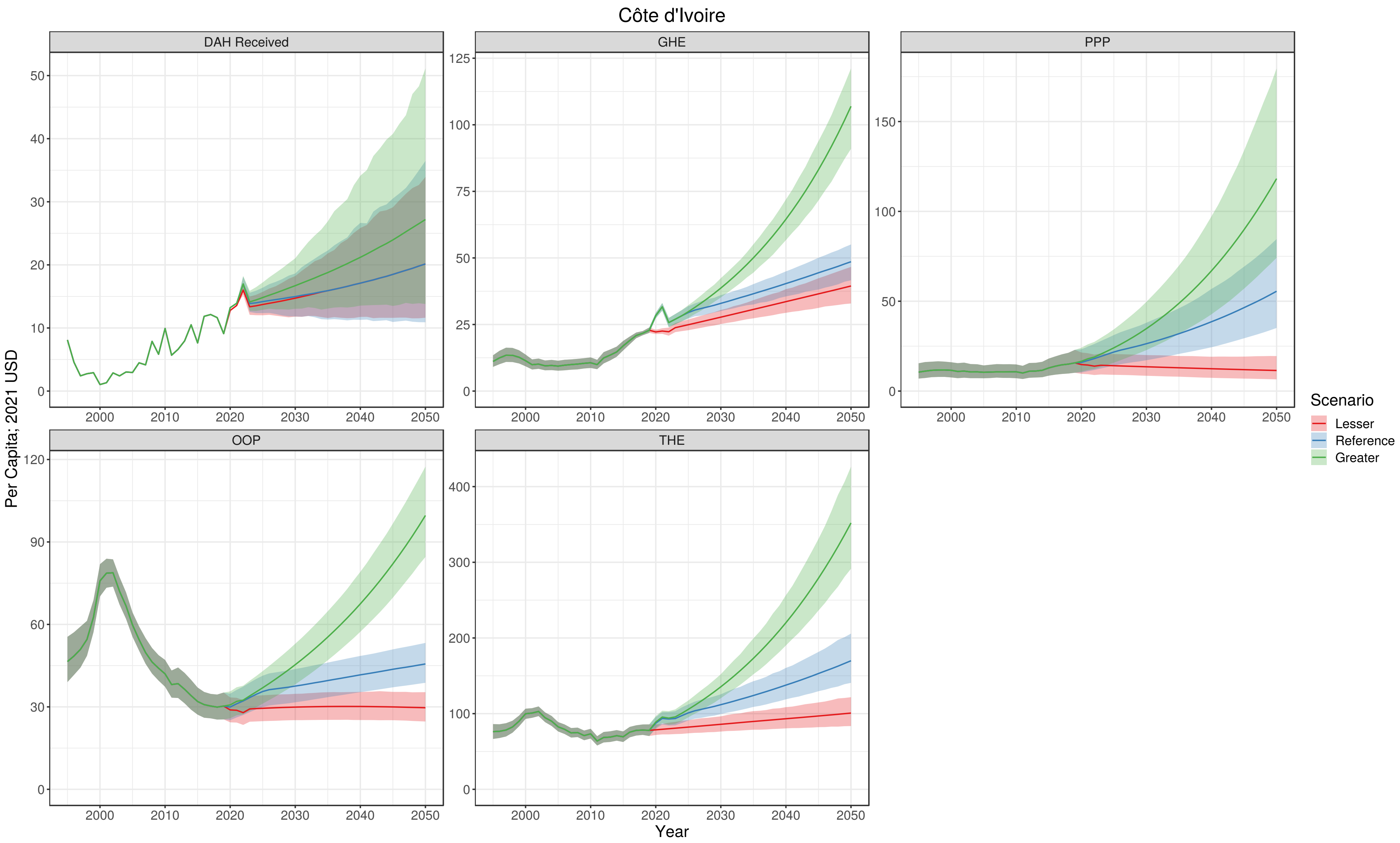

Côte d'Ivoire
DAH Received
GHE
PPP
OOP
THE
Per Capita; 2021 USD
Year
Scenario
Lesser
Reference
Greater

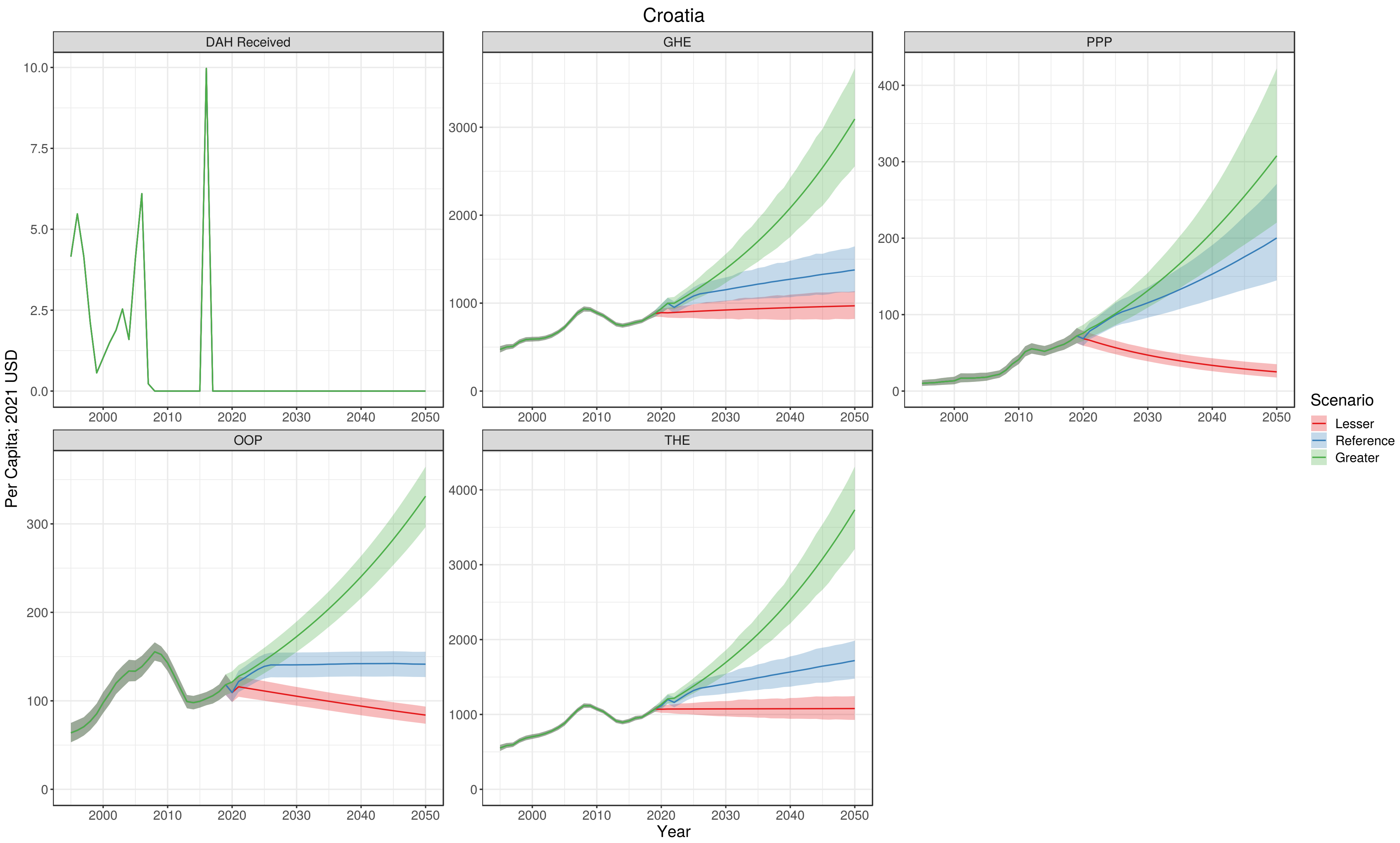

Croatia
DAH Received
GHE
PPP
OOP
THE
Per Capita; 2021 USD
Year
Scenario
Lesser
Reference
Greater
10.0
7.5
5.0
2.5
0.0
3000
2000
1000
0
400
300
200
100
4000
2000
2010
2020
2030
2040
2050

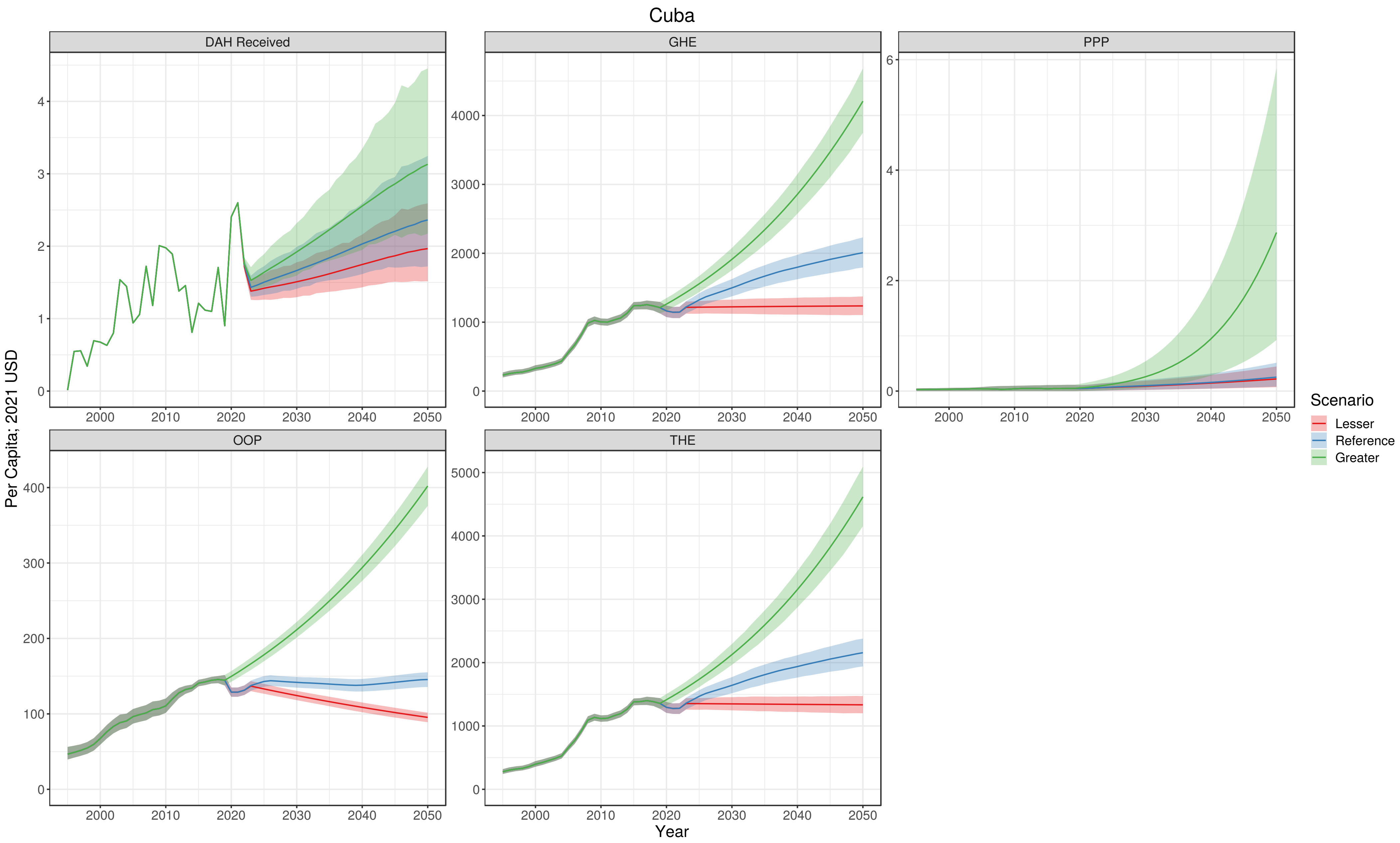
Cuba
DAH Received
GHE
PPP
OOP
THE
Per Capita; 2021 USD
Year
Scenario
Lesser
Reference
Greater

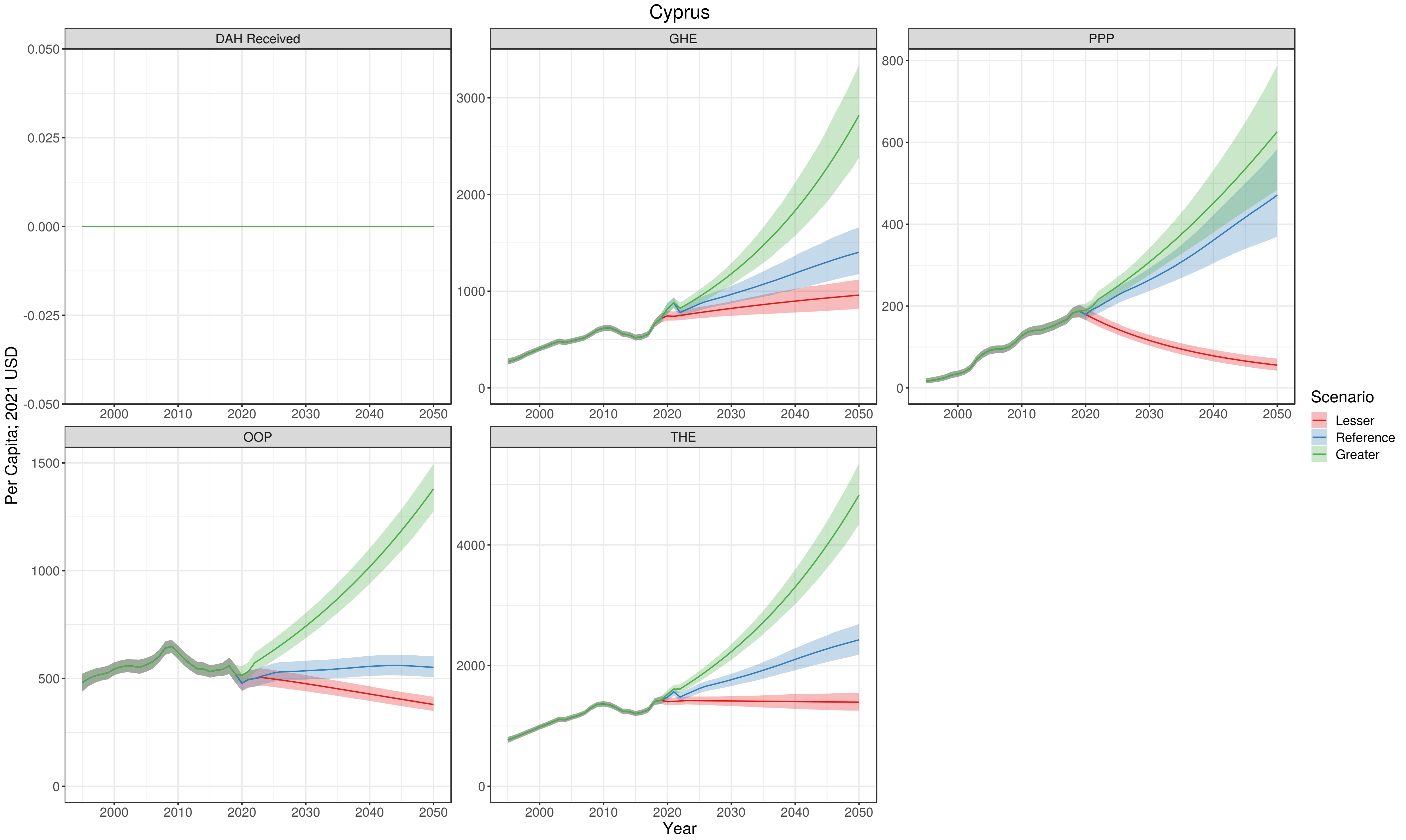
Cyprus
DAH Received
GHE
PPP
OOP
THE
Per Capita; 2021 USD
Year
Scenario
Lesser
Reference
Greater
0.050
0.025
0.000
-0.025
-0.050
3000
2000
1000
0
800
600
400
200
1500
500
4000
2000
2010
2020
2030
2040
2050

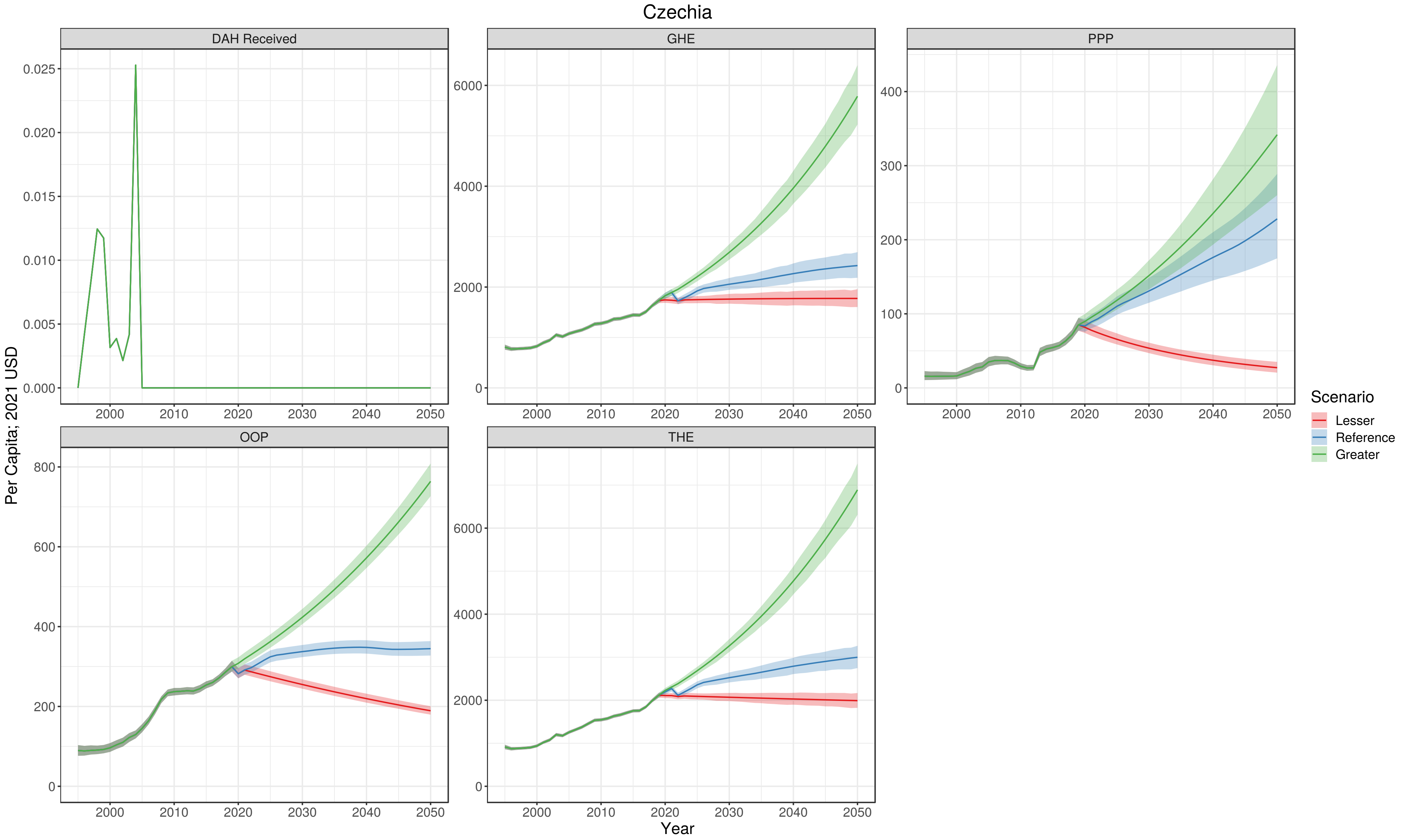

Czechia
DAH Received
GHE
PPP
OOP
THE
Per Capita; 2021 USD
Year
Scenario
Lesser
Reference
Greater
0.000
0.005
0.010
0.015
0.020
0.025
0
2000
4000
6000
0
100
200
300
400
0
200
400
600
800
2000
2010
2020
2030
2040
2050

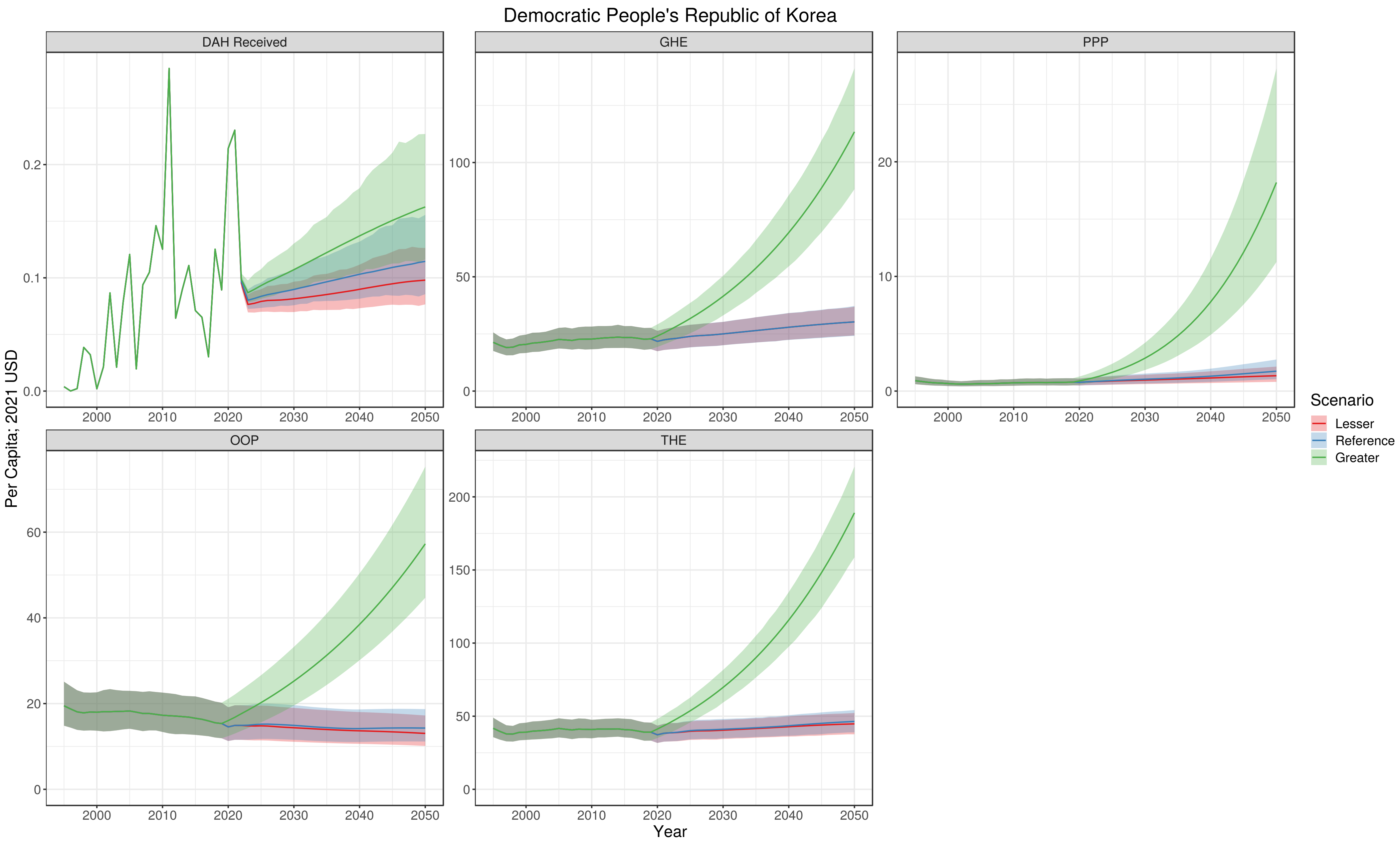
Democratic People's Republic of Korea
DAH Received
GHE
PPP
OOP
THE
Per Capita; 2021 USD
Year
Scenario
Lesser
Reference
Greater
0.0
0.1
0.2
0
50
100
10
20
40
60
150
200
2000
2010
2020
2030
2040
2050

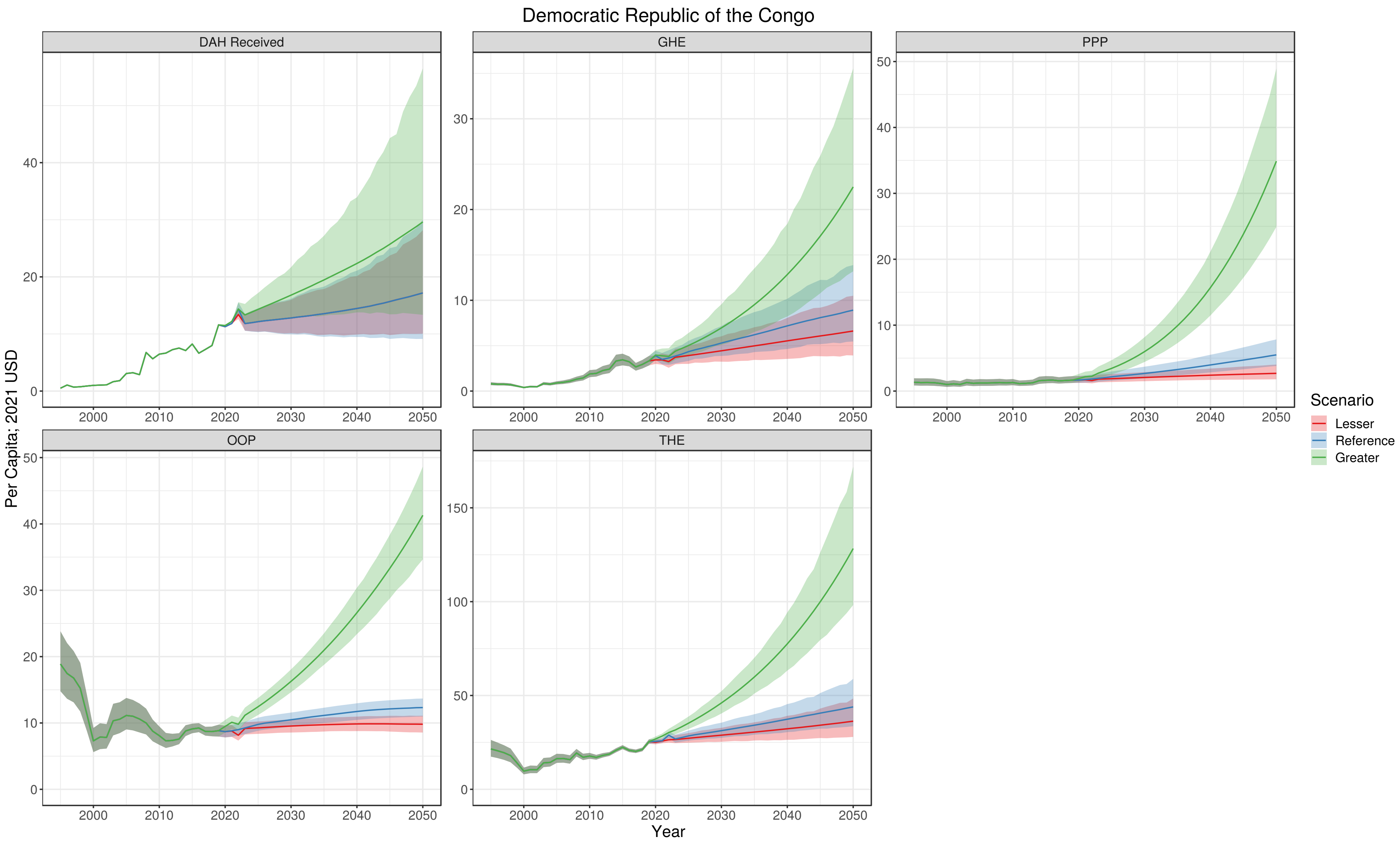
Democratic Republic of the Congo
DAH Received
GHE
PPP
OOP
THE
Per Capita; 2021 USD
Year
Scenario
Lesser
Reference
Greater
0
10
20
30
40
50
100
150
2000
2010
2020
2030
2040
2050

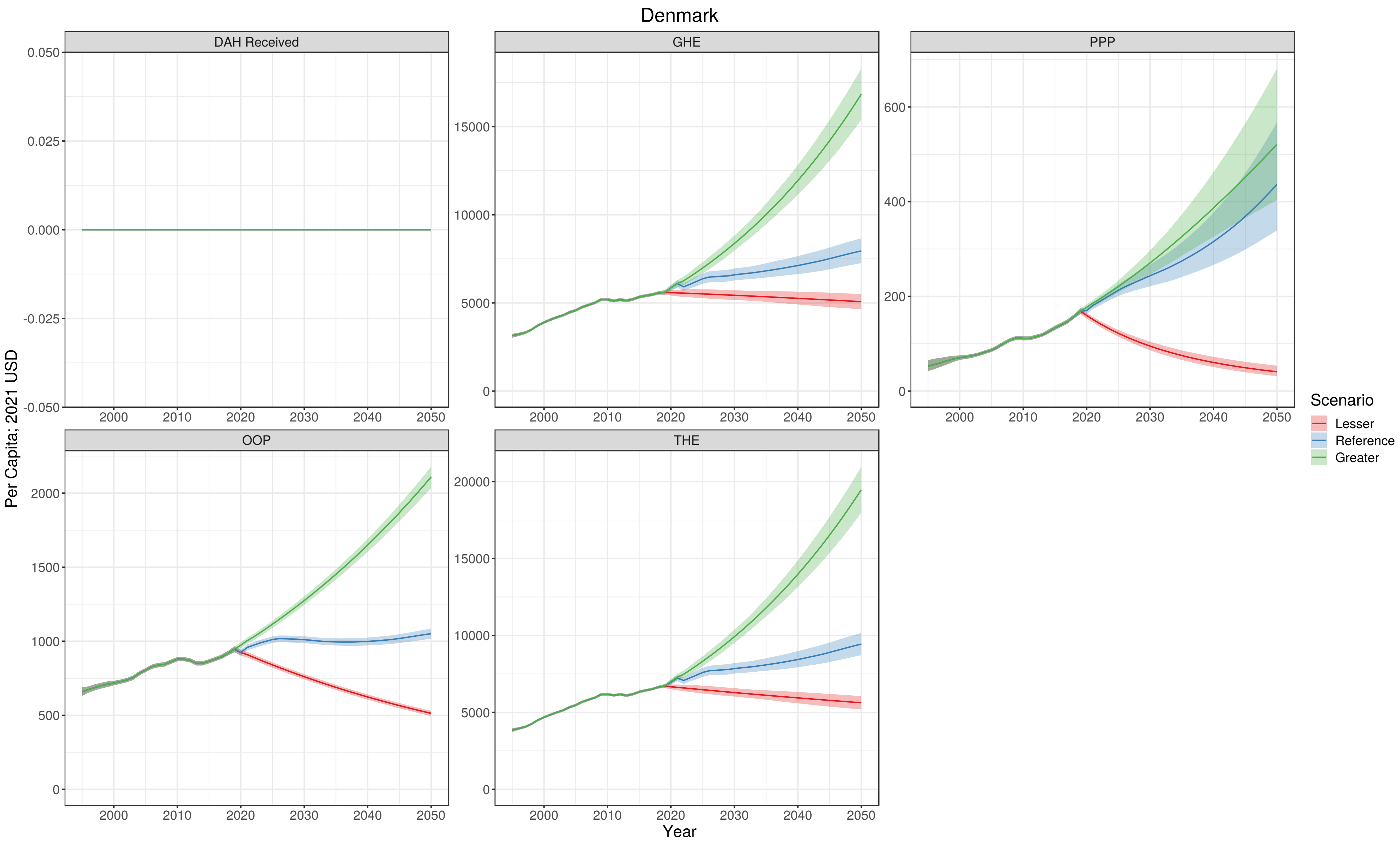

Denmark
DAH Received
GHE
PPP
OOP
THE
Per Capita; 2021 USD
Year
Scenario
Lesser
Reference
Greater

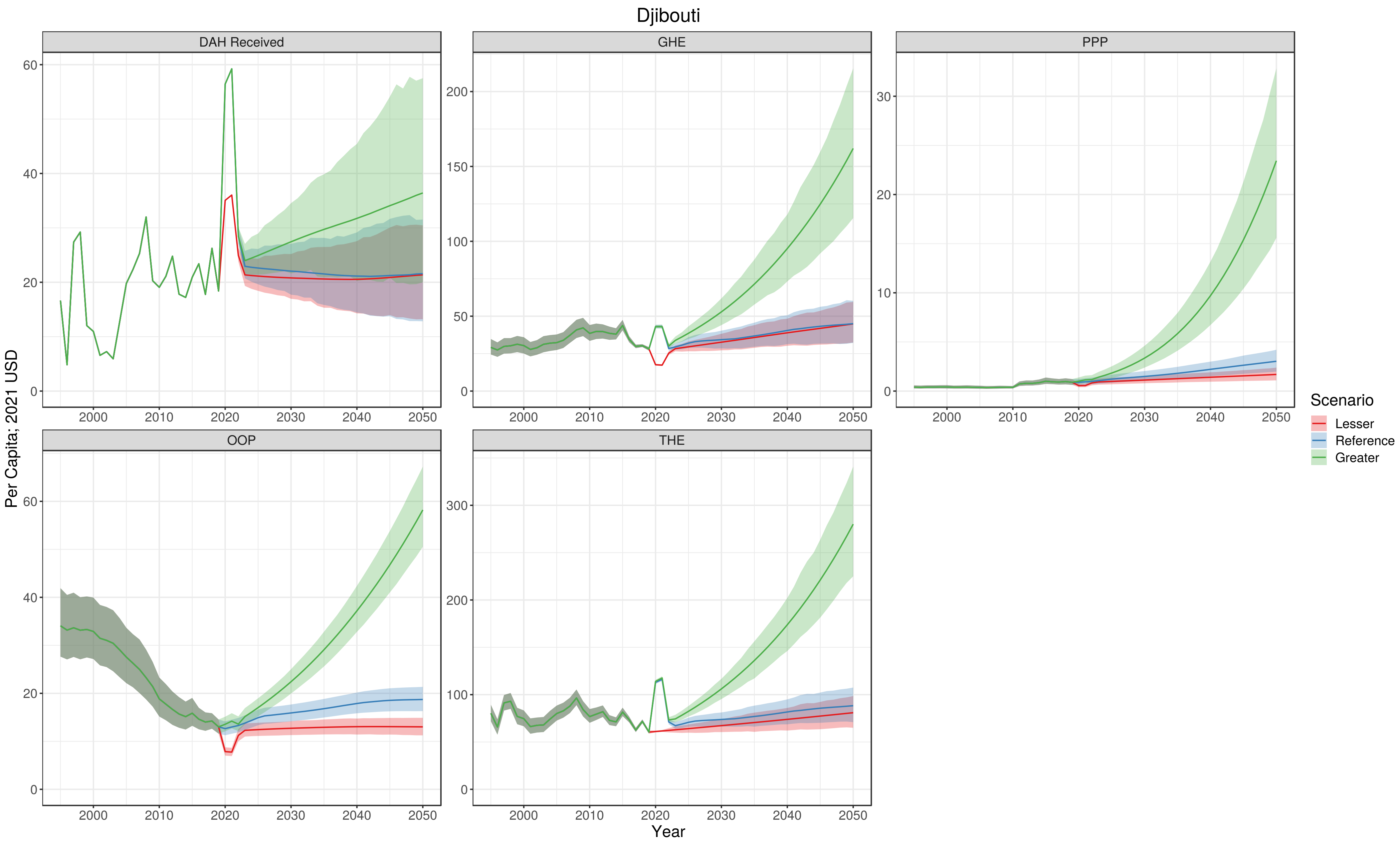
Djibouti
DAH Received
GHE
PPP
OOP
THE
Per Capita; 2021 USD
Year
Scenario
Lesser
Reference
Greater
0
20
40
60
0
50
100
150
200
0
10
20
30
0
20
40
60
0
100
200
300
2000
2010
2020
2030
2040
2050

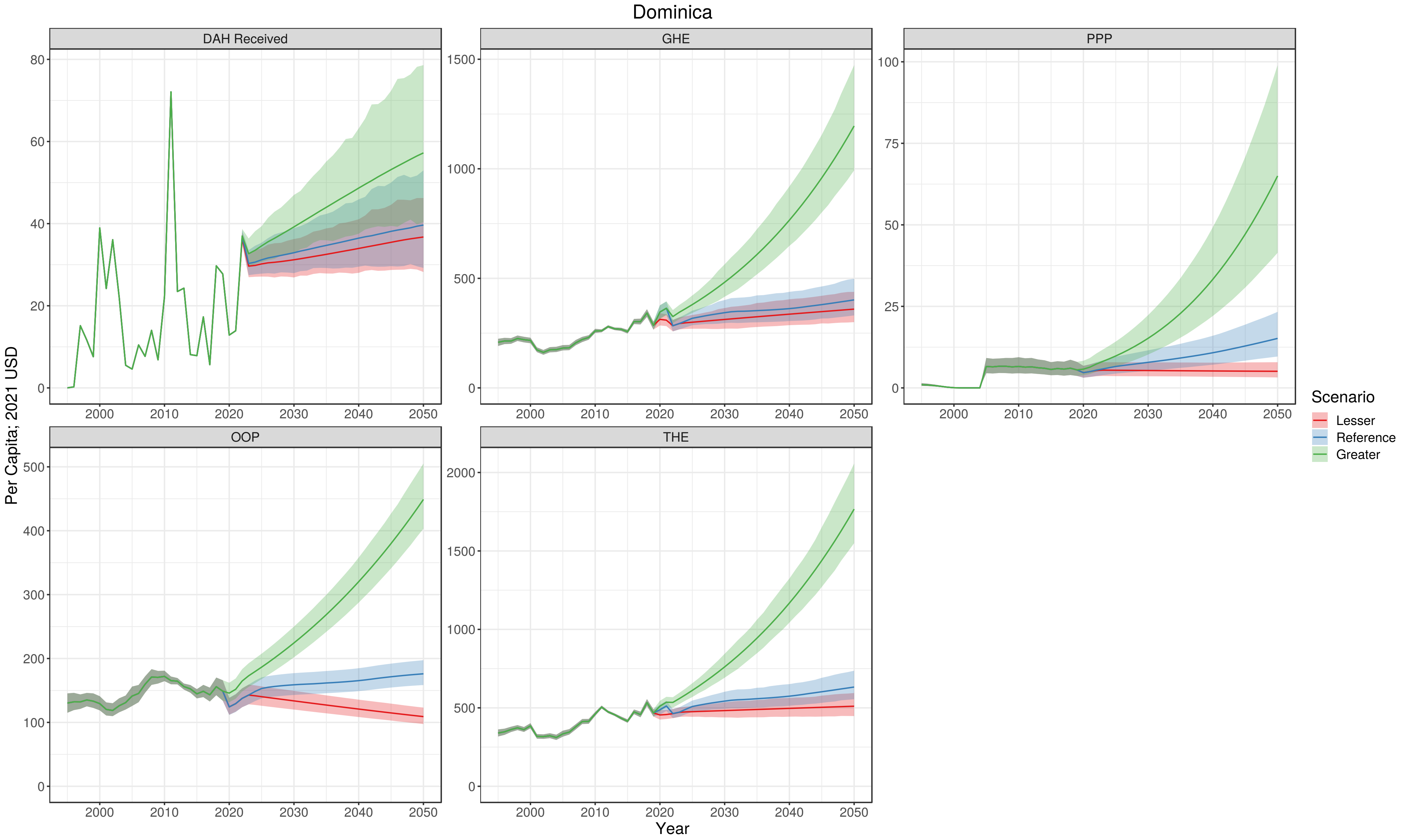

Dominica
DAH Received
GHE
PPP
OOP
THE
Per Capita; 2021 USD
Year
Scenario
Lesser
Reference
Greater

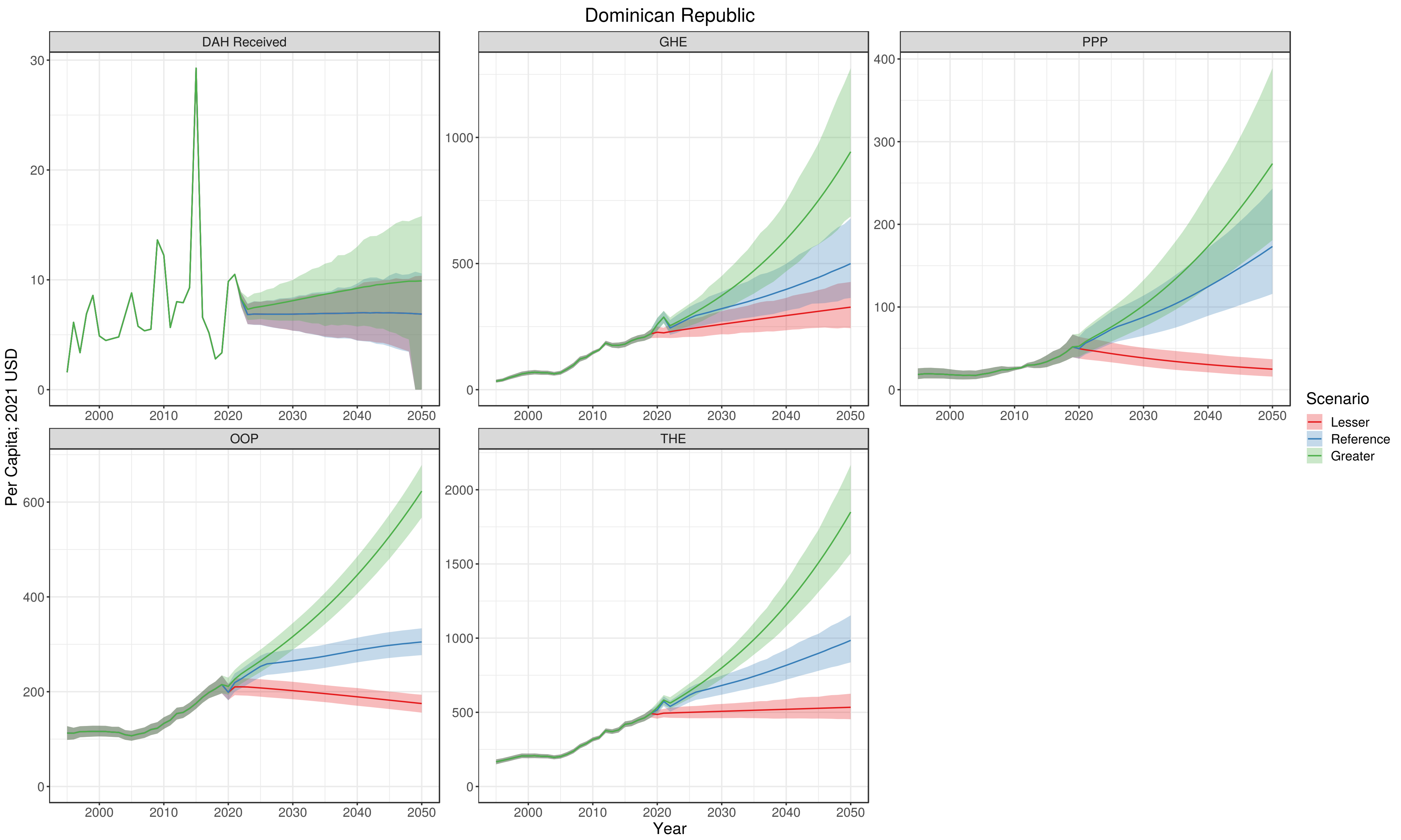

Dominican Republic
DAH Received
GHE
PPP
OOP
THE
Per Capita; 2021 USD
Year
Scenario
Lesser
Reference
Greater

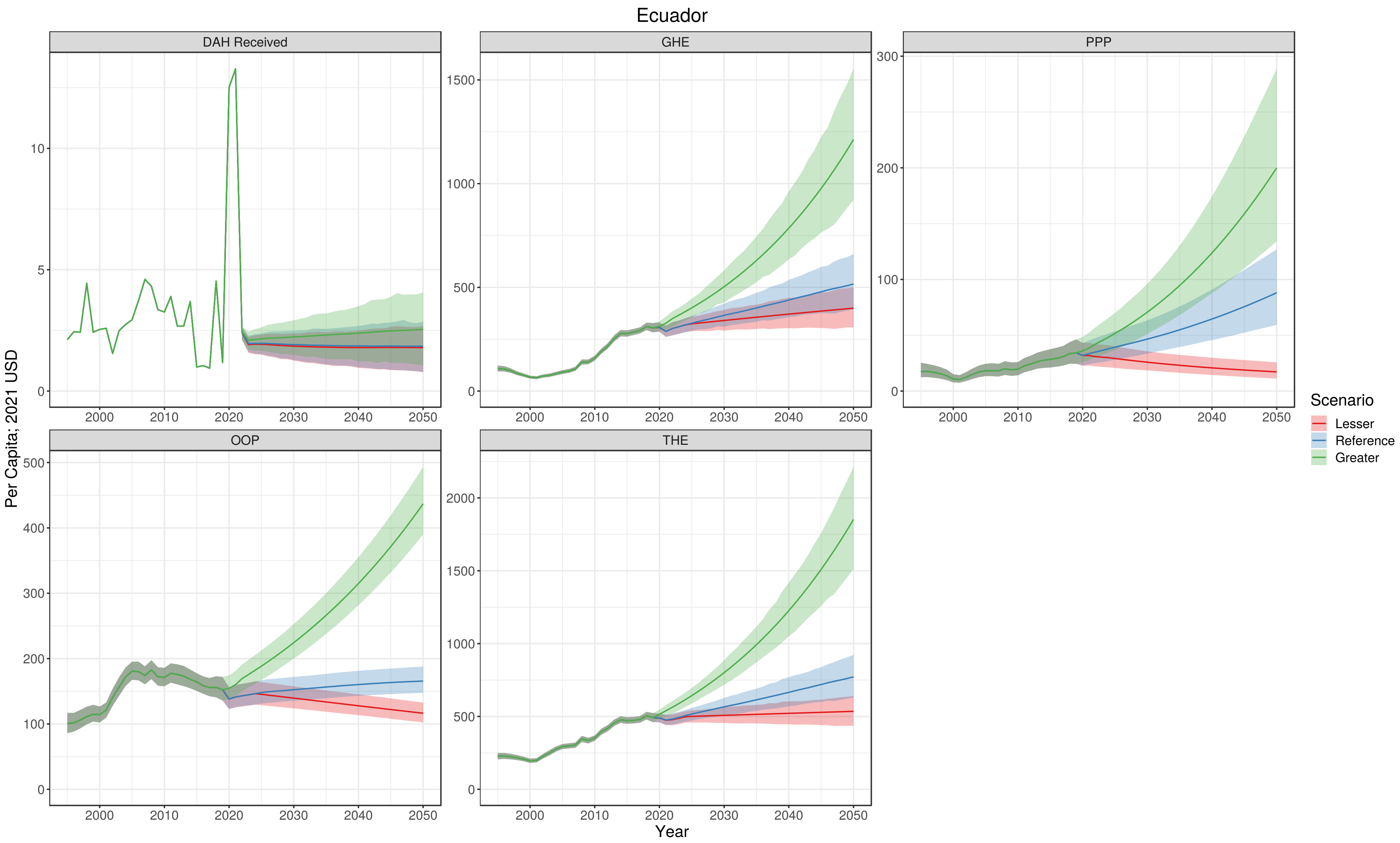

Ecuador
DAH Received
GHE
PPP
OOP
THE
Per Capita; 2021 USD
Year
Scenario
Lesser
Reference
Greater

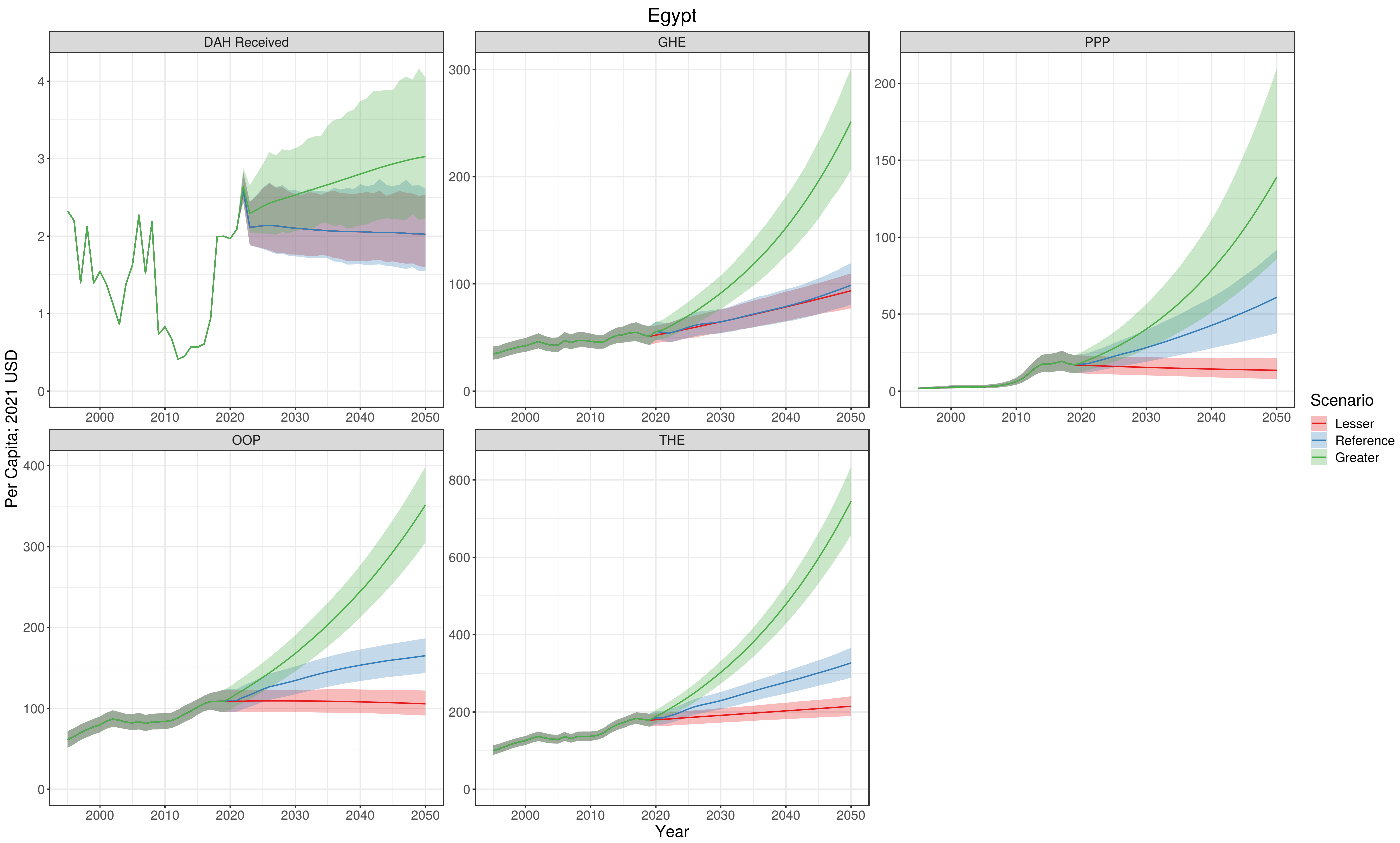
Egypt
DAH Received
GHE
PPP
OOP
THE
Per Capita; 2021 USD
Year
Scenario
Lesser
Reference
Greater

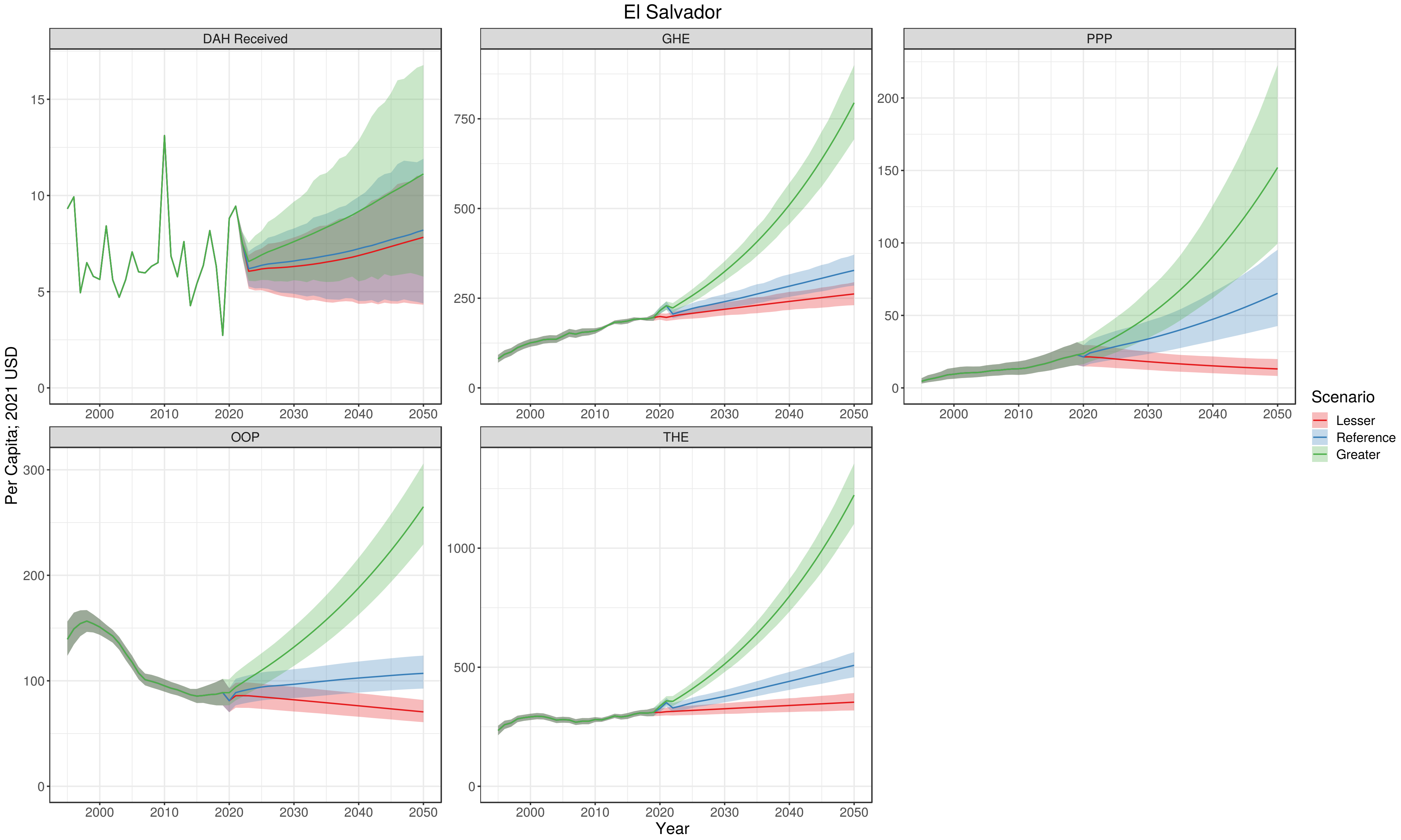
El Salvador
DAH Received
GHE
PPP
OOP
THE
Per Capita; 2021 USD
Year
Scenario
Lesser
Reference
Greater
0
5
10
15
250
500
750
50
100
150
200
300
1000
2000
2010
2020
2030
2040
2050

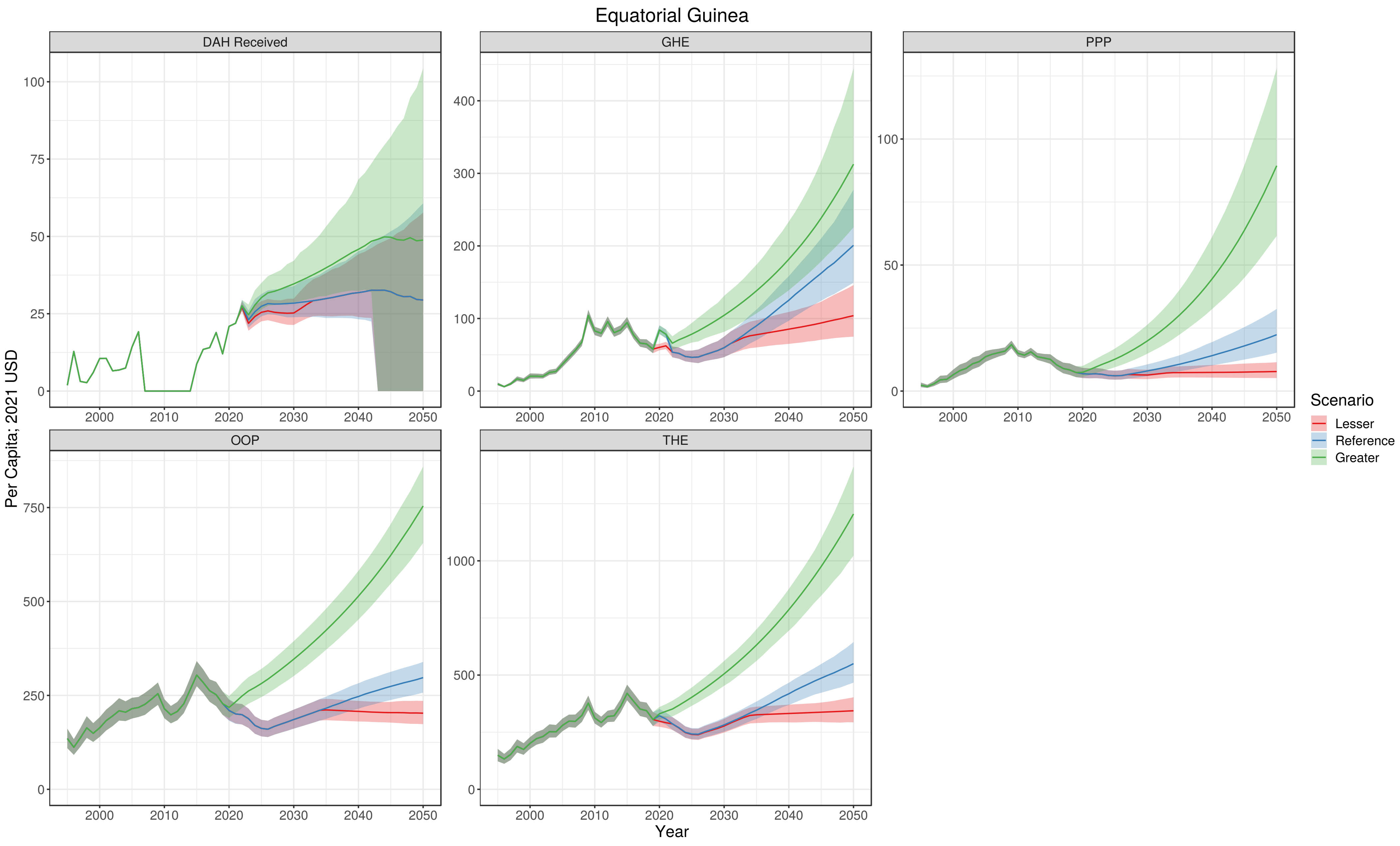

Equatorial Guinea
DAH Received
GHE
PPP
OOP
THE
Per Capita; 2021 USD
Year
Scenario
Lesser
Reference
Greater

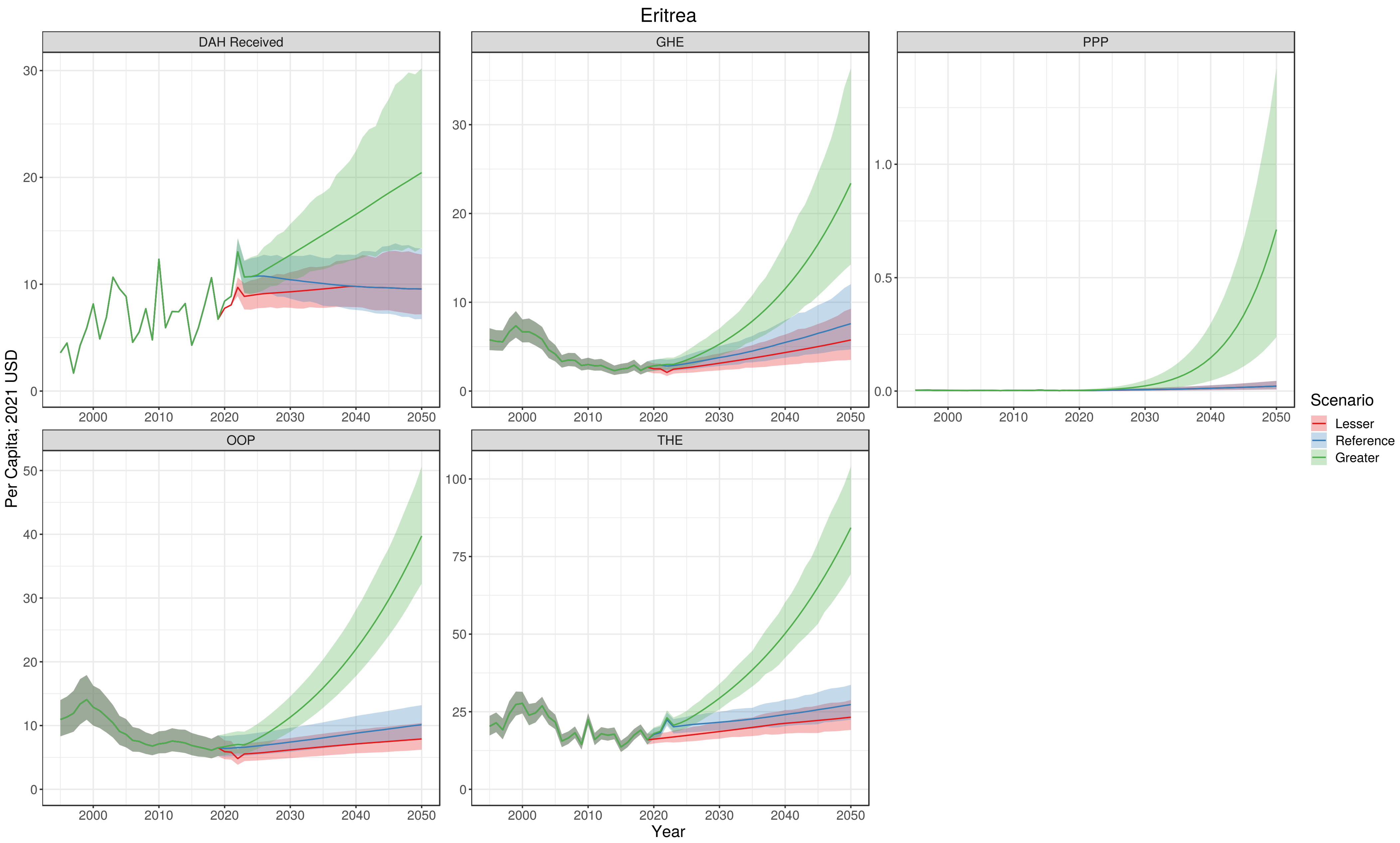

Eritrea
DAH Received
GHE
PPP
OOP
THE
Per Capita; 2021 USD
Year
Scenario
Lesser
Reference
Greater

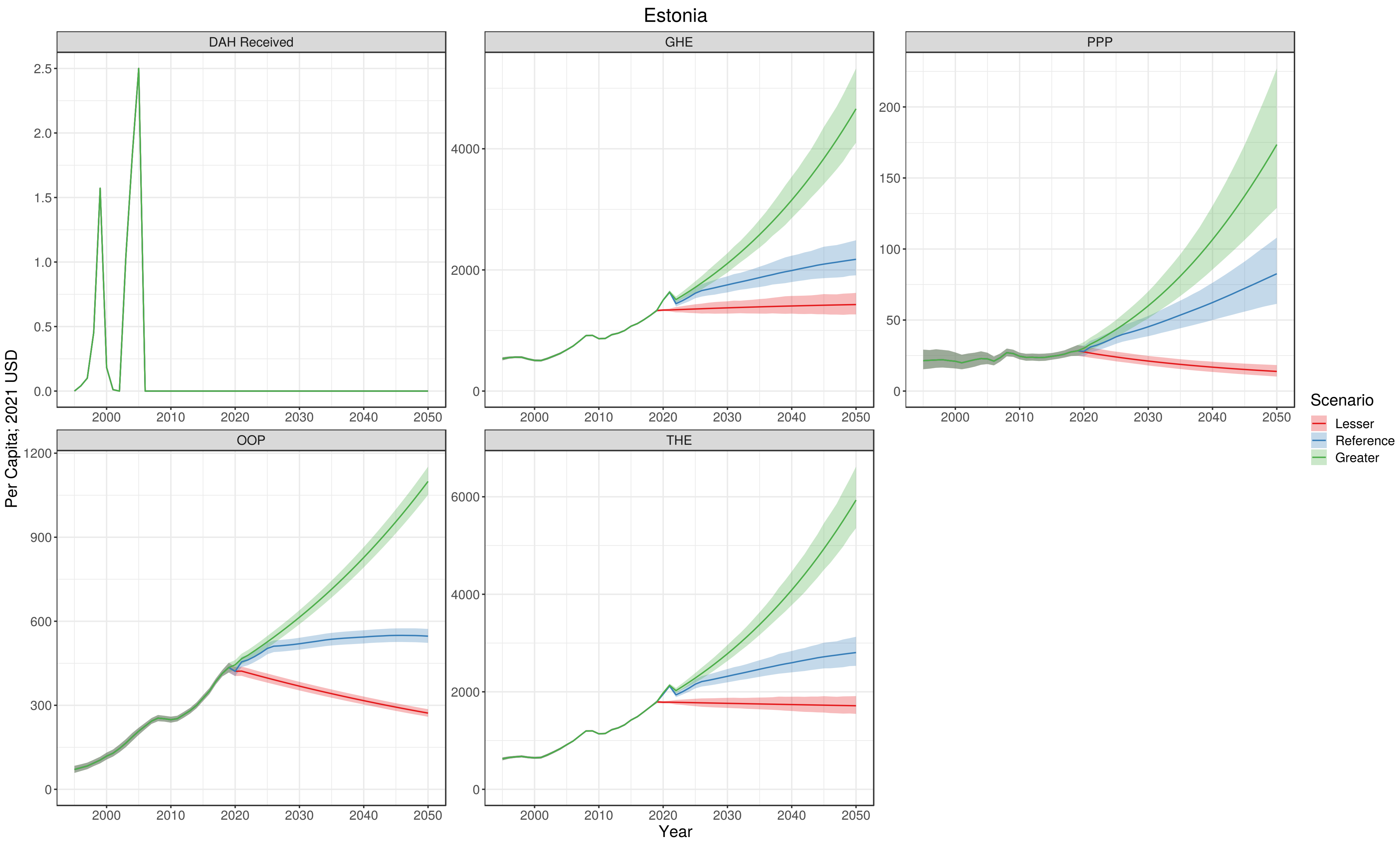

Estonia
DAH Received
GHE
PPP
OOP
THE
Per Capita; 2021 USD
Year
Scenario
Lesser
Reference
Greater

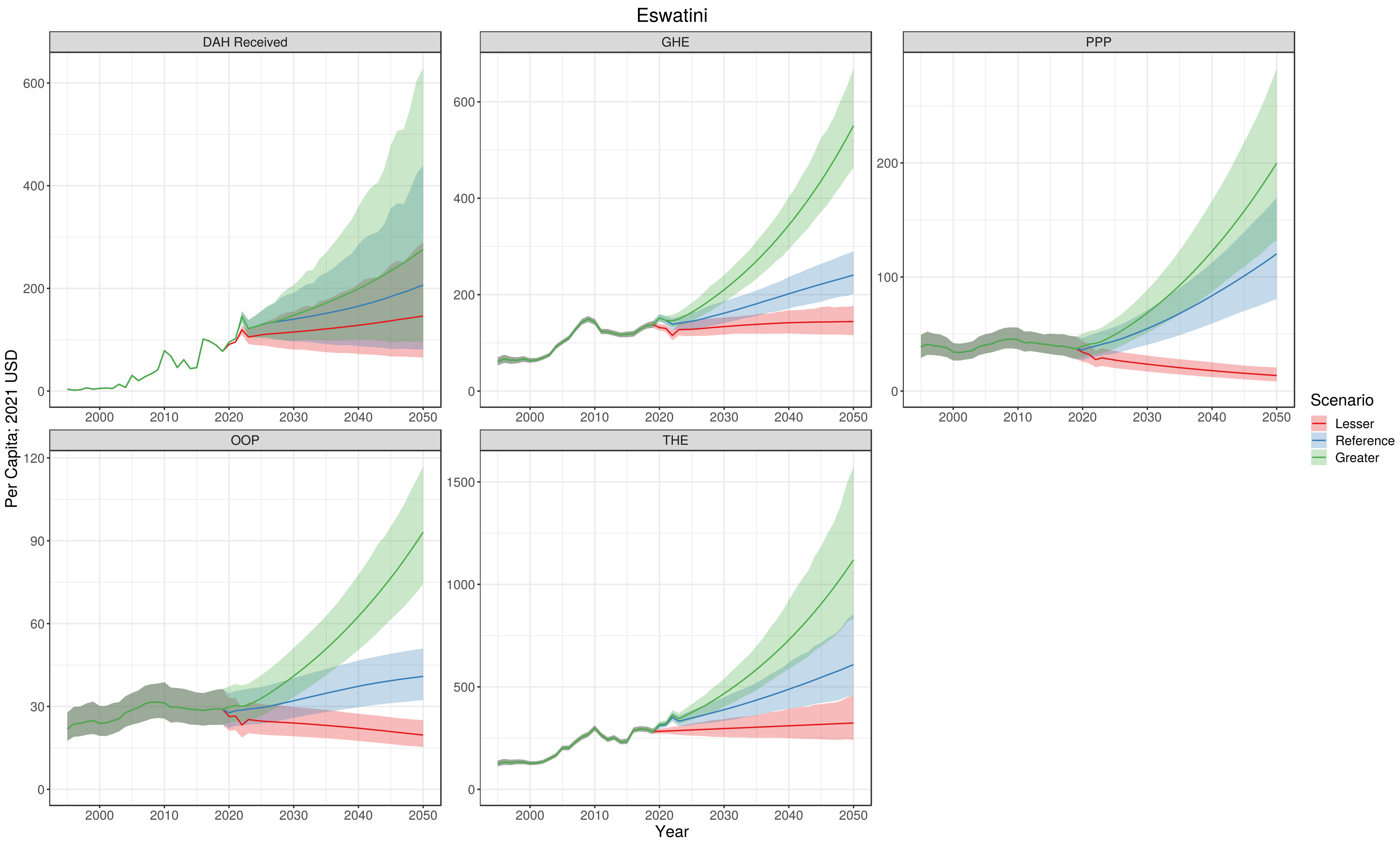

Eswatini
DAH Received
GHE
PPP
OOP
THE
Per Capita; 2021 USD
Year
Scenario
Lesser
Reference
Greater
0
200
400
600
100
30
60
90
120
500
1000
1500
2000
2010
2020
2030
2040
2050

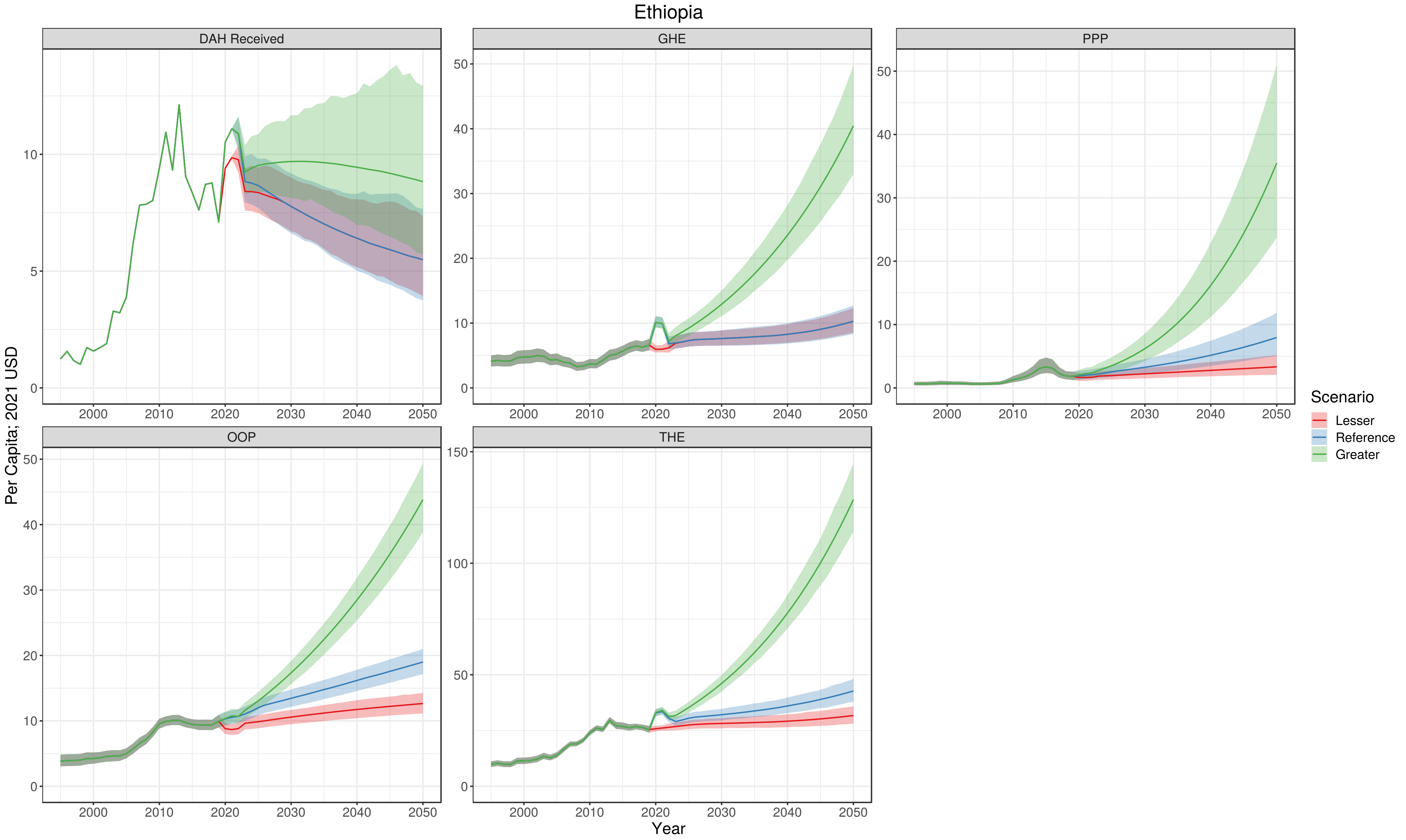

Ethiopia
DAH Received
GHE
PPP
OOP
THE
Per Capita; 2021 USD
Year
Scenario
Lesser
Reference
Greater
0
5
10
10
20
30
40
50
100
150
2000
2010
2020
2030
2040
2050

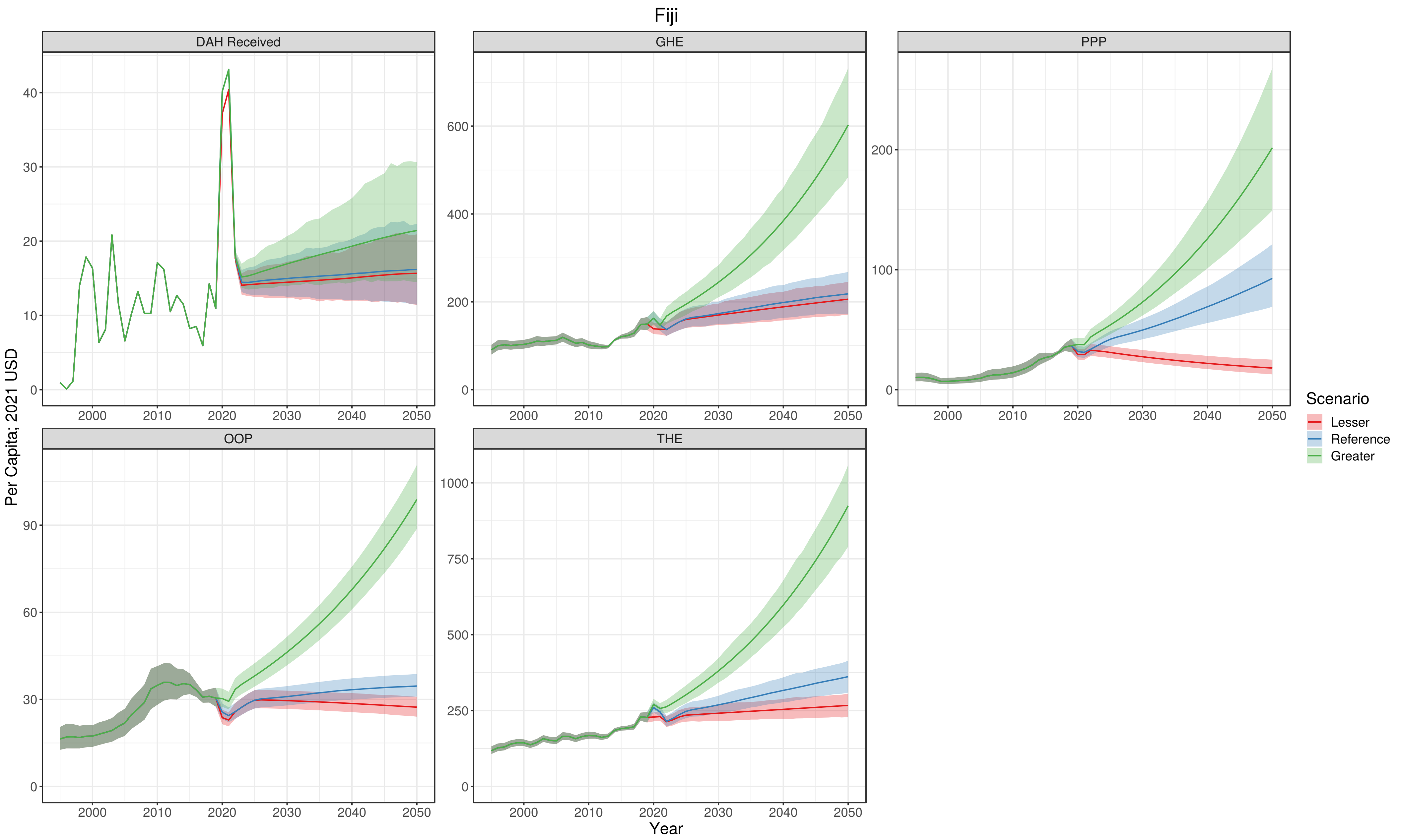

Fiji
DAH Received
GHE
PPP
OOP
THE
Per Capita; 2021 USD
Year
Scenario
Lesser
Reference
Greater

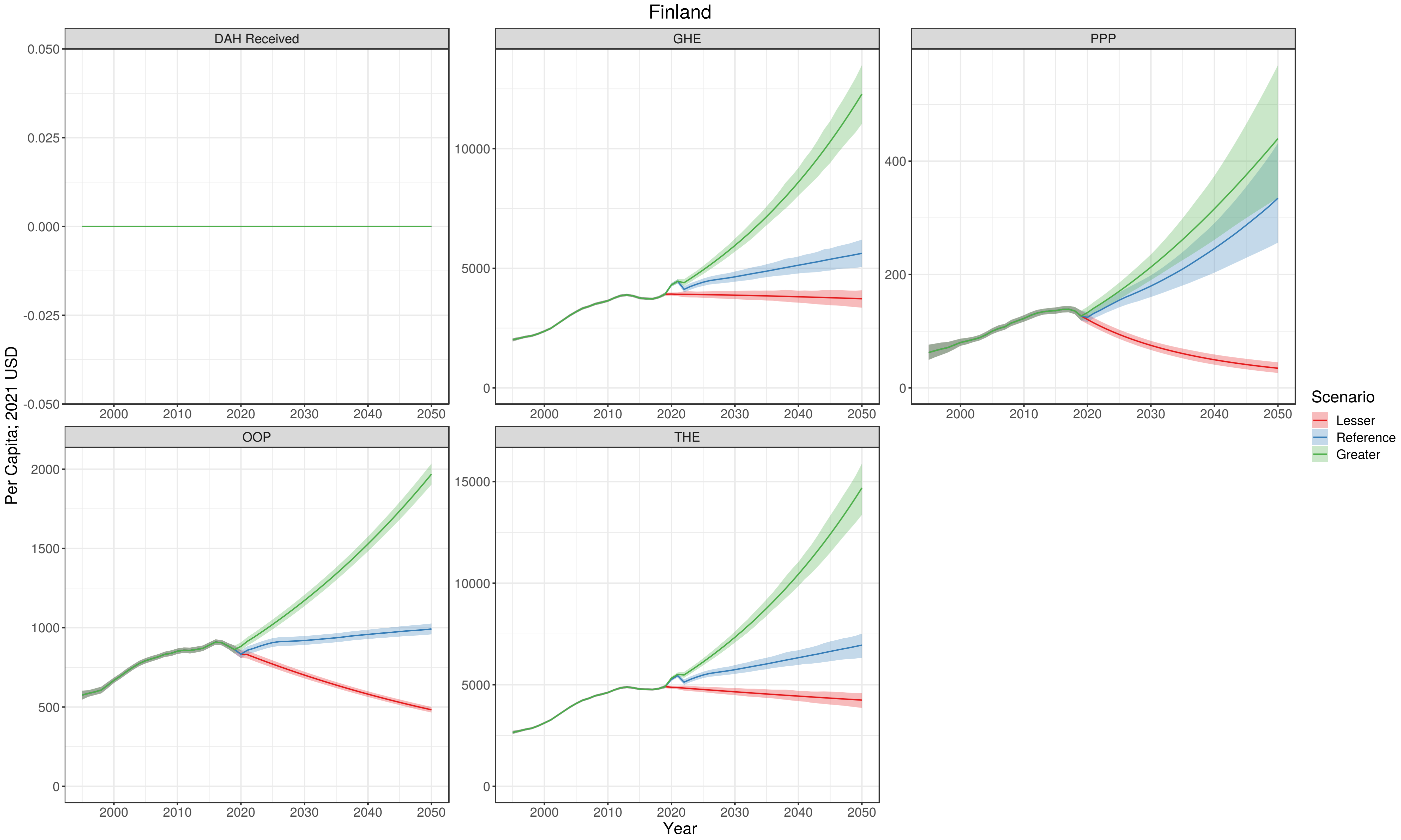

Finland
DAH Received
GHE
PPP
OOP
THE
Per Capita; 2021 USD
Year
Scenario
Lesser
Reference
Greater
0.050
0.025
0.000
-0.025
-0.050
10000
5000
0
400
200
2000
1500
1000
500
15000
2000
2010
2020
2030
2040
2050

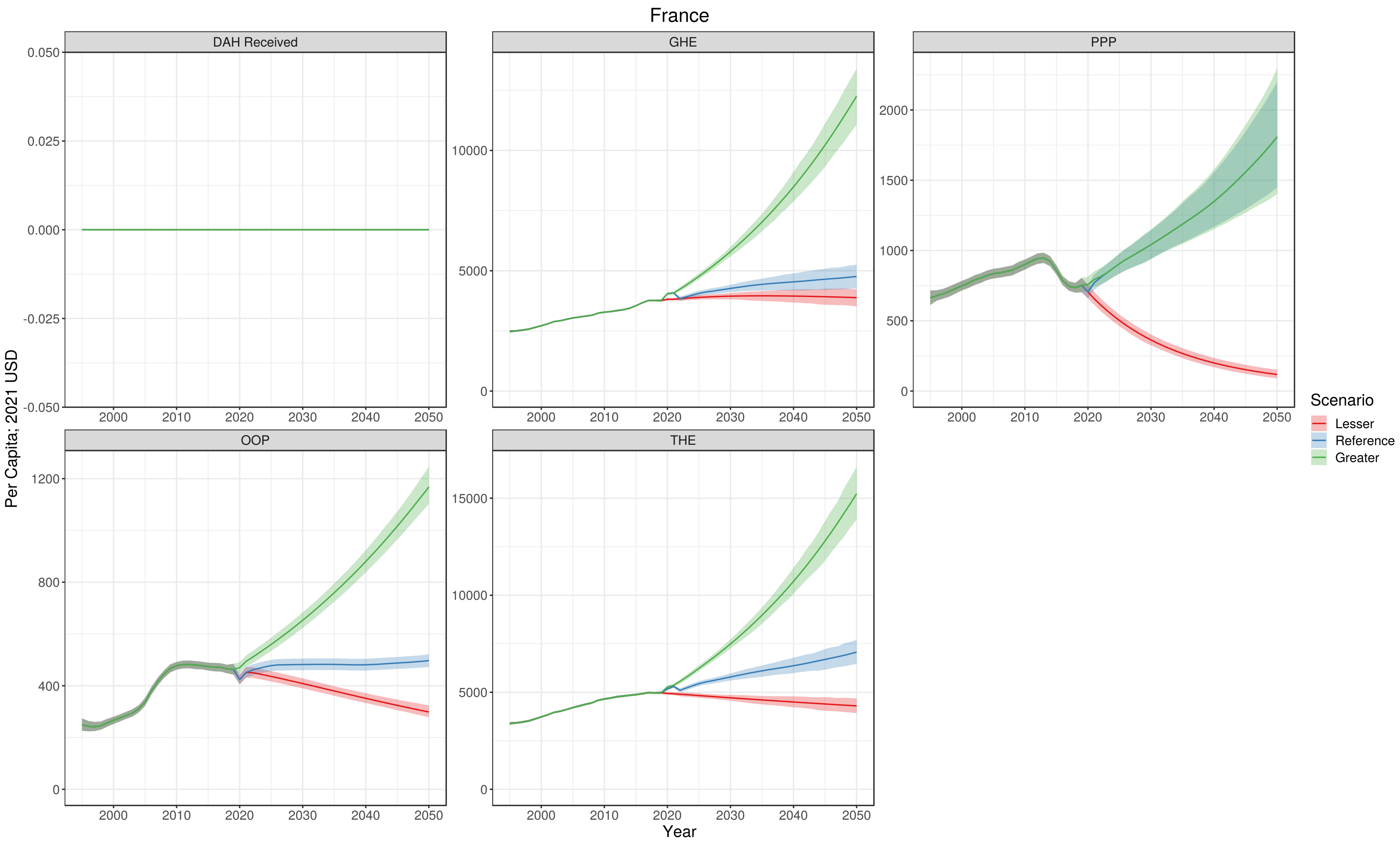

France
DAH Received
GHE
PPP
OOP
THE
0.050
0.025
0.000
-0.025
-0.050
10000
5000
0
2000
1500
1000
500
0
1200
800
400
0
15000
10000
5000
0
2000
2010
2020
2030
2040
2050
Per Capita; 2021 USD
Year
Scenario
Lesser
Reference
Greater

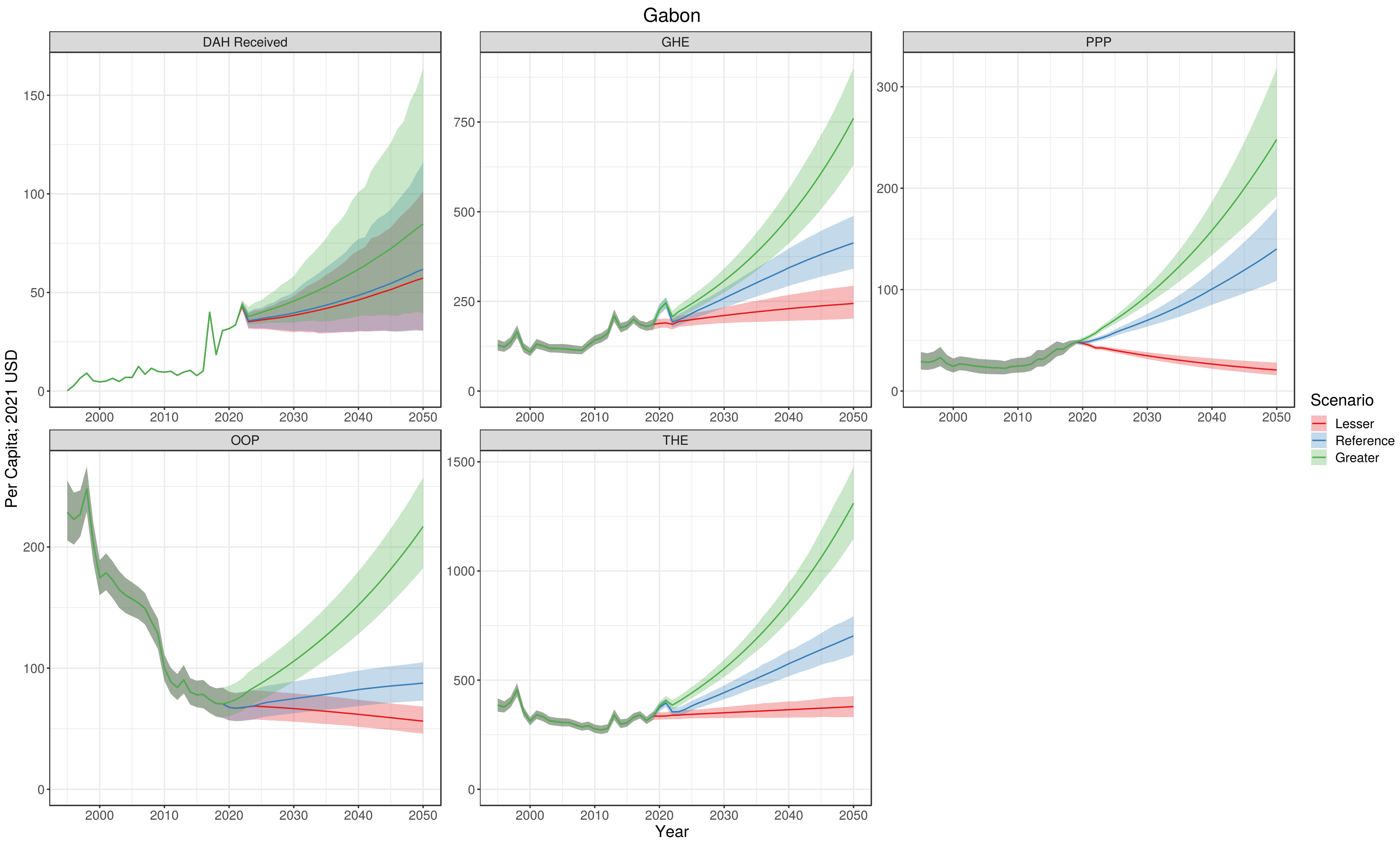

Gabon
DAH Received
GHE
PPP
OOP
THE
Per Capita; 2021 USD
Year
Scenario
Lesser
Reference
Greater

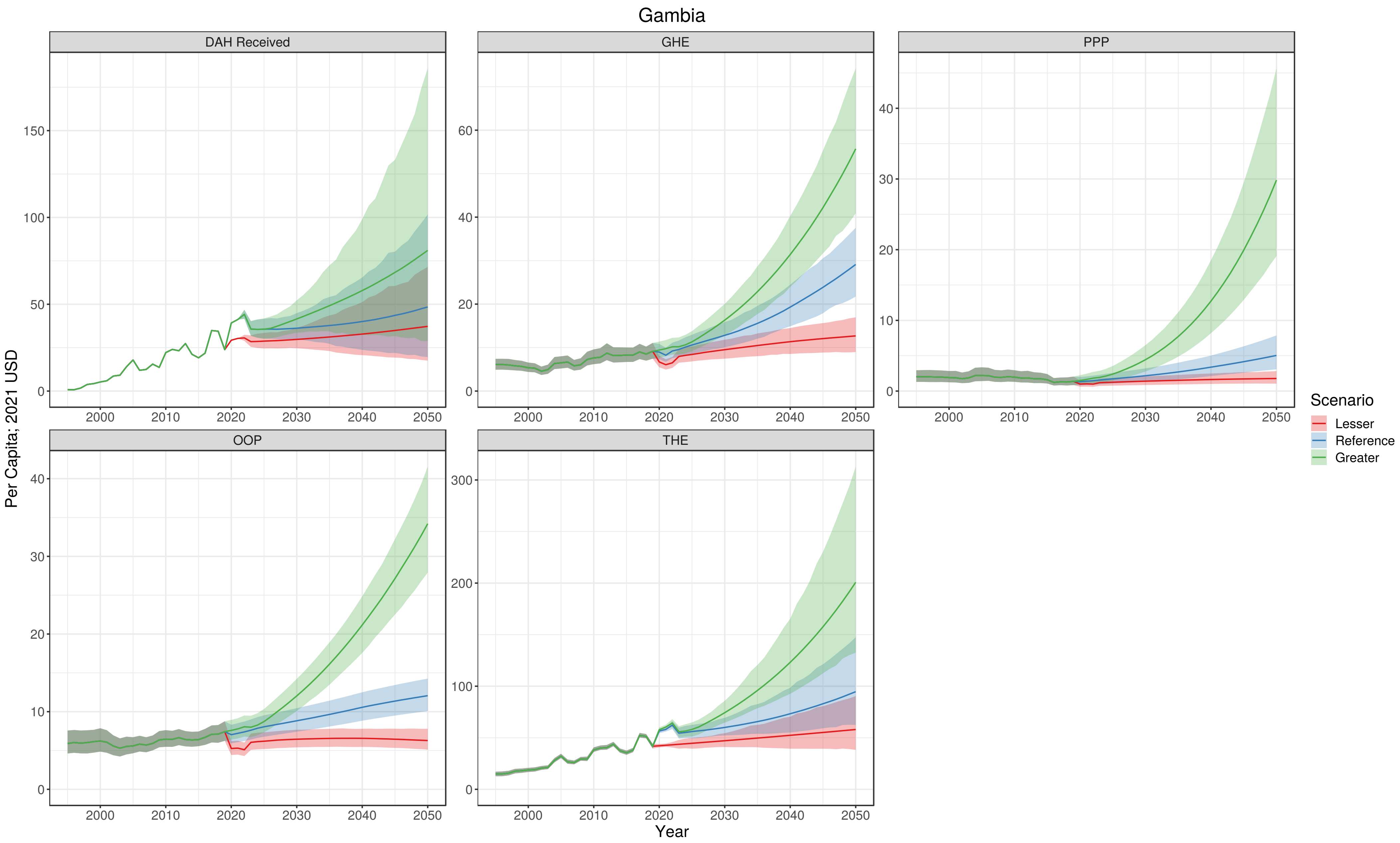

Gambia
DAH Received
GHE
PPP
OOP
THE
Per Capita; 2021 USD
Year
Scenario
Lesser
Reference
Greater

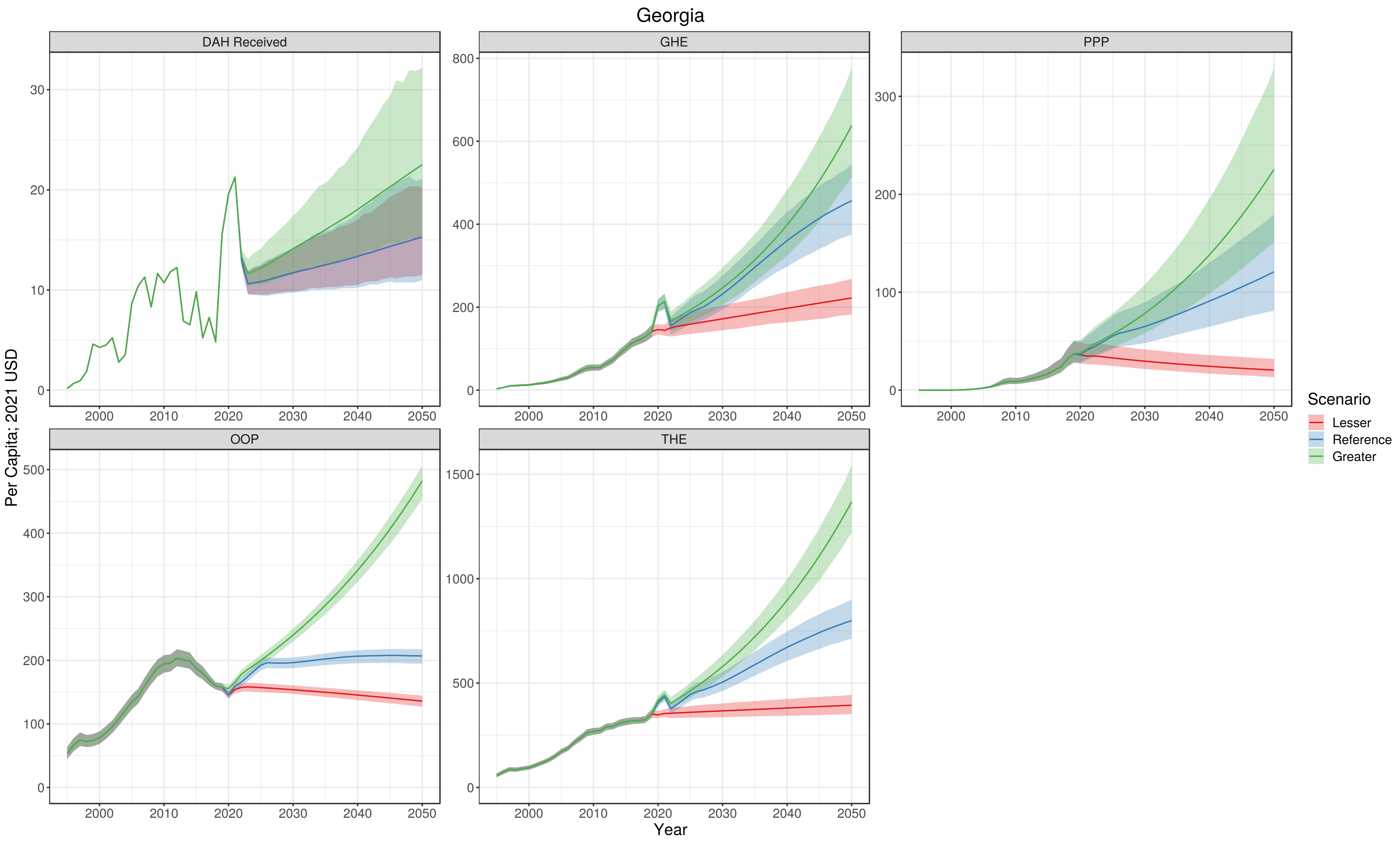

Georgia
DAH Received
GHE
PPP
OOP
THE
Per Capita; 2021 USD
Year
Scenario
Lesser
Reference
Greater

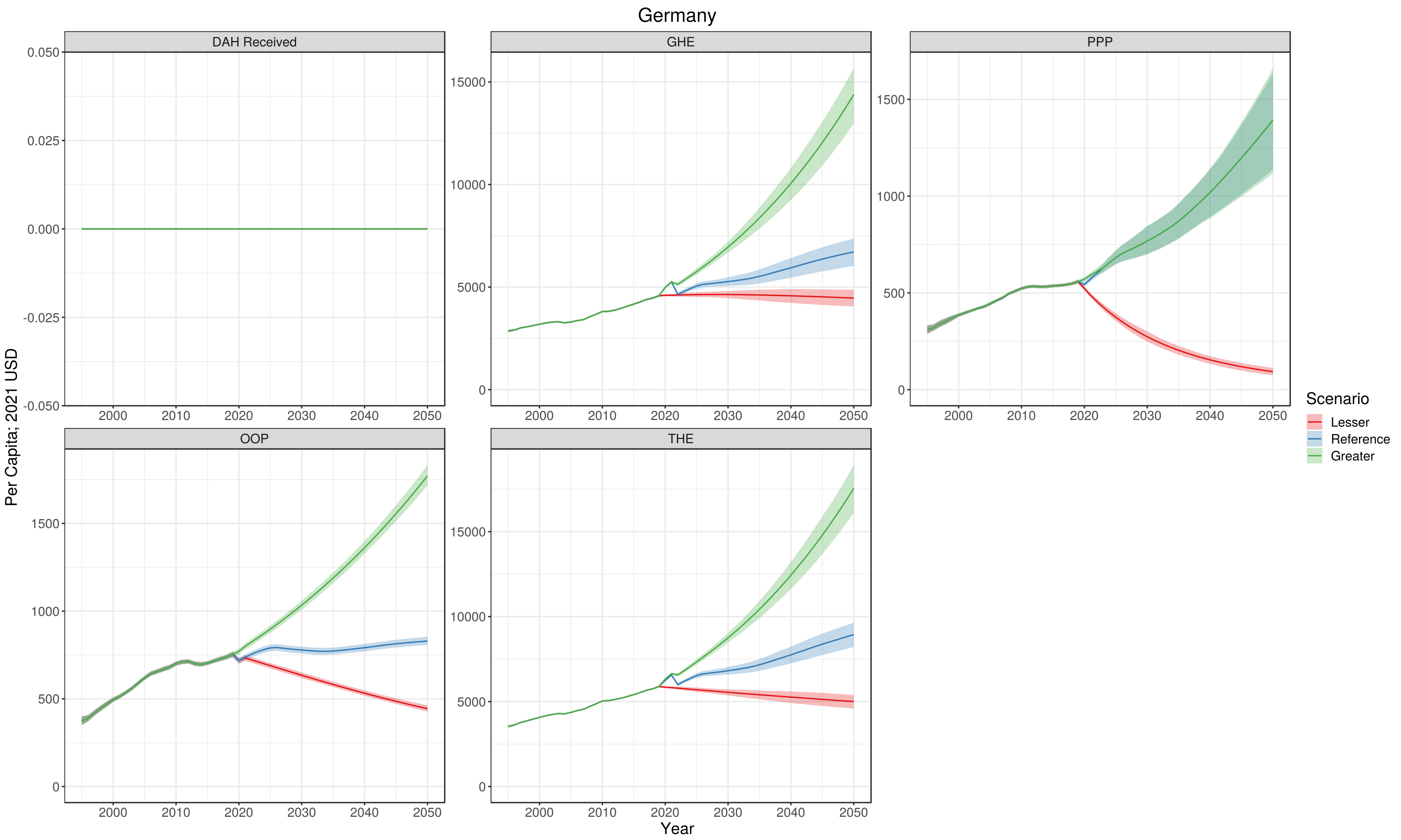

Germany
DAH Received
GHE
PPP
OOP
THE
Per Capita; 2021 USD
Year
Scenario
Lesser
Reference
Greater
0.050
0.025
0.000
-0.025
-0.050
15000
10000
5000
0
1500
1000
500
2000
2010
2020
2030
2040
2050

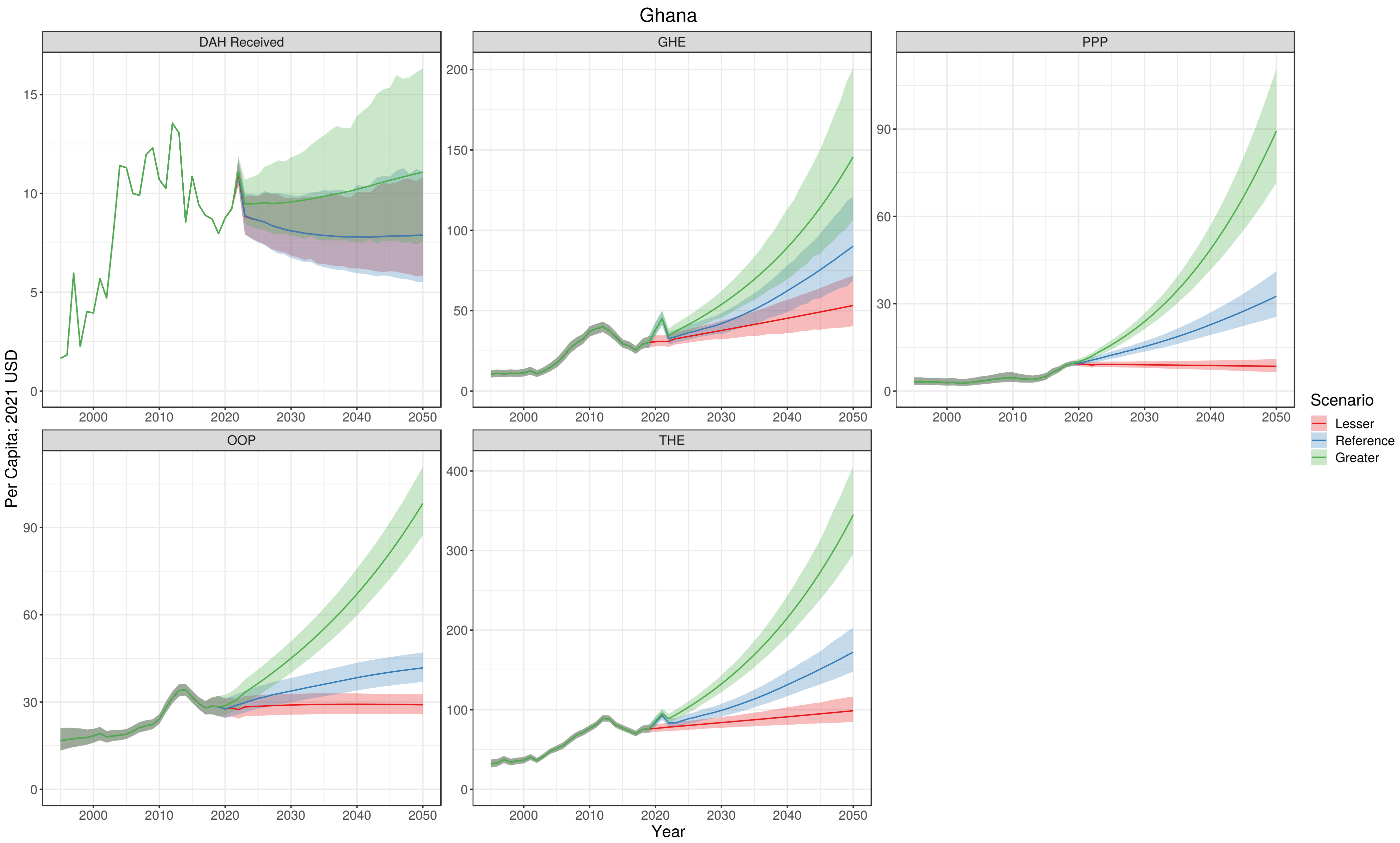

Ghana
DAH Received
GHE
PPP
OOP
THE
Per Capita; 2021 USD
Year
Scenario
Lesser
Reference
Greater

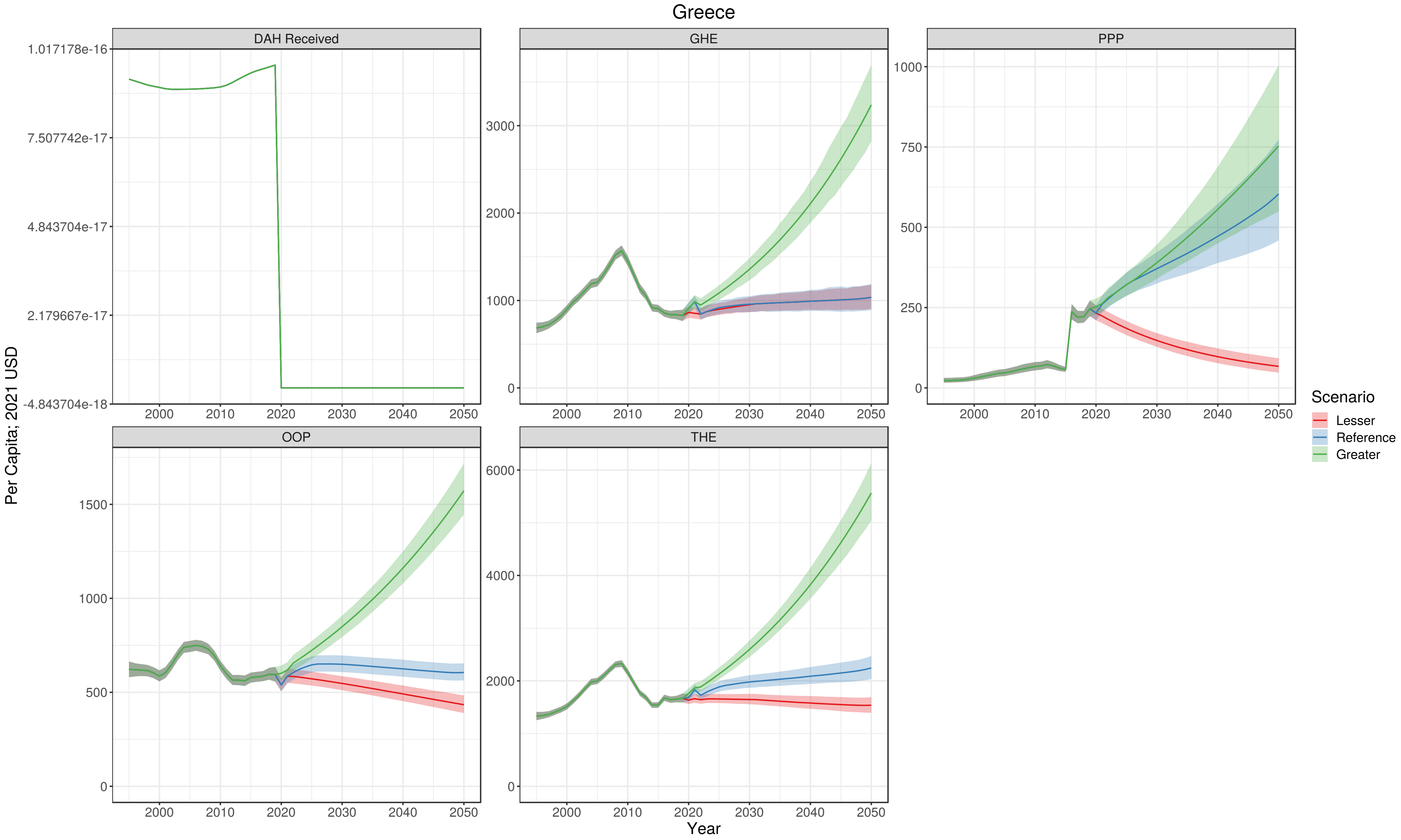

Greece
DAH Received
GHE
PPP
OOP
THE
1.017178e-16
7.507742e-17
4.843704e-17
2.179667e-17
-4.843704e-18
3000
2000
1000
0
1000
750
500
250
0
1500
1000
500
0
6000
4000
2000
0
2000
2010
2020
2030
2040
2050
Per Capita; 2021 USD
Year
Scenario
Lesser
Reference
Greater

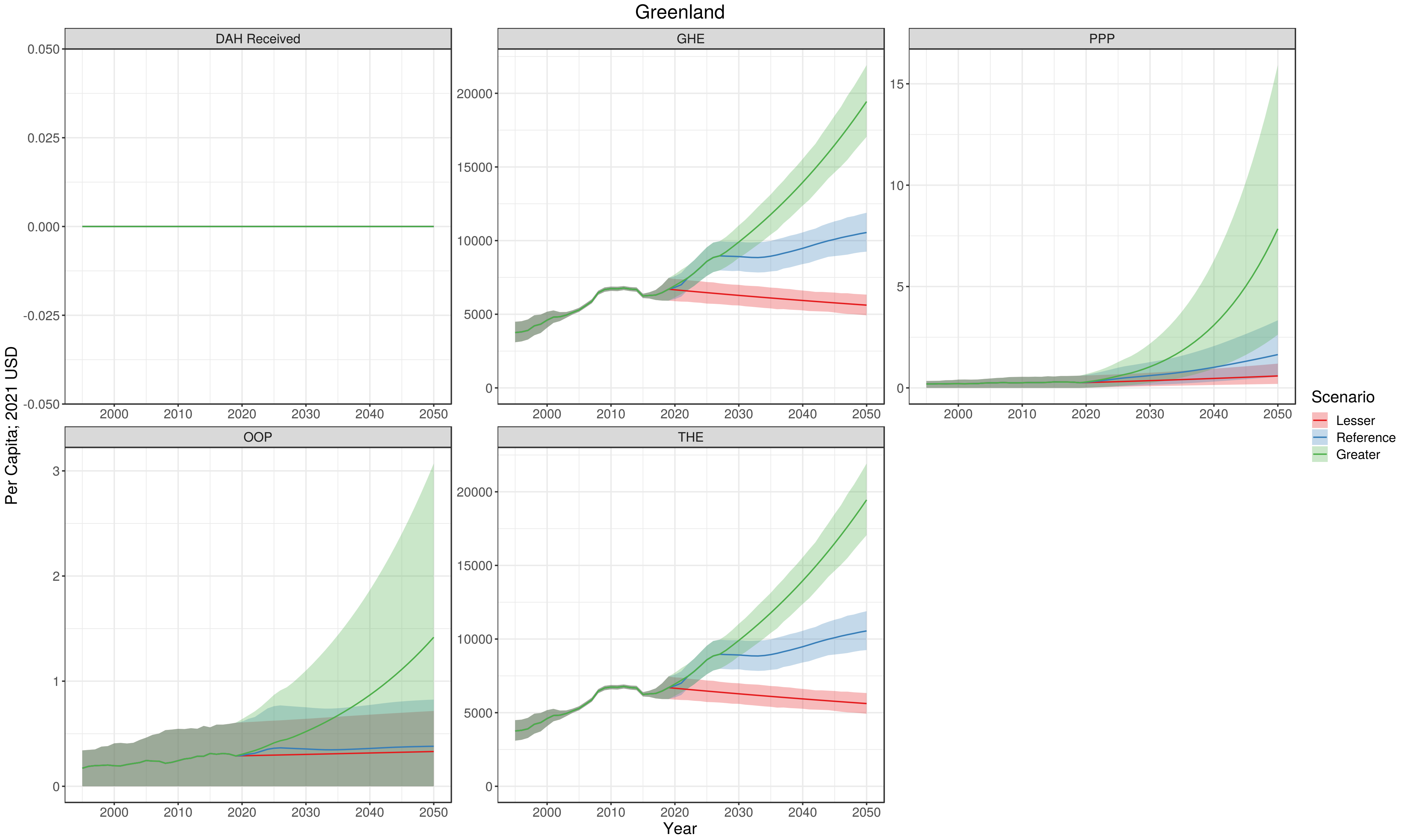

Greenland
DAH Received
GHE
PPP
OOP
THE
Per Capita; 2021 USD
Year
Scenario
Lesser
Reference
Greater
0.050
0.025
0.000
-0.025
-0.050
20000
15000
10000
5000
0
15
10
5
3
2
1
2000
2010
2020
2030
2040
2050

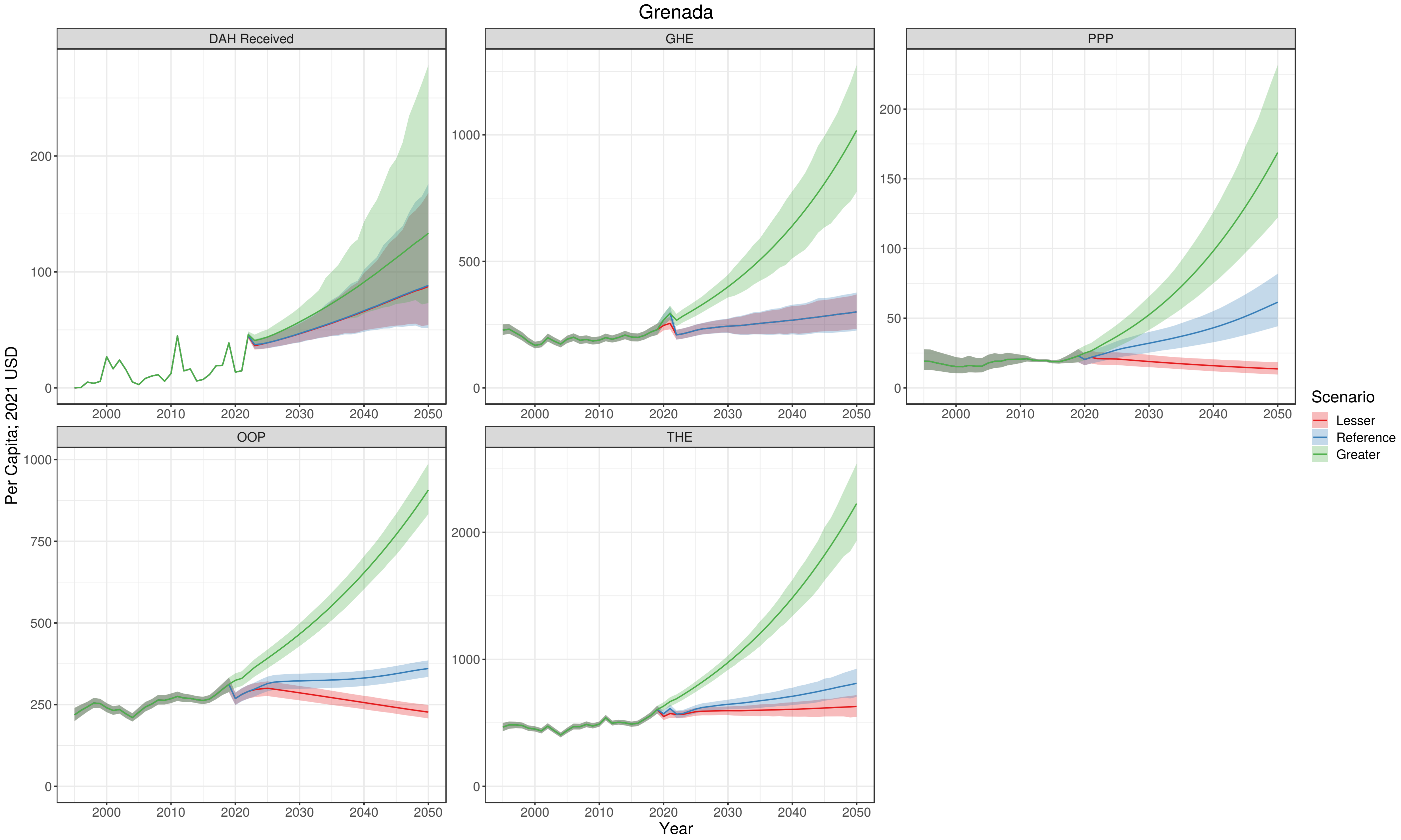

Grenada
DAH Received
GHE
PPP
OOP
THE
Per Capita; 2021 USD
Year
Scenario
Lesser
Reference
Greater

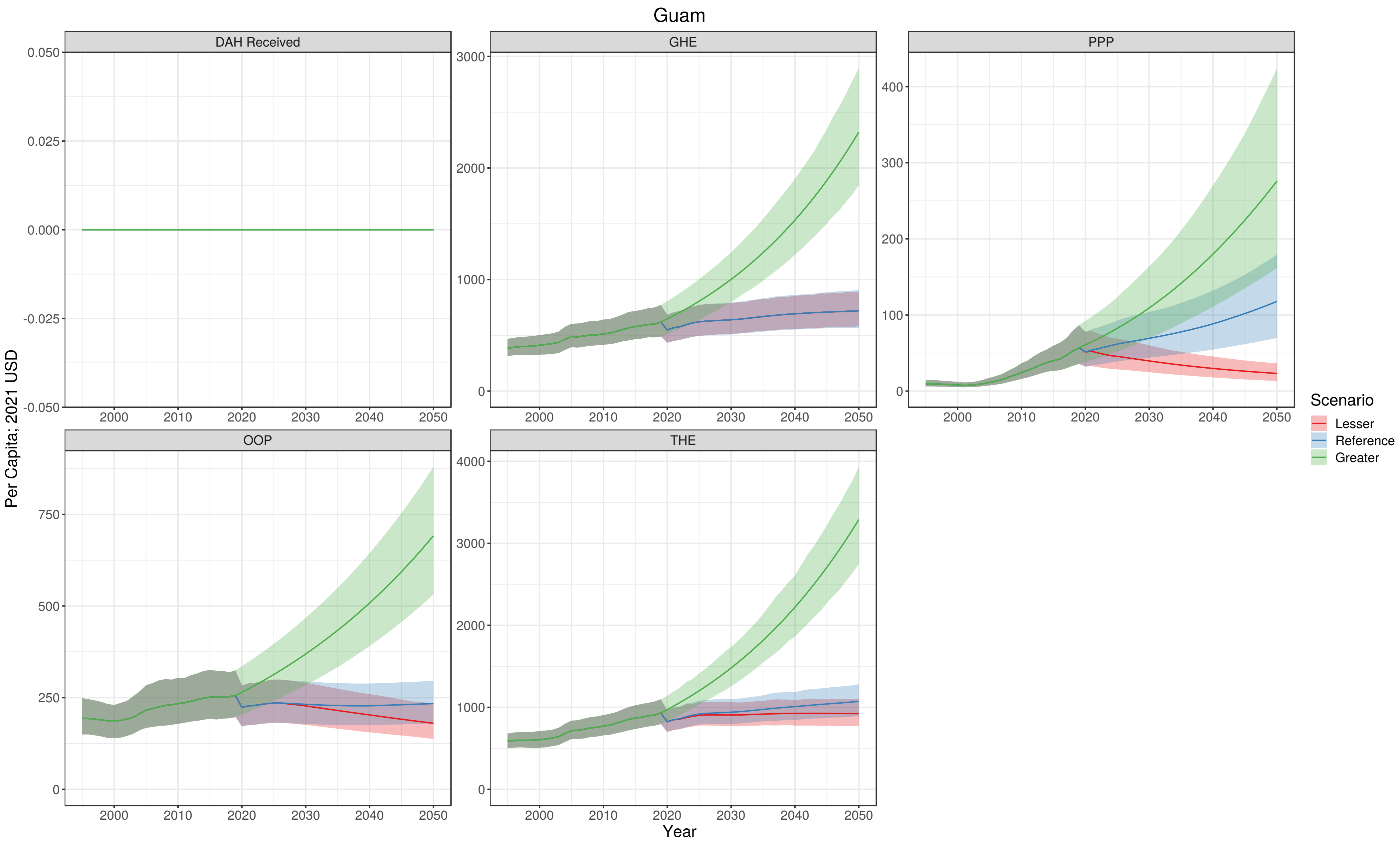

Guam
DAH Received
GHE
PPP
OOP
THE
Per Capita; 2021 USD
Year
Scenario
Lesser
Reference
Greater

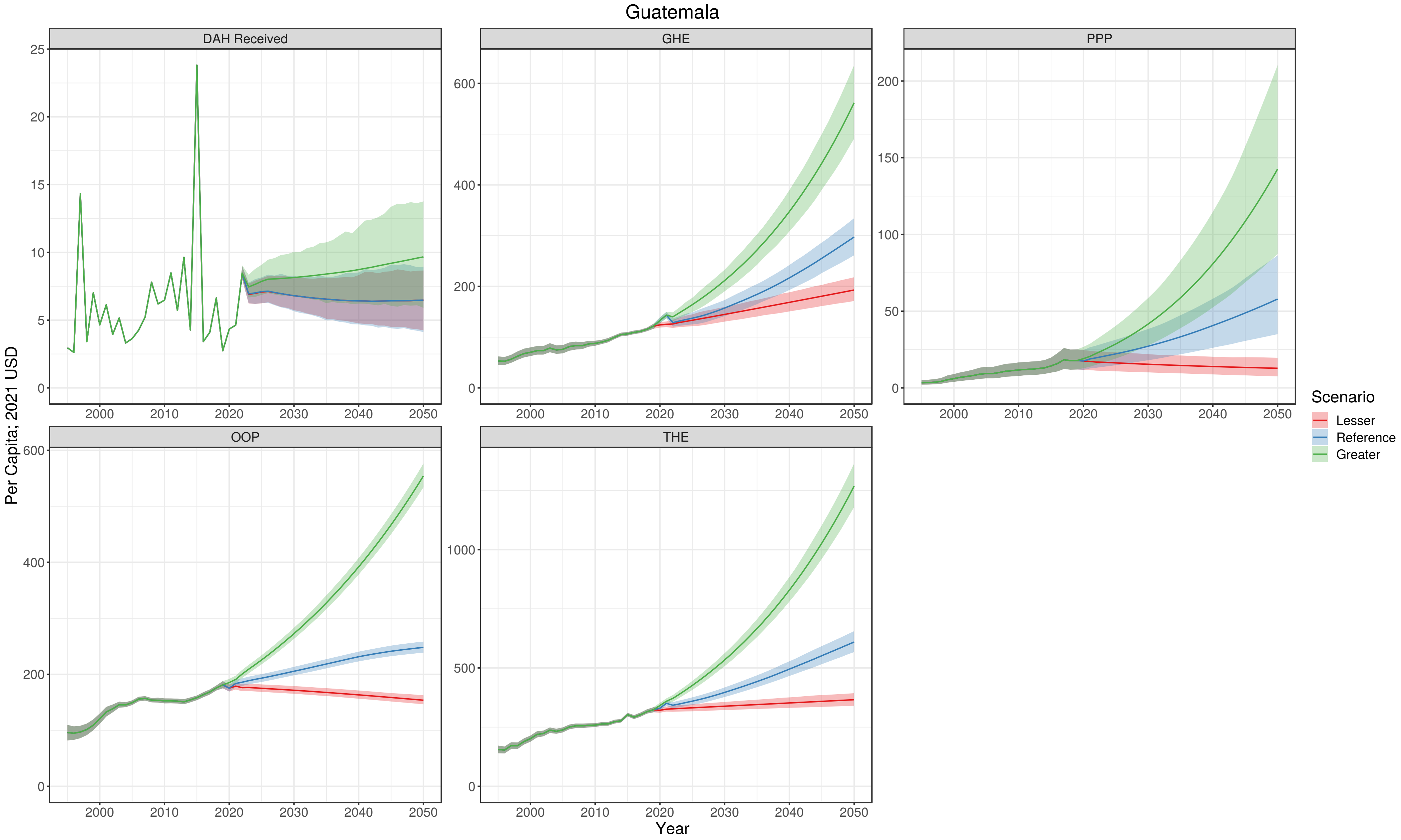
Guatemala
DAH Received
GHE
PPP
OOP
THE
Per Capita; 2021 USD
Year
Scenario
Lesser
Reference
Greater
0
5
10
15
20
25
200
400
600
1000
500
50
100
150
2000
2010
2020
2030
2040
2050

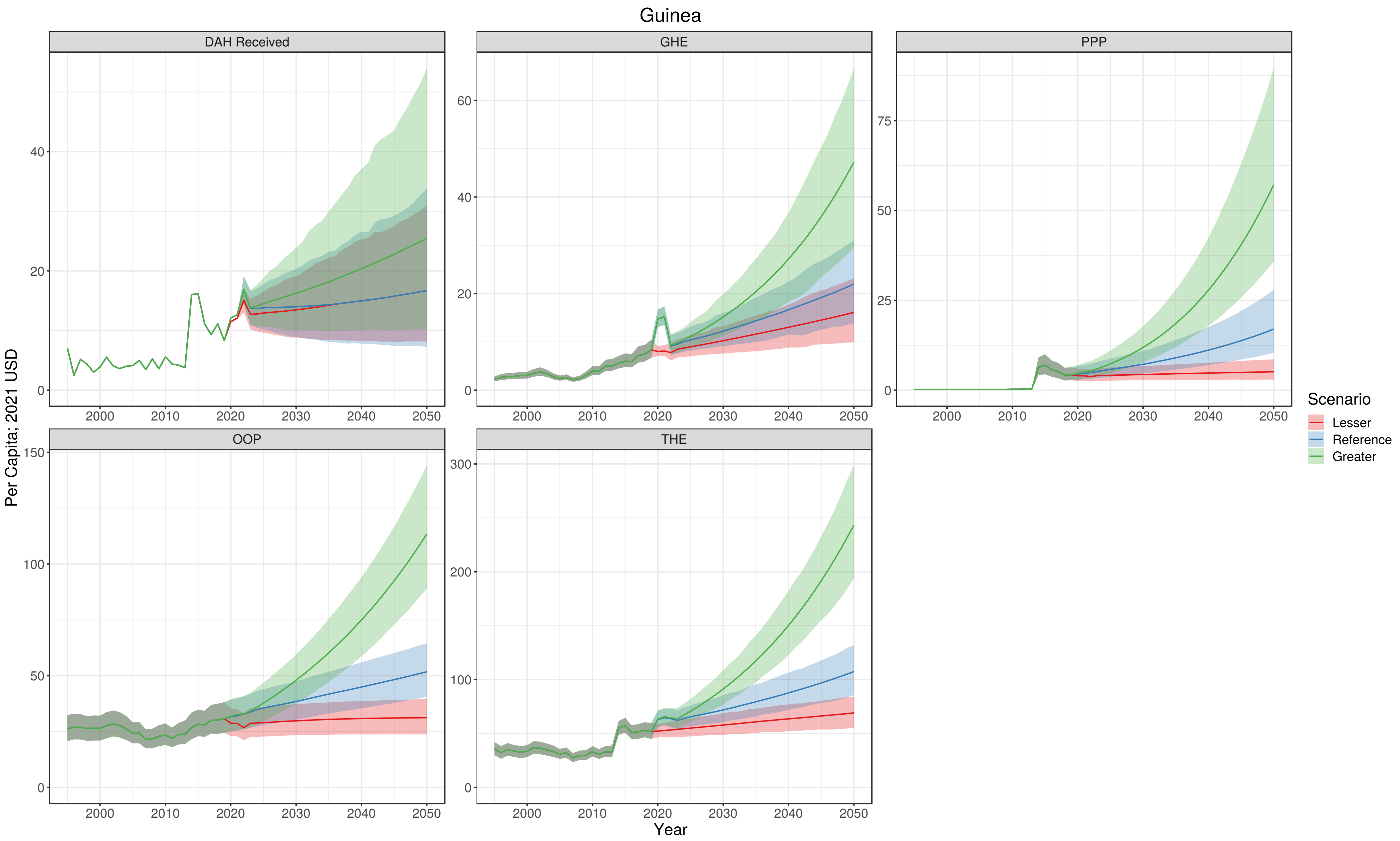

Guinea
DAH Received
GHE
PPP
OOP
THE
Per Capita; 2021 USD
Year
Scenario
Lesser
Reference
Greater
0
20
40
60
25
50
75
100
150
200
300
2000
2010
2020
2030
2040
2050

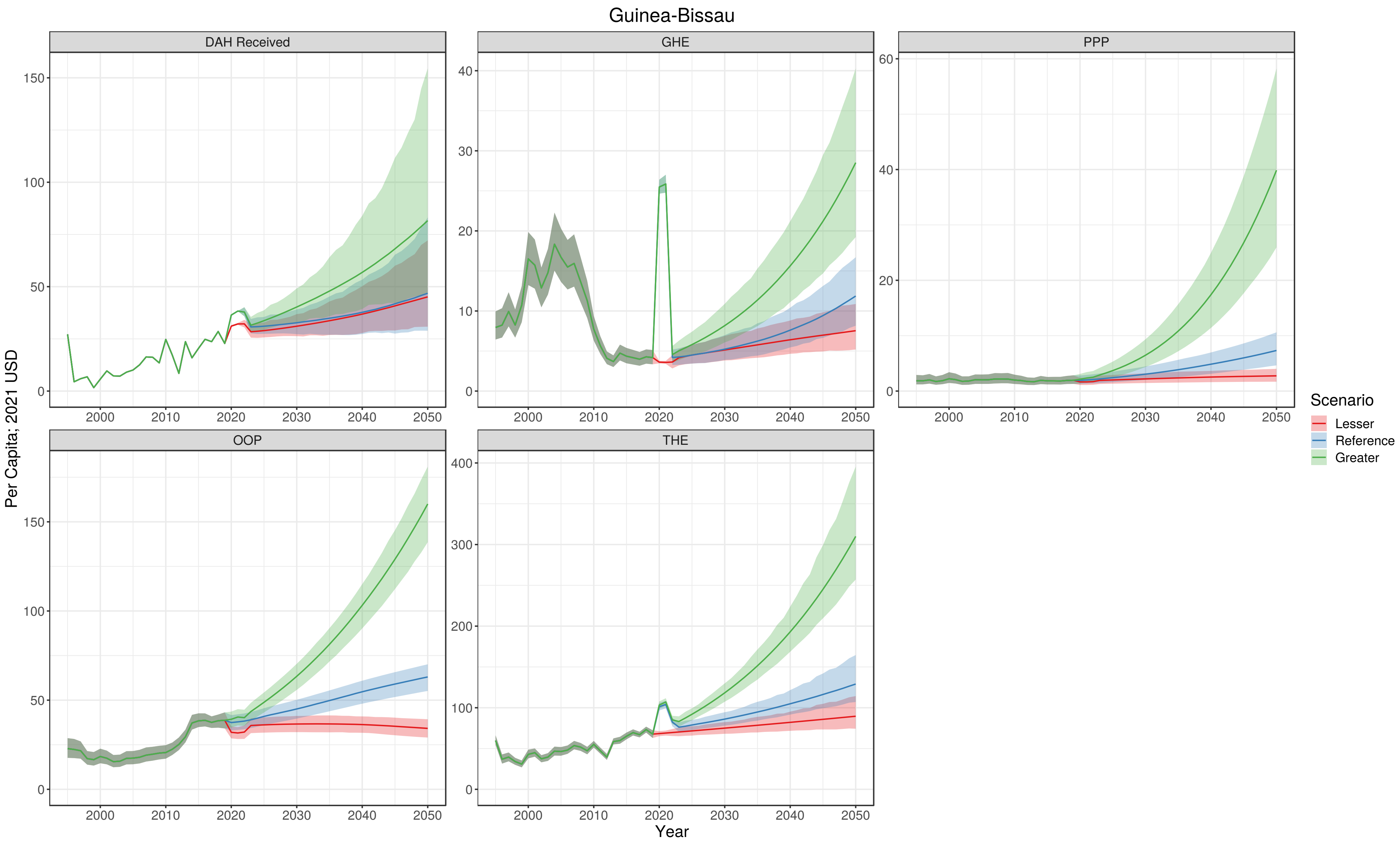
Guinea-Bissau
DAH Received
GHE
PPP
OOP
THE
Per Capita; 2021 USD
Year
Scenario
Lesser
Reference
Greater
0
50
100
150
0
10
20
30
40
0
20
40
60
0
50
100
150
0
100
200
300
400
2000
2010
2020
2030
2040
2050

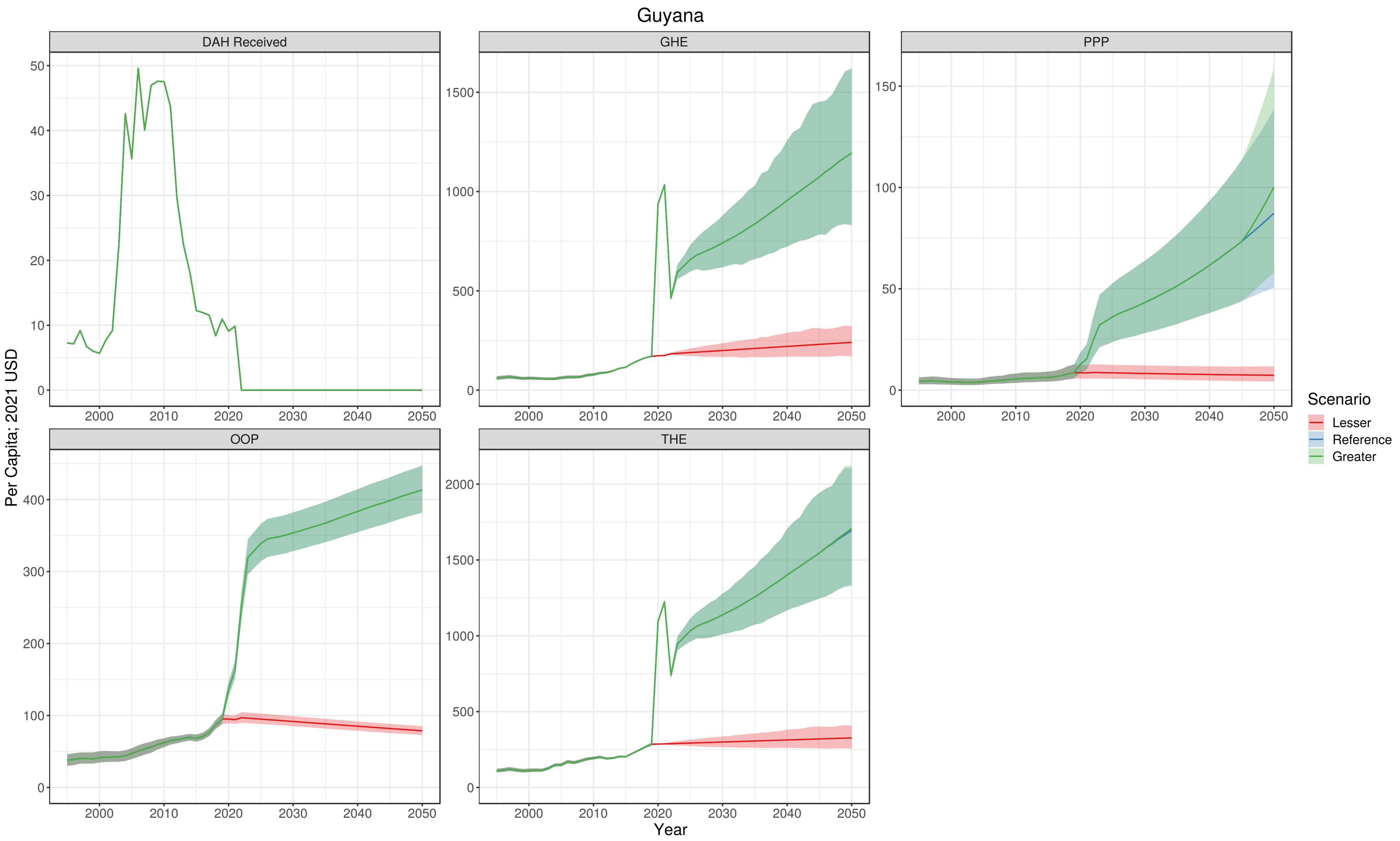

Guyana
DAH Received
GHE
PPP
OOP
THE
Per Capita; 2021 USD
Year
Scenario
Lesser
Reference
Greater

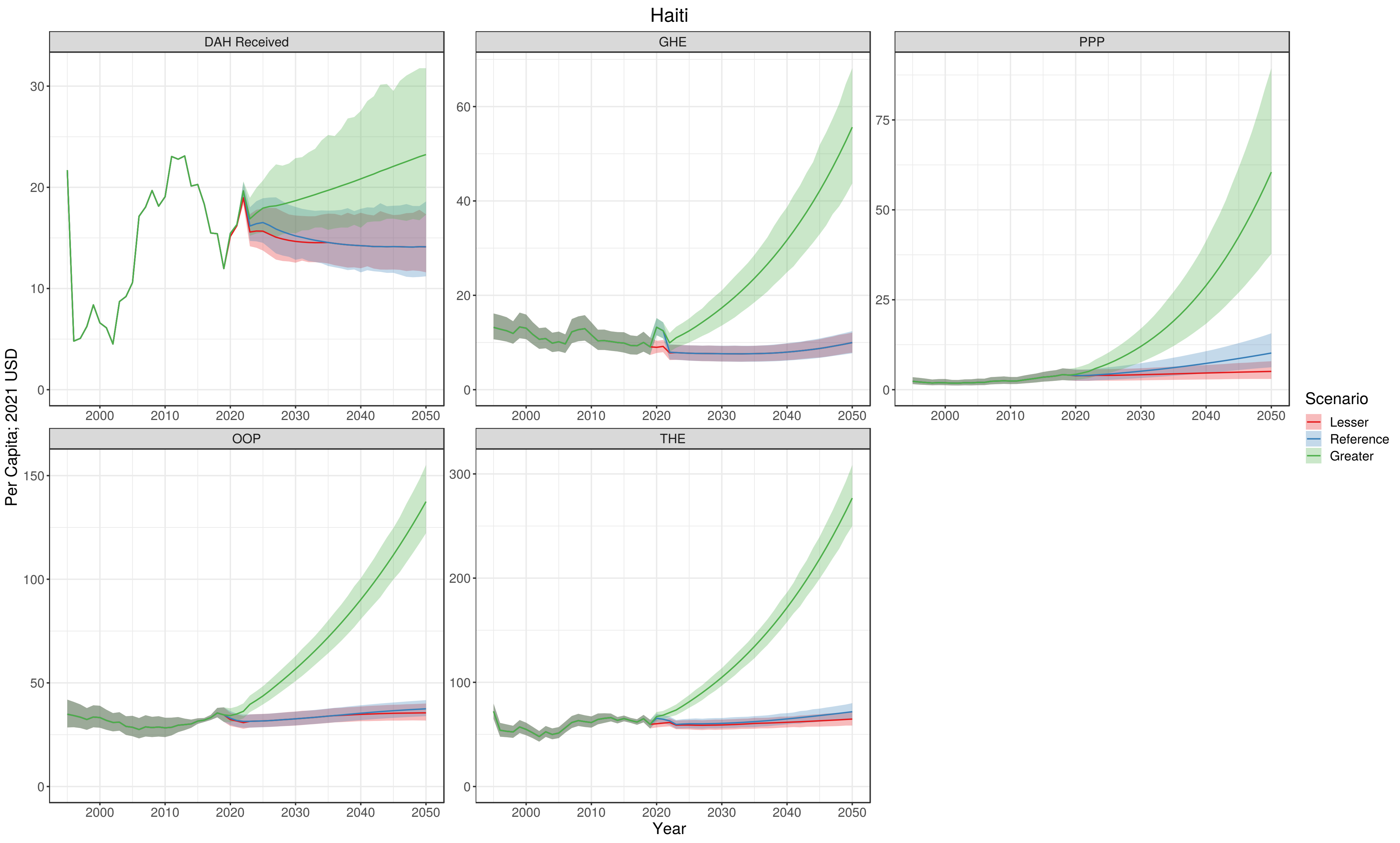
Haiti
DAH Received
GHE
PPP
OOP
THE
Per Capita; 2021 USD
Year
Scenario
Lesser
Reference
Greater
0
10
20
30
0
20
40
60
0
25
50
75
0
50
100
150
0
100
200
300
2000
2010
2020
2030
2040
2050

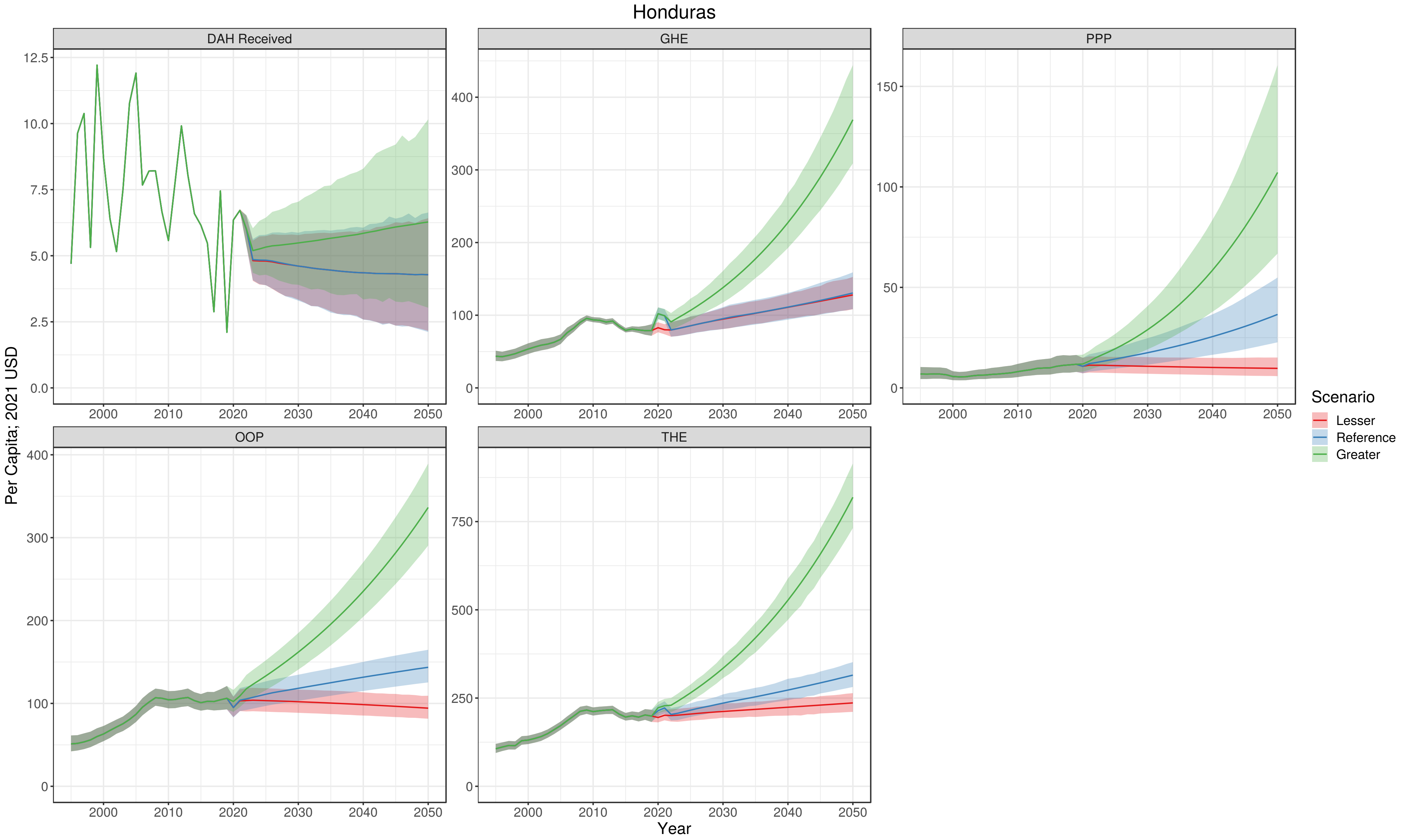

Honduras
DAH Received
GHE
PPP
OOP
THE
Per Capita; 2021 USD
Year
Scenario
Lesser
Reference
Greater

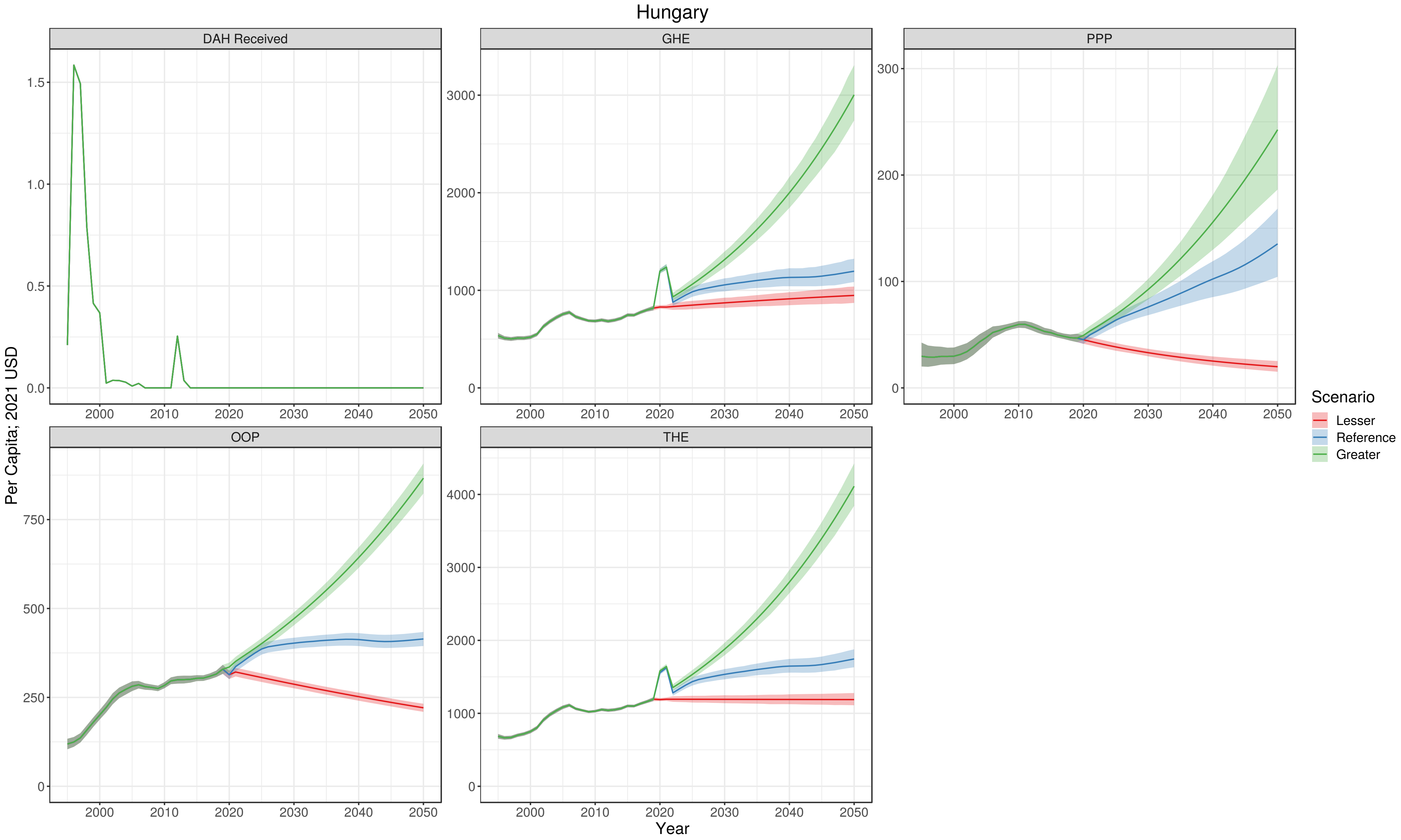
Hungary
DAH Received
GHE
PPP
OOP
THE
Per Capita; 2021 USD
Year
Scenario
Lesser
Reference
Greater
1.5
1.0
0.5
0.0
3000
2000
1000
0
300
200
100
750
500
250
4000
2000
2010
2020
2030
2040
2050

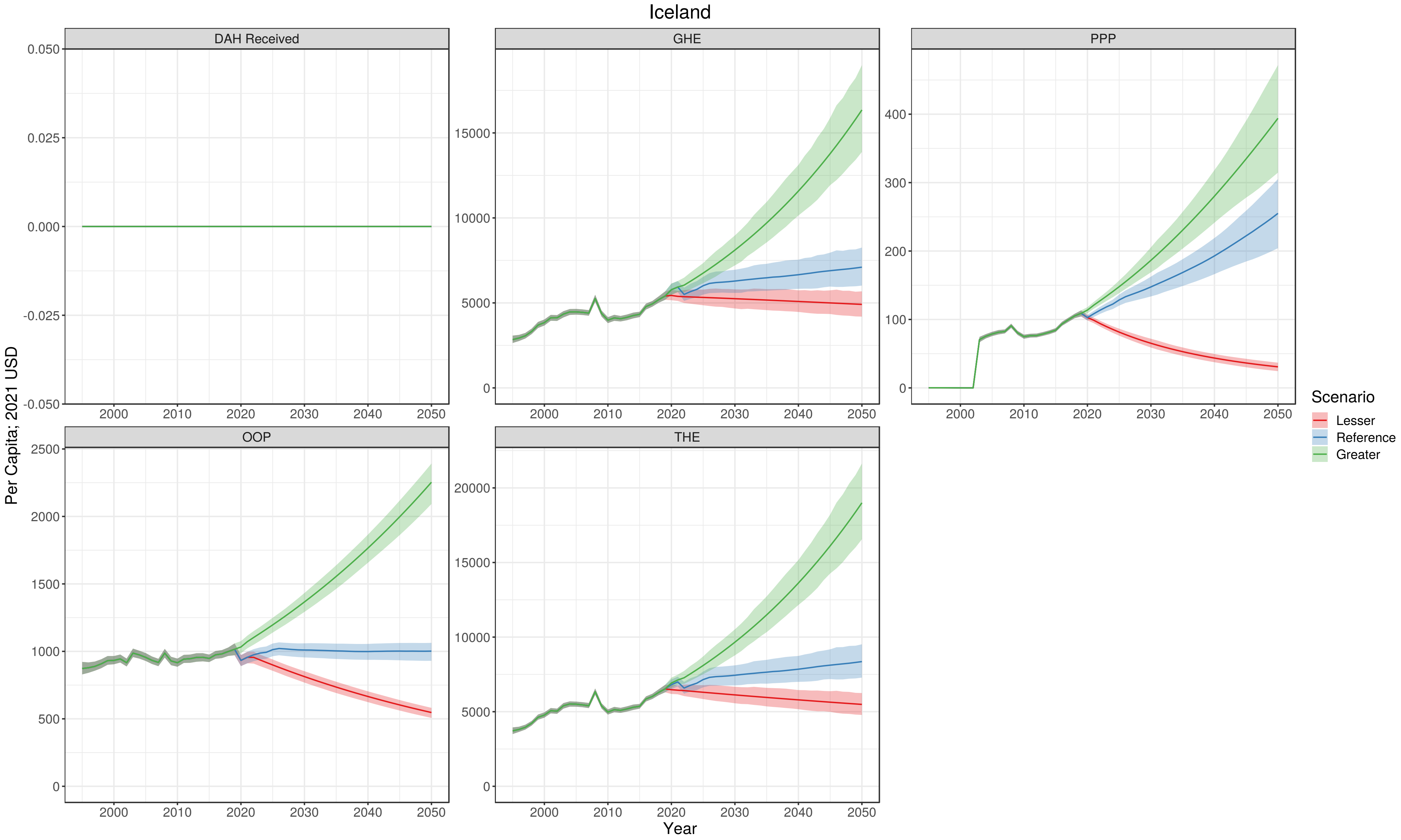
Iceland
DAH Received
GHE
PPP
OOP
THE
Per Capita; 2021 USD
Year
Scenario
Lesser
Reference
Greater
0.050
0.025
0.000
-0.025
-0.050
15000
10000
5000
0
400
300
200
100
2500
2000
1500
1000
500
20000
2000
2010
2020
2030
2040
2050

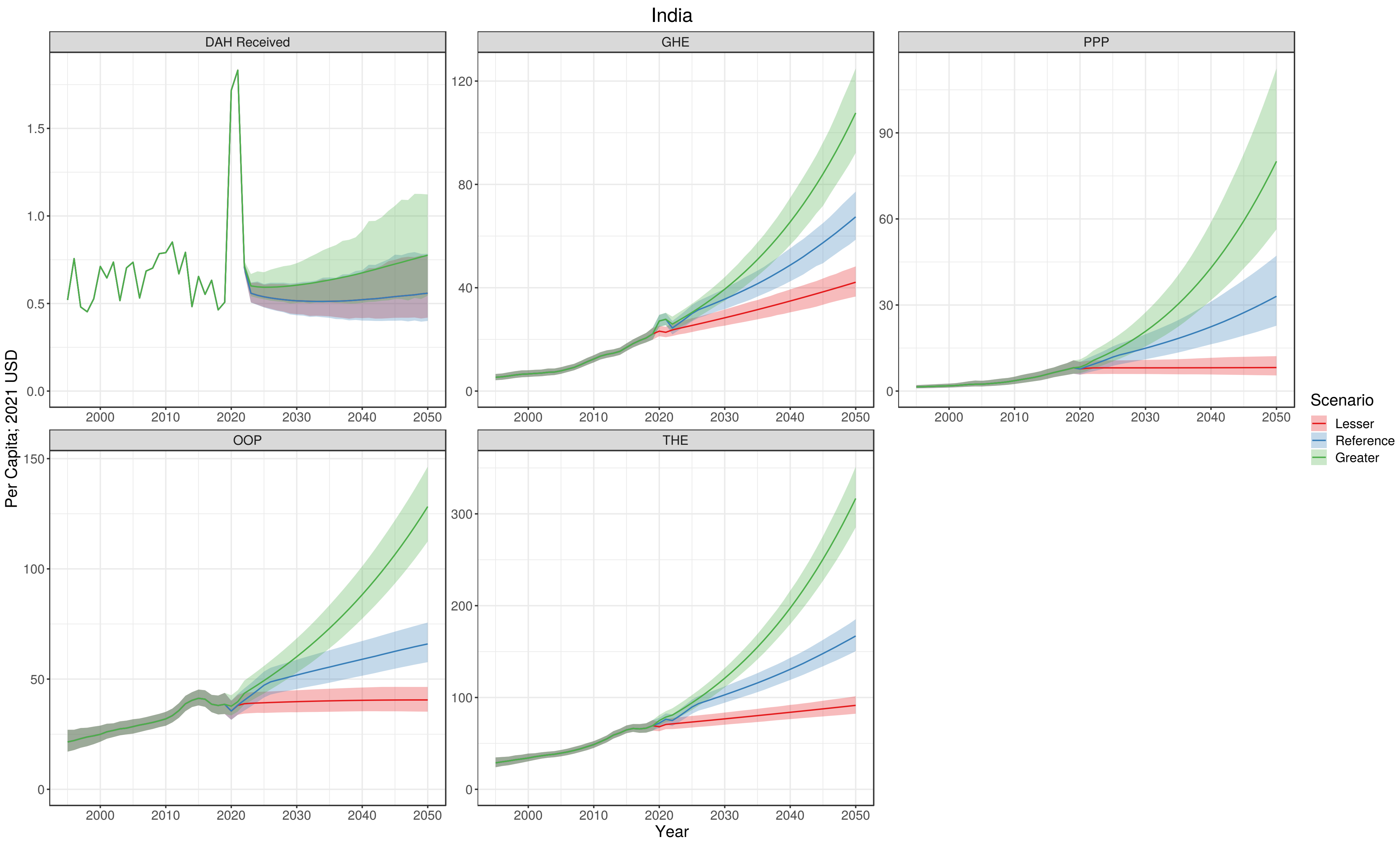
India
DAH Received
GHE
PPP
OOP
THE
Per Capita; 2021 USD
Year
Scenario
Lesser
Reference
Greater
0.0
0.5
1.0
1.5
0
40
80
120
0
30
60
90
0
50
100
150
0
100
200
300
2000
2010
2020
2030
2040
2050

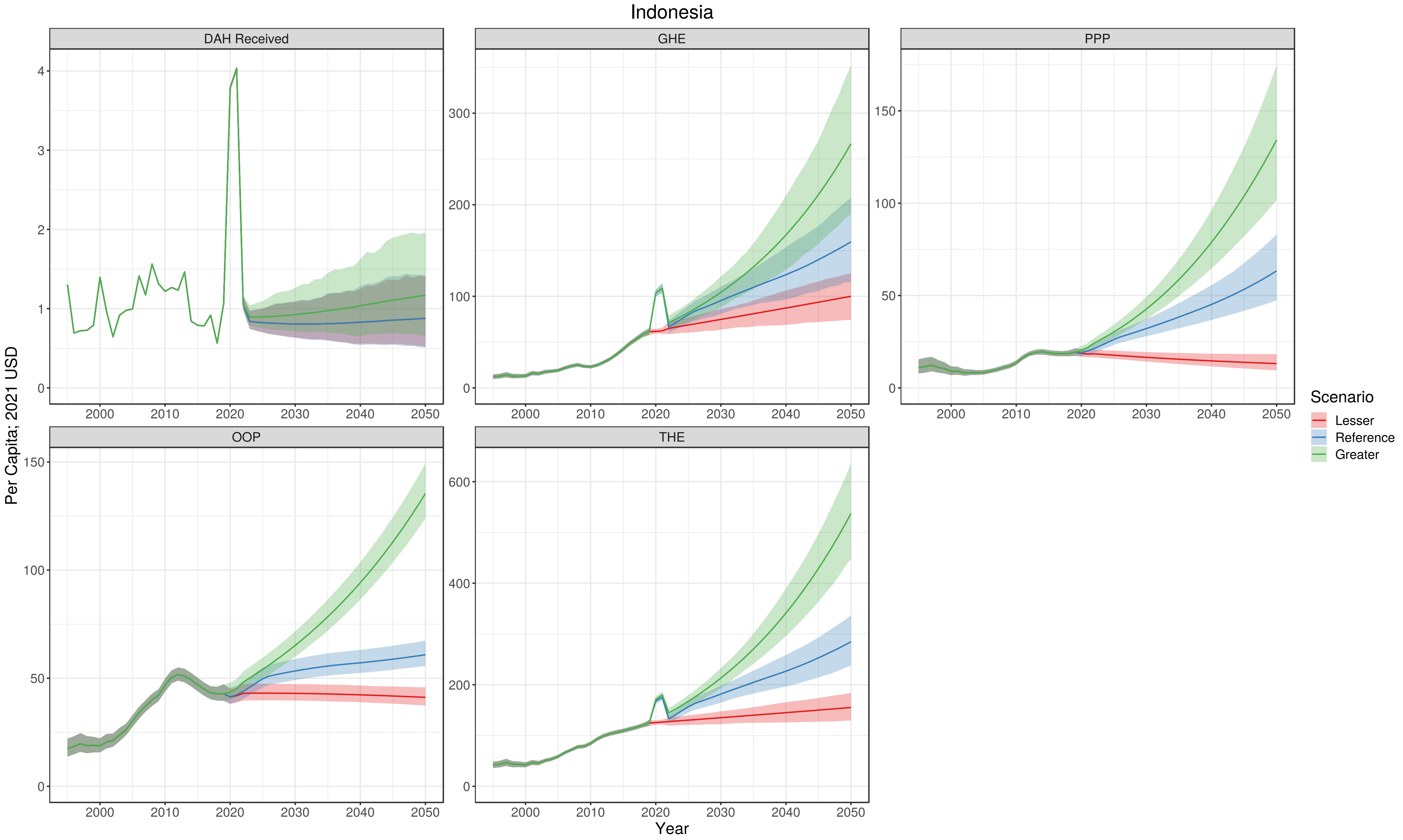

Indonesia
DAH Received
GHE
PPP
OOP
THE
Per Capita; 2021 USD
Year
Scenario
Lesser
Reference
Greater

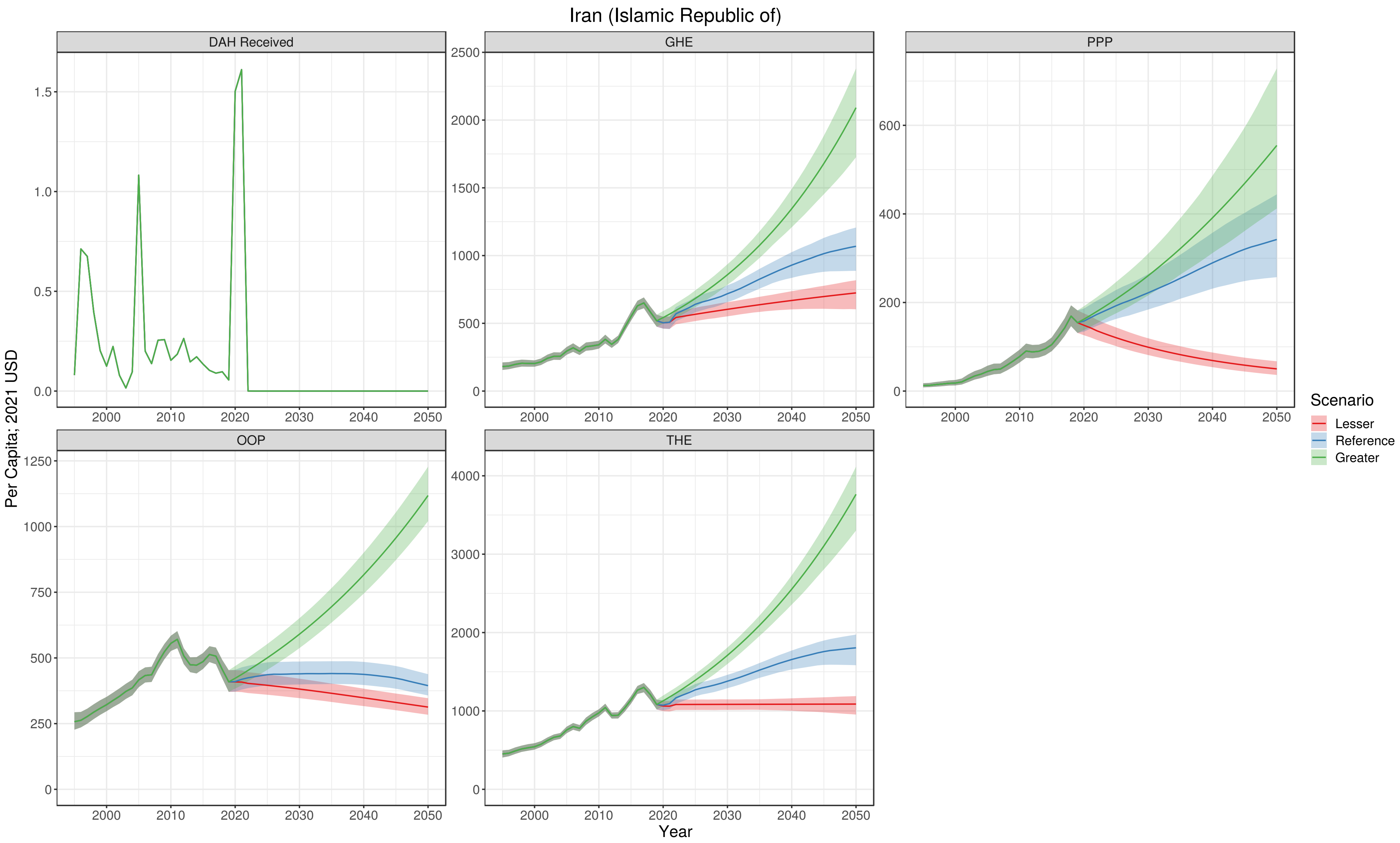

Iran (Islamic Republic of)
DAH Received
GHE
PPP
OOP
THE
Per Capita; 2021 USD
Year
Scenario
Lesser
Reference
Greater

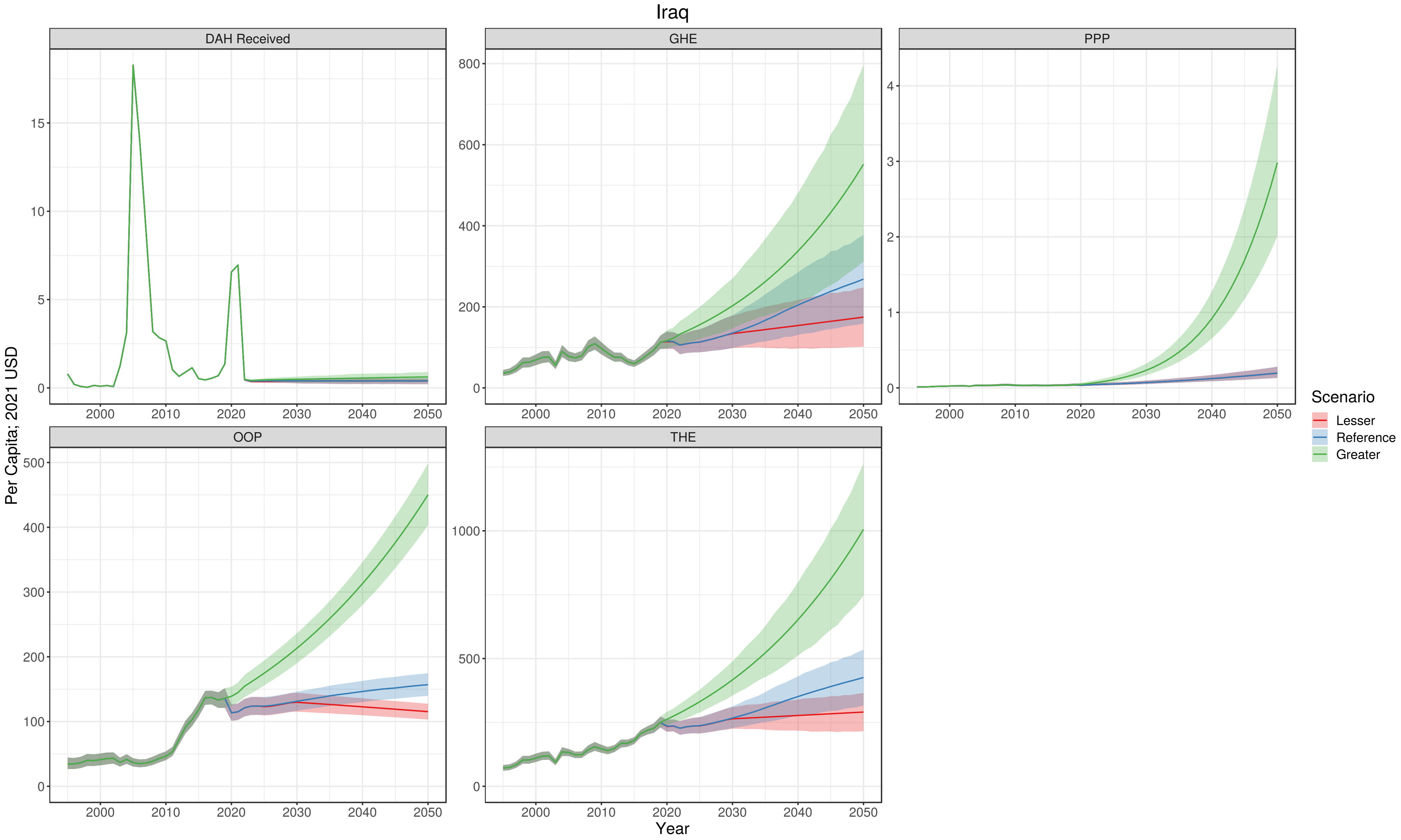
Iraq
DAH Received
GHE
PPP
OOP
THE
Per Capita; 2021 USD
Year
Scenario
Lesser
Reference
Greater

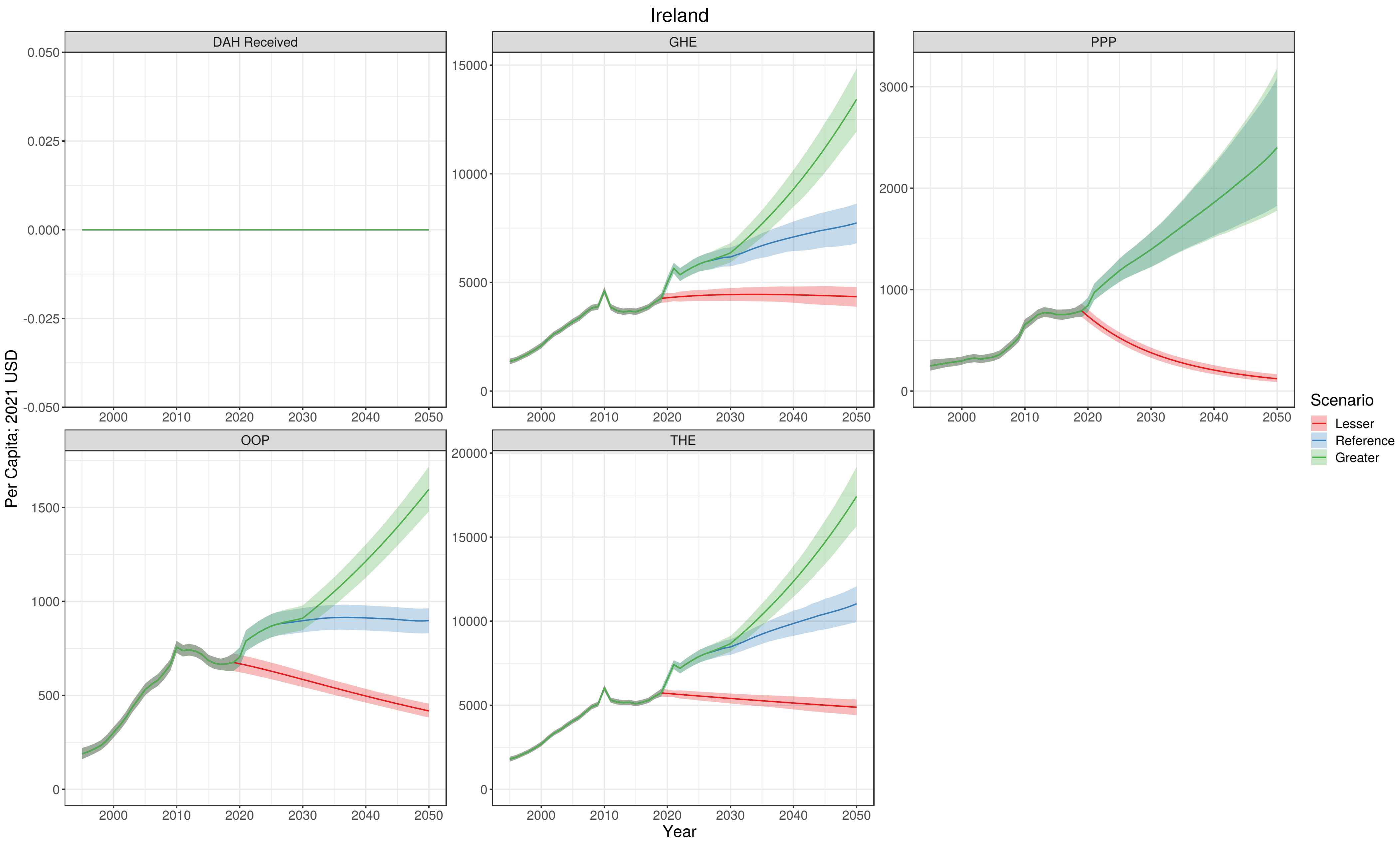
Ireland
DAH Received
GHE
PPP
OOP
THE
Per Capita; 2021 USD
Year
Scenario
Lesser
Reference
Greater
0.050
0.025
0.000
-0.025
-0.050
15000
10000
5000
0
3000
2000
1000
1500
1000
500
20000
2000
2010
2020
2030
2040
2050

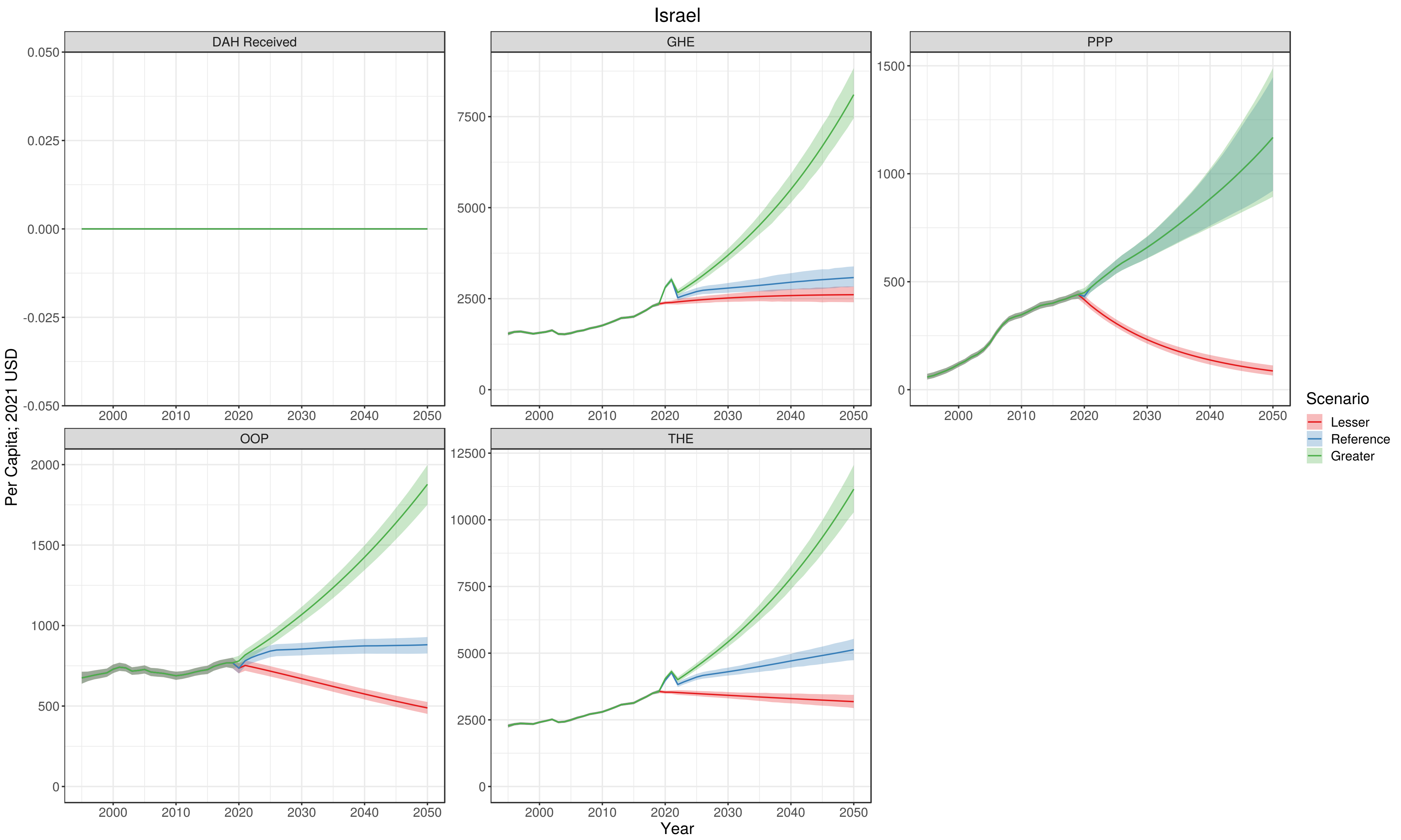

Israel
DAH Received
GHE
PPP
OOP
THE
0.050
0.025
0.000
-0.025
-0.050
7500
5000
2500
0
1500
1000
500
2000
1500
1000
500
12500
10000
2000
2010
2020
2030
2040
2050
Per Capita; 2021 USD
Year
Scenario
Lesser
Reference
Greater

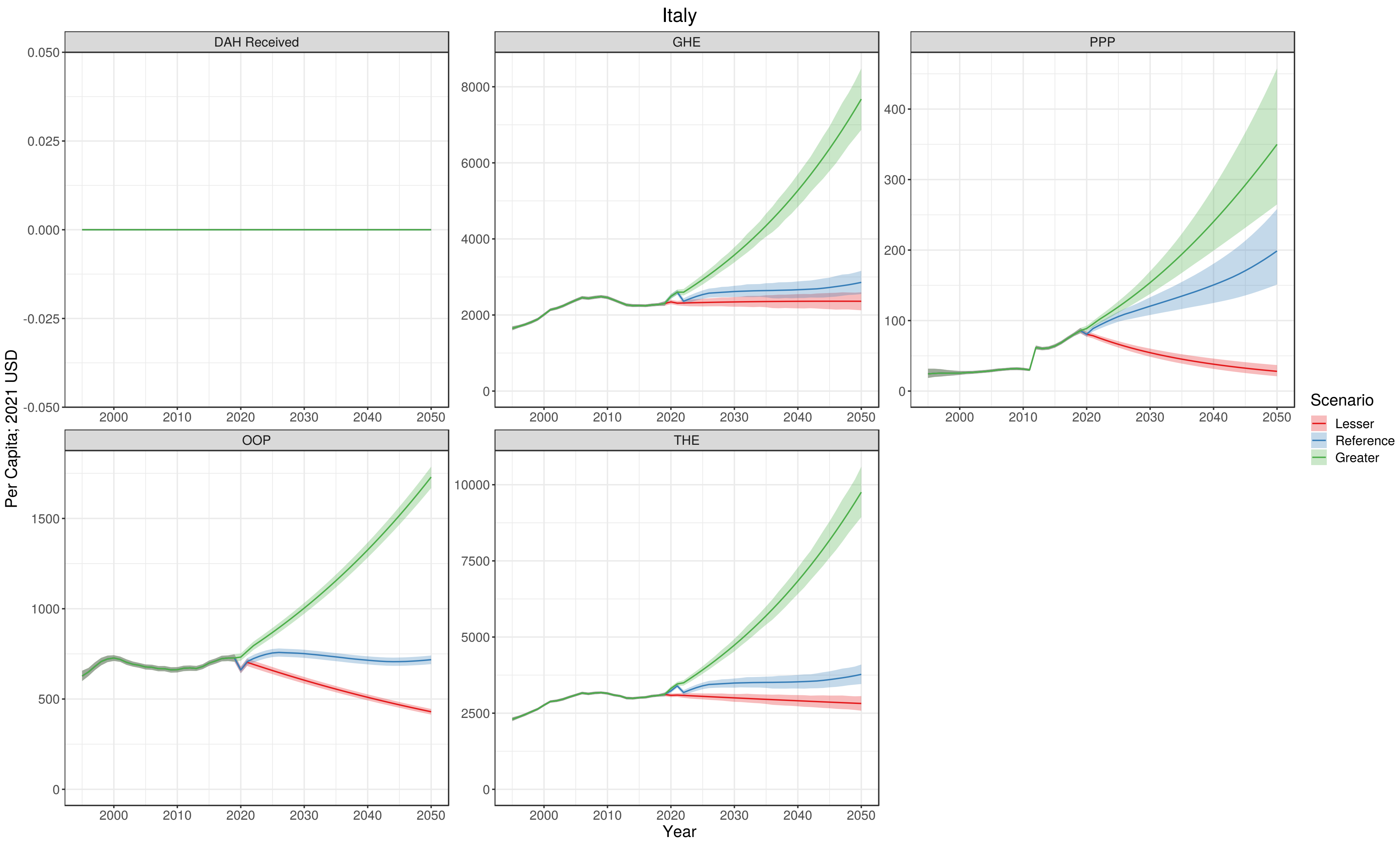

Italy
DAH Received
GHE
PPP
OOP
THE
0.050
0.025
0.000
-0.025
-0.050
8000
6000
4000
2000
0
400
300
200
100
1500
1000
500
10000
7500
5000
2500
2000
2010
2020
2030
2040
2050
Per Capita; 2021 USD
Year
Scenario
Lesser
Reference
Greater

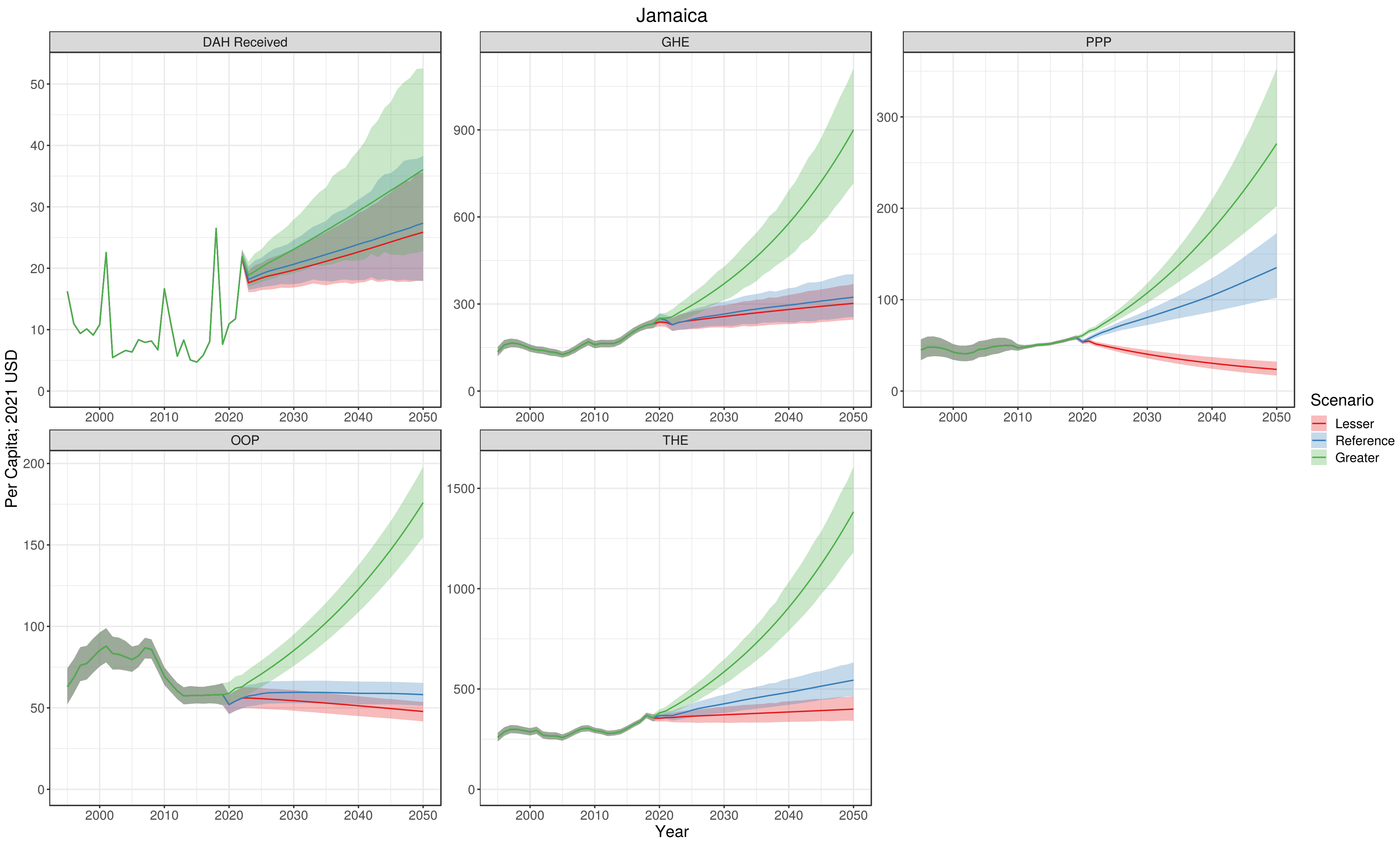
Jamaica
DAH Received
GHE
PPP
OOP
THE
Per Capita; 2021 USD
Year
Scenario
Lesser
Reference
Greater
0
10
20
30
40
50
300
600
900
100
200
150
500
1000
1500
2000
2010
2020
2030
2040
2050

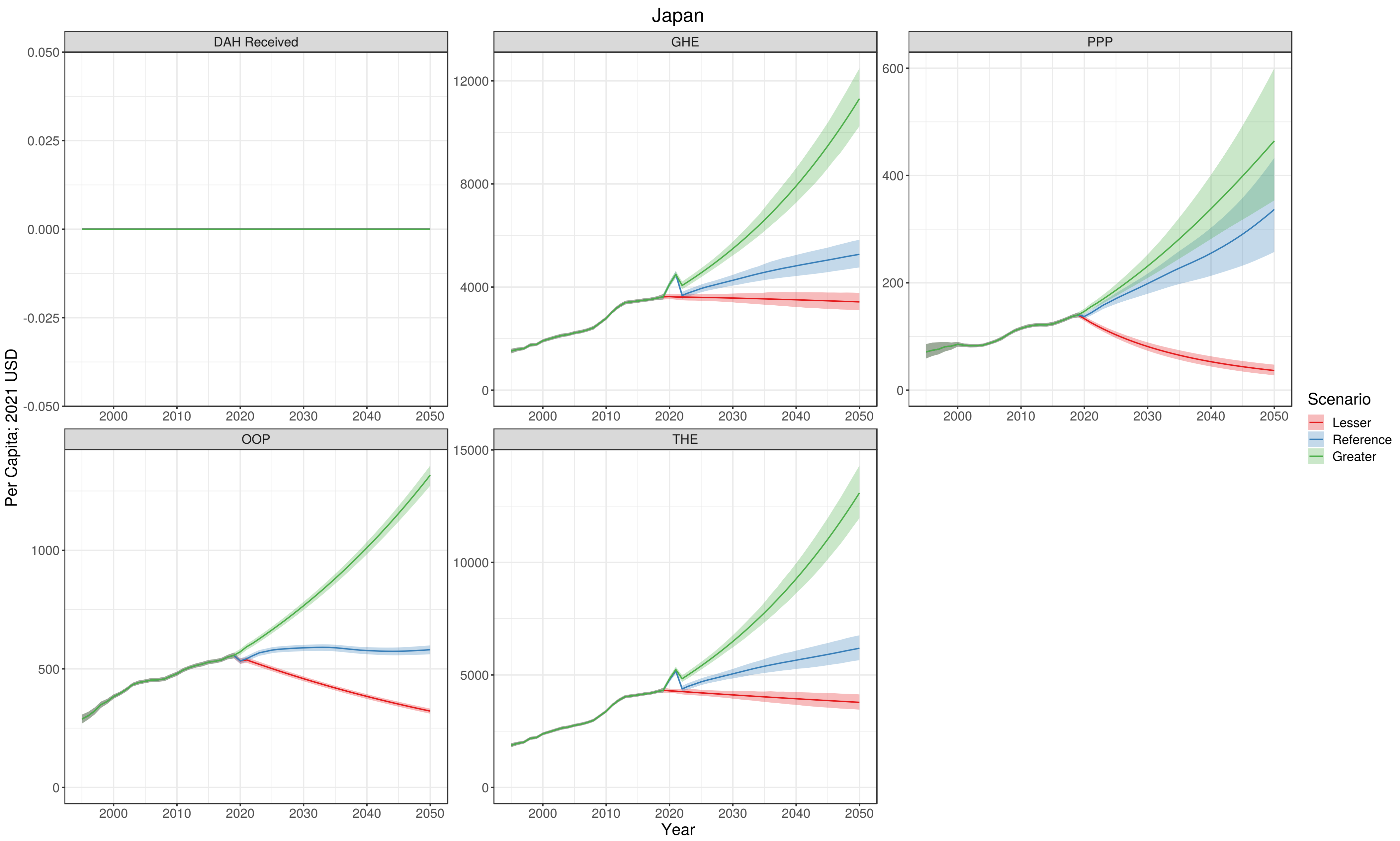

Japan
DAH Received
GHE
PPP
OOP
THE
Per Capita; 2021 USD
Year
Scenario
Lesser
Reference
Greater

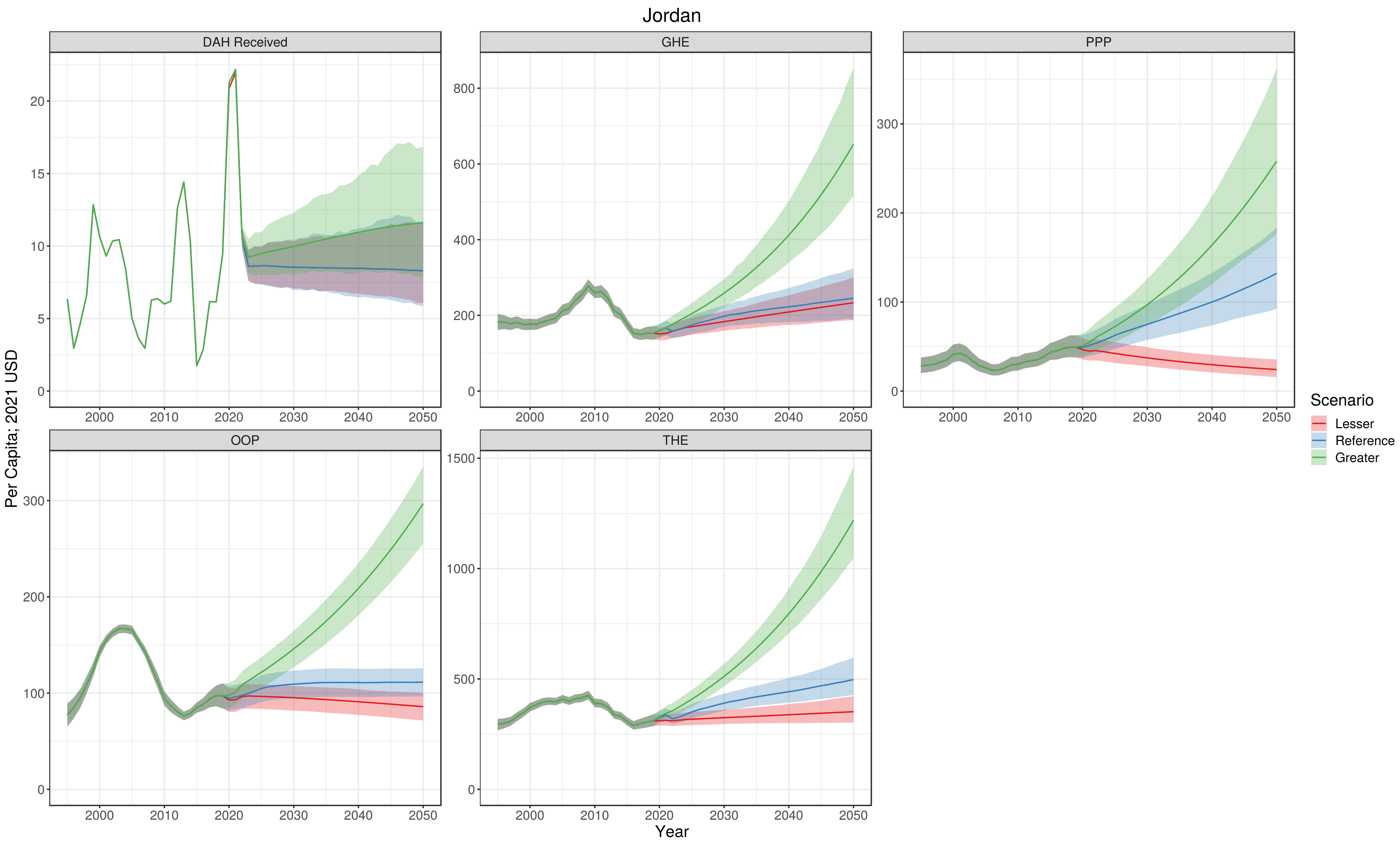

Jordan
DAH Received
GHE
PPP
OOP
THE
Per Capita; 2021 USD
Year
Scenario
Lesser
Reference
Greater

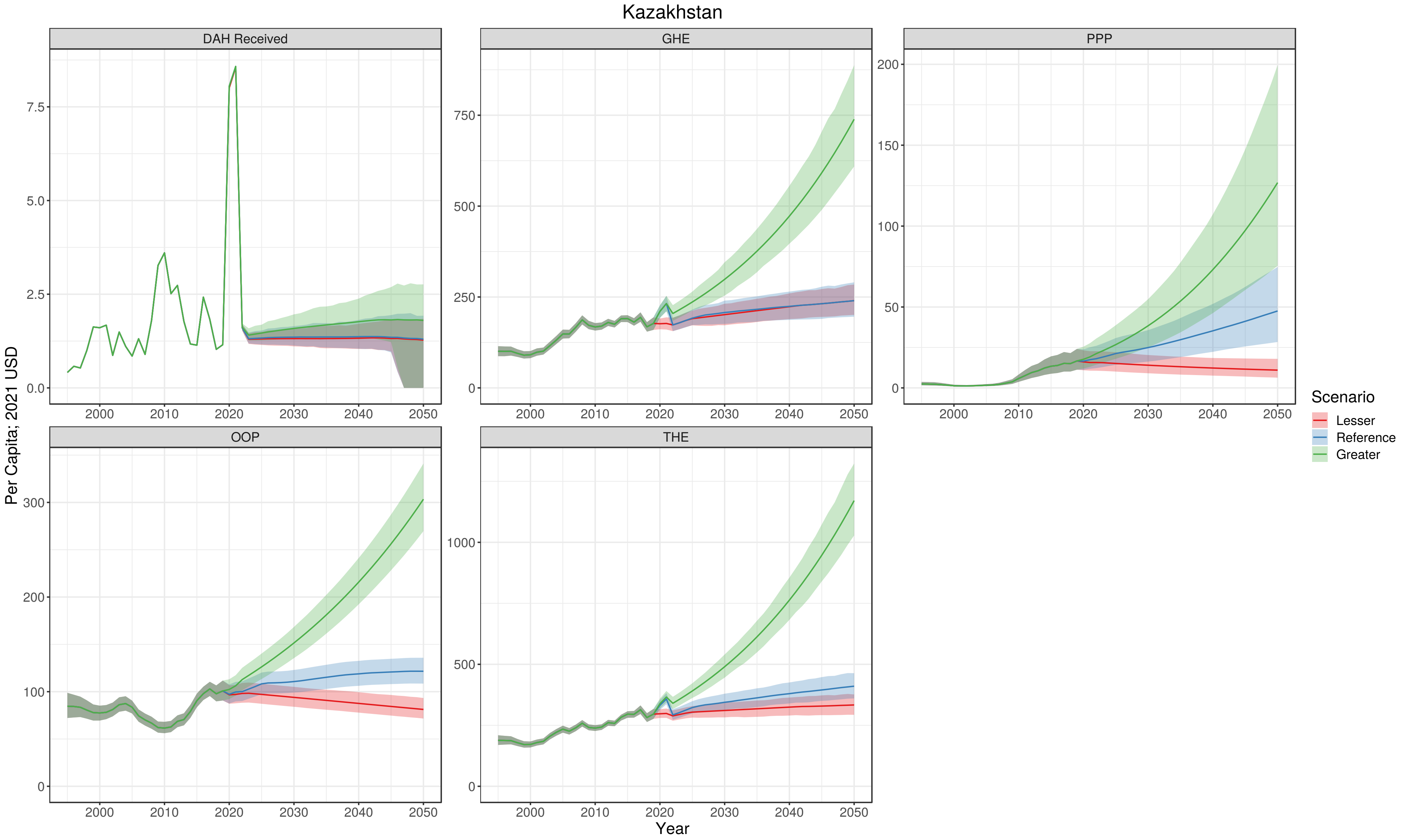

Kazakhstan
DAH Received
GHE
PPP
OOP
THE
Per Capita; 2021 USD
Year
Scenario
Lesser
Reference
Greater
0.0
2.5
5.0
7.5
0
250
500
750
0
50
100
150
200
0
100
200
300
0
500
1000
2000
2010
2020
2030
2040
2050

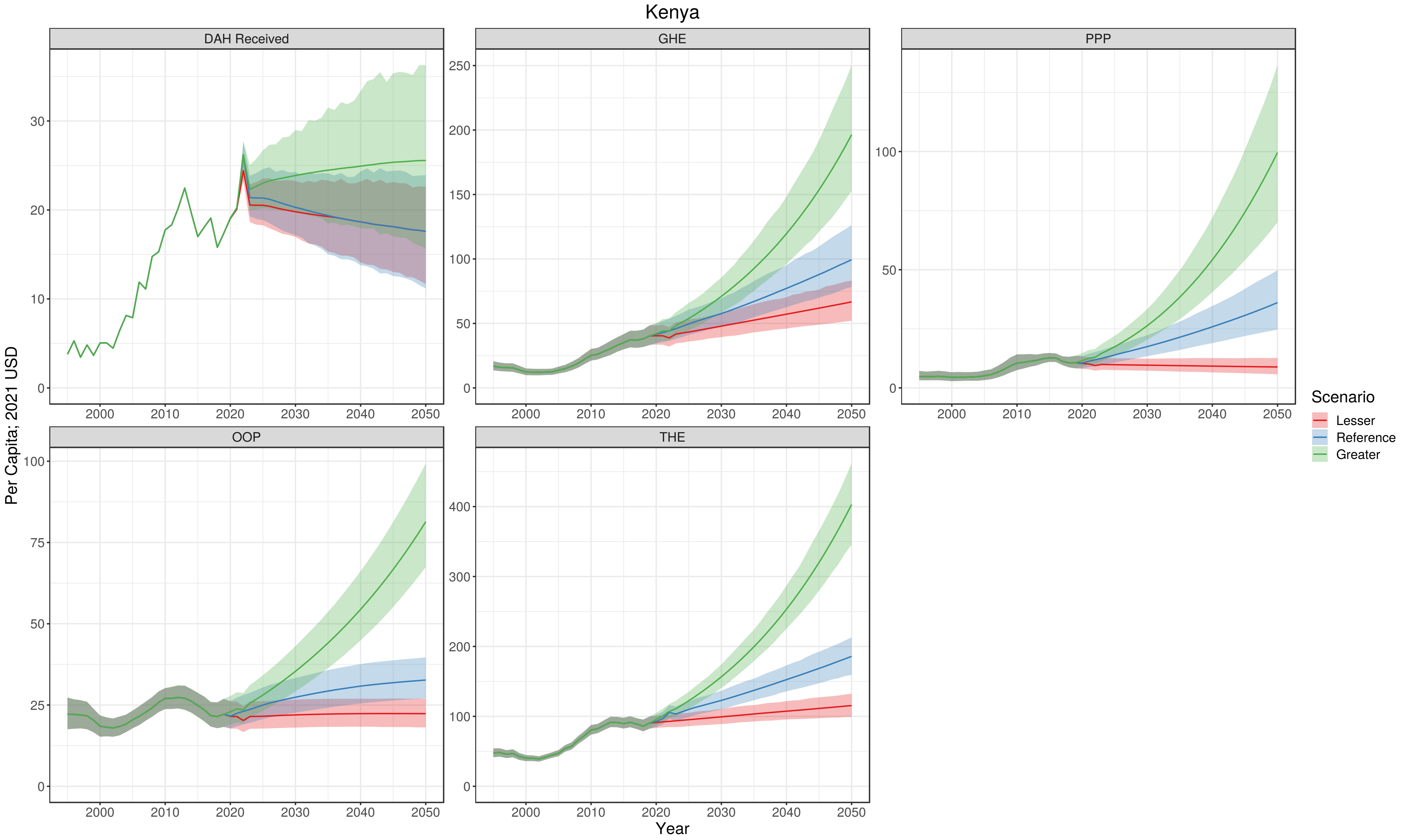

Kenya
DAH Received
GHE
PPP
OOP
THE
Per Capita; 2021 USD
Year
Scenario
Lesser
Reference
Greater

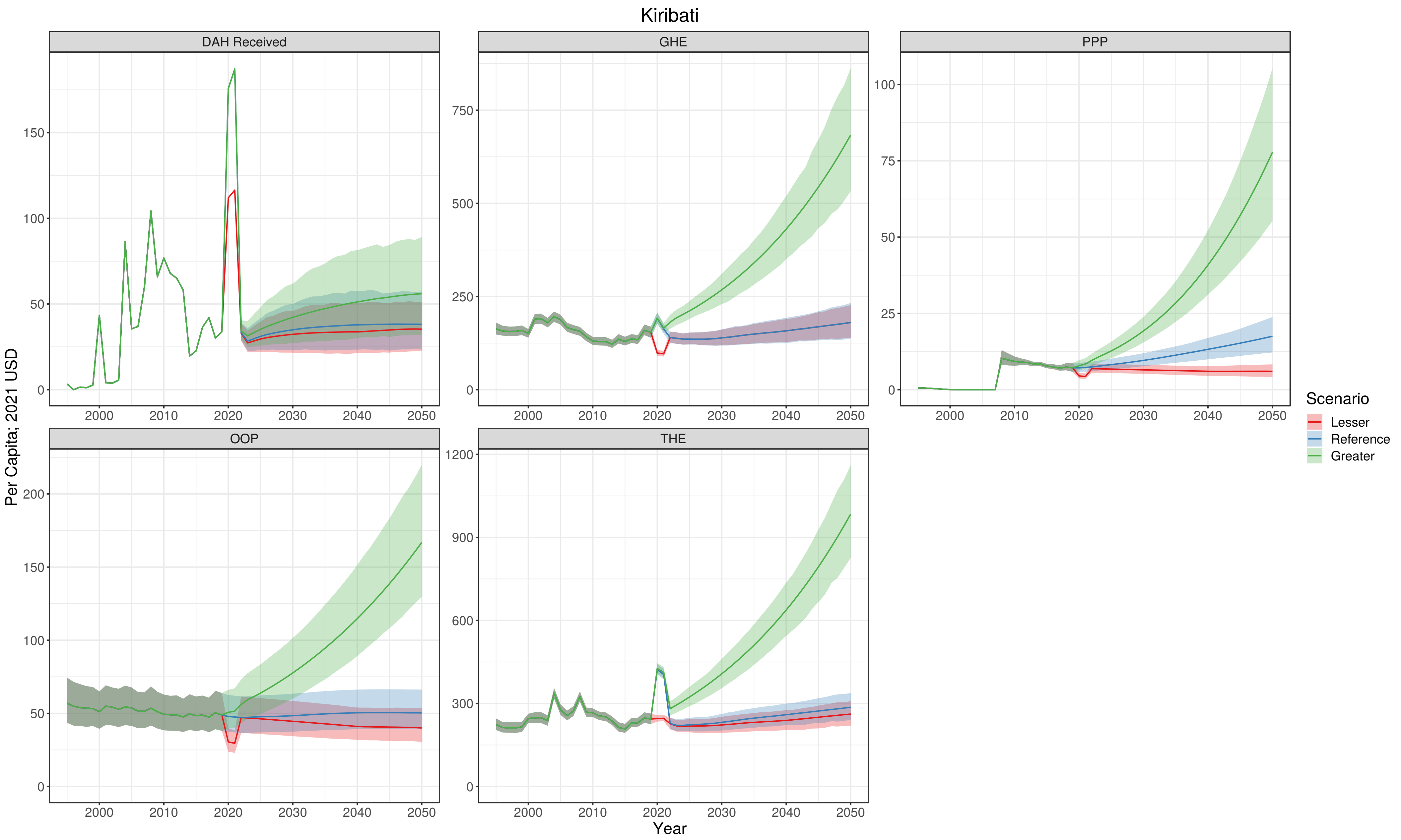

Kiribati
DAH Received
GHE
PPP
OOP
THE
Per Capita; 2021 USD
Year
Scenario
Lesser
Reference
Greater

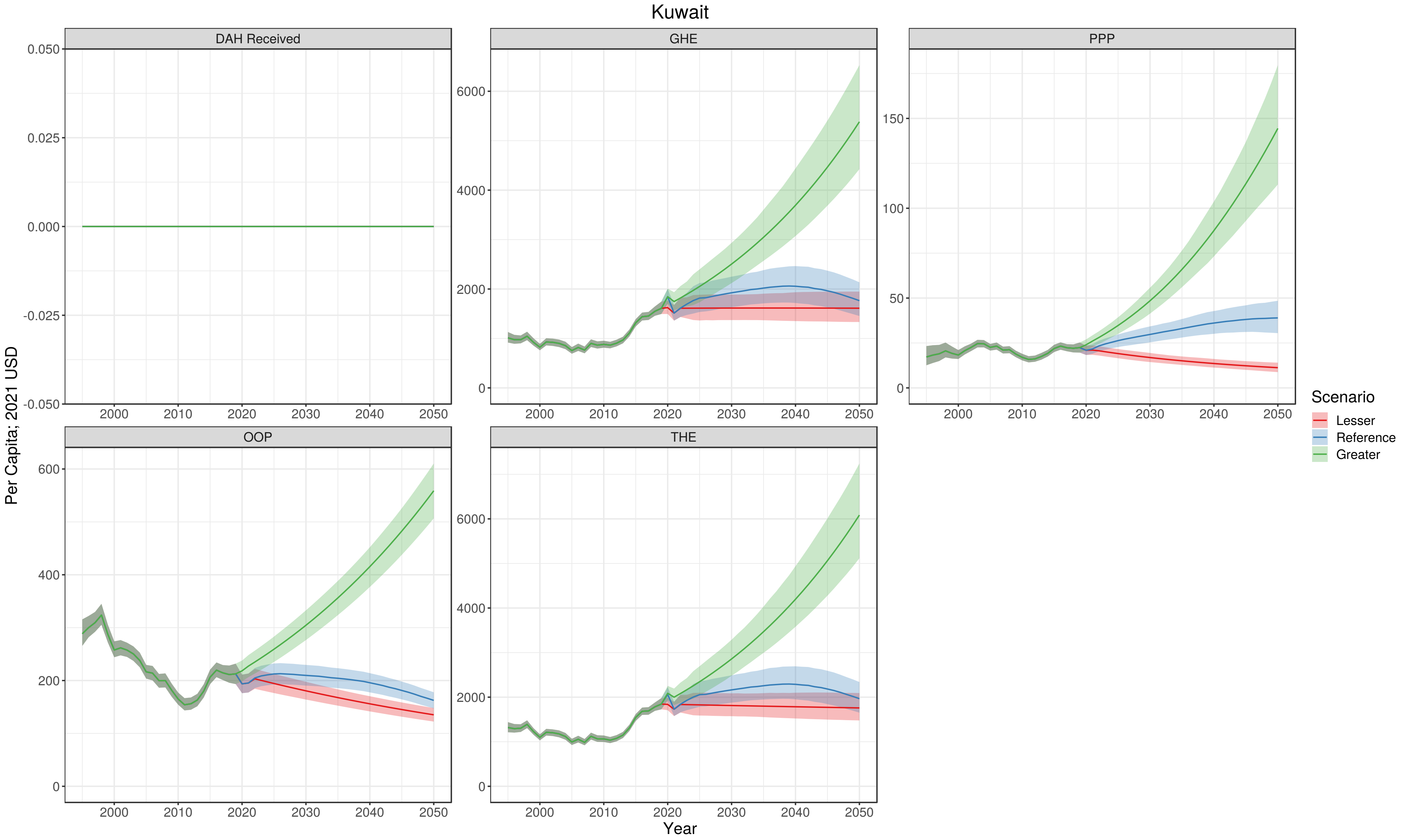

Kuwait
DAH Received
GHE
PPP
OOP
THE
Per Capita; 2021 USD
Year
Scenario
Lesser
Reference
Greater
0.050
0.025
0.000
-0.025
-0.050
6000
4000
2000
0
150
100
50
600
400
200
2000
2010
2020
2030
2040
2050

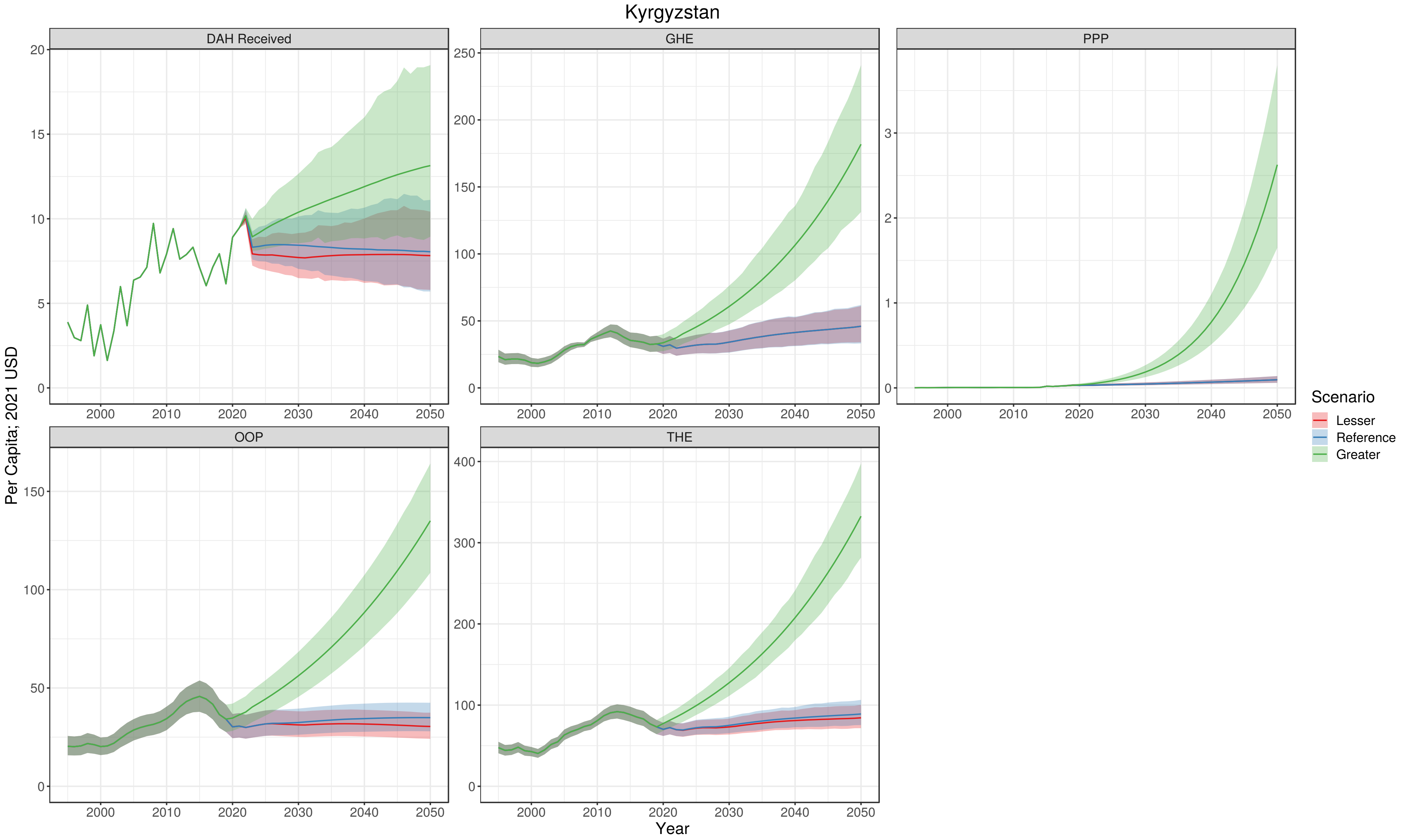
Kyrgyzstan
DAH Received
GHE
PPP
OOP
THE
Per Capita; 2021 USD
Year
Scenario
Lesser
Reference
Greater
2000
2010
2020
2030
2040
2050
0
5
10
15
20
50
100
150
200
250
1
2
3
300
400

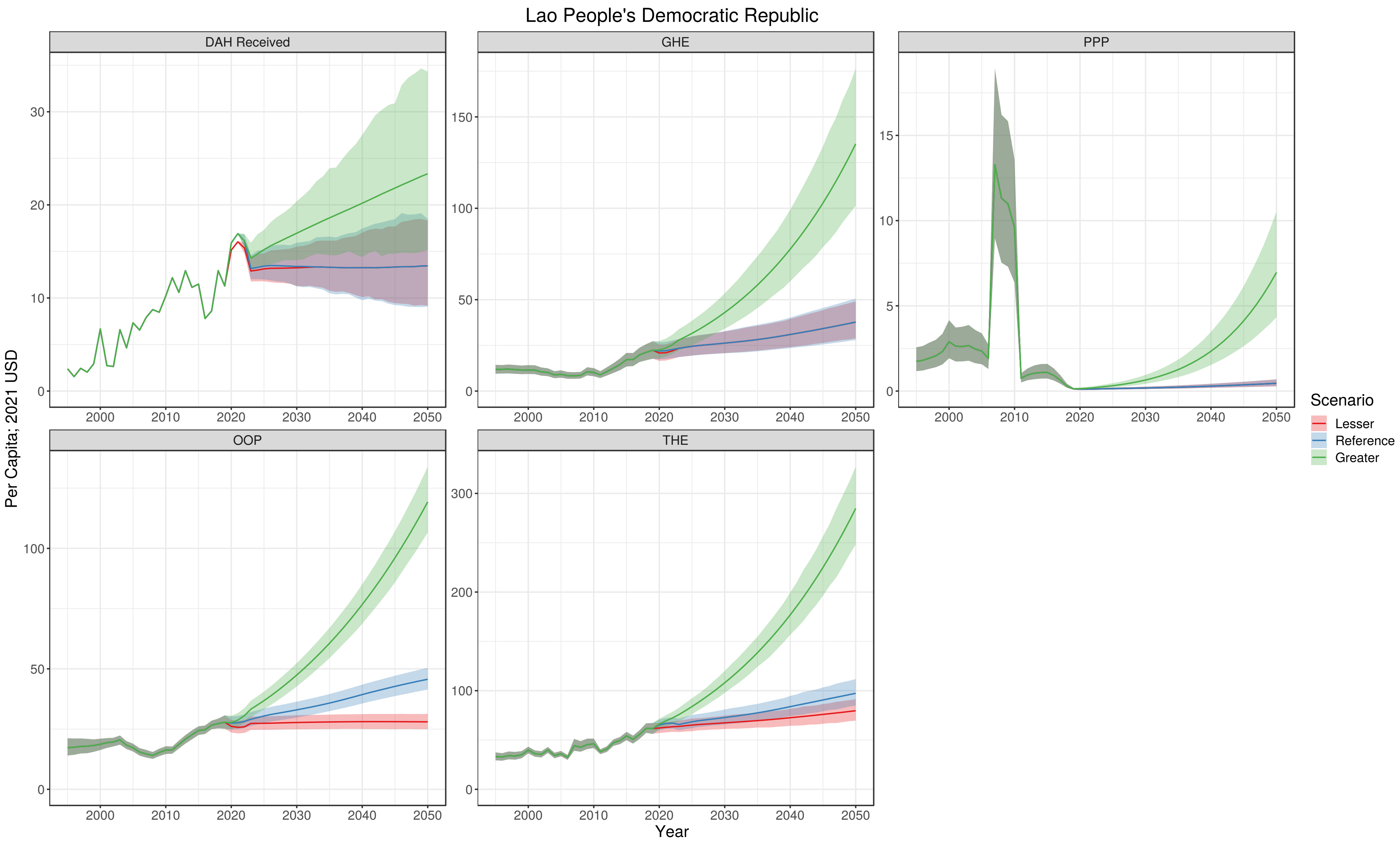
Lao People's Democratic Republic
DAH Received
GHE
PPP
OOP
THE
Per Capita; 2021 USD
Year
Scenario
Lesser
Reference
Greater
2000
2010
2020
2030
2040
2050

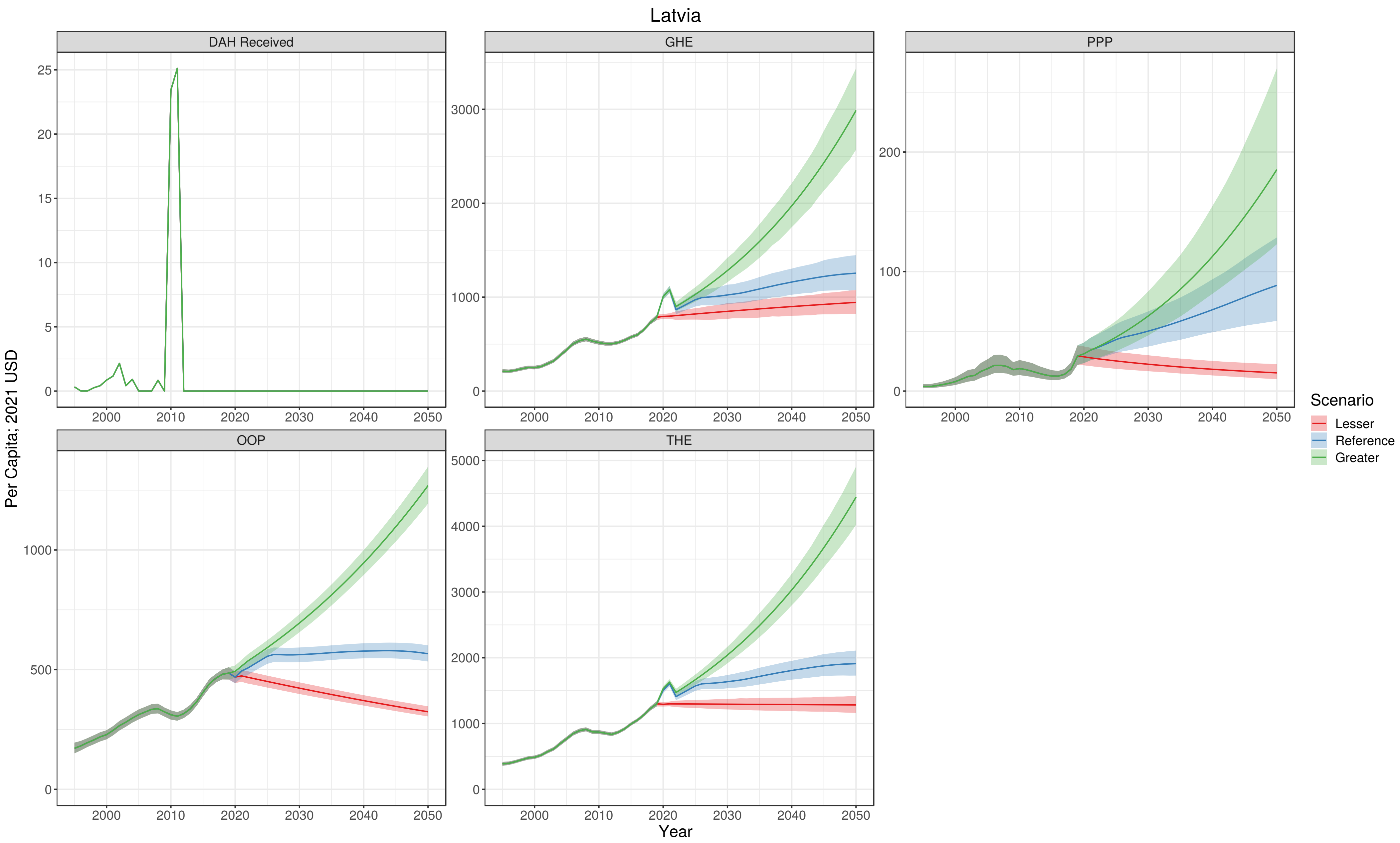
Latvia
DAH Received
GHE
PPP
OOP
THE
Per Capita; 2021 USD
Year
Scenario
Lesser
Reference
Greater
0
5
10
15
20
25
1000
2000
3000
100
200
500
4000
5000
2000
2010
2020
2030
2040
2050

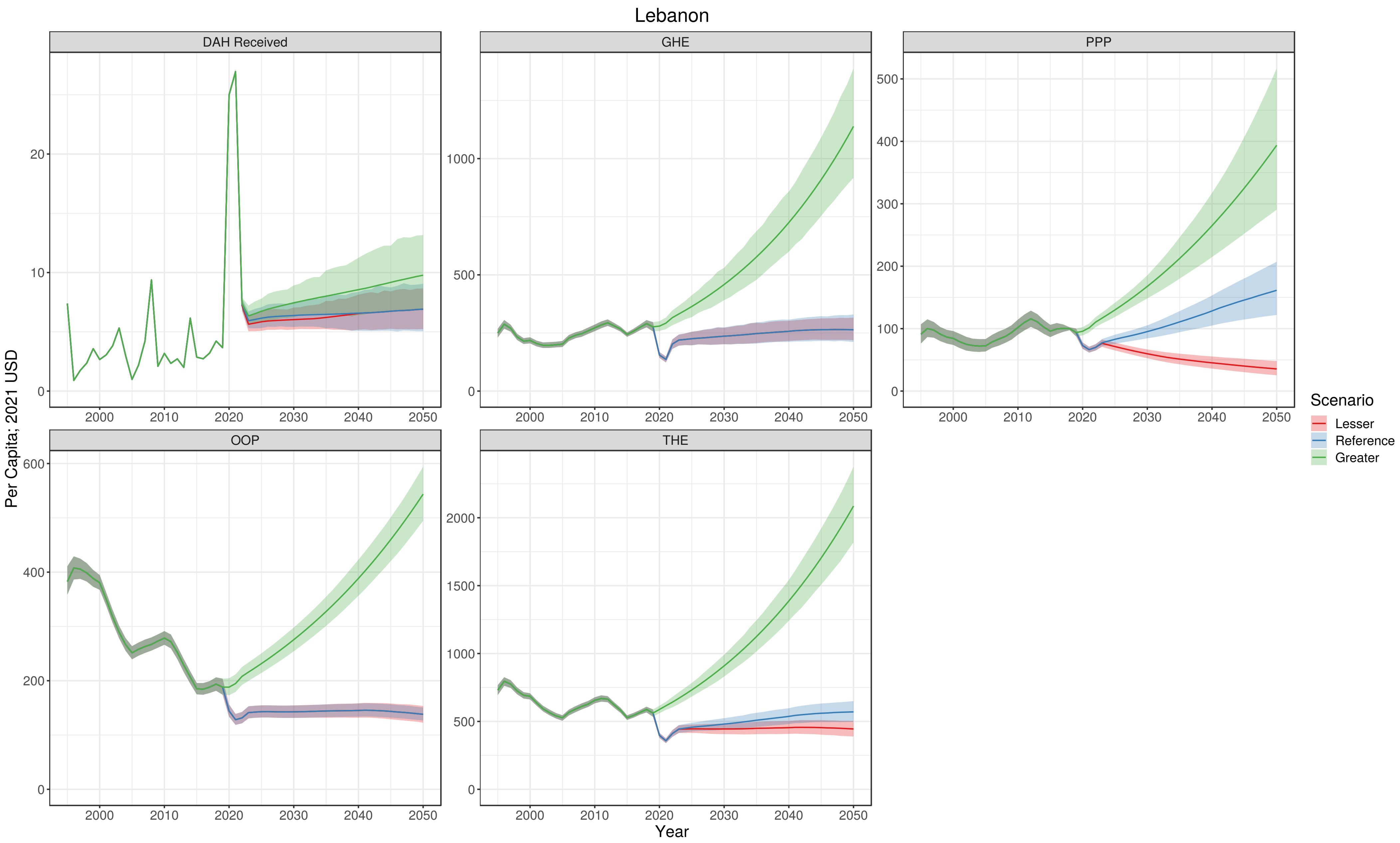

Lebanon
DAH Received
GHE
PPP
OOP
THE
Per Capita; 2021 USD
Year
Scenario
Lesser
Reference
Greater
0
10
20
0
500
1000
0
100
200
300
400
500
0
200
400
600
0
500
1000
1500
2000
2000
2010
2020
2030
2040
2050

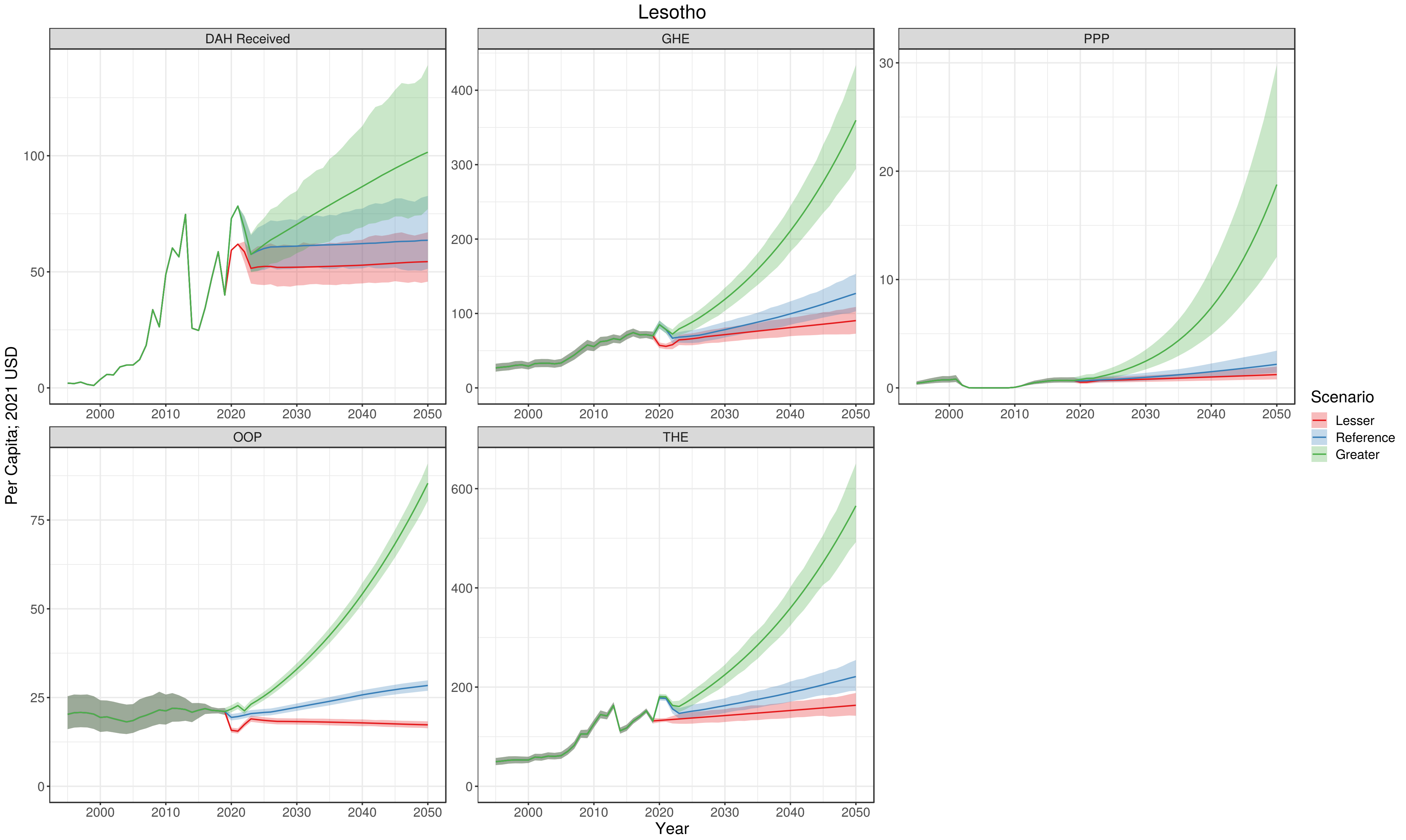

Lesotho
DAH Received
GHE
PPP
OOP
THE
Per Capita; 2021 USD
Year
Scenario
Lesser
Reference
Greater

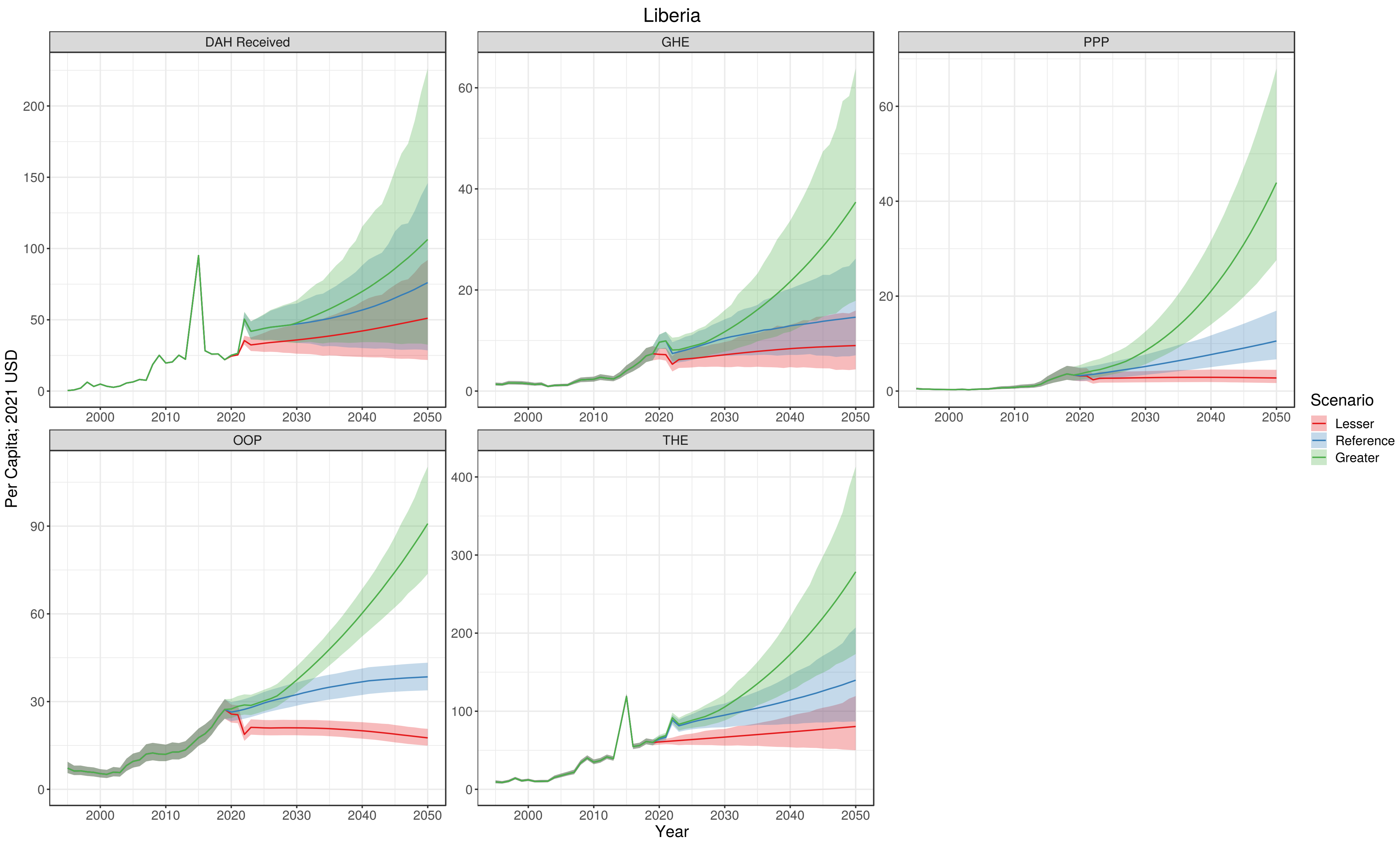
Liberia
DAH Received
GHE
PPP
OOP
THE
Per Capita; 2021 USD
Year
Scenario
Lesser
Reference
Greater

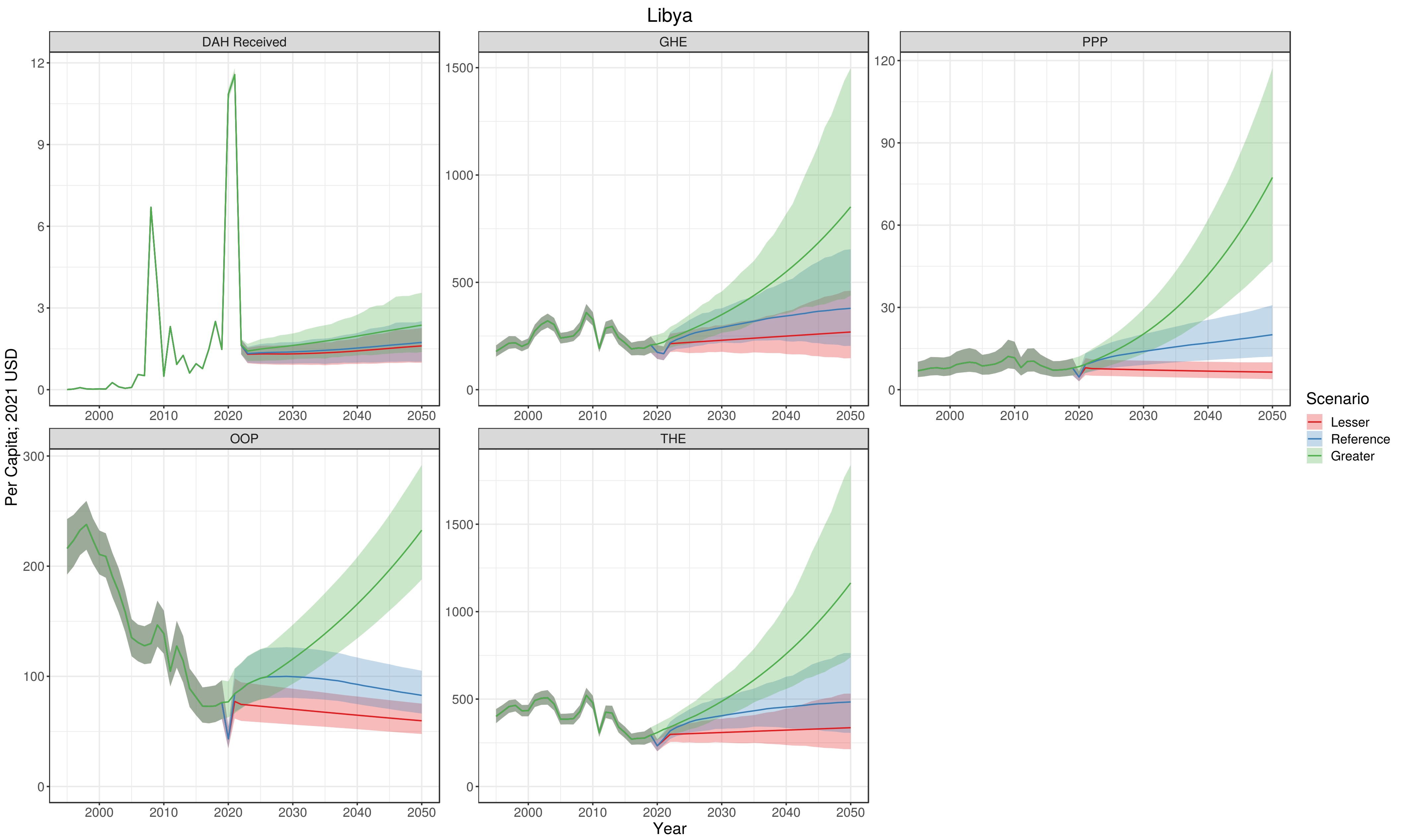

Libya
DAH Received
GHE
PPP
OOP
THE
Per Capita; 2021 USD
Year
Scenario
Lesser
Reference
Greater

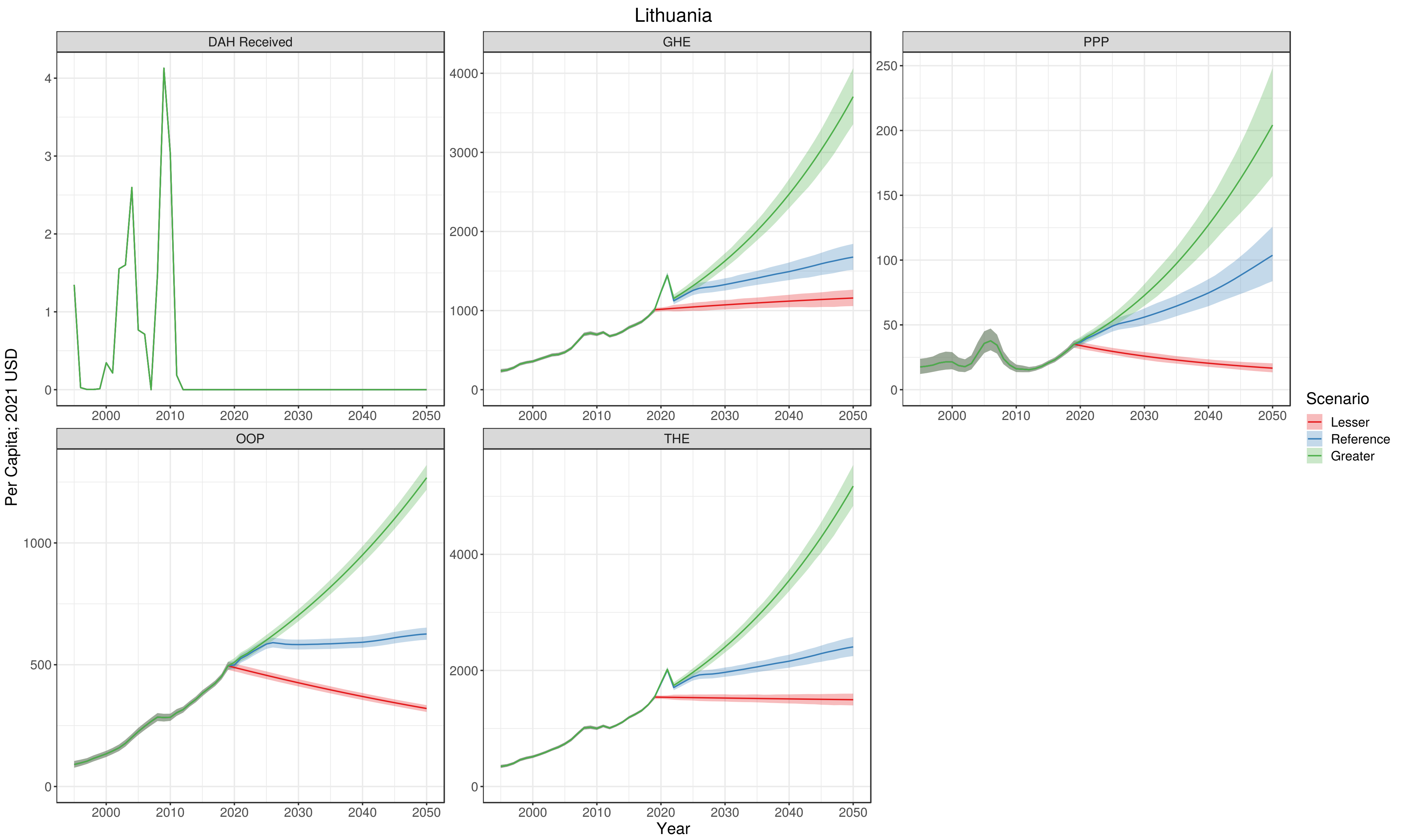

Lithuania
DAH Received
GHE
PPP
OOP
THE
Per Capita; 2021 USD
Year
Scenario
Lesser
Reference
Greater

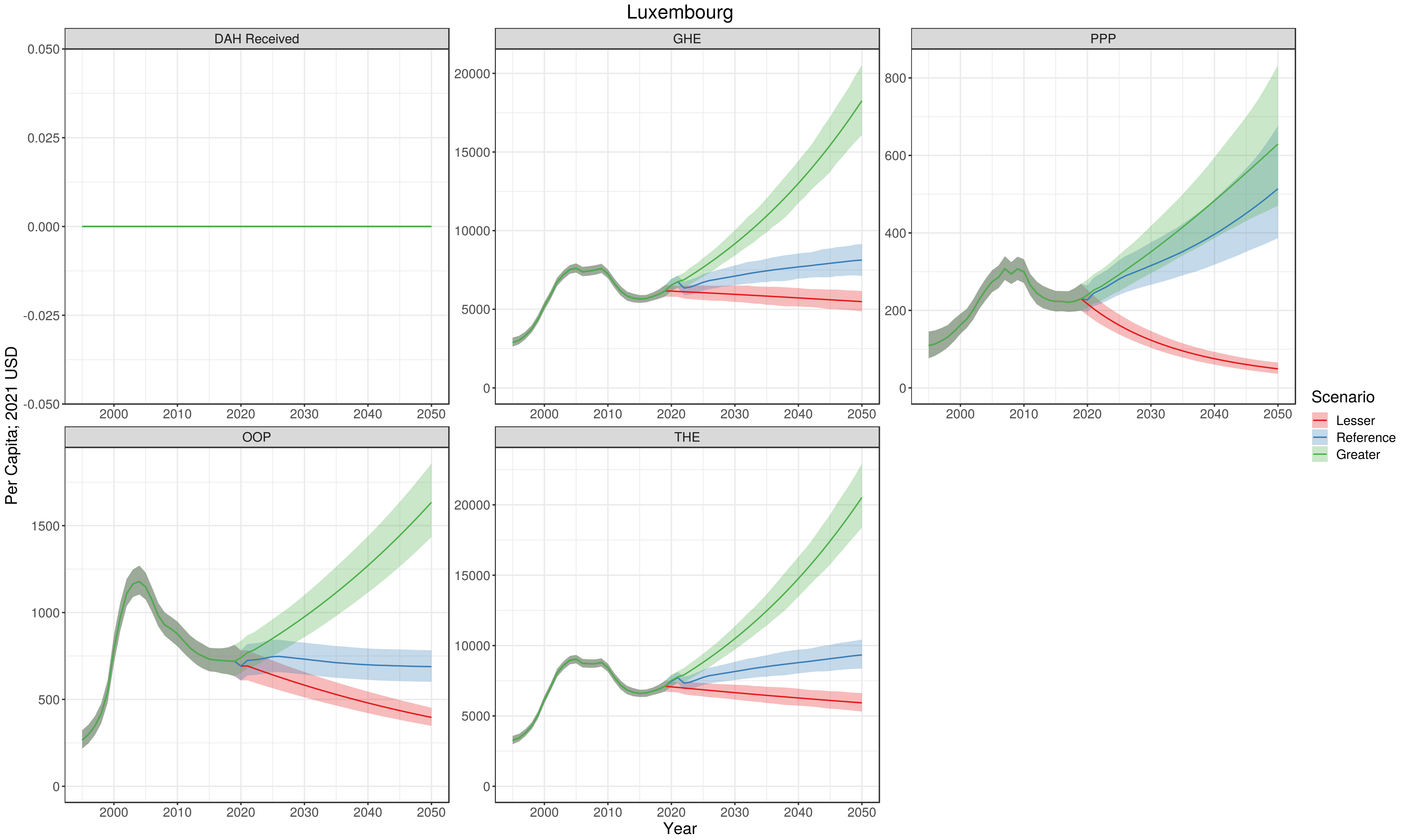

Luxembourg
DAH Received
GHE
PPP
OOP
THE
0.050
0.025
0.000
-0.025
-0.050
20000
15000
10000
5000
0
800
600
400
200
1500
1000
500
2000
2010
2020
2030
2040
2050
Per Capita; 2021 USD
Year
Scenario
Lesser
Reference
Greater

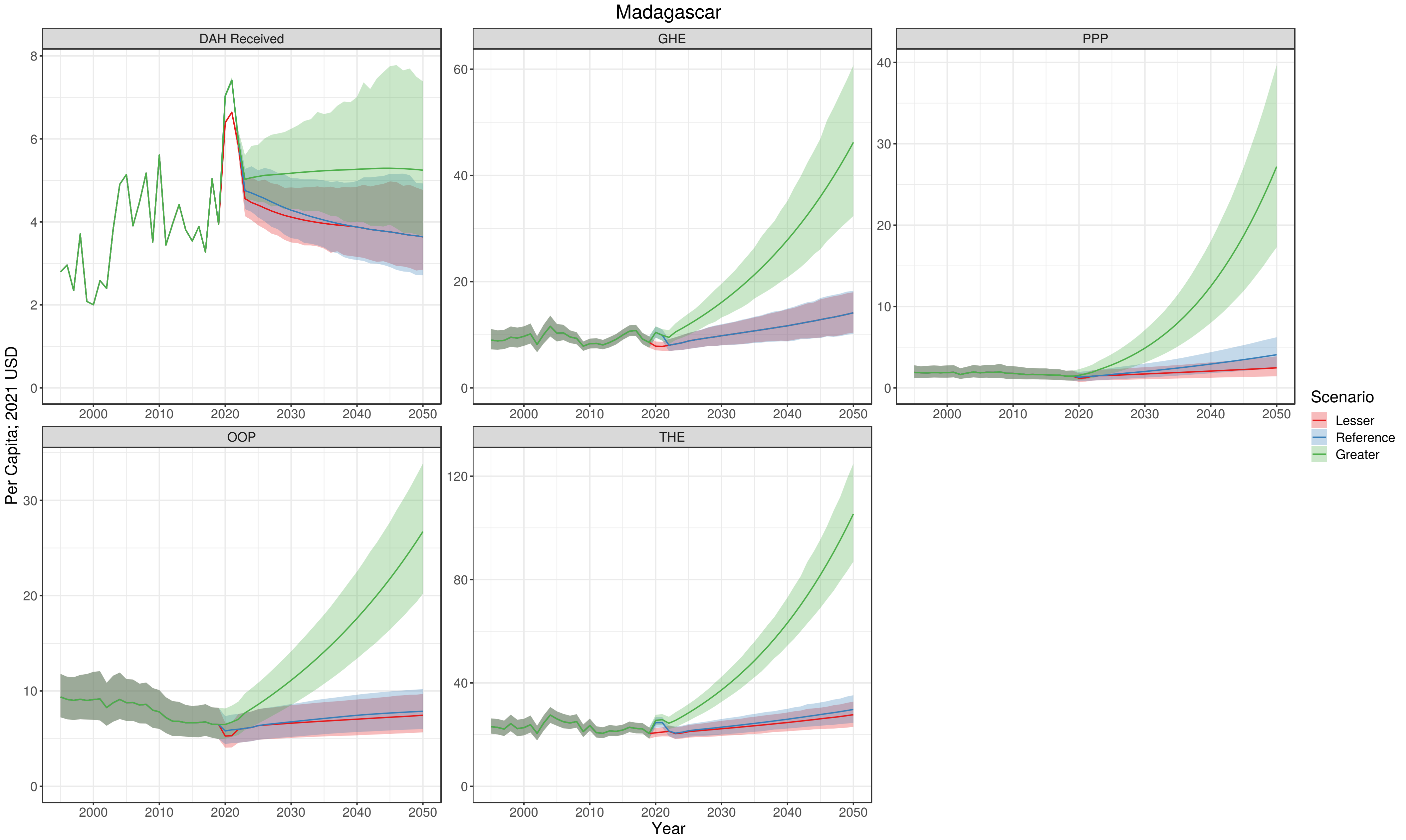

Madagascar
DAH Received
GHE
PPP
OOP
THE
Per Capita; 2021 USD
Year
Scenario
Lesser
Reference
Greater

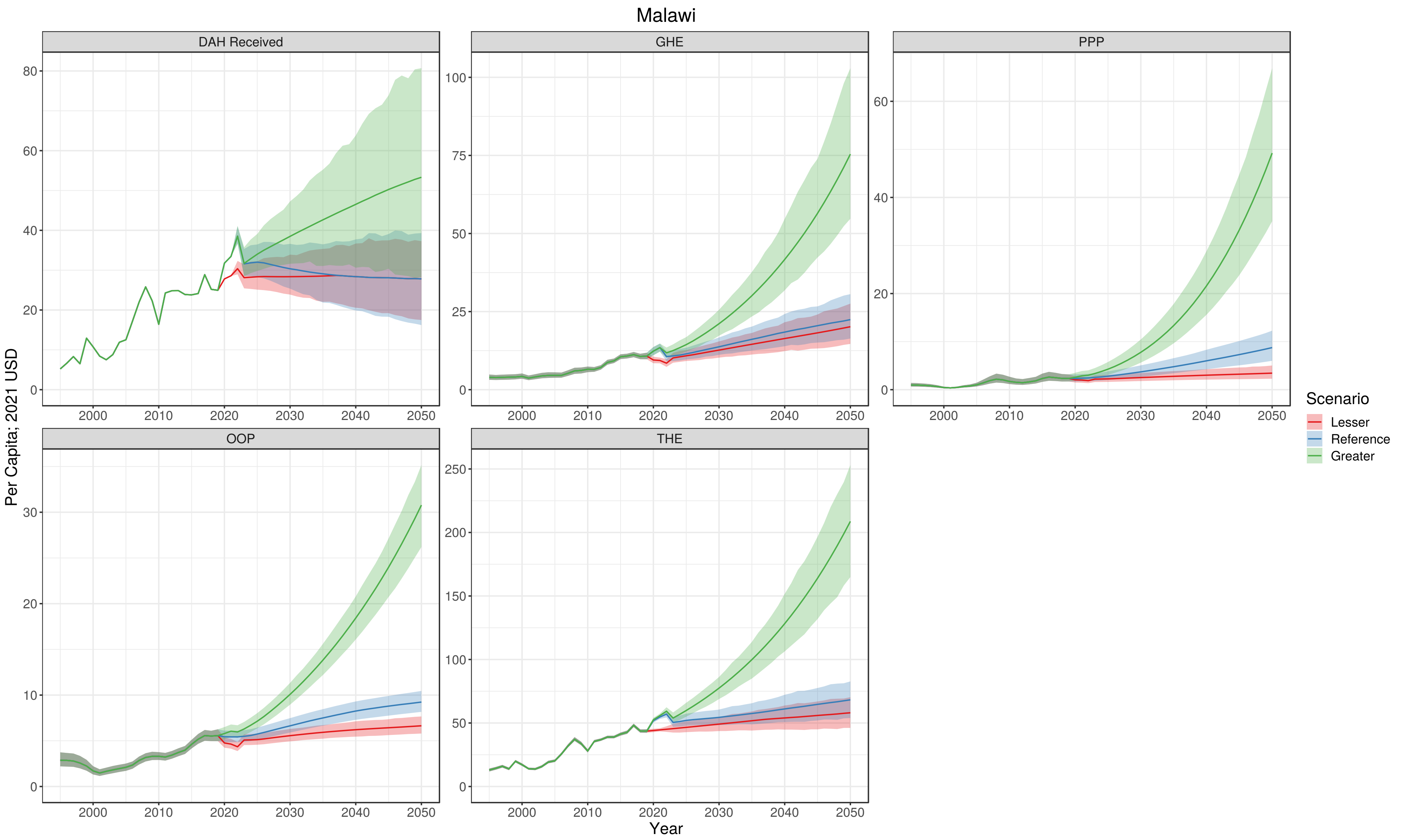

Malawi
DAH Received
GHE
PPP
OOP
THE
Per Capita; 2021 USD
Year
Scenario
Lesser
Reference
Greater

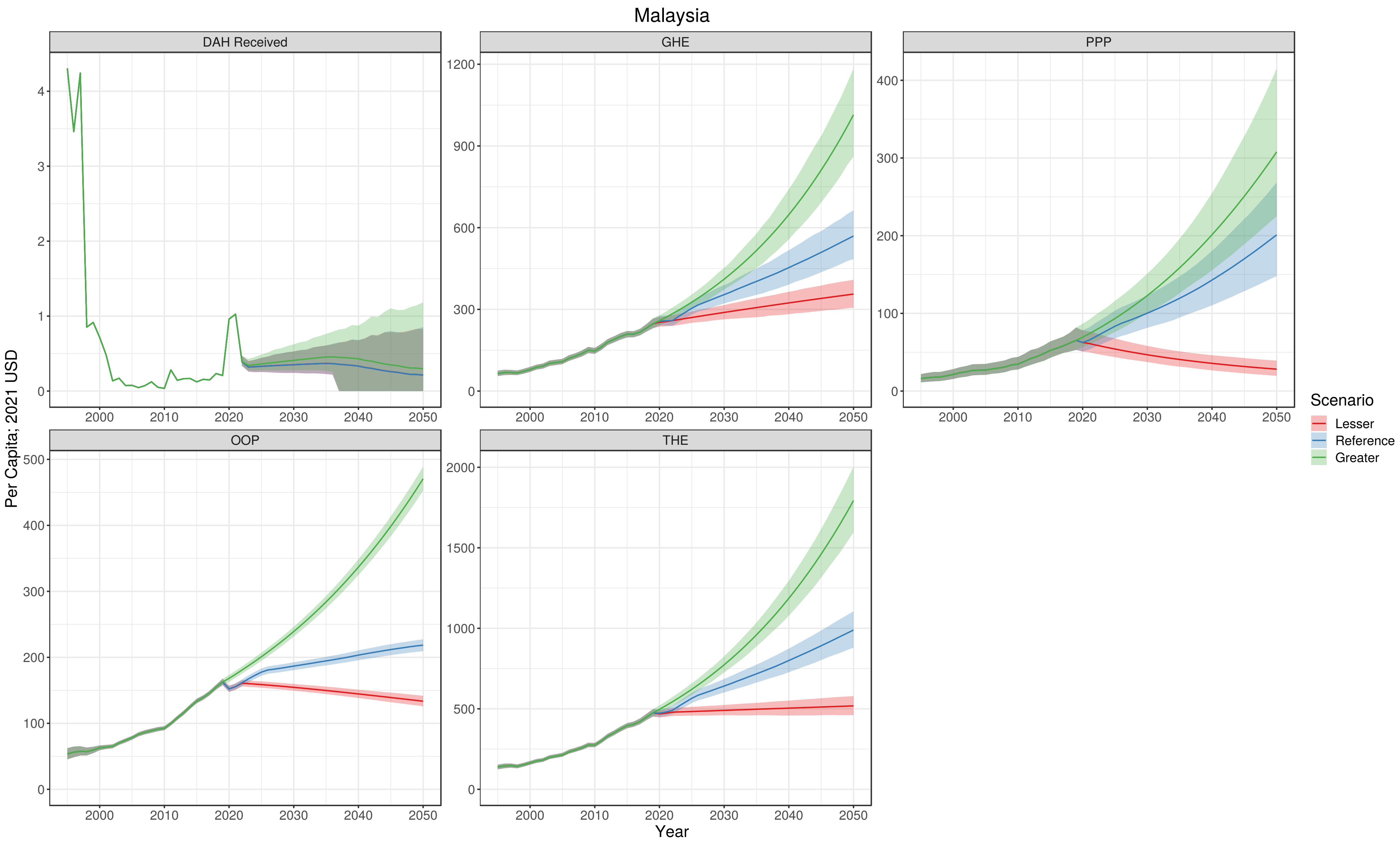

Malaysia
DAH Received
GHE
PPP
OOP
THE
Per Capita; 2021 USD
Year
Scenario
Lesser
Reference
Greater

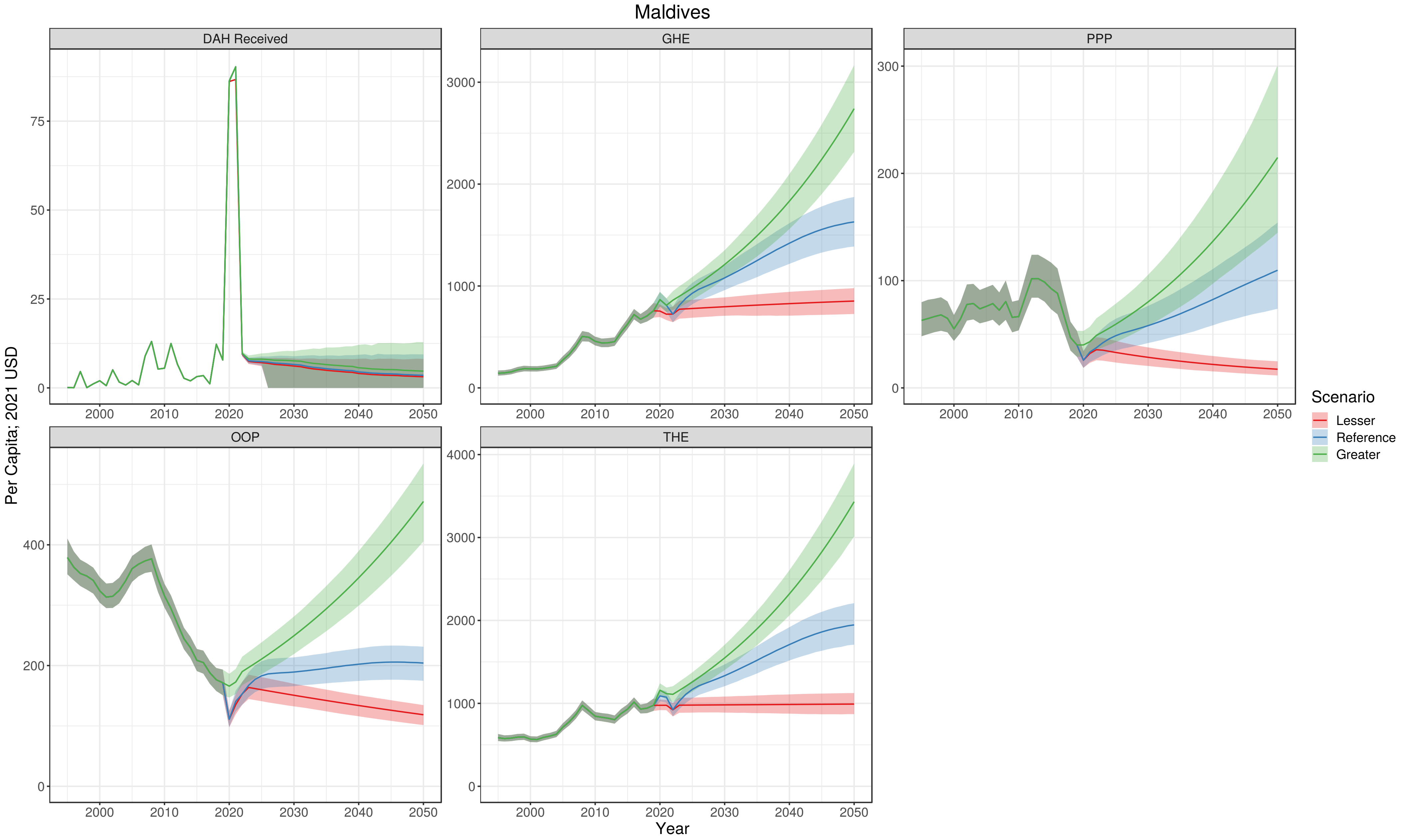
Maldives
DAH Received
GHE
PPP
OOP
THE
Per Capita; 2021 USD
Year
Scenario
Lesser
Reference
Greater
0
25
50
75
1000
2000
3000
100
200
300
400
4000
2000
2010
2020
2030
2040
2050

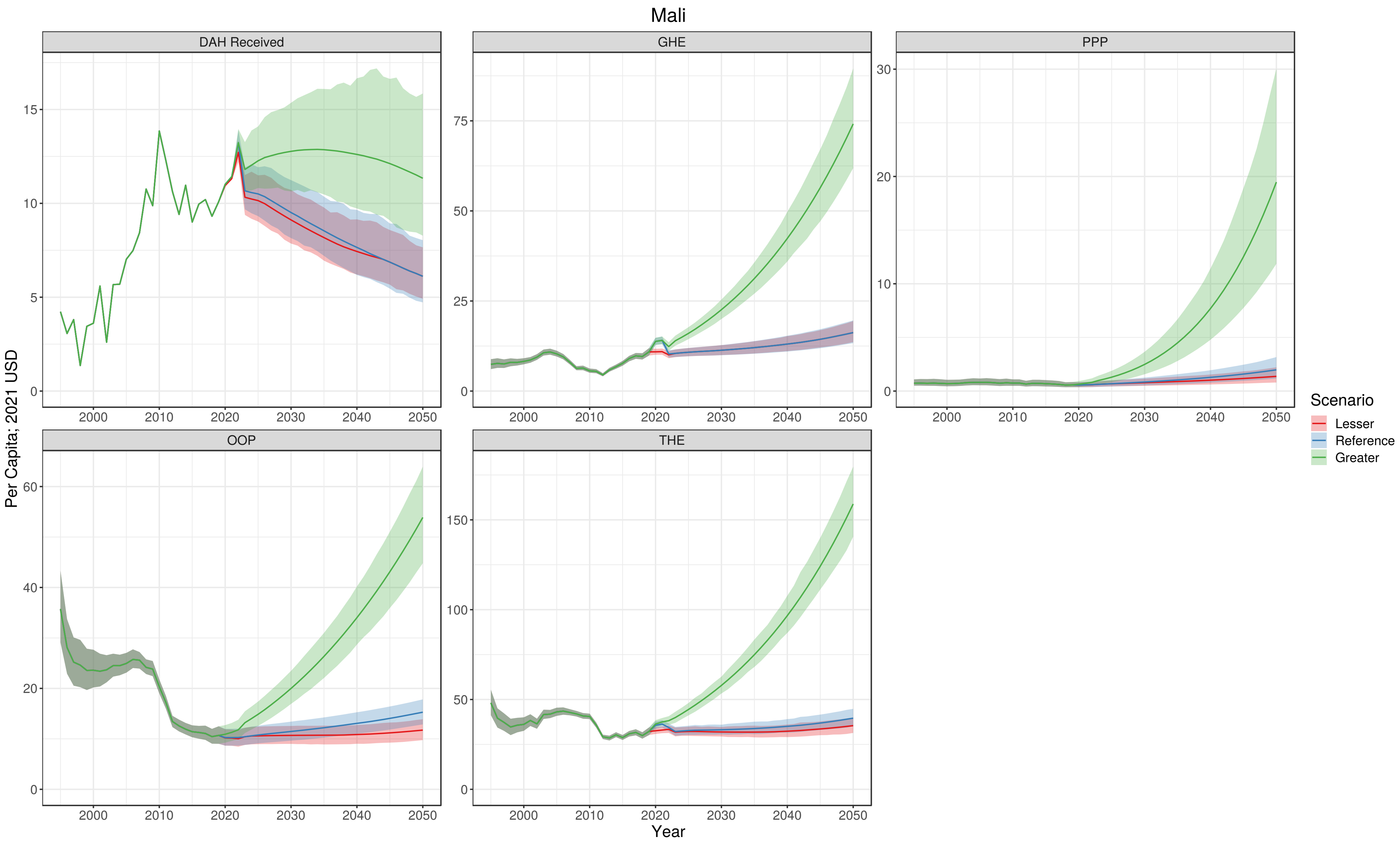

Mali
DAH Received
GHE
PPP
OOP
THE
Per Capita; 2021 USD
Year
Scenario
Lesser
Reference
Greater

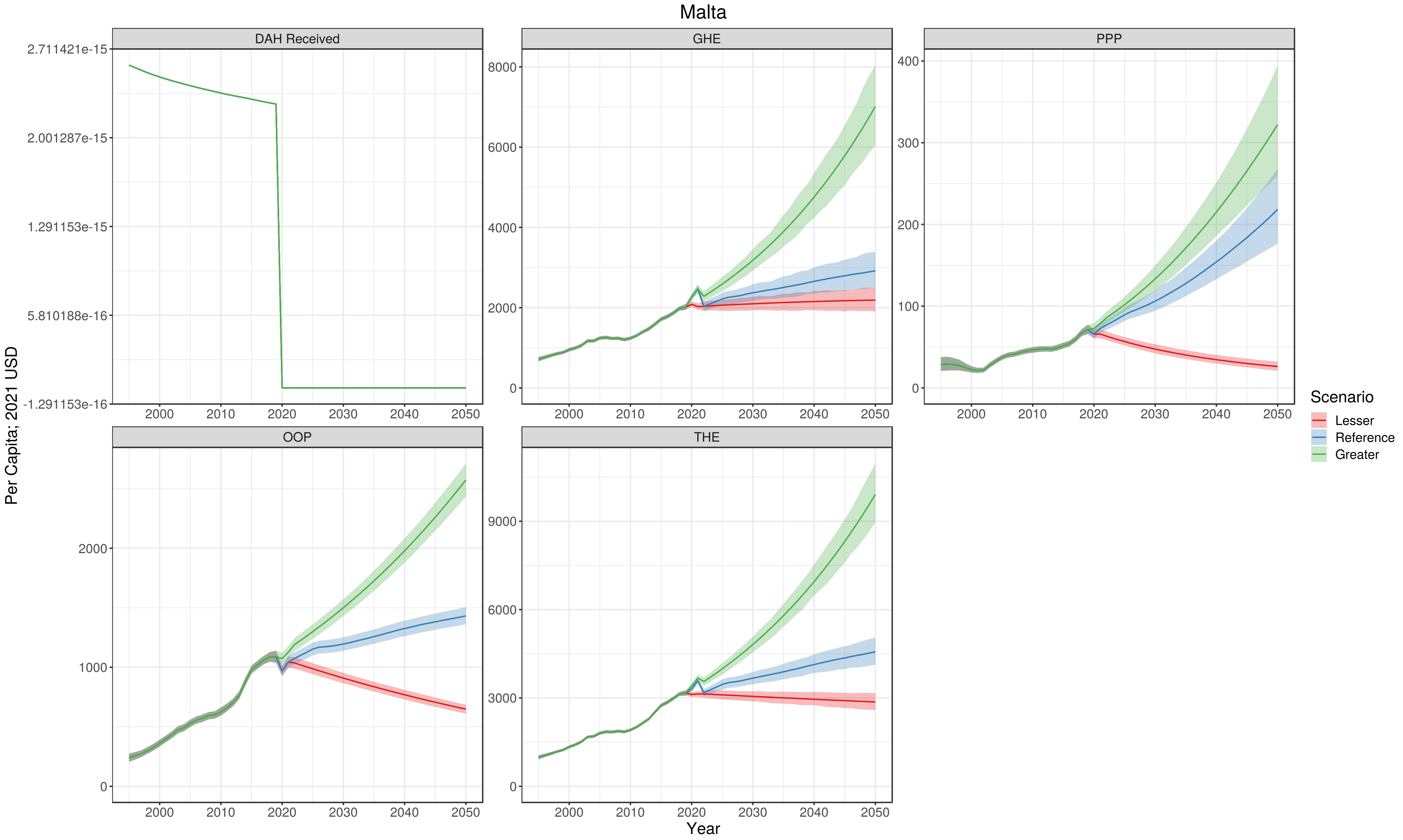

Malta
DAH Received
GHE
PPP
OOP
THE
2.711421e-15
2.001287e-15
1.291153e-15
5.810188e-16
-1.291153e-16
8000
6000
4000
2000
0
400
300
200
100
0
2000
1000
0
9000
6000
3000
0
2000
2010
2020
2030
2040
2050
Per Capita; 2021 USD
Year
Scenario
Lesser
Reference
Greater

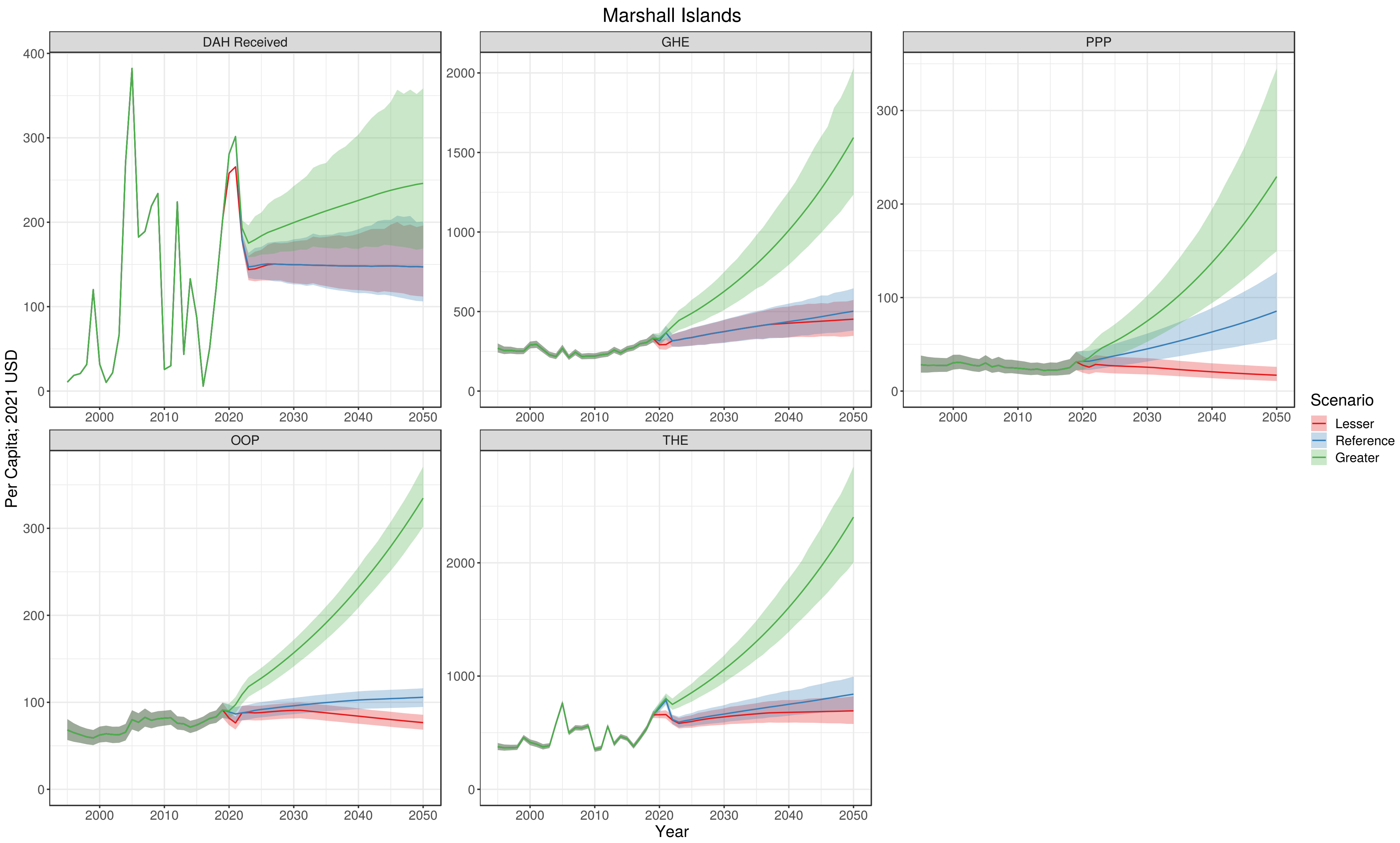

Marshall Islands
DAH Received
GHE
PPP
OOP
THE
Per Capita; 2021 USD
Year
Scenario
Lesser
Reference
Greater

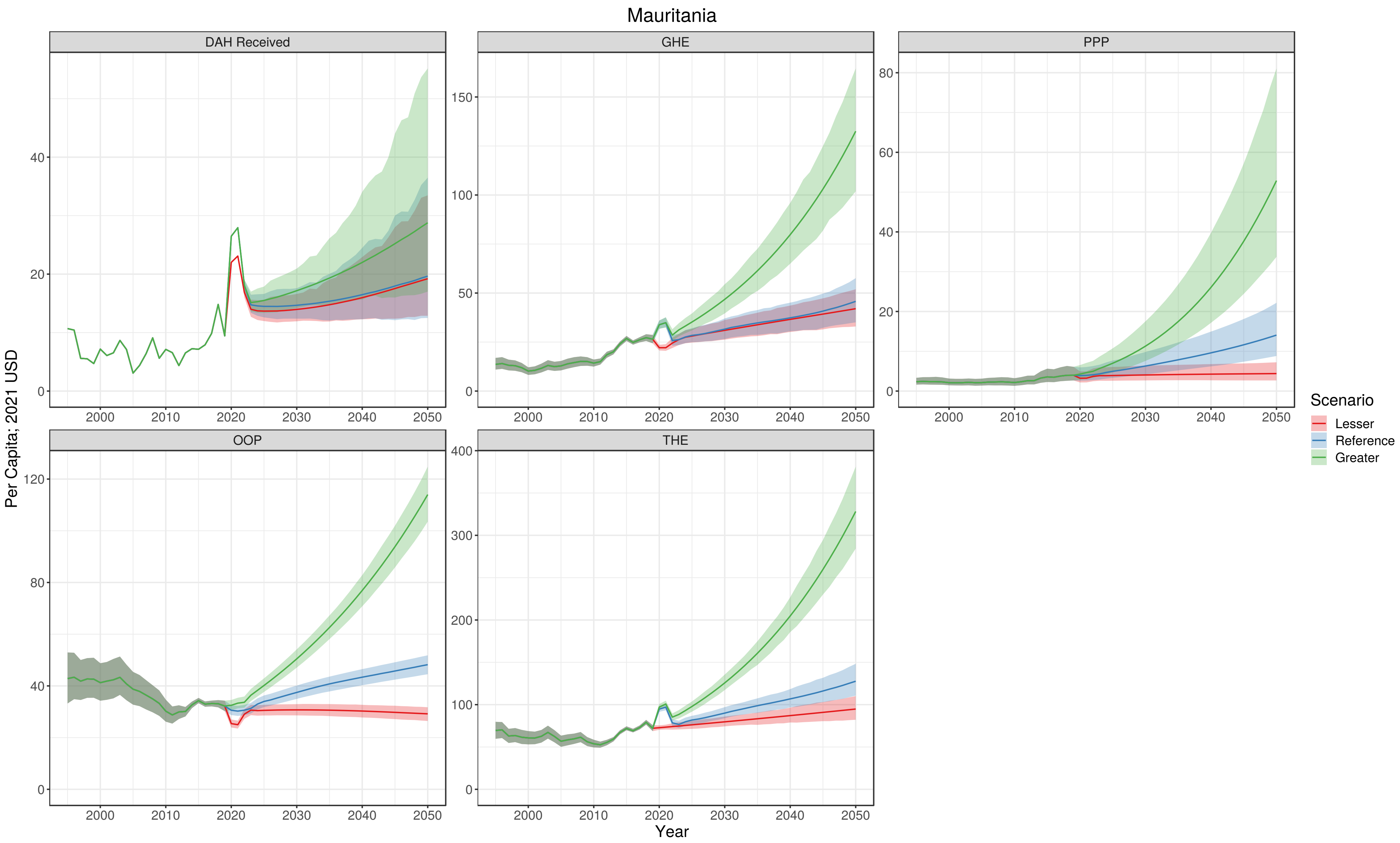

Mauritania
DAH Received
GHE
PPP
OOP
THE
Per Capita; 2021 USD
Year
Scenario
Lesser
Reference
Greater

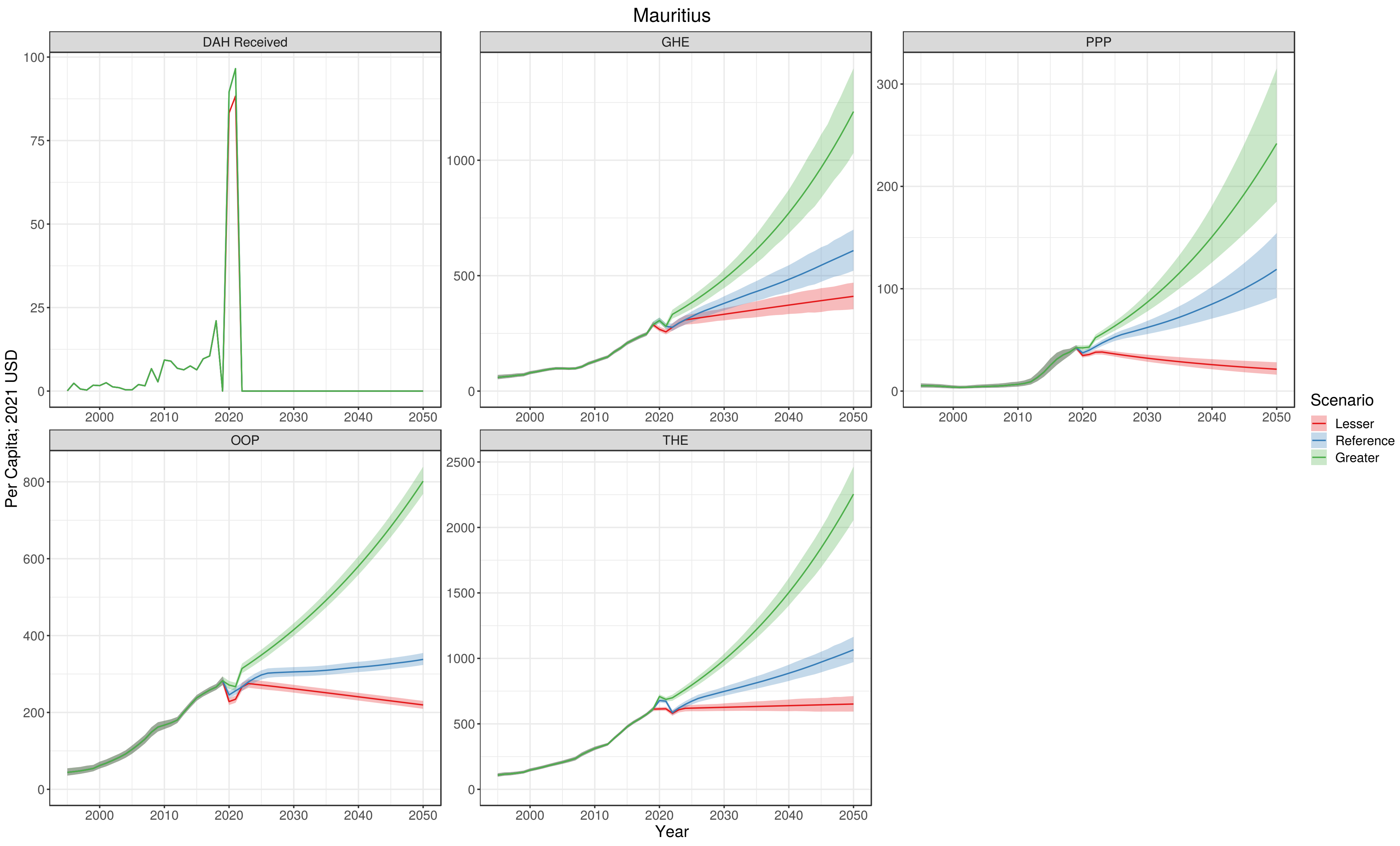

Mauritius
DAH Received
GHE
PPP
OOP
THE
Per Capita; 2021 USD
Year
Scenario
Lesser
Reference
Greater

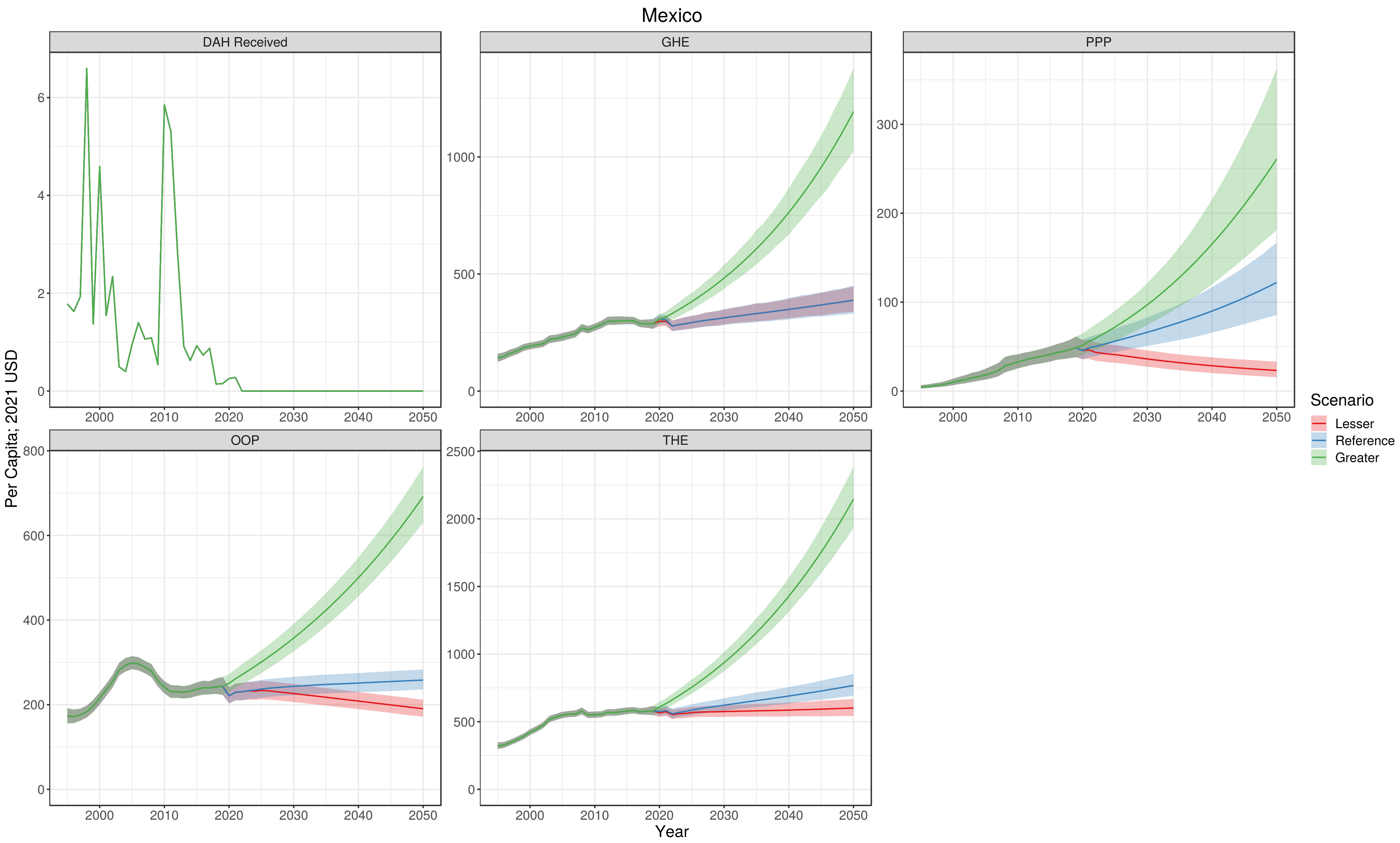

Mexico
DAH Received
GHE
PPP
OOP
THE
Per Capita; 2021 USD
Year
Scenario
Lesser
Reference
Greater

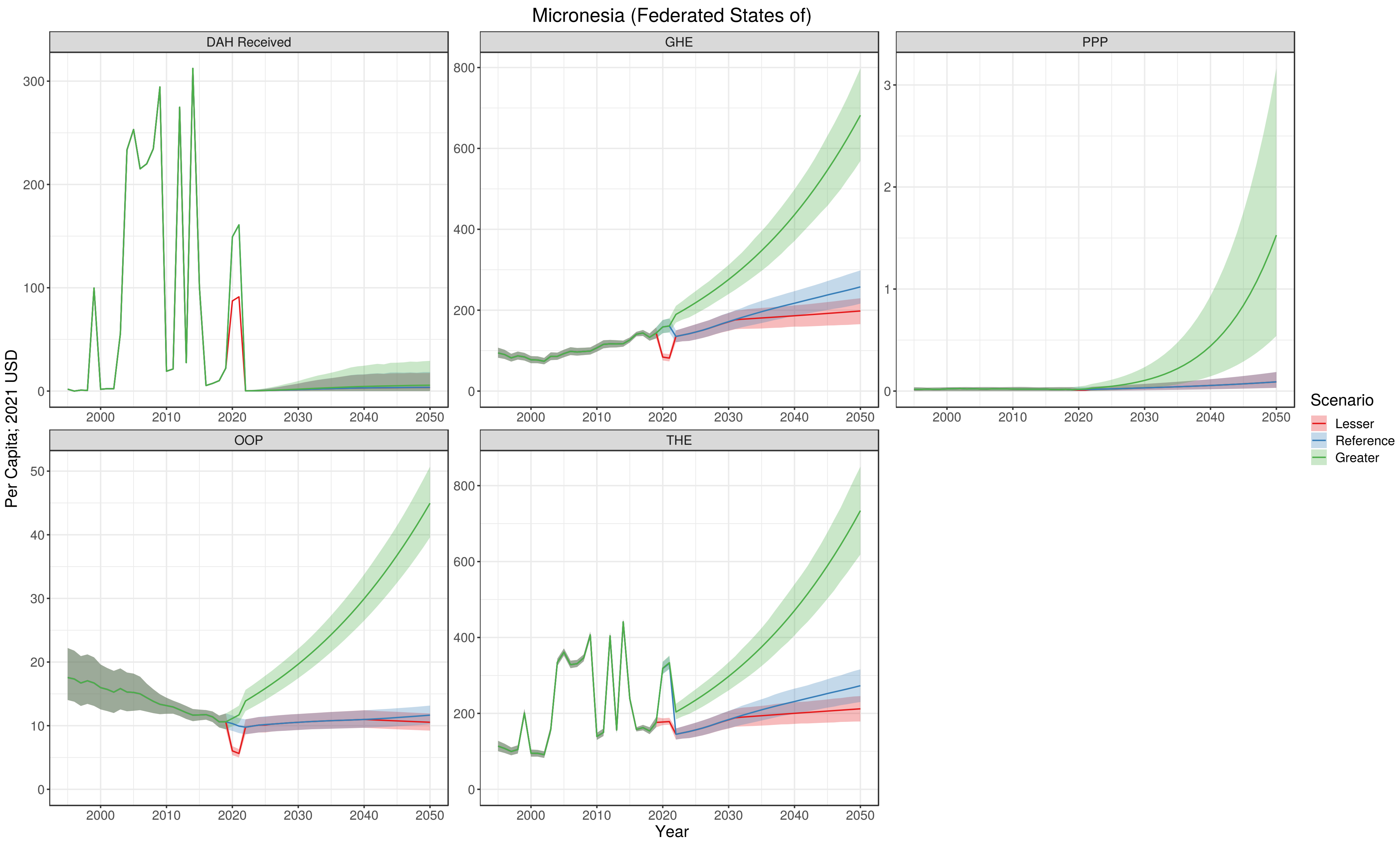

Micronesia (Federated States of)
DAH Received
GHE
PPP
OOP
THE
Per Capita; 2021 USD
Year
Scenario
Lesser
Reference
Greater

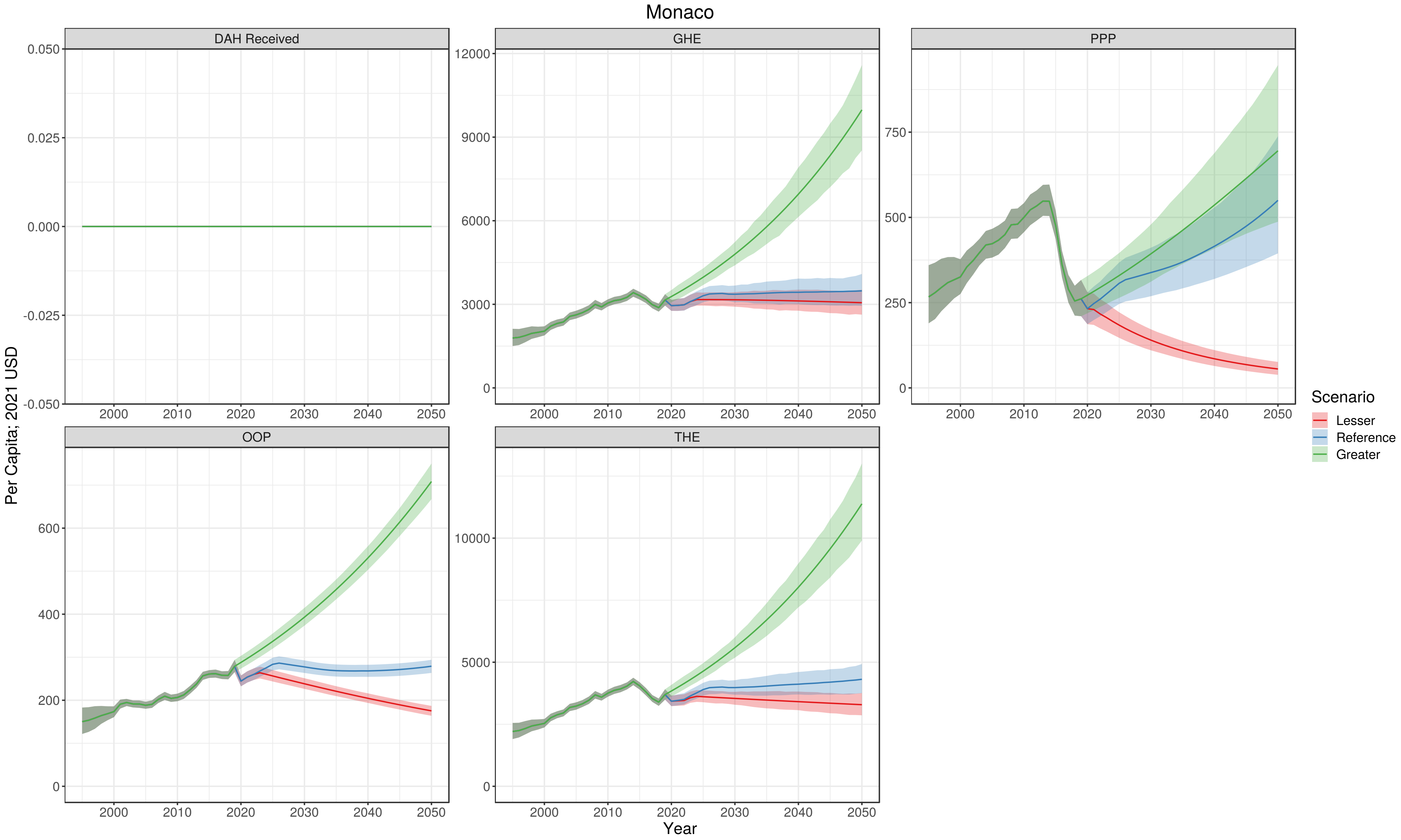

Monaco
DAH Received
GHE
PPP
OOP
THE
Per Capita; 2021 USD
Year
Scenario
Lesser
Reference
Greater

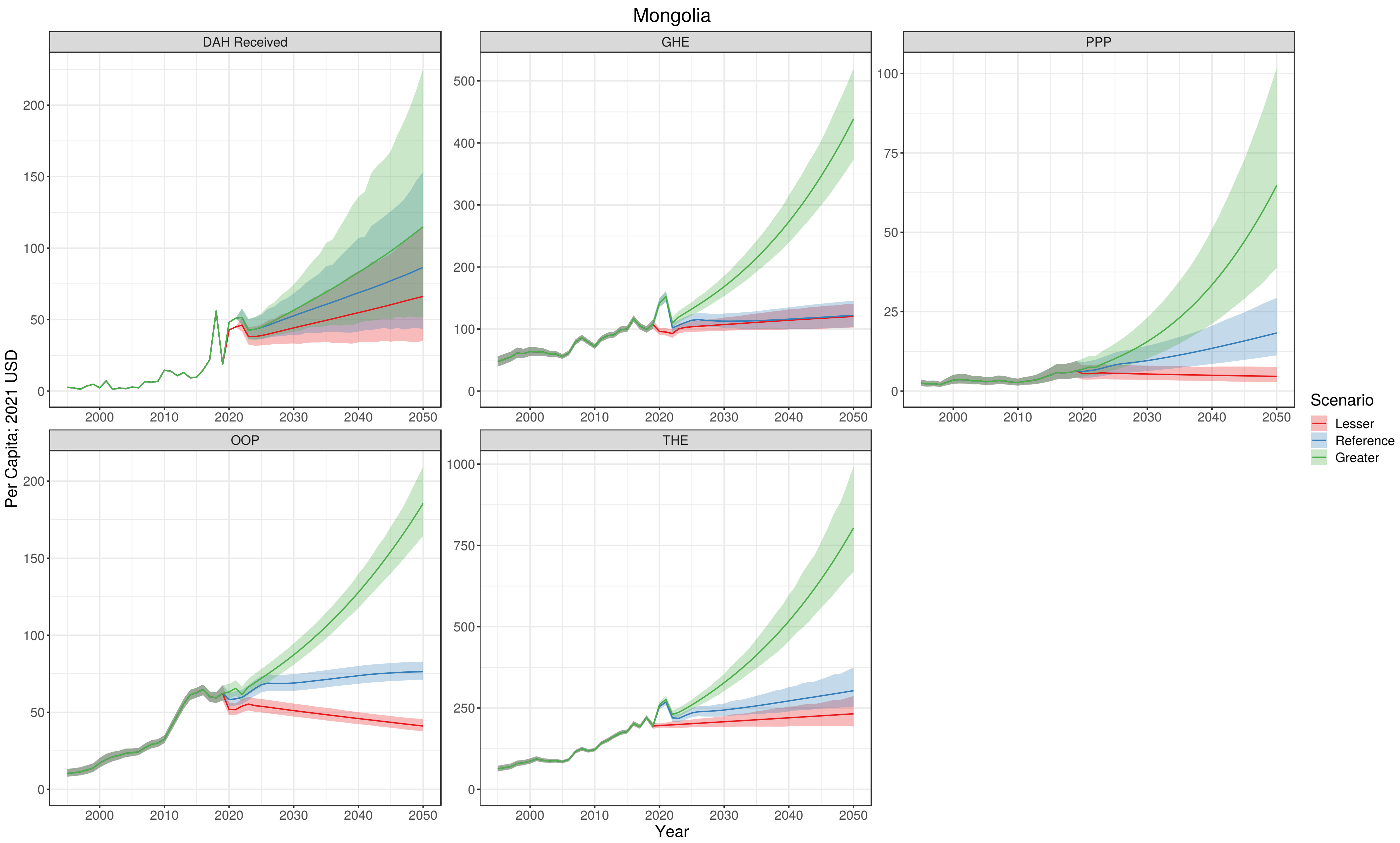

Mongolia
DAH Received
GHE
PPP
OOP
THE
Per Capita; 2021 USD
Year
Scenario
Lesser
Reference
Greater

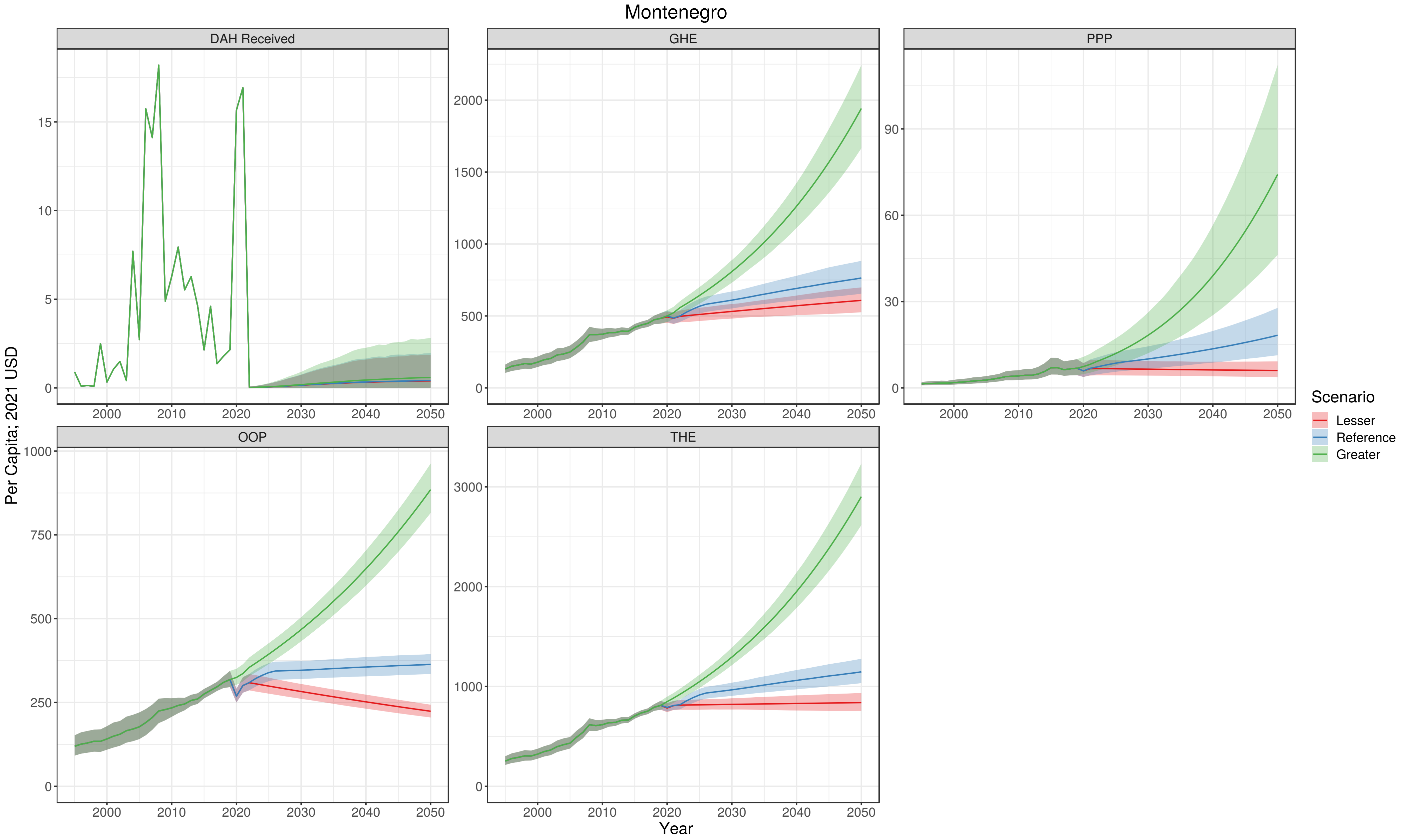

Montenegro
DAH Received
GHE
PPP
OOP
THE
Per Capita; 2021 USD
Year
Scenario
Lesser
Reference
Greater

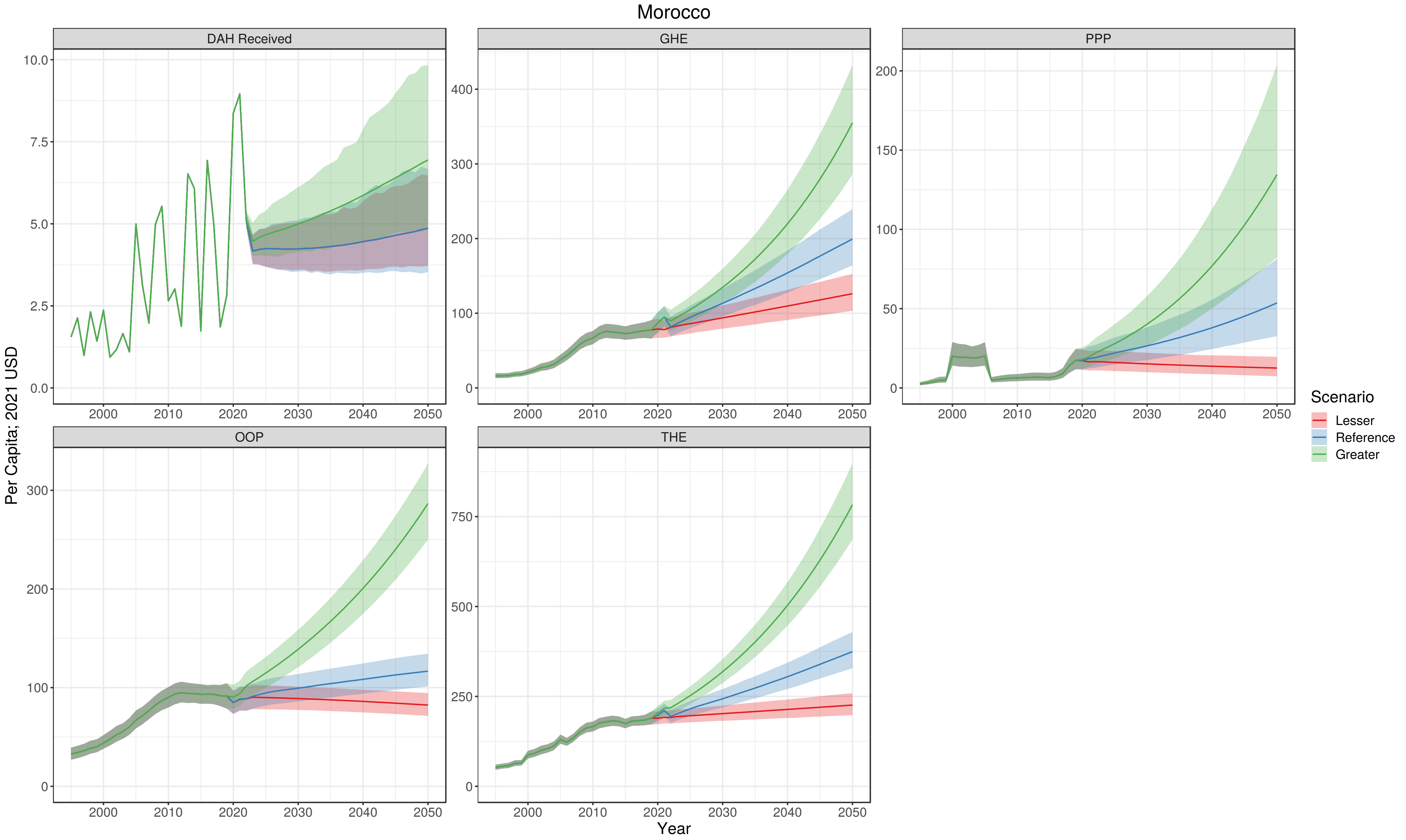

Morocco
DAH Received
GHE
PPP
OOP
THE
Per Capita; 2021 USD
Year
Scenario
Lesser
Reference
Greater

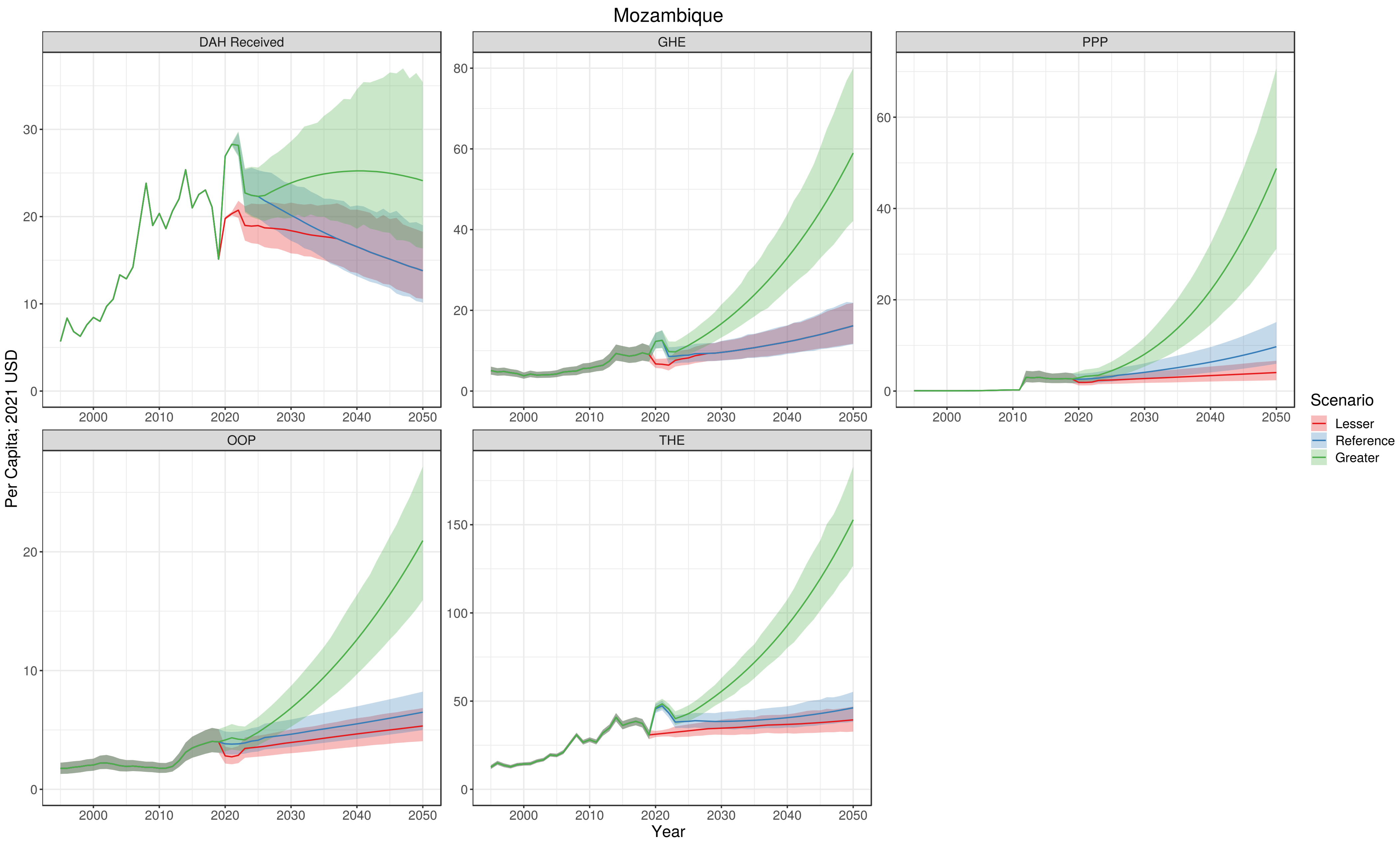

Mozambique
DAH Received
GHE
PPP
OOP
THE
Per Capita; 2021 USD
Year
Scenario
Lesser
Reference
Greater

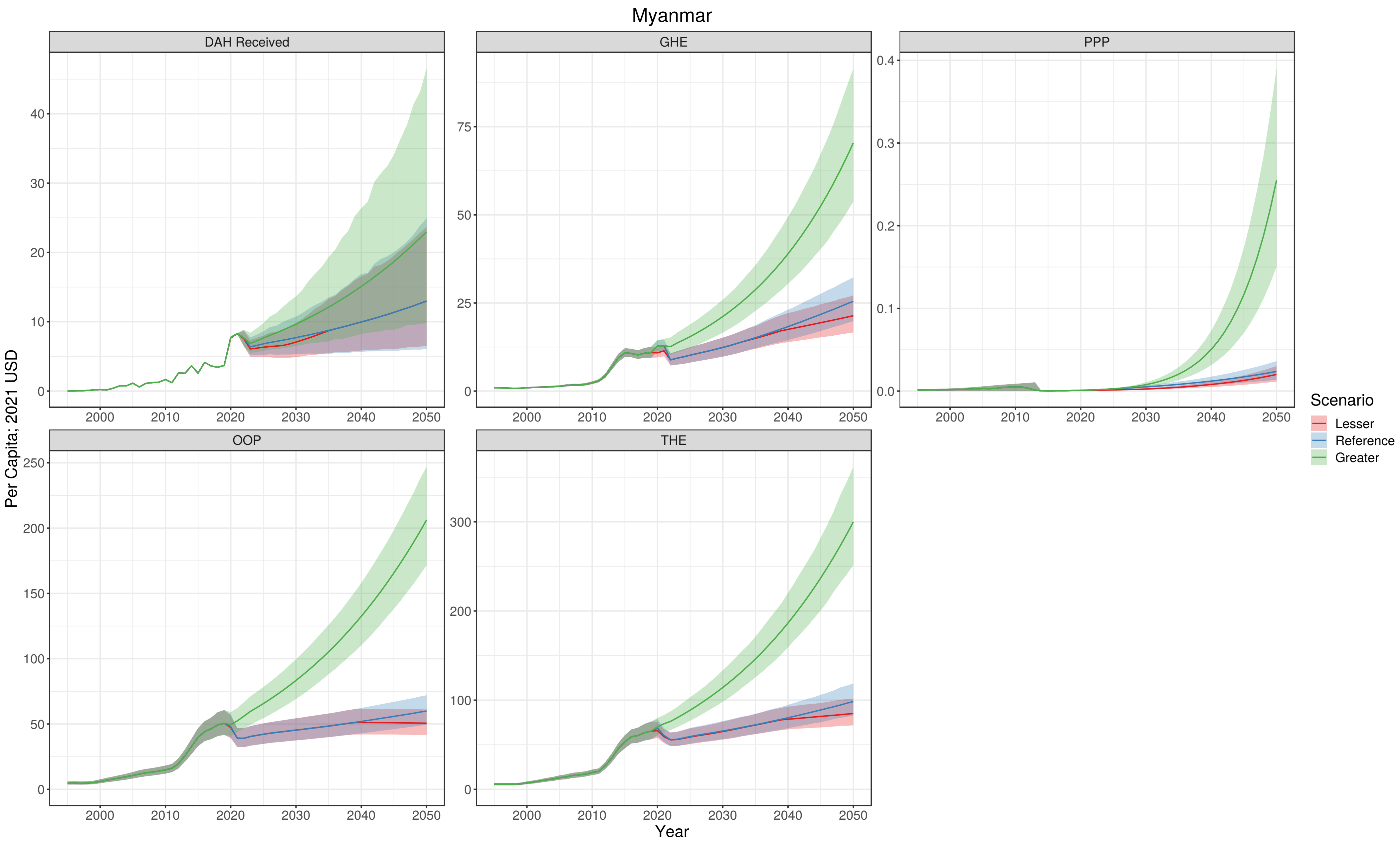

Myanmar
DAH Received
GHE
PPP
OOP
THE
Per Capita; 2021 USD
Year
Scenario
Lesser
Reference
Greater

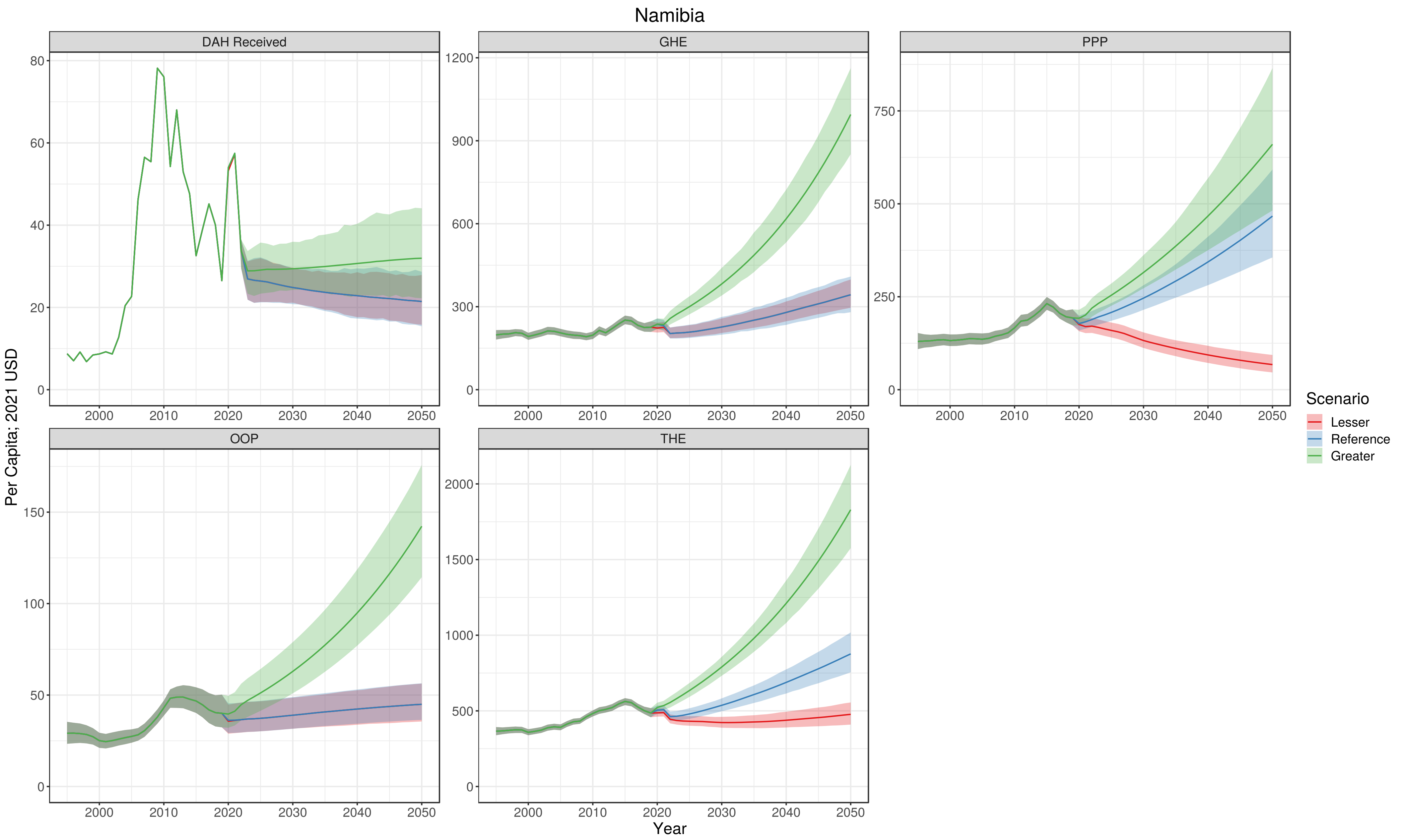

Namibia
DAH Received
GHE
PPP
OOP
THE
Per Capita; 2021 USD
Year
Scenario
Lesser
Reference
Greater

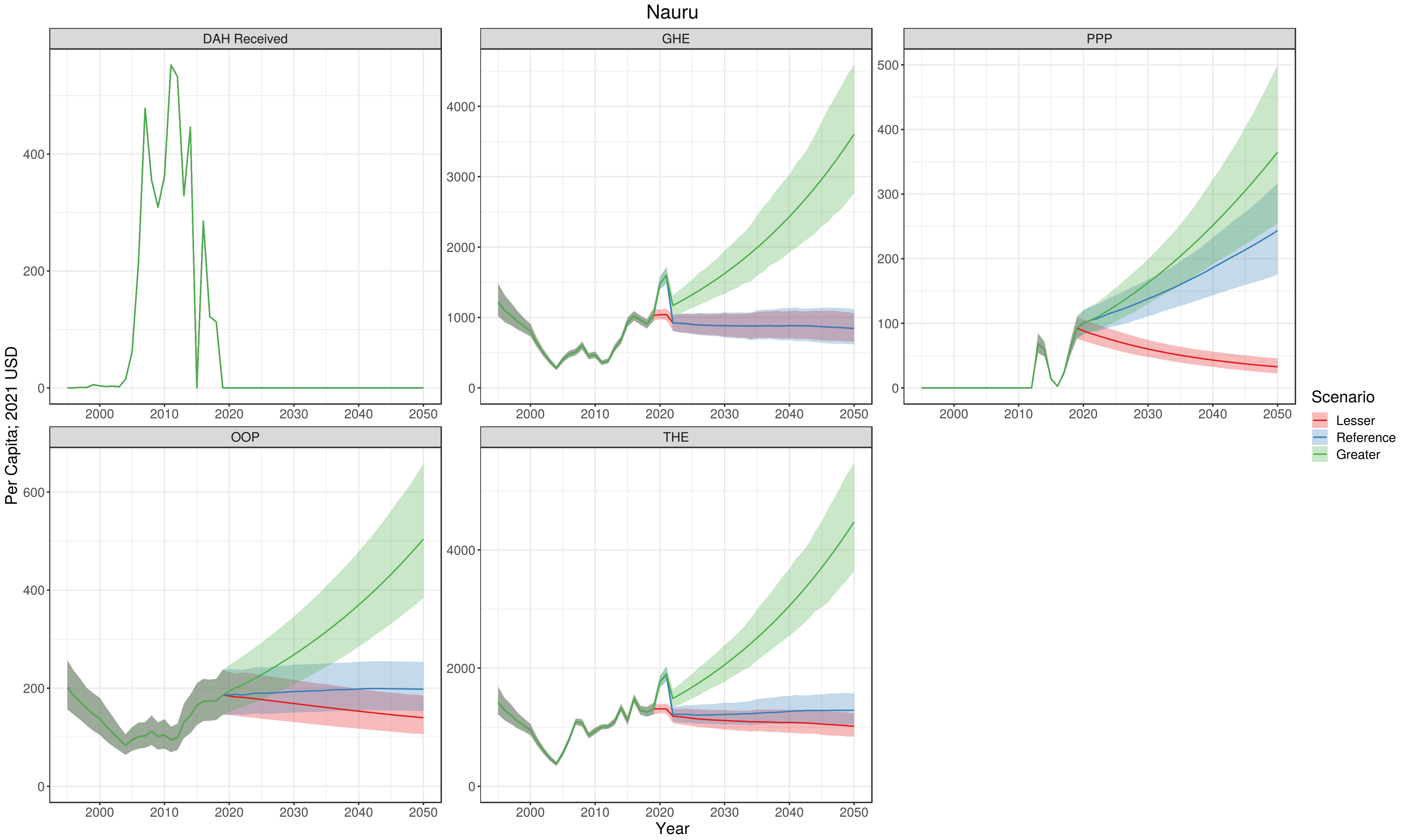

Nauru
DAH Received
GHE
PPP
OOP
THE
Per Capita; 2021 USD
Year
Scenario
Lesser
Reference
Greater

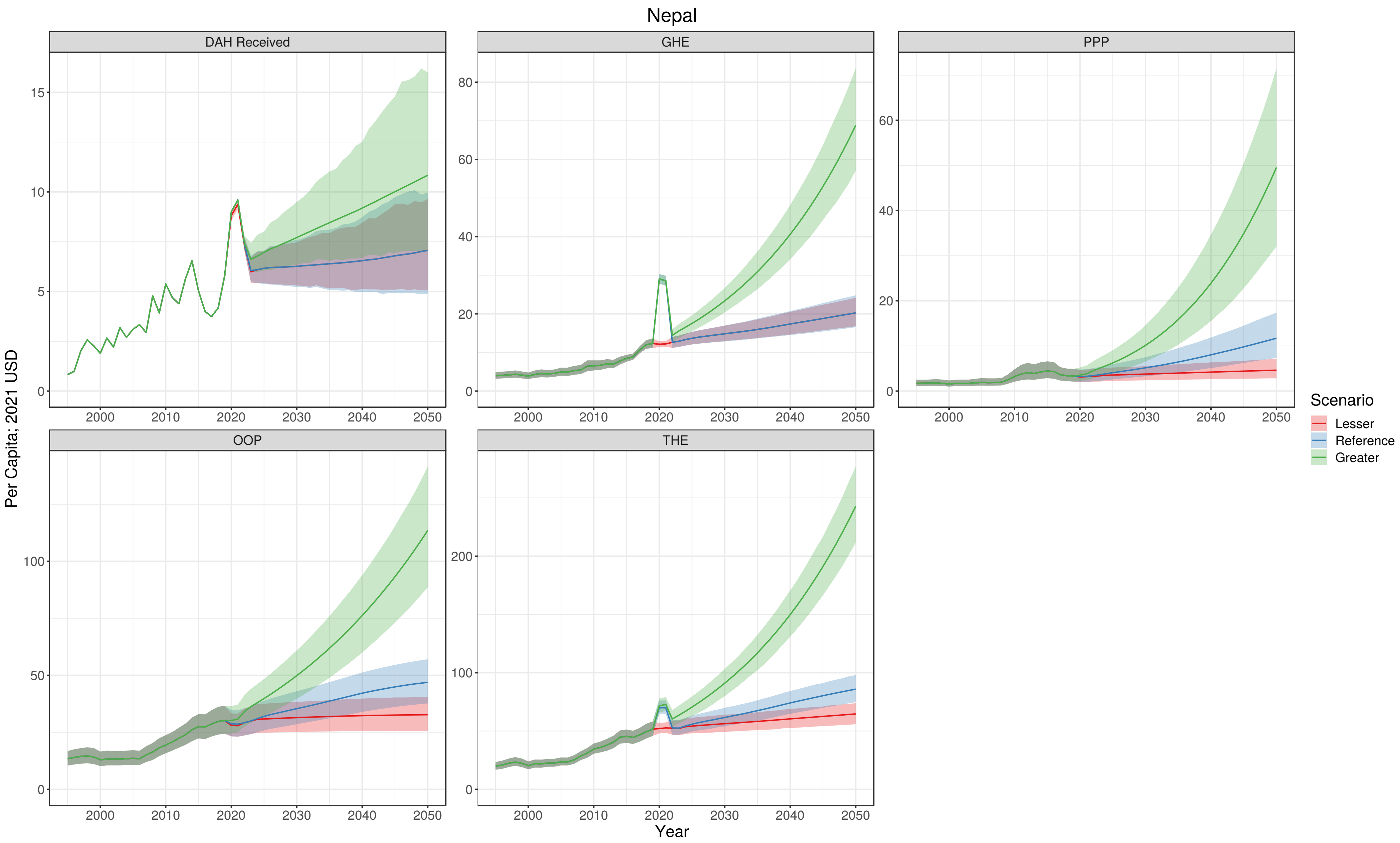

Nepal
DAH Received
GHE
PPP
OOP
THE
Per Capita; 2021 USD
Year
Scenario
Lesser
Reference
Greater
0
5
10
15
20
40
60
80
100
200
50
2000
2010
2020
2030
2040
2050

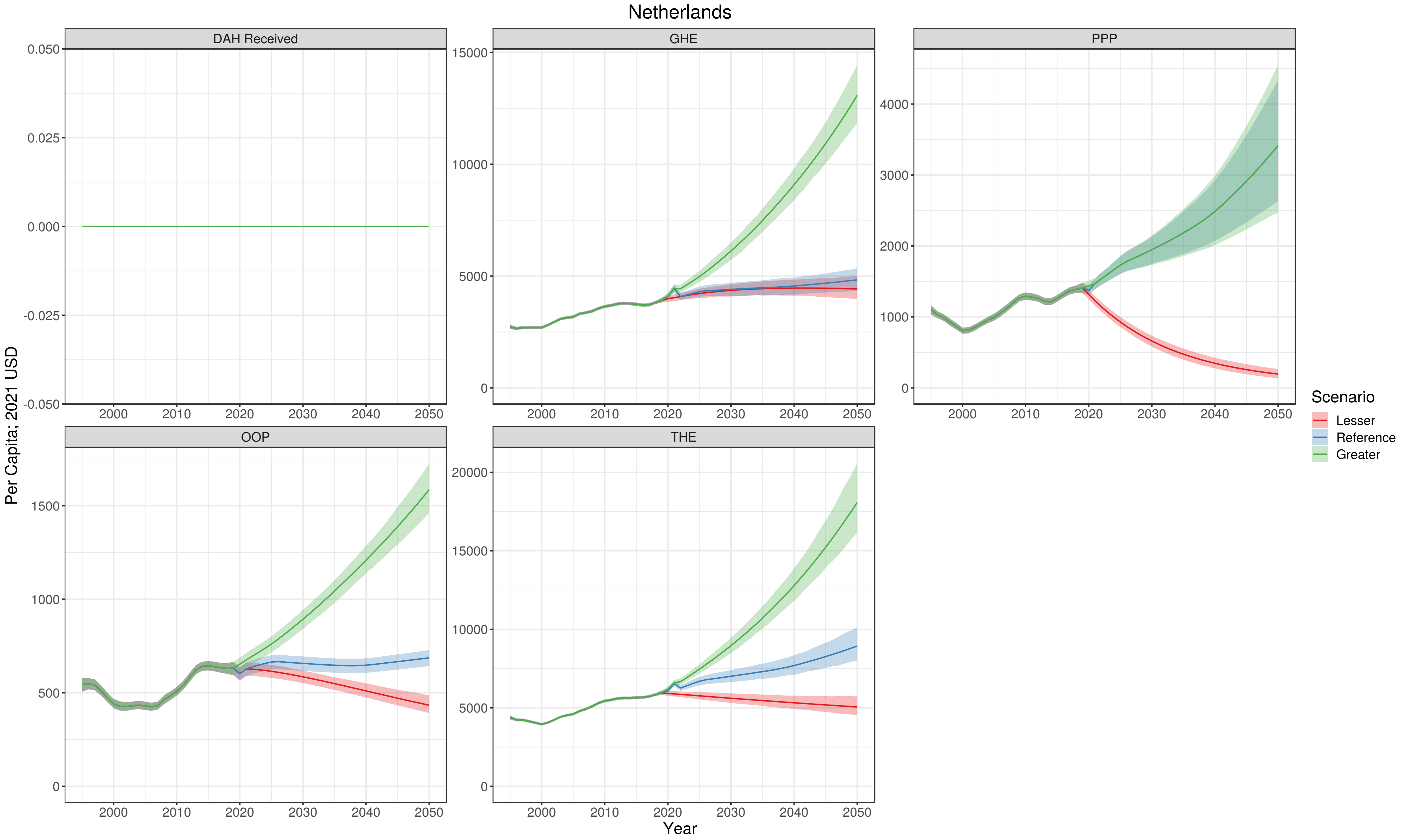

Netherlands
DAH Received
GHE
PPP
OOP
THE
Per Capita; 2021 USD
Year
Scenario
Lesser
Reference
Greater
0.050
0.025
0.000
-0.025
-0.050
15000
10000
5000
0
4000
3000
2000
1000
1500
1000
500
20000
2000
2010
2020
2030
2040
2050

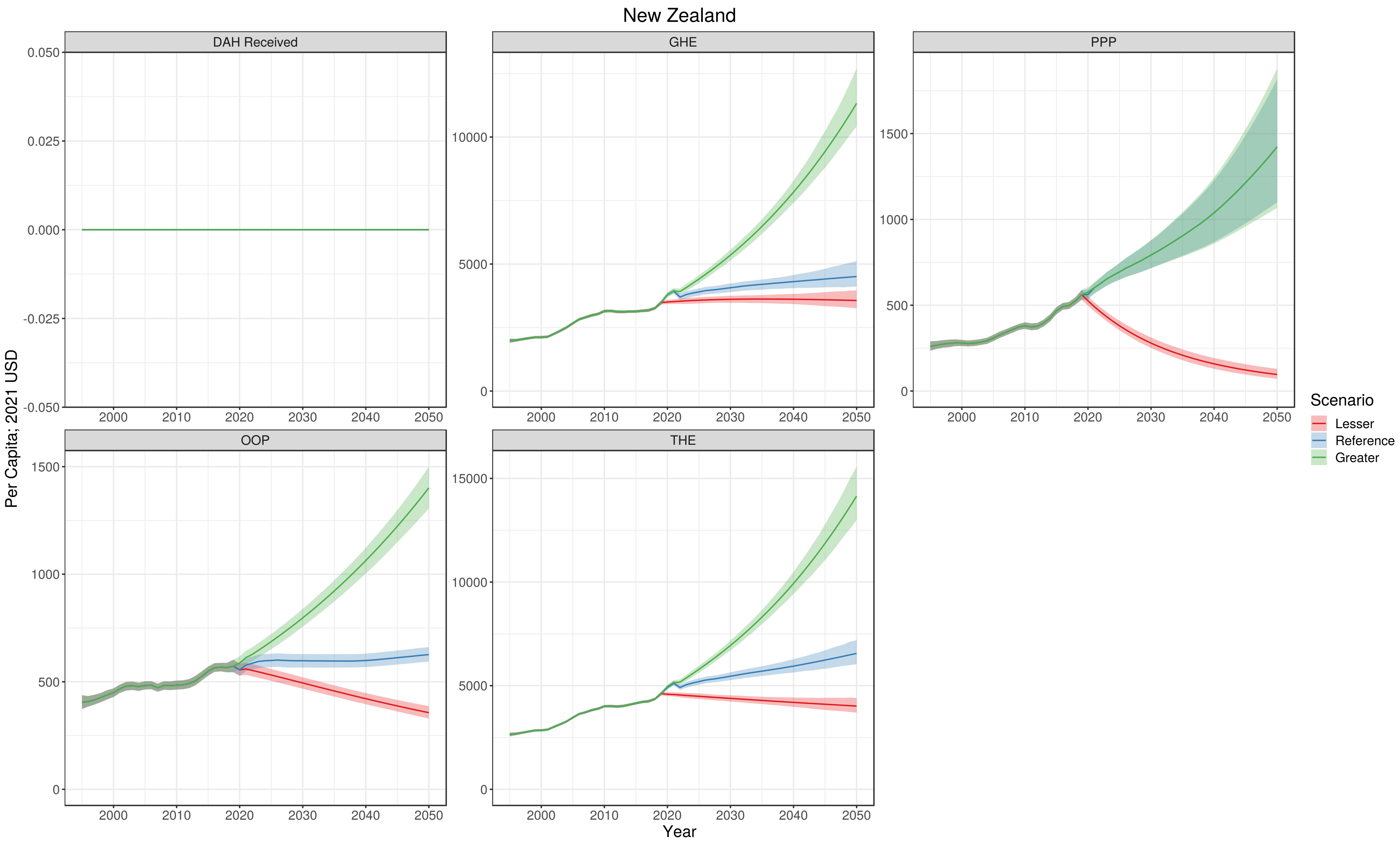

New Zealand
DAH Received
GHE
PPP
OOP
THE
Per Capita; 2021 USD
Year
Scenario
Lesser
Reference
Greater
0.050
0.025
0.000
-0.025
-0.050
10000
5000
0
1500
1000
500
15000
2000
2010
2020
2030
2040
2050

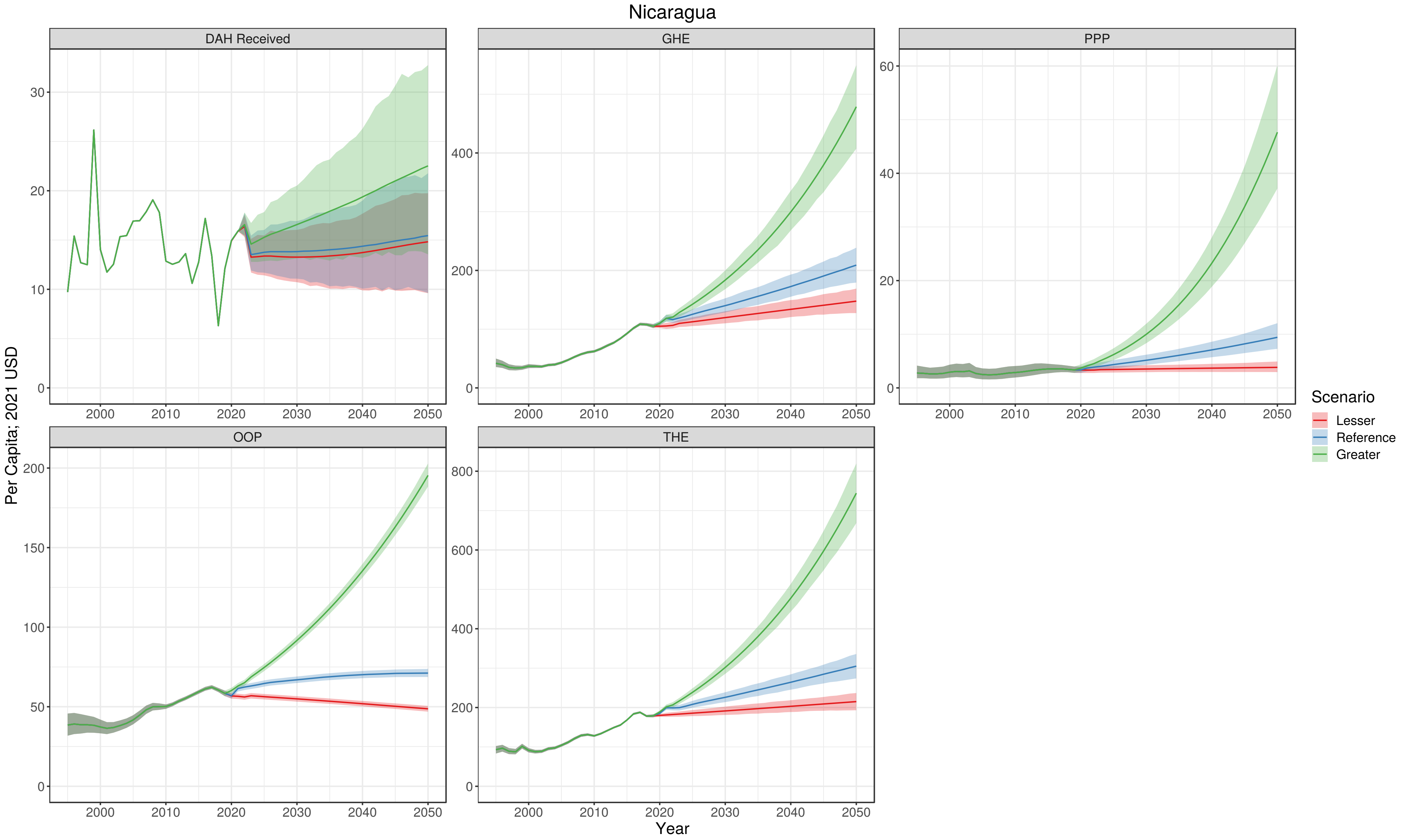

Nicaragua
DAH Received
GHE
PPP
OOP
THE
Per Capita; 2021 USD
Year
Scenario
Lesser
Reference
Greater

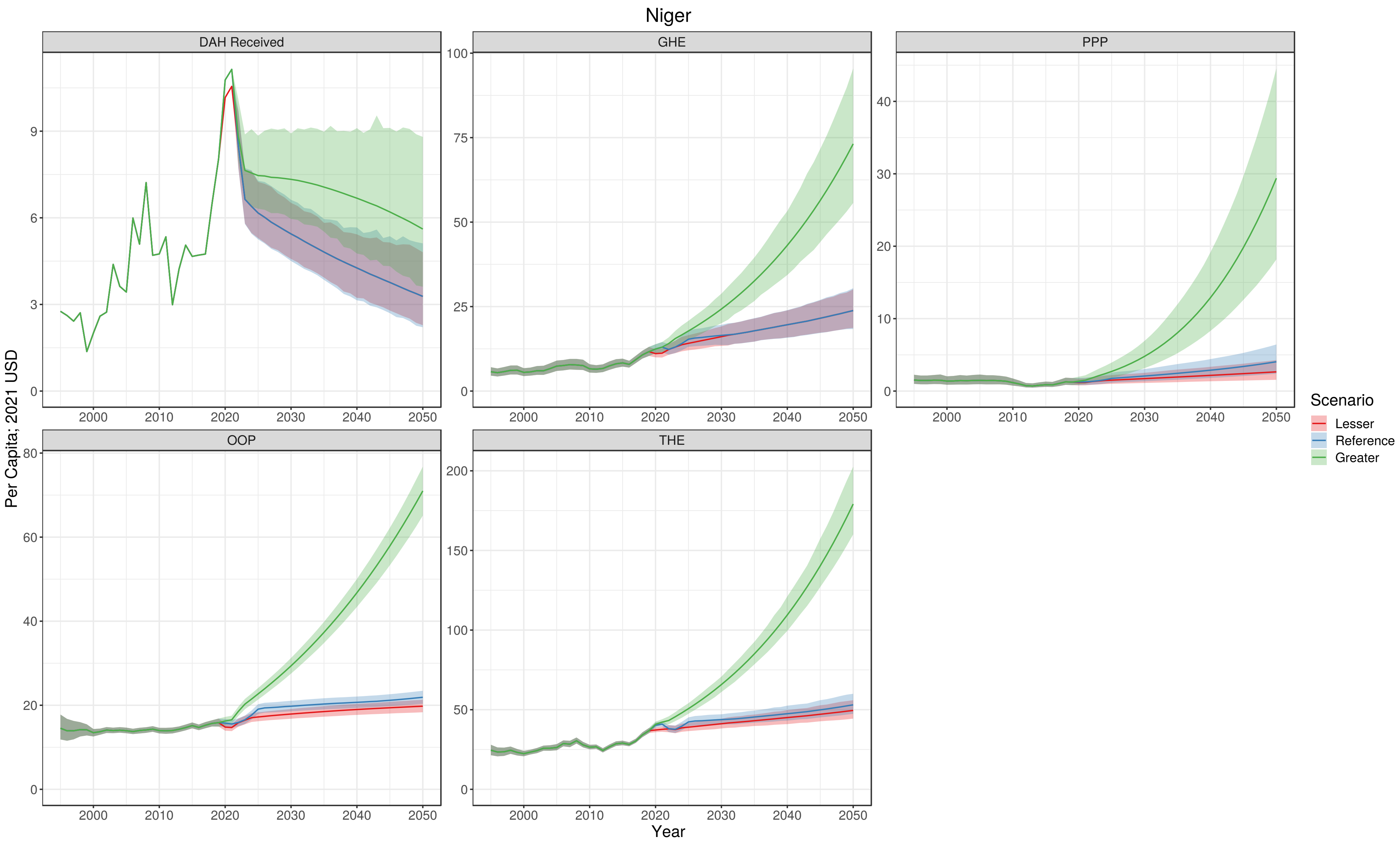
Niger
DAH Received
GHE
PPP
OOP
THE
Per Capita; 2021 USD
Year
Scenario
Lesser
Reference
Greater
0
3
6
9
25
50
75
100
10
20
30
40
60
80
150
200
2000
2010
2020
2030
2040
2050

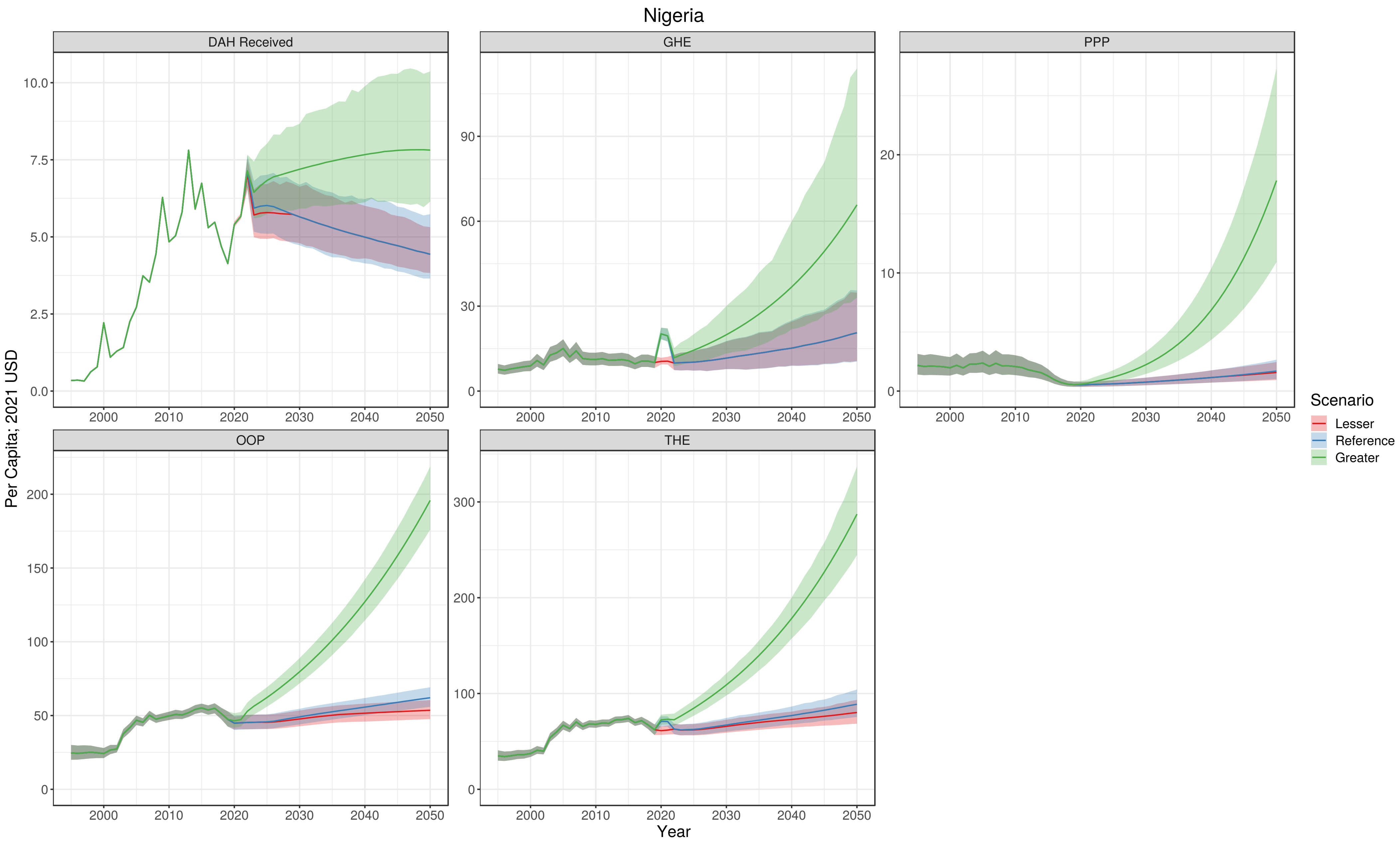

Nigeria
DAH Received
GHE
PPP
OOP
THE
Per Capita; 2021 USD
Year
Scenario
Lesser
Reference
Greater

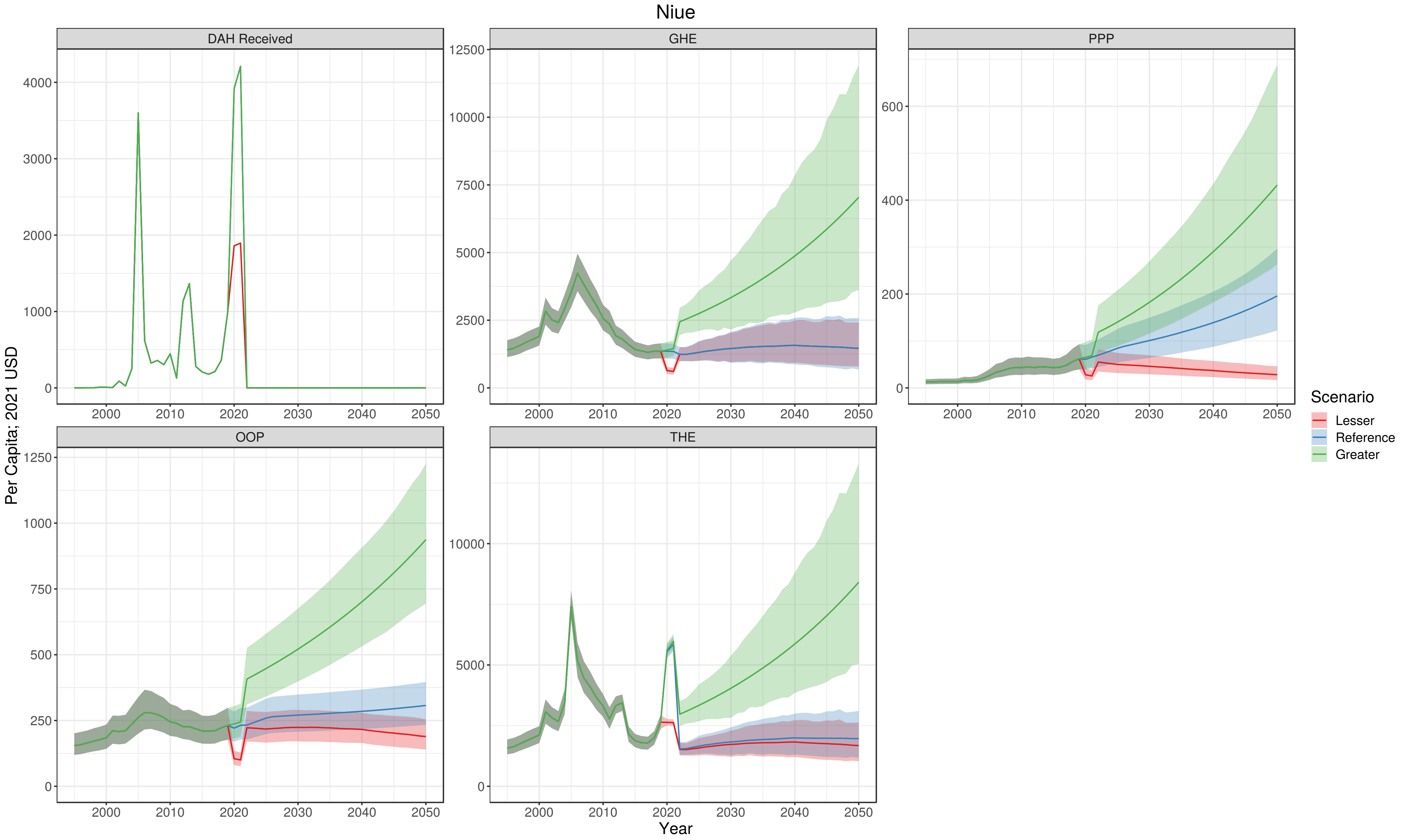

Niue
DAH Received
GHE
PPP
OOP
THE
Per Capita; 2021 USD
Year
Scenario
Lesser
Reference
Greater

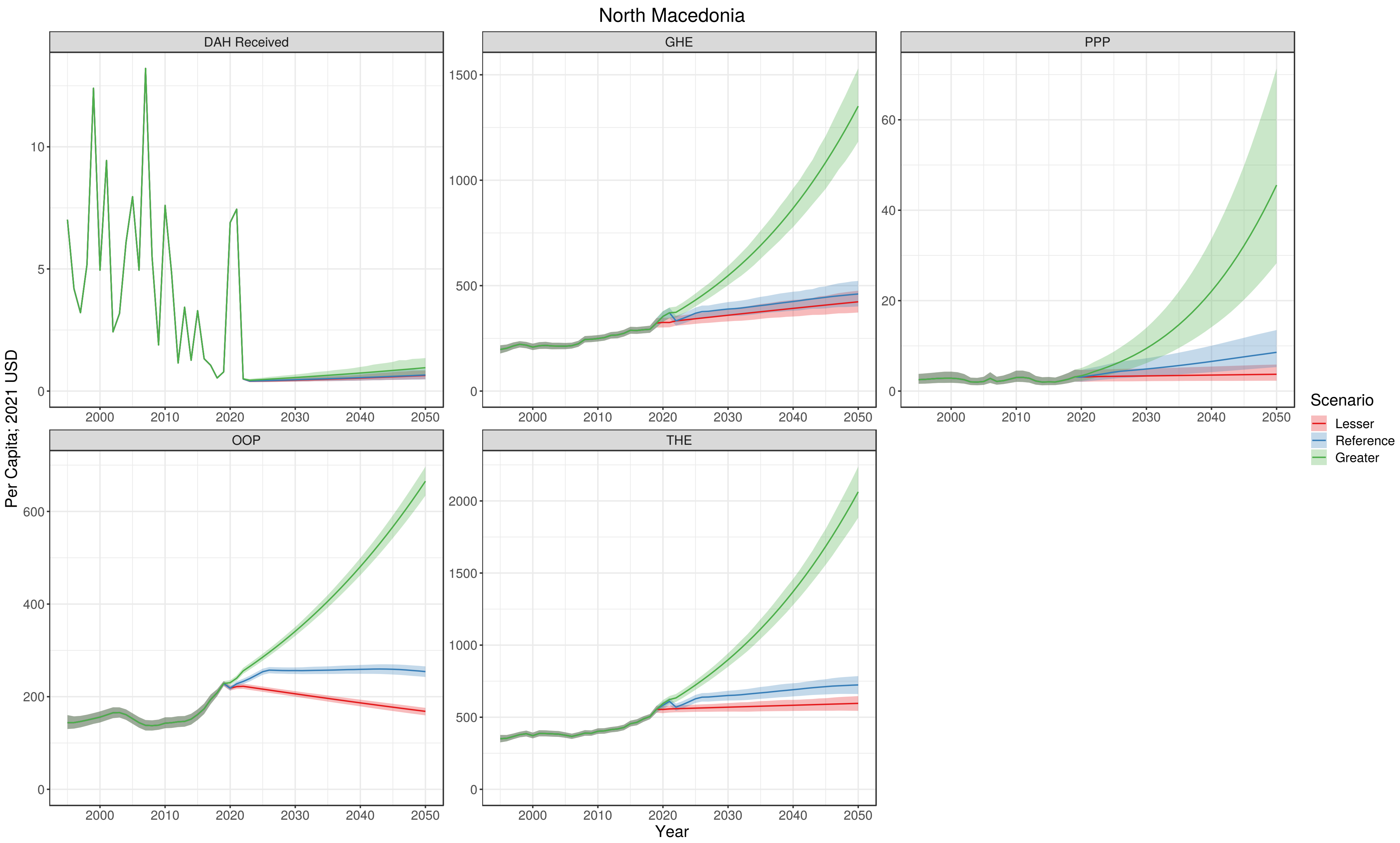
North Macedonia
DAH Received
GHE
PPP
OOP
THE
Per Capita; 2021 USD
Year
Scenario
Lesser
Reference
Greater
0
5
10
500
1000
1500
20
40
60
200
400
600
2000
2010
2020
2030
2040
2050

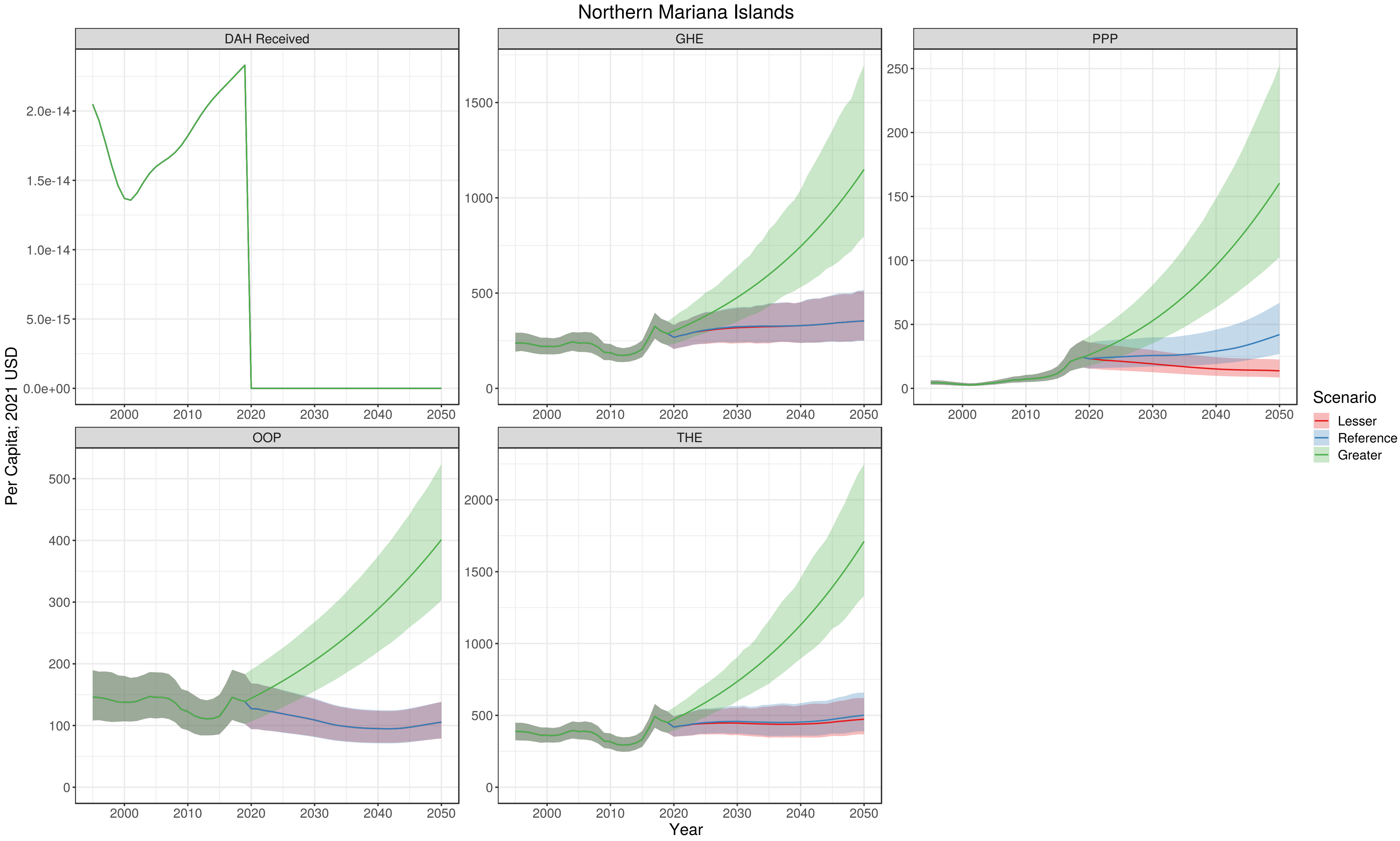

Northern Mariana Islands
DAH Received
GHE
PPP
OOP
THE
Per Capita; 2021 USD
Year
Scenario
Lesser
Reference
Greater

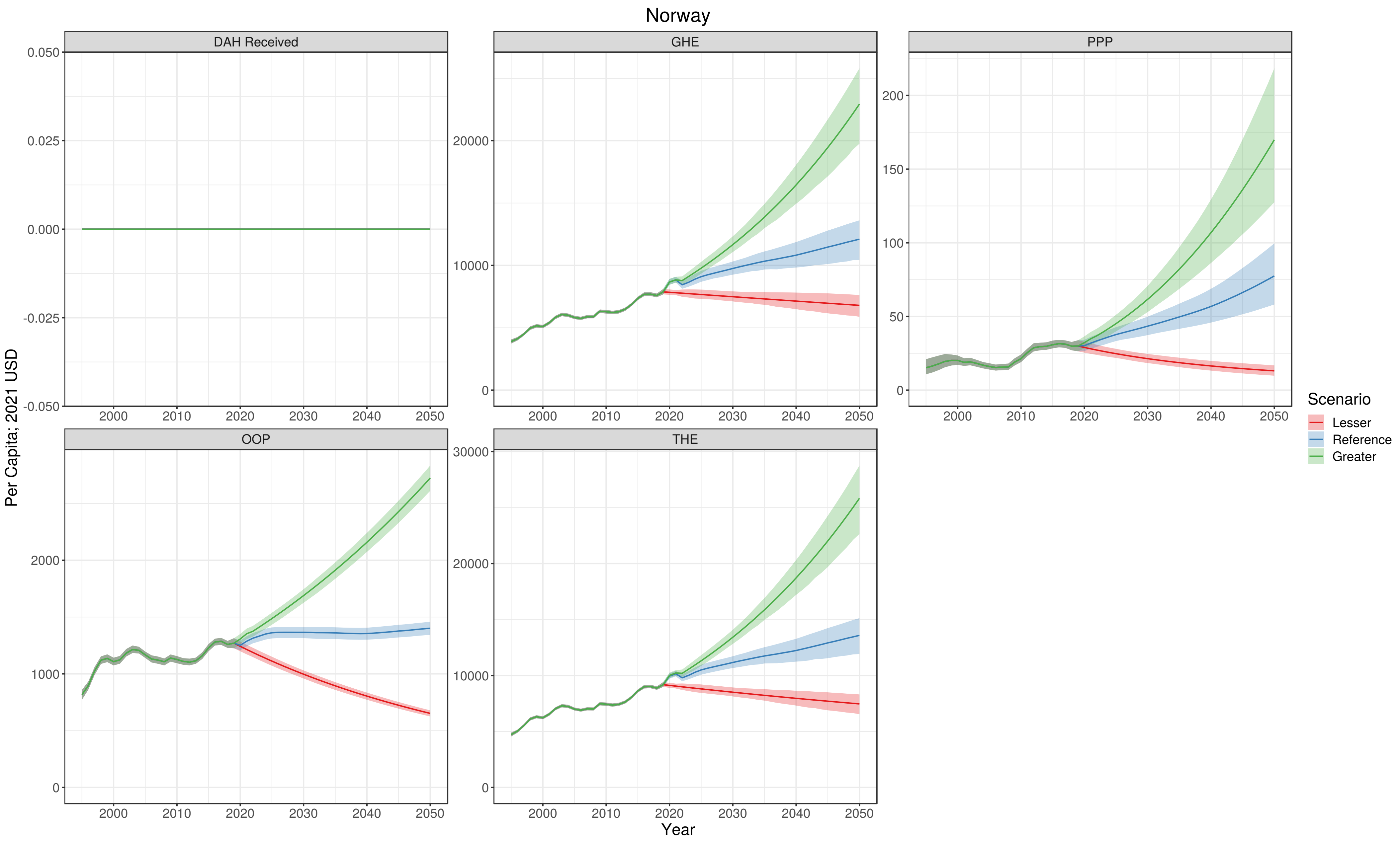

Norway
DAH Received
GHE
PPP
OOP
THE
Per Capita; 2021 USD
Year
Scenario
Lesser
Reference
Greater
0.050
0.025
0.000
-0.025
-0.050
20000
10000
0
200
150
100
50
2000
1000
30000
2000
2010
2020
2030
2040
2050

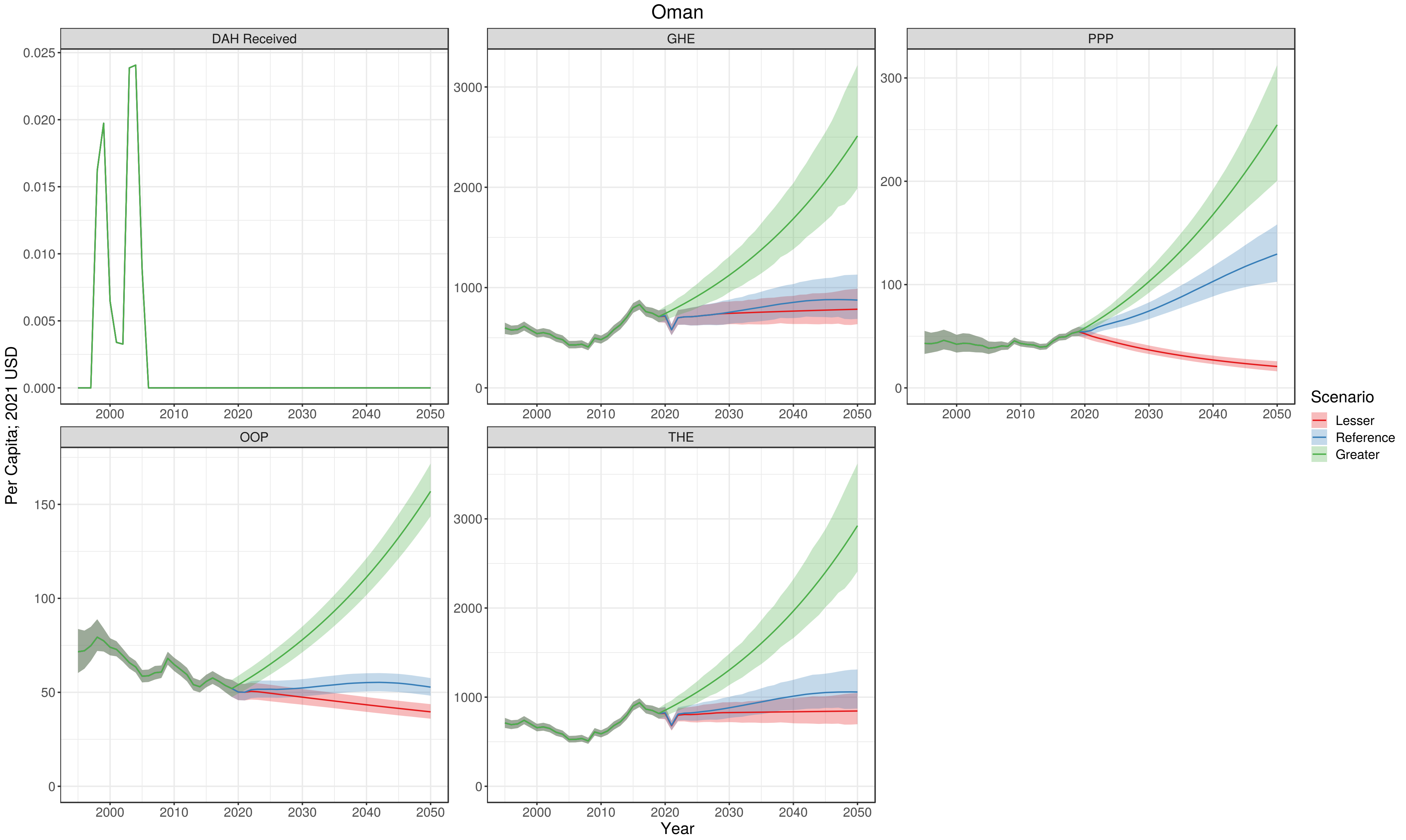

Oman
DAH Received
GHE
PPP
OOP
THE
Per Capita; 2021 USD
Year
Scenario
Lesser
Reference
Greater

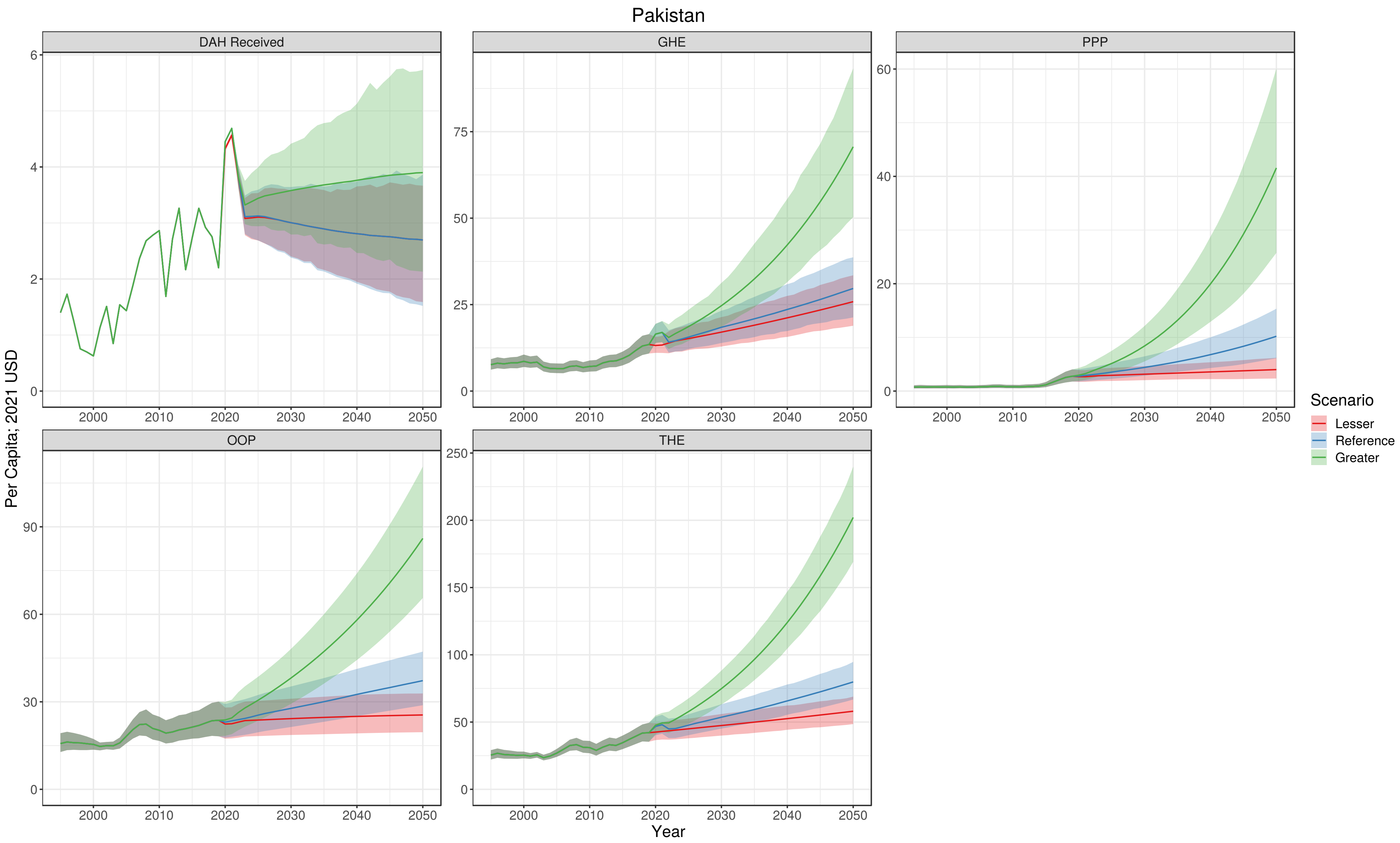
Pakistan
DAH Received
GHE
PPP
OOP
THE
Per Capita; 2021 USD
Year
Scenario
Lesser
Reference
Greater
2000
2010
2020
2030
2040
2050
0
2
4
6
25
50
75
20
40
60
30
60
90
100
150
200
250

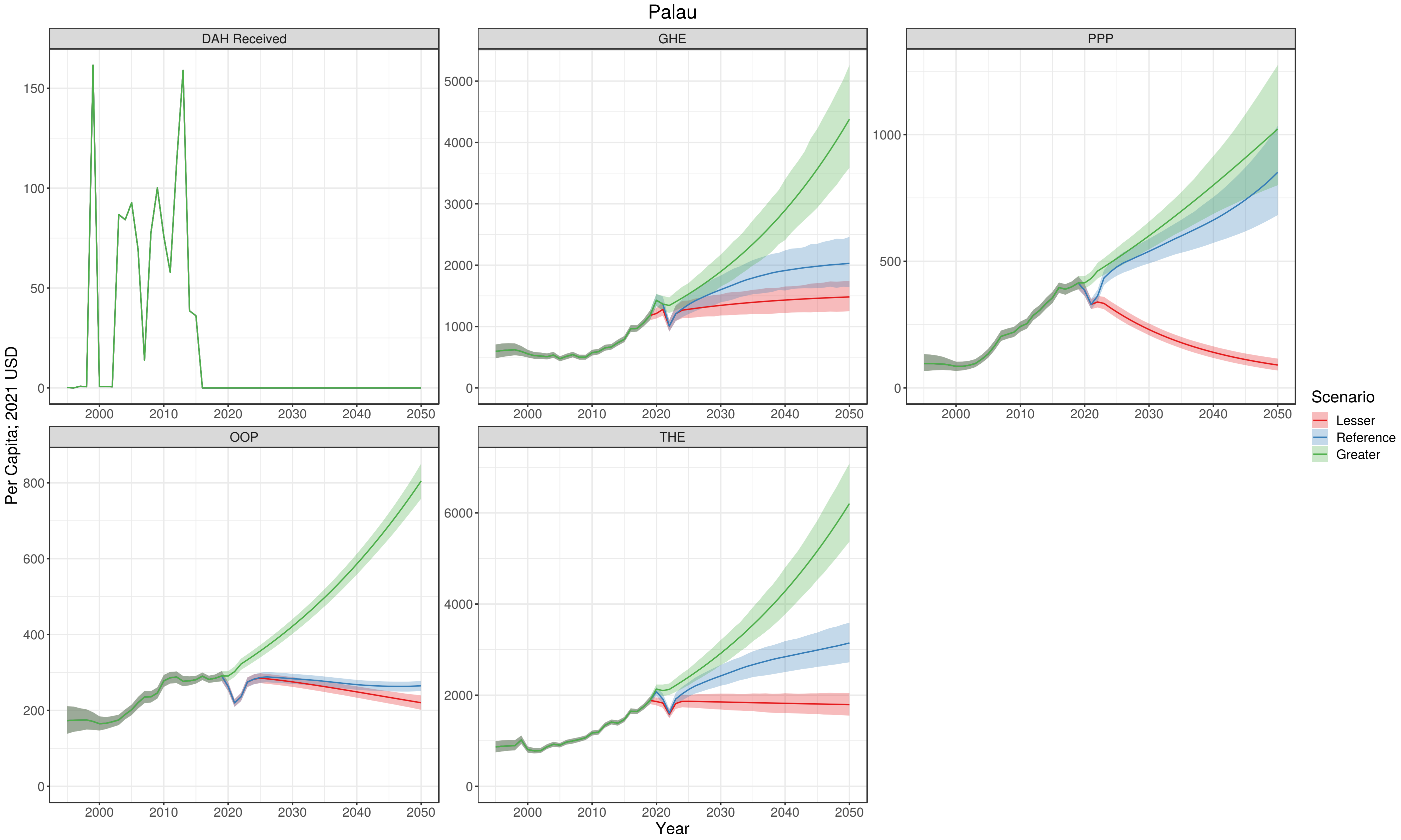
Palau
DAH Received
GHE
PPP
OOP
THE
Per Capita; 2021 USD
Year
Scenario
Lesser
Reference
Greater
0
50
100
150
1000
2000
3000
4000
5000
500
200
400
600
800
6000
2000
2010
2020
2030
2040
2050

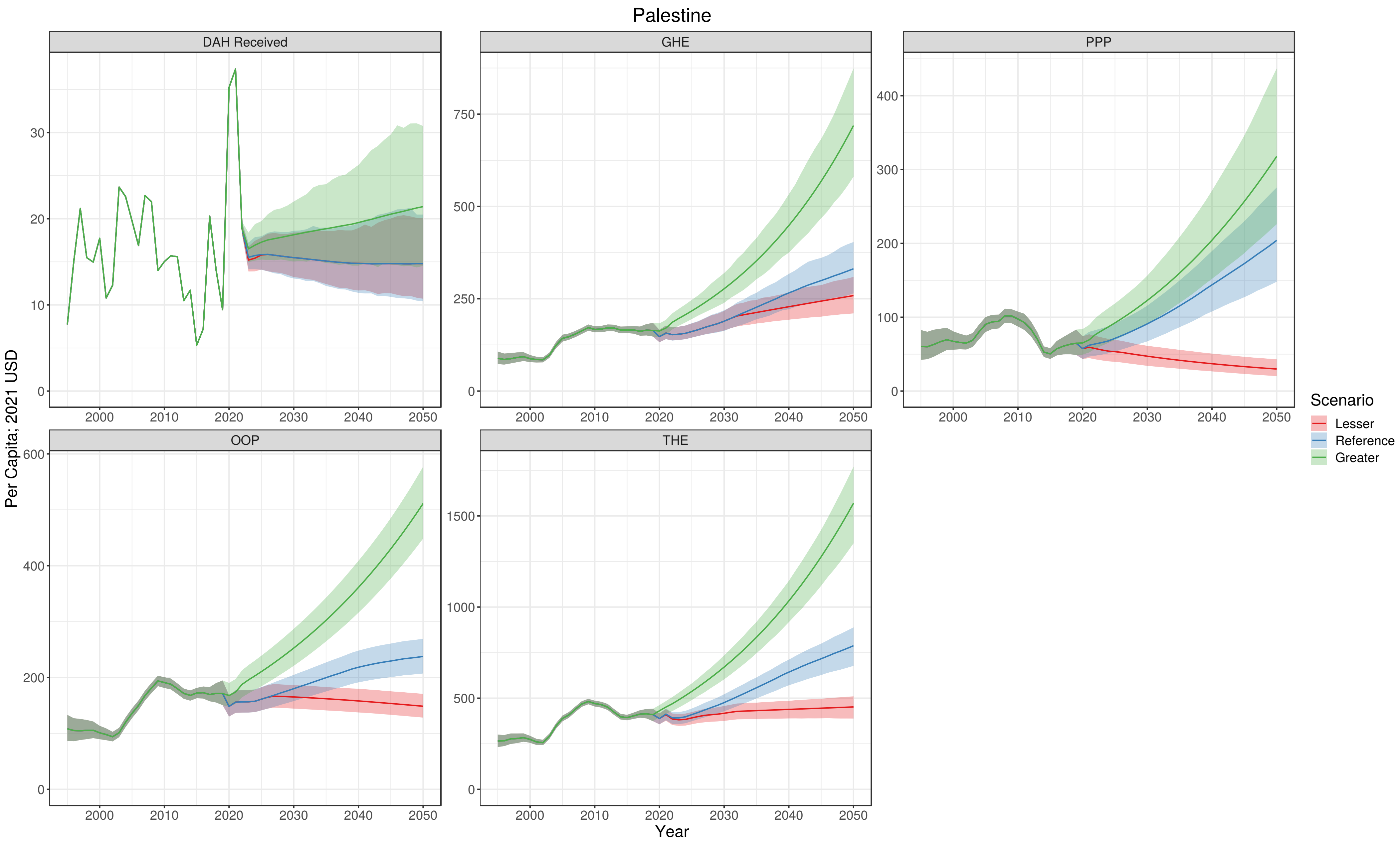

Palestine
DAH Received
GHE
PPP
OOP
THE
Per Capita; 2021 USD
Year
Scenario
Lesser
Reference
Greater

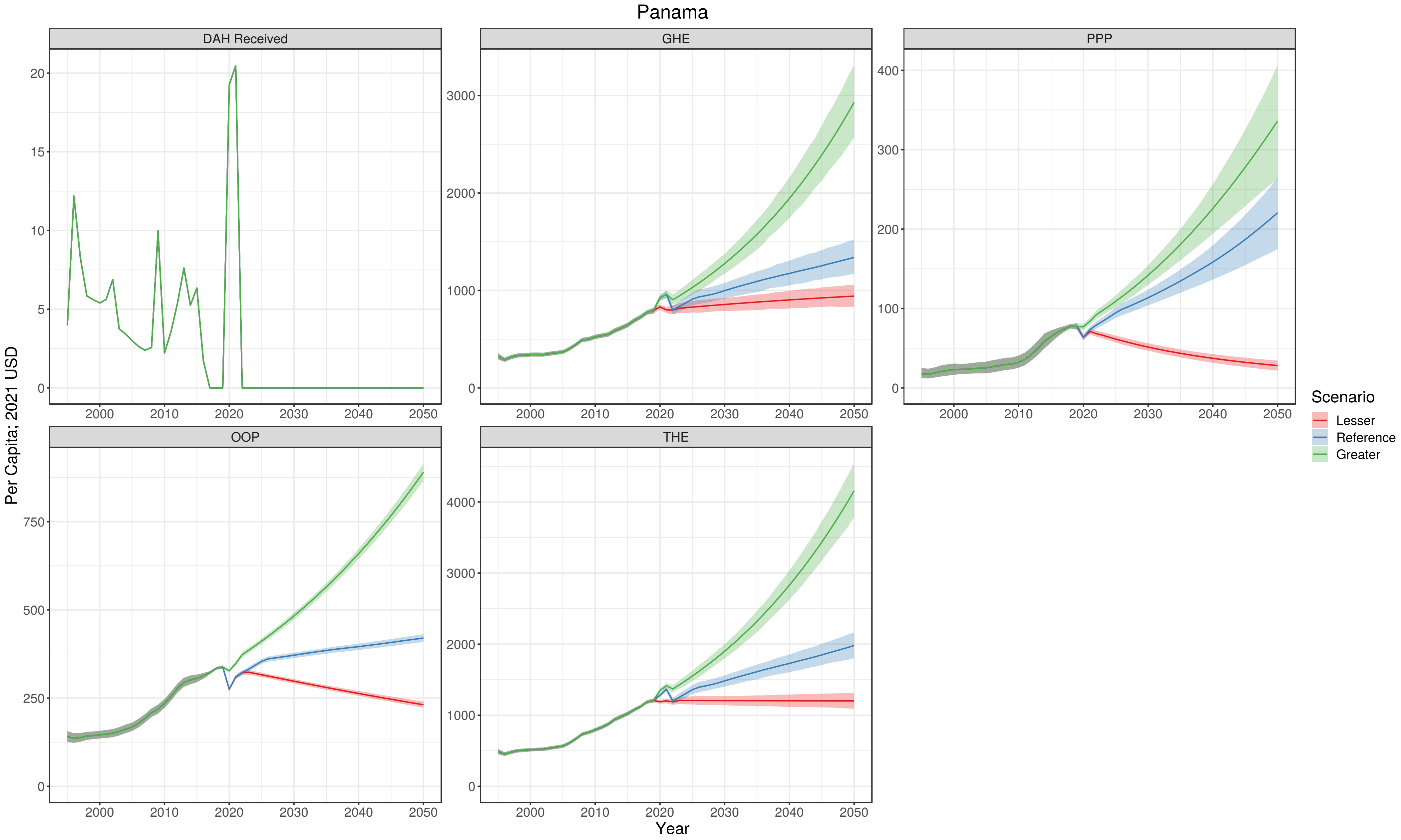

Panama
DAH Received
GHE
PPP
OOP
THE
Per Capita; 2021 USD
Year
Scenario
Lesser
Reference
Greater

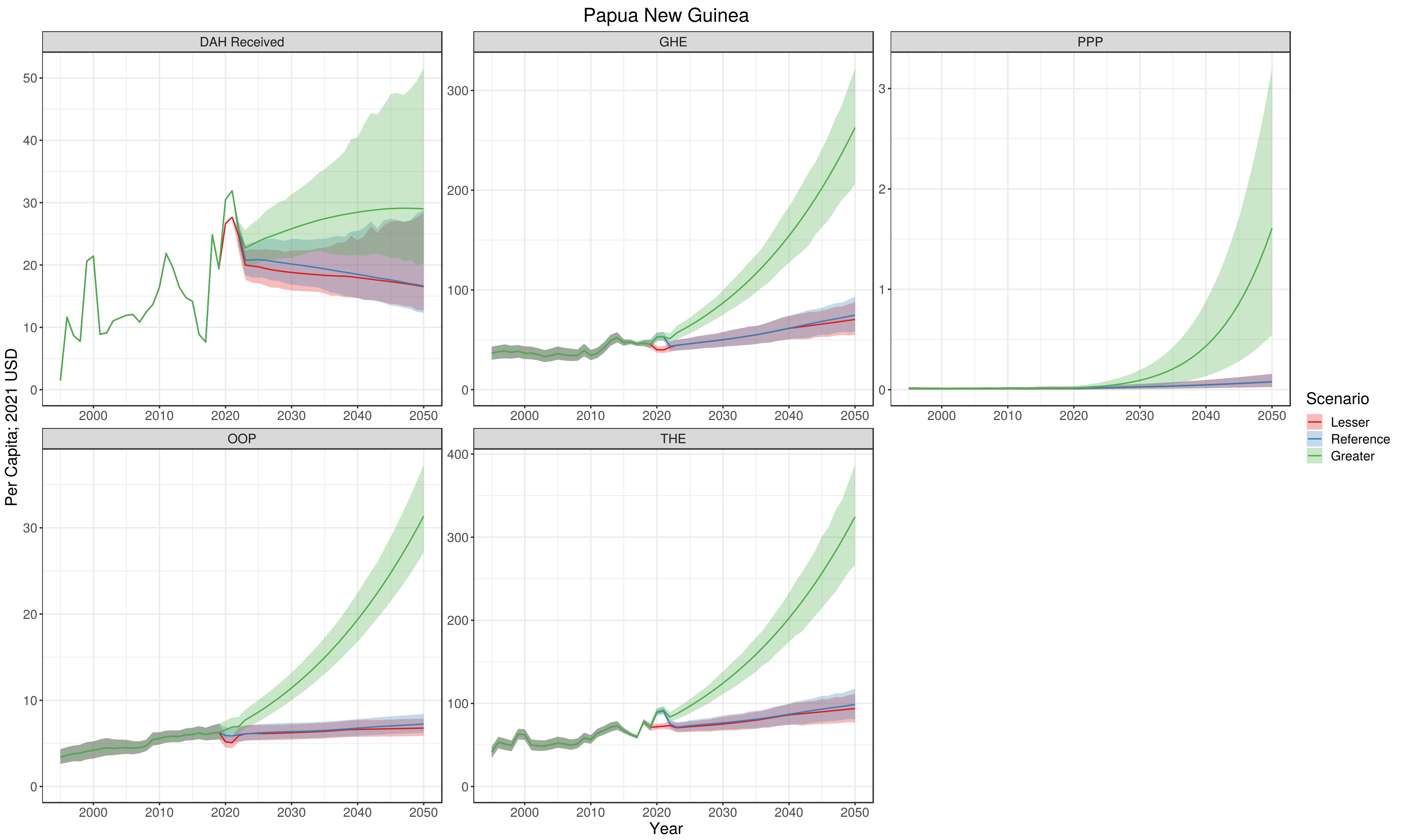
Papua New Guinea
DAH Received
GHE
PPP
OOP
THE
Per Capita; 2021 USD
Year
Scenario
Lesser
Reference
Greater

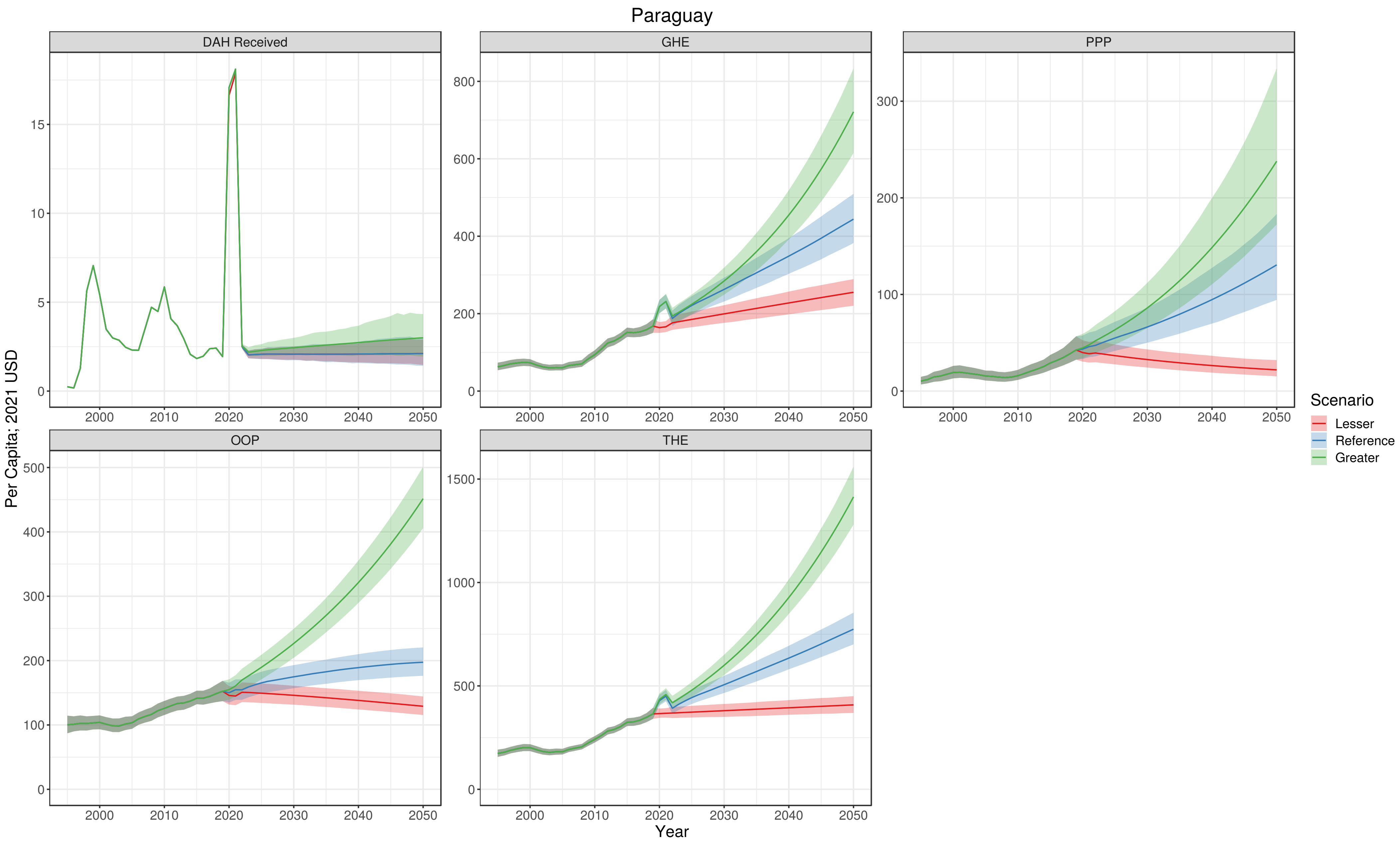

Paraguay
DAH Received
GHE
PPP
OOP
THE
Per Capita; 2021 USD
Year
Scenario
Lesser
Reference
Greater

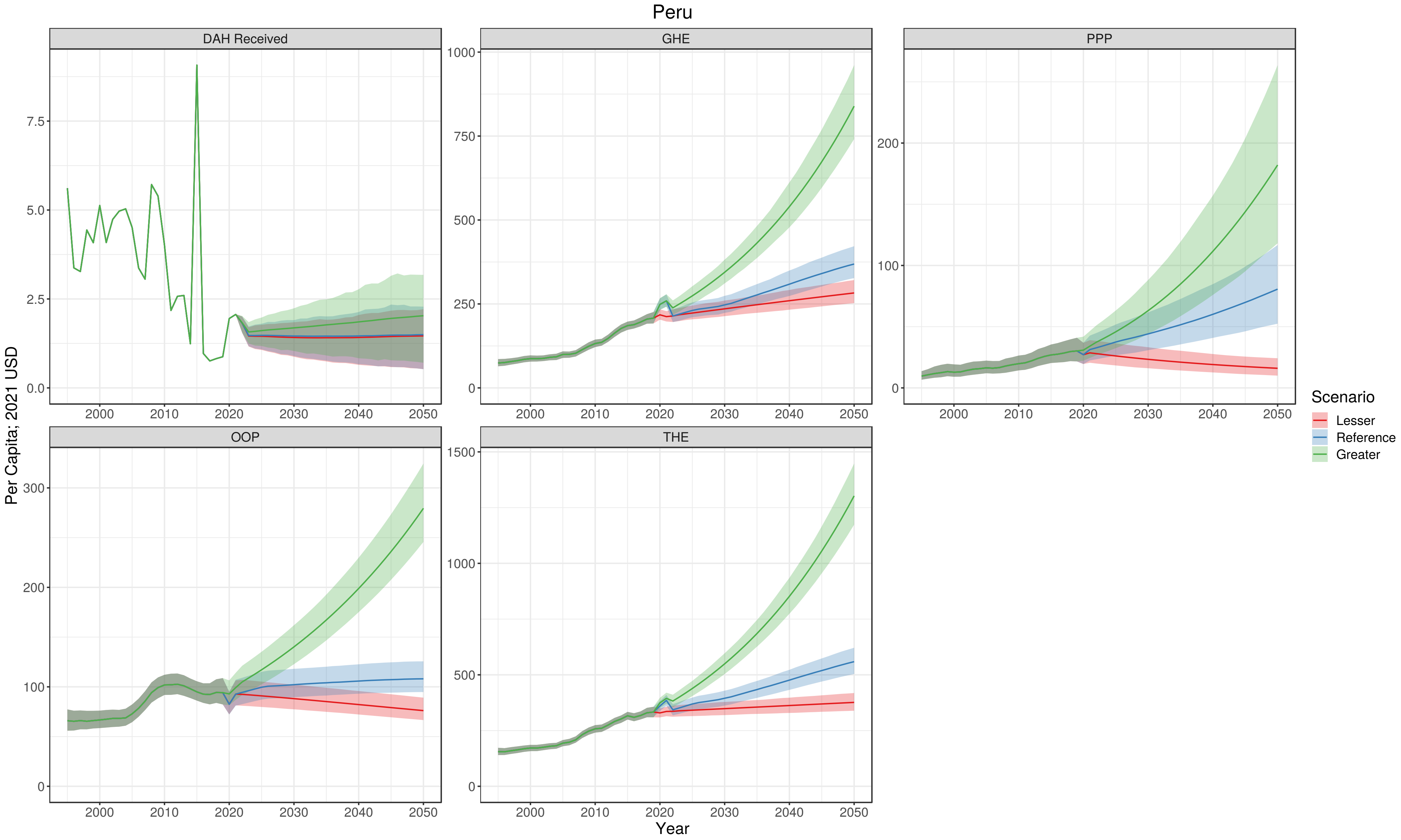

Peru
DAH Received
GHE
PPP
OOP
THE
Per Capita; 2021 USD
Year
Scenario
Lesser
Reference
Greater
0.0
2.5
5.0
7.5
0
250
500
750
1000
0
100
200
300
1500
2000
2010
2020
2030
2040
2050

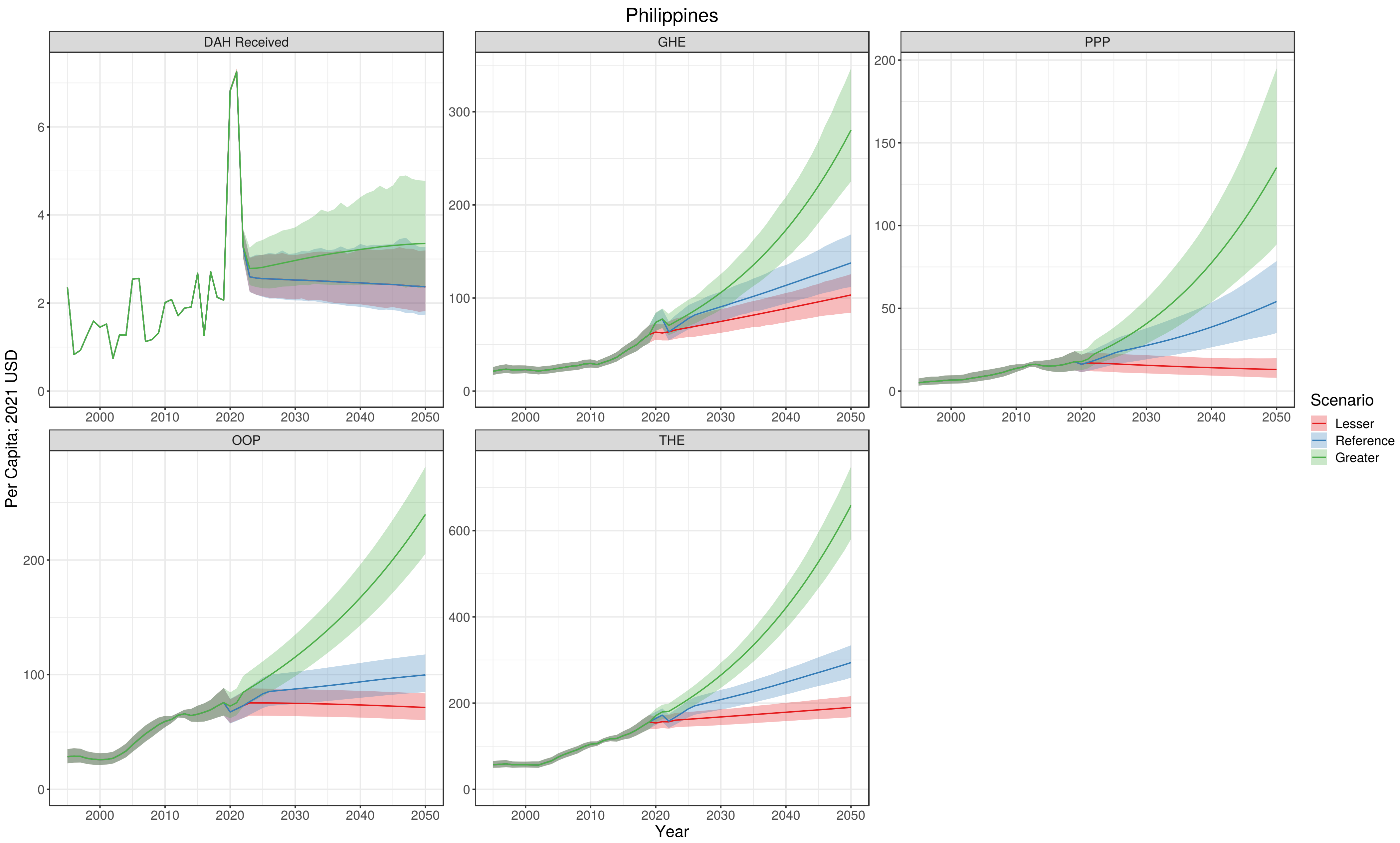
Philippines
DAH Received
GHE
PPP
OOP
THE
Per Capita; 2021 USD
Year
Scenario
Lesser
Reference
Greater

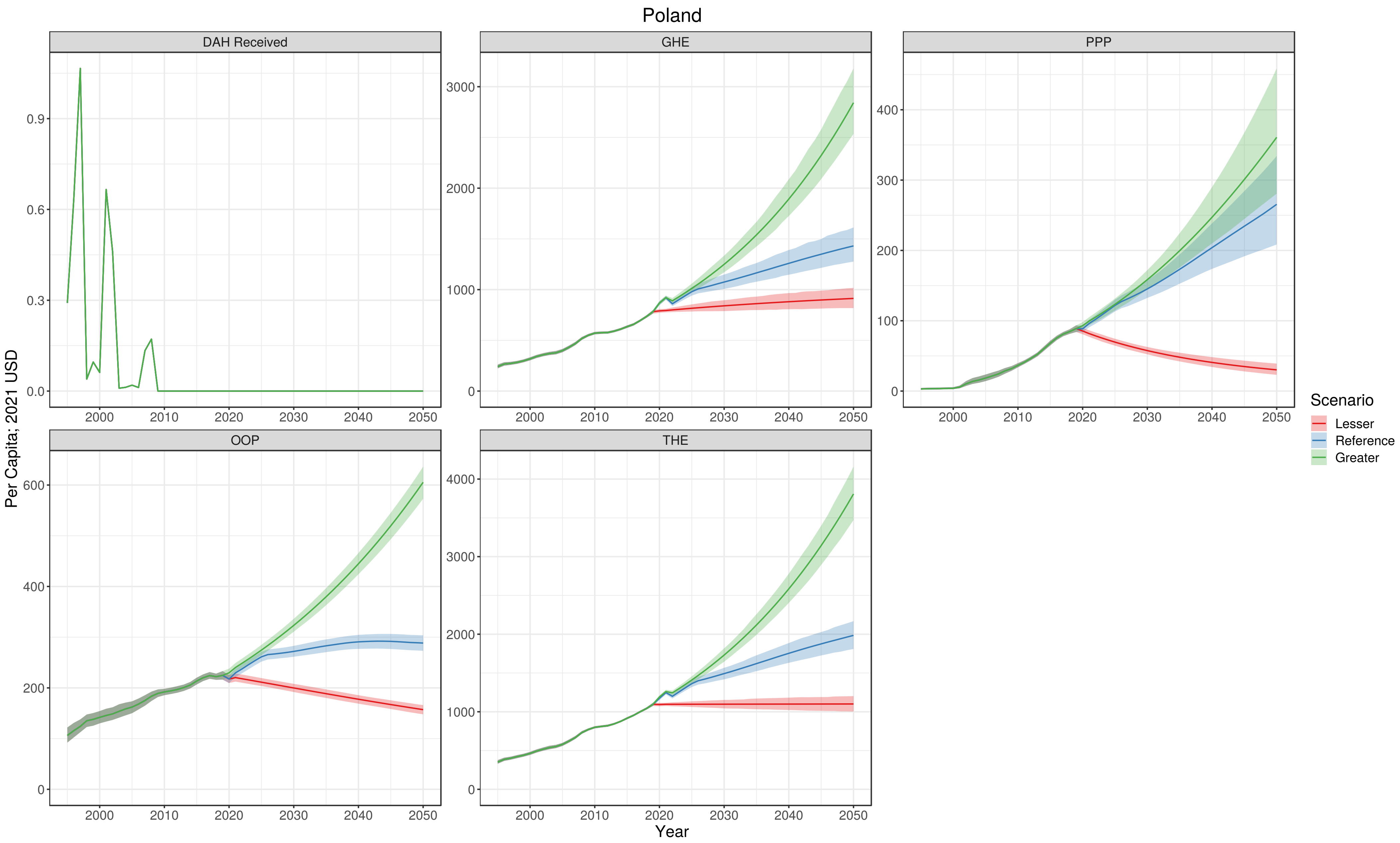

Poland
DAH Received
GHE
PPP
OOP
THE
Per Capita; 2021 USD
Year
Scenario
Lesser
Reference
Greater

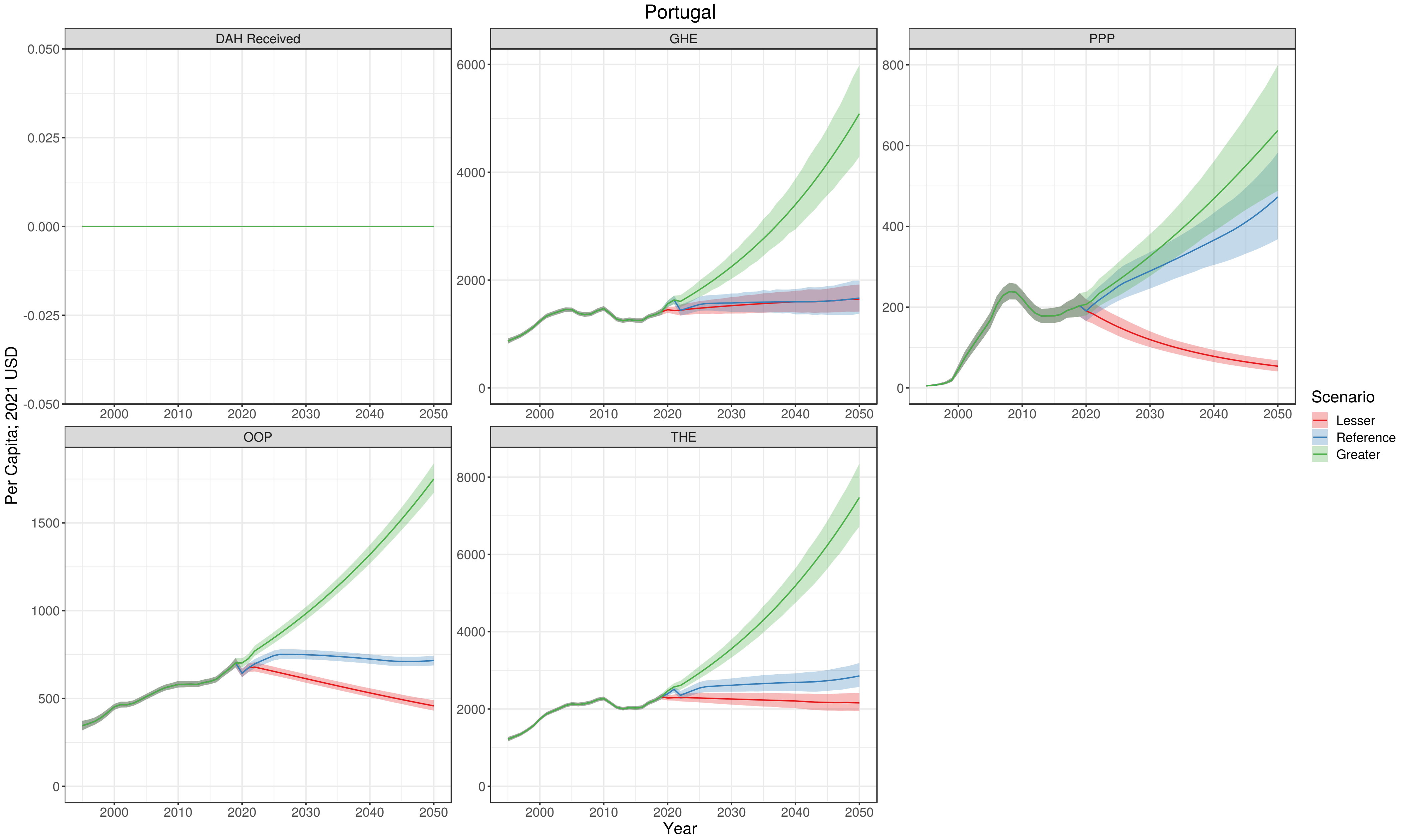
Portugal
DAH Received
GHE
PPP
OOP
THE
Per Capita; 2021 USD
Year
Scenario
Lesser
Reference
Greater
0.050
0.025
0.000
-0.025
-0.050
6000
4000
2000
0
800
600
400
200
1500
1000
500
8000
2000
2010
2020
2030
2040
2050

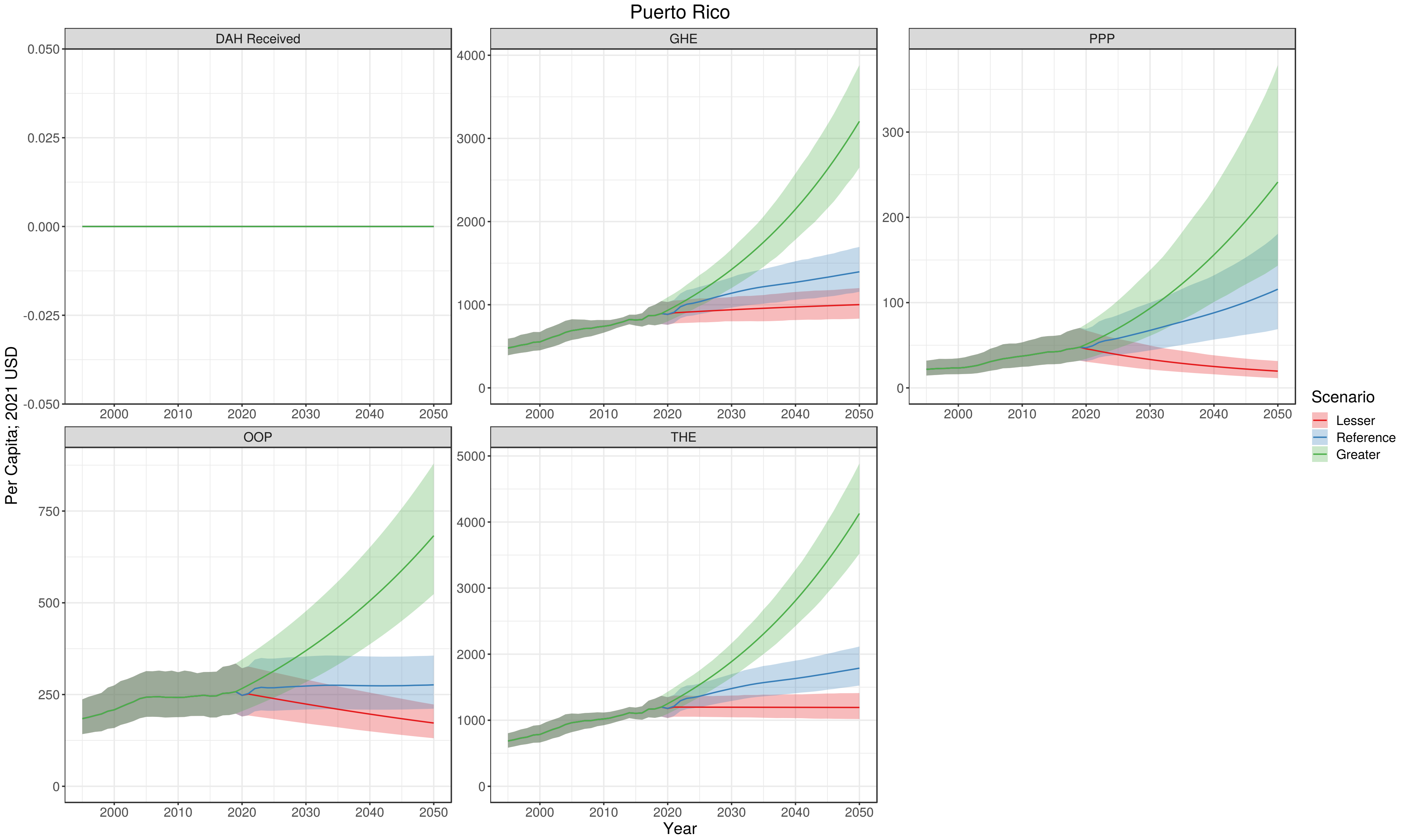

Puerto Rico
DAH Received
GHE
PPP
OOP
THE
0.050
0.025
0.000
-0.025
-0.050
4000
3000
2000
1000
0
300
200
100
750
500
250
5000
2000
2010
2020
2030
2040
2050
Year
Per Capita; 2021 USD
Scenario
Lesser
Reference
Greater

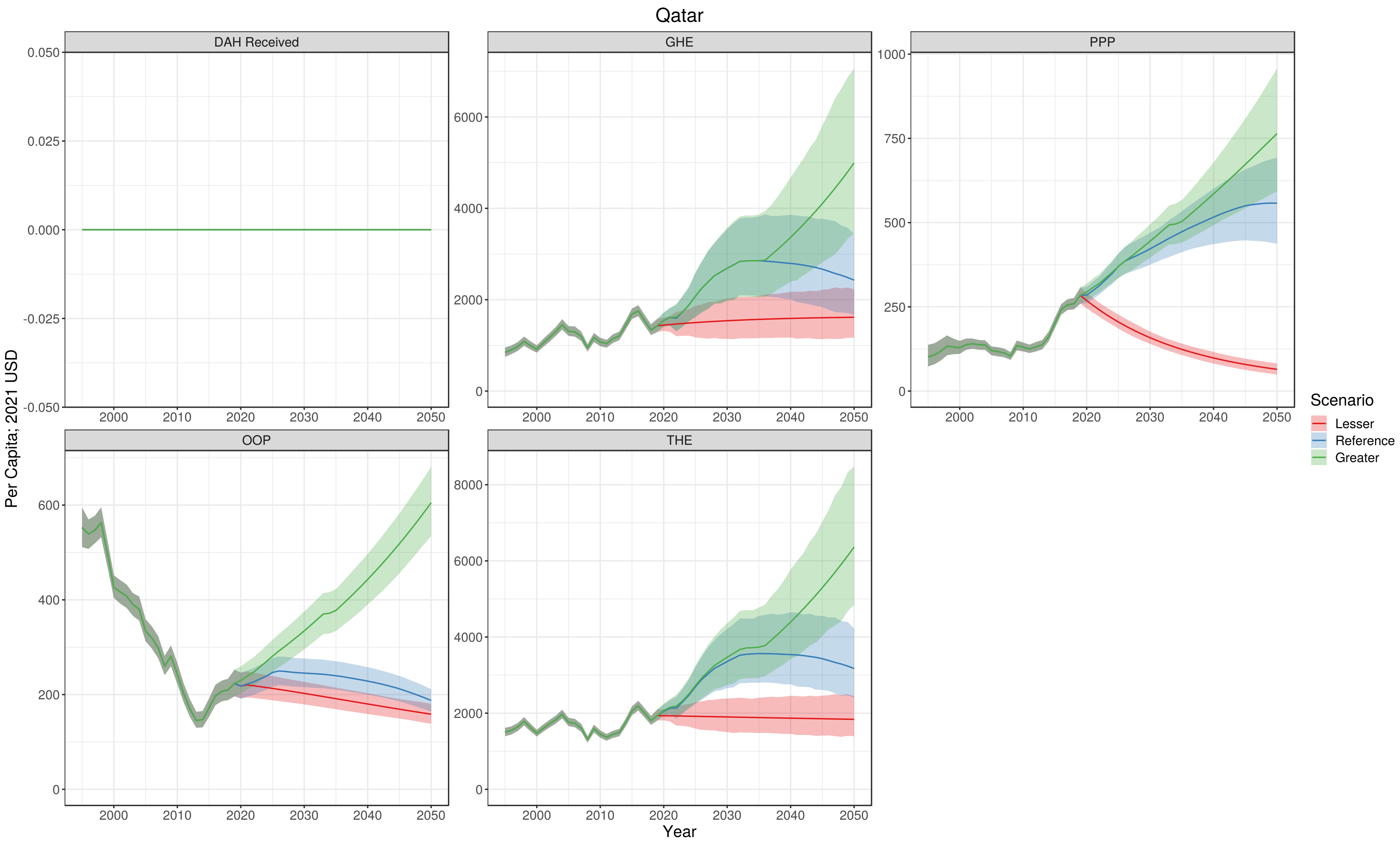
Qatar
DAH Received
GHE
PPP
OOP
THE
Per Capita; 2021 USD
Year
Scenario
Lesser
Reference
Greater
0.050
0.025
0.000
-0.025
-0.050
6000
4000
2000
0
1000
750
500
250
600
400
200
8000
2000
2010
2020
2030
2040
2050

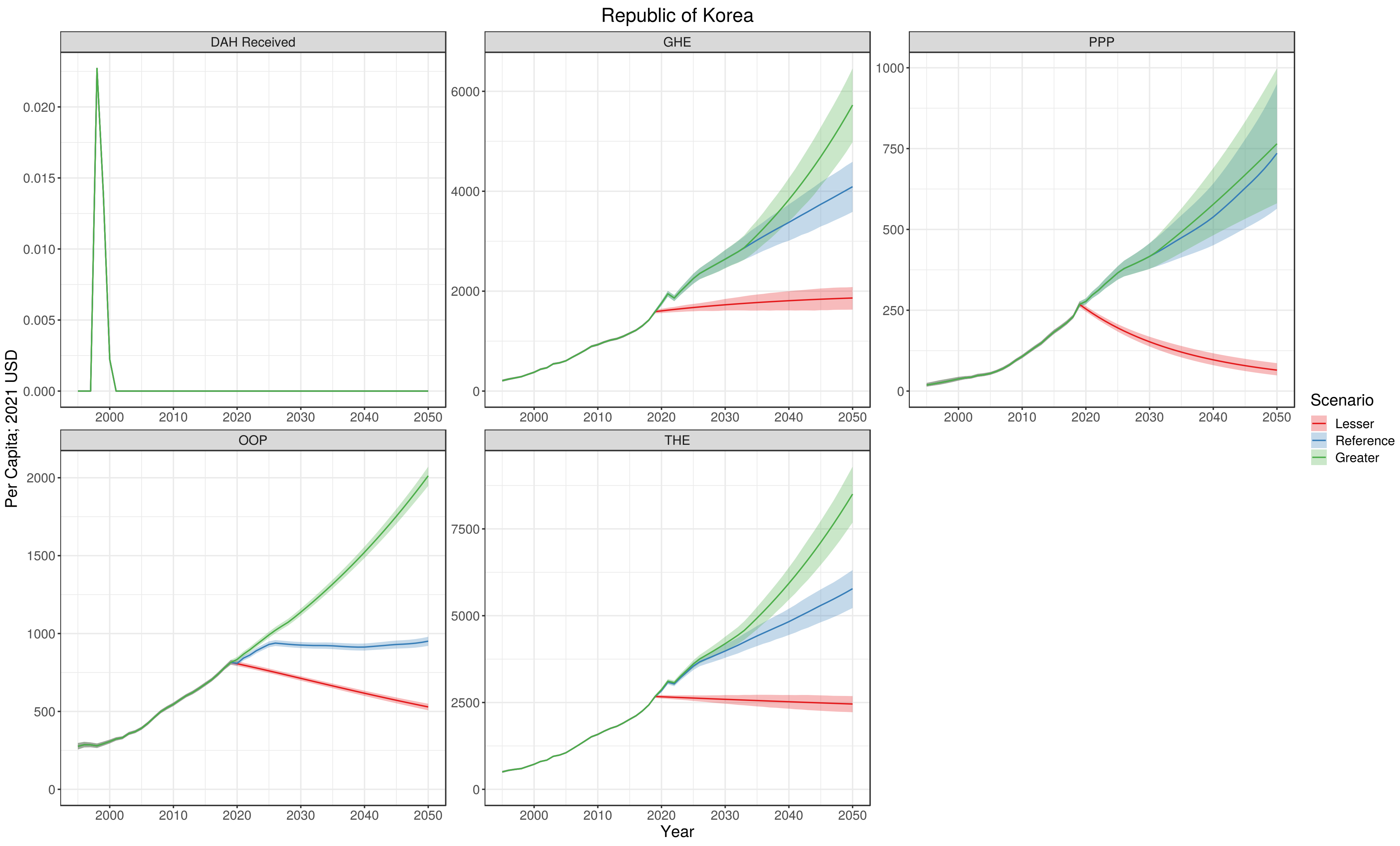

Republic of Korea
DAH Received
GHE
PPP
OOP
THE
0.000
0.005
0.010
0.015
0.020
0
2000
4000
6000
0
250
500
750
1000
0
500
1000
1500
2000
0
2500
5000
7500
2000
2010
2020
2030
2040
2050
Per Capita; 2021 USD
Year
Scenario
Lesser
Reference
Greater

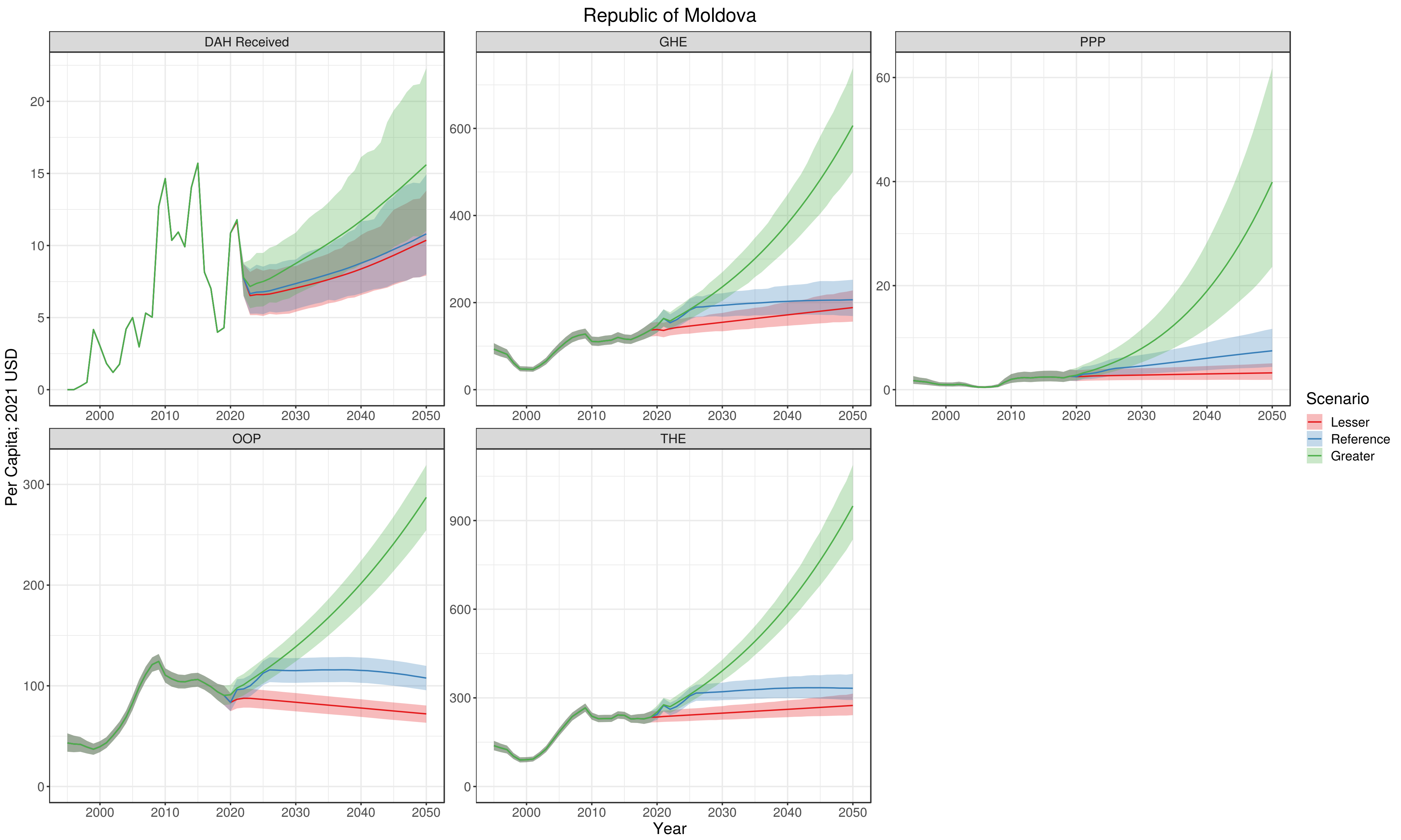

Republic of Moldova
DAH Received
GHE
PPP
OOP
THE
Per Capita; 2021 USD
Year
Scenario
Lesser
Reference
Greater
0
5
10
15
20
200
400
600
40
60
100
300
900
2000
2010
2020
2030
2040
2050

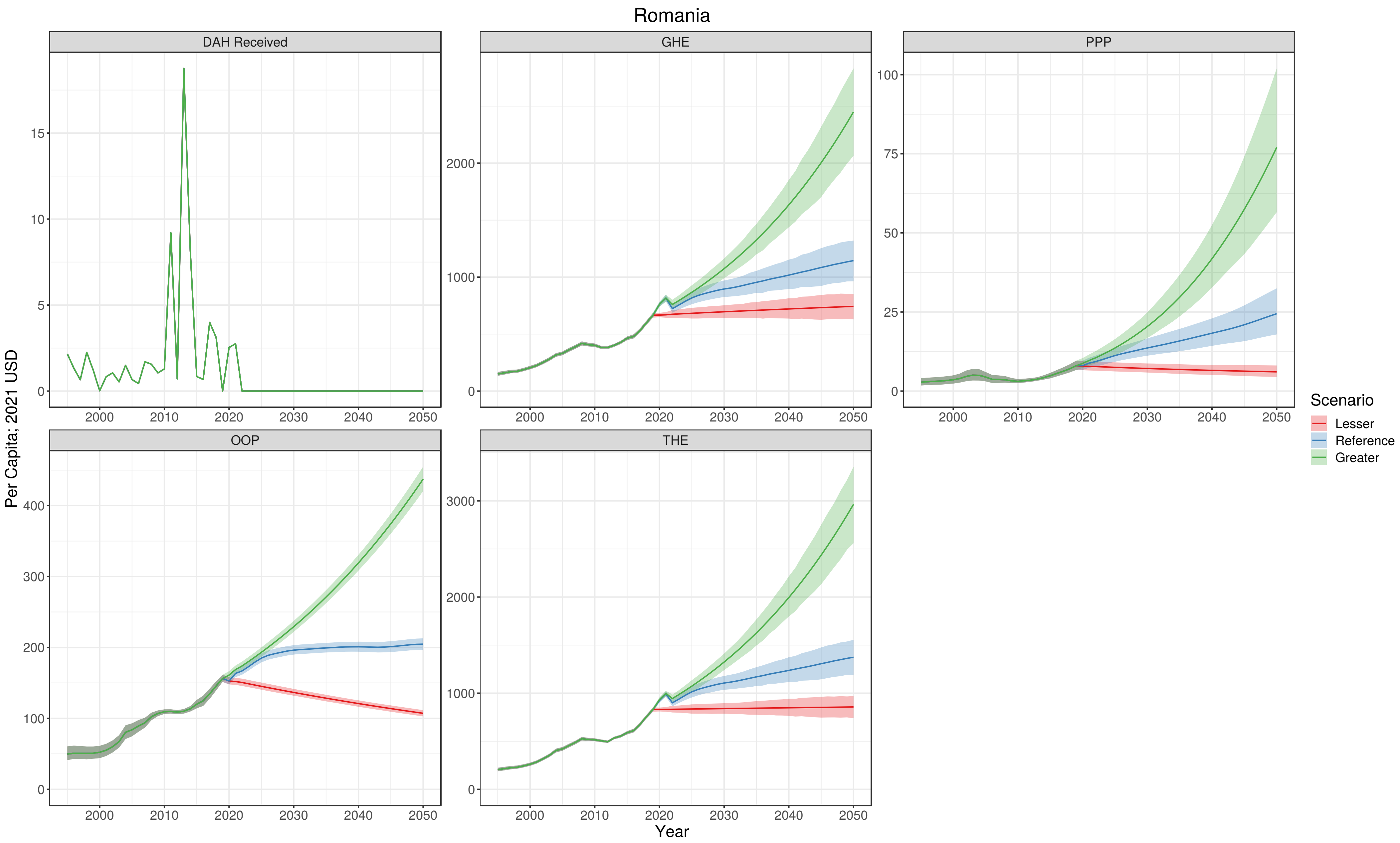

Romania
DAH Received
GHE
PPP
OOP
THE
Per Capita; 2021 USD
Year
Scenario
Lesser
Reference
Greater

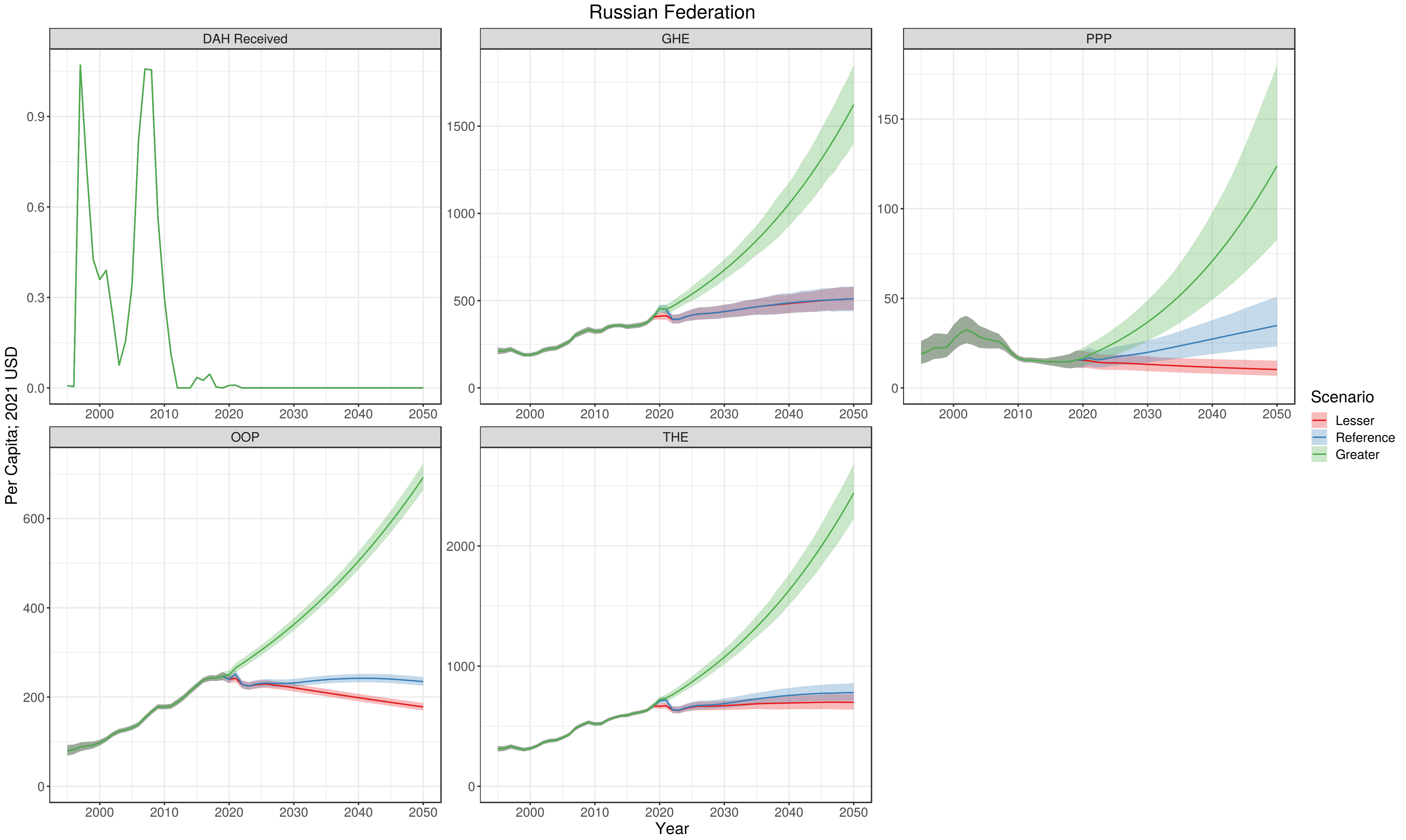

Russian Federation
DAH Received
GHE
PPP
OOP
THE
Per Capita; 2021 USD
Year
Scenario
Lesser
Reference
Greater

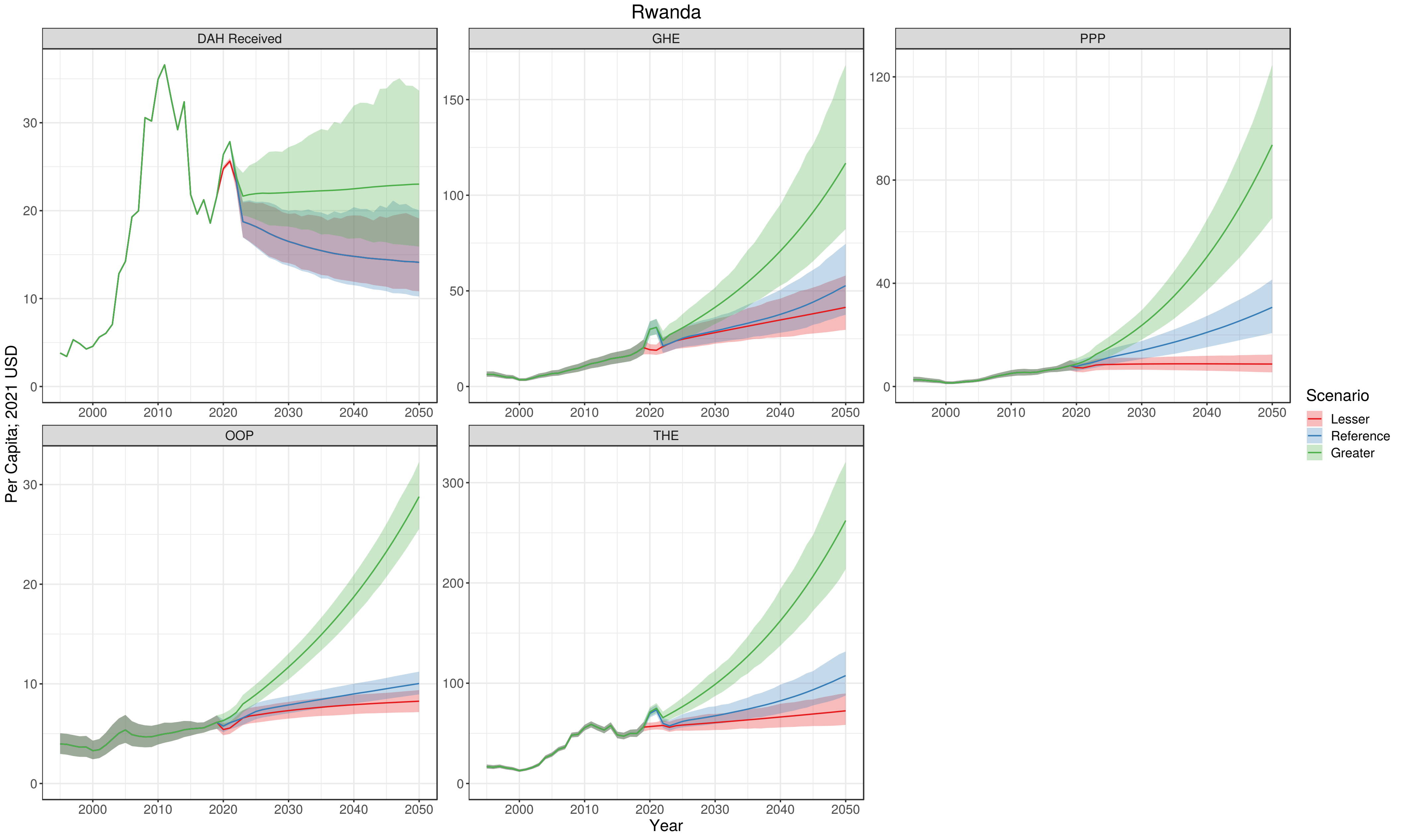
Rwanda
DAH Received
GHE
PPP
OOP
THE
Per Capita; 2021 USD
Year
Scenario
Lesser
Reference
Greater
0
10
20
30
0
50
100
150
0
40
80
120
0
100
200
300
2000
2010
2020
2030
2040
2050

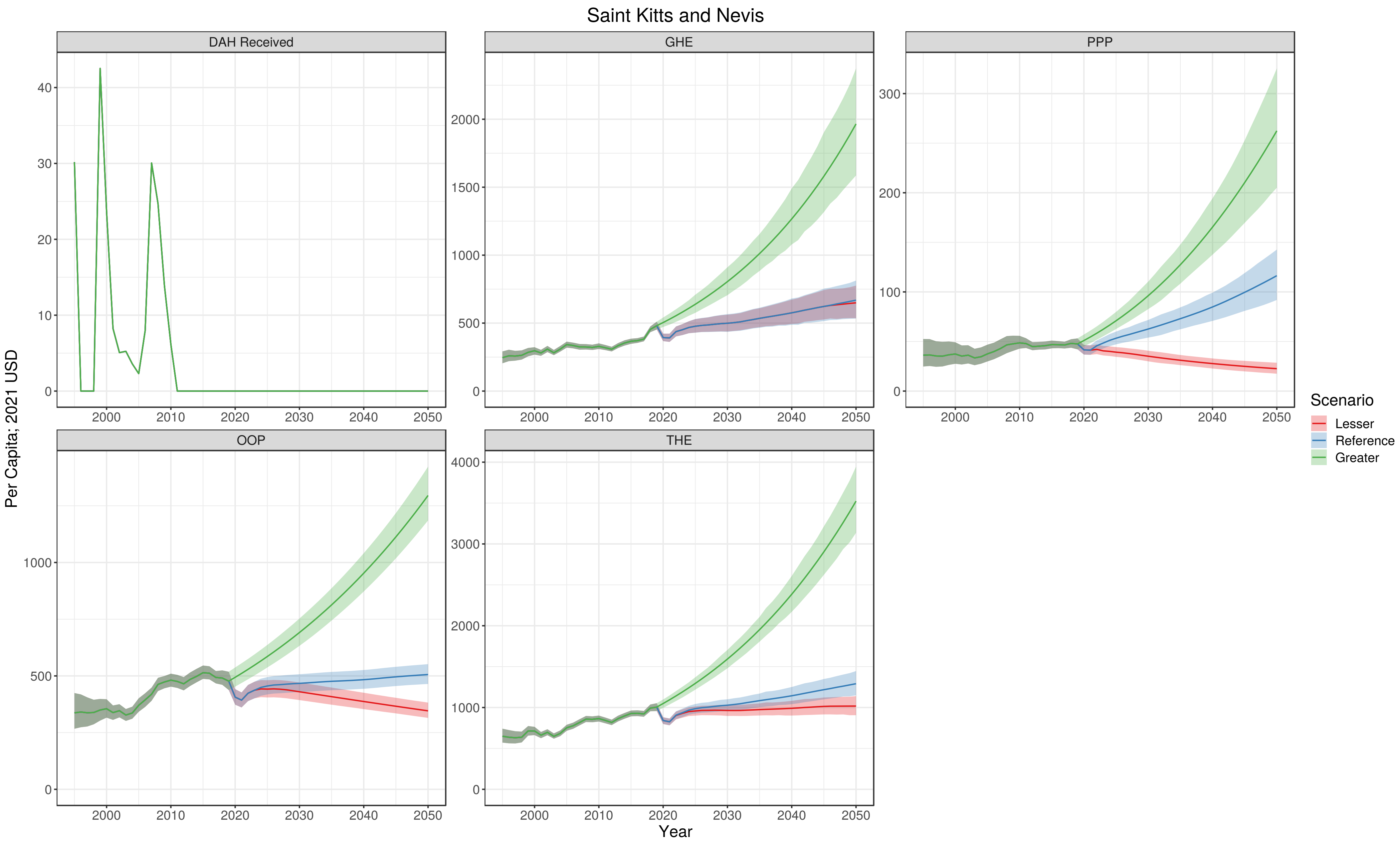

Saint Kitts and Nevis
DAH Received
GHE
PPP
OOP
THE
Per Capita; 2021 USD
Year
Scenario
Lesser
Reference
Greater

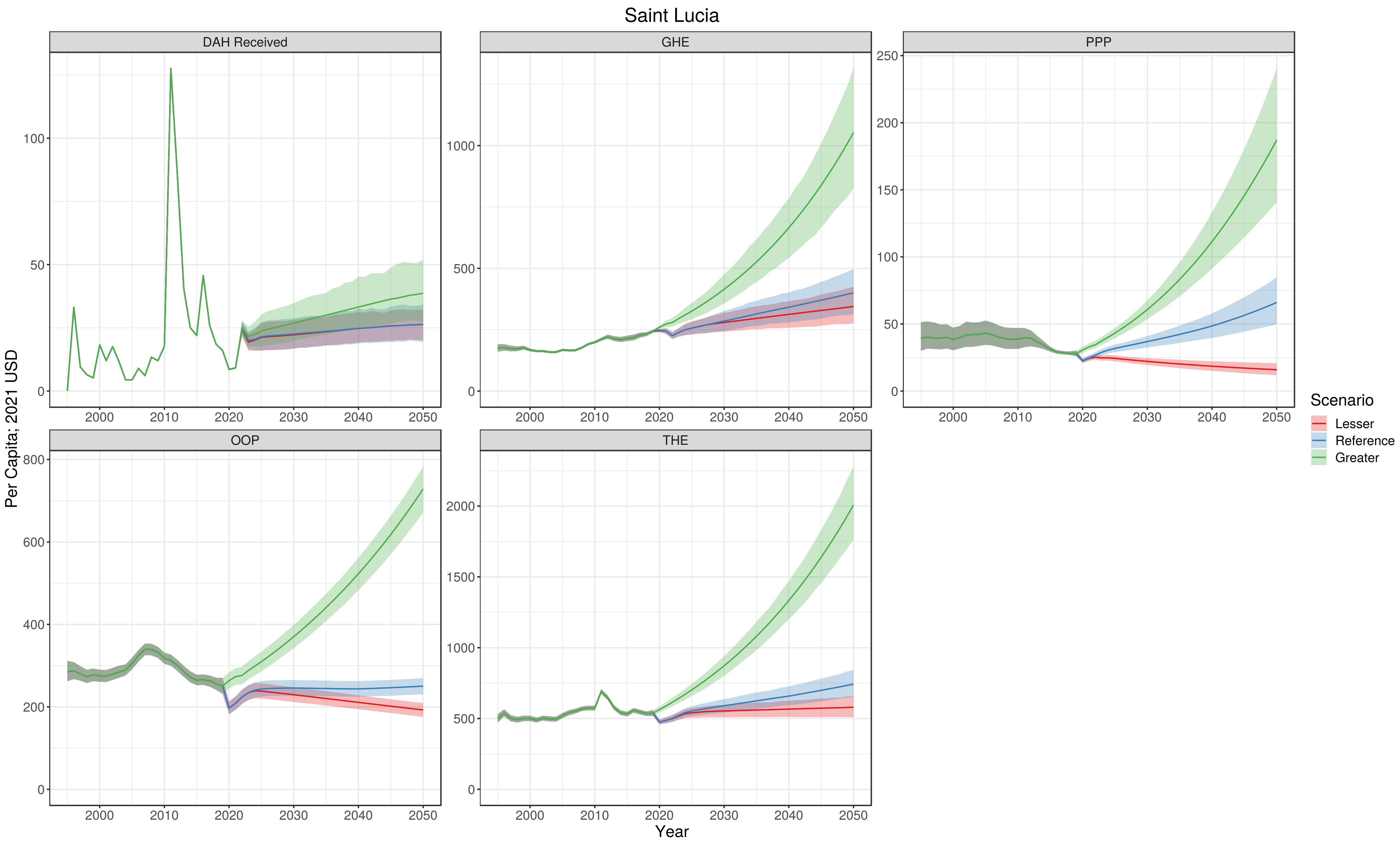

Saint Lucia
DAH Received
GHE
PPP
OOP
THE
Per Capita; 2021 USD
Year
Scenario
Lesser
Reference
Greater

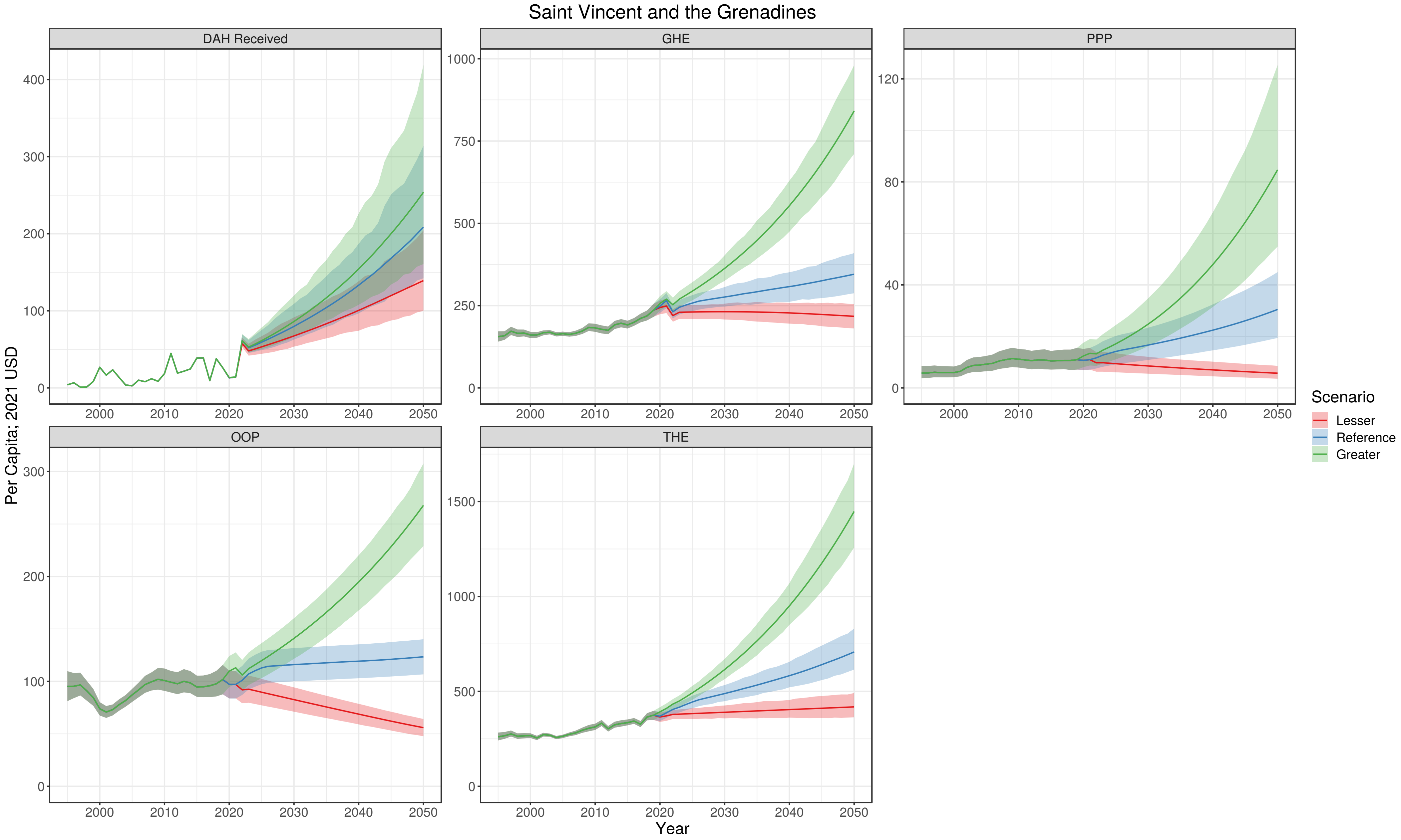

Saint Vincent and the Grenadines
DAH Received
GHE
PPP
OOP
THE
Per Capita; 2021 USD
Year
Scenario
Lesser
Reference
Greater

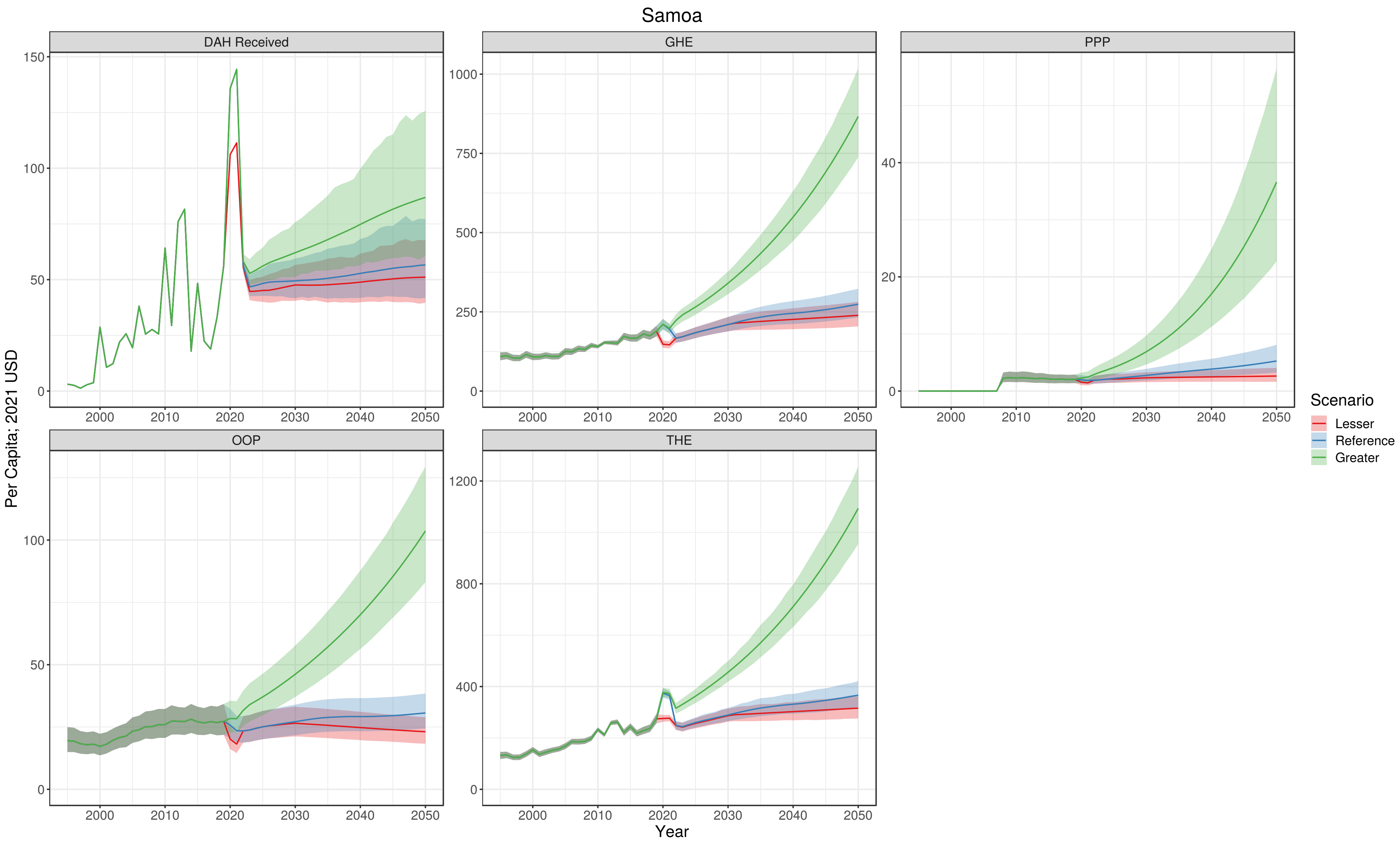

Samoa
DAH Received
GHE
PPP
OOP
THE
Per Capita; 2021 USD
Year
Scenario
Lesser
Reference
Greater

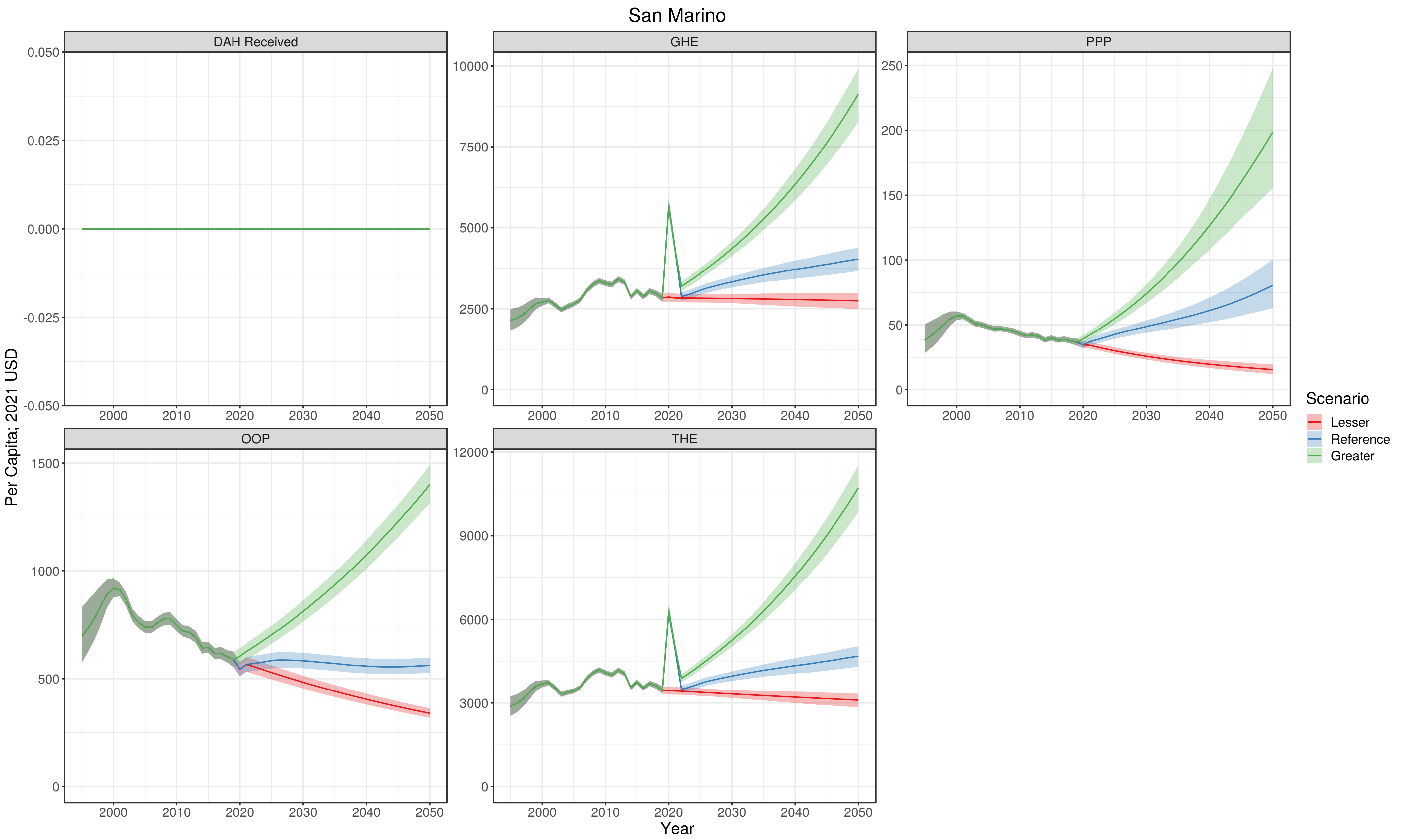
San Marino
DAH Received
GHE
PPP
OOP
THE
Per Capita; 2021 USD
Year
Scenario
Lesser
Reference
Greater
0.050
0.025
0.000
-0.025
-0.050
10000
7500
5000
2500
0
250
200
150
100
50
1500
1000
500
12000
9000
6000
3000
2000
2010
2020
2030
2040
2050

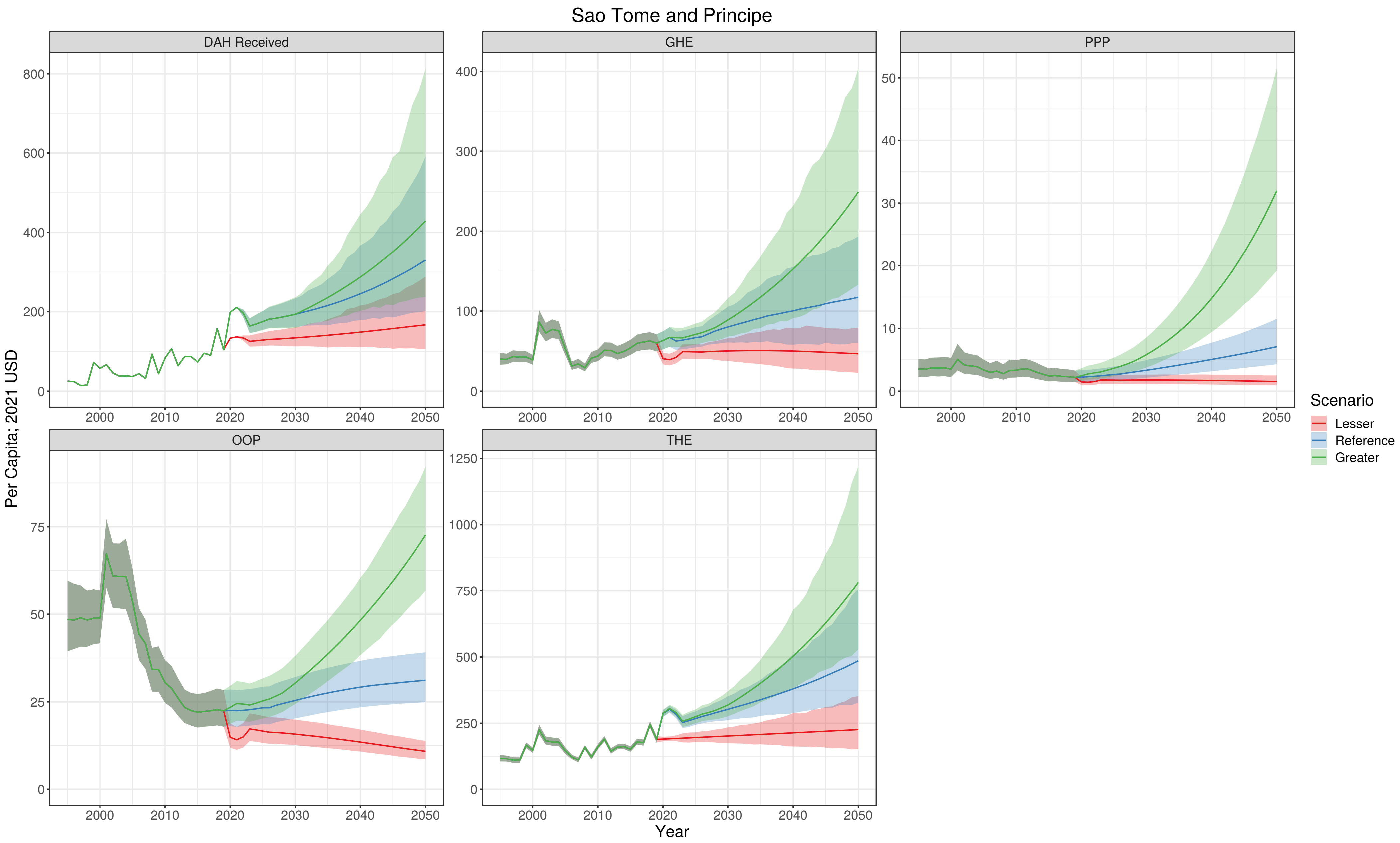

Sao Tome and Principe
DAH Received
GHE
PPP
OOP
THE
Per Capita; 2021 USD
Year
Scenario
Lesser
Reference
Greater

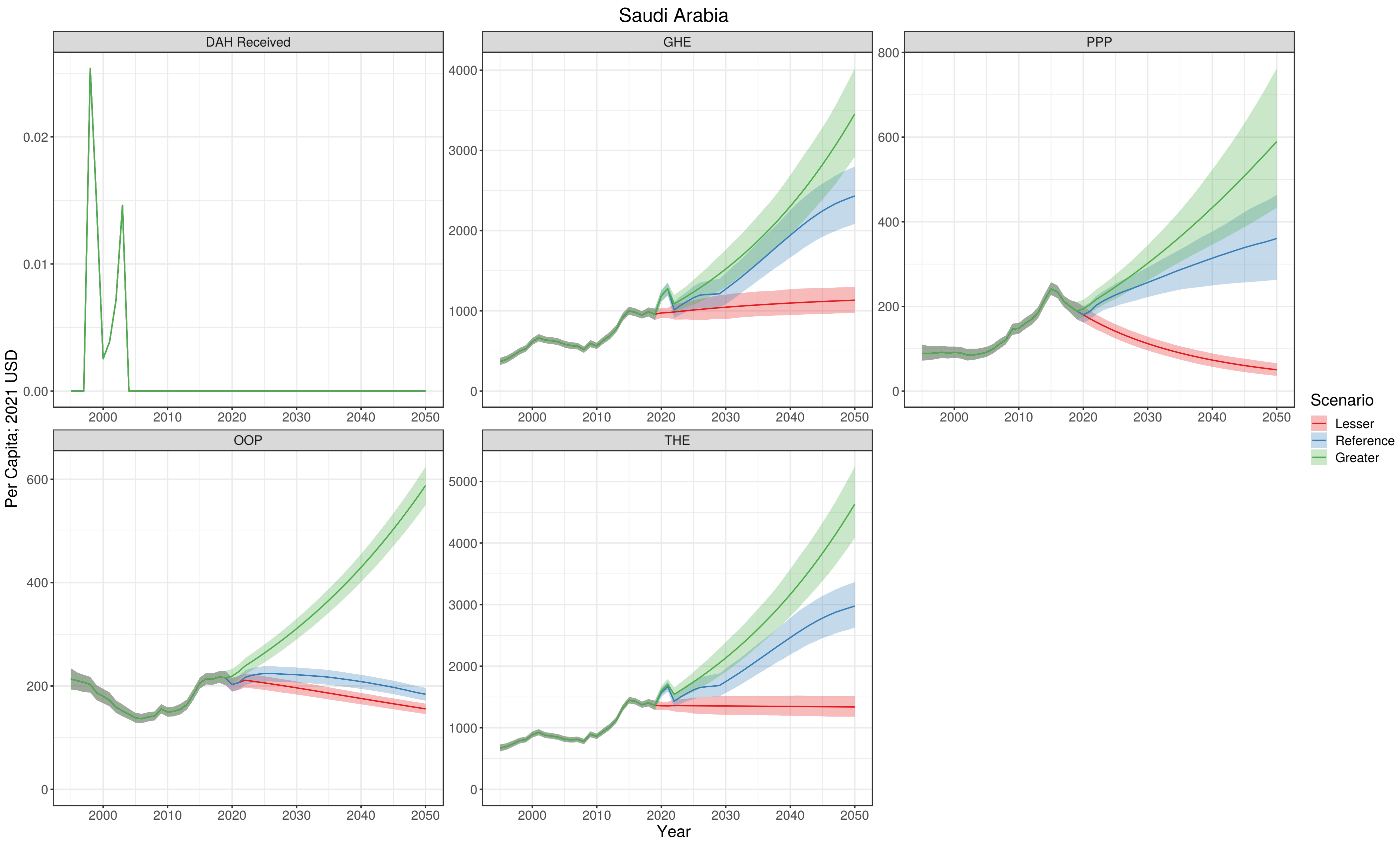

Saudi Arabia
DAH Received
GHE
PPP
OOP
THE
Per Capita; 2021 USD
Year
Scenario
Lesser
Reference
Greater
0.00
0.01
0.02
0
1000
2000
3000
4000
5000
200
400
600
800
2000
2010
2020
2030
2040
2050

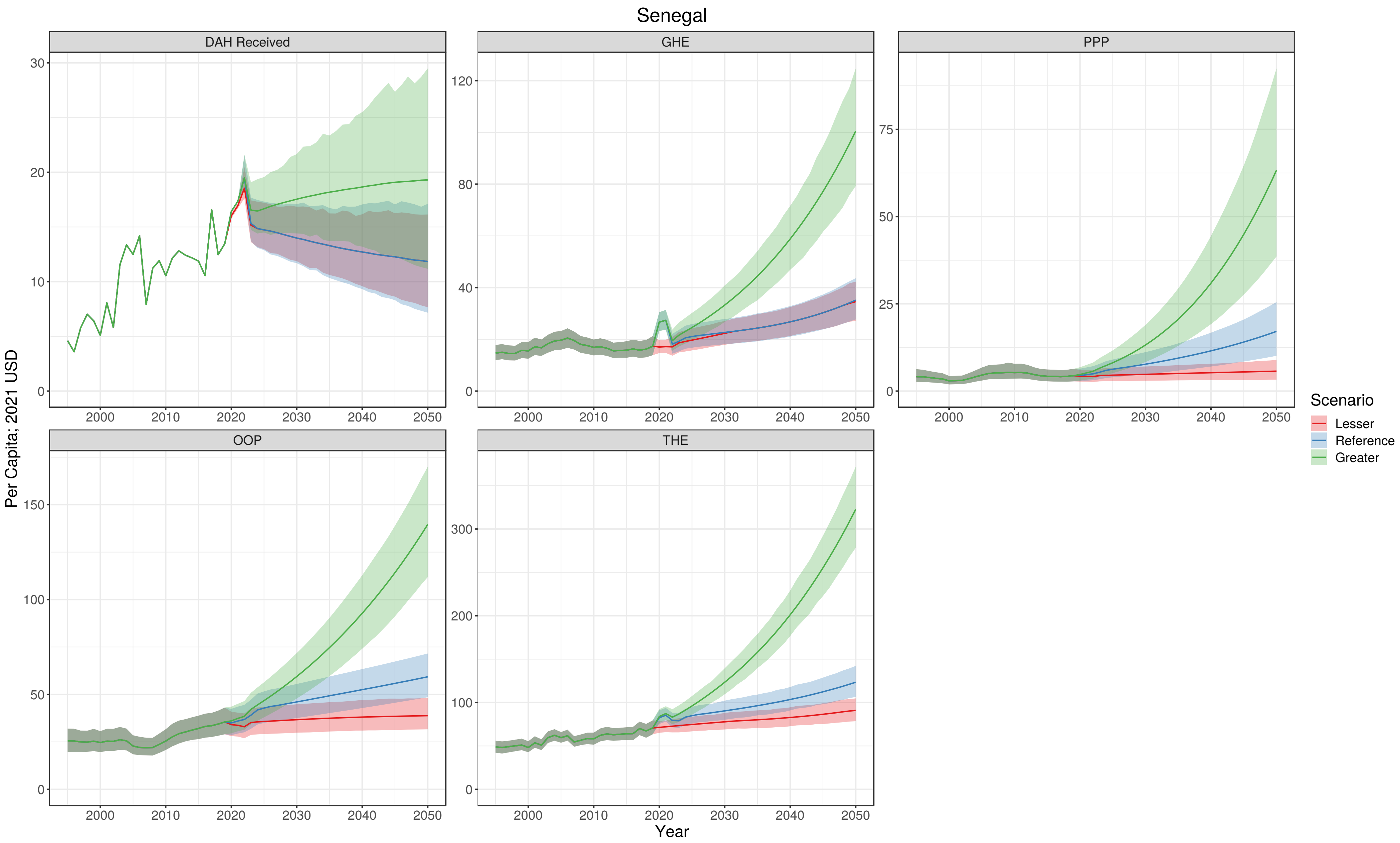
Senegal
DAH Received
GHE
PPP
OOP
THE
Per Capita; 2021 USD
Year
Scenario
Lesser
Reference
Greater

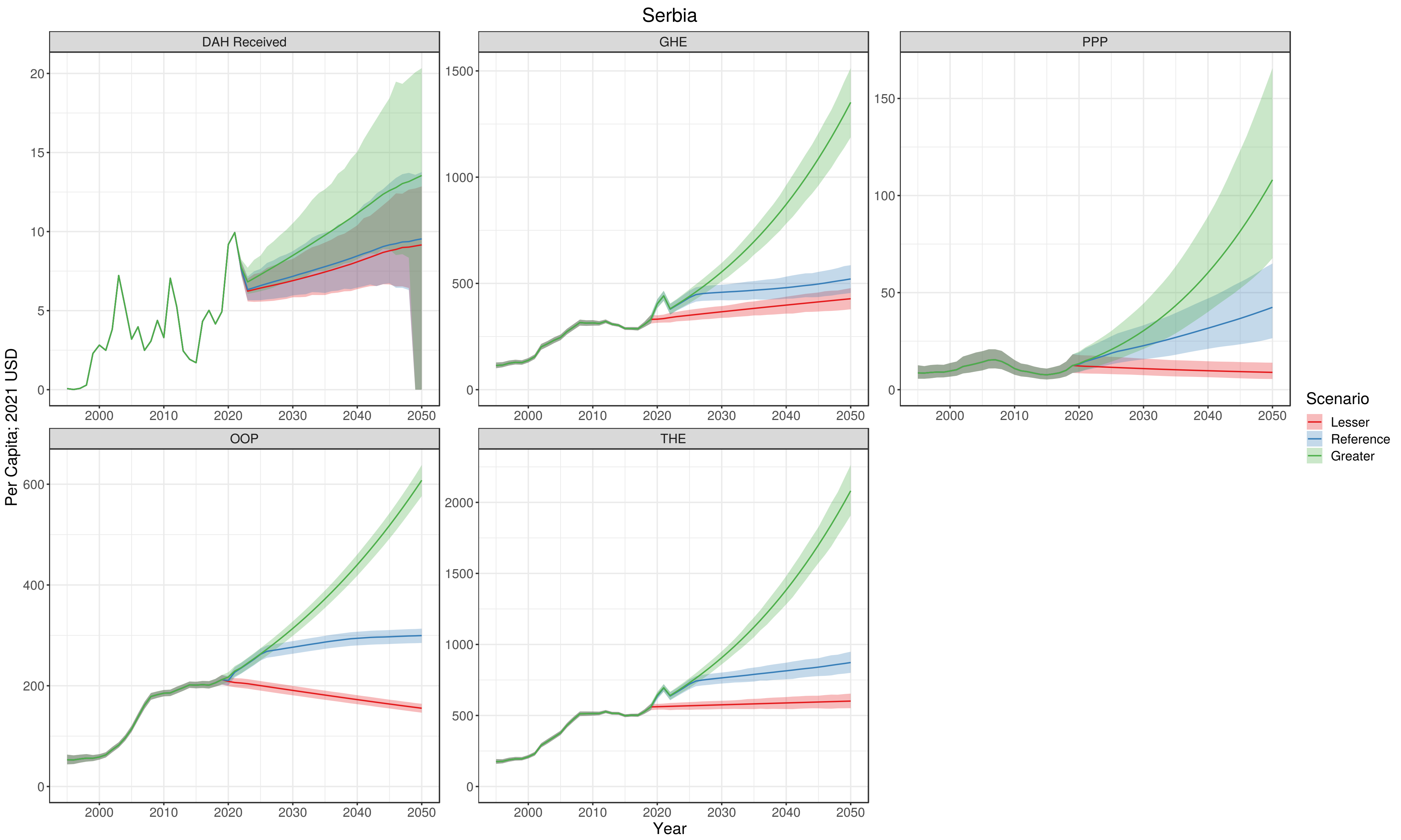
Serbia
DAH Received
GHE
PPP
OOP
THE
Per Capita; 2021 USD
Year
Scenario
Lesser
Reference
Greater
0
5
10
15
20
500
1000
1500
50
100
150
200
400
600
2000
2010
2020
2030
2040
2050

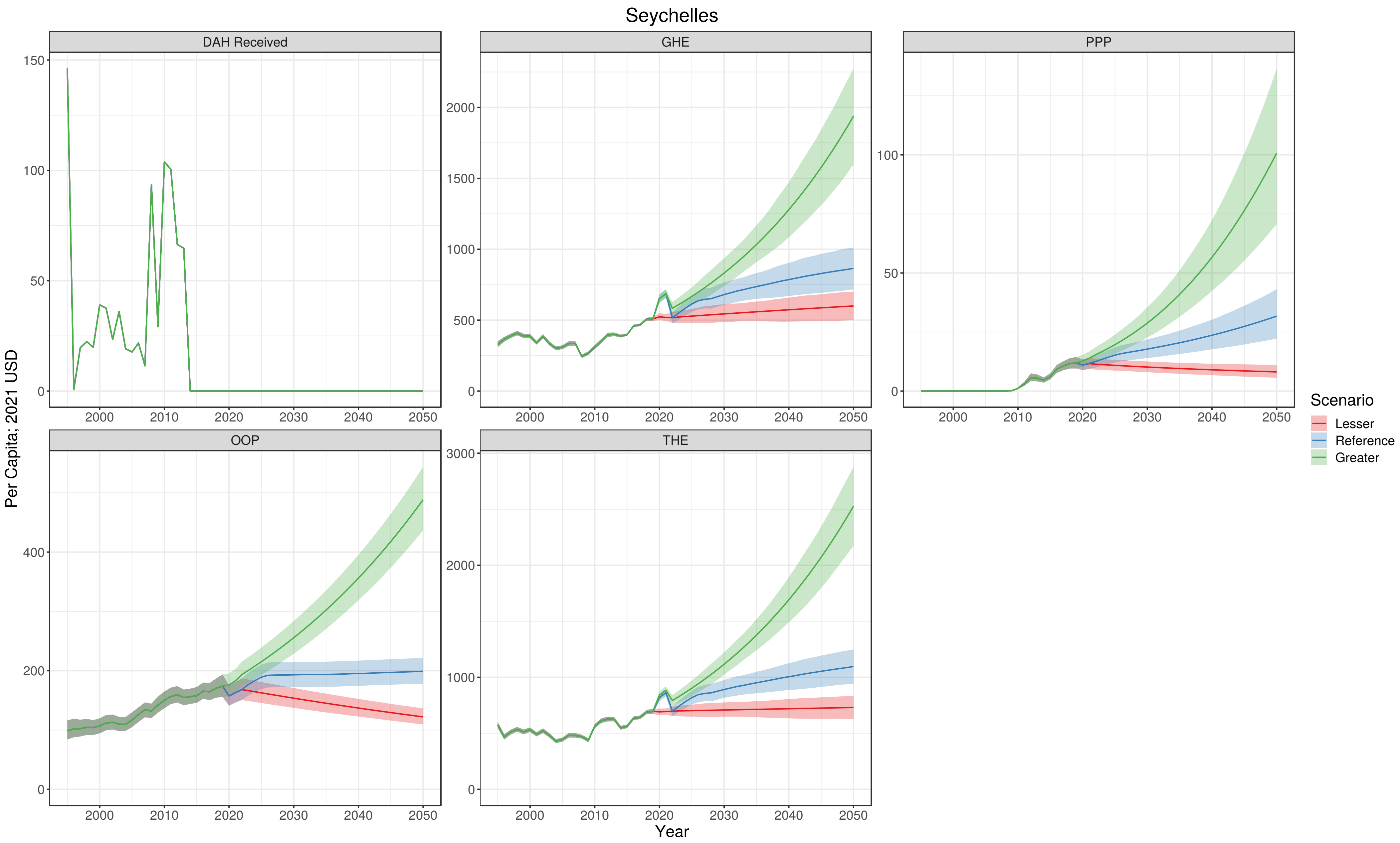

Seychelles
DAH Received
GHE
PPP
OOP
THE
Per Capita; 2021 USD
Year
Scenario
Lesser
Reference
Greater

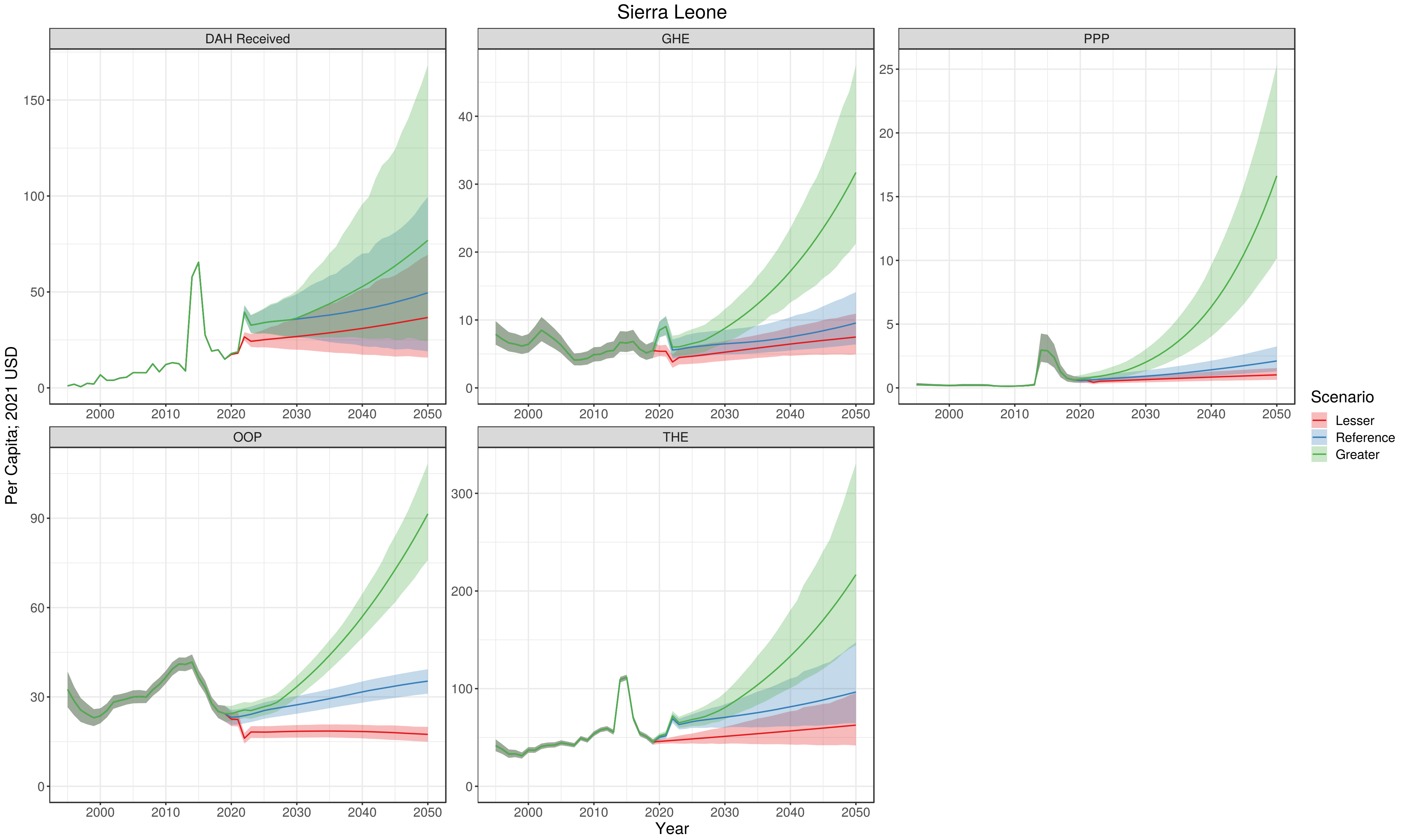

Sierra Leone
DAH Received
GHE
PPP
OOP
THE
Per Capita; 2021 USD
Year
Scenario
Lesser
Reference
Greater

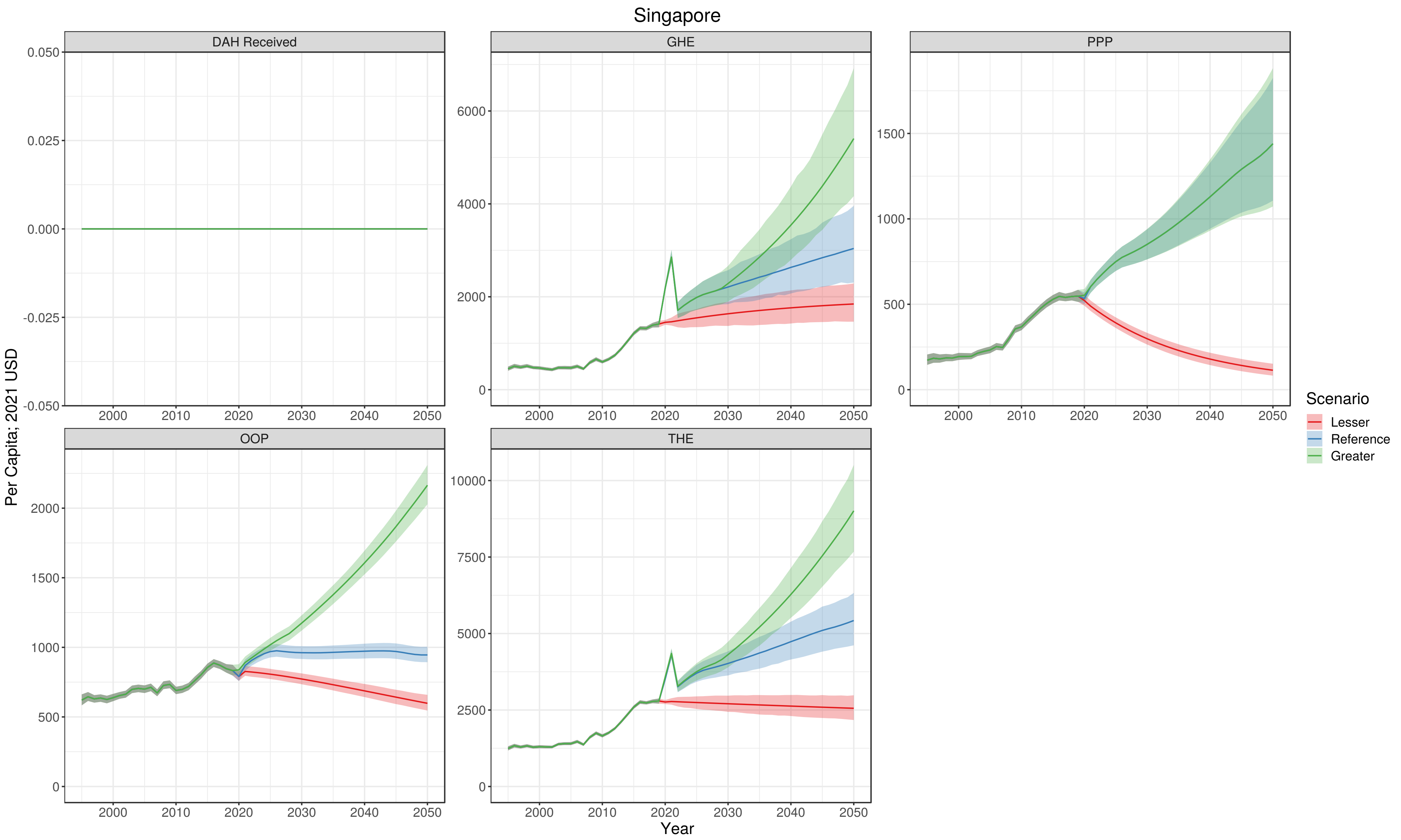

Singapore
DAH Received
GHE
PPP
OOP
THE
Per Capita; 2021 USD
Year
Scenario
Lesser
Reference
Greater
0.050
0.025
0.000
-0.025
-0.050
6000
4000
2000
0
1500
1000
500
2000
1500
1000
500
0
10000
7500
5000
2500
2000
2010
2020
2030
2040
2050

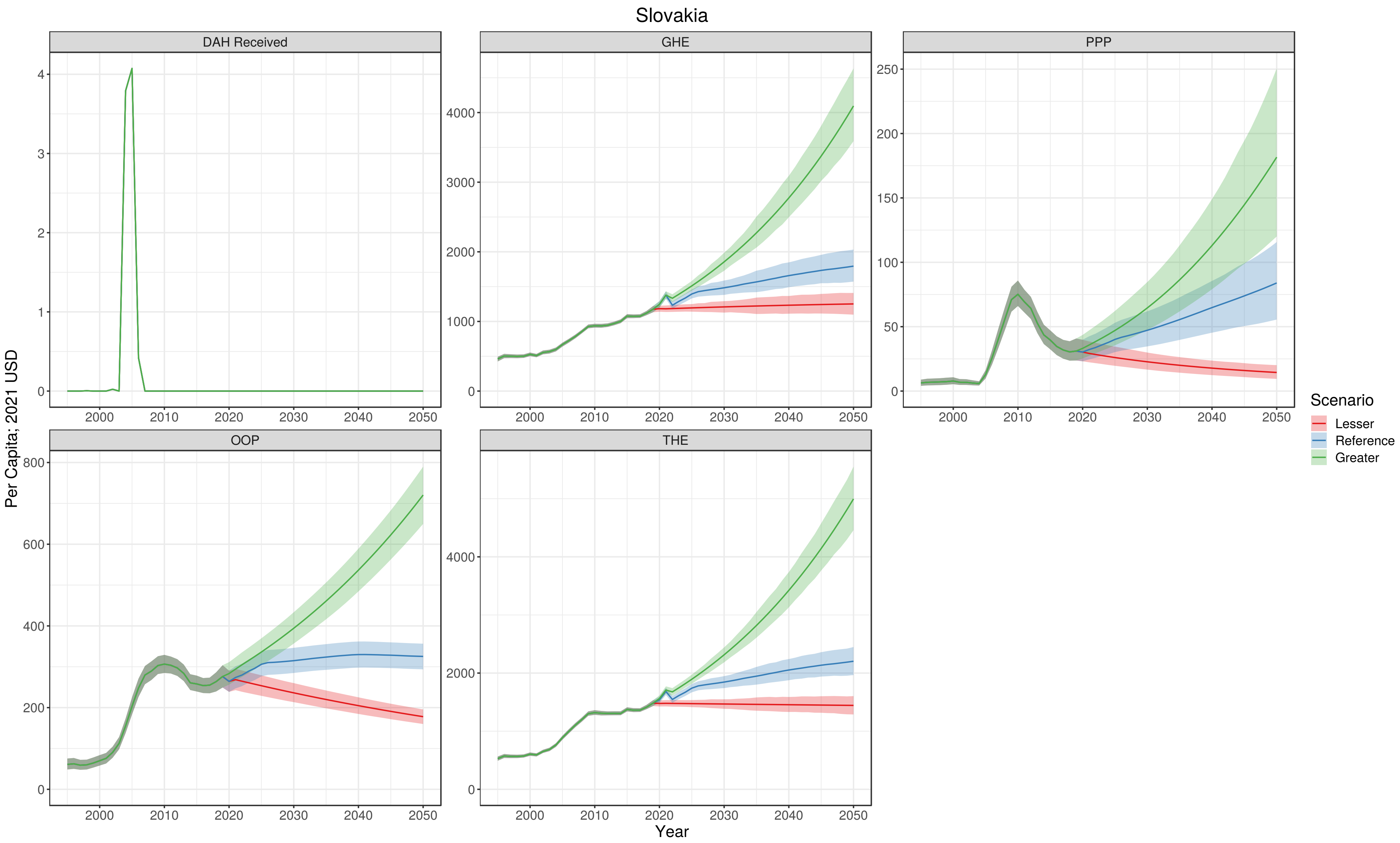

Slovakia
DAH Received
GHE
PPP
OOP
THE
Per Capita; 2021 USD
Year
Scenario
Lesser
Reference
Greater

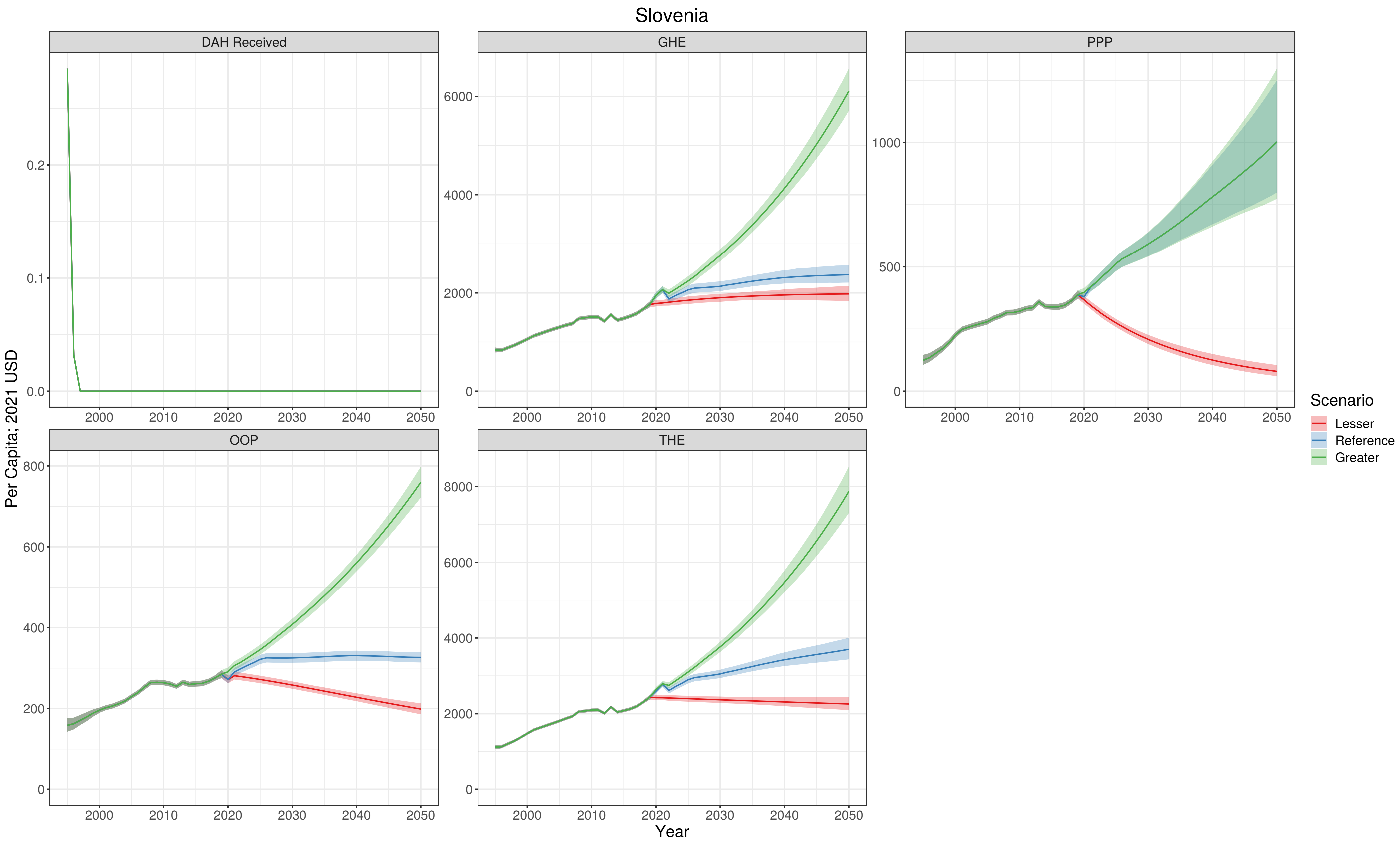

Slovenia
DAH Received
GHE
PPP
OOP
THE
Per Capita; 2021 USD
Year
Scenario
Lesser
Reference
Greater

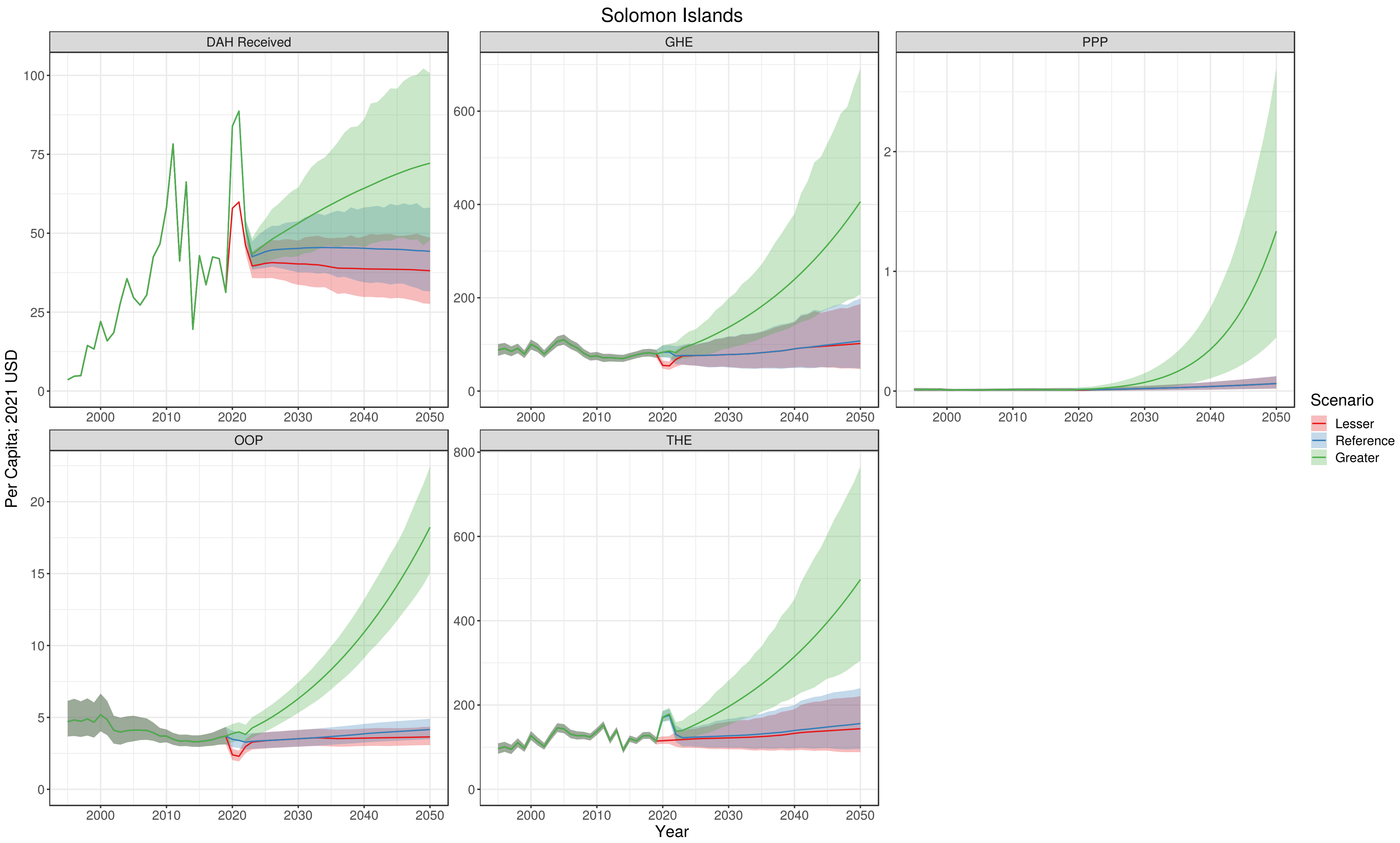

Solomon Islands
DAH Received
GHE
PPP
OOP
THE
Per Capita; 2021 USD
Year
Scenario
Lesser
Reference
Greater

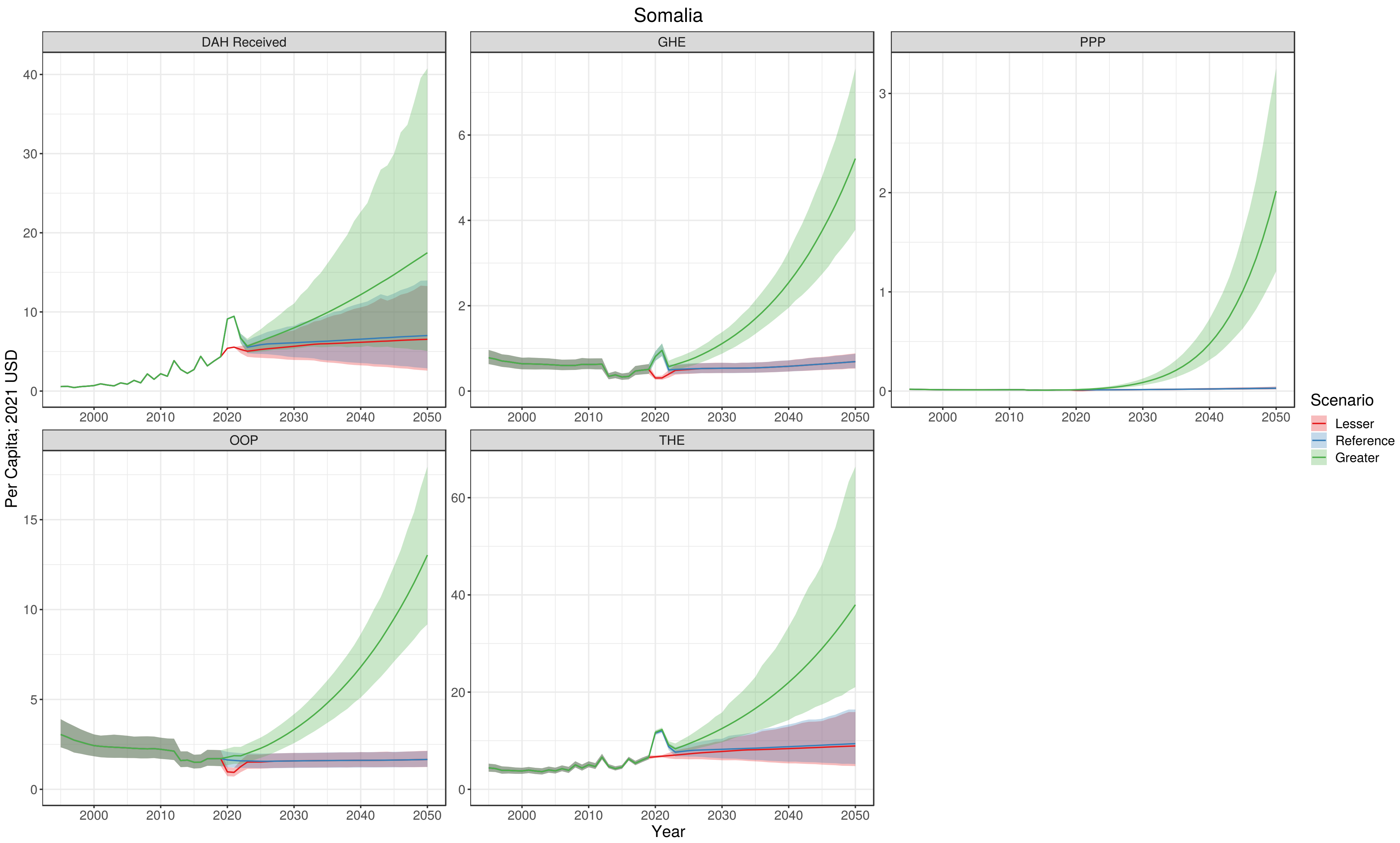

Somalia
DAH Received
GHE
PPP
OOP
THE
Per Capita; 2021 USD
Year
Scenario
Lesser
Reference
Greater

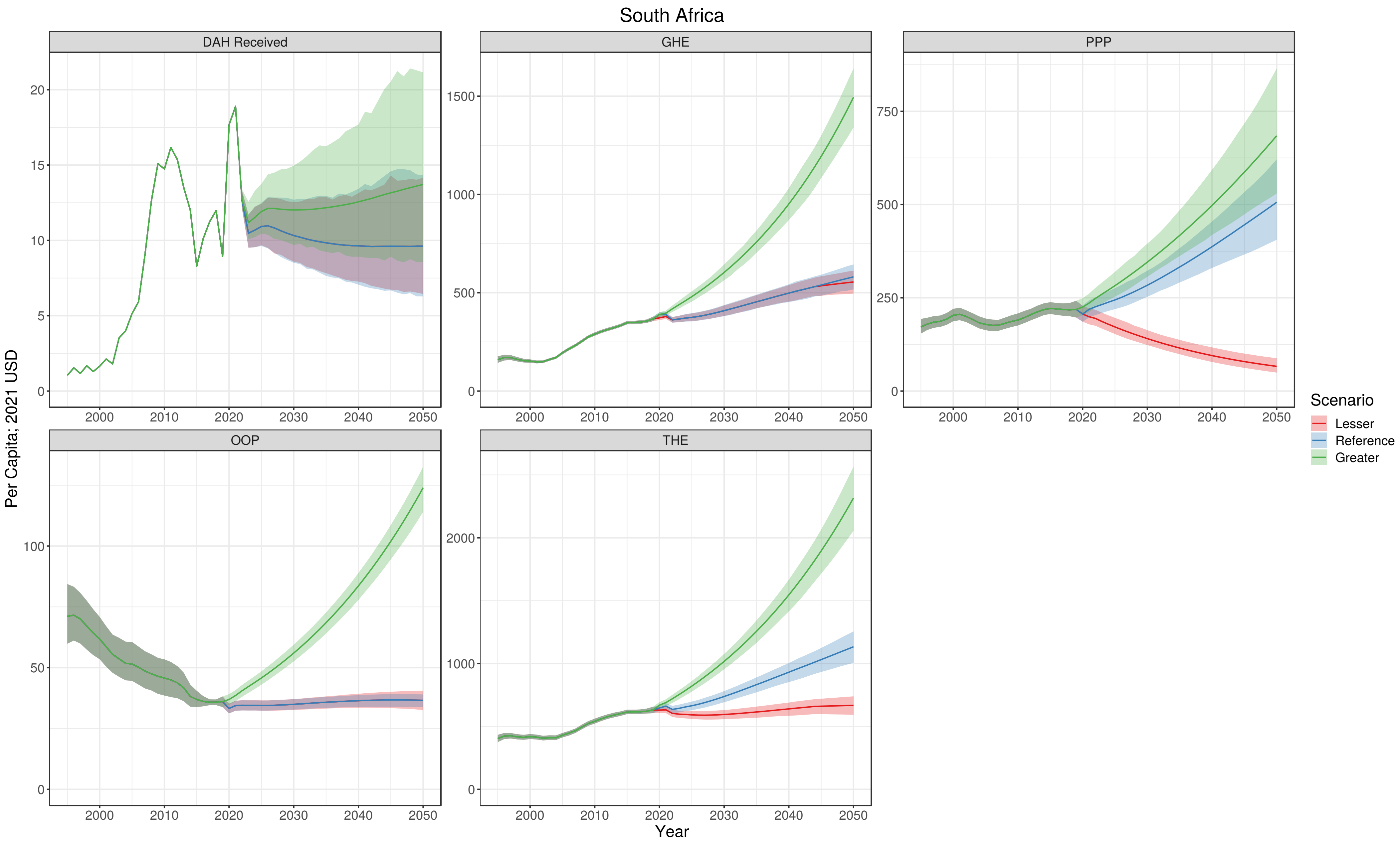

South Africa
DAH Received
GHE
PPP
OOP
THE
Per Capita; 2021 USD
Year
Scenario
Lesser
Reference
Greater

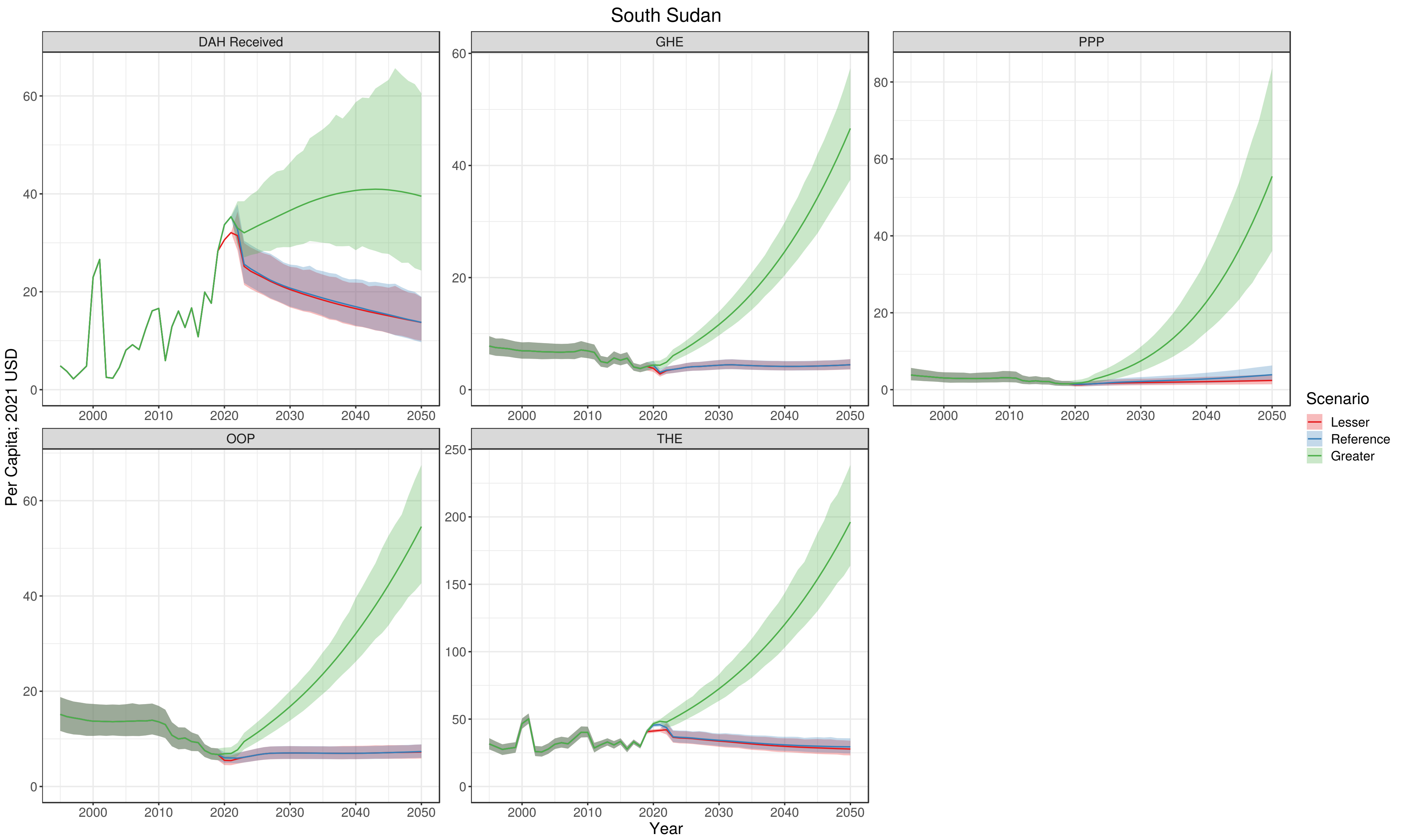

South Sudan
DAH Received
GHE
PPP
OOP
THE
Per Capita; 2021 USD
Year
Scenario
Lesser
Reference
Greater

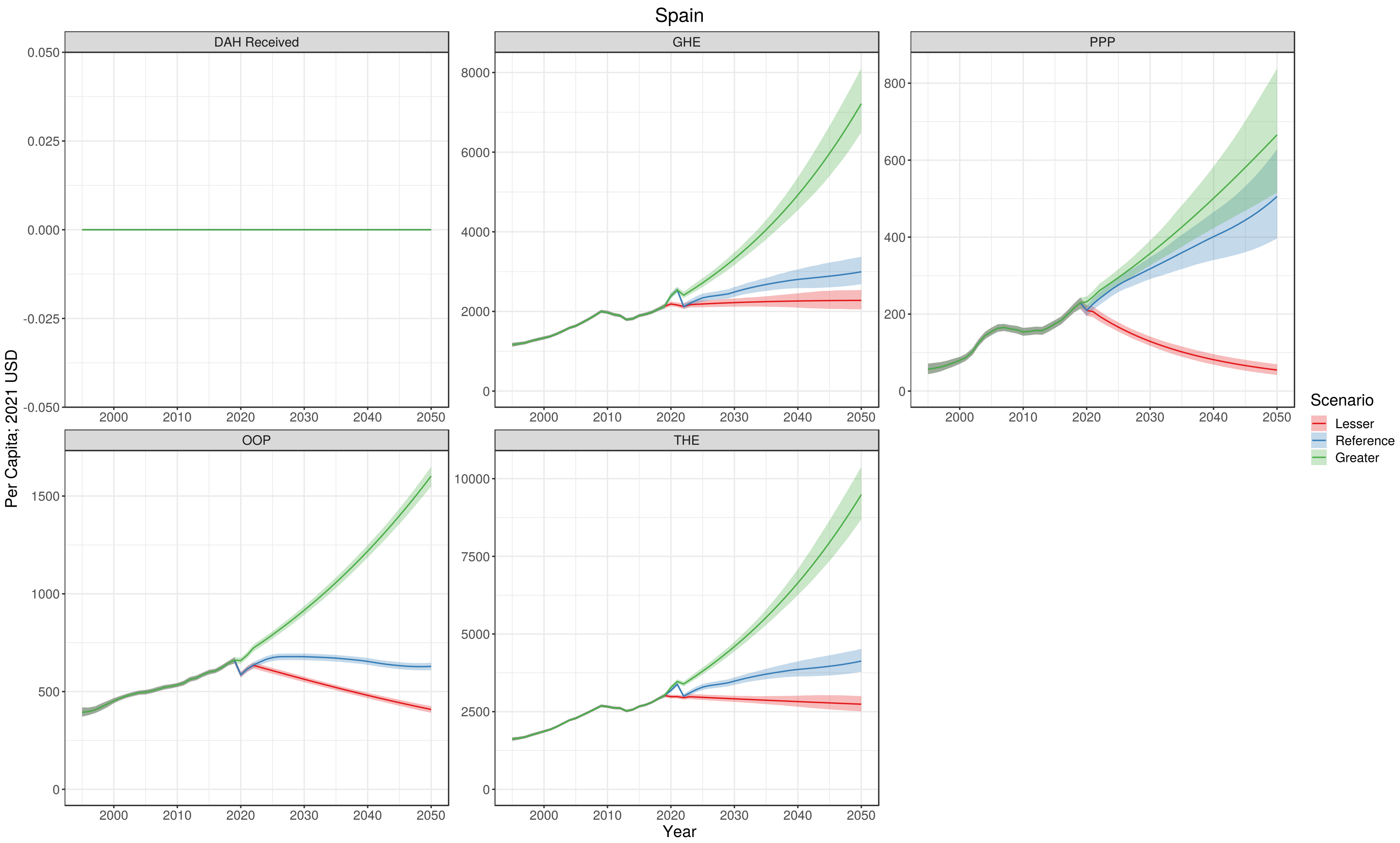

Spain
DAH Received
GHE
PPP
OOP
THE
Per Capita; 2021 USD
Year
Scenario
Lesser
Reference
Greater

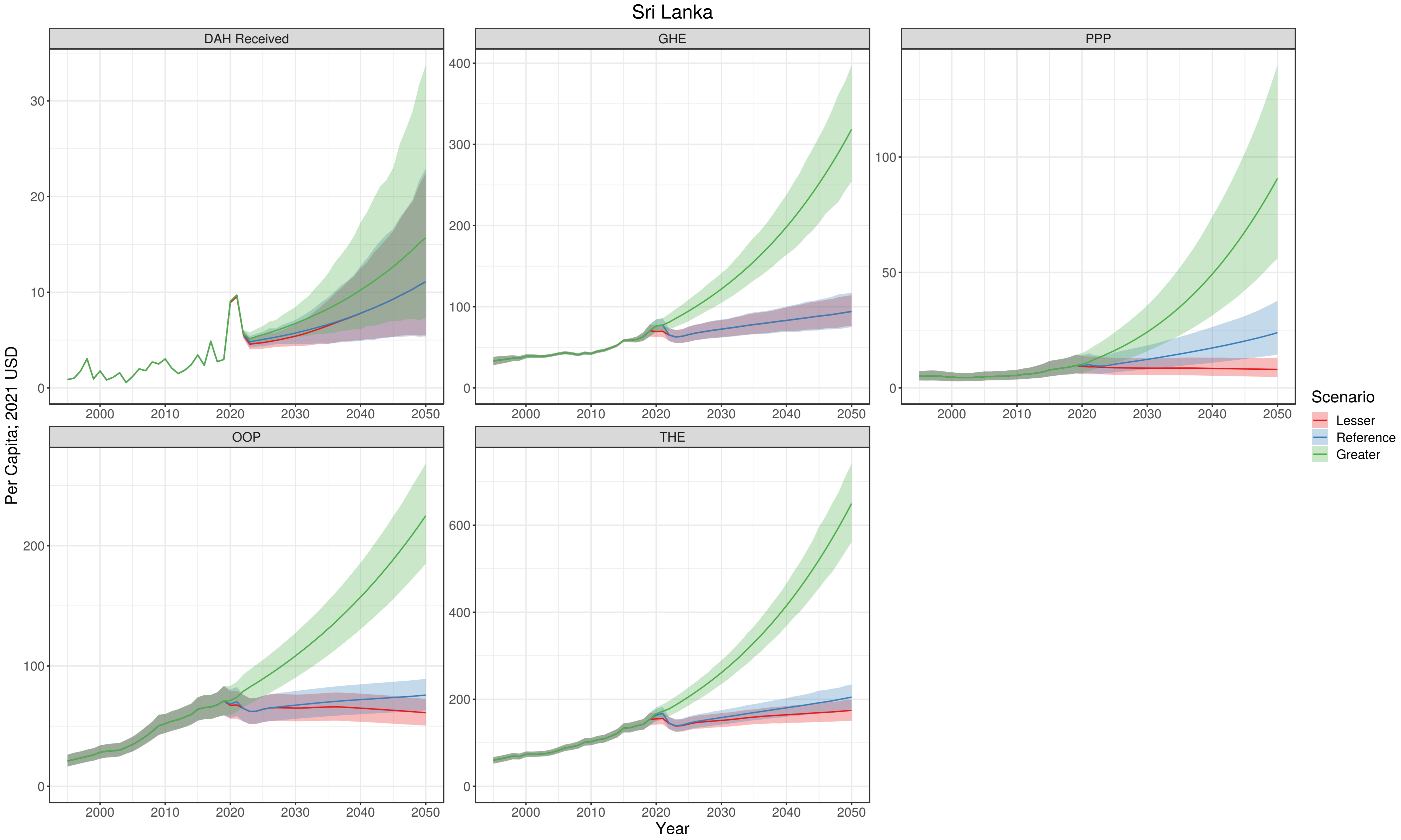

Sri Lanka
DAH Received
GHE
PPP
OOP
THE
Per Capita; 2021 USD
Year
Scenario
Lesser
Reference
Greater

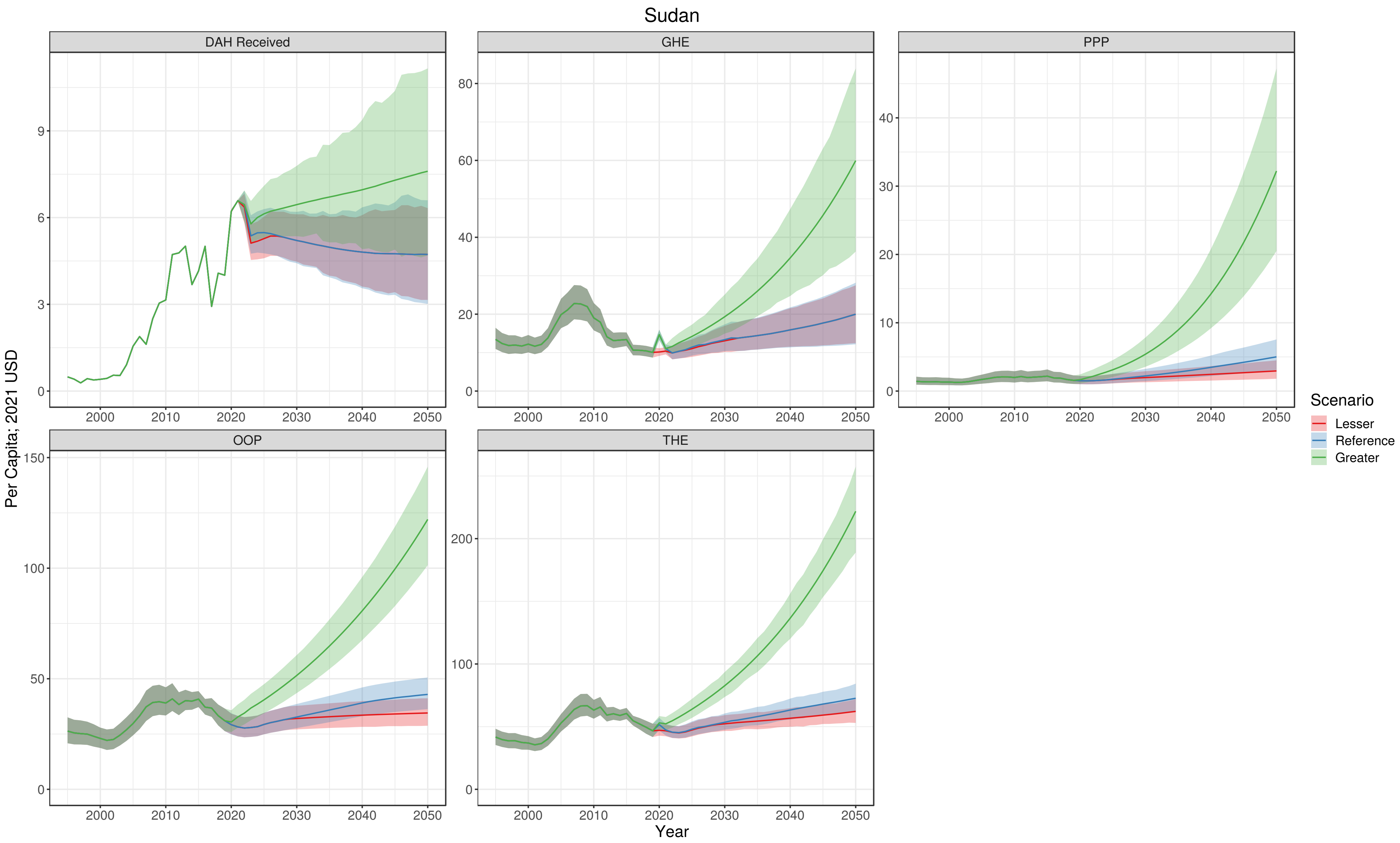

Sudan
DAH Received
GHE
PPP
OOP
THE
Per Capita; 2021 USD
Year
Scenario
Lesser
Reference
Greater
0
3
6
9
0
20
40
60
80
0
10
20
30
40
0
50
100
150
0
100
200
2000
2010
2020
2030
2040
2050

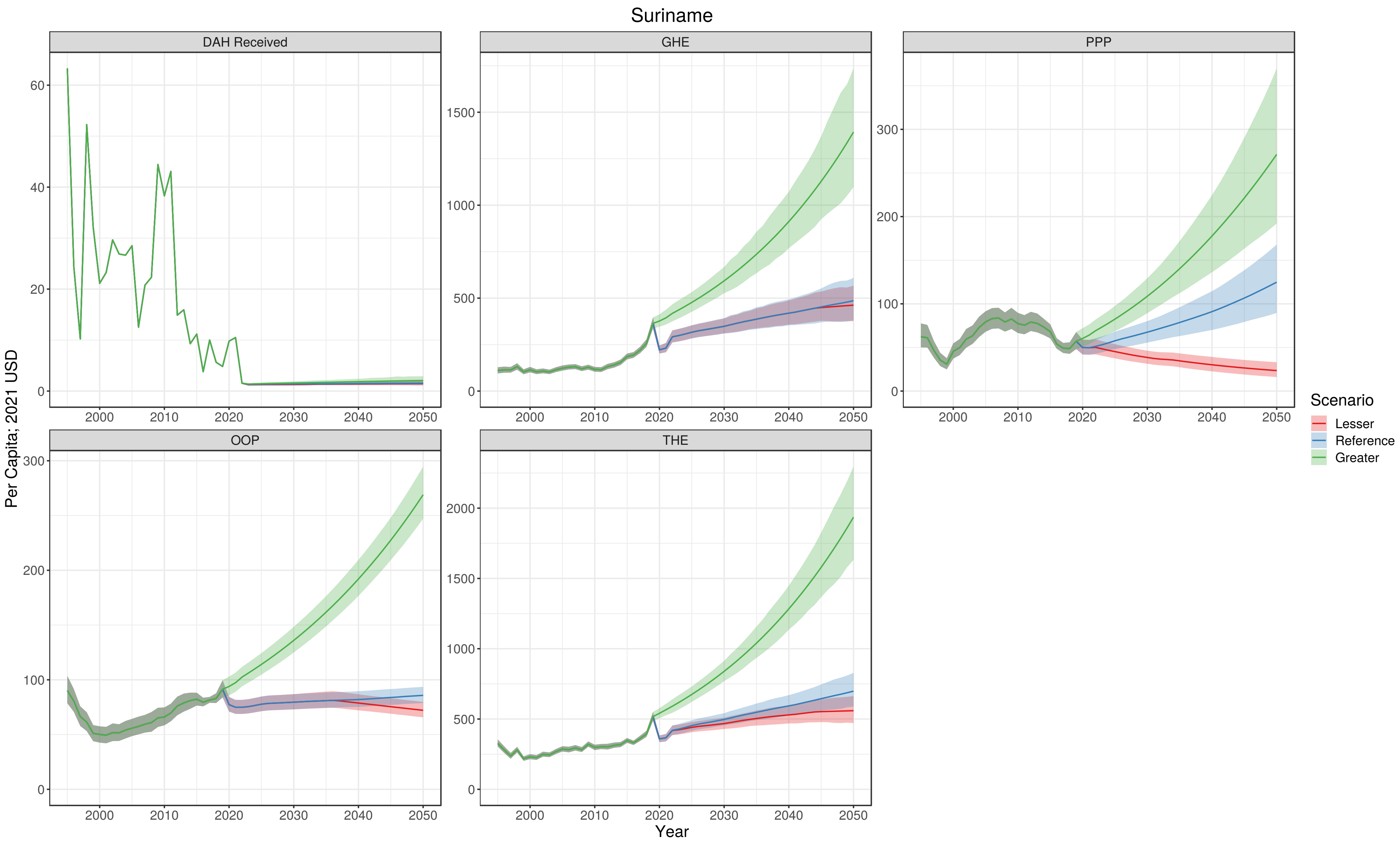

Suriname
DAH Received
GHE
PPP
OOP
THE
Per Capita; 2021 USD
Year
Scenario
Lesser
Reference
Greater

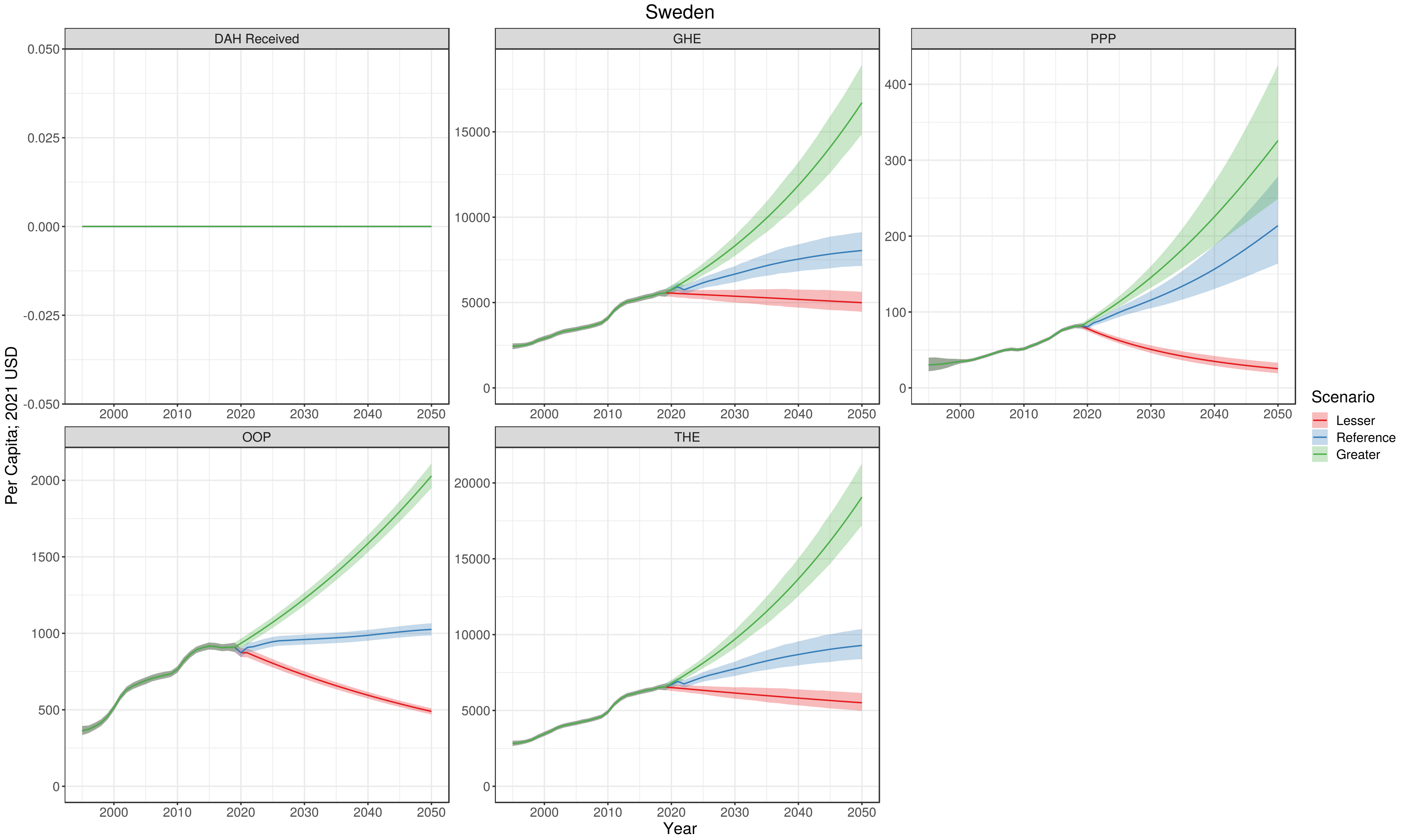
Sweden
DAH Received
GHE
PPP
OOP
THE
Per Capita; 2021 USD
Year
Scenario
Lesser
Reference
Greater

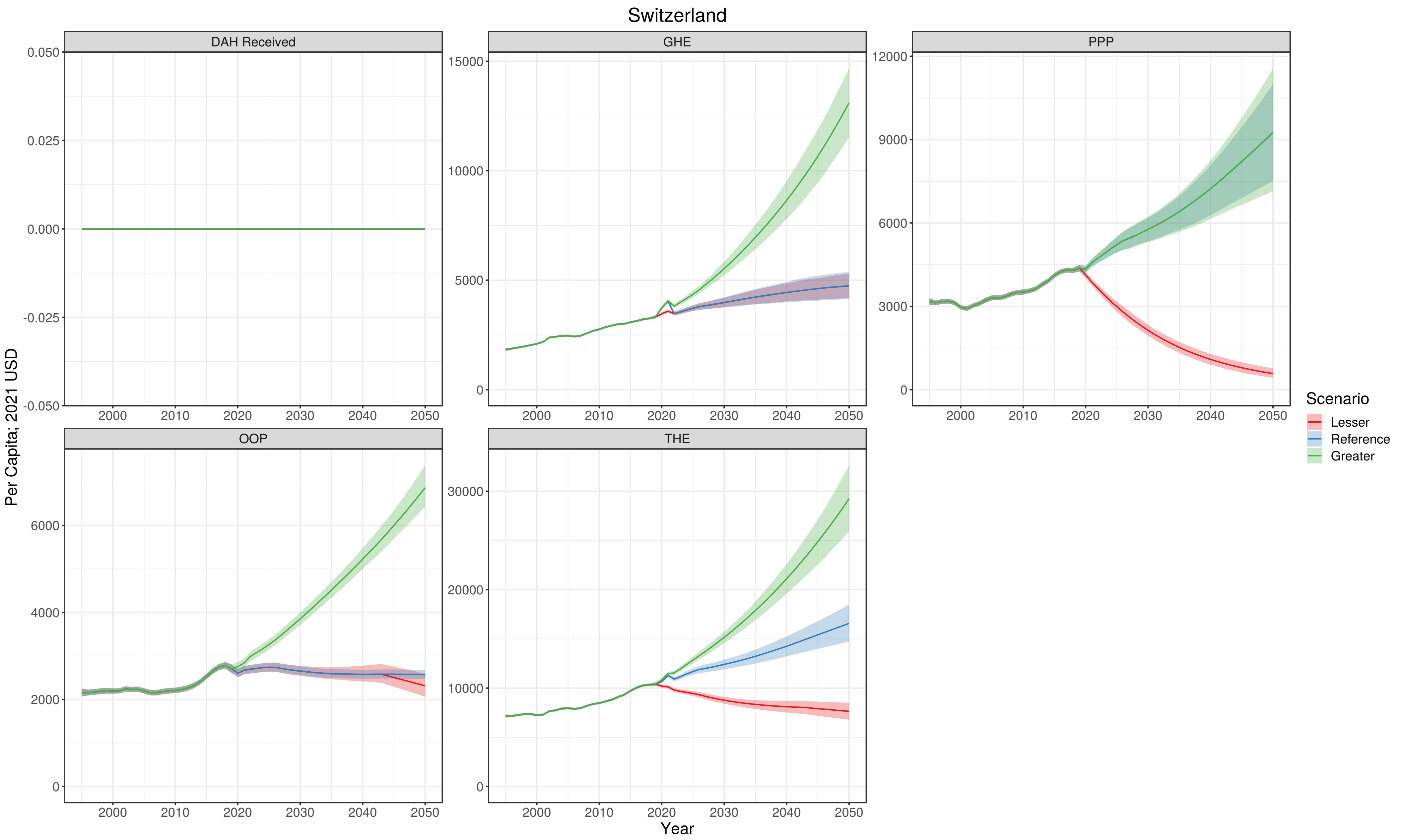

Switzerland
DAH Received
GHE
PPP
OOP
THE
Per Capita; 2021 USD
Year
Scenario
Lesser
Reference
Greater
0.050
0.025
0.000
-0.025
-0.050
15000
10000
5000
0
12000
9000
6000
3000
6000
4000
2000
30000
20000
2000
2010
2020
2030
2040
2050

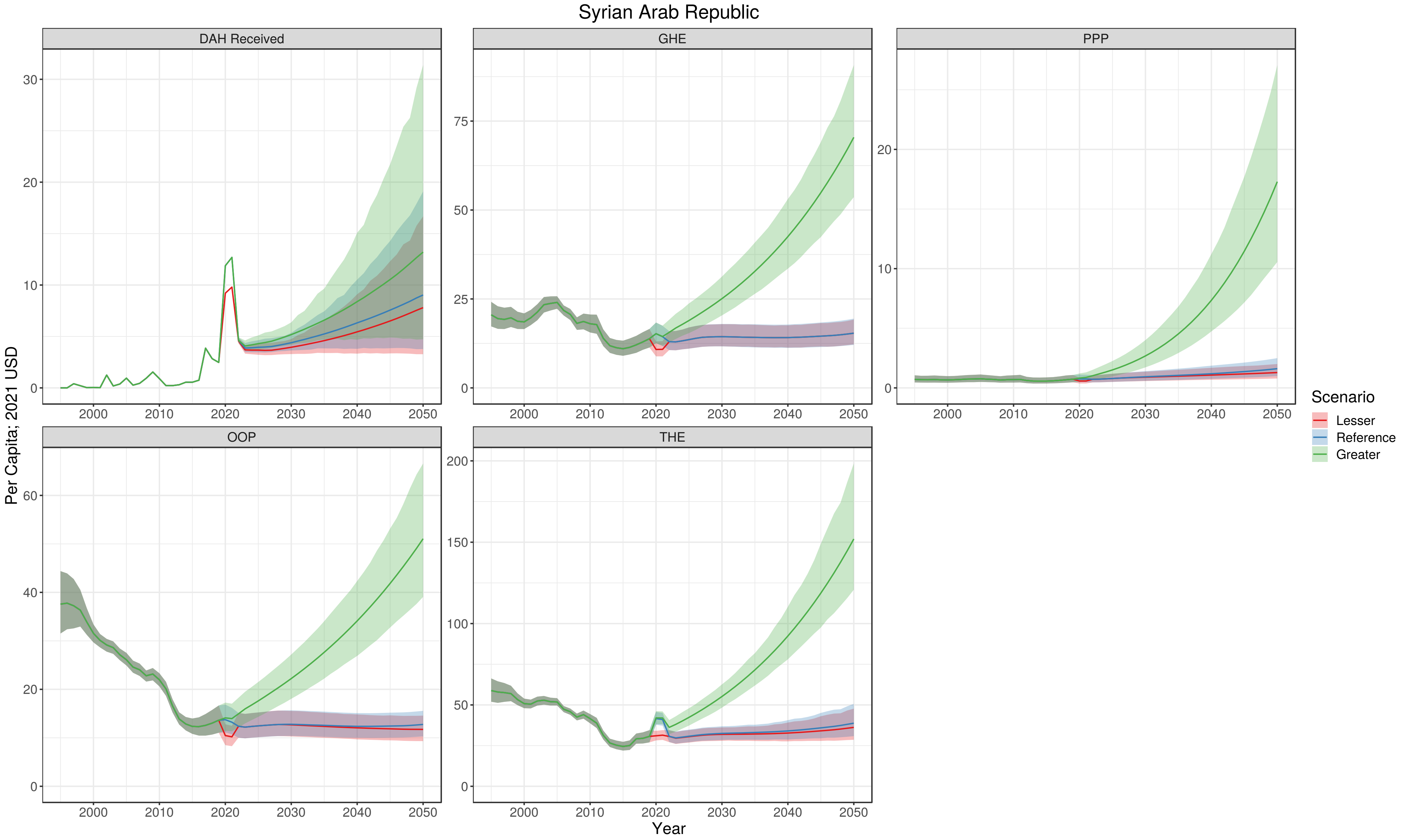
Syrian Arab Republic
DAH Received
GHE
PPP
OOP
THE
Per Capita; 2021 USD
Year
Scenario
Lesser
Reference
Greater

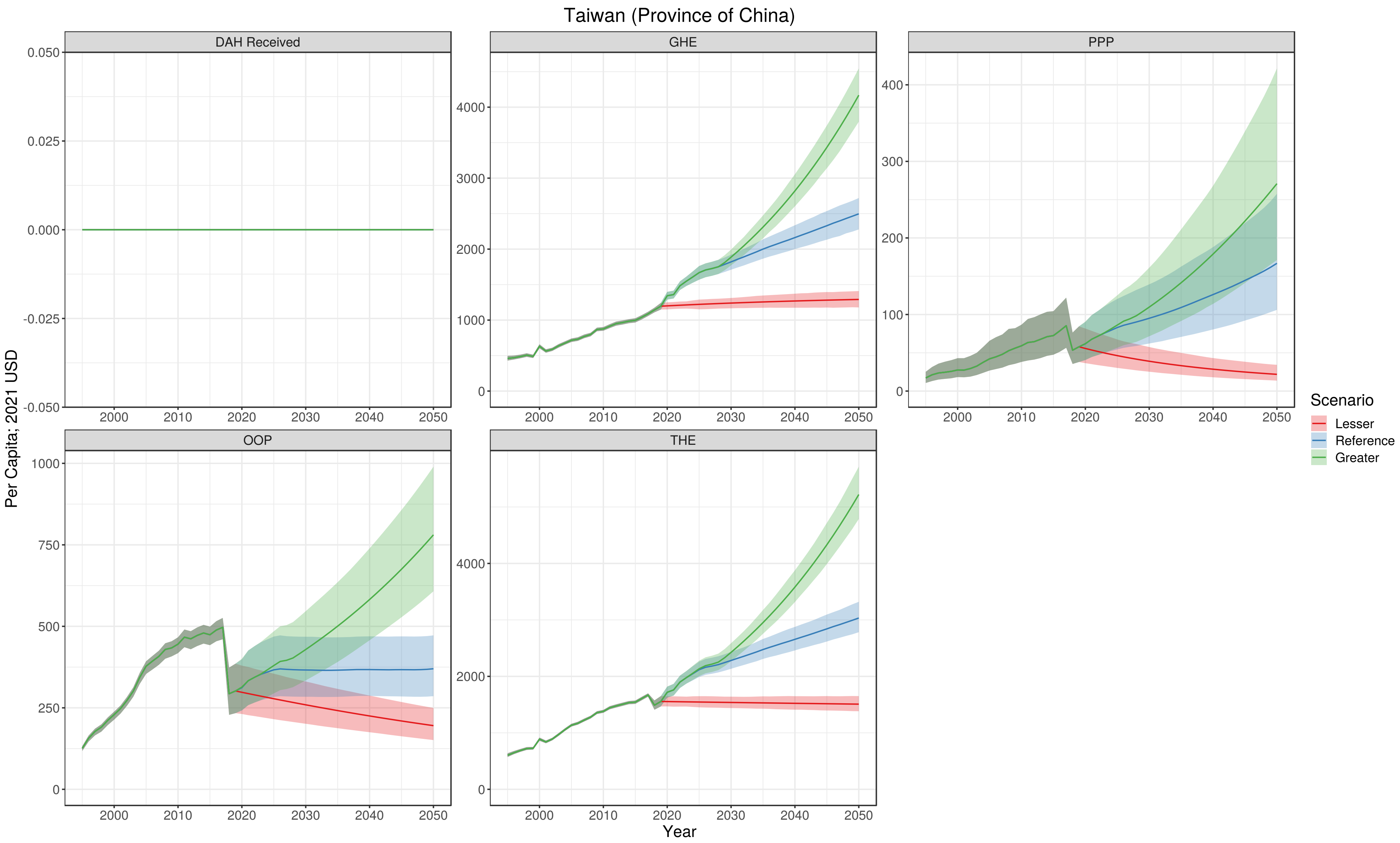

Taiwan (Province of China)
DAH Received
GHE
PPP
OOP
THE
Per Capita; 2021 USD
Year
Scenario
Lesser
Reference
Greater
0.050
0.025
0.000
-0.025
-0.050
4000
3000
2000
1000
0
400
300
200
100
1000
750
500
250
2000
2010
2020
2030
2040
2050

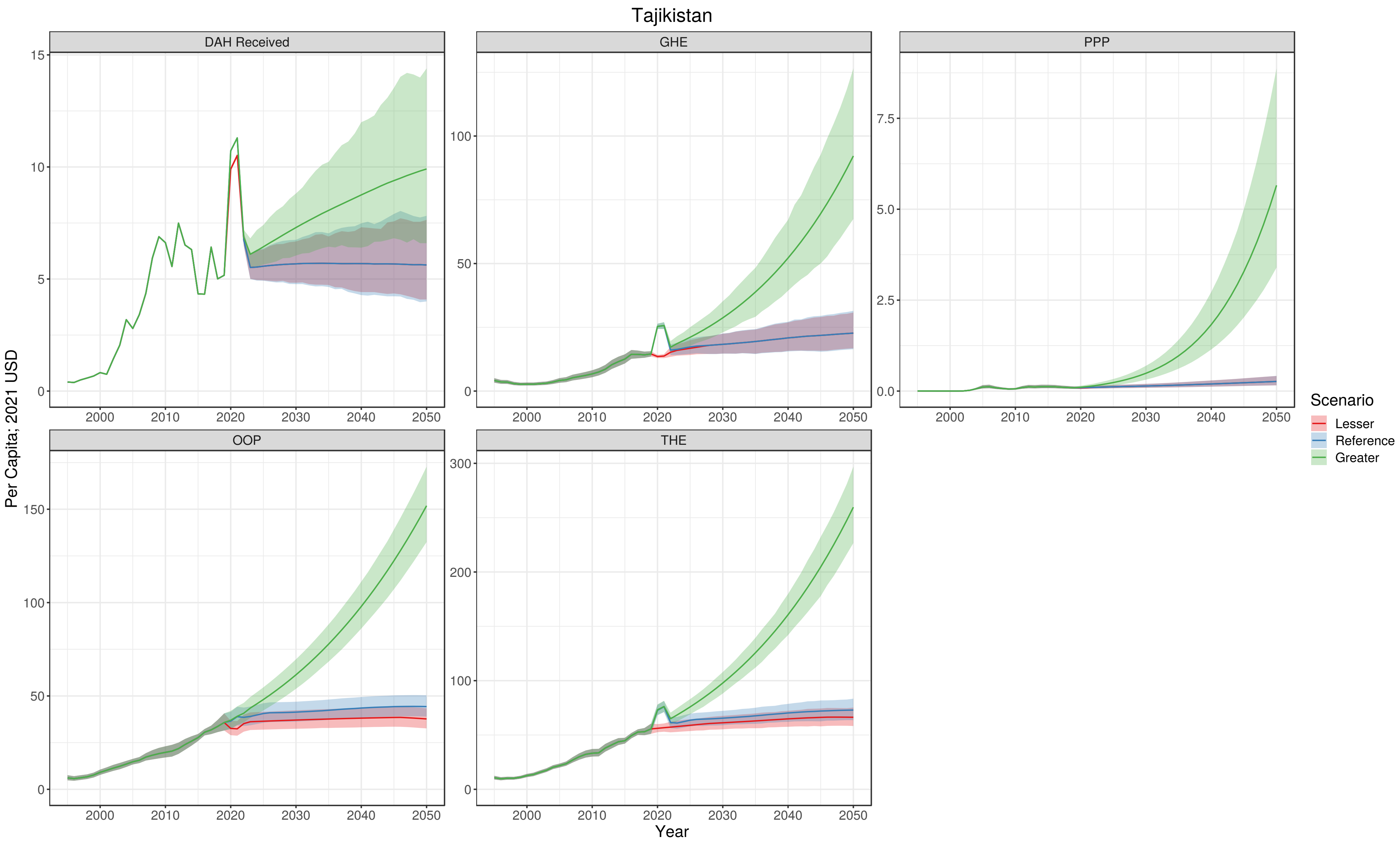

Tajikistan
DAH Received
GHE
PPP
OOP
THE
Per Capita; 2021 USD
Year
Scenario
Lesser
Reference
Greater

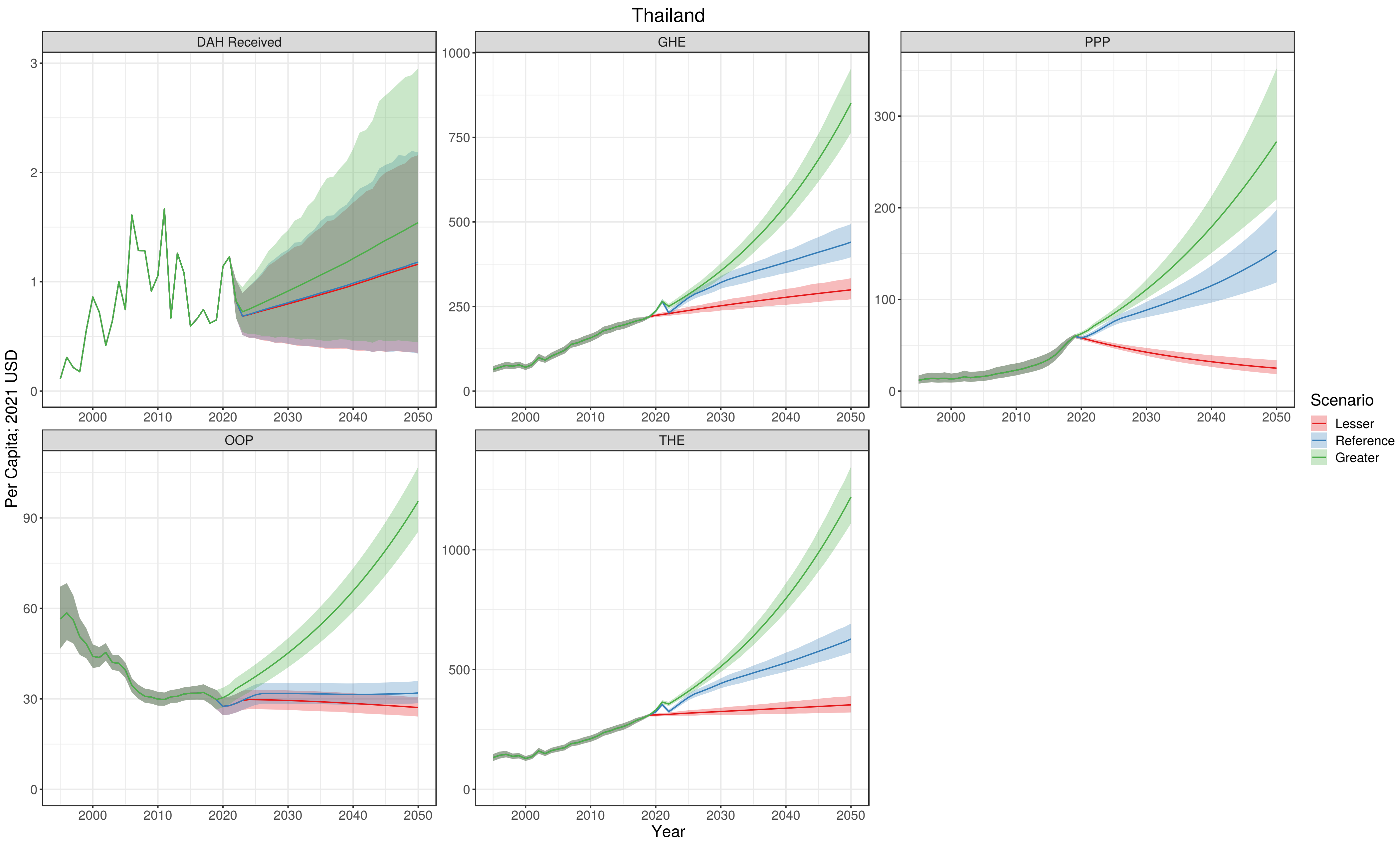

Thailand
DAH Received
GHE
PPP
OOP
THE
Per Capita; 2021 USD
Year
Scenario
Lesser
Reference
Greater

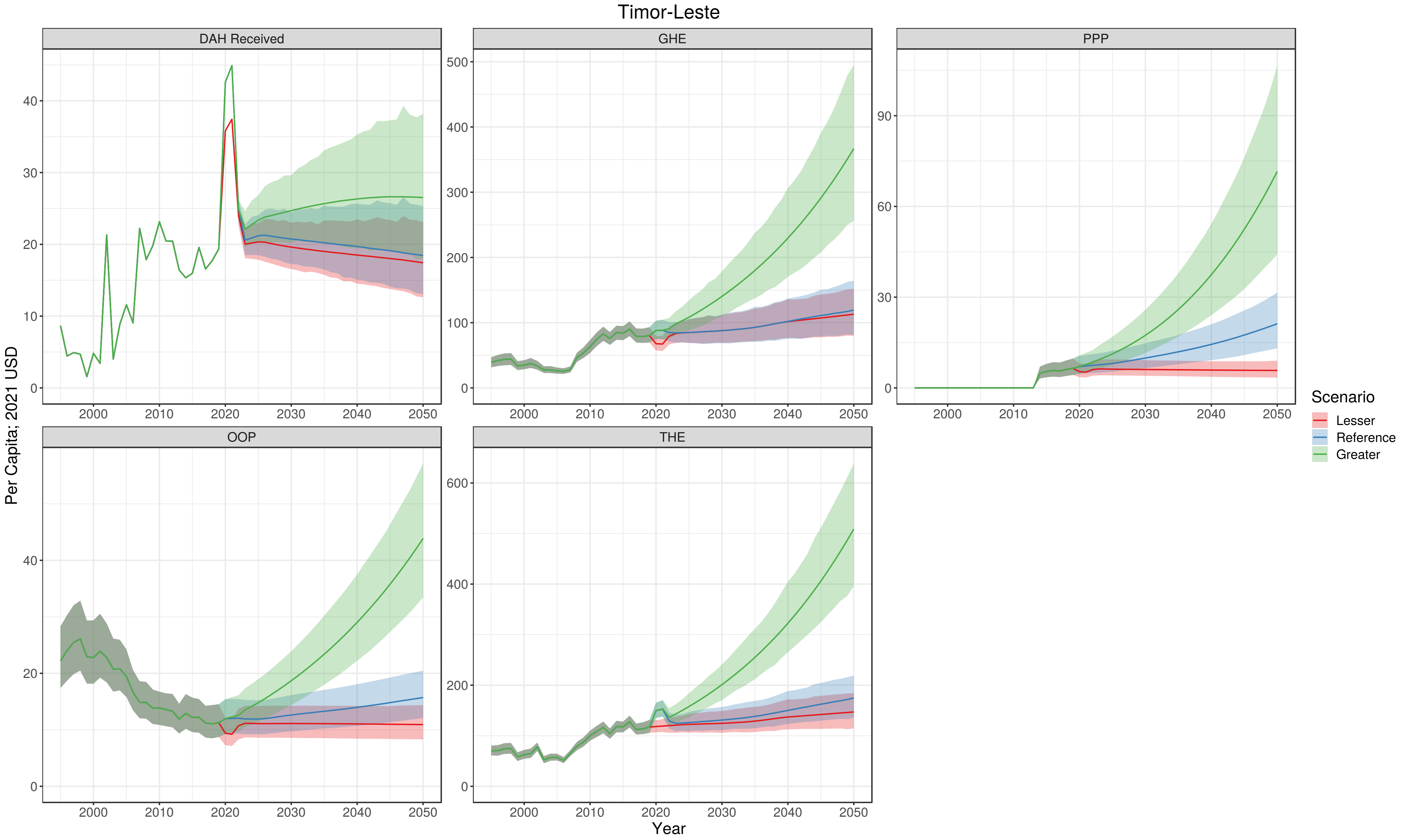
Timor-Leste
DAH Received
GHE
PPP
OOP
THE
Per Capita; 2021 USD
Year
Scenario
Lesser
Reference
Greater

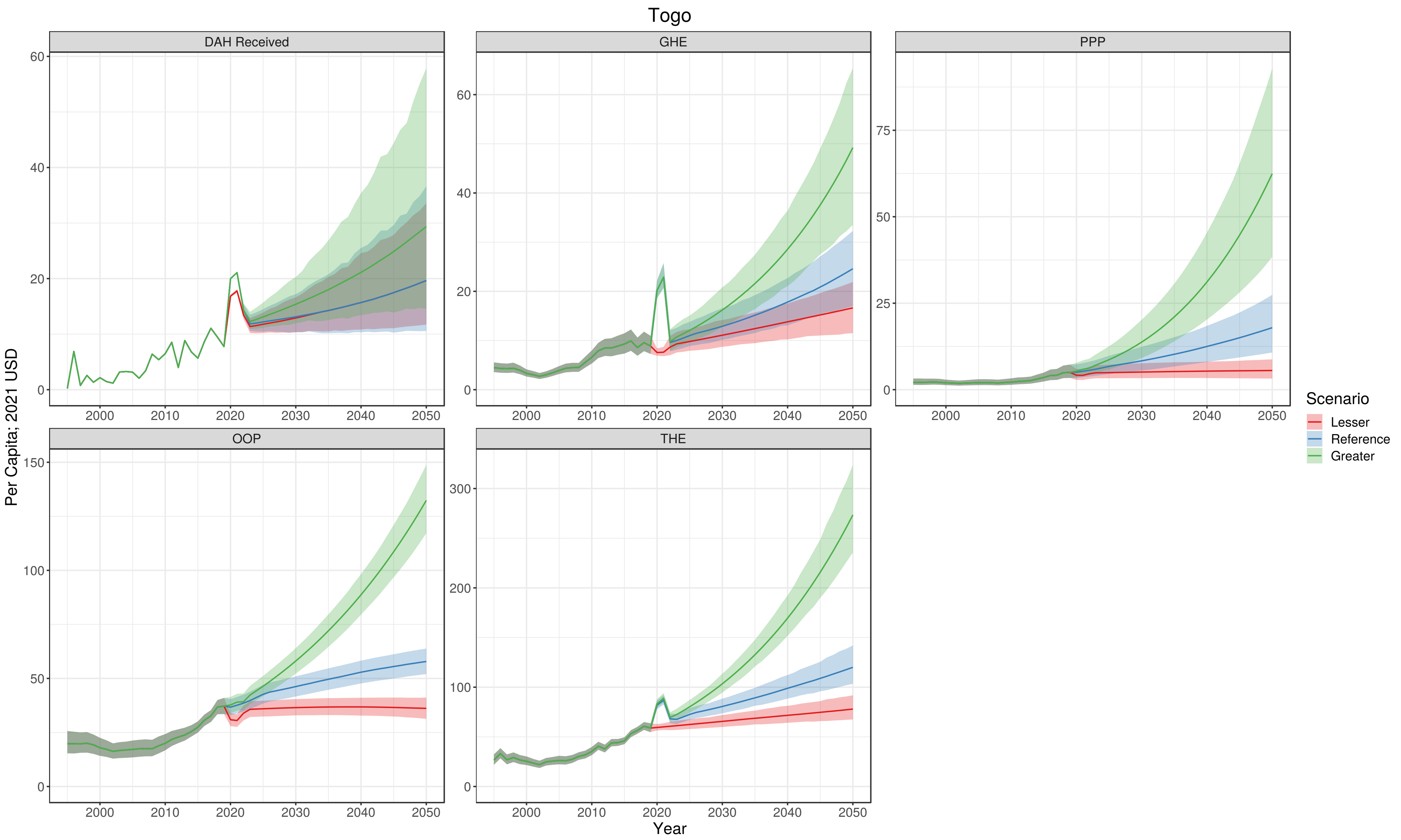
Togo
DAH Received
GHE
PPP
OOP
THE
Per Capita; 2021 USD
Year
Scenario
Lesser
Reference
Greater
0
20
40
60
25
50
75
100
150
200
300
2000
2010
2020
2030
2040
2050

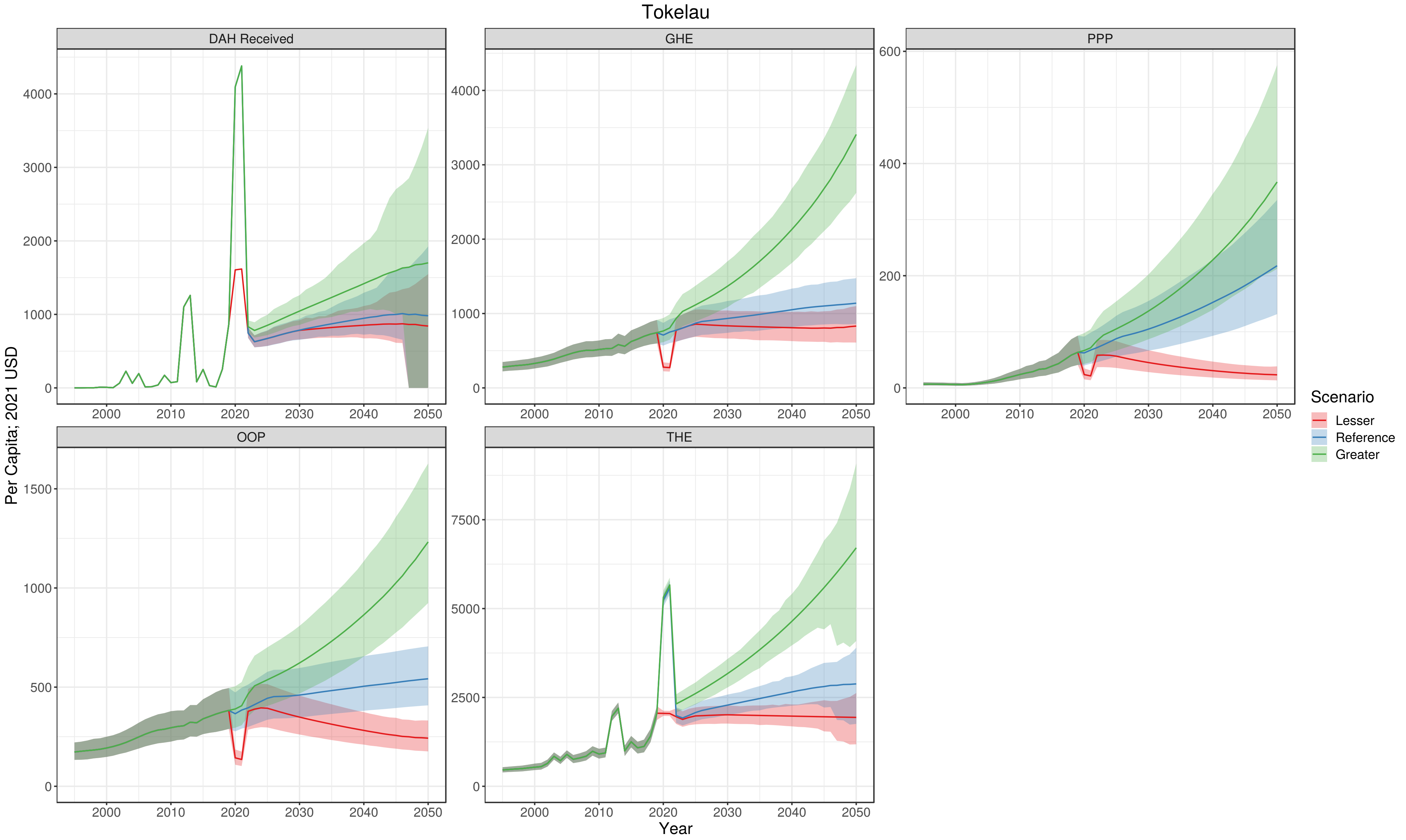

Tokelau
DAH Received
GHE
PPP
OOP
THE
Per Capita; 2021 USD
Year
Scenario
Lesser
Reference
Greater
0
1000
2000
3000
4000
200
400
600
500
1500
2500
5000
7500
2000
2010
2020
2030
2040
2050

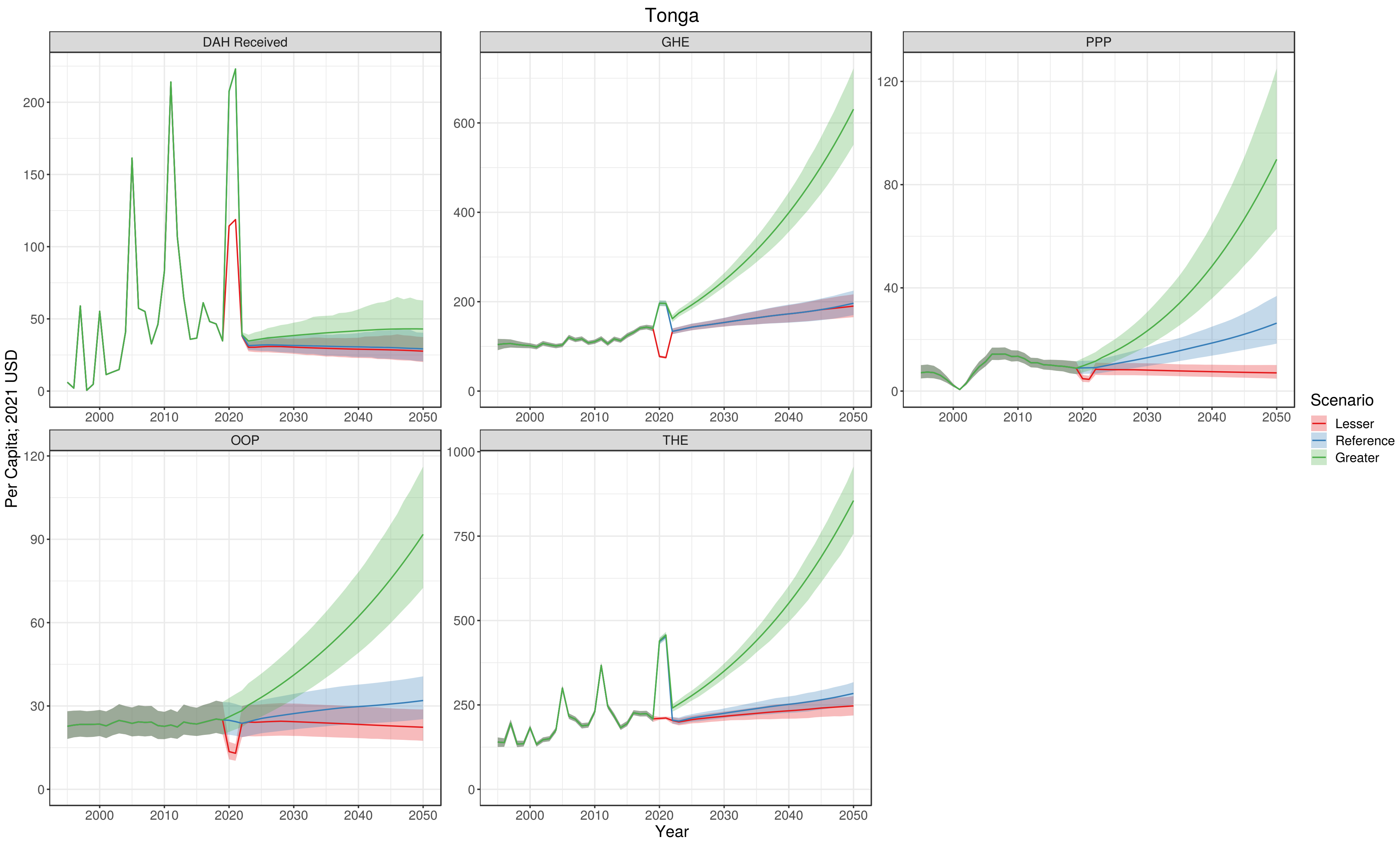
Tonga
DAH Received
GHE
PPP
OOP
THE
Per Capita; 2021 USD
Year
Scenario
Lesser
Reference
Greater
200
150
100
50
0
600
400
200
0
120
80
40
0
120
90
60
30
0
1000
750
500
250
0
2000
2010
2020
2030
2040
2050

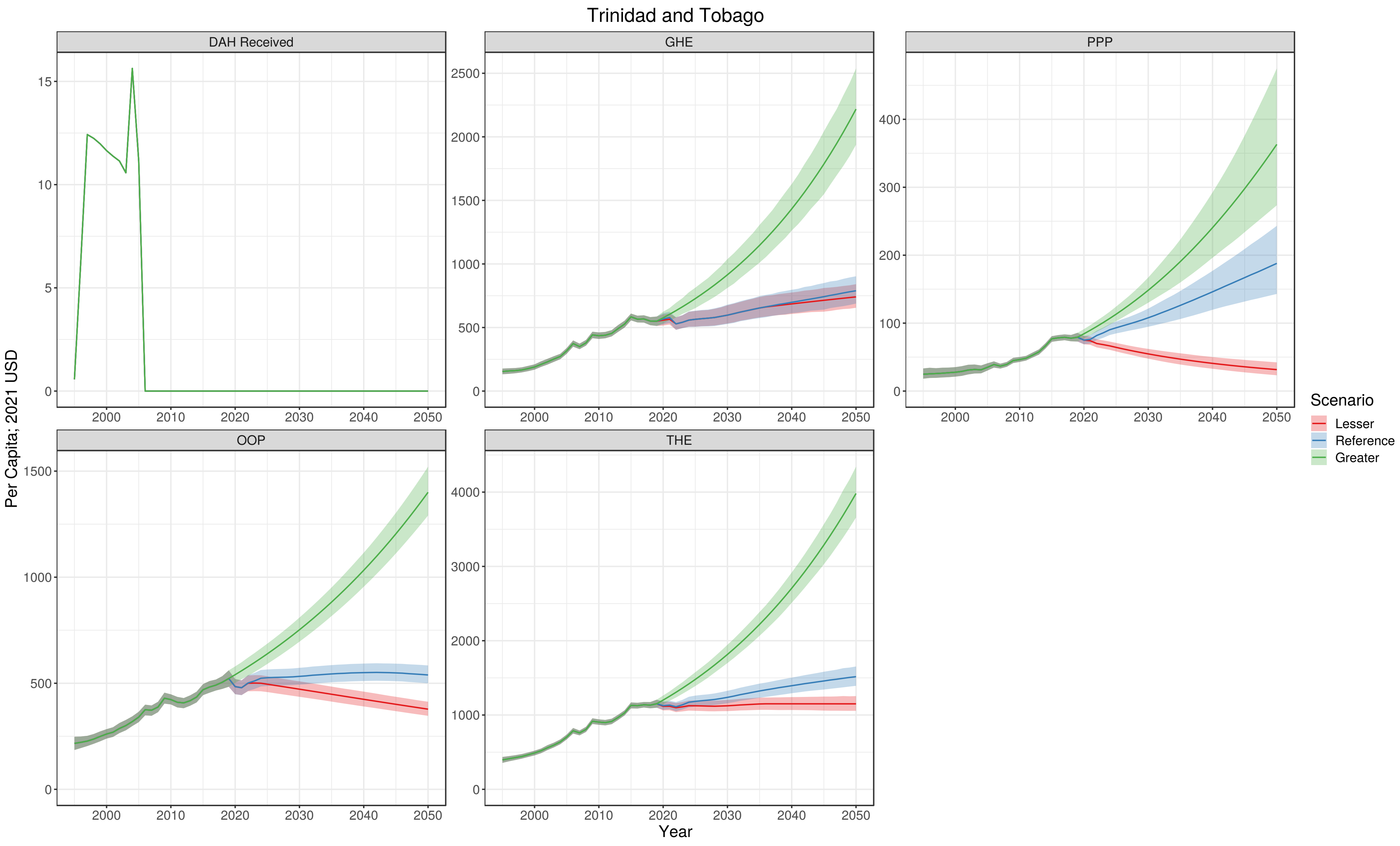

Trinidad and Tobago
DAH Received
GHE
PPP
OOP
THE
Per Capita; 2021 USD
Year
Scenario
Lesser
Reference
Greater

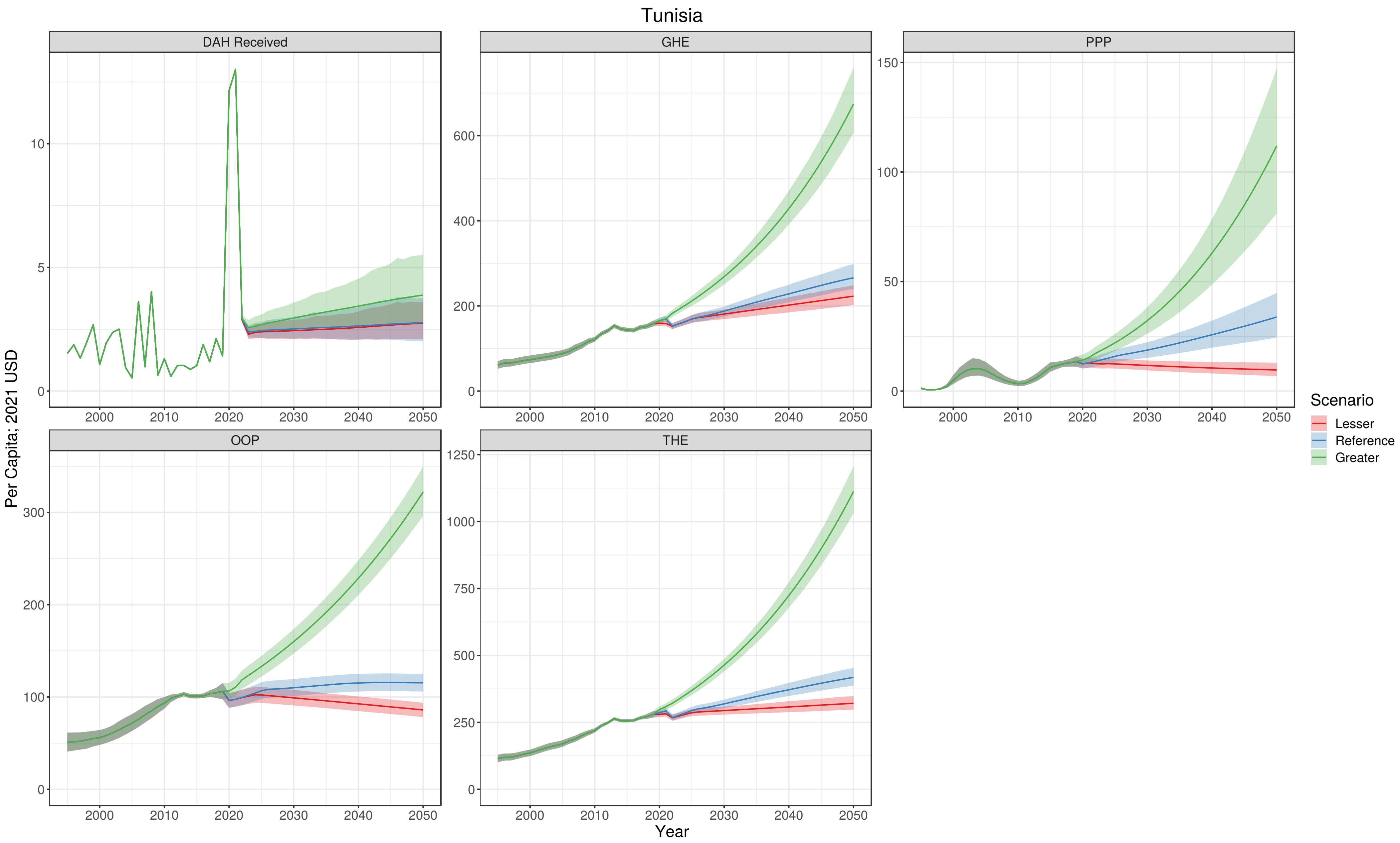

Tunisia
DAH Received
GHE
PPP
OOP
THE
Per Capita; 2021 USD
Year
Scenario
Lesser
Reference
Greater

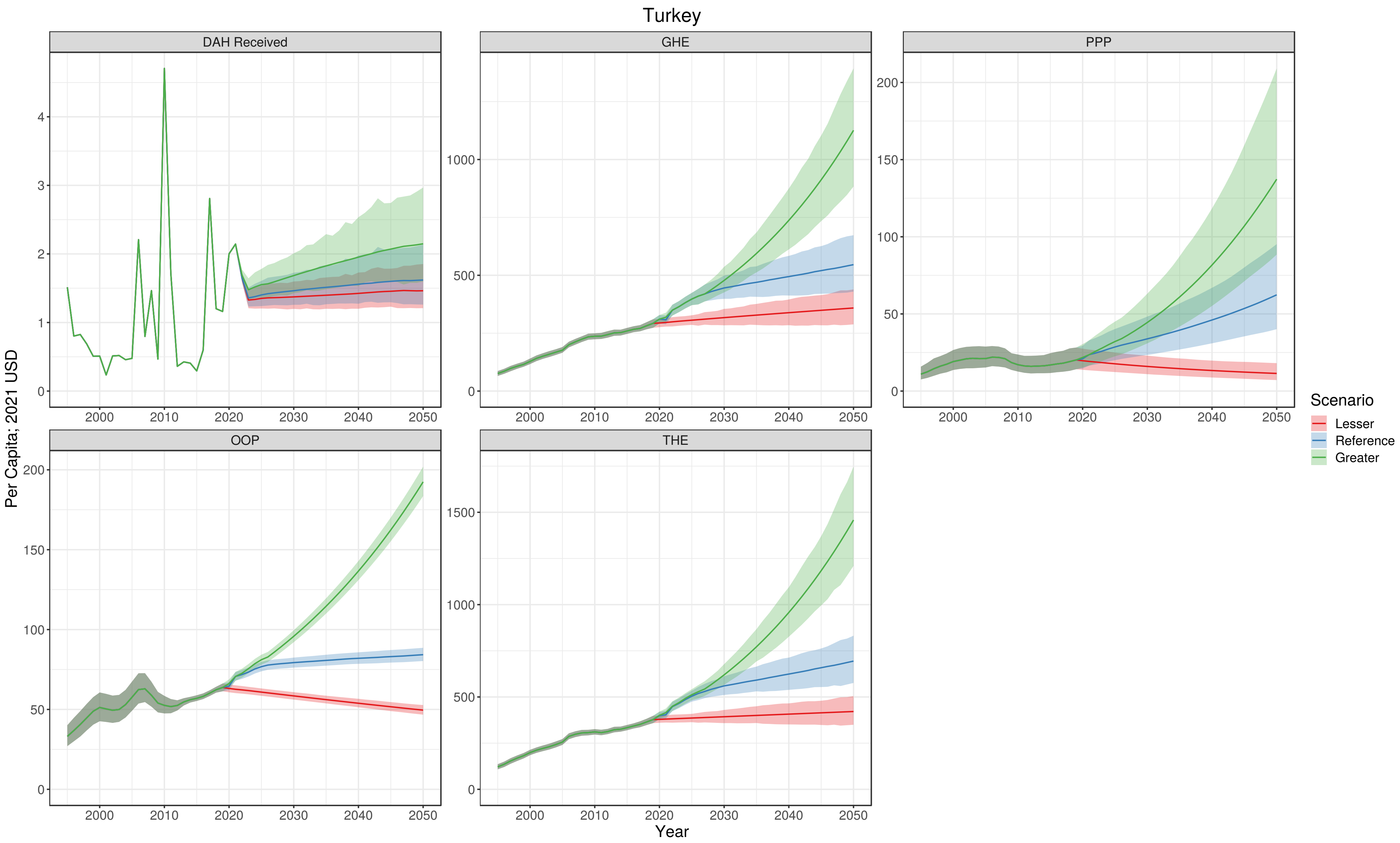

Turkey
DAH Received
GHE
PPP
OOP
THE
Per Capita; 2021 USD
Year
Scenario
Lesser
Reference
Greater

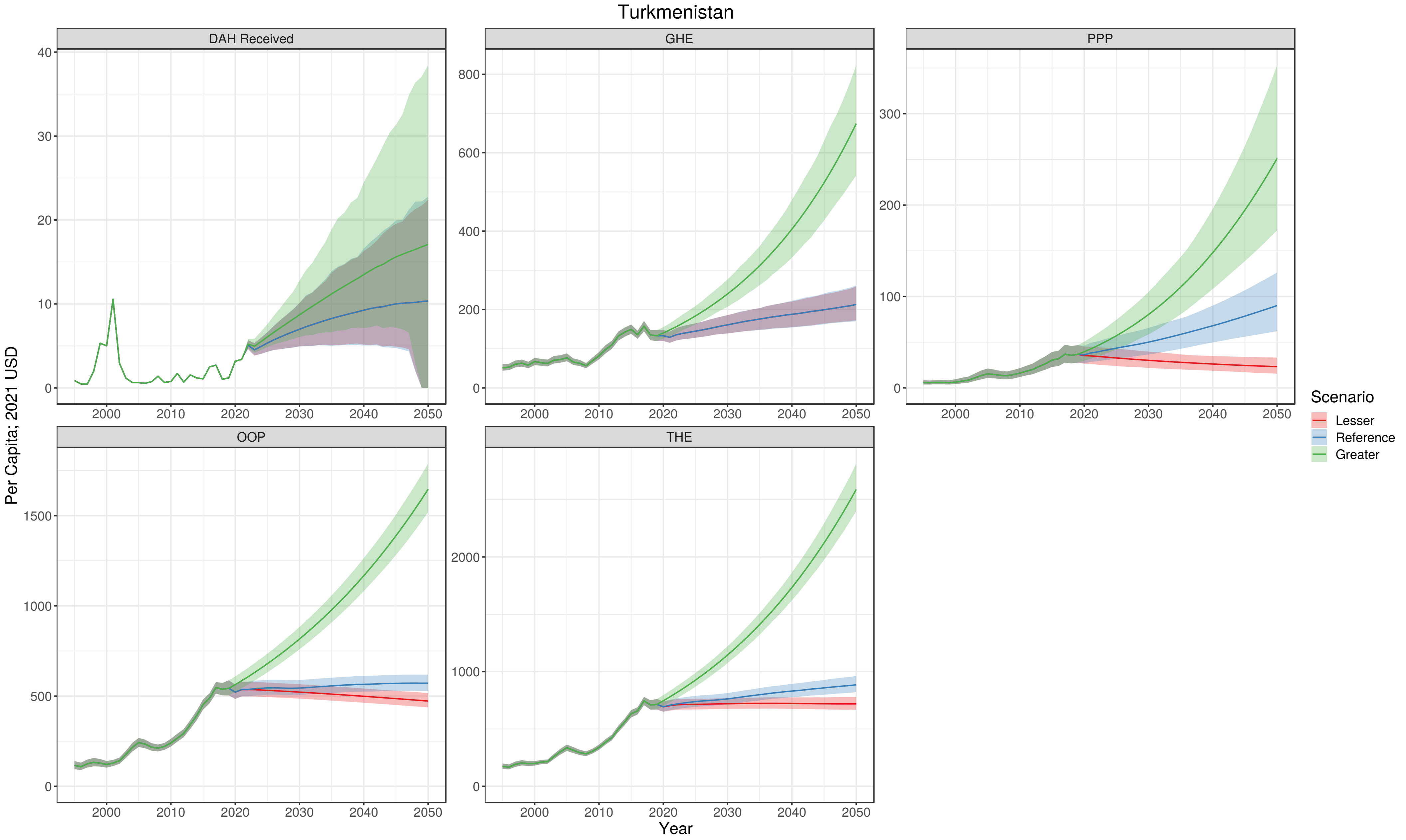
Turkmenistan
DAH Received
GHE
PPP
OOP
THE
Per Capita; 2021 USD
Year
Scenario
Lesser
Reference
Greater
0
10
20
30
40
200
400
600
800
100
300
500
1000
1500
2000
2010
2020
2030
2040
2050

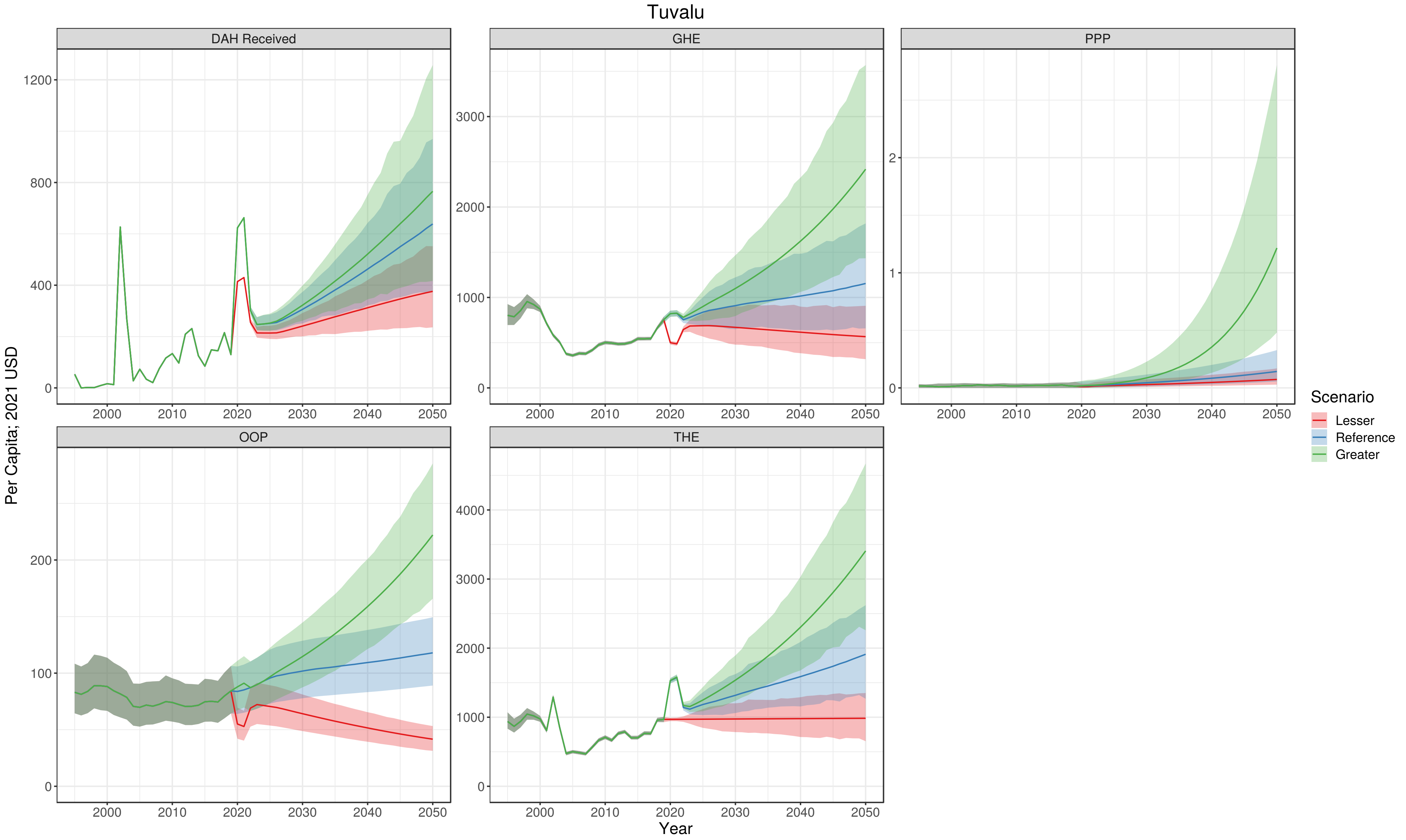

Tuvalu
DAH Received
GHE
PPP
OOP
THE
Per Capita; 2021 USD
Year
Scenario
Lesser
Reference
Greater

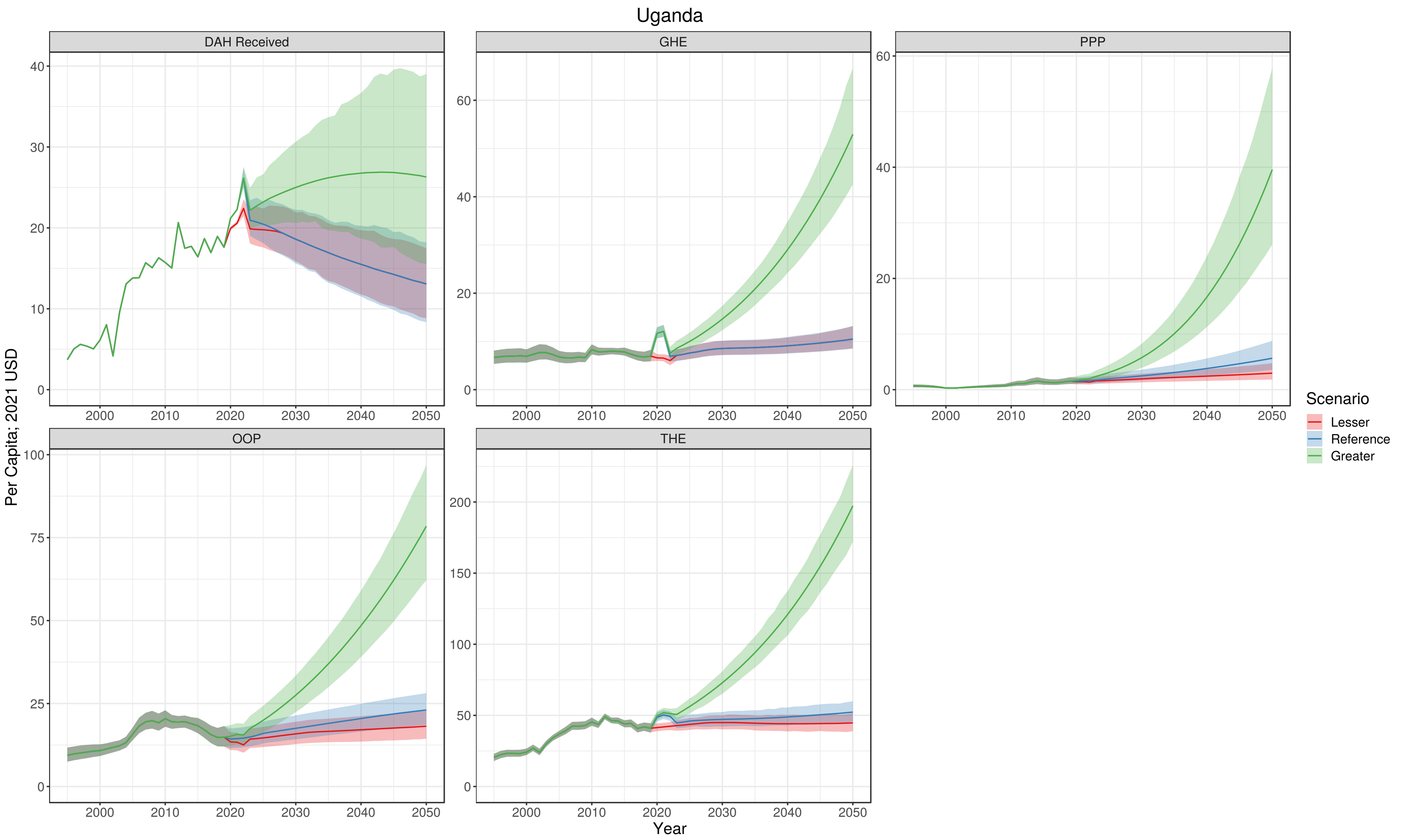
Uganda
DAH Received
GHE
PPP
OOP
THE
Per Capita; 2021 USD
Year
Scenario
Lesser
Reference
Greater

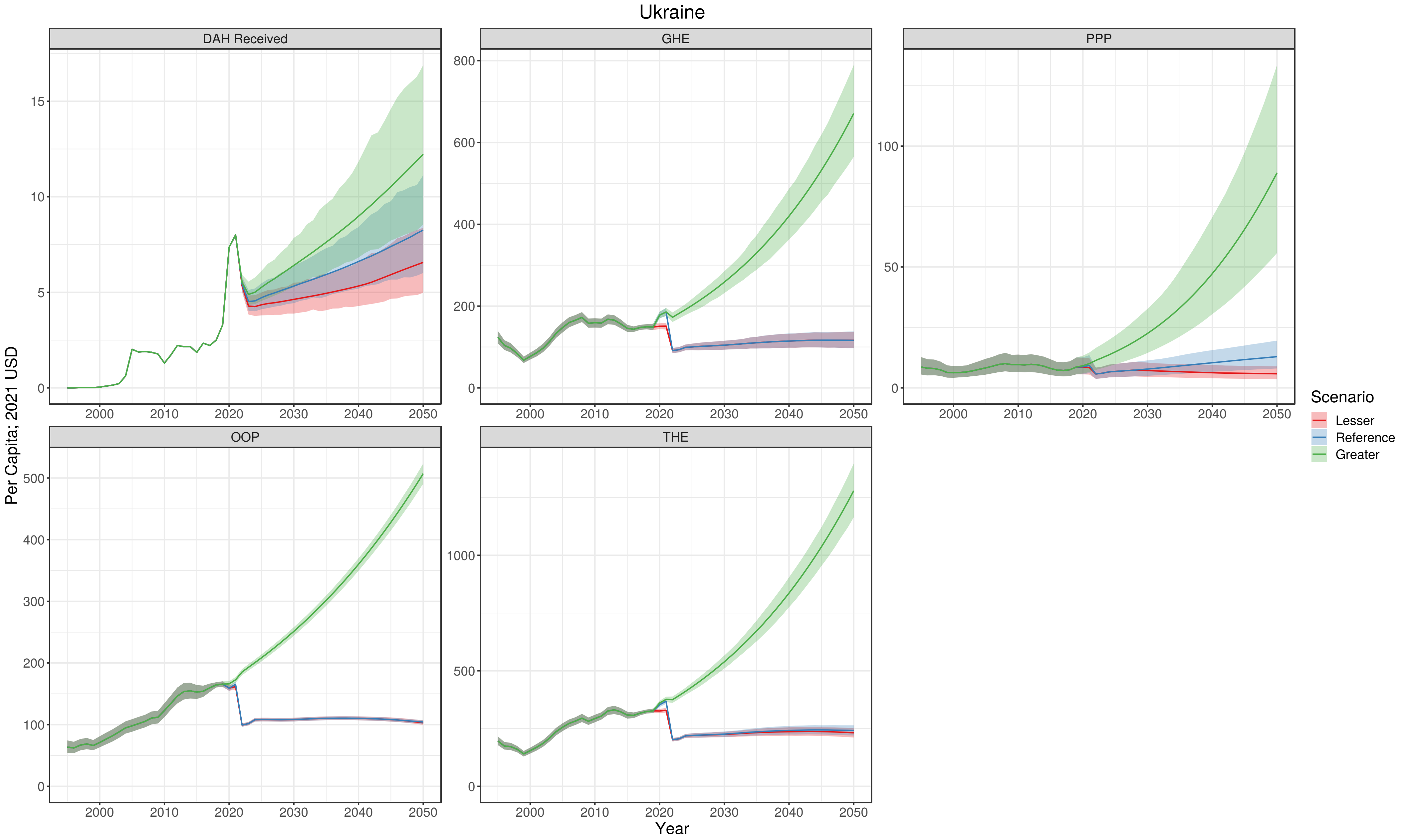

Ukraine
DAH Received
GHE
PPP
OOP
THE
Per Capita; 2021 USD
Year
Scenario
Lesser
Reference
Greater
2000
2010
2020
2030
2040
2050
0
5
10
15
200
400
600
800
50
100
300
500
1000

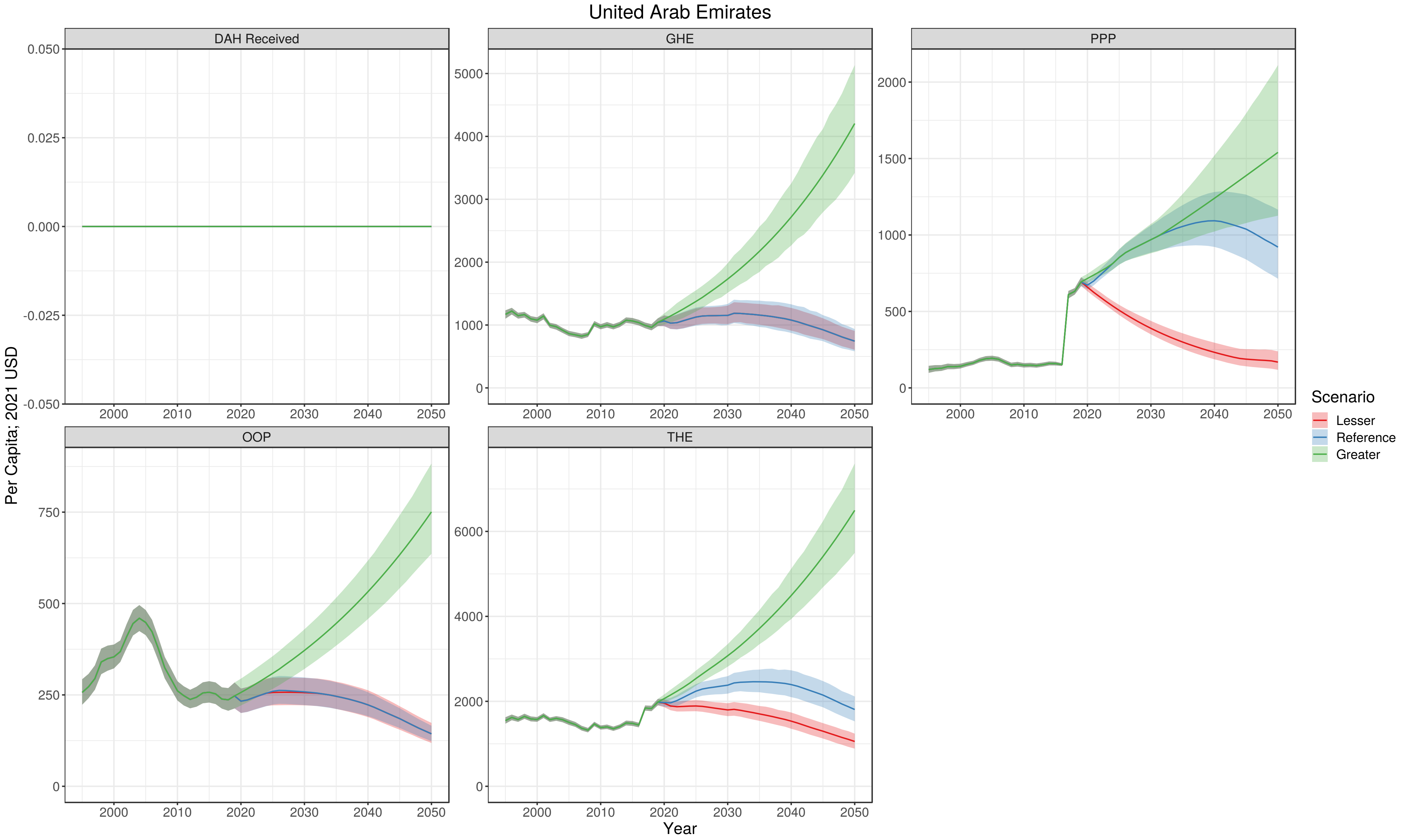

United Arab Emirates
DAH Received
GHE
PPP
OOP
THE
Per Capita; 2021 USD
Year
Scenario
Lesser
Reference
Greater

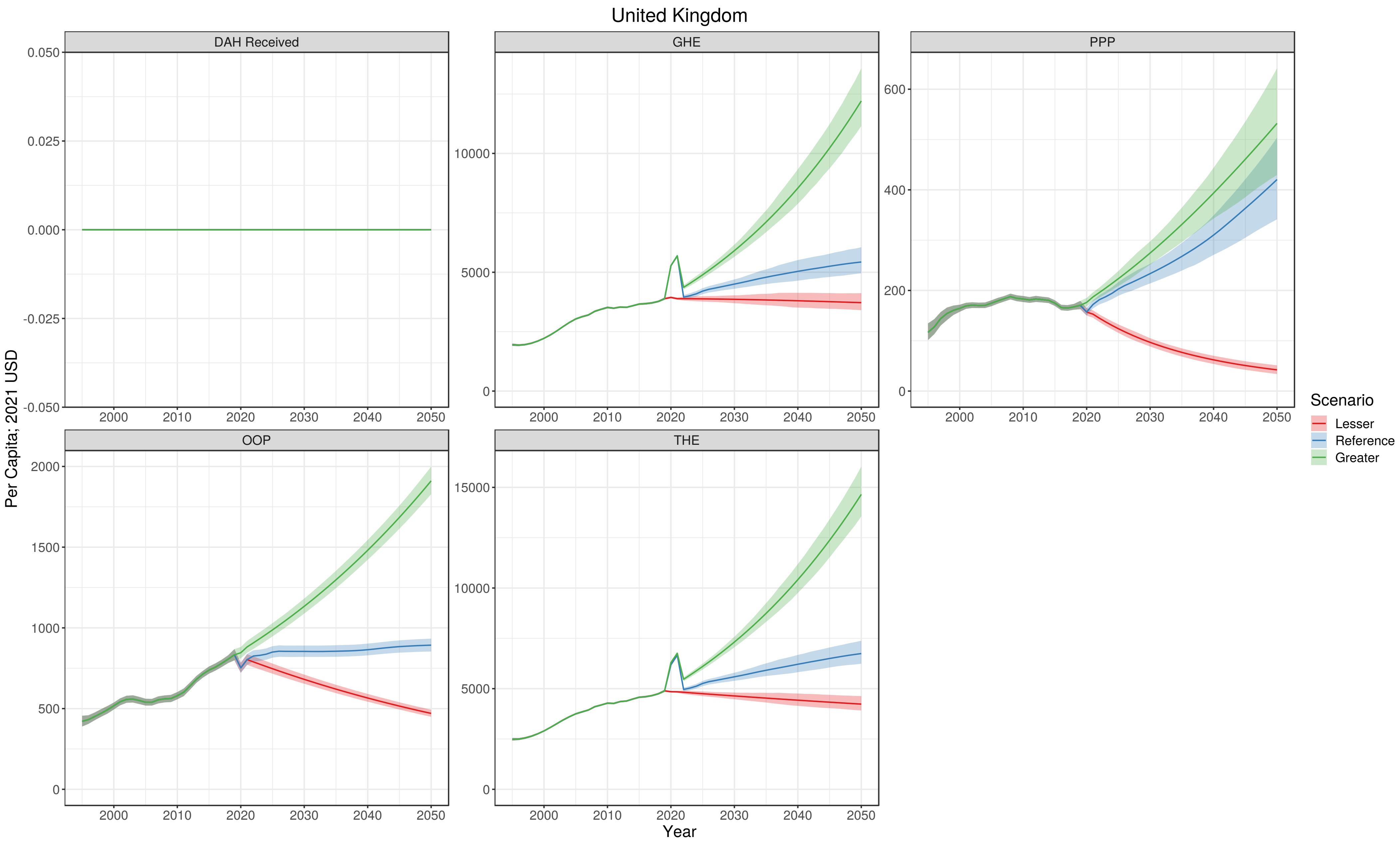

United Kingdom
DAH Received
GHE
PPP
OOP
THE
Per Capita; 2021 USD
Year
Scenario
Lesser
Reference
Greater
0.050
0.025
0.000
-0.025
-0.050
10000
5000
0
600
400
200
2000
1500
1000
500
15000
2000
2010
2020
2030
2040
2050

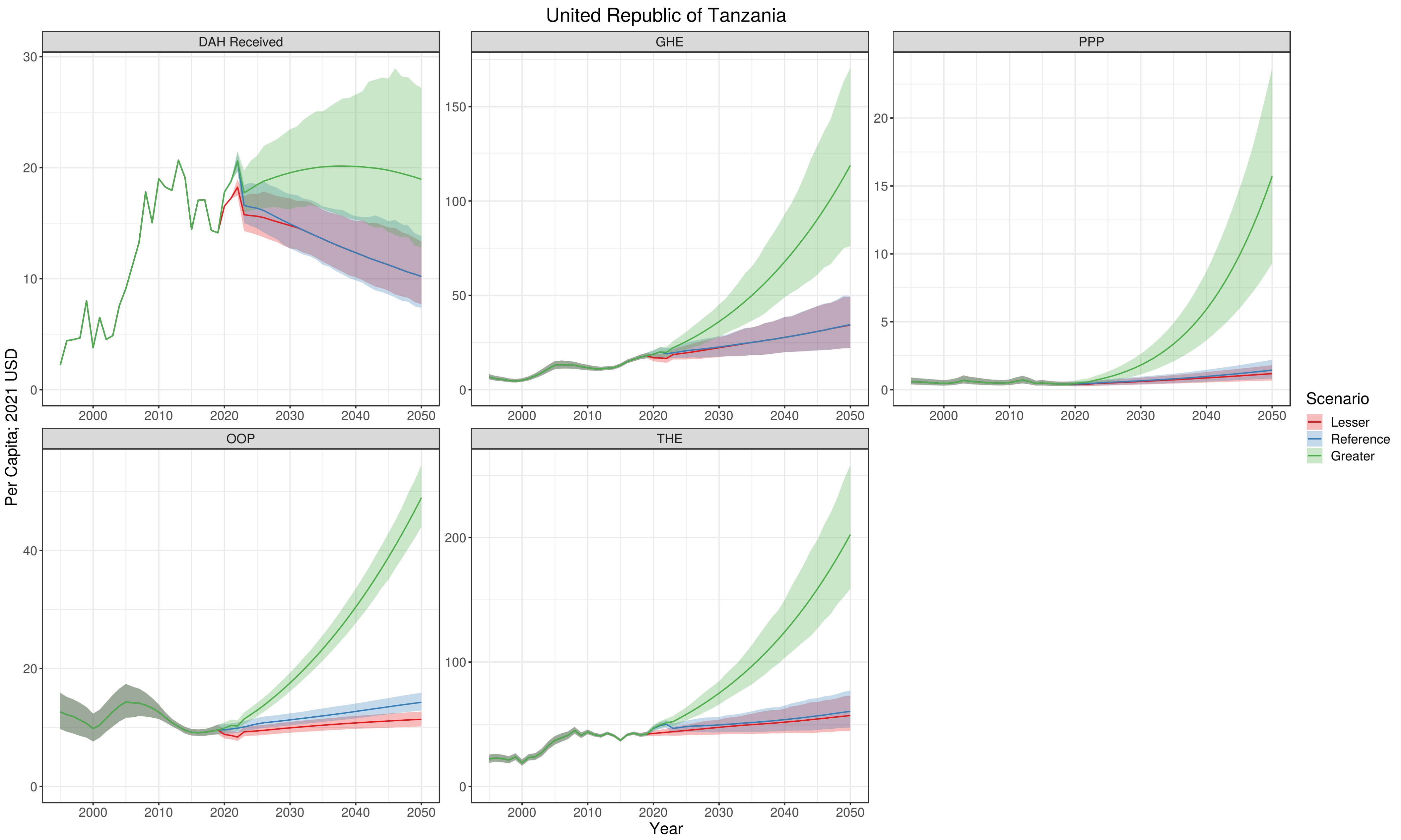

United Republic of Tanzania
DAH Received
GHE
PPP
OOP
THE
Per Capita; 2021 USD
Year
Scenario
Lesser
Reference
Greater
30
20
10
0
150
100
50
15
5
40
200
2000
2010
2020
2030
2040
2050

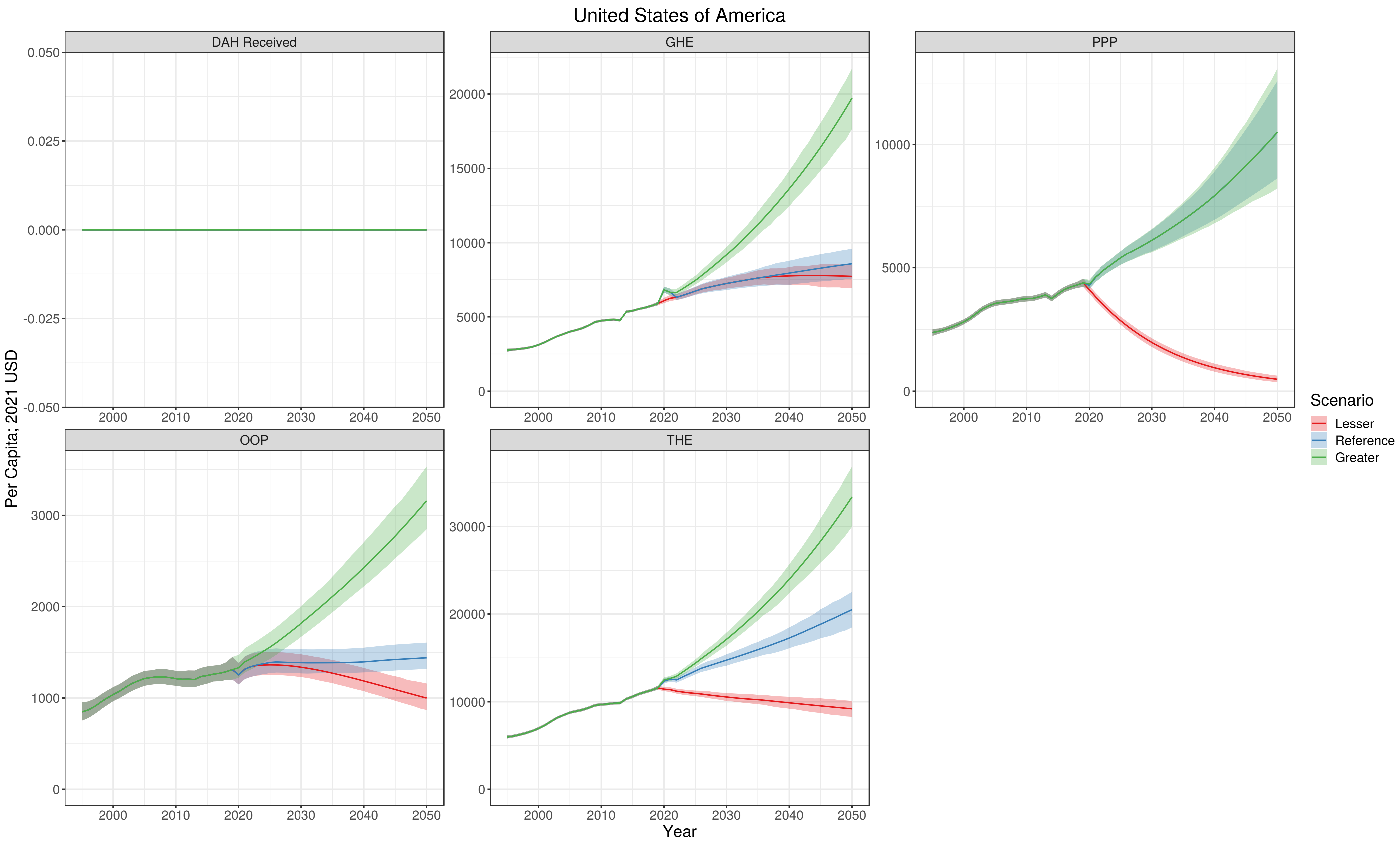

United States of America
DAH Received
GHE
PPP
OOP
THE
Per Capita; 2021 USD
Year
Scenario
Lesser
Reference
Greater
0.050
0.025
0.000
-0.025
-0.050
20000
15000
10000
5000
0
3000
2000
1000
30000
2000
2010
2020
2030
2040
2050

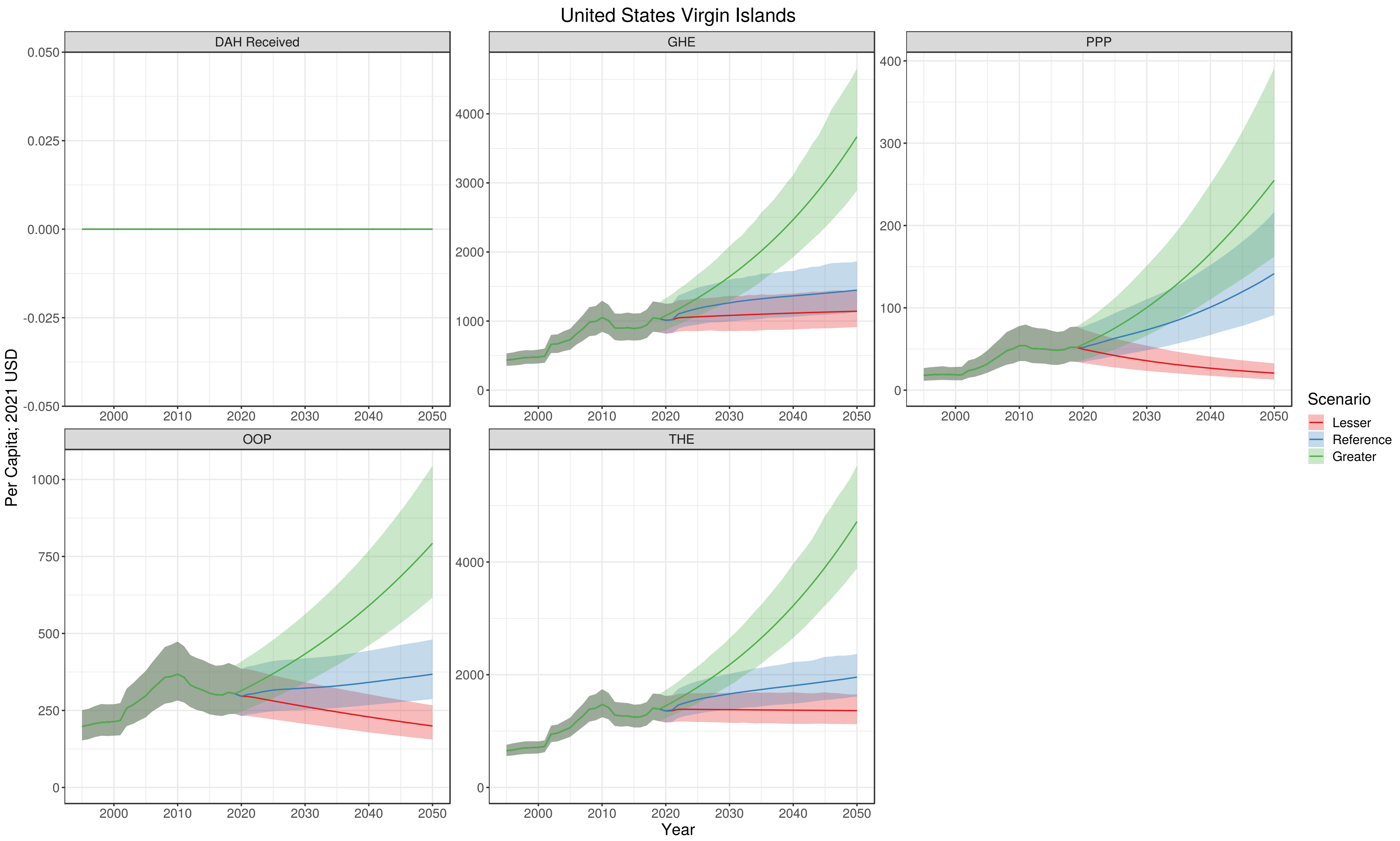

United States Virgin Islands
DAH Received
GHE
PPP
OOP
THE
Per Capita; 2021 USD
Year
Scenario
Lesser
Reference
Greater
0.050
0.025
0.000
-0.025
-0.050
0
1000
2000
3000
4000
100
200
300
400
250
500
750
1000
2000
2010
2020
2030
2040
2050

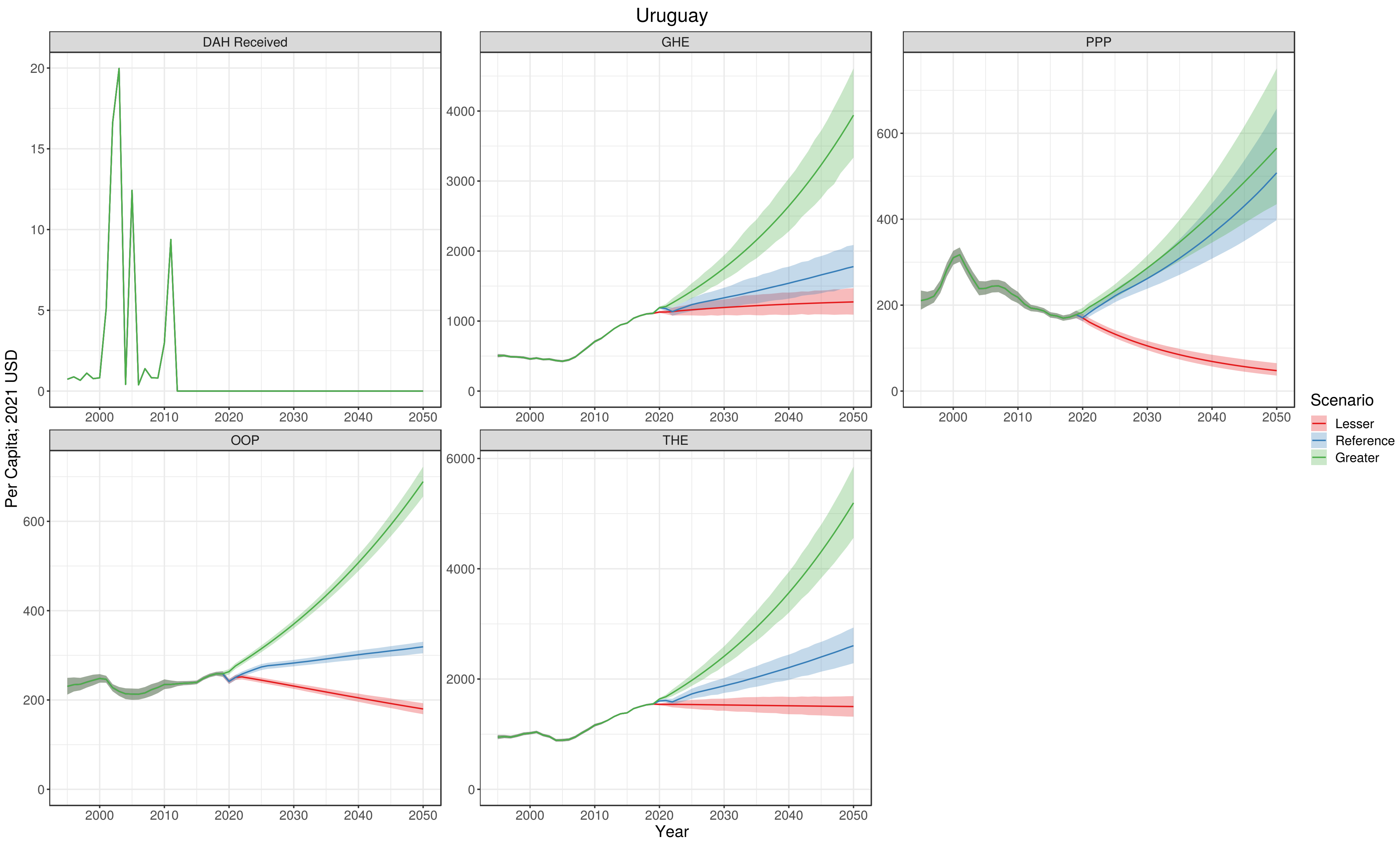

Uruguay
DAH Received
GHE
PPP
OOP
THE
Per Capita; 2021 USD
Year
Scenario
Lesser
Reference
Greater

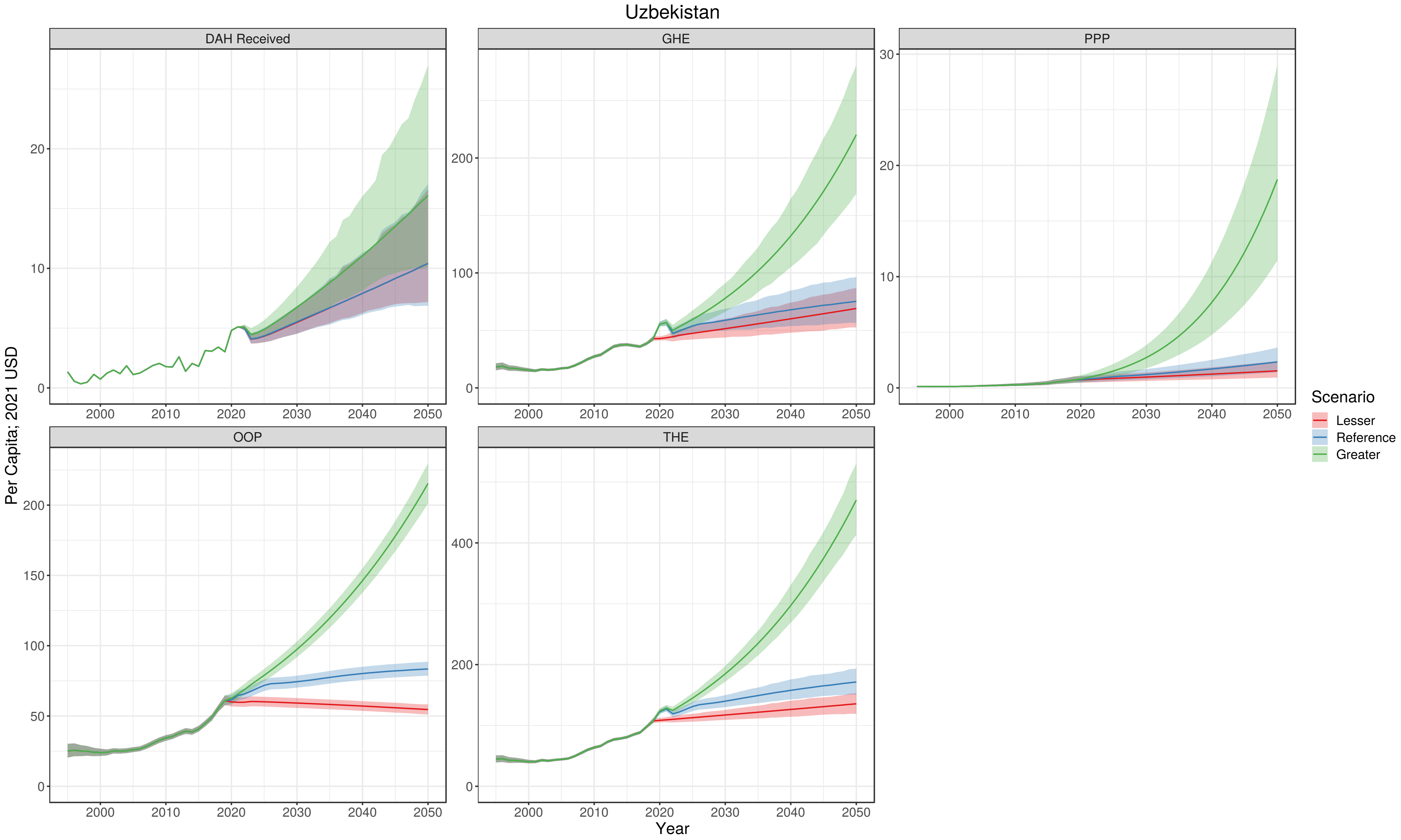

Uzbekistan
DAH Received
GHE
PPP
OOP
THE
Per Capita; 2021 USD
Year
Scenario
Lesser
Reference
Greater
0
10
20
100
200
30
50
150
400
2000
2010
2020
2030
2040
2050

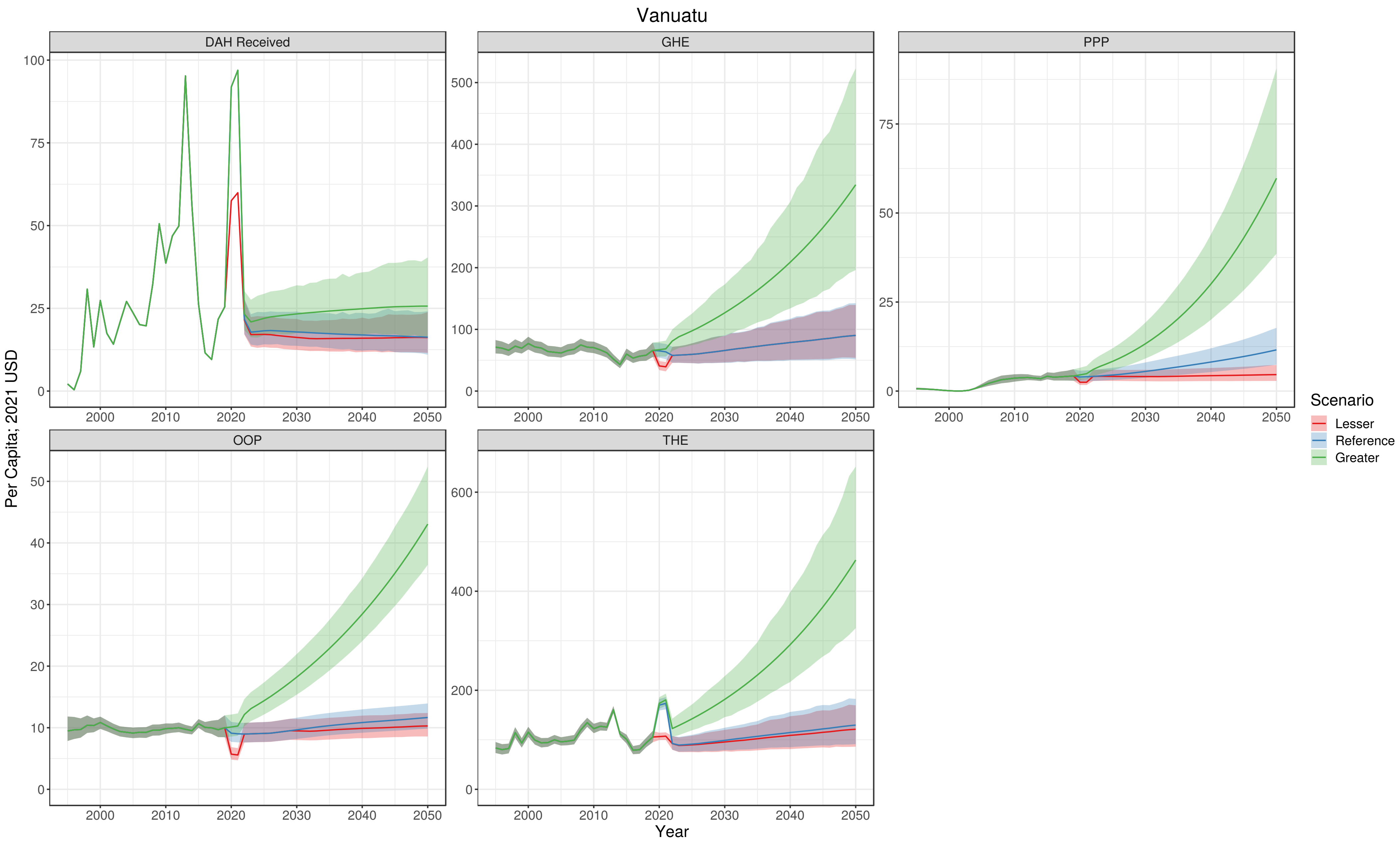

Vanuatu
DAH Received
GHE
PPP
OOP
THE
Per Capita; 2021 USD
Year
Scenario
Lesser
Reference
Greater

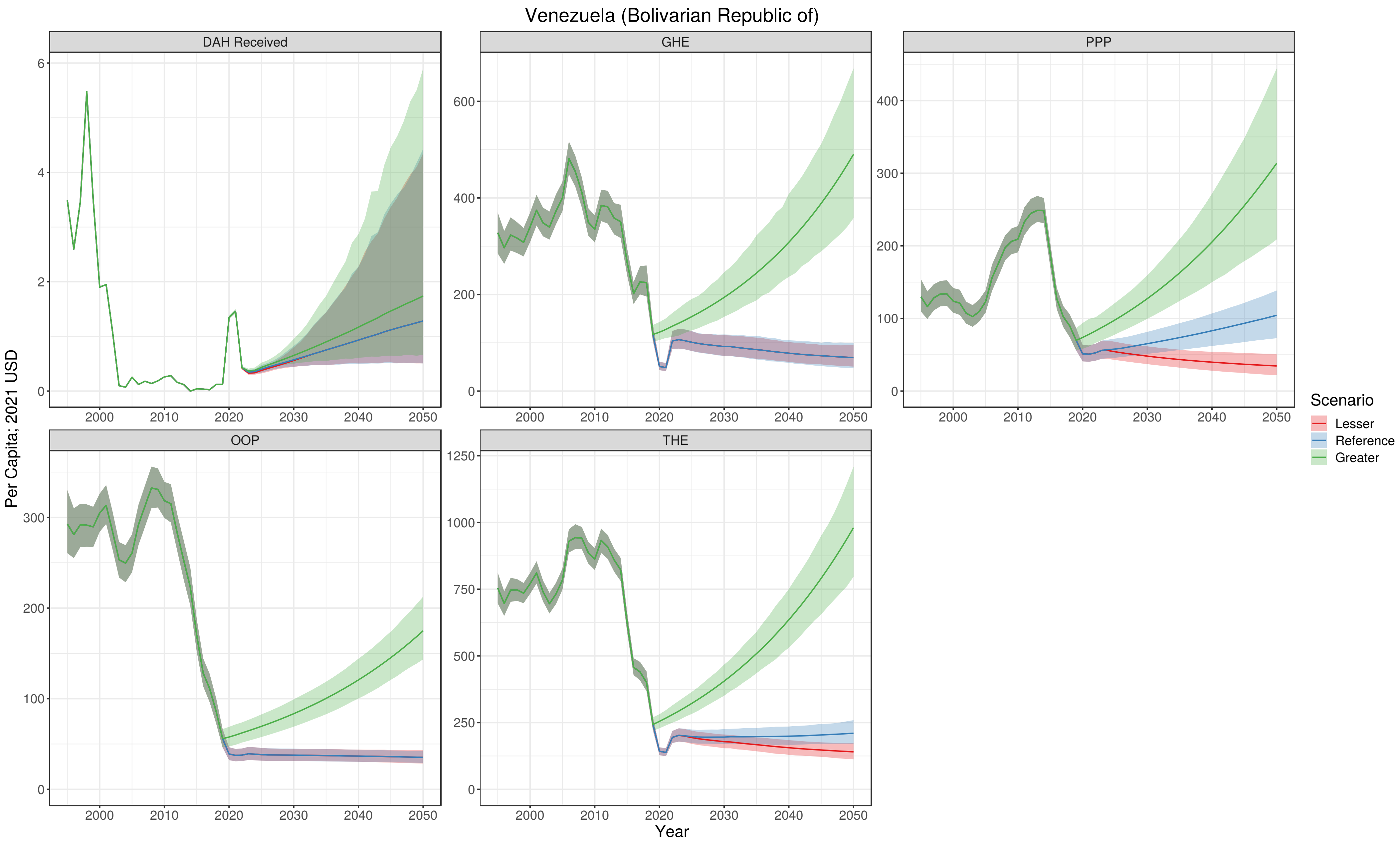

Venezuela (Bolivarian Republic of)
DAH Received
GHE
PPP
OOP
THE
Per Capita; 2021 USD
Year
Scenario
Lesser
Reference
Greater

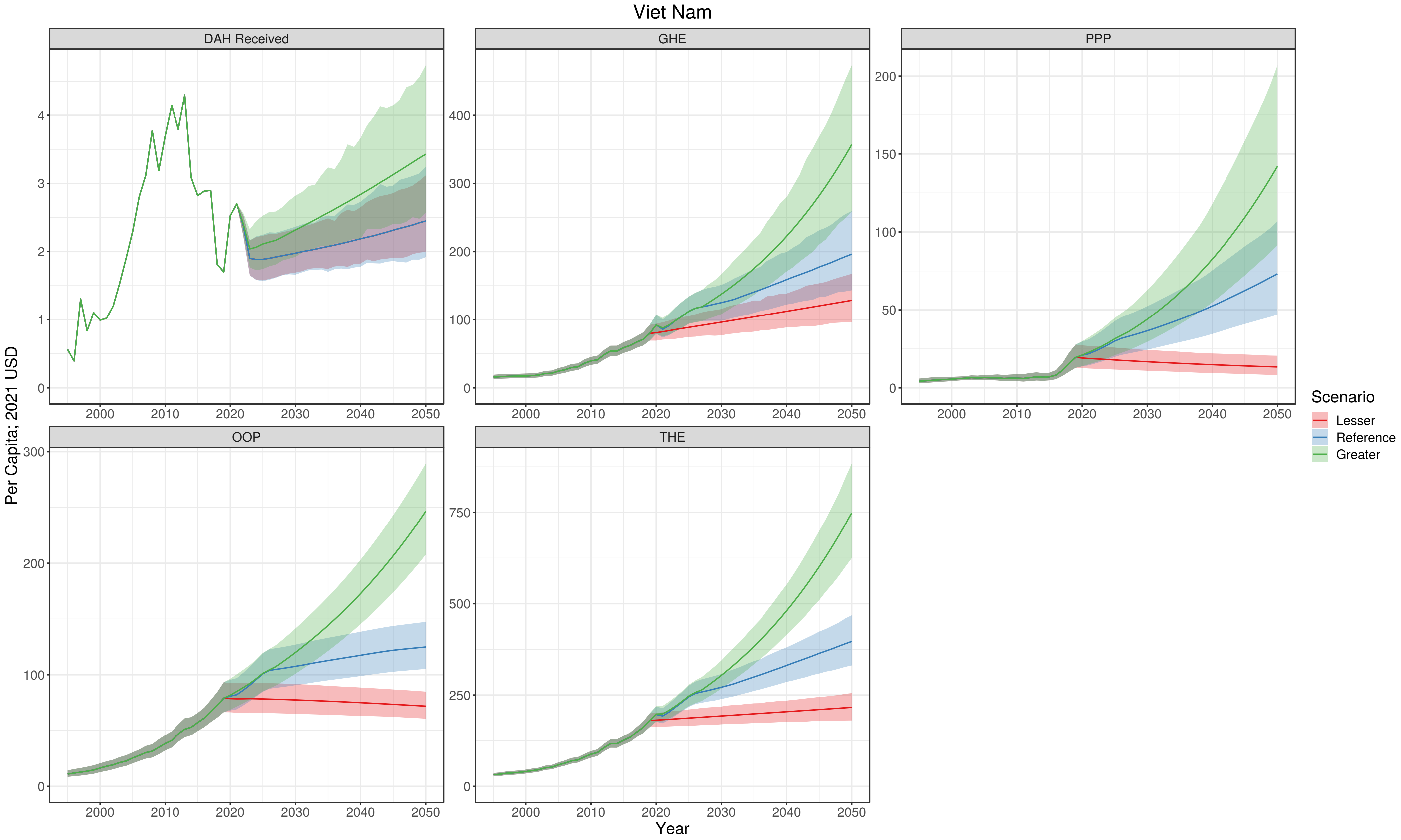

Viet Nam
DAH Received
GHE
PPP
OOP
THE
Per Capita; 2021 USD
Year
2000
2010
2020
2030
2040
2050
0
1
2
3
4
100
200
300
400
50
150
250
500
750
Scenario
Lesser
Reference
Greater

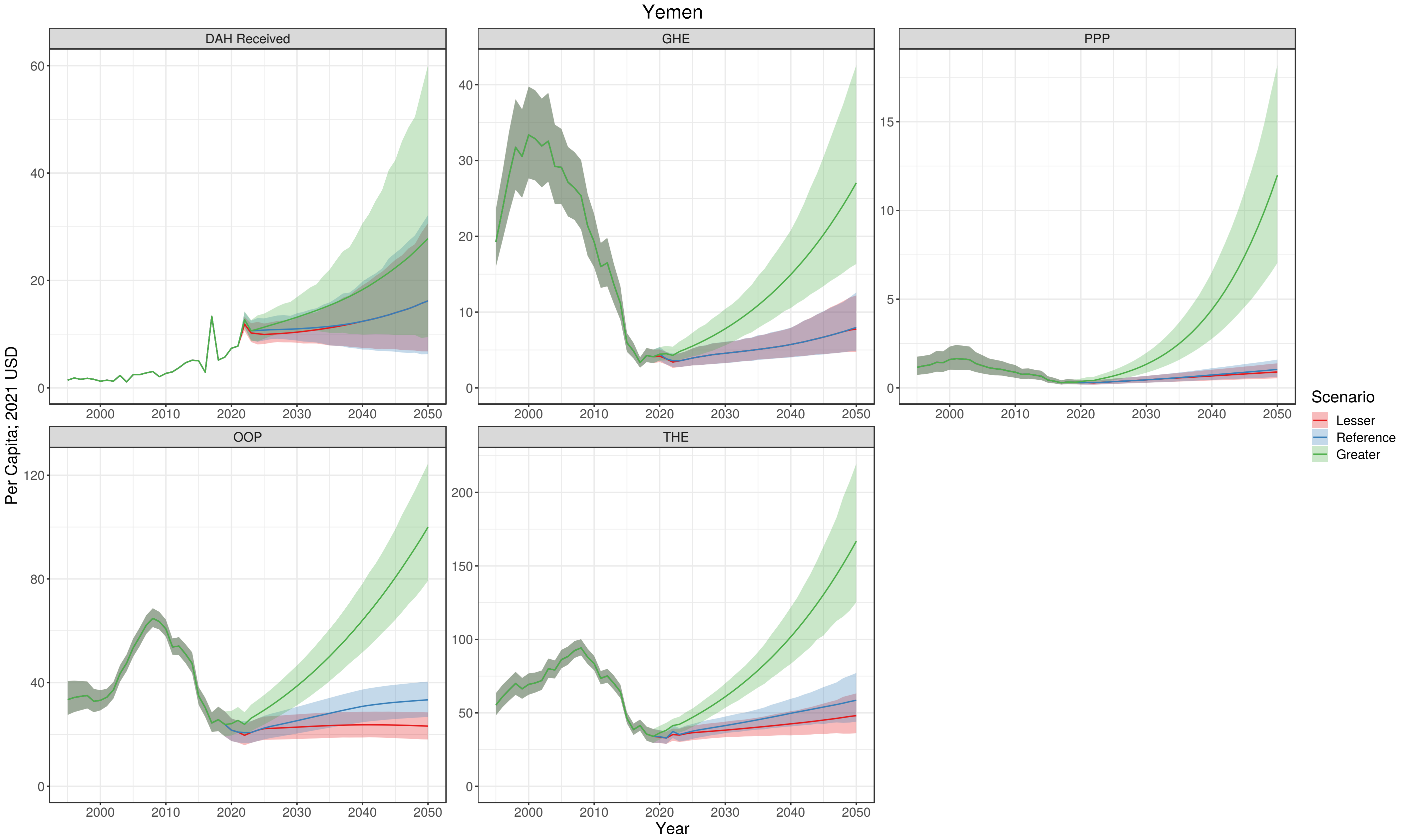
Yemen
DAH Received
GHE
PPP
OOP
THE
Per Capita; 2021 USD
Year
Scenario
Lesser
Reference
Greater
0
20
40
60
10
30
5
15
80
120
50
100
150
200
2000
2010
2020
2030
2040
2050

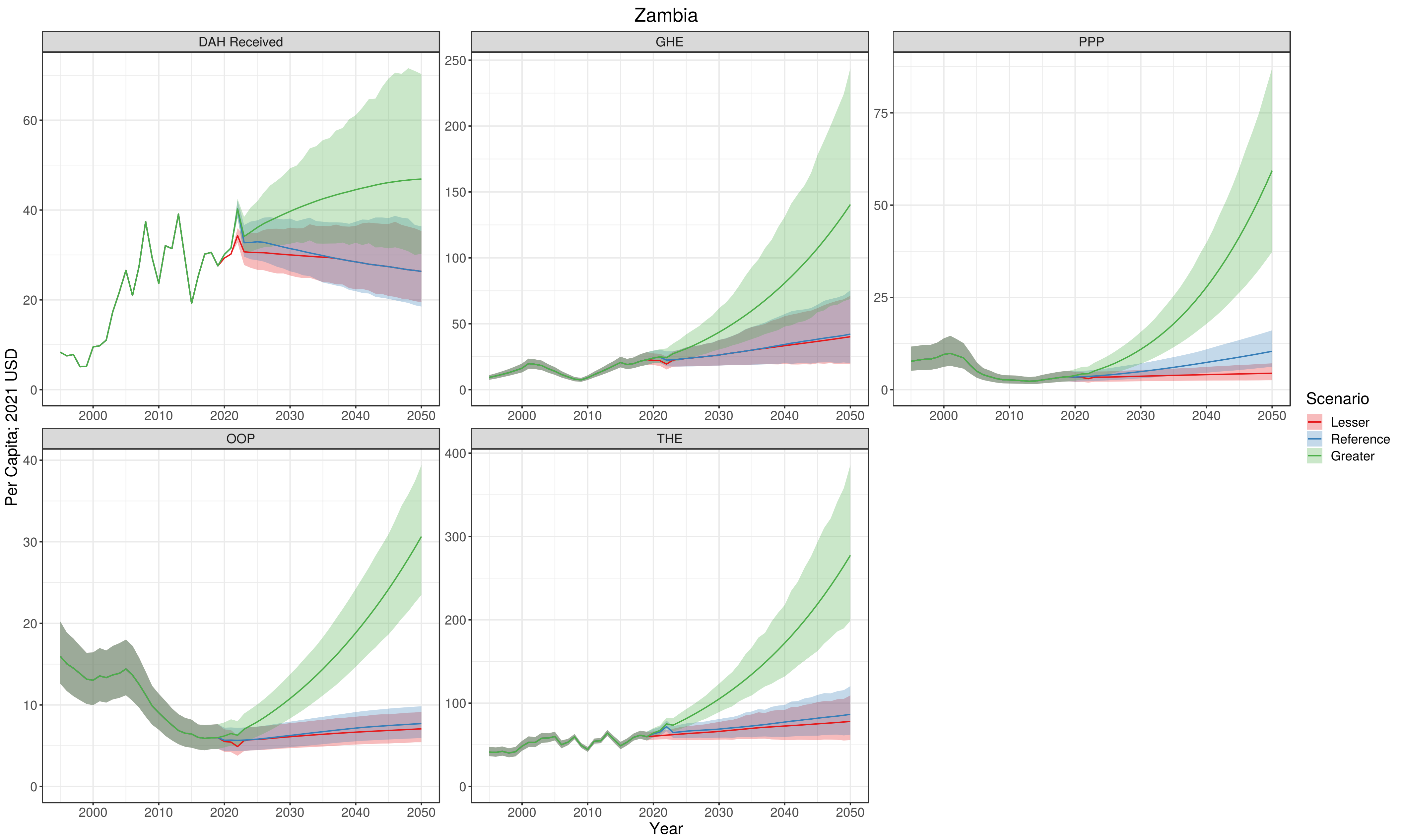

Zambia
DAH Received
GHE
PPP
OOP
THE
Per Capita; 2021 USD
Year
Scenario
Lesser
Reference
Greater

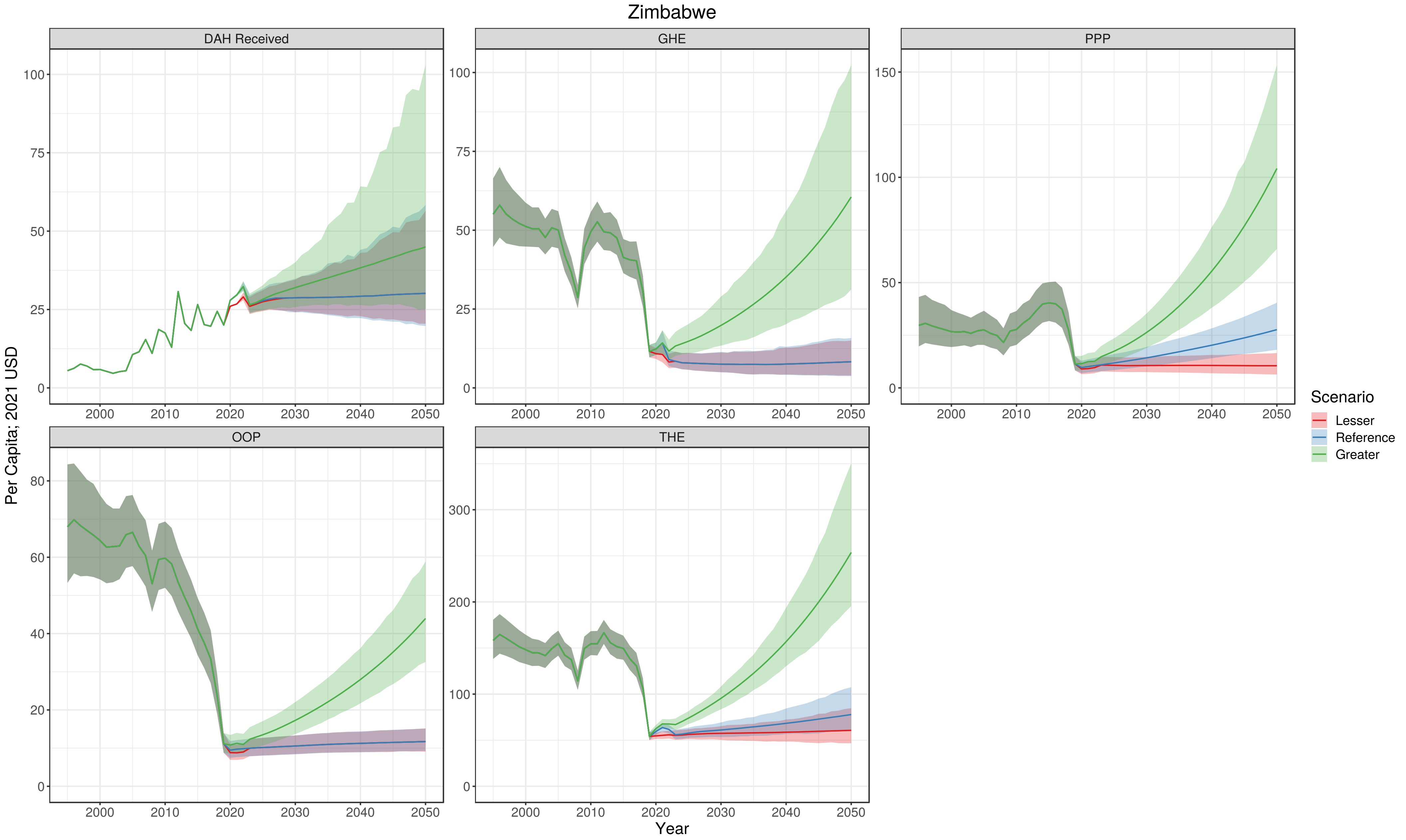

Zimbabwe
DAH Received
GHE
PPP
OOP
THE
Per Capita; 2021 USD
Year
Scenario
Lesser
Reference
Greater

Author Contributions:

Managing the estimation or publications process

Angela E Micah, Simon I Hay, Golsum Tsakalos, Christopher JL Murray, and Joseph L Dieleman.

Writing the first draft of the manuscript

Angela E Micah and Joseph L Dieleman.

Primary responsibility for applying analytical methods to produce estimates

Angela E Micah, Kayleigh Bhangdia, Ian E Cogswell, Dylan Lasher, Brendan Lidral-Porter, Emilie R Maddison, Trang Nguyen, Nishali Patel, Paola Pedroza, Juan Solorio, Hayley N Stutzman, Yifeng Wang, Wesley Warriner, Yingxi Zhao, Bianca S Zlavog, and Joseph L Dieleman.

Primary responsibility for seeking, cataloguing, extracting, or cleaning data; designing or coding figures and tables

Kayleigh Bhangdia, Ian E Cogswell, Dylan Lasher, Brendan Lidral-Porter, Emilie R Maddison, Trang Nguyen, Nishali Patel, Paola Pedroza, Juan Solorio, Hayley N Stutzman, Yifeng Wang, Wesley Warriner, Yingxi Zhao, and Bianca S Zlavog.

Providing data or critical feedback on data sources

Angela E Micah, Kayleigh Bhangdia, Ian E Cogswell, Dylan Lasher, Brendan Lidral-Porter, Emilie R Maddison, Trang Nguyen, Nishali Patel, Paola Pedroza, Juan Solorio, Hayley N Stutzman, Yifeng Wang, Wesley Warriner, Yingxi Zhao, Bianca S Zlavog, Cristiana Abbafati, Jaffar Abbas, Mohsen Abbasi-Kangevari, Zeinab Abbasi-Kangevari, Michael Abdelmasseh, Deldar Morad Abdulah, Aidin Abedi, Kedir Hussein Abegaz, E S Abhilash, Richard Gyan Aboagye, Hassan Abolhassani, Michael R M Abrigo, Hiwa Abubaker Ali, Eman Abu-Gharbieh, Mohammed Hussien Adem, Muhammad Sohail Afzal, Ali Ahmadi, Haroon Ahmed, Tarik Ahmed Rashid, Budi Aji, Hossein Akbarialiabad, Yibeltal Akelew, Hanadi Al Hamad, Khurshid Alam, Fahad Mashhour Alanezi, Turki M Alanzi, Mohammed Khaled Al-Hanawi, Robert Kaba Alhassan, Syed Mohamed Aljunid, Sami Almustanyir, Rajaa M Al-Raddadi, Nelson Alvis-Guzman, Nelson J Alvis-Zakzuk, Azmeraw T Amare, Edward Kwabena Ameyaw, Mostafa Amini-Rarani, Hubert Amu, Robert Ancuceanu, Tudorel Andrei, Sumadi Lukman Anwar, Francis Appiah, Muhammad Aqeel, Jalal Arabloo, Morteza Arab-Zozani, Aleksandr Y Aravkin, Olatunde Aremu, Raphael Taiwo Aruleba, Seyyed Shamsadin Athari, Leticia Avila-Burgos, Martin Amogre Ayanore, Samad Azari, Atif Amin Baig, Abere Tilahun Bantie, Amadou Barrow, Pritish Baskaran, Sanjay Basu, Abdul-Monim Mohammad Batiha, Bernhard T Baune, Zombor Berezvai, Nikha Bhardwaj, Pankaj Bhardwaj, Sonu Bhaskar, Micheal Kofi Boachie, Virginia Bodolica, João Silva Botelho Botelho, Dejana Braithwaite, Nicholas J K Breitborde, Reinhard Busse, Lucero Cahuana-Hurtado, Ferrán Catalá-López, Collins Chansa, Jaykaran Charan, Vijay Kumar Chattu, Simiao Chen, Isaac Sunday Chukwu, Omid Dadras, Lalit Dandona, Rakhi Dandona, Abdollah Dargahi, Sisay Abebe Debela, Edgar Denova-Gutiérrez, Belay Desye, Samath Dhamminda Dharmaratne, Nancy Diao, Linh Phuong Doan, Milad Dodangeh, Wendel Mombaque dos Santos, Leila Doshmangir, John Dube, Ebrahim Eini, Maysaa El Sayed Zaki, Maha El Tantawi, Daniel Berhanie Enyew, Sharareh Eskandarieh, Mohamad Ezati Asar, Adeniyi Francis Fagbamigbe, Emerito Jose A Faraon, Ali Fatehizadeh, Hamed Fattahi, Ginenus Fekadu, Florian Fischer, Nataliya A Foigt, Kayode Raphael Fowobaje, Alberto Freitas, Takeshi Fukumoto, Nancy Fullman, Peter Andras Gaal, Amiran Gamkrelidze, M A Garcia-Gordillo,

Mesfin Gebrehiwot, Urge Gerema, Mansour Ghafourifard, Seyyed-Hadi Ghamari, Reza Ghanbari, Ahmad Ghashghaee, Ali Gholamrezanezhad, Mahaveer Golechha, Davide Golinelli, Yitayal Ayalew Goshu, Girma Garedew Goyomsa, Avirup Guha, Damitha Asanga Gunawardane, Bhawna Gupta, Samer Hamidi, Harapan Harapan, Reza Hashempour, Khezar Hayat, Golnaz Heidari, Ileana Heredia-Pi, Claudiu Herteliu, Demisu Zenbaba Heyi, Kamal Hezam, Yuta Hiraike, Mbuzeleni Mbuzeleni Hlongwa, Ramesh Holla, Mohammad Enamul Hoque, Mehdi Hosseinzadeh, Sorin Hostiuc, Salman Hussain, Olayinka Stephen Ilesanmi, Mustapha Immurana, Arnaud Iradukunda, Nahlah Elkudssiah Ismail, Gaetano Isola, Linda Merin J, Mihajlo Jakovljevic, Mahsa Jalili, Manthan Dilipkumar Janodia, Tahereh Javaheri, Sathish Kumar Jayapal, Digisie Mequanint Jemere, Tamas Joo, Nitin Joseph, Jacek Jerzy Jozwiak, Mikk Jürisson, Billingsley Kaambwa, Vidya Kadashetti, Rajendra Kadel, Dler Hussein Kadir, Laleh R Kalankesh, Rajesh Kamath, Himal Kandel, Rami S Kantar, Shama D Karanth, Ibraheem M Karaye, Salah Eddin Karimi, Bekalu Getnet Kassa, Gbenga A Kayode, Leila Keikavoosi-Arani, Vikash Ranjan Keshri, Cumali Keskin, Yousef Saleh Khader, Morteza Abdullatif Khafaie, Himanshu Khajuria, Hamid Reza Khayat Kashani, Zemene Demelash Kifle, Hanna Kim, Jihee Kim, Min Seo Kim, Yun Jin Kim, Adnan Kisa, Farzad Kompani, Soewarta Kosen, Sindhura Lakshmi Koulmane Laxminarayana, Ai Koyanagi, Kewal Krishan, Dian Kusuma, Judit Lám, Demetris Lamnisos, Anders O Larsson, Sang-woong Lee, Shaun Wen Huey Lee, Wei-Chen Lee, Yo Han Lee, Jacopo Lenzi, Lee-Ling Lim, László Lorenzovici, Rafael Lozano, Vanessa Sintra Machado Machado, Farzan Madadizadeh, Mohammed Magdy Abd El Razek, Razzagh Mahmoudi, Azeem Majeed, Mohammad-Reza Malekpour, Ana Laura Manda, Borhan Mansouri, Mohammad Ali Mansournia, Lorenzo Giovanni Mantovani, Carlos Alberto Marrugo Arnedo, Miquel Martorell, Ali Masoud, Elezebeth Mathews, Richard James Maude, Enkeleint A Mechili, Entezar Mehrabi Nasab, José João João Mendes Mendes, Atte Meretoja, Tuomo J Meretoja, Mohamed Kamal Mesregah, Tomislav Mestrovic, Andreea Mirica, Erkin M Mirrakhimov, Mizan Kiros Mirutse, Moonis Mirza, Mohammad Mirza-Aghazadeh-Attari, Awoke Misganaw, Marcello Moccia, Javad Moghadasi, Esmaeil Mohammadi, Mokhtar Mohammadi, Abdollah Mohammadian-Hafshejani, Marita Mohammadshahi, Shafiu Mohammed, Mohammad Mohseni, Ali H Mokdad, Lorenzo Monasta, Elias Mossialos, Ebrahim Mostafavi, Haleh Mousavi Isfahani, Christine Mpundu-Kaambwa, Shruti Murthy, Saravanan Muthupandian, Ahamarshan Jayaraman Nagarajan, Kovin S Naidoo, Mukhammad David Naimzada, Vinay Nangia, Atta Abbas Naqvi, Biswa Prakash Nayak, Rawlance Ndejjo, Trang Huyen Nguyen, Nafise Noroozi, Jean Jacques Noubiap, Khan M Nuruzzaman, Chimezie Igwegbe Nzoputam, Ogochukwu Janet Nzoputam, Bogdan Oancea, Felix Chukwudi Abrahams Obi, Abiola Ogunkoya, In-Hwan Oh, Osaretin Christabel Okonji, Andrew T Olagunju, Tinuke O Olagunju, Babayemi Oluwaseun Olakunde, Ahmed Omar Bali, Obinna E Onwujekwe, John Nelson Opio, Adrian Otoiu, Nikita Otstavnov, Stanislav S Otstavnov, Mayowa O Owolabi, Tamás Palicz, Raffaele Palladino, Adrian Pana, Tarang Parekh, Deepak Kumar Pasupula, Jay Patel, George C Patton, Uttam Paudel, Mihaela Paun, Shrikant Pawar, Simone Perna, Navaraj Perumalsamy, Ionela-Roxana Petcu, Zahra Zahid Piracha, Mohsen Poursadeqiyan, Naeimeh Pourtaheri, Sergio I Prada, Sima Rafiei, Pankaja Raghav Raghav, Fakher Rahim, Mohammad Hifz Ur Rahman, Mosiur Rahman, Amir Masoud Rahmani, Chhabi Lal Ranabhat, Temam Beshir Raru, Sina Rashedi, Mohammad-Mahdi Rashidi, Ramin Ravangard, Salman Rawaf, Reza Rawassizadeh, Elrashdy Moustafa Mohamed Redwan, Robert C Reiner Jr., Andre M N Renzaho, Maryam Rezaei, Nazila Rezaei, Mavra A Riaz, Jefferson Antonio Buendia Rodriguez, Aly M A Saad, Basema Saddik, Saeid Sadeghian, Mohammad Reza Saeb, Umar Saeed, Maitreyi Sahu, Morteza Saki, Payman Salamati, Hedayat Salari, Sana Salehi, Abdallah M Samy, Juan Sanabria, Francesco Sanmarchi, João Vasco Santos, Milena M Santric-Milicevic, Bruno Piassi Sao Jose, Yaser Sarikhani, Brijesh Sathian, Maheswar Satpathy, Miloje Savic, Yaser Sayadi, Falk Schwendicke,

Subramanian Senthilkumaran, Sadaf G Sepanlou, Edson Serván-Mori, Naomi Setshegetso, Allen Seylani, Saeed Shahabi, Masood Ali Shaikh, Murad Ziyaudinovich Shakhmardanov, Mohd Shanawaz, Mequannent Melaku Sharew Sharew, Nigussie Tadesse Sharew, Rajesh Sharma, Maryam Shayan, Aziz Sheikh, Suchitra M Shenoy, Adithi Shetty, Pavanchand H Shetty, K M Shivakumar, Luís Manuel Lopes Rodrigues Silva, Wudneh Simegn, Jasvinder A Singh, Kuldeep Singh, Natia Skhvitaridze, Valentin Yurievich Skryabin, Anna Aleksandrovna Skryabina, Bogdan Socea, Yonatan Solomon, Suhang Song, Simona Cătălina Ștefan, Muhammad Suleman, Rafael Tabarés-Seisdedos, Nathan Y Tat, Vivian Y Tat, Belay Negash Tefera, Ales Tichopad, Ruoyan Tobe-Gai, Marcos Roberto Tovani-Palone, Lorainne Tudor Car, Derara Girma Tufa, Tommi Juhani Vasankari, Milena Vasic, Dominique Vervoort, Vasily Vlassov, Bay Vo, Linh Gia Vu, Yasir Waheed, Richard G Wamai, Cong Wang, Gizachew Tadesse Wassie, Nuwan Darshana Wickramasinghe, Sanni Yaya, Arzu Yigit, Vahit Yiğit, Naohiro Yonemoto, Mustafa Z Younis, Chuanhua Yu, Ismaeel Yunusa, Leila Zaki, Burhan Abdullah Zaman, Alireza Zangeneh, Ali Zare Dehnavi, Mikhail Sergeevich Zastrozhin, Wu Zeng, Zhi-Jiang Zhang, Liesl J Zuhlke, Yves Miel H Zuniga, and Joseph L Dieleman.

Developing methods or computational machinery

Angela E Micah, Kayleigh Bhangdia, Ian E Cogswell, Dylan Lasher, Brendan Lidral-Porter, Emilie R Maddison, Trang Nguyen, Nishali Patel, Paola Pedroza, Juan Solorio, Hayley N Stutzman, Yifeng Wang, Wesley Warriner, Yingxi Zhao, Bianca S Zlavog, and Joseph L Dieleman.

Providing critical feedback on methods or results

Angela E Micah, Kayleigh Bhangdia, Ian E Cogswell, Dylan Lasher, Brendan Lidral-Porter, Emilie R Maddison, Trang Nguyen, Nishali Patel, Paola Pedroza, Juan Solorio, Hayley N Stutzman, Yifeng Wang, Wesley Warriner, Yingxi Zhao, Bianca S Zlavog, Cristiana Abbafati, Jaffar Abbas, Mohsen Abbasi-Kangevari, Zeinab Abbasi-Kangevari, Michael Abdelmasseh, Deldar Morad Abdulah, Aidin Abedi, Kedir Hussein Abegaz, E S Abhilash, Richard Gyan Aboagye, Hassan Abolhassani, Michael R M Abrigo, Hiwa Abubaker Ali, Eman Abu-Gharbieh, Mohammed Hussien Adem, Muhammad Sohail Afzal, Ali Ahmadi, Haroon Ahmed, Tarik Ahmed Rashid, Budi Aji, Hossein Akbarialiabad, Yibeltal Akelew, Hanadi Al Hamad, Khurshid Alam, Fahad Mashhour Alanezi, Turki M Alanzi, Mohammed Khaled Al-Hanawi, Robert Kaba Alhassan, Syed Mohamed Aljunid, Sami Almustanyir, Rajaa M Al-Raddadi, Nelson Alvis-Guzman, Nelson J Alvis-Zakzuk, Azmeraw T Amare, Edward Kwabena Ameyaw, Mostafa Amini-Rarani, Hubert Amu, Robert Ancuceanu, Tudorel Andrei, Sumadi Lukman Anwar, Francis Appiah, Muhammad Aqeel, Jalal Arabloo, Morteza Arab-Zozani, Aleksandr Y Aravkin, Olatunde Aremu, Raphael Taiwo Aruleba, Seyyed Shamsadin Athari, Leticia Avila-Burgos, Martin Amogre Ayanore, Samad Azari, Atif Amin Baig, Abere Tilahun Bantie, Amadou Barrow, Pritish Baskaran, Sanjay Basu, Abdul-Monim Mohammad Batiha, Bernhard T Baune, Zombor Berezvai, Nikha Bhardwaj, Pankaj Bhardwaj, Sonu Bhaskar, Micheal Kofi Boachie, Virginia Bodolica, João Silva Botelho Botelho, Dejana Braithwaite, Nicholas J K Breitborde, Reinhard Busse, Lucero Cahuana-Hurtado, Ferrán Catalá-López, Collins Chansa, Jaykaran Charan, Vijay Kumar Chattu, Simiao Chen, Isaac Sunday Chukwu, Omid Dadras, Lalit Dandona, Rakhi Dandona, Abdollah Dargahi, Sisay Abebe Debela, Edgar Denova-Gutiérrez, Belay Desye, Samath Dhamminda Dharmaratne, Nancy Diao, Linh Phuong Doan, Milad Dodangeh, Wendel Mombaque dos Santos, Leila Doshmangir, John Dube, Ebrahim Eini, Maysaa El Sayed Zaki, Maha El Tantawi, Daniel Berhanie Enyew, Sharareh Eskandarieh, Mohamad Ezati Asar, Adeniyi Francis Fagbamigbe, Emerito Jose A Faraon, Fatehizadeh, Hamed Fattahi, Ginenus Fekadu, Florian Fischer, Nataliya A Foigt, Kayode Raphael Fowobaje, Alberto

Freitas, Takeshi Fukumoto, Nancy Fullman, Peter Andras Gaal, Amiran Gamkrelidze, M A Garcia-Gordillo, Mesfin Gebrehiwot, Urge Gerema, Mansour Ghafourifard, Seyyed-Hadi Ghamari, Reza Ghanbari, Ahmad Ghashghaee, Ali Gholamrezanezhad, Mahaveer Golechha, Davide Golinelli, Yitayal Ayalew Goshu, Girma Garedew Goyomsa, Avirup Guha, Damitha Asanga Gunawardane, Bhawna Gupta, Samer Hamidi, Harapan Harapan, Reza Hashempour, Khezar Hayat, Golnaz Heidari, Ileana Heredia-Pi, Claudiu Herteliu, Demisu Zenbaba Heyi, Kamal Hezam, Yuta Hiraike, Mbuzeleni Mbuzeleni Hlongwa, Ramesh Holla, Mohammad Enamul Hoque, Mehdi Hosseinzadeh, Sorin Hostiuc, Salman Hussain, Olayinka Stephen Ilesanmi, Mustapha Immurana, Arnaud Iradukunda, Nahlah Elkudssiah Ismail, Gaetano Isola, Linda Merin J, Mihajlo Jakovljevic, Mahsa Jalili, Manthan Dilipkumar Janodia, Tahereh Javaheri, Sathish Kumar Jayapal, Digisie Mequanint Jemere, Tamas Joo, Nitin Joseph, Jacek Jerzy Jozwiak, Mikk Jürisson, Billingsley Kaambwa, Vidya Kadashetti, Rajendra Kadel, Dler Hussein Kadir, Laleh R Kalankesh, Rajesh Kamath, Himal Kandel, Rami S Kantar, Shama D Karanth, Ibraheem M Karaye, Salah Eddin Karimi, Bekalu Getnet Kassa, Gbenga A Kayode, Leila Keikavoosi-Arani, Vikash Ranjan Keshri, Cumali Keskin, Yousef Saleh Khader, Morteza Abdullatif Khafaie, Himanshu Khajuria, Hamid Reza Khayat Kashani, Zemene Demelash Kifle, Hanna Kim, Jihee Kim, Min Seo Kim, Yun Jin Kim, Adnan Kisa, Stefan Kohler, Farzad Kompani, Soewarta Kosen, Sindhura Lakshmi Koulmane Laxminarayana, Ai Koyanagi, Kewal Krishan, Dian Kusuma, Judit Lám, Demetris Lamnisos, Anders O Larsson, Sang-woong Lee, Shaun Wen Huey Lee, Wei-Chen Lee, Yo Han Lee, Jacopo Lenzi, Lee-Ling Lim, László Lorenzovici, Rafael Lozano, Vanessa Sintra Machado Machado, Farzan Madadizadeh, Mohammed Magdy Abd El Razek, Razzagh Mahmoudi, Azeem Majeed, Mohammad-Reza Malekpour, Ana Laura Manda, Borhan Mansouri, Mohammad Ali Mansournia, Lorenzo Giovanni Mantovani, Carlos Alberto Marrugo Arnedo, Miquel Martorell, Ali Masoud, Elezebeth Mathews, Richard James Maude, Enkeleint A Mechili, Entezar Mehrabi Nasab, José João João Mendes Mendes, Atte Meretoja, Tuomo J Meretoja, Mohamed Kamal Mesregah, Tomislav Mestrovic, Andreea Mirica, Erkin M Mirrakhimov, Mizan Kiros Mirutse, Moonis Mirza, Mohammad Mirza-Aghazadeh-Attari, Awoke Misganaw, Marcello Moccia, Javad Moghadasi, Esmaeil Mohammadi, Mokhtar Mohammadi, Abdollah Mohammadian-Hafshejani, Marita Mohammadshahi, Shafiu Mohammed, Mohammad Mohseni, Ali H Mokdad, Lorenzo Monasta, Elias Mossialos, Ebrahim Mostafavi, Haleh Mousavi Isfahani, Christine Mpundu-Kaambwa, Shruti Murthy, Saravanan Muthupandian, Ahamarshan Jayaraman Nagarajan, Kovin S Naidoo, Mukhammad David Naimzada, Vinay Nangia, Atta Abbas Naqvi, Biswa Prakash Nayak, Rawlance Ndejjo, Trang Huyen Nguyen, Nafise Noroozi, Jean Jacques Noubiap, Khan M Nuruzzaman, Chimezie Igwegbe Nzoputam, Ogochukwu Janet Nzoputam, Bogdan Oancea, Felix Chukwudi Abrahams Obi, Abiola Ogunkoya, In-Hwan Oh, Osaretin Christabel Okonji, Andrew T Olagunju, Tinuke O Olagunju, Babayemi Oluwaseun Olakunde, Ahmed Omar Bali, Obinna E Onwujekwe, John Nelson Opio, Adrian Otoiu, Nikita Otstavnov, Stanislav S Otstavnov, Mayowa O Owolabi, Tamás Palicz, Raffaele Palladino, Adrian Pana, Tarang Parekh, Deepak Kumar Pasupula, Jay Patel, George C Patton, Uttam Paudel, Mihaela Paun, Shrikant Pawar, Simone Perna, Navaraj Perumalsamy, Ionela-Roxana Petcu, Zahra Zahid Piracha, Mohsen Poursadeqiyan, Naeimeh Pourtaheri, Sergio I Prada, Sima Rafiei, Pankaja Raghav Raghav, Fakher Rahim, Mohammad Hifz Ur Rahman, Mosiur Rahman, Amir Masoud Rahmani, Chhabi Lal Ranabhat, Temam Beshir Raru, Sina Rashedi, Mohammad-Mahdi Rashidi, Ramin Ravangard, Salman Rawaf, Reza Rawassizadeh, Elrashdy Moustafa Mohamed Redwan, Robert C Reiner Jr., Andre M N Renzaho, Maryam Rezaei, Nazila Rezaei, Mavra A Riaz, Jefferson Antonio Buendia Rodriguez, Aly M A Saad, Basema Saddik, Saeid Sadeghian, Mohammad Reza Saeb, Umar Saeed, Maitreyi Sahu, Morteza Saki, Payman Salamati, Hedayat Salari, Sana Salehi, Abdallah M Samy, Juan Sanabria, Francesco Sanmarchi, João Vasco Santos, Milena M

Santric-Milicevic, Bruno Piassi Sao Jose, Yaser Sarikhani, Brijesh Sathian, Maheswar Satpathy, Miloje Savic, Yaser Sayadi, Falk Schwendicke, Subramanian Senthilkumaran, Sadaf G Sepanlou, Edson Serván-Mori, Naomi Setshegetso, Allen Seylani, Saeed Shahabi, Masood Ali Shaikh, Murad Ziyaudinovich Shakhmardanov, Mohd Shanawaz, Mequannent Melaku Sharew Sharew, Nigussie Tadesse Sharew, Rajesh Sharma, Maryam Shayan, Aziz Sheikh, Suchitra M Shenoy, Adithi Shetty, Pavanchand H Shetty, K M Shivakumar, Luís Manuel Lopes Rodrigues Silva, Wudneh Simegn, Jasvinder A Singh, Kuldeep Singh, Natia Skhvitaridze, Valentin Yurievich Skryabin, Anna Aleksandrovna Skryabina, Bogdan Socea, Yonatan Solomon, Suhang Song, Simona Cătălina Ștefan, Muhammad Suleman, Rafael Tabarés-Seisdedos, Nathan Y Tat, Vivian Y Tat, Belay Negash Tefera, Ales Tichopad, Ruoyan Tobe-Gai, Marcos Roberto Tovani-Palone, Lorainne Tudor Car, Derara Girma Tufa, Tommi Juhani Vasankari, Milena Vasic, Dominique Vervoort, Vasily Vlassov, Bay Vo, Linh Gia Vu, Yasir Waheed, Richard G Wamai, Cong Wang, Gizachew Tadesse Wassie, Nuwan Darshana Wickramasinghe, Sanni Yaya, Arzu Yigit, Vahit Yiğit, Naohiro Yonemoto, Mustafa Z Younis, Chuanhua Yu, Ismaeel Yunusa, Leila Zaki, Burhan Abdullah Zaman, Alireza Zangeneh, Ali Zare Dehnavi, Mikhail Sergeevich Zastrozhin, Wu Zeng, Zhi-Jiang Zhang, Liesl J Zuhlke, Yves Miel H Zuniga, and Joseph L Dieleman.

Drafting the work or revising it critically for important intellectual content

Angela E Micah, Kayleigh Bhangdia, Ian E Cogswell, Dylan Lasher, Brendan Lidral-Porter, Emilie R Maddison, Trang Nguyen, Nishali Patel, Paola Pedroza, Juan Solorio, Hayley N Stutzman, Yifeng Wang, Wesley Warriner, Yingxi Zhao, Bianca S Zlavog, and Joseph L Dieleman.

Extracting, cleaning, or cataloging data; designing or coding figures and tables

Kayleigh Bhangdia, Ian E Cogswell, Dylan Lasher, Brendan Lidral-Porter, Emilie R Maddison, Trang Nguyen, Nishali Patel, Paola Pedroza, Juan Solorio, Hayley N Stutzman, Yifeng Wang, Wesley Warriner, Yingxi Zhao, and Bianca S Zlavog.

Managing the overall research enterprise

Angela E Micah, Simon I Hay, Golsum Tsakalos, Christopher J L Murray, and Joseph L Dieleman.
